# 2‑Lithiomethylindoles: Untapped Anions for the Synthesis of 2‑Functionalized Indoles

**DOI:** 10.1021/acs.joc.5c02679

**Published:** 2026-02-23

**Authors:** Sara Gómez-Gil, Marta Solas, Samuel Suárez-Pantiga, Roberto Sanz

**Affiliations:** Área de Química Orgánica, Departamento de Química, Facultad de Ciencias, 16725 Universidad de Burgos, Pza. Misael Bañuelos s/n, 09001, Burgos Spain

## Abstract

Optimal conditions for the metalation via α-lithiation of 1,2-dimethylindoles were established with *n*BuLi at room temperature, providing the most effective protocol. The resulting organolithium reagents bearing the structure of 2-lithiomethylindole were efficiently trapped with diverse electrophiles, including Weinreb amides, ketones, alkyl bromides, and epoxides, affording a broad range of C2-functionalized indoles in good yields. The methodology proved general and was successfully applied to 2-methylindoles bearing different substituents at the benzenoid ring, C3, and on the nitrogen atom, as well as to 2-benzylindoles, giving access to new functionalized indole derivatives. Importantly, deep eutectic solvents enabled these transformations under air- and moisture-tolerant conditions, highlighting their greener and operationally simple character. The synthetic utility of the method was further demonstrated through subsequent derivatizations, exemplified by the two-step preparation of 2,4-disubstituted carbazoles.

## Graphical abstract

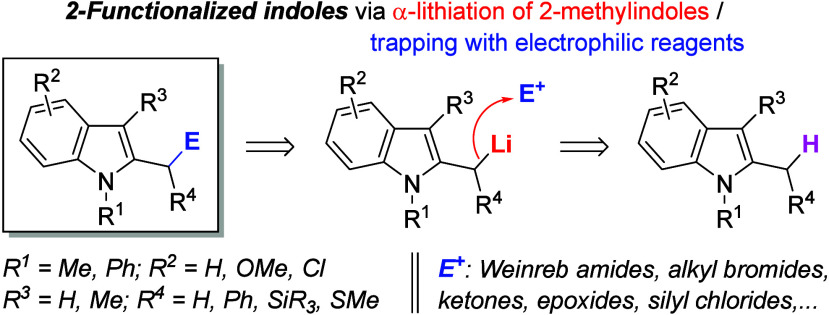

## Introduction

Indole is a ubiquitous heterocyclic core, widely present in natural products and biologically active molecules.^1^ Numerous methods have been developed not only for its synthesis but also for accessing fused indole-containing scaffolds.^2^ In addition, there is continuous interest in the synthesis of indole derivatives through the functionalization of simple and readily available indoles.^3^ Within this context, the selective introduction of functional group-containing substituents at the C2-position is a key transformation for subsequent elaborations, which is typically achieved by Pd-catalyzed cross-coupling reactions of the corresponding 2-halogenated or 2-metalated indoles,^4^ or through transition metal C2–H functionalization which typically requires directing groups or C3-substitution to overcome regioselectivity issues with C3.^5^ Moreover, 2-lithioindoles, accessible either by direct lithiation of the C2–H or by Br–Li exchange,^6^ represent a valuable complementary approach to C2-functionalization. The direct C2-lithiation process has been successfully applied mainly to *N*-methylindoles^7^ or to indoles bearing directing groups, such as sulfonyl or carbamates at the nitrogen atom.^7b,8^

An alternative approach to the synthesis of C2-substituted indoles involves the functionalization of 2-methylindoles. The direct transformation of these readily available indoles is an appealing step-economic approach; however, reported methodologies are scarce and typically limited to a specific type of electrophile, such as the organocatalytic enantioselective reaction of 2,3-dimethylindoles with ethyl 3,3,3-trifluoropyruvate,^9^ the photocatalyzed radical C–H acylation or alkylation to indolyl ketones,^10^ the Cu-catalyzed C2-oxidation of 2,3-disubstituted indoles with BocNHOH,^11^ the electrochemical amination of 1,3-disubstituted-2-methylindoles,^12^ or the TFA-promoted aminomethylation of 1,2,3-trimethylindoles with aminals (Scheme 1a).^13^

**Scheme 1:**
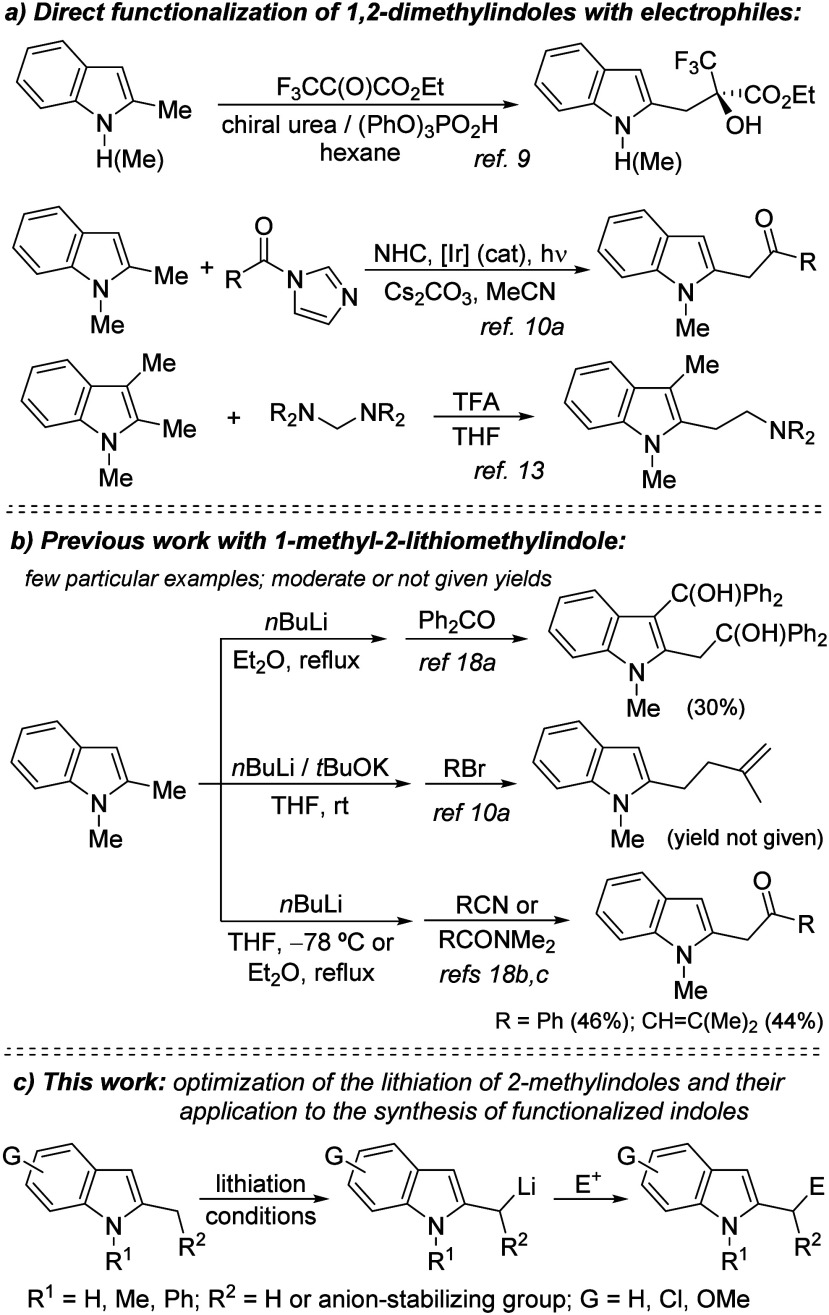
Functionalization of 1,2-Dimethylindoles with Electrophiles and 1-Methyl-2-Lithiomethylindoles (Previous Work and Proposal)

While these and other direct C–H functionalizations at benzylic positions are attractive options, they often display a narrow scope regarding both the substrate and the installed functionality. Owing to the enhanced acidity of benzylic protons, the lithiation/trapping with electrophiles at benzylic positions is a competitive methodology for accessing functionalized (hetero)­aromatic compounds.^14^ The main challenge in this strategy, aside from the inherent chemoselectivity issues associated with organolithiums, is the regioselectivity of the lithiation reaction that can typically be ensured at the benzylic position by using directing groups. This strategy has been extensively applied to the heterobenzylic lithiation of electron-deficient *N*-heterocycles, such as alkylpyridines and different azoles.^15^ However, for electron-rich heterocycles like indoles, these reactions remain considerably underdeveloped. Whereas the conditions for the generation of the C,N-dianion of 2-methylindole^16^ or for the deprotonation of 3-acyl-1,2-dimethyindoles^17^ have been reported, the benzylic lithiation of 1,2-dimethylindole has been scarcely reported in the literature, under different metalation conditions, and has been limited to a very few particular examples with moderate or unreported yields (Scheme 1b).^18^

Building on our experience with the α-lithiation/trapping of benzyl ethers,^19^ we report herein a general, experimentally simple, and selective benzylic lithiation of 1,2-dimethylindoles, combined with their reactivity toward electrophilic reagents, as an efficient approach to access C2-functionalized indole derivatives (Scheme 1c).^20^ More than 50 examples are presented across different 2-methylindoles and a broad range of electrophiles, including their application to the synthesis of carbazoles.

## Results and Discussion

First, we revisited the reaction conditions for the selective α-lithiation of 1,2-dimethylindole (**1a**), since the previously reported conditions were not well-established (see Scheme 1b).^18^ Looking for efficient and convenient conditions for generating 2-lithiomethyl-1-methylindole (**1a-Li**), we selected commercially available organolithium reagents, THF as solvent, and room temperature (Table 1). Using stoichiometric or slight excess amounts of *n*BuLi, as previously reported, led to incomplete lithiation, as determined by subsequent deuteration with MeOD (entries 1 and 2). Gratifyingly, increasing the amount of *n*BuLi up to 1.5 equiv gave rise to complete lithiation (entry 3). In addition, we also checked that both *s*BuLi and *t*BuLi resulted in the efficient and selective generation of **1a-Li** (entries 4 and 5).

**Table 1:**
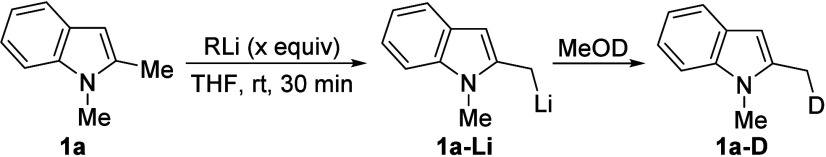
Optimization of the Reaction Conditions for the Formation of 2-Lithiomethyl-1-methylindole (**1a-Li**)­^a^

entry	RLi	x (equiv)	% D^b^	yield **1a-D** (%)^c^
1^c^	*n*BuLi	1	70	88
2	*n*BuLi	1.2	86	91
3	*n*BuLi	1.5	>95	90
4	*s*BuLi	1.5	>95	92
5	*t*BuLi	1.5	>95	91

a Reaction conditions: **1a** (1 mmol) in anhydrous THF (2 mL), rt, 30 min, under N_2_ atmosphere

b Determined by ^1^H NMR.

c Isolated yield referred to **1a**.

Once the conditions for an efficient and selective generation of **1a-Li** are well-established, the electrophile scope of the lithiation/trapping sequence of **1a** was investigated (Scheme 2). First, looking for an efficient access to α-(2-indolyl) ketones, which are valuable indole derivatives for further transformations^21^ that have previously been prepared through different synthetic sequences such as the reactions of 2-lithioindoles with epoxides and subsequent oxidation^22^ or the reduction of nitrostyrenes derived from indole-2-carboxaldehydes,^21^ among others,^23^ Weinreb amides were first tested as electrophiles. In this way, different (*cyclo*)-alkyl and (hetero)­aromatic Weinreb amides led to good yields of the corresponding α-(2-indolyl) ketones **2a**–**e** (Scheme 2a) Then, primary bromides, including allylic, benzylic and acetal-functionalized ones, reacted smoothly with anion **1a-Li** to provide the C2-alkylated products **2f**–**j** in moderate to high yields, containing additional functional groups (Scheme 2b). Notably, this α-lithiation/trapping protocol could be easily scaled up, as demonstrated with the gram synthesis of **2j**, isolated in 74% yield at 10 mmol scale. Other useful electrophilic reagents that would lead to 2-functionalized indoles are epoxides, which were demonstrated to behave as suitable electrophiles leading to 2-(3-hydroxyalkyl)­indole derivative **2k**–**t**. Both monosubstituted and 1,1-disubstituted epoxides afforded the expected products **2k**–**s** in good to high yields, including the gram-scale (10 mmol) synthesis of **2l**. Remarkably, enantiomerically pure (*S*)-propylene oxide provided the chiral hydroxyindole (*S*)-**2l** in high yield. Furthermore, a 1,2-disubstituted epoxide, such as cyclohexene oxide, underwent ring opening with **1a-Li**, producing diastereoisomerically pure 2-hydroxy functionalized indole **2t** (Scheme 2c).

**Scheme 2:**
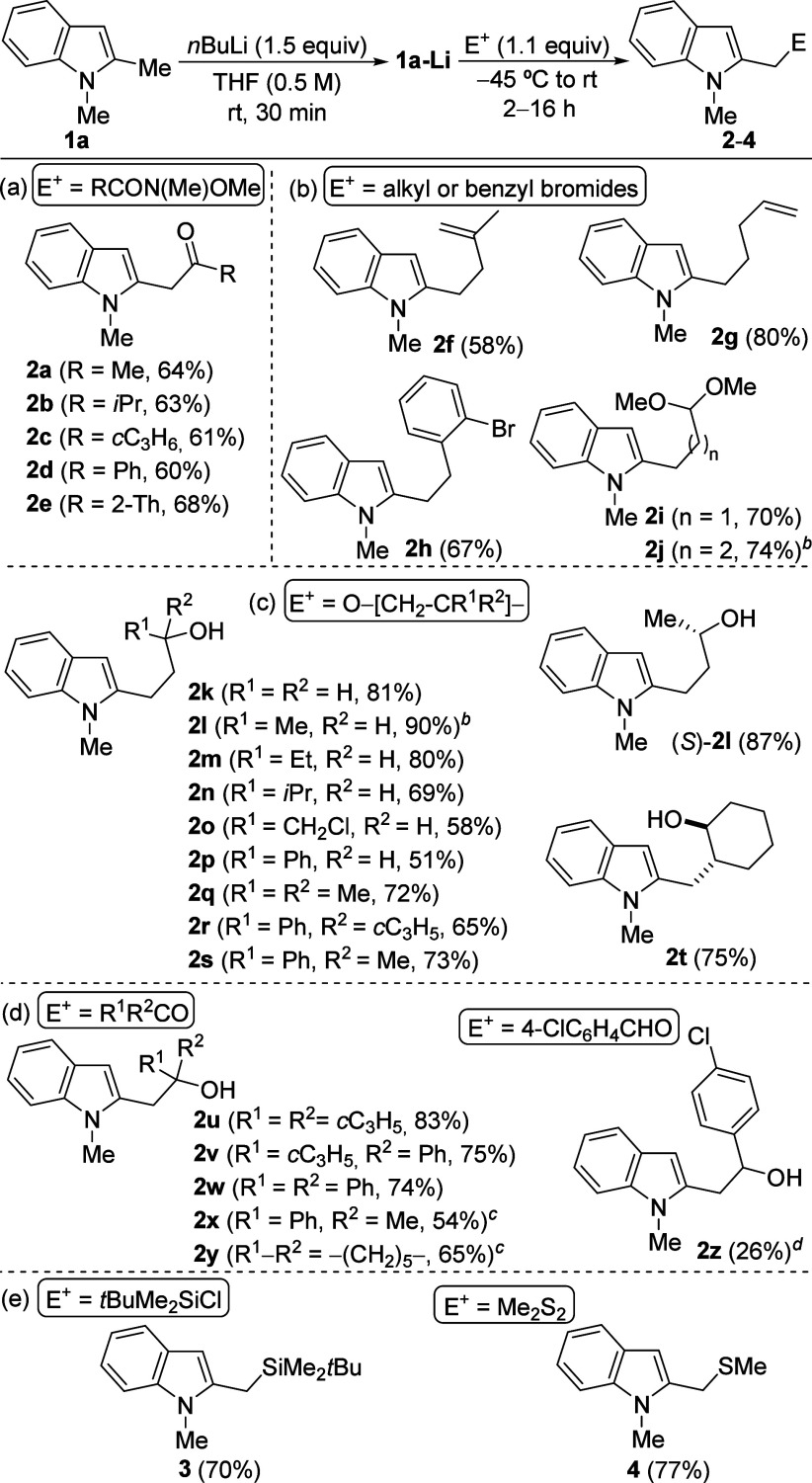
Synthesis of C2-Functionalized Indoles 2-4 from 1,2-Dimethylindole (**1a**)­^a^

a Reaction conditions: **1a** (1 mmol), *n*BuLi (0.6 mL of a 2.5 M sol in hexane, 1.5 mmol), THF (2 mL), E^+^ (1.1 mmol).
b Carried out at 10 mmol scale.
c Carried out by adding LaCl_3_·LiCl (1.5 mmol) prior to the electrophile.
d The aldehyde was added at −78 °C and reacted 1 h at low temperature.

On the other hand, the reaction of **1a-Li** with cyclopropyl and/or aromatic ketones also proceeded efficiently, leading to tertiary alcohols **2u**–**w**. In contrast, the reactions with alkyl ketones such as acetophenone or cyclohexanone provided lower yields, likely due to competing enolization. Gratifyingly, moderate yields of **2x**,**y** were obtained when adding LaCl_3_.2LiCl solution to **1a-Li** prior to the reaction with the ketone. The low electronegativity and high oxophilicity of La favor the addition pathway.^24^ Disappointingly, the use of aldehydes as electrophiles provided very low yields of the desired alcohols due to competitive decomposition. Nevertheless, when using 4-chlorobenzaldehyde, the hydroxy-functionalized indole **2z** could be isolated in low yield (Scheme 2d). Finally, trialkylsilyl and thiomethyl groups could be introduced at the benzylic position of **1a** by employing trialkylsilyl chlorides and dimethyl disulfide as electrophiles, respectively, affording 2-functionalized indoles **3** and **4** in good yields (Scheme 2e). Interestingly, only a few methods for the synthesis of 2-thiomethylindoles such as **4** are known.^25^

Considering the few methods available for the efficient preparation of 2-benzylindoles,^26^ we envisioned that the Pd-catalyzed coupling of 2-lithiomethylindole **1a-Li**, or related organometallic reagents, with aryl halides could provide an efficient route to 2-benzylindole derivatives. In this way, initial attempts employing Feringa conditions^27^ for the direct coupling of **1a-Li** with 3-bromo or 3-chloroanisole proved unsuccessful. We then turned our attention to the well-established Negishi reaction, which employs organozinc reagents for the C–C coupling. In this way, treatment of **1a-Li** with a ZnCl_2_ solution in THF, followed by Pd-catalyzed cross-coupling with a selection of aryl bromides, led to functionalized 2-benzylindoles **5** in moderate to good yields (Scheme 3). The reaction conditions employed for the Negishi coupling were previously described by Knochel and co-workers for the benzylic arylation of 2-methylbenzo­[*b*]­thiophene and 2-methylbenzo­[*b*]­furan, and were not reoptimized.^20^

**Scheme 3:**
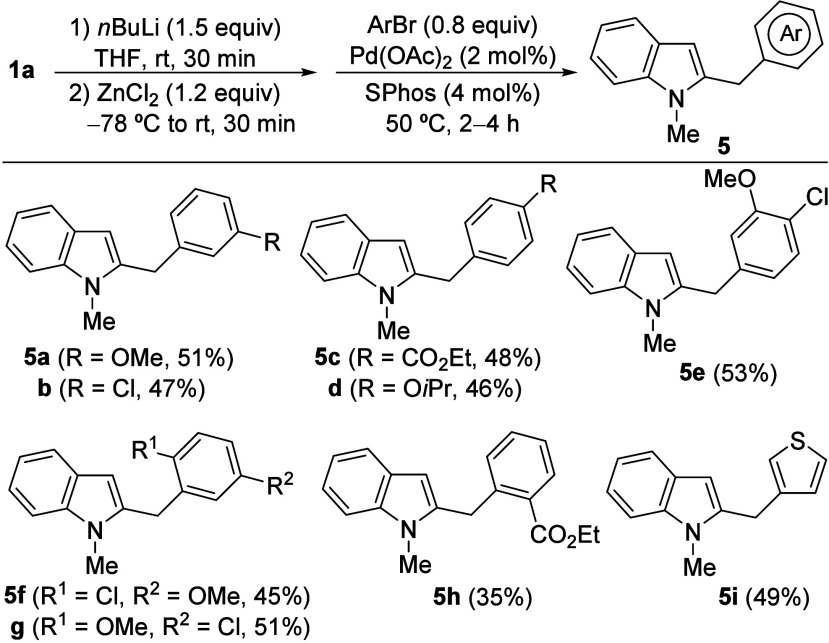
Synthesis of 2-Benzylindoles **5** via Negishi Reaction

We next examined the α-lithiation of various 2-methylindoles **1b**–**n** bearing additional functional groups, such as halogens or methoxy substituents on the benzenoid ring, as well as different substituents at the *N*-atom and C3 positions (Table 2). First, we checked that the above optimized conditions were also effective for the metalation of 5-chloro-1,2-dimethylindole (**1b**), as evidenced by the isolation of the corresponding deuterated derivative **1b-D** with nearly complete deuterium incorporation (entry 1). In addition, selected electrophilic reagents such as an alkyl bromide or a ketone, led to the functionalized indole derivatives **6** (entries 2 and 3). Similarly, 4-chloro-2-methylindole **1c** was efficiently lithiated at the benzylic position, as confirmed by the formation of **1c-D** (entry 4). In contrast, the 6-chloro derivative **1d** underwent decomposition, likely due to competitive lithiation at C7 followed by benzyne generation (entry 5). With respect to methoxy-functionalized indoles, 5-methoxy-1,2-dimethylindole (**1e**) required *t*-BuLi as base to achieve efficient lithiation, affording **1e-D** and 2-functionalized indoles **7** after deuteration or electrophilic trapping with an alkyl bromide or an epoxide (entries 6–8). The corresponding 4-methoxy-2-methylindole **1f** was also lithiated at the 2-methyl group, although to a lesser extent (entry 9), whereas the 6-methoxy derivative **1g** was selectively metalated at C7, yielding the 7-deuterioindole **1g-D** (entry 10). As expected, bromine-functionalized indoles **1h,i** mainly led to Br–Li exchange rather than α-lithiation, thereby preventing the formation of the corresponding 2-lithiomethylindoles (entries 11–13). The 5-fluoro-2-methylindole **1j** did not prove to be a useful substrate, likely owing to competitive aryne formation and subsequent decomposition (entry 14). When *o*-lithiation adjacent to fluorine atom was avoided, as in 4-fluoro-5-methoxyindole **1k**, successful lithiation at the C2-methyl group was observed, allowing the isolation of **1k-D** (entry 15). The presence of a nitro group was not tolerated, as demonstrated with 2-methyl-5-nitroindole **1l** (entry 16). Finally, 1,2,3-trimethylindole (**1m**) and 1-phenyl-2-methylindole (**1n**) were also efficiently lithiated at the methyl group, after brief reoptimization (entries 17 and 19),^28^ and the corresponding organolithium intermediates **1-Li** furnished the functionalized indole derivatives **8a** and **9a** in synthetically useful yields (entries 18 and 20).

**Table 2:**
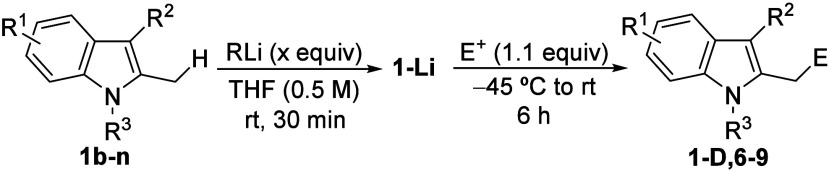
Lithiation of Functionalized 2-Methylindoles 1b–e and Further Reaction with Electrophiles: Synthesis of Functionalized Indole Derivatives **6**–**9** ^a^

entry	**1**	R^1^	R^2^	R^3^	RLi (x equiv)	E^+^	product	E	yield (%)^b^
1	**1b**	5-Cl	H	Me	*n*BuLi (1.5)	MeOD	1b-D	D	89^c^
2	**1b**	5-Cl	H	Me	*n*BuLi (1.5)	Br(CH_2_)_2_CH(OMe)_2_	**6a**	(CH_2_)_2_CH(OMe)_2_	62
3	**1b**	5-Cl	H	Me	*n*BuLi (1.5)	(*c*C_3_H_5_)_2_CO	**6b**	C(OH)(*c*C_3_H_5_)_2_	76
4	**1c**	4-Cl	H	Me	*n*BuLi (1.5)	MeOD	1c-D	D	85^c^
5	**1d**	6-Cl	H	Me	*n*BuLi (1.5)	MeOD	–	–	–^d^
6	**1e**	5-MeO	H	Me	*t*BuLi (1.5)	MeOD	1e-D	D	83^c^
7	**1e**	5-MeO	H	Me	*t*BuLi (1.5)	Br(CH_2_)_2_CH(OMe)_2_	**7a**	(CH_2_)_2_CH(OMe)_2_	61
8	**1e**	5-MeO	H	Me	*t*BuLi (1.5)	O–[(CH_2_)_2_]–	**7b**	(CH_2_)_2_OH	79
9	**1f**	4-MeO	H	Me	*n*BuLi (1.5)	MeOD	**1f-D**	D	80^e^
10	**1g**	6-MeO	H	Me	*n*BuLi (1.5)	MeOD	**1g-D** ^f^	D	82^c^
11	**1h**	5-Br	H	Me	*t*BuLi (3.5)	MeOD	**1h-D** ^g^	D	69^c^
12	**1i**	6-Br	H	Me	*t*BuLi (3.5)	MeOD	**1i-D** ^h^	D	75^c^
13	**1i**	6-Br	H	Me	*n*BuLi (1.5)	MeOD	**1i-Bu** ^i^	*n*Bu	58
14	**1j**	5-F	H	Me	*n*BuLi (1.5)	MeOD	–	–	–^j^
15	**1k**	4-F-5-MeO	H	Me	*n*BuLi (1.5)	MeOD	1k-D	D	79^c^
16	**1l**	5-NO_2_	H	Me	*n*BuLi (1.5)	MeOD	–	–	–^j^
17	**1m**	H	Me	Me	*t*BuLi (1.5)	MeOD	1m-D	D	83^c^
18	**1m**	H	Me	Me	*t*BuLi (1.5)	O–[(CH_2_)_2_]–	**8a**	(CH_2_)_2_OH	60
19^k^	**1n**	H	H	Ph	*n*BuLi (2)	MeOD	1n-D	D	88^c^
20^k^	**1n**	H	H	Ph	*n*BuLi (2)	Br(CH_2_)_2_CH(OMe)_2_	**9a**	(CH_2_)_2_CH(OMe)_2_	62

a Reaction conditions: **1** (0.5–1 mmol) in anhydrous THF (1–2 mL), rt, 30 min, under N_2_ atmosphere.

b Isolated yield referred to the corresponding 2-methylindole derivative **1**.

c >90% deuteration as determined by ^1^H NMR.

d Lithiation likely occurs at C7 position leading to benzyne generation and subsequent decomposition.

e >65% deuteration as determined by ^1^H NMR.

f **1g-D** is deuterated at C7 position.

g **1h-D** is ca. 85% deuterated at C5 position.

h **1i-D** is ca. 85% deuterated at C6 position and ca. 10% deuteration is observed at the 2-methyl group.

i After Br–Li exchange, reaction with the generated *n*BuBr took place leading to **1i-Bu** along with **1a**.

j Only decomposition was observed in the crude reaction mixture.

k 60 min instead 30 min for the lithiation step.

We then turned our attention toward indoles bearing an anion-stabilizing group at C-2, such as phenyl, trialkylsilyl or alkylthio, aiming to extend the α-lithiation methodology to these substrates, which were expected to be easier deprotonated than parent 1,2-dimethylindole (**1a**). Notably, the α-lithiation of these substrates has not been previously reported in the literature.^29^ 2-Benzyl-1-methylindole (**1o**) was prepared by lithiation of *N*-methylindole and further reaction with benzyl bromide, whereas silyl- and thio-functionalized indoles **3** and **4** had been previously synthesized from **1a** (see Scheme 2). Treatment with *n*BuLi as base, from 0 °C to rt, resulted in efficient deprotonation of the methylene group as confirmed by deuteration experiments and isolation of the corresponding deuterated derivatives **1o-D**, **3-D** and **4-D** (Scheme 4). Subsequent trapping of the resulting organolithiums, **1o-Li**, **3-Li** or **4-Li**, with a selection of electrophilic reagents, including epoxides, disulfides and alkyl bromides, provided indole derivatives **10a**–**i**, bearing two functional groups at the benzylic C-2 position, in moderate to good yields. Interestingly, the reaction of **1o-Li** with an aldehyde afforded **10d** with a significantly higher yield compared with the same reaction for **1a-Li**. In addition, compounds **10** bearing two stereogenic centers were obtained with low or negligible diastereoselectivity.

**Scheme 4:**
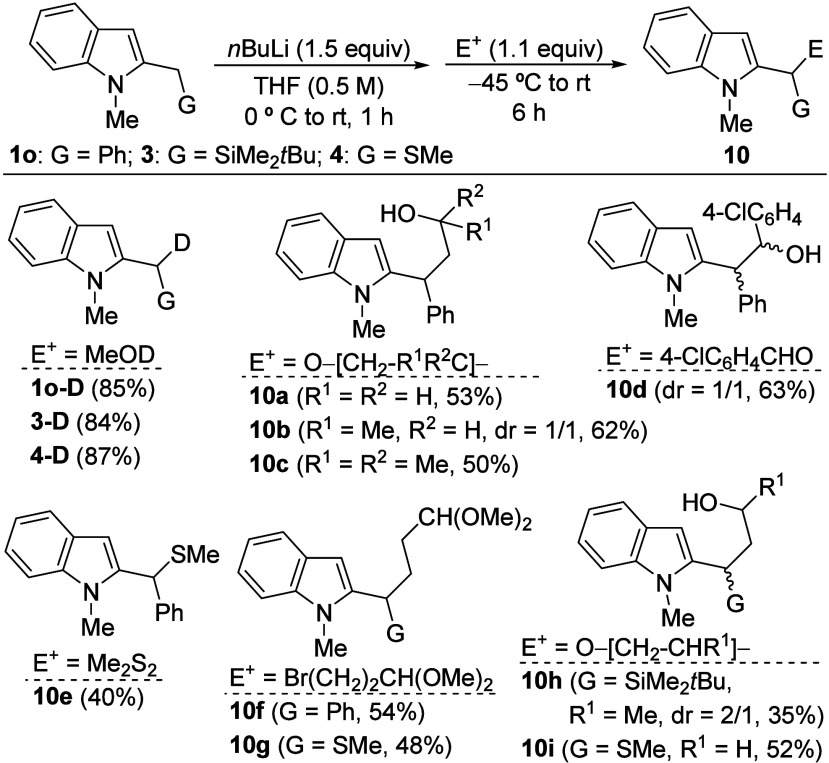
Synthesis of 2-Difunctionalized Indoles **10**

Over the last years, the use of more sustainable solvents in organic synthesis has emerged as an important research area. Biomass-derived solvents offer attractive alternatives to conventional organic solvents as they are biorenewable, safe and cheap. From this perspective, Deep Eutectic Solvents (DESs) represent a promising type of unconventional green solvents.^30^ A typical DES is formed by a Brønsted/Lewis base such as choline chloride or natural amino acids, and a hydrogen bond donor, typically polyalcohols, natural carboxylic acids, urea, or even water. Pioneering work by Hevia and García-Álvarez has demonstrated that classical transformations involving Grignard and organolithium reagents can be efficiently carried out at room temperature, under air, using DESs as solvents.^31^ For instance, they have reported the chemoselective alkylation of ketones,^31a^ the addition to imines and quinolines,^32^ the chemoselective addition of aryllithium reagents to nitriles,^33^ the regioselective addition to α,β-unsaturated carbonyls,^34^ or the synthesis of tertiary alcohols from esters.^35^ Similarly, Capriati and co-workers have developed interesting transformations of organolithiums and organomagnesium reagents using water as solvent.^36^ Considering the recent implementation of DESs in organolithium chemistry, we wondered if our functionalization of indoles via α-lithiation of 2-methylindoles could also work under the above-mentioned conditions. Initially, **1a-Li** was generated under conventional Schlenk techniques and we tested its addition to dicyclopropyl ketone dissolved in a DES. Three different DESs, those that led to better results in related transformations described in the literature,^37^ were essayed, and the mixture based on choline chloride (ChCl)/glycerol (Gly) (1/2) provided the best result with a useful 64% yield of isolated **2u**.

Building on this result, and inspired by the work Prandi and Capriati on the *o*-lithiation of amides and subsequent electrophilic trapping of the resulting organolithium, using 1 ChCl/2 Gly as DES, performing both the initial metalation and the reaction with the electrophile under air at room temperature,^38^ we explored the direct in situ generation of the organolithium **1a-Li** in the eutectic mixture, followed by trapping with dicyclopropyl ketone. A brief study of the reaction conditions was performed (Table 3).

**Table 3:**
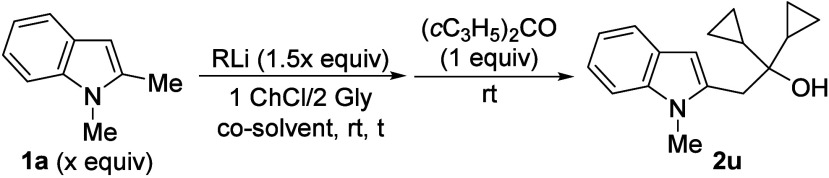
Optimization of the Reaction Conditions for the Synthesis of **2u** in ChCl/Gly^a^

entry	**1a** (x equiv)	RLi	cosolvent	t (s)	yield (%)^c^
1	1	*n*BuLi	–	4	0
2	1	*n*BuLi	THF	4	0
3	1	*s*BuLi	THF	4	35
4	1	*s*BuLi	CPME	4	0
5	1	*s*BuLi	2-MeTHF	4	0
6	1.5	*s*BuLi	THF	4	37
7	1.5	*s*BuLi	THF	6	45
8	1.5	*s*BuLi	THF	10	53
9	1.5	*s*BuLi	THF	15	62
10	1.5	*s*BuLi	THF	20	56

a Reaction conditions: **1a** (0.5 mmol) in DES (0.5 g), co-solvent (0.1 mL), rt, t, under air.

b Determined by ^1^H NMR using CH_2_Br_2_ as internal standard.

In the first attempt, indole **1a** was suspended in the eutectic mixture 1 ChCl/2 Gly and treated at rt in air with *n*BuLi under continuous stirring. Quenching the reaction mixture with dicyclopropylketone after 4 s resulted in recovery of **1a** after workup (entry 1). At this point, we decided to solubilize **1a** in a minimal amount of a cosolvent (0.1 mL, 5 M) prior to suspending in the DES with vigorous stirring, forming an emulsion. The addition of *n*BuLi and trapping with the ketone after 4 s did not provide a positive result (entry 2). Gratifyingly, changing the base from *n*BuLi to *s*BuLi led to the desired alcohol **2u**, although in moderate yield (entry 3). However, greener cyclopentyl methyl ether (CPME) or 2-methyltetrahydrofuran (2-MeTHF) failed to emulate THF as co-solvent (entries 4 and 5).^39^ Higher yields were achieved by adjusting the reaction time of the metalation step prior to the electrophilic quench (entries 6–10). Just an increase of a few seconds demonstrated to be crucial in favoring the formation of **1a-Li**, achieving optimal results after 15 s (entry 9). The higher basicity of *s*BuLi compared to *n*BuLi could be responsible for its success in the DES, a feature that has been crucial for other reported lithiation reactions in DESs,^38a,39c^ although the role and nature of organolithium aggregates in DES remains poorly understood.^31b^ So, conditions from entry 9 were selected to evaluate this environmentally friendly transformation with other electrophilic reagents (Scheme 5). In this way, a selection of 2-functionalized indoles **2**, previously synthesized under anhydrous and typical Schlenk conditions, were also prepared with this air- and moisture-tolerant approach.

**Scheme 5:**
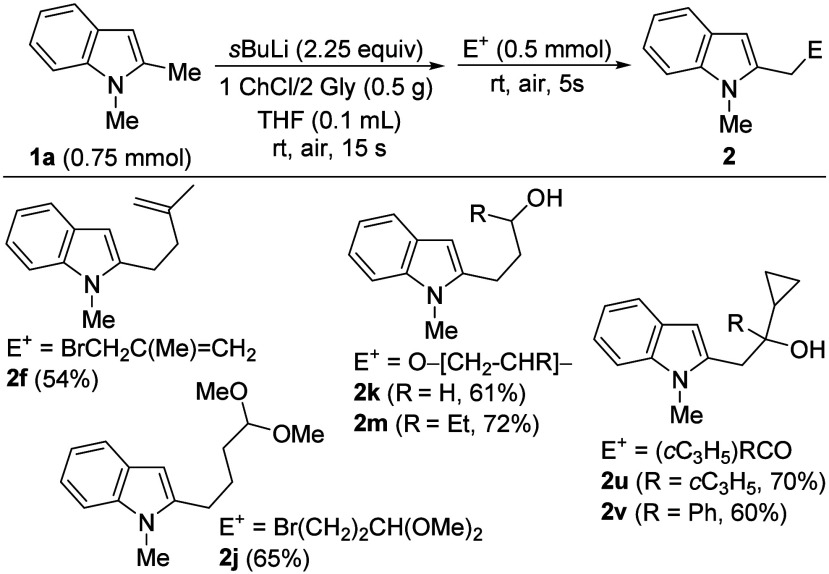
Synthesis of C2-Functionalized Indoles **2** under Air in DES

To further demonstrate the synthetic utility of the products prepared via the α-lithiation/trapping of 2-methylindoles, subsequent transformations of selected 2-functionalized indoles **2** were performed. For example, hydroboration and subsequent oxidation of **2f** gave rise to the expected alcohol **11** in moderate yield (Scheme 6). Interestingly, the cyclopenta­[*b*]­indole derivative **12** was synthesized in high yield by the Brønsted acid-catalyzed cyclization of tertiary alcohol **2r** (Scheme 6).^40^ In addition, an oxepino­[4,3-*b*]­indole derivative such as **13** could also be prepared, in this case through an oxa-Pictec-Spengler reaction of alcohol **2l** with benzaldehyde (Scheme 6).^41^ Finally, we envisaged a two-step protocol for accessing 2,4-functionalized carbazoles^42^ **15** via the gold-catalyzed cyclization of alkynols **14**, which could be prepared by the reaction of 2-lithiomethylindoles **1-Li** with selected alkynones (Scheme 6).

**Scheme 6:**
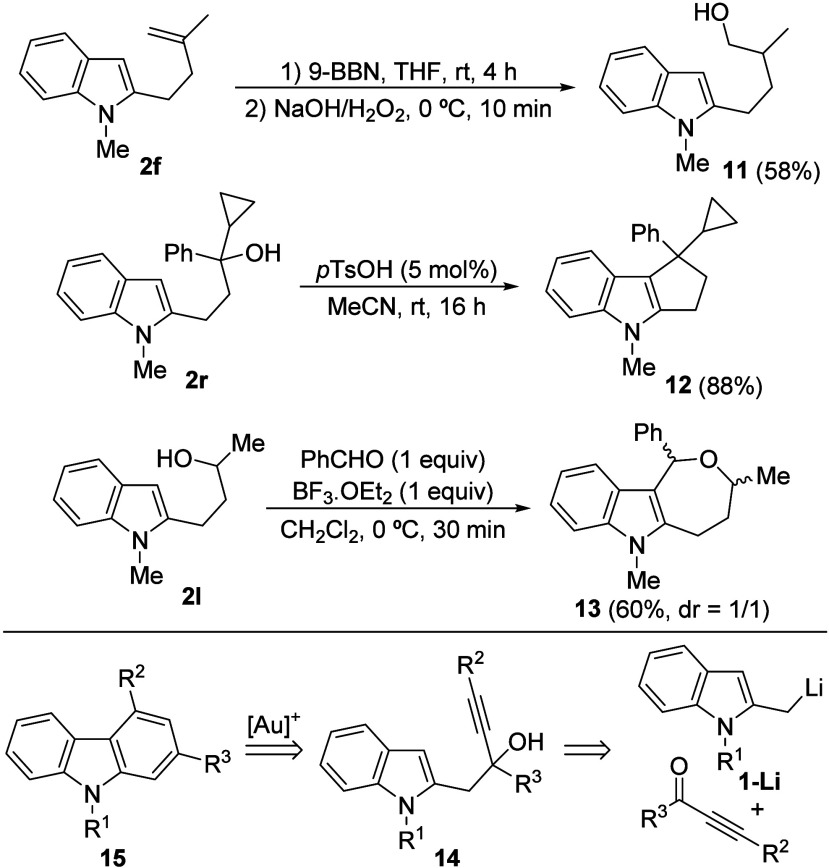
Derivatization of Selected 2-Functionalized Indoles 2 and Retrosynthetic Analysis for Carbazoles **15**

The reaction of *N*-methyl-2-lithiomethylindole (**1a-Li**) with a selection of aromatic alkynones afforded the corresponding alkynol derivatives **14** in good yields (Table 4). Next, their treatment with a catalytic amount of gold­(I) complex, IPrAuNTf_2_, led to the *N*-methyl-2,4-disubstituted carbazoles **15** that were isolated in high yields (entries 1–6). Alkynones bearing different aromatic (entries 1–3), cycloalkenyl (entry 4), and trimethylsilyl (entry 5) groups, as alkyne substituents could be employed. Interestingly, the gold-catalyzed cyclization also proceeded efficiently with terminal alkynone **14f**, prepared by desilylation of **14e**, providing monosubstituted carbazole **15f** also in high yield. In addition, a *NH*-carbazole **15g** was also prepared from alkynol **14g** that was obtained from 2-methyl *NH*-indole (**1p**), which was α-metalated upon treatment with *n*BuLi/*t*BuOK (entry 7).^16^ The reaction of alkynols **14** under gold­(I)-catalysis to afford carbazole derivatives **15** likely proceeds via C3-attack of the indole to the alkyne, which was activated by coordination to the gold complex, followed by protodemetalation and dehydration processes.

**Table 4:**
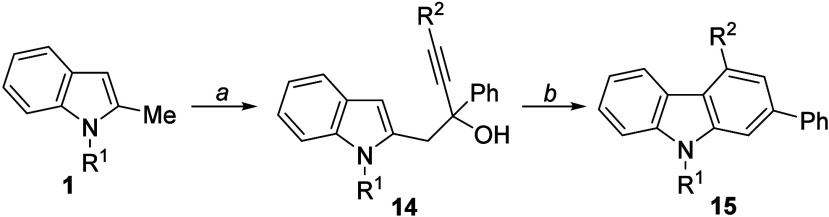
Synthesis of 2,4-Disubstituted Carbazoles **15** ^a^

entry	**1**	R^1^	R^2^	**14**	yield (%)^c^	**15**	yield (%)^d^
1	**1a**	Me	Ph	**14a**	78	**15a**	87
2	**1a**	Me	4-MeC_6_H_4_	**14b**	70	**15b**	84
3	**1a**	Me	4-MeOC_6_H_4_	**14c**	68	**15c**	89
4	**1a**	Me	*c*C_6_H_9_	**14d**	60	**15d**	74
5	**1a**	Me	SiMe_3_	**14e**	75	**15e**	90
6^e^	**1a**	Me	H	**14f**	89	**15f**	88
7^f^	**1p**	H	Ph	**14g**	55	**15g**	83

a Reaction conditions: **1** (1 mmol) in anhydrous THF (2 mL), rt, 30 min, under N_2_ atmosphere; then PhCOC≡CR^2^ (1.2 mmol), −78 °C to rt, 3 h.

b Reaction conditions: **14** (0.5 mmol), IPrAuNTf_2_ (5 mol %), CH_2_Cl_2_, rt, 1 h.

c Isolated yield referred to **1**.

d Isolated yield referred to **14**.

e **14f** was prepared from **14e** by base-mediated desilylation (TBAF, THF, rt).

f The metalation of 2-methylindole (**1p**) was performed with *n*BuLi (3 equiv)/*t*BuOK (2 equiv) in Et_2_O at 0 °C.

## Conclusions

In conclusion, the synthetic potential of underexplored 2-lithiomethylindoles has been highlighted by the preparation of a wide range of valuable indole derivatives decorated with a wide variety of functional groups at the C2-position. Remarkably, experimentally simple conditions for the effective α-lithiation of 2-methylindoles (at rt) and a diverse scope of electrophilic reagents have been established. Moreover, a greener, air- and moisture-tolerant alternative method for generating 1-methyl-2-lithiomethylindole has been developed using deep eutectic mixtures. Finally, the C2-functionalized indoles obtained are suitable for useful further derivatization reactions, as exemplified by the two-step process to synthesize 2,4-disubstituted carbazoles from 2-methyindoles.

### Safety Issues

Caution: Solutions of *n*-butyllithium (*n*-BuLi) react violently with water and may ignite upon exposure to moist air. Commercial solutions (15–20% in hexanes) are flammable. In contact with water releases butane which can ignite spontaneously. Contact with air or moisture must be strictly avoided. *n*-BuLi should be handled under an inert atmosphere by properly trained personnel wearing suitable personal protective equipment.

***Caution:*** Solutions of *sec*-butyllithium (*s*-BuLi) and *tert*-butyllithium (*t*-BuLi) react violently with water and may ignite in moist air. They are pyrophoric and must be handled under strictly inert conditions. Exposure to air or moisture must be avoided. These reagents should be handled only by individuals trained in its proper and safe use.

***Caution:*** Iodomethane is highly toxic. Inhalation, ingestion, or skin absorption may be fatal. It is a suspected carcinogen and alkylating agent. All manipulations should be performed in a well-ventilated fume hood while wearing appropriate protective equipment.

***Caution:*** Sodium hydride (NaH) is a flammable solid. In contact with water releases flammable gases which may ignite spontaneously. It should be handled and store under inert gas and protect from moisture.

## Supplementary Material



## Data Availability

The data underlying this study are available in the published article, in its Supporting Information, and openly available in zenodo at 10.5281/zenodo.17192034.

## Notes

Experimental procedures, compound characterization and NMR data, and copies of ^1^H and ^13^C­{^1^H} NMR spectra (PDF)
