# Supplementary material for: 2‑Lithiomethylindoles: Untapped Anions for the Synthesis of 2‑Functionalized Indoles
Source: J Org Chem. 2026 Feb 23;91(9):3466–74. doi: 10.1021/acs.joc.5c02679 (PMC12973304; doi:10.1021/acs.joc.5c02679)
Supplement: Supplementary file 1 [file jo5c02679_si_001.pdf]

2-Lithiomethylindoles: Untapped Anions for the Synthesis of 2-Functionalized Indoles

Sara Gómez-Gil, Marta Solas, Samuel Suárez-Pantiga, Roberto Sanz*

Área de Química Orgánica, Departamento de Química, Facultad de Ciencias, Universidad de Burgos, Pza. Misael Bañuelos s/n, 09001-Burgos, Spain

Supporting Information

Table of Contents

EXPERIMENTAL SECTION S2
- **General information and safety issues** S2
- **Preparation of starting substrates** S2
  - **Synthesis of 2-methylindoles 1b-o** S2
  - **Synthesis of epoxides S1,2** S6
- **Synthesis of 2-substituted 1-methyl-1*H*-indoles 2-4 from 1a** S6
- **Synthesis of 2-benzylindoles 5 from 1a *via* Negishi reaction** S13
- **Optimization of the lithiation conditions for 5-substituted-1,2-dimethylindoles 1b,e** S15
- **Synthesis of 2-substituted 1-methyl-1*H*-indoles 1-D,6, and 7 from 1b-k** S15
- **Optimization of the lithiation conditions for 1,2,3-trimethylindole (1m)** S18
- **Synthesis of 2-substituted 1,3-dimethyl-1*H*-indole 1m-D and 8a from 1m** S18
- **Optimization of the lithiation conditions for *N*-phenylindole (1n)** S19
- **Synthesis of 2-substituted 1-phenyl-1*H*-indole 1n-D and 9a from 1n** S20
- **Synthesis of 2-difunctionalized indoles 10** S20
- **Synthesis of alcohol 2u in Deep Eutectic Solvents** S23
  - **Preparation of Deep Eutectic Solvents (DESs)** S23
- **Synthesis of selected indole derivatives 2 in Deep Eutectic Solvents** S24
- **Synthesis of 2-methyl-4-(1-methyl-1*H*-indol-2-yl)butan-1-ol (11)** S25
- **Synthesis of tetrahydrocyclopenta[*b*]indole 12** S25
- **Synthesis of oxepino[4,3-*b*]indole 13** S26
- **Synthesis of 2-homopropargylindoles 14** S26
  - **Synthesis of 1-(1-methyl-1*H*-indol-2-yl)-2-phenylbut-3-yn-2-ol (14f)** S28
  - **Synthesis of 1-(1*H*-indol-2-yl)-2,4-diphenylbut-3-yn-2-ol (14g)** S28
- **Synthesis of carbazoles 15** S29
- **HPLC Traces** S31
- **References** S32

NMR SPECTRA S33

EXPERIMENTAL SECTION

General information and safety issues

All reactions involving air-sensitive compounds were carried out under an N_2 atmosphere in oven-dried glassware. All common reagents and solvents were purchased from commercial suppliers and used without any further purification.When heating was necessary an oil bath was used TLC was performed on alumina-backed plates coated with silica gel 60 with F_{254} indicator, using UV light or Ce/Mo solution and heat as a visualization agent. Flash silica gel chromatography was performed using Merk silica gel 60, 230–240 mesh. NMR spectra were recorded on a Varian Mercury Plus or Bruker Advanced III HD (300 MHz ^{1}H; 75.4 MHz ^{13}C, 282 MHz ^{19}F) or Bruker Advanced NEO 4500 (500 MHz ^{1}H, 126 MHz ^{13}C) instrument at room temperature. Chemical shifts (δ) are reported in ppm, using residual solvent peak as the internal reference ($CDCl_3$: δ_H = 7.26 and δ_C = 77.16). Coupling constants (*J*) are given in Hertz (Hz). Data are reported as follows: chemical shift, multiplicity (s: singlet, bs: broad single, bm: broad multiplet, d: doublet, dd: doublet of doublets, ddd: doublet of doublets of doublets, dddd: doublet of doublets of doublets of doublets, dq: doublet of quartets, dt: doublet of triplets, ddt: doublet of doublets of triplets, dtd: doublet of triplets of doublets, td: triplet of doublets, t: triplet, tt: triplet of triplets, q: quartet, p: quintuplet, h: hexuplet, m: multiplet), coupling constants and integration. Low-resolution electron impact mass spectra (EI-LRMS) were obtained at 70 eV and only the molecular ion and/or base peaks and significant MSpeaks are given. High resolution mass spectra (HRMS) were recorded on an instrument equipped with a QTOF analyser using ESI (+) or APCI (+). Melting points were measured on a Gallenkamp apparatus using open capillary tubes and were uncorrected.

Safety issues:

-Solutions of *n*-butyllithium (*n*-BuLi) react violently with water and may ignite upon exposure to moist air. Commercial solutions (15–20% in hexanes) are flammable. In contact with water releases butane which can ignite spontaneously. Contact with air or moisture must be strictly avoided. *n*-BuLi should be handled under an inert atmosphere by properly trained personnel wearing suitable personal protective equipment.

-Solutions of *sec*-butyllithium (*s*-BuLi) and *tert*-butyllithium (*t*-BuLi) react violently with water and may ignite in moist air. They are pyrophoric and must be handled under strictly inert conditions. Exposure to air or moisture must be avoided. These reagents should be handled only by individuals trained in its proper and safe use.

-Iodomethane is highly toxic. Inhalation, ingestion, or skin absorption may be fatal. It is a suspected carcinogen and alkylating agent. All manipulations should be performed in a well-ventilated fume hood while wearing appropriate protective equipment.

-Sodium hydride (NaH) is a flammable solid. In contact with water releases flammable gases which may ignite spontaneously. It should be handled and store under inert gas and protect from moisture.

Preparation of starting substrates

Synthesis of 2-methylindoles 1b-o

General procedure I-a

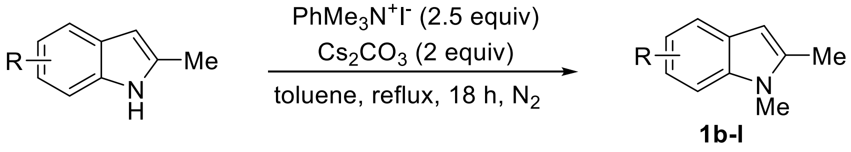

1b-l

To a stirred solution of the corresponding 5-susbtituted indole (3 mmol) in anhydrous toluene (20 mL, 0.15 M) phenyl trimethylammonium iodide (1.97 g, 7.5 mmol) and Cs_2CO_3 (1.95 g, 6 mmol) were added, and the resulting mixture was stirred for 18 h at reflux. Then, the mixture was cooled to 0 ºC and quenched with HCl (1 M) and extracted with EtOAc (3 × 20 mL). The combined organic layers were washed with HCl (1 M) (1 × 20 mL), brine (1 × 20 mL) and dried over anhydrous Na_2SO_4, filtered and concentrated under reduced pressure. The residue was filtered through a pad of silica using different mixtures of hexane/EtOAc to afford the indole derivatives.[S1a]

General procedure I-b

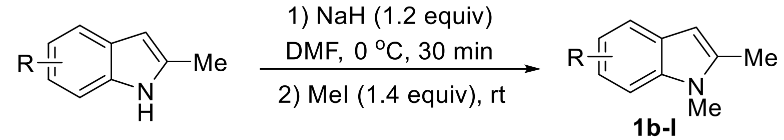

1b-l

To a stirred solution of the corresponding indole (3 mmol) in anhydrous DMF (10 mL, 0.3 M) was added NaH (144 mg, 3.6 mmol) at 0 ºC and stirred for 30 min at the same temperature. Then, MeI (0.26 mL, 4.2 mmol) was added at rt and stirred until completion (monitored by GC/MS). The reaction was quenched with H_2O (10 mL) and extracted with Et_2O (3 × 15 mL). The combined organic layers were washed with H_2O (2 × 15 mL), dried over anhydrous Na_2SO_4, filtered and concentrated under reduced pressure. The residue was filtered through a pad of silica using different mixtures of hexane/EtOAc to afford the indole derivatives.[S1b]

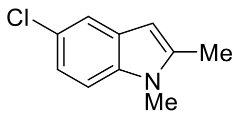

5-Chloro-1,2-dimethyl-1H-indole (1b):[S2a] General procedure I-a was followed using 5-chloro-2-methyl-1*H*-indole (496 mg, 3 mmol), which was isolated by flash column chromatography (hexane/EtOAc, 15/1) obtaining **1b** as a brownish solid (485 mg, 90%). M.p.: 60–62 ºC (lit. M.p.: 56–58 ºC).[S2a] R_f = 0.35 (hexane/EtOAc, 15/1). **^{1}H NMR** (300 MHz, $CDCl_3$): δ (ppm) = 7.69–7.45 (m, 1H), 7.45–6.99 (m, 2H), 6.24 (s, 1H), 3.64 (s, 3H), 2.45 (s, 3H). **^{13}C{^{1}H} NMR** (75.4 MHz, $CDCl_3$) δ (ppm) = 138.4, 135.8, 129.0, 124.9, 120.5, 119.0, 109.7, 99.3, 29.5, 12.8.

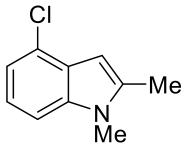

4-Chloro-1,2-dimethyl-1H-indole (1c): General procedure I-a was followed using 4-chloro-2-methyl-1*H*-indole (496 mg, 3 mmol), which was isolated by flash column chromatography (hexane/EtOAc, 15/1) obtaining **1c** as a colorless solid (415 mg, 75%). M.p.: 66–68 ºC. R_f = 0.34 (hexane/EtOAc, 15/1). **^{1}H NMR** (300 MHz, $CDCl_3$): δ (ppm) = 7.20–7.14 (m, 1H), 7.14–7.06 (m, 2H), 6.39 (s, 1H), 3.67 (s, 3H), 2.46 (s, 3H). **^{13}C{^{1}H} NMR** (75.4 MHz, $CDCl_3$) δ (ppm) = 138.1, 137.8, 126.7, 125.0, 121.1, 119.1, 107.5, 98.4, 29.8, 12.9. **HRMS** (ESI+) calcd for $C_{10}H_{11}ClN^+$ $[M+H]^+$ 180.0575, found 180.0574.

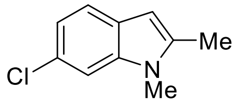

6-Chloro-1,2-dimethyl-1H-indole (1d): General procedure I-a was followed using 6-chloro-2-methyl-1*H*-indole (496 mg, 3 mmol), which was isolated by flash column chromatography (hexane/EtOAc, 15/1) obtaining **1d** as a colorless solid (377 mg, 70%). M.p.: 83–85 ºC. R_f = 0.35 (hexane/EtOAc, 15/1). **^{1}H NMR** (300 MHz, $CDCl_3$): δ (ppm) = 7.42 (d, J = 8.3 Hz, 1H), 7.24 (as, 1H), 7.05 (dd, J = 8.3, 1.8 Hz, 1H), 6.23 (s, 1H), 3.60 (s, 3H), 2.41 (s, 3H). **^{13}C{^{1}H} NMR** (75.4 MHz, $CDCl_3$) δ (ppm) = 137.84, 137.78, 126.6, 126.4, 120.4, 119.8, 108.9, 99.8, 29.6, 12.8. **HRMS** (ESI+) calcd for $C_{10}H_{11}ClN^+$ $[M+H]^+$ 180.0575, found 180.0574.

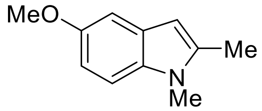

5-Methoxy-1,2-dimethyl-1H-indole (1e):[S2b] General procedure I-a was followed using 5-methoxy-2-methyl-1*H*-indole (483 mg, 3 mmol), which was isolated by flash column chromatography (hexane/EtOAc, 15/1) obtaining **1e** as a colourless solid (452 mg, 86%). M.p.: 65–67 ºC (lit. M.p.: 67–68 ºC).[S2b] R_f = 0.28 (hexane/EtOAc, 15/1). **^{1}H NMR** (300 MHz, $CDCl_3$): δ (ppm) = 7.18 (d, J = 8.8 Hz, 1H), 7.07 (d, J = 2.3 Hz, 1H), 6.95–6.78 (m, 1H), 6.23 (s, 1H), 3.90 (s, 3H), 3.64 (s, 3H), 2.44 (s, 3H). **^{13}C{^{1}H} NMR** (75.4 MHz, $CDCl_3$): δ (ppm) = 154.0, 137.5, 132.8, 128.3, 110.2, 109.4, 102.0, 99.3, 56.0, 29.5, 12.8.

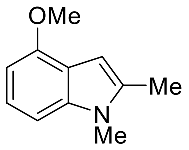

4-Methoxy-1,2-dimethyl-1H-indole (1f):[S1b] General procedure I-b was followed using 4-methoxy-2-methyl-1*H*-indole (483 mg, 3 mmol), which was isolated by flash column chromatography (hexane/EtOAc, 15/1) obtaining **1f** as a cololess solid (431 mg, 82%). M.p.: 120–122 ºC. R_f = 0.25 (hexane/EtOAc, 15/1). **^{1}H NMR** (300 MHz, $CDCl_3$): δ (ppm) = 7.14 (t, J = 8.0 Hz, 1H), 6.95 (d, J = 8.0 Hz, 1H), 6.57 (d, J = 8.0 Hz, 1H), 6.40 (s, 1H), 4.00 (s, 3H), 3.68 (s, 3H), 2.46 (s, 3H). **^{13}C{^{1}H} NMR** (75.4 MHz, $CDCl_3$): δ (ppm) = 152.7, 138.9, 135.3, 121.2, 118.3, 102.6, 99.6, 96.8, 55.4, 29.8, 12.8.

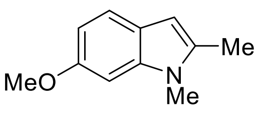

6-Methoxy-1,2-dimethyl-1H-indole (1g):[S1b] General procedure I-b was followed using 6-methoxy-2-methyl-1*H*-indole (483 mg, 3 mmol), which was isolated by flash column chromatography (hexane/EtOAc, 15/1) obtaining **1g** as a colorless solid (447 mg, 85%). M.p.: 69–71 ºC (lit. M.p.: 67–71 ºC).[S1b] R_f = 0.28 (hexane/EtOAc, 15/1). **^{1}H NMR** (300 MHz, $CDCl_3$): *δ* (ppm) = 7.45–7.35 (m, 1H), 6.84–6.72 (m, 2H), 6.19 (s, 1H), 3.90 (s, 3H), 3.62 (s, 4H), 2.41 (s, 4H). **^{13}C{^{1}H} NMR** (75.4 MHz, $CDCl_3$): *δ* (ppm) = 155.6, 138.1, 135.8, 122.3, 120.2, 108.7, 99.3, 93.2, 55.9, 29.5, 12.9.

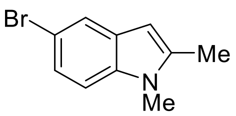

5-Bromo-1,2-dimethyl-1H-indole (1h):[S2c] General procedure I-b was followed using 5-bromo-2-methyl-1*H*-indole (630 mg, 3 mmol), which was isolated by flash column chromatography (hexane/EtOAc, 15/1) obtaining **1h** as a colorless solid (537 mg, 80%). M.p.: 70–72 ºC (lit. M.p.: 76–79 ºC).[S2c] R_f = 0.34 (hexane/EtOAc, 15/1). **^{1}H NMR** (300 MHz, $CDCl_3$): *δ* (ppm) = 7.41 (ad, *J* = 10.9 Hz, 2H), 7.20 (d, *J* = 8.3 Hz, 1H), 6.26 (s, 1H), 3.62 (s, 3H), 2.43 (s, 3H). **^{13}C{^{1}H} NMR** (75.4 MHz, $CDCl_3$): *δ* (ppm) = 138.3, 137.7, 126.9, 122.4, 120.9, 114.0, 111.9, 99.9, 29.5, 12.8.

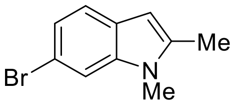

6-Bromo-1,2-dimethyl-1H-indole (1i):[S2d] General procedure I-b was followed using 6-bromo-2-methyl-1*H*-indole (630 mg, 3 mmol), which was pure without further purification. Colorless solid (585 mg, 87%). M.p.: 76–78 ºC. R_f = 0.28 (hexane/EtOAc, 15/1). **^{1}H NMR** (300 MHz, $CDCl_3$): *δ* (ppm) = 7.65 (d, *J* = 1.8 Hz, 1H), 7.24 (dd, *J* = 8.6, 1.8 Hz, 1H), 7.13 (d, *J* = 8.6 Hz, 1H), 6.21 (s, 1H), 3.65 (s, 3H), 2.44 (s, 3H). **^{13}C{^{1}H} NMR** (75.4 MHz, $CDCl_3$): *δ* (ppm) = 138.3, 136.1, 129.7, 123.2, 122.2, 112.6, 110.2, 99.4, 29.6, 12.9.

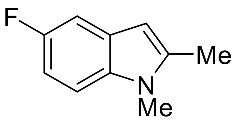

***5-Fluoro-1,2-dimethyl-1H-indole* (*1j*).**[S2e] General procedure I-b was followed using 5-fluoro-2-methyl-1*H*-indole (447 mg, 3 mmol), which was isolated by flash column chromatography (hexane/EtOAc, 15/1) obtaining **1j** as a colorless solid (406 mg, 83%). M.p = 58–60 ºC (lit. M.p.: 60–61 ºC).[S2e] R_f = 0.32 (hexane/EtOAc, 15/1). **^{1}H-NMR** (300 MHz, $CDCl_3$): *δ* (ppm) = 7.26–7.11 (m, 2H), 6.95 (dd, *J* = 9.4, 2.5 Hz, 1H), 6.25 (s, 1H), 3.64 (s, 3H), 2.44 (s, 3H). **^{13}C-NMR** (75.4 MHz, $CDCl_3$): *δ* (ppm) = 158.0 (d, J^1_{C-F} = 232.8 Hz, C), 138.7 (C), 134.1 (C), 128.2 (d, J^3_{C-F} = 10.2 Hz, C), 109.2 (d, J^3_{C-F} = 9.8 Hz, CH), 108.5 (d, J^2_{C-F} = 26.1 Hz, CH), 104.5 (d, J^2_{C-F} = 23.4 Hz, CH), 99.7 (d, J^4_{C-F} = 4.5 Hz, CH), 29.6 (CH_3), 12.9 (CH_3). **^{19}F-NMR** (282 MHz, $CDCl_3$): *δ* (ppm) = –125.86 (s, F).

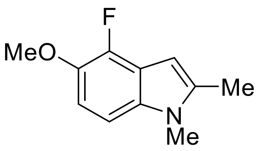

4-Fluoro-5-methoxy-1,2-dimethyl-1H-indole (1k): General procedure I-b was followed using 4-fluoro-2-methyl-1*H*-indol-5-ol (496 mg, 3 mmol), NaH (2.4 equiv) and MeI (2.8 equiv), which was isolated by flash column chromatography (hexane/EtOAc, 15/1) obtaining **1k** as a colorless solid (406 mg, 70%). M.p.: 71–73 ºC. R_f = 0.23 (hexane/EtOAc, 10/1). **^{1}H NMR** (300 MHz, $CDCl_3$): *δ* (ppm) = 7.07–6.84 (m, 2H), 6.31 (s, 1H), 3.95 (s, 3H), 3.63 (s, 3H), 2.42 (s, 3H). **^{13}C{^{1}H} NMR** (75.4 MHz, $CDCl_3$): *δ* (ppm) = 145.4 (d, J^1_{C-F} = 244.9 Hz), 140.0 (d, J^3_{C-F} = 9.6 Hz), 138.0, 135.3 (d, J^3_{C-F} = 10.9 Hz), 118.0 (d, J^2_{C-F} = 19.7 Hz), 110.5, 104.0 (d, J^4_{C-F} = 3.9 Hz), 95.3, 59.0, 29.8, 12.9. **HRMS** (ESI+) calcd for $C_{11}H_{13}FNO^+$ $[M+H]^+$ 194.0976, found 194.0976.

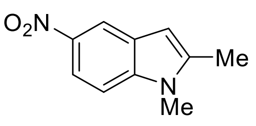

1,2-Dimethyl-5-nitro-1H-indole (1l):[S2c] General procedure I-b was followed using 2-methyl-5-nitro-1*H*-indole (528 mg, 3 mmol), which was isolated pure without further purification. Brown solid (462 mg, 81%). M.p.: 118–120 ºC (lit. M.p.: 122–126 ºC).[S2c] R_f = 0.22 (hexane/EtOAc, 10/1). **^{1}H NMR** (300 MHz, DMSO): *δ* (ppm) = 8.41 (d, *J* = 2.1 Hz, 1H), 7.95 (dd, *J* = 9.0, 2.1 Hz, 1H), 7.56 (d, *J* = 9.0 Hz, 1H), 6.50 (s, 1H), 3.74 (s, 3H), 2.44 (s, 3H). **^{13}C{^{1}H} NMR** (75.4 MHz, DMSO): *δ* (ppm) = 141.5, 140.5, 140.0, 126.7, 116.0, 115.5, 109.6, 101.7, 29.9, 12.5.

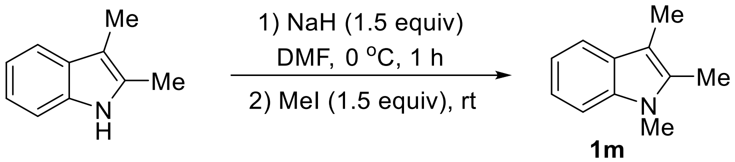

1m

To a stirred solution of 2,3-dimethyl-1*H*-indole (1.45 g, 10 mmol) in anhydrous DMF (10 mL, 1 M) was added NaH (600 mg, 15 mmol) at 0 ºC and stirred for 1 h at the same temperature. Then, MeI (2.2 g, 15 mmol) was added at rt and stirred until completion (monitored by GC/MS). The reaction was quenched with H_2O (10 mL) and extracted with Et_2O (3 × 15 mL). The combined organic layers were washed with H_2O (2 × 15 mL), dried over anhydrous Na_2SO_4, filtered and concentrated under reduced pressure. The residue was purified by flash column chromatography on silica gel using a 20/1 mixture of hexane/EtOAc as the eluent affording **1m**.

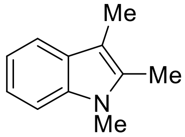

***1,2,3-Trimethyl-1H-indole (1m)**:*[S3] Pink oil (1.51 g, 95%): R_f = 0.43 (hexane/EtOAc, 20/1). **^{1}H NMR** (300 MHz, $CDCl_3$): *δ* (ppm) = 7.72–7.56 (m, 1H), 7.36-7.33 (m, 1H), 7.32–7.11 (m, 2H), 3.72 (s, 3H), 2.45 (s, 3H), 2.39 (s, 3H). **^{13}C{^{1}H} NMR** (75.4 MHz, $CDCl_3$): *δ* (ppm) = 136.6, 132.7, 128.5, 120.6, 118.6, 118.0, 108.5, 106.3, 29.5, 10.2, 8.9.

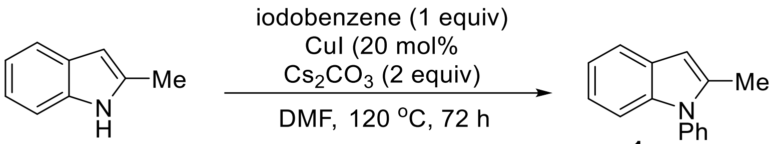

(1.4 equiv) **1n**

To a solution of 2-methyl-1*H*-indole (1.84 g, 14 mmol) in DMF (20 mL) were added PhI (2.04 g, 10 mmol), CuI (381 mg, 2 mmol) and $CsCO_3$ (6.5 g, 20 mmol). The resulting mixture was stirred at 120 ºC for 72 h. After completion, the reaction was filtered through a pad of silica using 100 mL of EtOAc as eluent and concentrated under reduced pressure. The residue was purified by flash column chromatography on silica gel using a 50/1 mixture of hexane/EtOAc as the eluent affording **1n**.[S4]

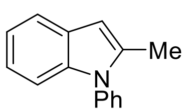

***2-Methyl-1-phenyl-1H-indole (1n)**:* Yellow oil (1.03 g, 50%): R_f = 0.31 (hexane/EtOAc, 50/1). **^{1}H NMR** (300 MHz, $CDCl_3$): *δ* (ppm) = 7.70–7.55 (m, 3H), 7.54–7.45 (m, 1H), 7.47–7.33 (m, 2H), 7.20–7.11 (m, 3H), 6.48 (s, 1H), 2.38 (s, 3H). **^{13}C{^{1}H} NMR** (75.4 MHz, $CDCl_3$): *δ* (ppm) = 138.3, 138.1, 137.1, 129.5, 128.3, 128.1, 127.8, 121.2, 120.1, 119.7, 110.1, 101.4, 13.5.

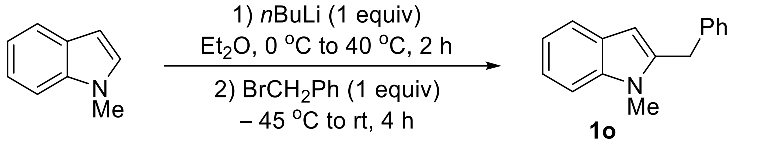

1o

To a stirred solution of *N*-methylindole (5.24 g, 40 mmol) in anhydrous Et_2O (40 mL, 1 M) was added *n*-BuLi (16 mL, 40 mmol, 2.5 M solution in hexane) at 0 ºC, and the resulting mixture was heated at 40 ºC for 2 h. Next, benzylbromide (7.53 g, 44 mmol) was added at –45 ºC and the resulting mixture was stirred for 4 h at rt. Then, the mixture was quenched with aq NH_4Cl (25 mL) and extracted with Et_2O (3 × 15 mL). The combined organic layers were dried over anhydrous Na_2SO_4, filtered and concentrated under reduced pressure. The residue was filtered through a pad of silica using a mixture of hexane/EtOAc (2/1) to afford the indole derivative **1o** (4.4 g, 50%), which could not be isolated in pure form.

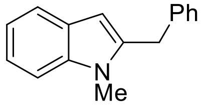

***2-Benzyl-1-methyl-1H-lindole (1o)**:*[S5] Brown oil. R_f = 0.41 (hexane/EtOAc, 20/1). (lit. M.p.: 60.6–61.3 ºC).[S5] **^{1}H NMR** (300 MHz, $CDCl_3$): *δ* (ppm) = 7.78–7.57 (m, 1H), 7.39–7.24 (m, 7H), 7.20–7.17 (m, 1H), 6.36 (s, 1H), 4.21 (s, 2H), 3.61 (s, 3H). **^{13}C{^{1}H} NMR** (75.4 MHz, $CDCl_3$): *δ* (ppm) = 139.2, 138.5, 137.8, 129.3, 128.7, 127.9, 126.6, 121.0, 120.1, 119.5, 109.0, 101.3, 33.5, 29.8.

Synthesis of epoxides S1,2

General procedure I-c

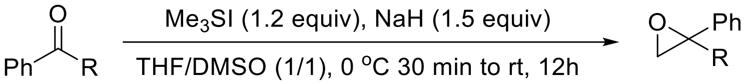

To a stirred solution of NaH (300 mg, 7.5 mmol) in anhydrous THF (10 mL) and DMSO (10 mL) was added a solution of trimethylsulfonium iodide (1.22 g, 6 mmol) in DMSO (4 mL) in an ice bath. Next, the corresponding ketone (5 mmol) was added and the resulting mixture was stirred for 30 min at 0 ºC and 16 h at rt. Then, the mixture was quenched with H_2O (5 mL) and extracted with DCM (3 × 15 mL). The combined organic layers were washed with H_2O (2 × 15 mL), dried over anhydrous Na_2SO_4, filtered and concentrated under redued pressure obtaining the corresponding epoxides in pure form.

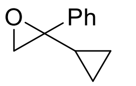

2-Cyclopropyl-2-phenyloxirane (S1): General procedure I-c was followed using cyclopropyl phenyl ketone (731 mg, 5 mmol) obtaining **S1** as a colourless oil (734 mg, 92%). ^{1}H NMR (500 MHz, $CDCl_3$): δ 7.71–7.47 (m, 2H), 7.47–7.36 (m, 2H), 7.37–7.28 (m, 1H), 2.93 (dd, J = 5.5, 1.2 Hz, 1H), 2.80 (dd, J = 5.5, 1.2 Hz, 1H), 1.76–1.51 (m, 1H), 0.67–0.56 (m, 2H), 0.55–0.37 (m, 2H). ^{13}C{^{1}H} NMR (126 MHz, $CDCl_3$): δ (ppm): 140.8, 128.2, 127.5, 126.0, 59.3, 54.5, 14.2, 2.3, 1.9. HRMS (ESI+) calcd for $C_{11}H_{13}O^+$ $[M+H]^+$ 161.0961, found 161.0957.

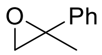

2-Methyl-2-phenyloxirane (S2): General procedure I-c was followed using methyl phenyl ketone (600 mg, 5 mmol) obtaining **S2** as a yellow oil (576 mg, 86%). ^{1}H NMR (300 MHz, $CDCl_3$): δ 7.80–7.03 (m, 5H), 2.98 (d, J = 5.4 Hz, 1H), 2.81 (dq, J = 5.4, 0.8 Hz, 1H), 1.73 (d, J = 0.8 Hz, 3H). ^{13}C{^{1}H} NMR (75.4 MHz, $CDCl_3$): δ (ppm): 141.3, 128.5, 127.6, 125.4, 57.2, 56.9, 21.9. HRMS (ESI+) calcd for $C_9H_{11}O^+$ $[M+H]^+$ 135.0804, found 135.0801.

Synthesis of 2-substituted 1-methyl-1*H*-indoles 2-4 from 1a

General procedure II

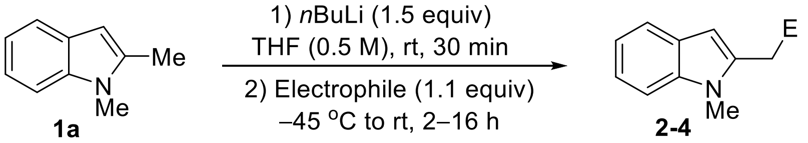

To a stirred solution of 1,2-dimethylindole (**1a**) (175 mg, 1 mmol) in anhydrous THF (2 mL, 0.5 M) was added *n*-BuLi (0.6 mL, 1.5 mmol, 2.5 M in hexane) at rt and stirred for 30 min. Then, the corresponding electrophile (1.1 mmol) was added at –45 ºC and the resulting solution was stirred at rt until completion (monitored by CG/MS). The resulting mixture was quenched with aq NH_4Cl (10 mL) and extracted with Et_2O (3 × 10 mL). The combined organic layers were dried over anhydrous Na_2SO_4, filtered and concentrated under reduced pressure. The residue was purified by flash column chromatography on silica gel using mixtures of hexane/EtOAc as the eluent to afford the corresponding 2-substituted indoles **2-4**.

General procedure III

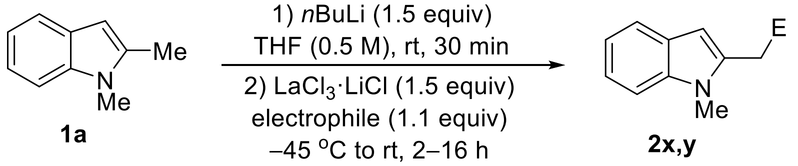

To a stirred solution of 1,2-dimethylindole (**1a**) (175 mg, 1 mmol) in anhydrous THF (2 mL, 0.5 M) was added *n*BuLi (0.6 mL, 1.5 mmol, 2.5 M in hexane) at rt and stirred for 30 min. In another Schlenck, the corresponding ketone (1 mmol) was added to $LaCl_3$·LiCl (2.5 mL, 0.6 M in THF, 1.5 mmol) and stirred at rt for 1 h. Then, the first solution was added to the second one at –45 ºC and the resulting mixture was stirred at rt until completion (monitored by CG/MS). The resulting mixture was quenched with aq. NH_4Cl (10 mL) and extracted with Et_2O (3 × 10 mL). The combined organic layers were dried over anhydrous Na_2SO_4, filtered and concentrated under reduced pressure. The residue was purified by flash column chromatography on silica gel using mixtures of hexane/EtOAc as the eluent to afford the 2-substituted indoles **2x,y**.

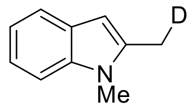

2-Deuteriomethyl-1-methyl-1H-indole (1a-D): General procedure II was followed using MeOD (excess) as electrophile obtaining **1a-D**, as a brown solid (131 mg, 90%). M.p.: 53–55 ºC. **^{1}H NMR** (300 MHz, $CDCl_3$): *δ* (ppm): 7.56 (d, *J* = 7.8 Hz, 1H), 7.28 (t, *J* = 6.5 Hz, 1H), 7.19 (t, *J* = 7.5 Hz, 1H), 7.16–7.05 (m, 1H), 6.29 (s, 1H), 2.45-2.43 (m, 2H). **^{13}C{^{1}H} NMR** (75.4 MHz, $CDCl_3$): *δ* (ppm): 137.4, 136.9, 128.1, 120.5, 119.7, 119.3, 108.8, 99.7, 29.4, 12.6 (t, $J_{C\text{-}D}$ = 19.5 Hz). **HRMS** (ESI+) calcd for $C_{10}H_{10}DN^+$ $[M+H]^+$ 147.1027, found 147.1026.

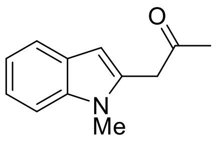

1-(1-Methyl-1H-indol-2-yl)propan-2-one (2a):[S6] General procedure II was followed using *N*-methoxy-*N*-methylacetamide (103 mg, 1.1 mmol) as electrophile obtaining **2a**, which was isolated by flash column chromatography (hexane/EtOAc, 5/1) as an orange oil (120 mg, 64%). R_f = 0.1 (hexane/EtOAc, 5/1). **^{1}H NMR** (300 MHz, $CDCl_3$): *δ* (ppm): 7.63 (d, *J* = 7.8 Hz, 1H), 7.34 (d, *J* = 8.2 Hz, 1H), 7.30–7.22 (m, 1H), 7.21–7.10 (m, 1H), 6.45 (s, 1H), 3.90 (s, 2H), 3.67 (s, 3H), 2.23 (s, 3H). **^{13}C{^{1}H} NMR** (75.4 MHz, $CDCl_3$): *δ* (ppm): 205.1, 137.8, 133.1, 127.8, 121.5, 120.3, 119.7, 109.3, 102.1, 43.0, 29.9, 29.0.

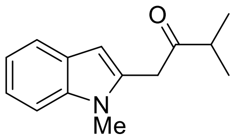

3-Methyl-1-(1-methyl-1H-indol-2-yl)butan-2-one (2b): General procedure II was followed using *N*-methoxy-*N*-methylisobutyramide (144 mg, 1.1 mmol) as electrophile obtaining **2b**, which was isolated by flash column chromatography (hexane/EtOAc, 5/1) as an orange solid (135 mg, 63%). M.p.: 81–83 ºC. R_f = 0.32 (hexane/EtOAc, 5/1). **^{1}H NMR** (300 MHz, $CDCl_3$): *δ* (ppm): 7.60 (d, *J* = 7.8 Hz, 1H), 7.33 (d, *J* = 8.2 Hz, 1H), 7.24 (t, *J* = 7.5 Hz, 1H), 7.14 (t, *J* = 7.5 Hz, 1H), 6.42 (s, 1H), 3.96 (s, 2H), 3.67 (s, 3H), 2.84 (p, *J* = 6.9 Hz, 1H), 1.16 (d, *J* = 6.9 Hz, 6H). **^{13}C{^{1}H} NMR** (75.4 MHz, $CDCl_3$): *δ* (ppm): 210.8, 137.8, 133.4, 127.8, 121.4, 120.3, 119.6, 109.3, 102.0, 40.0, 39.8, 30.0, 18.5. **HRMS** (ESI+) calcd for $C_{14}H_{17}NO^+$ $[M+H]^+$ 216.1383, found 216.1389.

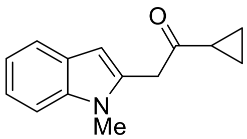

1-Cyclopropyl-2-(1-methyl-1H-indol-2-yl)ethan-1-one (2c): General procedure II was followed using *N*-methoxy-*N*-methylcyclopropanecarboxamide (142 mg, 1.1 mmol) as electrophile obtaining **2c**, which was isolated by flash column chromatography (hexane/EtOAc, 5/1) as an orange solid (130 mg, 61%). M.p.: 64–66 ºC. R_f = 0.27 (hexane/EtOAc, 5/1). **^{1}H NMR** (300 MHz, $CDCl_3$): *δ* (ppm): 7.64 (d, *J* = 7.8 Hz, 1H), 7.35 (d, *J* = 8.1 Hz, 1H), 7.31–7.23 (m, 1H), 7.16 (t, *J* = 7.1 Hz, 1H), 6.50 (s, 1H), 4.03 (s, 2H), 3.67 (s, 3H), 2.38–1.96 (m, 1H), 1.15-1.10 (m, 2H), 0.98–0.87 (m, 2H). **^{13}C{^{1}H} NMR** (126 MHz, $CDCl_3$): *δ* (ppm): 207.1, 137.9, 133.4, 127.9, 121.4, 120.3, 119.7, 109.2, 102.2, 43.0, 29.9, 19.6, 11.8. **HRMS** (ESI+) calcd for $C_{14}H_{15}NO^+$ $[M+H]^+$ 214.1226, found 214.1232.

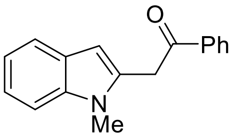

2-(1-Methyl-1H-indol-2-yl)1-phenylethan-1-one (2d):[S6] General procedure II was followed using *N*-methoxy-*N*-methylbenzamide (182 mg, 1.1 mmol) as electrophile obtaining **2d**, which was isolated by flash column chromatography (hexane/EtOAc, 6/1) as a yellow solid (182 mg, 60%). M.p.: 130–132 ºC (lit. M.p.: 121–123 ºC).[S6] R_f = 0.33 (hexane/EtOAc, 6/1). **^{1}H NMR** (300 MHz, $CDCl_3$): *δ* (ppm): 8.22–7.98 (m, 2H), 7.68–7.55 (m, 2H), 7.52 (t, *J* = 7.5 Hz, 2H), 7.34 (d, *J* = 8.2 Hz, 1H), 7.30–7.18 (m, 1H), 7.18–7.07 (m, 1H), 6.43 (s, 1H), 4.49 (s, 2H), 3.72 (s,

3H). **^{13}C{^{1}H} NMR** (75.4 MHz, $CDCl_3$): *δ* (ppm): 195.9, 137.8, 136.3, 133.6, 133.4, 128.9, 128.7, 127.8, 121.4, 120.3, 119.6, 109.2, 102.2, 37.8, 30.1.

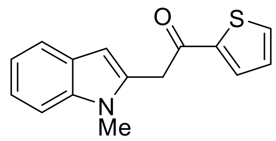

2-(1-Methyl-1H-indol-2-yl)-1-(thiophen2-yl)ethan-1-one (***2e***): General procedure II was followed using *N*-methoxy-*N*-methyl-2-thiophenecarboxamide (188 mg, 1.1 mmol) as electrophile obtaining **2e**, which was isolated by flash column chromatography (hexane/EtOAc, 4/1) as a yellow oil (173mg, 68%). R_f = 0.31 (hexane/EtOAc, 4/1). **^{1}H NMR** (300 MHz, $CDCl_3$): *δ* (ppm): 7.83 (dd, *J* = 3.8, 1.1 Hz, 1H), 7.66 (dd, *J* = 5.0, 1.1 Hz, 1H), 7.63–7.53 (m, 1H), 7.36–7.26 (m, 1H), 7.29–7.16 (m, 1H), 7.18–7.06 (m, 2H), 6.45 (s, 1H), 4.38 (s, 2H), 3.72 (s, 3H). **^{13}C{^{1}H} NMR** (75.4 MHz, $CDCl_3$): *δ* (ppm): 188.7, 143.3, 137.9, 134.5, 133.1, 133.0, 128.4, 127.7, 121.5, 120.3, 119.6, 109.3, 102.4, 38.8, 30.2. **HRMS** (ESI+) calcd for $C_{15}H_{14}NOS^+$ $[M+H]^+$ 256.0791, found 256.0794.

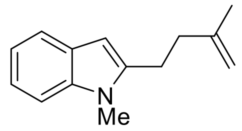

1-Methyl-2-(3-methylbut-3-en-1-yl)-1H-indole (2f):[S7] General procedure II was followed using 1,2-dimethylindole (**1a**) (580 mg, 4 mmol) and 3-bromo-2-methylprop-1-ene (592 mg, 4.4 mmol) as electrophile obtaining **2f**, which was isolated by flash column chromatography (hexane/EtOAc, 10/1) as a yellow oil (460 mg, 58%). R_f = 0.36 (hexane/EtOAc, 10/1). **^{1}H NMR** (300 MHz, $CDCl_3$): *δ* (ppm): 7.58 (d, *J* = 7.7 Hz, 1H), 7.31 (d, *J* = 8.1 Hz, 1H), 7.20 (td, *J* = 8.1, 1.3 Hz, 1H), 7.16–7.04 (m, 1H), 6.32 (s, 1H), 4.85 (s, 2H), 3.71 (s, 3H), 3.21–2.81 (m, 2H), 2.57–2.43 (m, 2H), 1.86 (s, 3H). **^{13}C{^{1}H} NMR** (75.4 MHz, $CDCl_3$): *δ* (ppm): 145.0, 140.9, 137.5, 128.0, 120.7, 119.9, 119.4, 110.6, 108.8, 98.8, 36.7, 29.5, 25.4, 22.8.

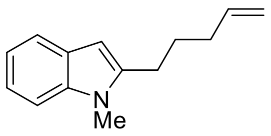

1-Methyl-2-(pent-4-en-1-yl)-1H-indole (2g): General procedure II was followed using 3-bromobuten-1-ene (148 mg, 1.1 mmol) as electrophile obtaining **2g** as an orange oil (159 mg, 80%).**^{1}H NMR** (300 MHz, $CDCl_3$): *δ* (ppm): 7.62–7.58 (m, 1H), 7.36–7.29 (m, 1H), 7.27–7.18 (m, 1H), 7.16–7.10 (m, 1H), 6.32 (s, 1H), 5.93 (ddt, *J* = 16.9, 10.1, 6.6 Hz, 1H), 5.23–4.97 (m, 2H), 3.70 (s, 3H), 2.80 (t, *J* = 7.7 Hz, 2H), 2.36–2.14 (m, 2H), 1.97–1.78 (m, 2H). **^{13}C{^{1}H} NMR** (75.4 MHz, $CDCl_3$): *δ* (ppm): 141.1, 138.3, 137.4, 128.0, 120.6, 119.8, 119.3, 115.3, 108.8, 98.9, 33.5, 29.5, 27.9, 26.2. **HRMS** (ESI+) calcd for $C_{14}H_{18}N^+$ $[M+H]^+$ 200.1434, found 200.1436.

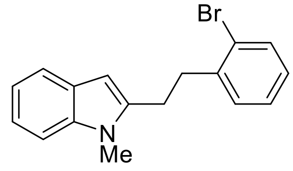

2-(2-Bromophenylethyl)-1-methyl-1H-indole (2h): General procedure II was followed using 1-bromo-2-(bromoethyl)benzene (274 mg, 1.1 mmol) as electrophile obtaining **2h**, which was isolated by flash column chromatography (hexane/EtOAc, 10/1) as a pink solid (210 mg, 67%) slightly contaminated with **1a**. M.p.: 57–59 ºC. R_f = 0.38 (hexane/EtOAc, 10/1). **^{1}H NMR** (300 MHz, $CDCl_3$): *δ* (ppm): 7.67–7.57 (m, 2H), 7.42–7.08 (m, 7H), 6.39 (s, 1H), 3.70 (s, 3H), 3.27–3.15 (m, 2H), 3.13-3.06 (m, 1H). **^{13}C{^{1}H} NMR** (75.4 MHz, $CDCl_3$): *δ* (ppm): 140.6, 140.2, 137.5, 133.0, 130.6, 128.2, 128.0, 127.8, 124.5, 120.9, 120.0, 119.5, 109.0, 99.2, 36.1, 29.6, 27.4. **HRMS** (ESI+) calcd for $C_{17}H_{16}BrN^+$ $[M+H]^+$ 314.0539, found 314.0545.

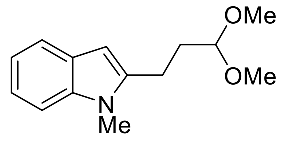

2-(3,3-Dimethoxypropyl)-1-methyl-1H-indole (2i): General procedure II was followed using 2-bromo-1,1-dimethoxyethane (186 mg, 1.1 mmol) as electrophile obtaining **2i**, which was isolated by flash column chromatography (hexane/EtOAc, 5/1) as a yellow oil (163 mg, 70%). R_f = 0.24 (hexane/EtOAc, 5/1). **^{1}H NMR** (300 MHz, $CDCl_3$): *δ* (ppm): 7.59 (dd, *J* = 7.7, 1.0 Hz, 1H), 7.31 (dd, *J* = 8.2, 7.1 Hz, 1H), 7.27–7.17 (m, 1H), 7.20–7.03 (m, 1H), 6.32 (s, 1H), 4.54 (t, *J* = 5.6 Hz, 1H), 3.72 (s, 3H), 3.43 (s, 6H), 2.98–2.77 (m, 2H), 2.24–2.02 (m, 2H). **^{13}C{^{1}H} NMR** (75.4 MHz, $CDCl_3$): *δ* (ppm): 140.4, 137.5, 127.9, 120.8, 119.9, 119.4, 108.9, 103.9, 98.8, 53.2, 31.5, 29.5, 21.9. **HRMS** (ESI+) calcd for $C_{14}H_{19}NO_2^+$ $[M+H]^+$ 234.1489, found 234.1496.

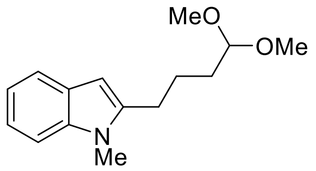

2-(4,4-Dimethoxybutyl)-1-methyl-1H-indole (2j): General procedure II was followed using 3-bromo-1,1-dimethoxypropane (201 mg, 1.1 mmol) as electrophile obtaining **2j**, which was isolated by flash column chromatography (hexane/EtOAc, 4/1) as an orange oil (173 mg, 70%). R_f = 0.32 (hexane/EtOAc, 4/1). **^{1}H NMR** (300 MHz, $CDCl_3$): δ (ppm): 7.61 (d, J = 7.4 Hz, 1H), 7.33 (d, J = 8.3 Hz, 1H), 7.23 (td, J = 8.2, 1.4 Hz, 1H), 7.20–7.09 (m, 1H), 6.34 (s, 1H), 4.50 (t, J = 4.6 Hz, 1H), 3.71 (s, 3H), 3.40 (s, 6H), 2.83 (t, J = 7.0 Hz, 2H), 2.29–1.62 (m, 4H). **^{13}C{^{1}H} NMR** (75.4 MHz, $CDCl_3$): δ (ppm): 140.8, 137.4, 128.0, 120.6, 119.8, 119.3, 108.8, 104.4, 99.0, 52.9, 32.2, 29.4, 26.6, 23.7. **HRMS** (ESI+) calcd for $C_{15}H_{22}NO_2^+$ $[M+H]^+$ 248.1645, found 248.1654. **2j** could also be obtained in a gram scale from **1a** (1.45 g, 10 mmol), *n*BuLi (6 mL, 15 mmol, 2.5 M in hexane) and 3-bromo-1,1-dimethoxypropane (2.01 g, 11 mmol) obtaining **2j**, which was isolated by flash column chromatography (hexane/EtOAc, 4/1) as an orange oil (1.83 g, 74%).

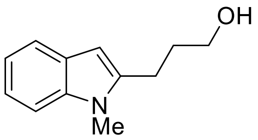

3-(1-Methyl-1H-indol-2-yl)propan-1-ol (2k): General procedure II was followed using oxirane (0.44 mL, 1.1 mmol, 2.5 M in ether) as electrophile obtaining **2k**, which was isolated by flash column chromatography (hexane/EtOAc, 2/1) as a yellow oil (153 mg, 81%). R_f = 0.20 (hexane/EtOAc, 2/1). **^{1}H NMR** (300 MHz, $CDCl_3$): δ (ppm): 7.61 (d, J = 7.7 Hz, 1H), 7.33 (d, J = 8.1 Hz, 1H), 7.27-7.20 (m, 1H), 7.18–7.10 (m, 1H), 6.33 (s, 1H), 3.79 (t, J = 6.2 Hz, 2H), 3.71 (s, 3H), 2.89 (t, J = 7.6 Hz, 2H), 2.03 (p, J = 7.8 Hz, 2H), 1.81 (bs, 1H). **^{13}C{^{1}H} NMR** (75.4 MHz, $CDCl_3$): δ (ppm): 140.9, 137.6, 128.1, 120.8, 120.0, 119.5, 109.1, 98.8, 62.1, 31.4, 29.4, 23.2. **HRMS** (ESI+) calcd for $C_{12}H_{16}NO^+$ $[M+H]^+$ 190.1226, found 190.1232.

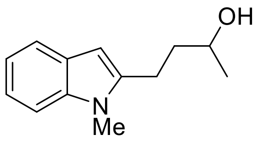

4-(1-Methyl-1H-indol-2-yl)butan-2-ol (2l):[S8] General procedure II was followed using 2-methyloxirane (64 mg, 1.1 mmol) obtaining **2l**, which was isolated by flash column chromatography (hexane/EtOAc, 1/1) as a yellow oil (173 mg, 85%). R_f = 0.32 (hexane/EtOAc, 1/1). **^{1}H NMR** (500 MHz, $CDCl_3$): δ (ppm): 7.57 (d, J = 7.3 Hz, 1H), 7.37–7.26 (m, 1H), 7.24–7.16 (m, 1H), 7.16–7.06 (m, 1H), 6.29 (s, 1H), 3.94 (h, J = 6.0 Hz, 1H), 3.69 (s, 3H), 2.92 (dt, J = 15.5, 7.2 Hz, 1H), 2.83 (dt, J = 15.5, 7.9 Hz, 1H), 1.96–1.83 (m, 2H), 1.70 (bs, 1H), 1.29 (d, J = 6.0 Hz, 3H). **^{13}C{^{1}H} NMR** (126 MHz, $CDCl_3$): δ (ppm): 140.9, 137.5, 127.9, 120.7, 119.8, 119.4, 108.9, 98.7, 67.6, 37.8, 29.5, 23.9, 23.2. **HRMS** (ESI+) calcd for $C_{13}H_{18}NO^+$ $[M+H]^+$ 204.1383, found 204.1385. **2l** could also be obtained in a gram scale from **1a** (1.45 g, 10 mmol), *n*BuLi (6 mL, 15 mmol, 2.5 M in hexane) and 2-methyloxirane (640 mg, 11 mmol) obtaining **2l**, which was isolated by flash column chromatography (hexane/EtOAc, 1/1) as a yellow oil (1.83 g, 90%).

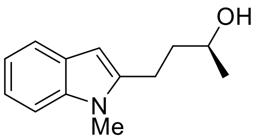

(S)-4-(1-Methyl-1H-indol-2-yl)butan-2-ol ((S)-2l): General procedure II was followed using (*S*)-2-methyloxirane (64 mg, 1.1 mmol) as electrophile obtaining ***(S)*-2l**, which was isolated by flash column chromatography (hexane/EtOAc, 1/1) as a yellow oil (177 mg, 87%). R_f = 0.32 (hexane/EtOAc, 1/1). **Column**: Chiralpak OD-H, **Flow**: 0.5 mL/min, **Eluent**: Hex/*i*PrOH 90/10, **Yield**: 177 mg, 87%, 99% *ee*. $[\alpha]_D^{25}$ = +7º (*c* 0.2, acetone).

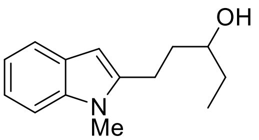

1-(1-Methyl-1H-indol-2-yl)pentan-3-ol (2m): General procedure II was followed using 2-ethyloxirane (79 mg, 1.1 mmol) as electrophile obtaining **2m**, which was isolated by flash column chromatography (hexane/EtOAc, 3/1) as a pink solid (174 mg, 80%). M.p.: 69–71 ºC. R_f = 0.18 (hexane/EtOAc, 3/1). **^{1}H NMR** (300 MHz, $CDCl_3$): δ (ppm): 7.62 (d, J = 7.8 Hz, 1H), 7.33 (d, J = 8.1 Hz, 1H), 7.29–7.18 (m, 1H), 7.21–7.10 (m, 1H), 6.34 (s, 1H), 3.72 (s, 3H), 3.72–3.63 (m, 1H), 3.08–2.74 (m, 2H), 2.06–1.80 (m, 2H), 1.72 (bs, 1H), 1.67–1.51 (m, 1H), 1.04 (t, J = 7.4 Hz, 3H). **^{13}C{^{1}H} NMR** (75.4 MHz, $CDCl_3$): δ (ppm): 141.0, 137.5, 127.9, 120.7, 119.8, 119.3, 108.8, 98.7, 72.7, 35.6, 30.5, 29.5, 23.1, 10.0. **HRMS** (ESI+) calcd for $C_{14}H_{20}NO^+$ $[M+H]^+$ 218.1539, found 218.1542.

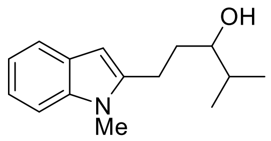

4-Methyl-1-(1-methyl-1H-indol-2-yl)pentan-3-ol (2n): General procedure II was followed using 2-isopropyloxirane (95 mg, 1.1 mmol) as electrophile obtaining **2n**, which was isolated by flash column chromatography (hexane/EtOAc, 3/1) as a yellowish solid (159 mg, 69%). M.p.: 85–87 ºC. R_f = 0.27 (hexane/EtOAc, 3/1). **^{1}H NMR** (300 MHz, $CDCl_3$): δ (ppm): 7.62 (d, J = 7.7 Hz, 1H), 7.34 (d, J = 8.1 Hz, 1H), 7.24 (t, J = 7.6 Hz, 1H), 7.22–7.12 (m, 1H), 6.35 (s, 1H), 3.73 (s, 3H), 3.53 (bs, 1H), 3.11–2.94 (m, 1H), 2.95–2.76 (m, 1H), 2.06–1.95 (m, 1H), 1.93–1.83 (m, 1H), 1.82–1.72 (m, 1H), 1.63 (bs, 1H), 1.03 (dd, J = 6.8, 1.1 Hz, 7H). **^{13}C{^{1}H} NMR** (75.4 MHz, $CDCl_3$): δ (ppm): 141.2, 137.5, 128.0, 120.7, 119.8, 119.3, 108.8, 98.7, 76.2, 33.9, 33.0, 29.5, 23.5, 18.9, 17.4. **HRMS** (ESI+) calcd for $C_{15}H_{22}NO^+$ $[M+H]^+$ 232.1696, found 232.1699.

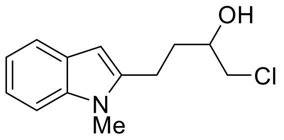

1-Chloro-4-(1-methyl-1H-2-yl)butan-2-ol (2o): General procedure II was followed using epichlorohydrin (101 mg, 1.1 mmol) as electrophile obtaining **2o**, which was isolated by flash column chromatography (hexane/EtOAc, 7/1) as a yellowish oil (138 mg, 58%): R_f = 0.12 (hexane/EtOAc, 7/1). **^{1}H NMR** (300 MHz, $CDCl_3$): δ (ppm): 7.61 (d, J = 7.6 Hz, 1H), 7.33 (d, J = 8.1 Hz, 1H), 7.29–7.15 (m, 1H), 7.21–7.08 (m, 1H), 6.34 (s, 1H), 3.72 (s, 3H), 3.15-3.09 (m, 1H), 3.02–2.92 (m, 2H), 2.90–2.82 (m, 1H), 2.66–2.56 (m, 1H), 2.19–2.06 (m, 1H), 2.02–1.87 (m, 1H). **^{13}C{^{1}H} NMR** (75.4 MHz, $CDCl_3$): δ (ppm): 139.9, 137.5, 127.9, 120.9, 119.9, 119.4, 108.9, 98.9, 51.7, 47.3, 31.6, 29.5, 23.3. **HRMS** (ESI+) could not be recorded.

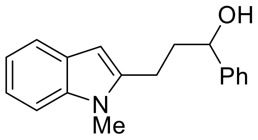

3-(1-Methyl-1H-indol-2-yl)-1-phenylpropan-1-ol (2p): General procedure II was followed using 2-phenyloxirane (132 mg, 1.1 mmol) as electrophile obtaining **2p**, which was isolated by flash column chromatography (hexane/EtOAc, 3.5/1) as a yellow oil (135 mg, 51%): R_f = 0.27 (hexane/EtOAc, 3.5/1). **^{1}H NMR** (500 MHz, $CDCl_3$): δ (ppm): 7.58 (dd, J = 7.7, 3.5 Hz, 1H), 7.47–7.31 (m, 4H), 7.33–7.26 (m, 2H), 7.23–7.15 (m, 1H), 7.17–7.06 (m, 1H), 6.32 (s, 1H), 4.91–4.76 (m, 1H), 3.67 (s, 3H), 2.92-2.86 (m, 2H), 2.32–2.16 (m, 2H), 1.99 (bs, 1H). **^{13}C{^{1}H} NMR** (126 MHz, $CDCl_3$): δ (ppm): 144.5, 140.6, 137.5, 128.8, 128.0, 127.9, 126.0, 120.8, 119.9, 119.4, 108.9, 98.9, 73.9, 37.7, 29.6, 23.2. **HRMS** (ESI+) calcd for $C_{18}H_{20}NO^+$ $[M+H]^+$ 266.1539, found 266.1541.

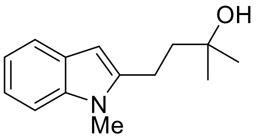

2-Methyl-4-(1-methyl-1H-indole-2-yl)butan-2-ol (2q):[S9] General procedure II was followed using 2,2-dimethyloxirane (79 mg, 1.1 mmol) as electrophile obtaining **2q**, which was isolated by flash column chromatography (hexane/EtOAc, 2.5/1) as a yellow solid (156 mg, 72%). M.p.: 79–81 ºC. R_f = 0.31 (hexane/EtOAc, 2.5/1). **^{1}H NMR** (500 MHz, $CDCl_3$): δ (ppm): 7.59-7.56 (m, 1H), 7.31-7.28 (m, 1H), 7.23–7.15 (m, 1H), 7.12-7.10 (m, 1H), 6.30 (s, 1H), 3.69 (s, 3H), 2.86 (t, J = 7.4 Hz, 2H), 1.93 (t, J = 7.4 Hz, 2H), 1.48 (bs, 1H), 1.36 (s, 6H). **^{13}C{^{1}H} NMR** (126 MHz, $CDCl_3$): δ (ppm): 141.3, 137.5, 128.0, 120.7, 119.9, 119.4, 108.8, 98.5, 70.8, 42.3, 29.5, 21.8. **HRMS** (ESI+) calcd for $C_{14}H_{20}NO^+$ $[M+H]^+$ 218.1539, found 218.1542.

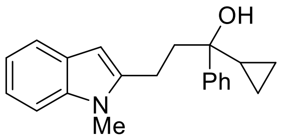

1-Cyclopropyl-3-(1-methyl-1H-2-yl)-1-phenylpropan-1-ol (2r): General procedure II was followed using 2-cyclopropyl-2-phenyloxirane (176 mg, 1.1 mmol) as electrophile obtaining **2r**, which was isolated by flash column chromatography (hexane/EtOAc, 3/1) as a yellow oil (198 mg, 65%). R_f = 0.37 (hexane/EtOAc, 3/1). **^{1}H NMR** (500 MHz, $CDCl_3$): δ (ppm): 7.58–7.44 (m, 3H), 7.45-7.39 (m, 2H), 7.36–7.24 (m, 2H), 7.18-7.10 (m, 2H), 6.26 (s, 1H), 3.57 (s, 3H), 3.05–2.70 (m, 1H), 2.59–2.47 (m, 1H), 2.39-2.25 (m, 1H), 1.67 (t, J = 4.2 Hz, 1H), 1.50–1.28 (m, 1H), 0.71–0.50 (m, 2H), 0.49–0.27 (m, 2H), the OH signal is missing. **^{13}C{^{1}H} NMR** (126 MHz, $CDCl_3$): δ (ppm): 145.8, 141.4, 137.5, 128.3, 127.9, 127.0, 125.7, 120.7, 119.9, 119.4, 108.8, 98.4, 75.2, 40.9, 29.5, 22.5, 21.4, 1.7, 1.0. **HRMS** (ESI+) calcd for $C_{21}H_{24}NO^+$ $[M+H]^+$ 306.1852, found 306.1854.

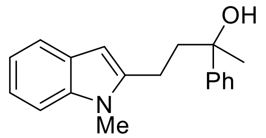

4-(1-Methyl-1H-indol-2-yl)-2-phenylbutan-2-ol (2s): General procedure II was followed using 2-methyl-2-phenyloxirane (147 mg, 1.1 mmol) as electrophile obtaining **2s**, which was isolated by flash column chromatography (hexane/EtOAc, 3/1) as an orange oil (204 mg, 73%). R_f = 0.23 (hexane/EtOAc, 3/1). **^{1}H NMR** (500 MHz, $CDCl_3$): δ (ppm): 7.57–7.51 (m, 3H), 7.44-7.41 (m, 2H), 7.36–7.28 (m, 1H), 7.29–7.23 (m, 1H), 7.17 (ddd, J = 8.2, 7.0, 1.3 Hz, 1H), 7.09 (ddd, J = 8.0, 7.0, 1.1 Hz, 1H), 6.25 (s, 1H), 3.59 (s, 3H), 2.82 (ddd, J = 15.4, 11.1, 5.8 Hz, 1H), 2.57 (ddd, J = 15.7, 11.1, 5.1 Hz, 1H), 2.35–2.20 (m, 2H), 1.86 (bs, 1H), 1.69 (s, 3H). **^{13}C{^{1}H} NMR** (126 MHz, $CDCl_3$): δ (ppm): 147.3, 141.1, 137.5, 128.5, 127.9, 127.0, 124.9, 120.7, 119.9, 119.4, 108.8, 98.5, 74.7, 42.8, 31.0, 29.5, 21.6. **HRMS** (ESI+) calcd for $C_{19}H_{22}NO^+$ $[M+H]^+$ 280.1696, found 280.1697.

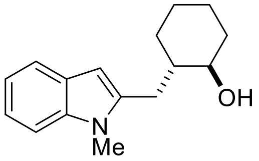

(1R*,2S*)-2-((1-methyl-1H-indol-2-yl)methyl)cyclohexan-1-ol (2t): General procedure II was followed using 1,2-dimethylindole (145 mg, 1 mmol) and cyclohexene oxide (108 mg, 1.1 mmol) as electrophile obtaining **2t**, which was isolated by flash column chromatography (hexane/EtOAc, 3/1) as an orange solid (182 mg, 75%). M.p.: 90–92 ºC. R_f = 0.27 (hexane/EtOAc, 3/1). **^{1}H NMR** (500 MHz, $CDCl_3$): δ (ppm): 7.58-7.56 (m, 1H), 7.31 (d, J = 8.1 Hz, 1H), 7.22-7.17 (m, 1H), 7.13-7.09 (m, 1H), 6.31 (s, 1H), 3.73 (s, 3H), 3.41–3.29 (m, 2H), 2.53 (dd, J = 14.9, 9.2 Hz, 1H), 2.05–1.96 (m, 1H), 1.92–1.83 (m, 1H), 1.81–1.70 (m, 2H), 1.69–1.50 (m, 2H), 1.38–1.24 (m, 2H), 1.15-1.13 (m, 1H), 1.07–0.95 (m, 1H). **^{13}C{^{1}H} NMR** (126 MHz, $CDCl_3$): δ (ppm): 139.8, 137.5, 127.9, 120.6, 119.7, 119.4, 109.0, 100.3, 74.9, 45.3, 36.0, 30.7, 30.2, 29.7, 25.5, 25.0. **HRMS** (ESI+) calcd for $C_{16}H_{22}NO^+$ $[M+H]^+$ 244.1696, found 244.1697.

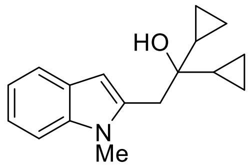

1,1-Dicyclopropyl-2-(1-methyl-1H-indol-2-yl)ethan-1-ol (2u): General procedure II was followed using dicyclopropyl ketone (121 mg, 1.1 mmol) as electrophile obtaining **2u**, which was isolated by flash column chromatography (hexane/EtOAc, 6/1) as an orange oil (212 mg, 83%). R_f = 0.29 (hexane/EtOAc, 6/1). **^{1}H NMR** (500 MHz, $CDCl_3$): δ (ppm): 7.68-7.64 (m, 1H), 7.40-7.37 (m, 1H), 7.31–7.26 (m, 1H), 7.22–7.19 (m, 1H), 6.49 (s, 1H), 3.83 (s, 3H), 3.17 (s, 2H), 1.38–1.37 (m, 1H), 1.00–0.94 (m, 2H), 0.57–0.45 (m, 6H), 0.40–0.35 (m, 2H). **^{13}C{^{1}H} NMR** (126 MHz, $CDCl_3$): δ (ppm): 137.6, 136.8, 127.8, 120.8, 119.9, 119.5, 109.3, 101.8, 70.7, 39.0, 30.2, 18.7, 1.3, –0.4. **HRMS** (ESI+) calcd for $C_{17}H_{22}NO^+$ $[M+H]^+$ 256.1696, found 256.1703.

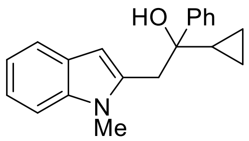

1-Cyclopropyl-2-(1-methyl-1H-indol-2-yl)-1-phenylethan-1-ol (2v): General procedure II was followed using cyclopropyl phenyl ketone (161 mg, 1.1 mmol) as electrophile obtaining **2v**, which was isolated by flash column chromatography (hexane/EtOAc, 5/1) as a yellow oil (218 mg, 75%). R_f = 0.30 (hexane/EtOAc, 5/1). **^{1}H NMR** (500 MHz, $CDCl_3$): δ 7.59–7.49 (m, 1H), 7.52–7.42 (m, 2H), 7.41–7.28 (m, 3H), 7.29–7.16 (m, 2H), 7.15–7.02 (m, 1H), 6.22 (s, 1H), 3.42 (s, 3H), 2.12 (bs, 1H), 1.58–1.36 (m, 1H), 0.53–0.31 (m, 4H). **^{13}C{^{1}H} NMR** (126 MHz, $CDCl_3$): δ (ppm): 146.4, 137.6, 135.7, 128.2, 127.7, 127.1, 125.7, 121.1, 120.1, 119.6, 109.3, 102.3, 73.8, 40.3, 29.7, 21.2, 1.6, 1.2. **HRMS** (ESI+) calcd for $C_{20}H_{22}NO^+$ $[M+H]^+$ 292.1696, found 292.1697.

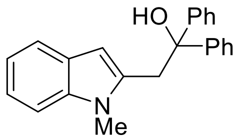

2-(1-Methyl-1H-indol-2-yl)-1,1-diphenylethan-1-ol (2w): General procedure II was followed using benzophenone (200 mg, 1.1 mmol) as electrophile obtaining **2w**, which was isolated by flash column chromatography (hexane/EtOAc, 4/1) as a yellow solid (242 mg, 74%). M.p.: 112–114 ºC. R_f = 0.32 (hexane/EtOAc, 4/1). **^{1}H NMR** (500 MHz, $CDCl_3$): δ (ppm): 7.65–7.52 (m, 1H), 7.53–7.43 (m, 4H), 7.42–7.28 (m, 6H), 7.28–7.19 (m, 1H), 7.14-7.09 (m, 1H), 6.18 (s, 1H), 3.83 (s, 2H), 3.28 (s, 3H), 3.18–2.90 (m, 1H). **^{13}C{^{1}H} NMR** (126 MHz, $CDCl_3$): δ (ppm): 146.6, 137.6, 128.4, 127.7, 127.3, 126.2, 121.3, 120.2, 120.0, 109.4, 102.6, 39.4, 29.5, one carbon signal is missing due to overlapping. **HRMS** (ESI+) calcd for $C_{23}H_{22}NO^+$ $[M+H]^+$ 328.1696, found 328.1697.

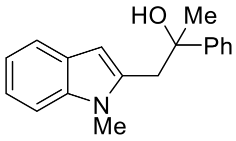

***1-(1-Methyl-1H-indol-2-yl)-2-phenylpropan-2-ol** (**2x**):* General procedure III was followed using acetophenone (120 mg, 1.1 mmol) as electrophile obtaining **2x**, which was isolated by flash column chromatography (hexane/EtOAc, 2/1) as a yellow oil (143 mg, 54%). R_f = 0.38 (hexane/EtOAc, 2/1). **^{1}H NMR** (300 MHz, $CDCl_3$): δ (ppm): 7.66–7.56 (m, 1H), 7.50–7.40 (m, 2H), 7.41–7.31 (m, 3H), 7.30–7.21 (m, 2H), 7.20–7.10 (m, 1H), 6.34 (s, 1H), 3.38 (s, 3H), 3.34–3.25 (m, 2H), 2.37 (bs, 1H), 1.71 (d, J = 1.3 Hz, 3H). **^{13}C{^{1}H} NMR** (75.4 MHz, $CDCl_3$): δ (ppm): 147.4, 137.6, 135.9, 128.4, 127.7, 127.0, 124.9, 121.2, 120.1, 119.6, 109.4, 102.3, 73.9, 41.5, 29.7, 29.6. **HRMS** (ESI+) calcd for $C_{18}H_{20}NO^+$ [M+H]$^+$ 266.1539, found 266.1541.

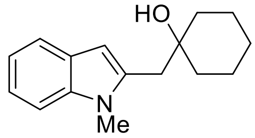

***1-((1-Methyl-1H-indol-2-yl)methyl)cyclohexan-1-ol** (**2y**).* General procedure III was followed using cyclohexanone (108 mg, 1.1 mmol) as electrophile obtaining **2y**, which was isolated by flash column chromatography (hexane/EtOAc, 3.5/1) as a yellow oil (158 mg, 65%). R_f = 0.38 (hexane/EtOAc, 3.5/1). **^{1}H NMR** (500 MHz, $CDCl_3$): δ (ppm): 7.57 (dd, J = 7.7, 1.0 Hz, 1H), 7.32–7.29 (m, 1H), 7.19 (ddd, J = 8.2, 7.0, 1.2 Hz, 1H), 7.13–7.07 (m, 1H), 6.36 (s, 1H), 3.74 (s, 3H), 2.92 (s, 2H), 1.73–1.40 (m, 10H), 1.27 (bs, 1H). **^{13}C{^{1}H} NMR** (126 MHz, $CDCl_3$): δ (ppm): 137.7, 136.5, 127.8, 121.0, 119.9, 119.6, 109.4, 102.1, 71.4, 39.6, 37.6, 30.4, 25.9, 22.2. **HRMS** (ESI+) calcd for $C_{16}H_{22}NO^+$ [M+H]$^+$ 244.1696, found 244.1696.

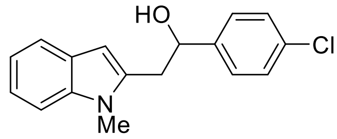

***1-(4-Chlorophenyl)-2-(1-methyl-1H-indol-2-yl)ethan-1-ol** (**2z**):* General procedure II was followed using 4-chlorobenzaldehyde (155 mg, 1.1 mmol) as electrophile except the electrophile was added at -78 ºC and stirred for 1 h at the same temperature after quenching, obtaining **2z**, which was isolated by flash column chromatography (hexane/EtOAc, 3.5/1) as an orange solid (74 mg, 26%). M.p.: 159–161 ºC. R_f = 0.22 (hexane/EtOAc, 3.5/1). **^{1}H NMR** (500 MHz, $CDCl_3$): δ (ppm): 7.58 (d, J = 7.8 Hz, 1H), 7.35-7.28 (m, 5H), 7.22 (dd, J = 8.3, 6.9 Hz, 1H), 7.16–7.08 (m, 1H), 6.39 (s, 1H), 4.96 (t, J = 5.9 Hz, 1H), 3.59 (s, 3H), 3.31–2.95 (m, 2H). **^{13}C{^{1}H} NMR** (126 MHz, $CDCl_3$): δ (ppm): 141.9, 137.7, 136.2, 133.6, 128.8, 127.8, 127.2, 121.4, 120.2, 119.8, 109.3, 101.3, 72.7, 37.3, 29.8. **HRMS** (ESI+) calcd for $C_{17}H_{17}ClNO^+$ [M+H]$^+$ 286.0993, found 286.0994.

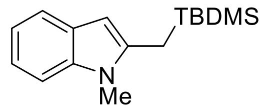

***2-((tert-Butyldimethylsilyl)methyl)-1-methyl-1H-indole** (**3**):* General procedure II was followed using *tert*-butyldimethylsilyl chloride (165 mg, 1.1 mmol) as electrophile obtaining **3**, which was isolated by flash column chromatography (hexane/EtOAc, 100/1) as a pink solid (181 mg, 70%), slightly contaminated with **1a**. M.p.: 49–51 ºC. R_f = 0.33 (hexane/EtOAc, 100/1). **^{1}H NMR** (500 MHz, $CDCl_3$): δ (ppm): 7.60–7.49 (m, 1H), 7.32–7.24 (m, 1H), 7.17–7.11 (m, 1H), 7.09 (td, J = 7.3, 1.2 Hz, 1H), 6.15 (s, 1H), 3.66 (s, 3H), 2.26 (s, 2H), 1.01 (s, 9H), 0.01 (s, 6H). **^{13}C{^{1}H} NMR** (126 MHz, $CDCl_3$): δ (ppm): 139.6, 137.3, 128.6, 119.7, 119.3, 119.0, 108.7, 98.3, 29.8, 26.6, 17.0, 12.5, -6.0. **HRMS** (ESI+) calcd for $C_{16}H_{25}NSi^+$ [M+H]$^+$ 260.1829, found 260.1838.

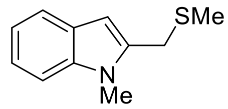

***1-Methyl-2-((methylthio)methyl)-1H-indole** (**4**):* General procedure II was followed using dimethyldisulfide (104 mg, 1.1 mmol) as electrophile obtaining **4**, which was isolated by flash column chromatography (hexane/EtOAc, 9/1) as an orange oil (147 mg, 77%). R_f = 0.5 (hexane/EtOAc, 9/1). **^{1}H NMR** (500 MHz, $CDCl_3$): δ (ppm): 7.59-7.56 (m, 1H), 7.33-7.30 (m, 1H), 7.24-7.20 (m, 1H), 7.13-7.09 (m, 1H), 6.39 (s, 1H), 3.85 (s, 2H), 3.78 (s, 3H), 2.04 (s, 3H). **^{13}C{^{1}H} NMR** (126 MHz, $CDCl_3$): δ (ppm): 138.2, 135.0, 127.3, 121.5, 120.3, 119.6, 109.1, 102.3, 29.9, 29.7, 14.7. **HRMS** (ESI+) calcd for $C_{11}H_{14}NS^+$ [M+H]$^+$ 192.0841, found 192.0843.

Synthesis of 2-benzylindoles 5 from 1a *via* Negishi reaction[S10]

General procedure IV

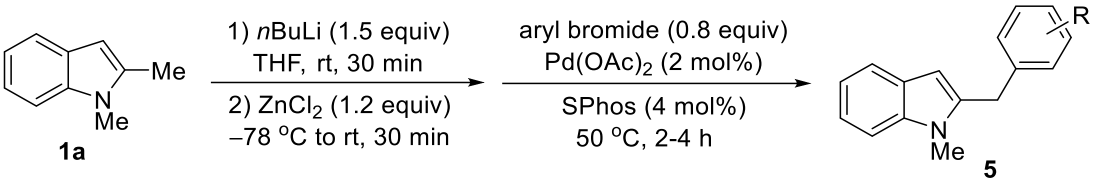

To a stirred solution of 1,2-dimethylindole (**1a**) (145 mg, 1 mmol) in anhydrous THF (2 mL, 0.5 M) was added *n*BuLi (0.6 mL, 1.5 mmol, 2.5 M in hexane) at rt and stirred for 30 min. Then, $ZnCl_2$ (0.6 mL, 1.2 mmol, 2 M in 2-MeTHF) was added at –78 ºC and stirred at rt for 30 min. Next, the corresponding arylbromide (0.8 mmol), SPhos (16mg, 0.04 mmol) and $Pd(OAc)_2$ (4.6 mg, 0.02 mmol) were added, and the resulting mixture was heated at 50 ºC for 2 h (monitored by CG/MS). The resulting mixture was quenched with aq NH_4Cl (10 mL) and extracted with Et_2O (3 × 10 mL). The combined organic layers were dried over anhydrous Na_2SO_4, filtered and concentrated under reduced pressure. The residue was purified by flash column chromatography on silica gel using mixtures of hexane/EtOAc as the eluent to afford the corresponding 2-benzylindoles **5a-i**.

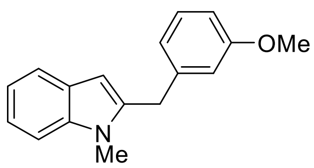

2-(3-Methoxybenzyl)-1-methyl-1H-indole (5a): General procedure IV was followed using 3-bromoanisole (149 mg, 0.8 mmol) as aryl bromide obtaining **5a**, which was isolated by flash column chromatography (hexane/EtOAc, 7/1) as a yellow oil (128 mg, 51%). R_f = 0.46 (hexane/EtOAc, 7/1). **^{1}H NMR** (300 MHz, $CDCl_3$): *δ* (ppm): 7.61 (dd, *J* = 8.0, 1.2 Hz, 1H), 7.40–7.20 (m, 3H), 7.24–7.09 (m, 1H), 6.94–6.74 (m, 3H), 6.35 (s, 1H), 4.17 (s, 2H), 3.81 (s, 3H), 3.61 (s, 3H). **^{13}C{^{1}H} NMR** (75.4 MHz, $CDCl_3$): *δ (*ppm*):* 160.0, 140.2, 139.0, 137.8, 129.7, 127.9, 121.1, 121.0, 120.2, 119.5, 114.6, 111.8, 109.0, 101.3, 55.3, 33.6, 29.9. **HRMS** (ESI+) calcd for $C_{17}H_{18}NO^+$ $[M+H]^+$ 252.1383, found 252.1381.

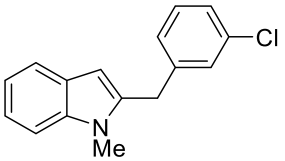

2-(3-Chlorobenzyl)-1-methyl-1H-indole (5b): General procedure IV was followed using 1-bromo-3-chlorobenzene (153 mg, 0.8 mmol) as aryl bromide obtaining **5b**, which was isolated by flash column chromatography (hexane/EtOAc, 10/1) as an orange oil (120 mg, 47%). R_f = 0.30 (hexane/EtOAc, 10/1). **^{1}H NMR** (300 MHz, $CDCl_3$): *δ* (ppm): 7.62 (d, *J* = 8.2 Hz, 1H), 7.47–7.21 (m, 5H), 7.21–7.05 (m, 2H), 6.35 (s, 1H), 4.16 (s, 2H), 3.60 (s, 3H). **^{13}C{^{1}H} NMR** (75.4 MHz, $CDCl_3$): *δ* (ppm): 140.6, 138.2, 137.8, 134.6, 129.9, 128.8, 127.8, 126.9, 121.2, 120.2, 119.6, 109.0, 101.6, 33.2, 29.8. **HRMS** (ESI+) calcd for $C_{16}H_{15}ClN^+$ $[M+H]^+$ 256.0888, found 256.0894.

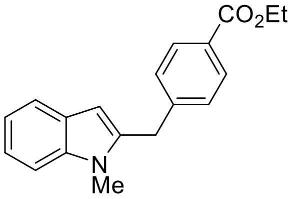

Ethyl 4-((1-methyl-1H-indol-2-yl)methyl)benzoate (5c): General procedure IV was followed using ethyl 4-bromobenzoate (183 mg, 0.8 mmol) as aryl bromide obtaining **5c**, which was isolated by flash column chromatography (hexane/EtOAc, 7/1) as a yellow oil (141 mg, 48%). R_f = 0.35 (hexane/EtOAc, 7/1). **^{1}H NMR** (300 MHz, $CDCl_3$): *δ* (ppm): 8.05 (d, *J* = 8.2 Hz, 2H), 7.63 (d, *J* = 7.8 Hz, 1H), 7.32–7.27 (m, 3H), 7.26–7.10 (m, 2H), 6.34 (s, 1H), 4.43 (q, *J* = 7.1 Hz, 2H), 4.24 (s, 2H), 3.58 (s, 3H), 1.45 (t, *J* = 7.1 Hz, 3H). **^{13}C{^{1}H} NMR** (75.4 MHz, $CDCl_3$): *δ* (ppm): 166.6, 143.8, 138.2, 137.8, 130.0, 129.0, 128.7, 127.8, 121.2, 120.2, 119.6, 109.0, 101.6, 61.0, 33.5, 29.8, 14.4. **HRMS** (ESI+) calcd for $C_{19}H_{20}NO_2^+$ $[M+H]^+$ 294.1489, found 294.1496.

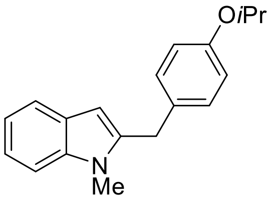

2-(4-Isopropxybenzyl)1-methyl-1H-indole (5d): General procedure IV was followed using 1-bromo-4-isopropoxybenzene (172 mg, 0.8 mmol) as aryl bromide obtaining **5d**, which was isolated by flash column chromatography (hexane/EtOAc, 10/1) as a pink solid (128 mg, 46%). M.p.: 84–86 ºC. R_f = 0.43 (hexane/EtOAc, 10/1). **^{1}H NMR** (300 MHz, $CDCl_3$): *δ* (ppm): 7.61 (d, *J* = 7.8 Hz, 1H), 7.35–7.27 (m, 1H), 7.28–7.17 (m, 1H), 7.19–7.09 (m, 3H), 6.87 (d, *J* = 8.5 Hz, 2H), 6.32 (s, 1H), 4.56 (hept, *J* = 6.1 Hz, 1H), 4.12 (s,

2H), 3.61 (s, 3H), 1.38 (d, *J* = 6.1 Hz, 6H). **^{13}C{^{1}H} NMR** (75.4 MHz, $CDCl_3$): *δ* (ppm): 156.6, 139.7, 137.8, 130.4, 129.7, 127.9, 120.9, 120.1, 119.4, 116.1, 108.9, 101.1, 70.0, 32.7, 29.8, 22.2. **HRMS** (ESI+) calcd for $C_{19}H_{22}NO^+$ $[M+H]^+$ 280.1696, found 280.1703.

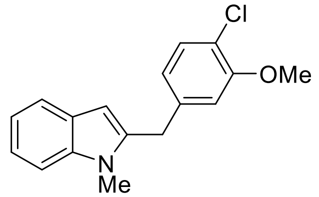

2-(4-Chloro-3-methoxybenzyl)1-methyl-1H-indole (5e): General procedure IV was followed using 5-bromo-2-chloroanisole (177 mg, 0.8 mmol) as aryl bromide obtaining **5e**, which was isolated by flash column chromatography (hexane/EtOAc, 10/1) as an orange oil (151 mg, 53%). R_f = 0.30 (hexane/EtOAc, 10/1). **^{1}H NMR** (300 MHz, $CDCl_3$): *δ* (ppm): 7.59 (d, *J* = 7.8 Hz, 1H), 7.35–7.25 (m, 2H), 7.28–7.16 (m, 1H), 7.18–7.07 (m, 1H), 6.86–6.72 (m, 2H), 6.31 (s, 1H), 4.15 (s, 2H), 3.86 (s, 3H), 3.60 (s, 3H). **^{13}C{^{1}H} NMR** (75.4 MHz, $CDCl_3$): *δ* (ppm): 155.2, 138.7, 138.5, 137.8, 130.3, 127.8, 121.6, 121.2, 120.7, 120.2, 119.6, 112.6, 109.0, 101.5, 56.2, 33.5, 29.9. **HRMS** (ESI+) calcd for $C_{17}H_{17}ClNO^+$ $[M+H]^+$ 286.0993, found 286.0999.

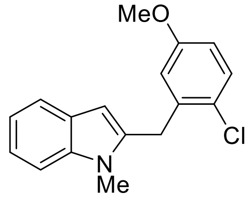

2-(2-Chloro-5-methoxybenzyl)1-methyl-1H-indole (5f): General procedure IV was followed using 2-bromo-1-chloro-4-methoxybenzene (177 mg, 0.8 mmol) as aryl bromide obtaining 5**f**, which was isolated by flash column chromatography (hexane/EtOAc, 10/1) as a yellowish solid (129 mg, 45%). M.p.: 80–82 ºC. R_f = 0.38 (hexane/EtOAc, 10/1). **^{1}H NMR** (300 MHz, $CDCl_3$): *δ* (ppm): 7.60 (d, *J* = 7.8 Hz, 1H), 7.39–7.30 (m, 2H), 7.27–7.20 (m, 1H), 7.18–7.10 (m, 1H), 6.78 (dd, *J* = 8.8, 2.8 Hz, 1H), 6.63-6.62 (m, 1H), 6.30 (s, 1H), 4.24 (s, 2H), 3.71 (s, 3H), 3.63 (s, 3H). **^{13}C{^{1}H} NMR** (75.4 MHz, $CDCl_3$): *δ* (ppm): 158.7, 137.8, 137.7, 137.4, 130.1, 127.9, 125.2, 121.1, 120.2, 119.5, 116.2, 113.3, 109.1, 101.6, 55.6, 31.0, 29.8. **HRMS** (ESI+) calcd for $C_{17}H_{17}ClNO^+$ $[M+H]^+$ 286.0993, found 286.0997.

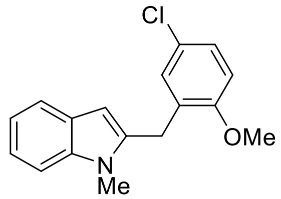

2-(5-Chloro-2-methoxybenzyl)1-methyl-1H-indole (5g): General procedure IV was followed using 2-bromo-4-chloro-1-methoxybenzene (177 mg, 0.8 mmol) as aryl bromide obtaining **5g**, which was isolated by flash column chromatography (hexane/EtOAc, 10/1) as an orange oil (146 mg, 51%). R_f = 0.36 (hexane/EtOAc, 10/1). **^{1}H NMR** (300 MHz, $CDCl_3$): *δ* (ppm) 7.54 (d, *J* = 7.7 Hz, 1H), 7.32–7.23 (m, 1H), 7.23–7.12 (m, 1H), 7.14–7.02 (m, 1H), 6.89 (s, 1H), 6.83-6.81 (m, 2H), 6.23 (s, 1H), 4.06 (s, 2H), 3.87 (s, 3H), 3.57 (s, 3H). **^{13}C{^{1}H} NMR** (75.4 MHz, $CDCl_3$): *δ* (ppm): 157.6, 138.7, 137.7, 133.1, 130.6, 128.0, 125.8, 120.9, 120.7, 120.1, 119.5, 111.1, 109.0, 101.2, 55.8, 29.7, 26.6. **HRMS** (ESI+) calcd for $C_{17}H_{17}ClNO^+$ $[M+H]^+$ 286.0993, found 286.0999.

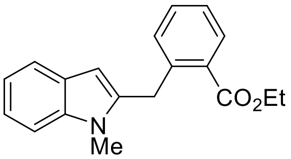

Ethyl 2-((1-methyl-1H-indol-2-yl)methyl)benzoate (5h): General procedure IV was followed using ethyl 2-bromobenzoate (183 mg, 0.8 mmol) as aryl bromide obtaining **5h**, which was isolated by flash column chromatography (hexane/EtOAc, 10/1) as an orange oil (103 mg, 35%). R_f = 0.27 (hexane/EtOAc, 10/1). **^{1}H NMR** (300 MHz, $CDCl_3$): *δ* (ppm): 8.00 (dd, *J* = 7.7, 1.5 Hz, 1H), 7.55 (d, *J* = 7.7 Hz, 1H), 7.48–7.36 (m, 1H), 7.40–7.25 (m, 2H), 7.26–7.15 (m, 1H), 7.16–7.04 (m, 2H), 6.15 (s, 1H), 4.60 (s, 2H), 4.35 (q, *J* = 7.1 Hz, 2H), 3.63 (s, 3H), 1.36 (t, *J* = 7.1 Hz, 3H).**^{13}C{^{1}H} NMR** (75.4 MHz, $CDCl_3$): *δ* (ppm): 167.7, 140.1, 139.5, 137.7, 132.3, 130.9, 130.7, 129.9, 128.0, 126.7, 120.9, 120.1, 119.4, 109.0, 101.3, 61.1, 31.4, 29.8, 14.4. **HRMS** (ESI+) calcd for $C_{19}H_{20}NO_2^+$ $[M+H]^+$ 294.1489, found 294.1496.

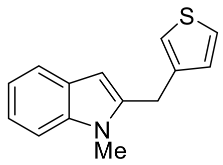

1-Methyl-2-(thiophen-3-ylmethyl)-1H-indole (5i): General procedure IV was followed using 3-bromothiophene (130 mg, 0.8 mmol) as aryl bromide obtaining **5i**, which was isolated by flash column chromatography (hexane/EtOAc, 10/1) as a yellow oil (111 mg, 49%), slightly contaminated with **1a**. R_f = 0.44 (hexane/EtOAc, 10/1). **^{1}H NMR** (300 MHz, $CDCl_3$): *δ* (ppm) 7.61 (d, *J* = 7.7 Hz, 1H), 7.35–7.28 (m, 2H), 7.28–7.17 (m, 1H), 7.19–7.09 (m, 1H), 7.04–6.95 (m, 2H), 6.35 (s, 1H), 4.18 (s, 2H), 3.63 (s, 3H). **^{13}C{^{1}H} NMR** (75.4 MHz, $CDCl_3$): *δ* (ppm): 139.1, 139.0, 137.7,

128.3, 127.9, 125.9, 121.7, 121.0, 120.2, 119.5, 109.0, 100.7, 29.7, 28.5. **HRMS** (ESI+) calcd for $C_{14}H_{14}NS^{+}$ $[M+H]^{+}$ 228.0841, found 228.0848.

Optimization of the lithiation conditions for 5-substituted-1,2-dimethylindoles 1b,e

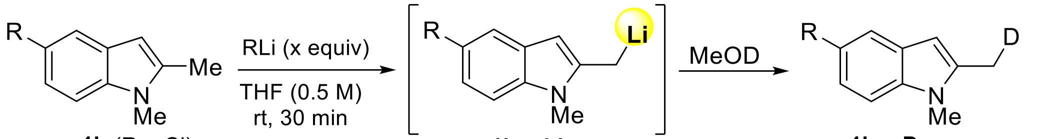

1b (R = Cl) **e** (R = MeO) → **1b,e-Li** → **1b,e-D**

Entry	1	R	RLi (equiv)	Deuteration (%)[b]	Yield (%)[c]
1	**1b**	Cl	*n*BuLi (1.5)	95	89
2	**1e**	OMe	*n*BuLi (1.5)	80	80
3	**1e**	OMe	*n*BuLi (2)	85	77
4	**1e**	OMe	*s*BuLi (1.5)	70	75
5	**1e**	OMe	*t*BuLi (1.5)	>95	83

[a]Reaction conditions: **1b,e** (1 mmol) in THF (2 mL) at rt. [b]The percentage of deuteration was determined by integration of the ^{1}H-NMR spectra. [c]Isolated yield referred to **1b,e**.

When optimal conditions for 1,2-dimethyl indole (**1a**) were essayed with **1b**, almost complete metalation was observed (entry 1). However, the same conditions for **1e** did not result in a complete lithiation (entry 2). After some attempts (entries 3 and 4), with the addition of a stronger base, *t*BuLi, the metalation was achieved in 30 min (entry 5).

Synthesis of 2-substituted 1-methyl-1*H*-indoles 1-D,6, and 7 from 1b-k

General procedure V

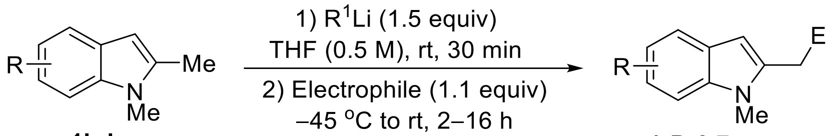

1b-k → **1-D,6,7**

To a stirred solution of the corresponding substituted 1,2-dimethylindole **1b-k** (0.5 mmol) in anhydrous THF (1 mL, 0.5 M) was added the corresponding organolithium reagent (*n*BuLi or *t*BuLi) at rt. The mixture was stirred for 30 min, after which the corresponding electrophile (0.55 mmol) was added at –45 ºC. The resulting solution was stirred at rt until completion (monitored by CG/MS). The resulting mixture was quenched with aq NH_4Cl (10 mL) and extracted with Et_2O (3 × 10 mL). The combined organic layers were dried over anhydrous Na_2SO_4, filtered and concentrated under reduced pressure. The residue was purified by flash column chromatography on silica gel using mixtures of hexane/EtOAc as the eluent to afford the corresponding 2-deuteriomethyl indoles **1-D** and 2-substituted indoles **6**,**7**.

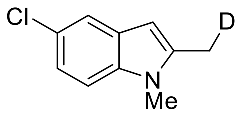

***5-Chloro-2-deuteriomethyl-1-methyl-1H-indole (1b-D)**:* General procedure V was followed using 5-chloro-1,2-dimethylindole (**1b**) (90 mg, 0.5 mmol), *n*BuLi (0.3 mL, 0.75 mmol, 2.5 M in hexane) and MeOD (excess) as electrophile obtaining **1b-D**, as a yellow oil

(79 mg, 89%): **^{1}H NMR** (500 MHz, $CDCl_3$): δ (ppm): 7.49–7.48 (m, 1H), 7.20–7.04 (m, 2H), 6.20 (s, 1H), 3.63 (s, 3H), 2.42–2.40 (m, 2H). **^{13}C{^{1}H} NMR** (126 MHz, $CDCl_3$): δ (ppm): 138.4, 135.8, 129.0, 125.0, 120.6, 119.1, 109.7, 99.4, 29.6, 12.6 (t, $J_{C\text{-}D}$ = 19.5 Hz). **HRMS** (ESI+) calcd for $C_{10}H_{10}DClN^+$ $[M+H]^+$ 181.0637, found 181.0633.

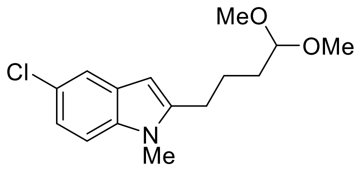

5-Chloro-2-(4,4-dimethoxybutyl)-1-methyl-1H-indole (6a): General procedure V was followed using 5-chloro-1,2-dimethylindole (**1b**) (90 mg, 0.5 mmol), *n*BuLi (0.3 mL, 0.75 mmol, 2.5 M in hexane) and 3-bromo-1,1-dimethoxypropane (100 mg, 0.55 mmol) as electrophile obtaining **6a**, which was isolated by flash column chromatography (hexane/EtOAc, 4/1) as a yellow oil (88 mg, 62%). R_f = 0.30 (hexane/EtOAc, 4/1). R_f = 0.28 (hexane/EtOAc, 4/1). **^{1}H NMR** (300 MHz, $CDCl_3$): δ (ppm): 7.57–7.43 (m, 1H), 7.19–7.04 (m, 2H), 6.21 (s, 1H), 4.43 (t, J = 5.2 Hz, 1H), 3.63 (s, 3H), 3.35 (s, 6H), 2.75 (t, J = 7.0 Hz, 2H), 1.86–1.61 (m, 4H). **^{13}C{^{1}H} NMR** (75.4 MHz, $CDCl_3$): δ (ppm): 142.4, 135.9, 128.9, 124.9, 120.8, 119.2, 109.8, 104.4, 98.7, 52.9, 32.2, 29.6, 26.7, 23.5. **HRMS** (ESI+) calcd for $C_{15}H_{21}ClNO_2^+$ $[M+H]^+$ 282.1255, found 282.1255.

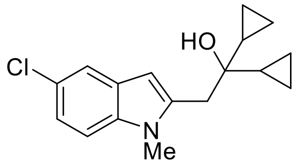

2-(5-Chloro-1-methyl-1H-indol-2-yl)-1,1-dicyclopropylethan-1-ol (6b): General procedure V was followed using 5-chloro-1,2-dimethylindole (**1b**) (90 mg, 0.5 mmol), *n*BuLi (0.3 mL, 0.75 mmol, 2.5 M in hexane) and dicyclopropyl ketone (60 mg, 0.55 mmol) as ectrophile obtaining **6b**, which was isolated by flash column chromatography (hexane/EtOAc, 4/1) as an orange oil (110 mg, 76%). R_f = 0.33 (hexane/EtOAc, 4/1). R_f = 0.31 (hexane/EtOAc, 4/1). **^{1}H NMR** (300 MHz, $CDCl_3$): δ (ppm): 7.54 (dd, J = 2.0, 0.6 Hz, 1H), 7.22 (d, J = 8.7 Hz, 1H), 7.14 (dd, J = 8.7, 2.0 Hz, 1H), 6.36 (s, 1H), 3.76 (s, 3H), 3.09 (s, 2H), 1.21 (s, 1H), 0.90 (tt, J = 8.3, 5.6 Hz, 2H), 0.54–0.19 (m, 8H). **^{13}C{^{1}H} NMR** (75.4 MHz, $CDCl_3$): δ (ppm): 138.5, 136.0, 128.8, 125.2, 121.0, 119.2, 110.2, 101.6, 71.0, 39.2, 30.4, 18.7, 1.4, –0.4. **HRMS** (ESI+) calcd for $C_{17}H_{21}ClNO^+$ $[M+H]^+$ 290.1306, found 290.1308.

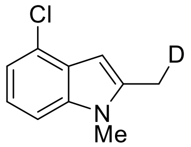

4-Chloro-2-deuteriomethyl-1-methyl-1H-indole (1c-D): General procedure V was followed using 4-chloro-1,2-dimethylindole (**1c**) (90 mg, 0.5 mmol), *n*BuLi (0.3 mL, 0.75 mmol, 2.5 M in hexane) and MeOD (excess) as electrophile obtaining **1c-D**, as a colorless solid (77 mg, 85%). M.p. = 66–68 °C. R_f = 0.25 (hexane/EtOAc, 15/1) **^{1}H NMR** (500 MHz, $CDCl_3$): δ (ppm): 7.21–7.13 (m, 1H), 7.13–7.04 (m, 2H), 6.38 (s, 1H), 3.67 (s, 3H), 2.69–2.16 (m, 2H). **^{13}C{^{1}H} NMR** (126 MHz, $CDCl_3$): δ (ppm): 138.2, 137.8, 126.7, 125.0, 121.1, 119.1, 107.5, 98.4, 29.9, 12.6 (t, $J_{C\text{-}D}$ = 19.5 Hz). **HRMS** (ESI+) calcd for $C_{10}H_{10}DClN^+$ $[M+H]^+$ 181.0637, found 181.0637.

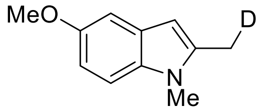

2-Deuteriomethyl-5-methoxy-1-methyl-1H-indole (1e-D): General procedure V was followed using 5-methoxy-1,2-dimethylindole (**1e**) (88 mg, 0.5 mmol), *t*BuLi (0.44 mL, 0.75 mmol, 1.7 M in pentane) and MeOD (excess) as electrophile obtaining **1c-D**, wich was isolated by flash column chromatography (hexane/EtOAc, 3/1) as a brown solid (73 mg, 83%).R_f = 0.4 (hexane/EtOAc, 3/1). M.p.: 61–63 ºC. **^{1}H NMR** (300 MHz, $CDCl_3$): δ (ppm): 7.16 (d, J = 8.8 Hz, 1H), 7.03 (d, J = 2.5 Hz, 1H), 6.90–6.79 (m, 1H), 6.19 (s, 1H), 3.87 (s, 3H), 3.63 (s, 3H), 2.45–2.37 (m, 2H). **^{13}C{^{1}H} NMR** (75.4 MHz, $CDCl_3$): δ (ppm): 154.1, 137.5, 132.8, 128.3, 110.3, 109.4, 102.0, 99.3, 56.1, 29.6, 12.6 (t, $J_{C\text{-}D}$ = 19.4 Hz). **HRMS** (ESI+) calcd for $C_{11}H_{13}DNO^+$ $[M+H]^+$ 177.1133, found 177.1133.

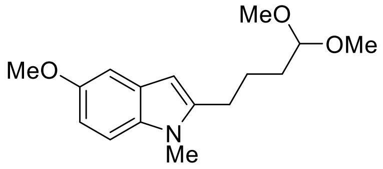

2-(4,4-Dimethoxybutyl)-5-methoxy-1-mehyl-1H-indole (7a): General procedure V was followed using 5-methoxy-1,2-dimethylindole (**1e**) (88 mg, 0.5 mmol), *t*BuLi (0.44 mL, 0.75 mmol, 1.7 M in pentane) and 3-bromo-1,1-dimethoxypropane (100 mg, 0.55 mmol) as electrophile obtaining **7a**, which was isolated by flash column chromatography (hexane/EtOAc, 3/1) as a colourless oil (85 mg, 61%). R_f = 0.33 (hexane/EtOAc, 3/1). **^{1}H NMR** (300 MHz, $CDCl_3$): δ (ppm): 7.16 (d, J = 8.8 Hz, 1H), 7.04 (t, J = 1.9 Hz, 1H), 6.83 (ddd, J = 8.8, 2.5, 1.4 Hz, 1H), 6.21 (s, 1H), 4.49–4.37 (m, 1H), 3.86 (s, 3H), 3.64 (s, 3H), 3.35 (s, 6H), 2.75 (t, J = 7.0 Hz, 2H), 1.86–1.70 (m, 4H). **^{13}C{^{1}H} NMR** (75.4

MHz, $CDCl_3$): δ (ppm): 154.1, 141.5, 132.9, 128.3, 110.5, 109.4, 104.5, 102.2, 98.7, 56.1, 52.9, 32.3, 29.6, 26.8, 23.8. **HRMS** (ESI+) calcd for $C_{16}H_{24}NO_3^+$ $[M+H]^+$ 278.1751, found 278.1749.

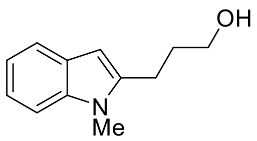

3-(5-Methoxy-1-methyl-1H-indol-2-yl)propan-1-ol (7b): General procedure V was followed using 5-methoxy-1,2-dimethylindole (**1e**) (88 mg, 0.5 mmol), *t*BuLi (0.44 mL, 0.75 mmol, 1.7 M in pentane) and oxirane (0.22 mL, 0.55 mmol, 2.5 M in ether) as electrophile obtaining **7b**, which was isolated by flash column chromatography (hexane/EtOAc, 2/1) as a pink oil (87 mg, 79%). R_f = 0.35 (hexane/EtOAc, 2/1). **^{1}H NMR** (500 MHz, $CDCl_3$): δ (ppm): 7.06 (d, J = 8.8 Hz, 1H), 6.93 (d, J = 2.2 Hz, 1H), 6.73 (dt, J = 8.8, 1.8 Hz, 1H), 6.10 (s, 1H), 3.75 (s, 3H), 3.66 (t, J = 6.3 Hz, 2H), 3.54 (s, 3H), 2.73 (t, J = 7.6 Hz, 2H), 1.91-1.85 (m, 2H). **^{13}C{^{1}H} NMR** (126 MHz, $CDCl_3$): δ (ppm): 154.1, 141.3, 132.9, 128.2, 110.6, 109.5, 102.1, 98.6, 62.3, 56.1, 31.6, 29.6, 23.3. **HRMS** (ESI+) calcd for $C_{13}H_{18}NO_2^+$ $[M+H]^+$ 220.1332, found 220.1333.

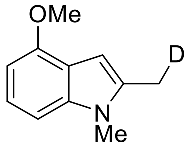

2-Deuteriomethyl-4-methoxy-1-methyl-1H-indole (1f-D): General procedure V was followed using 4-methoxy-1,2-dimethylindole (**1f**) (88 mg, 0.5 mmol), *n*BuLi (0.3 mL, 0.75 mmol, 2.5 M in hexane) and MeOD (excess) as electrophile obtaining **1f-D**, as a colorless solid (70 mg, 80%). M.p.: 120–122 ºC. R_f = 0.25 (hexane/EtOAc, 15/1). **^{1}H NMR** (500 MHz, $CDCl_3$): δ (ppm): 7.12 (t, J = 8.0 Hz, 1H), 6.94 (d, J = 8.0 Hz, 1H), 6.56 (d, J = 8.0 Hz, 1H), 6.39 (s, 1H), 3.99 (s, 3H), 3.67 (s, 3H), 2.71–2.16 (m, 2H). **^{13}C{^{1}H} NMR** (126 MHz, $CDCl_3$): δ (ppm): 152.7, 138.9, 135.3, 121.3, 118.3, 102.6, 99.6, 96.8, 55.5, 29.8, 12.5 (t, $J_{C\text{-}D}$ = 19.6Hz). **HRMS** (ESI+) calcd for $C_{11}H_{13}DNO^+$ $[M+H]^+$ 177.1133, found 177.1133.

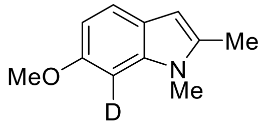

7-Deuterio-6-methoxy-1,2-dimethyl-1H-indole-7 (1g-D): General procedure V was followed using 6-methoxy-1,2-dimethylindole (**1g**) (88 mg, 0.5 mmol), *n*BuLi (0.3 mL, 0.75 mmol, 2.5 M in hexane) and MeOD (excess) as electrophile obtaining **1g-D**, as a colorless solid (72 mg, 82%). M.p.: 70–72 ºC. R_f = 0.24 (hexane/EtOAc, 15/1). **^{1}H NMR** (500 MHz, $CDCl_3$): δ (ppm): 7.42 (d, J = 8.6 Hz, 1H), 6.78 (d, J = 8.6 Hz, 1H), 6.21 (s, 1H), 3.91 (s, 3H), 3.64 (s, 3H), 2.43 (s, 3H). **^{13}C{^{1}H} NMR** (126 MHz, $CDCl_3$): δ (ppm): 155.6, 138.0, 135.8, 122.3, 120.2, 108.7, 99.3, 92.8 (t, $J_{C\text{-}D}$ = 24.1 Hz), 55.9, 29.5, 12.9. **HRMS** could not be recorded.

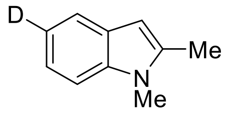

5-Deuterio-1,2-dimethyl-1H-indole (1h-D): General procedure V was followed using 5-bromo-1,2-dimethyl-1*H*-indole (**1h**) (112 mg, 0.5 mmol), *t*BuLi (1.03 mL, 1.75 mmol, 1.7 M in pentane) and MeOD (excess) as electrophile obtaining **1h-D**, as a colorless solid (50 mg, 69%). M.p. = 55–57 °C. R_f = 0.31 (hexane/EtOAc, 15/1). ~85% D incorporation. **^{1}H NMR** (500 MHz, $CDCl_3$): δ (ppm): 7.60 (s, 1H), 7.33 (d, J = 8.2 Hz, 1H), 7.23 (d, J = 8.2 Hz, 1H), 6.33 (s, 1H), 3.71 (s, 3H), 2.49 (s, 3H). **^{13}C{^{1}H} NMR** (126 MHz, $CDCl_3$): δ (ppm): 137.4, 136.9, 128.1, 120.4, 119.6, 118.9 (t, $J_{C\text{-}D}$ = 24.2 Hz), 108.8, 99.7, 29.4, 12.8.

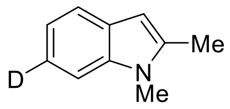

6-Deuterio-1,2-dimethyl-1H-indole (1i-D): General procedure V was followed using 6-bromo-1,2-dimethyl-1*H*-indole (**1i**) (112 mg, 0.5 mmol), *t*BuLi (1.03 mL, 1.75 mmol, 1.7 M in pentane) and MeOD (excess) as electrophile obtaining **1i-D**, as a colorless solid (75 mg, 77%). M.p. = 77–79 °C. R_f = 0.31 (hexane/EtOAc, 15/1). ~85% D was incorporated at C-6 and ~10% D was observed at the methyl group. **^{1}H NMR** (500 MHz, $CDCl_3$): δ (ppm): 7.59 (d, J = 7.8 Hz, 1H), 7.31 (s, 1H), 7.13 (d, J = 7.8 Hz, 1H), 6.31 (s, 1H), 3.71 (s, 3H), 2.48 (s, 3H). **^{13}C{^{1}H} NMR** (126 MHz, $CDCl_3$): δ (ppm): 136.8, 128.0, 120.5, 120.0 (t, $J_{C\text{-}D}$ = 24.1 Hz), 119.6, 119.2, 108.6, 99.6, 29.4, 12.8.

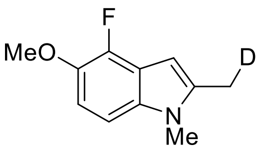

2-Deuteriomethyl-4-fluoro-5-methoxy-1-methyl-1H-indole (1k-D): General procedure V was followed using 4-fluoro-5-methoxy-1,2-dimethyl-1*H*-indole (**1k**) (97 mg, 0.5 mmol), *n*BuLi (0.3 mL, 0.75 mmol, 2.5 M in hexane) and MeOD (excess) as electrophile obtaining **1k-D**, as a colorless solid (75 mg, 77%). M.p = 70–72 °C. R_f = 0.23 (hexane/EtOAc, 10/1):

^{1}H NMR (500 MHz, $CDCl_3$): δ (ppm): 7.00–6.85 (m, 2H), 6.31 (s, 1H), 3.96 (s, 3H), 3.63 (s, 3H), 2.67–2.34 (m, 2H). **^{13}C{^{1}H} NMR** (126 MHz, $CDCl_3$): δ (ppm): 145.4 (d, J = 244.8 Hz), 139.9 (d, J = 9.5 Hz), 138.0, 135.3 (d, J = 10.7 Hz), 118.0 (d, J = 19.6 Hz), 110.5, 104.0 (d, J = 4.0 Hz), 95.3, 59.0, 29.7, 12.5 (t, $J_{C\text{-}D}$ = 19.5 Hz). **HRMS** could not be recorded.

Optimization of the lithiation conditions for 1,2,3-trimethylindole (1m)

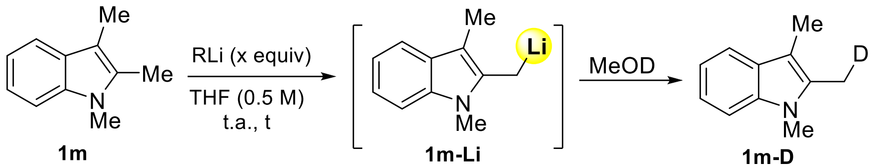

1m **1m-Li** **1m-D**

Entry	RLi (equiv)	t (min)	Deuteration (%)[b]	Yield (%)[c]
1	*n*BuLi (1.5)	30	50	81
2	*s*BuLi (1.5)	30	50	80
3	*s*BuLi (1.5)	60	70	77
4	*s*BuLi (2)	60	85	80
5	*t*BuLi (1.5)	30	92	83

[a]Reaction conditions: **1d** (80 mg, 0.5 mmol) in THF (1 mL) at rt. [b]The percentage of deuteration was determined by integration of the ^{1}H-NMR spectra. [c]Isolated yield referred to **1m**.

First, standard conditions optimized for **1a** were tested with **1m**, but complete lithiation was not achieved (entry 1). The addition of a stronger base, *s*BuLi, and extended time, led to better deuteration (entries 2–4). Finally, almost complete lithiation was achieved using *t*BuLi as base (entry 5).

Synthesis of 2-substituted 1,3-dimethyl-1*H*-indole 1m-D and 8a from 1m

General procedure VI

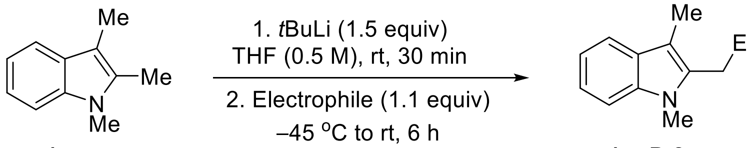

1m **1m-D,8a**

To a stirred solution of 1,2,3-trimethylindole (**1m**) (159 mg, 1 mmol) in anhydrous THF (2 mL, 0.5 M) was added *t*-BuLi (0.88 mL, 1.5 mmol, 1.7 M in pentane) at rt and stirred for 30 min. Then, the corresponding electrophile (1.1 mmol) was added at –45 °C and the resulting solution was stirred at rt until completion (monitored by CG/MS). The resulting mixture was quenched with aq NH_4Cl (10 mL) and extracted with Et_2O (3 × 10 mL). The combined organic layers were dried over anhydrous Na_2SO_4, filtered and concentrated under reduced pressure. The residue was purified by flash column chromatography on silica gel using mixtures of hexane/EtOAc as the eluent to afford the 2,3-disubstituted indoles **1m-D** and **8a**.

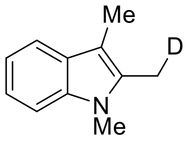

2-Deuteriomethyl-1,3-dimethyl-1H-indole (1m-D): General procedure VI was followed using MeOD (excess) as electrophile obtaining **1m-D**, as a yellow oil (133 mg, 83%). **^{1}H NMR** (300 MHz, $CDCl_3$): δ (ppm): 7.54 (d, J = 7.5 Hz, 1H), 7.32–7.23 (m, 1H), 7.23–7.17 (m, 1H), 7.16–7.08 (m, 1H), 3.67 (s, 3H), 2.38 (d, J = 4.5, 1H), 2.37 (d, J = 2.0 Hz, 1H), 2.31 (s, 3H).

^{13}**C{^{1}H} NMR** (75.4 MHz, CDCl$_3$): δ (ppm): 128.5, 120.6, 118.7, 118.0, 108.5, 106.3, 29.6, 10.0 (t, J_{C-D} = 19.8 Hz), 8.9, two carbon signals are missing due to overlapping. **HRMS** (ESI+) calcd for $C_{11}H_{13}DN^+$ [M+H]$^+$ 161.1184, found 161.1176.

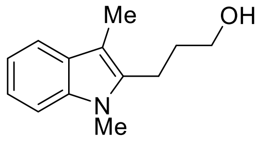

3-(1,3-Dimethyl-1H-indol-2-yl)propan-1-ol (8a): General procedure VI was followed using oxirane (0.44 mL, 1.1 mmol, 2.5 M in ether) as electrophile obtaining **8a**, which was isolated by flash column chromatography (hexane/EtOAc, 1/1) as an orange oil (122 mg, 60%). R_f = 0.16 (hexane/EtOAc, 1/1). ^{1}**H NMR** (300 MHz, CDCl$_3$): δ (ppm): 7.67–7.54 (m, 1H), 7.37–7.11 (m, 3H), 3.77–3.66 (m, 5H), 2.93 (t, J = 7.5 Hz, 2H), 2.37 (s, 3H), 2.02–1.67 (m, 3H). ^{13}**C{^{1}H} NMR** (75.4 MHz, CDCl$_3$): δ (ppm): 136.7, 136.2, 128.4, 120.8, 118.7, 118.1, 108.7, 106.6, 62.0, 32.5, 29.6, 20.7, 8.9. **HRMS** (ESI+) calcd for $C_{13}H_{18}NO^+$ [M+H]$^+$ 204.1383, found 204.1389.

Optimization of the lithiation conditions for 2-methyl-*N*-phenylindole (1n)

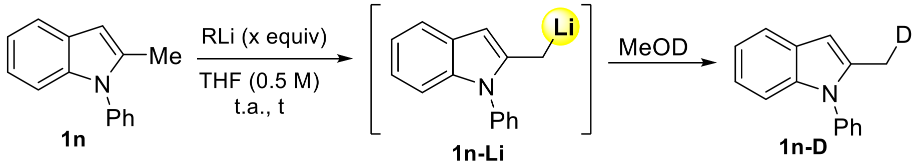

Entry	RLi (equiv)	t (min)	Deuteration (%)[b]	Yield (%)[c]
1	*n*BuLi (1.5)	30	30	84
2	*n*BuLi (1.5)	60	50	86
3	*s*BuLi (1.5)	60	0	82
4	*n*BuLi (2)	60	90	88

[a]Reaction conditions: **1n** (104 mg, 0.5 mmol) in THF (1 mL) at rt. [b]The percentage of deuteration was determined by integration of the ^{1}H-NMR spectra. [c]Isolated yield referred to **1n**.

First, optimized conditions for **1a** were tested with **1n**, but complete lithiation was not achieved (entry 1). Upon increasing the reaction time no complete deuteration was obtained (entry 2). Changing to *s*BuLi did not provide a better result (entry 3). Finally, the addition of an excess of *n*BuLi led to a higher percentage of deuteration (entry 4).

Synthesis of 2-substituted 1-phenyl-1*H*-indole 1n-D and 9a from 1n

General procedure VII

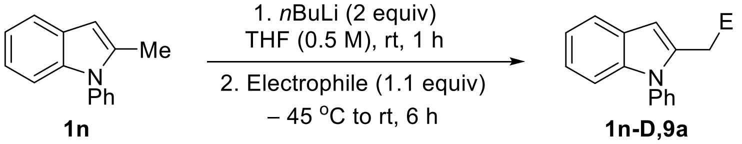

To a stirred solution of 2-methyl-1-phenylindole (**1n**) (207 mg, 1 mmol) in anhydrous THF (2 mL, 0.5 M) was added *n*BuLi (0.8 mL, 2 mmol, 2.5 M in hexane) at rt and stirred for 1 h. Then, the corresponding electrophile (1.1 mmol) was added at –45 °C and the resulting solution was stirred at rt until completion (monitored by CG/MS). The resulting mixture was quenched with aq NH_4Cl (10 mL) and extracted with Et_2O (3 × 5 mL). The combined organic layers were dried over anhydrous Na_2SO_4, filtered and concentrated under reduced pressure. The residue was purified by flash column chromatography on silica gel using mixtures of hexane/EtOAc as the eluent to afford the 2-substituted indoles **1n-D** and **9a**.

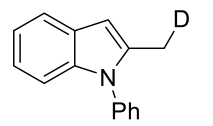

2-Deuteriomethyl-1-phenyl-1H-indole (1n-D): General procedure VII was followed using MeOD (excess) as electrophile, obtaining **1en-D** as a yellow oil (183 mg, 88%). **^{1}H NMR** (300 MHz, $CDCl_3$): δ (ppm): 7.63–7.53 (m, 3H), 7.51–7.44 (m, 1H), 7.43–7.36 (m, 2H), 7.24–7.05 (m, 3H), 6.44 (s, 1H), 2.41–2.26 (m, 2H). **^{13}C{^{1}H} NMR** (75.4 MHz, $CDCl_3$): δ (ppm): 138.3, 138.1, 137.1, 129.5, 128.3, 128.1, 127.8, 121.2, 120.1, 119.7, 110.1, 101.4, 13.26 (t, $J_{C\text{-}D}$ = 19.0 Hz). **HRMS** (ESI+) calcd for $C_{15}H_{13}DN^+$ [M+H]$^+$ 209.1184, found 209.1187.

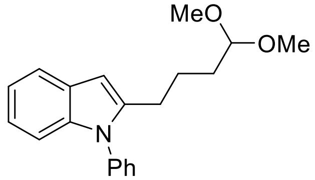

2-(4,4-Dimethoxybutyl)-1-phenyl-1H-indole (9a): General procedure VII was followed using 3-bromo-1,1-dimethoxypropane (201 mg, 1.1 mmol) as electrophile obtaining **9a**, which was isolated by flash column chromatography (hexane/EtOAc, 7/1) as an orange oil (192 mg, 62%). R_f = 0.29 (hexane/EtOAc, 7/1). **^{1}H NMR** (300 MHz, $CDCl_3$): δ (ppm): 7.66–7.52 (m, 3H), 7.49 (d, J = 6.7 Hz, 1H), 7.40–7.35 (m, 2H), 7.18–7.06 (m, 3H), 6.48 (s, 1H), 4.33 (t, J = 4.9 Hz, 1H), 3.29 (s, 6H), 2.69 (t, J = 6.9 Hz, 2H), 1.79–1.53 (m, 4H). **^{13}C{^{1}H} NMR** (75.4 MHz, $CDCl_3$): δ (ppm): 141.3, 138.4, 138.1, 129.6, 128.4, 128.2, 128.0, 121.2, 120.1, 119.8, 110.1, 104.3, 100.5, 52.7, 32.1, 26.9, 23.8. **HRMS** (ESI+) calcd for $C_{20}H_{24}NO_2^+$ [M+H]$^+$ 310.1802, found 310.1810.

Synthesis of 2-difunctionalized indoles 10

General procedure VIII

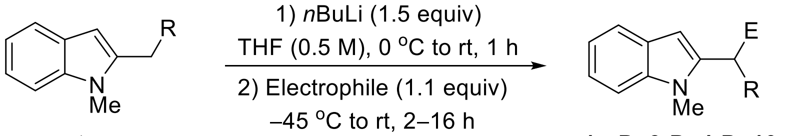

1o (R^1=Ph)
3 (R^1=TBDMS)
4 (R^1=SMe)

1o-D, 3-D, 4-D, 10

To a stirred solution of the corresponding indole (1 mmol) in anhydrous THF (2 mL, 0.5 M) was added *n*BuLi (0.6 mL, 1.5 mmol, 2.5 M in hexane) at 0 °C and stirred at rt for 1 h. Then, the corresponding electrophile (1.1 mmol) was added at –45 °C and the resulting solution was stirred at rt until completion (monitored by CG/MS). The resulting mixture was quenched with aq NH_4Cl (10 mL) and extracted with Et_2O (3 × 5 mL). The combined organic layers were dried over anhydrous Na_2SO_4, filtered and concentrated under reduced pressure. The residue was purified by flash column chromatography on silica gel using mixtures of hexane/EtOAc as the eluent to afford the corresponding 2-substituted indoles **10**.

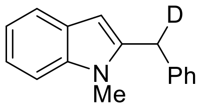

2-α-Deuteriobenzyl-1H-indole (1o-D): General procedure VIII was followed using 2-benzyl-1-methyl-1*H*-indole (**1f**) (221 mg, 1 mmol) and MeOD (excess) as electrophile obtaining **1f-D** as an orange oil (189 mg, 85%). Contaminated with unidentified byproducts. **^{1}H NMR** (300 MHz, $CDCl_3$): δ (ppm): 7.70–7.51 (m, 1H), 7.46–7.21 (m, 8H), 6.35 (s, 1H), 4.24 (s, 1H), 3.61 (s, 3H).

$^{13}C\{^1H\}$ NMR (75.4 MHz, $CDCl_3$): *δ* (ppm): 141.8, 128.6, 128.4, 128.3, 128.2, 126.5, 120.9, 120.1, 119.4, 108.9, 101.2, 33.7 (t, $J_{C\text{-}D}$ = 19.4 Hz), 29.8. **HRMS** (ESI+) could not be recorded.

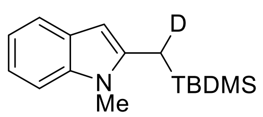

2-(Tert-butyldimethylsilyl)deuteriomethyl-1-methyl-1H-indole (3-D): General procedure VIII was followed using 2-((*tert*-butyldimethylsilyl)methyl)-1-methyl-1*H*-indole (**3**) (259 mg, 1 mmol) and MeOD (excess) as electrophile obtaining **3-D** as a pink oil (219 mg, 84%). **^1H NMR** (500 MHz, $CDCl_3$): *δ* (ppm): 7.53 (t, *J* = 6.5 Hz, 1H), 7.36–7.24 (m, 1H), 7.21–7.01 (m, 2H), 6.15 (s, 1H), 3.66 (s, 3H), 2.25 (s, 1H), 1.01 (s, 9H), 0.01 (s, 6H). **$^{13}C\{^1H\}$ NMR** (126 MHz, $CDCl_3$): *δ* (ppm): 139.6, 137.3, 128.6, 119.7, 119.3, 119.0, 108.7, 98.3, 29.8, 26.6, 17.0, 12.14 (t, $J_{C\text{-}D}$ = 18.8 Hz), -6.03. **HRMS** (ESI+) could not be recorded.

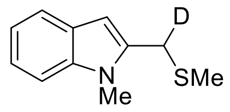

2-(Deuterio)methylthiomethyl-1-methyl-1H-indole (4-D): General procedure VIII was followed using 1-methyl-2-((methylthio)methyl)-1*H*-indole (**4**) (191 mg, 1 mmol) and MeOD (1 mL) obtaining **4-D** as a red oil (167 mg, 87%): **^1H NMR** (500 MHz, $CDCl_3$): *δ* (ppm): 7.57 (dd, *J* = 7.8, 1.0 Hz, 1H), 7.34–7.28 (m, 1H), 7.25–7.18 (m, 1H), 7.14–7.08 (m, 1H), 6.38 (s, 1H), 3.87–3.81 (m, 1H), 3.77 (s, 3H), 2.03 (s, 3H). **$^{13}C\{^1H\}$ NMR** (126 MHz, $CDCl_3$): *δ* (ppm): 138.2, 134.9, 127.3, 121.6, 120.3, 119.6, 102.3, 29.8, 29.6 (t, $J_{C\text{-}D}$ = 20.6 Hz), 14.7. **HRMS** (ESI+) calcd for $C_{11}H_{12}DNS^+$ $[M+H]^+$ 193.0904, found 193.0906.

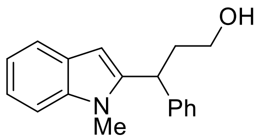

3-(1-Methyl-1H-indol-2-yl)-3-phenylpropan-1-ol (10a): General procedure VIII was followed using 2-benzyl-1-methyl-1*H*-indole (**1f**) (221 mg, 1 mmol) and oxirane (0.44 mL, 1.1 mmol, 2.5 M in ether) as electrophile obtaining **10a**, which was isolated by flash column chromatography (hexane/EtOAc, 4/1) as a yellow oil (141 mg, 53%). R_f = 0.17 (hexane/EtOAc, 4/1). **^1H NMR** (500 MHz, $CDCl_3$): *δ* (ppm): 7.87–7.59 (m, 1H), 7.39–7.20 (m, 7H), 7.20–7.03 (m, 1H), 6.58 (s, 1H), 4.39 (t, *J* = 7.7 Hz, 1H), 3.88–3.75 (m, 1H), 3.73–3.64 (m, 1H), 3.49 (s, 3H), 2.51 (dq, *J* = 13.4, 6.6 Hz, 1H), 2.28-2.21 (m, 1H), 1.60 (bs, 1H). **$^{13}C\{^1H\}$ NMR** (126 MHz, $CDCl_3$): *δ* (ppm): 142.7, 142.7, 137.7, 128.8, 128.2, 127.7, 126.8, 121.1, 120.2, 119.4, 108.9, 99.2, 60.5, 39.9, 38.8, 29.8. **HRMS** (ESI+) calcd for $C_{18}H_{20}NO^+$ $[M+H]^+$ 266.1539, found 266.1548.

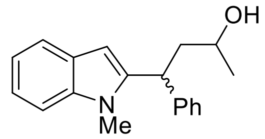

4-(1-Methyl-1H-indol-2-yl)-4-phenylbutan-2-ol (10b): General procedure VIII was followed using 2-benzyl-1-methyl-1*H*-indole (**1f**) (221 mg, 1 mmol) and 2-methyloxirane (64 mg, 1.1 mmol) as electrophile obtaining **10b** as a c.a. 1/1 mixture of diastereoisomers, which were isolated by flash column chromatography (hexane/EtOAc, 3/1) as a c.a. 1/1 mixture of diastereoisomers. Yellow oil (173 mg, 62%). R_f = 0.15 (hexane/EtOAc, 3/1). **^1H NMR** (500 MHz, $CDCl_3$): *δ* (ppm): 7.68–7.61 (m, 2H, both diast), 7.38–7.17 (m, 14H, both diast), 7.15–7.09 (m, 2H, both diast), 6.56 (s, 1H, one diast), 6.52 (s, 1H, one diast), 4.53–4.38 (m, 2H, both diast), 4.06-4.00 (m, 1H, one diast), 3.77-3.71 (m, 1H, one diast), 3.51 (s, 3H, one diast), 3.49 (s, 3H, one diast), 2.40–2.27 (m, 2H, both diast), 2.18–2.02 (m, 2H, both diast), 1.41 (bs, 2H, both diast), 1.32 (d, *J* = 6.3 Hz, 3H, one diast), 1.26 (d, *J* = 6.2 Hz, 3H, one diast). **$^{13}C\{^1H\}$ NMR** (126 MHz, $CDCl_3$): *δ* (ppm): 143.6, 143.5, 142.5, 142.2, 137.7, 128.9, 128.8, 128.5, 127.9, 127.7, 126.8, 126.7, 121.10, 121.07, 120.2, 119.5, 119.4, 109.0, 108.9, 99.3, 99.1, 66.1, 65.3, 46.2, 45.2, 40.5, 40.3, 29.8, 29.8, 24.7, 24.3, five carbon signals are missing due to overlapping. **HRMS** (ESI+) calcd for $C_{19}H_{22}NO$ $[M+H]^+$ 280.1696, found 280.1703.

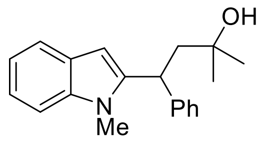

2-Methyl-4-(1-methyl-1H-indol-2-yl)-4-phenylbutan-2-ol (10c): General procedure VIII was followed using 2-benzyl-1-methyl-1*H*-indole (**1f**) (221 mg, 1 mmol) and 2,2-dimethyloxirane (79 mg, 1.1 mmol) as electrophile obtaining **10c**, which was isolated by flash column chromatography (hexane/EtOAc, 2/1) as an orange oil (147 mg, 50%). R_f = 0.43 (hexane/EtOAc, 2/1). **^1H NMR** (500 MHz, $CDCl_3$): *δ* (ppm): 7.81–7.59 (m, 1H), 7.40–7.27 (m, 5H), 7.25–7.18 (m, 2H), 7.18–7.06 (m, 1H), 6.69–6.51 (m, 1H), 4.63–4.44 (m, 1H), 3.59 (s, 3H), 2.60 (dd, *J* = 14.5, 7.5 Hz, 1H), 2.26 (dd, *J* = 14.5, 5.5 Hz, 1H), 1.58–1.45 (m, 1H), 1.30 (s, 3H), 1.27 (s, 3H). **$^{13}C\{^1H\}$ NMR**

(126 MHz, $CDCl_3$): δ (ppm): 144.0, 143.6, 137.7, 128.9, 128.1, 127.7, 126.6, 121.2, 120.3, 119.5, 109.0, 99.5, 71.2, 49.4, 39.5, 30.5, 30.3, 29.9. **HRMS** (ESI+) calcd for $C_{20}H_{24}NO$ $[M+H]^+$ 294.1852, found 294.1854.

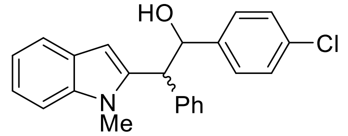

1-(4-Chlorophenyl)-2-(1-methyl-1H-indol-2-yl)-2-phenylethan-1-ol (10d): General procedure VIII was followed using 2-benzyl-1-methyl-1*H*-indole (**1f**) (221 mg, 1 mmol) and 4-chlorobenzaldehyde (155 mg, 1.1 mmol) as electrophile obtaining **10d** as a c.a. 1/1mixture of diastereoisomers, which was isolated by flash column chromatography (hexane/EtOAc, 5/1) as a c.a. 6/1 mixture of diastereoisomers. Orange oil (228 mg, 63%). R_f = 0.33 (hexane/EtOAc, 5/1). Data for major diastereoisomer: **^{1}H NMR** (500 MHz, $CDCl_3$): δ (ppm): 7.72 (t, *J* = 8.0 Hz, 1H), 7.42–7.00 (m, 10H), 6.94-6.90 (m, 2H), 6.86 (d, *J* = 3.2 Hz, 1H), 5.29 (dd, *J* = 9.2, 1.9 Hz, 1H), 4.32 (dd, *J* = 9.3, 5.2 Hz, 1H), 3.51 (s, 3H), 3.08–3.02 (m, 1H). **^{13}C{^{1}H} NMR** (126 MHz, $CDCl_3$): δ 139.7, 139.5, 138.1, 137.8, 133.4, 129.0, 128.5, 128.4, 128.2, 127.7, 127.2, 121.7, 120.5, 119.9, 109.2, 98.9, 76.9, 54.1, 29.8. **HRMS** (ESI+) calcd for $C_{23}H_{21}ClNO^+$ $[M+H]^+$ 362.1306, found 362.1314.

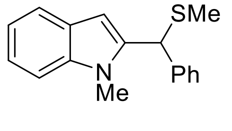

1-Methyl-2-((methylthio)(phenyl)methyl)-1H-indole (10e): General procedure VIII was followed using 2-benzyl-1-methyl-1*H*-indole (**1f**) (221 mg, 1 mmol) and dimethyldisulfide (104 mg, 1.1 mmol) as electrophile obtaining **10e**, which was isolated by flash column chromatography (hexane/EtOAc, 15/1) as an orange oil (107 mg, 40%). R_f = 0.3 (hexane/EtOAc, 15/1). **^{1}H NMR** (300 MHz, $CDCl_3$): δ (ppm): 7.62 (d, *J* = 7.8 Hz, 1H), 7.51–7.43 (m, 2H), 7.41–7.27 (m, 4H), 7.23 (t, *J* = 7.5 Hz, 1H), 7.18–7.09 (m, 1H), 6.60 (s, 1H), 5.24 (s, 1H), 3.65 (s, 3H), 2.12 (s, 3H). **^{13}C{^{1}H} NMR** (75.4 MHz, $CDCl_3$): δ (ppm): 139.4, 138.8, 138.2, 128.7, 128.6, 127.6, 127.4, 121.6, 120.6, 119.6, 109.1, 102.6, 48.5, 30.1, 16.0. **HRMS** (ESI+) calcd for $C_{17}H_{18}NS^+$ $[M+H]^+$ 268.1154, found 268.1156.

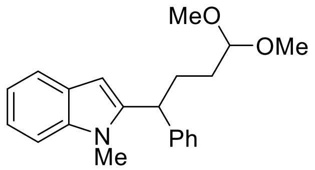

2-(4,4-Dimethoxy-1-phenylbutyl)-1-methyl-1H-indole (10f): General procedure VIII was followed using 2-benzyl-1-methyl-1*H*-indole (**1f**) (221 mg, 1 mmol) and 3-bromo-1,1-dimethoxypropane (201 mg, 1.1 mmol) as electrophile obtaining **10f**, which was isolated by flash column chromatography (hexane/EtOAc, 7/1) as an orange oil (174 mg, 54%). R_f = 0.18 (hexane/EtOAc, 7/1). **^{1}H NMR** (300 MHz, $CDCl_3$): δ (ppm): 7.79–7.66 (m, 2H), 7.41–7.12 (m, 7H), 6.67 (s, 1H), 4.58–4.42 (m, 1H), 4.15 (t, *J* = 7.3 Hz, 1H), 3.53 (s, 3H), 3.42 (s, 3H), 3.37 (s, 3H), 2.44–2.27 (m, 1H), 2.25–2.04 (m, 1H), 2.03–1.82 (m, 1H), 1.82–1.68 (m, 1H). **^{13}C{^{1}H} NMR** (75.4 MHz, $CDCl_3$): δ (ppm): 143.0, 142.9, 137.6, 128.7, 128.1, 127.7, 126.7, 120.2, 120.2, 119.3, 108.8, 104.5, 99.2, 52.9, 52.7, 43.8, 31.3, 31.0, 29.7. **HRMS** (ESI+) calcd for $C_{21}H_{26}NO_2^+$ $[M+H]^+$ 324.1958, found 324.1966.

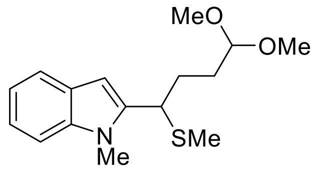

2-(4,4-Dimethoxy-1-(methylthio)butyl)-1-methyl-1H-indole (10g): General procedure VIII was followed using 1-methyl-2-((methylthio)methyl)-1*H*-indole (**4**) (191 mg, 1 mmol) and 3-bromo-1,1-dimethoxypropane (201 mg, 1.1 mmol) as electrophile obtaining **10g**, which was isolated by flash column chromatography (hexane/EtOAc, 7/1) as an orange oil (141 mg, 48%). R_f = 0.23 (hexane/EtOAc, 7/1). **^{1}H NMR** (300 MHz, $CDCl_3$): δ (ppm): 7.61 (d, *J* = 7.8 Hz, 1H), 7.35 (d, *J* = 8.2 Hz, 1H), 7.33–7.20 (m, 1H), 7.13 (tap, *J* = 7.4 Hz, 1H), 6.43 (s, 1H), 4.47 (t, *J* = 5.5 Hz, 1H), 3.97 (t, *J* = 7.6 Hz, 1H), 3.82 (s, 3H), 3.36 (s, 6H), 2.24–2.07 (m, 2H), 1.98–1.76 (m, 5H). **^{13}C{^{1}H} NMR** (75.4 MHz, $CDCl_3$): δ (ppm): 138.6, 137.9, 127.3, 121.5, 120.3, 119.5, 109.0, 104.3, 100.9, 53.0, 52.9, 41.5, 30.8, 29.8, 28.2, 11.5. **HRMS** (ESI+) calcd for $C_{16}H_{24}NO_2S^+$ $[M+H]^+$ 294.1522, found 294.1523.

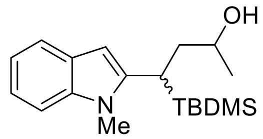

4-(Tert-butyldimethylsilyl)-4-(1-methyl-1H-indol-2-yl)butan-2-ol (10h): General procedure VIII was followed using 2-((*tert*-butyldimethylsilyl)methyl)-1-methyl-1*H*-indole (**3**) (259 mg, 1 mmol) and 2-methyloxirane (64 mg, 1.1 mmol) as electrophile obtaining **10h** as c.a. 2/1 mixture of diastereoisomers, which was isolated by flash column chromatography (hexane/EtOAc, 3/1) as a c.a. 10/1 mixture of diastereoisomers. Data for major diastereoisomer: yellow oil (111 mg, 35%). R_f = 0.3 (hexane/EtOAc, 3/1). **^{1}H NMR** (300 MHz, $CDCl_3$): δ (ppm):

7.63–7.46 (m, 1H), 7.27 (d, J = 5.9 Hz, 1H), 7.21–7.02 (m, 2H), 6.25 (s, 1H), 3.81-3.72 (m, 1H) 3.69 (s, 3H), 2.52-2.48 (m, 1H), 2.17 (ddd, J = 13.1, 12.5, 5.3 Hz, 1H), 1.99 (bs, 1H), 1.18 (d, J = 6.1 Hz, 3H), 0.99 (s, 9H), 0.14 (s, 3H), –0.31 (s, 3H). **^{13}C{^{1}H} NMR** (75.4 MHz, $CDCl_3$): δ (ppm): 143.9, 136.9, 128.5, 120.0, 119.4, 119.2, 108.8, 97.3, 68.6, 41.1, 29.6, 27.4, 22.7, 20.9, 17.8, –6.8, –8.1. **HRMS** (ESI+) calcd for $C_{19}H_{32}NOSi$ [M+H]$^+$ 318.2248, found 318.2250.

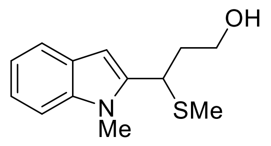

***3-(1-Methyl-1H-indol-2-yl)-3-(methylthio)propan-1-ol (10i)**:* General procedure VIII was followed using 1-methyl-2-((methylthio)methyl)-1*H*-indole (**4**) (191 mg, 1 mmol) and oxirane (0.44 mL, 1.1 mmol, 2.5 M in ether) as electrophile obtaining **10i**, which was isolated by flash column chromatography (hexane/EtOAc, 2/1) as a yellow oil (122 mg, 52%). R_f = 0.45 (hexane/EtOAc, 2/1). **^{1}H NMR** (300 MHz, $CDCl_3$): δ (ppm): 7.67–7.54 (m, 1H), 7.35 (d, J = 8.2 Hz, 1H), 7.29–7.19 (m, 1H), 7.14 (tap, J = 7.4 Hz, 1H), 6.41 (s, 1H), 4.24 (t, J = 7.6 Hz, 1H), 4.01–3.89 (m, 1H), 3.83 (s, 4H), 2.50–2.22 (m, 2H), 1.89 (bs, 1H), 1.90 (s, 3H). **^{13}C{^{1}H} NMR** (75.4 MHz, $CDCl_3$): δ (ppm): 138.4, 138.0, 127.2, 121.6, 120.3, 119.6, 109.1, 100.9, 60.7, 38.3, 35.4, 29.9, 11.3. **HRMS** (ESI+) calcd for $C_{13}H_{18}NOS$ [M+H]$^+$ 236.1104, found 236.1111.

Synthesis of 2u in Deep Eutectic Solvents

Preparation of Deep Eutectic Solvents (DESs)

Deep Eutectic Solvents (DESs) have been obtained according to a previous report.[S11] To a round-bottom flask, choline chloride (5 mmol, 712 mg) and 10 mmol of the corresponding hydrogen donor (0.72 mL of glycerol, or 0.18 mL of water, or 0.74 mL of lactic acid) were added. The resultant solid mixture was heated at 80 ºC for 20 min to yield a clear solution. The obtained 1 *ChCl/*2 *Gly*, 1 *ChCl/*2 *H_2O, and* 1 *ChCl/*2 *LA* were used without further purification.

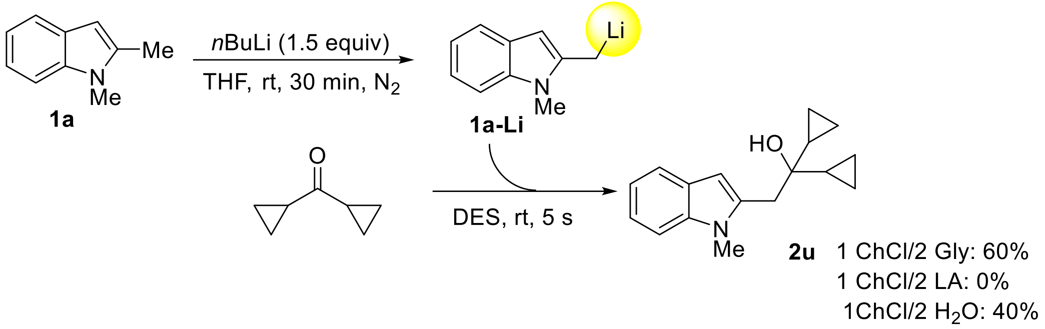

To a stirred solution of 1,2-dimethylindole (**1a**) (145 mg, 1 mmol) in anhydrous THF (2 mL, 0.5 M) was added *n*BuLi (0.6 mL, 1.5 mmol, 2.5 M in hexane) at rt and stirred for 30 min. In a round-bottom flask, dicycloproyl ketone (220 mg, 2 mmol) and the corresponding *DES* (1 g) were added under air. Then, the preformed organolithium was added to the above mixture under vigorous stirring (1000 rpm) at rt, and the reaction mixture was stirred for 2–3 s. The reaction was quenched with water and extracted with EtOAc (3 × 5mL). The combined organic layers were dried over anhydrous Na_2SO_4, filtered and concentrated under reduced pressure, obtaining alcohol **2u,** which was isolated by flash column chromatography (hexane/EtOAc, 6/1) as an orange oil. Spectroscopy data are reported above. Yields: 1 *ChCl*2 *Gly*: 60%; 1 *ChCl*2 *LA*: 0%; 1 *ChCl/*2 *H_2O:* 40%.

Synthesis of selected indole derivatives 2 in Deep Eutectic Solvents

General procedure IX

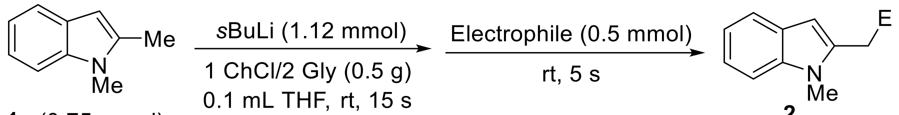

1a (0.75 mmol) **2**

Reactions were performed under air and at room temperature. In a round-bottom flask, 1,2-dimethylindol (**1a**) (109 mg, 0.75 mmol) was dissolved in the DES 1 *ChCl*/2 *Gly* (0.5 g) and THF (0.1 mL) under air, followed by the rapid addition of *s*BuLi (0.8 mL, 1.125 mmol, 1.4 M in cyclohexane) under vigorous stirring (1000 rpm) at rt, which was kept under vigorous stirring for 15 s. Then, the corresponding electrophile (0.5 mmol) was added and stirred for an additional 5 s. The reaction was quenched with water and extracted with EtOAc (3 × 5 mL). The combined organic layers were dried over anhydrous Na_2SO_4, filtered and concentrated under reduced pressure. The residue was purified by flash column chromatography on silica gel using mixtures of hexane/EtOAc as the eluent to afford the corresponding indole derivatives **2**.

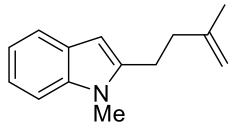

1-Methyl-2-(3-methylbut-3-en-1-yl)-1H-indole (2f):[S7] General procedure IX was followed using 3-bromo-2-methylprop-1-ene (73 mg, 0.5 mmol) as electrophile obtaining **2f**, which was isolated by flash column chromatography (hexane/EtOAc, 10/1) as a yellow oil (54 mg, 54%). Spectroscopic data have been reported above.

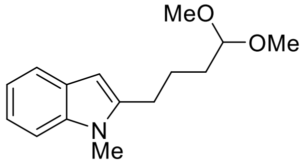

2-(4,4-Dimethoxybutyl)-1-methyl-1H-indole (2j): General procedure IX was followed using 3-bromo-1,1-dimethoxypropane (99 mg, 0.5 mmol) as electrophile obtaining **2j**, which was isolated by flash column chromatography (hexane/EtOAc, 4/1) as an orange oil (80 mg, 65%). Spectroscopic data have been reported above.

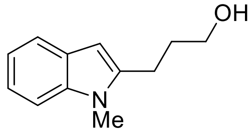

3-(1-Methyl-1H-indol-2-yl)propan-1-ol (2k): General procedure IX was followed using oxirane (0.21 mL, 0.5 mmol, 2.5 M in ether) as electrophile obtaining **2k**, which was isolated by flash column chromatography (hexane/EtOAc, 2/1) as a yellow oil (58 mg, 61%). Spectroscopic data have been reported above.

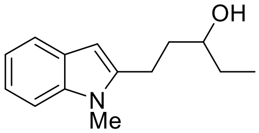

1-(1-Methyl-1H-indol-2-yl)pentan-3-ol (2m): General procedure IX was followed using 2-ethyloxirane (39 mg, 0.5 mmol) as electrophile obtaining **2m**, which was isolated by flash column chromatography (hexane/EtOAc, 3/1) as a pink solid (73 mg, 72%). Spectroscopic data have been reported above.

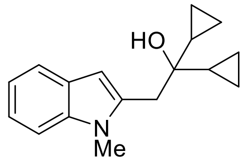

1,1-Dicyclopropyl-2-(1-methyl-1H-indol-2-yl)ethan-1-ol (2u): General procedure IX was followed using dicyclopropyl ketone (60 mg, 0.5 mmol) as electrophile obtaining **2u**, which was isolated by flash column chromatography (hexane/EtOAc, 6/1) as an orange oil (89 mg, 70%). Spectroscopic data have been reported above.

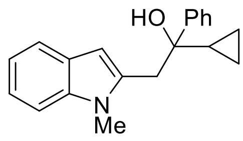

1-Cyclopropyl-2-(1-methyl-1H-indol-2-yl)-1-phenylethan-1-ol (2v): General procedure IX was followed using cyclopropyl phenyl ketone (80 mg, 0.5 mmol) as electrophile obtaining **2v**, which was isolated by flash column chromatography (hexane/EtOAc, 5/1) as a yellow oil (87 mg, 60%). Spectroscopic data have been reported above.

Synthesis of 2-methyl-4-(1-methyl-1*H*-indol-2-yl)butan-1-ol (11)

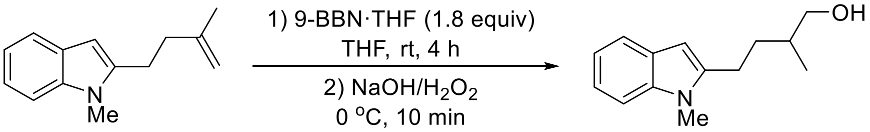

2f **11** (58%)

To a stirred solution of 1-methyl-2-(3-methylbut-3-en-1-yl)-1*H*-indole (**2f**) (438 mg, 2.2 mmol) in anhydrous THF (4 mL, 0.5 M) was added 9-BBN (7.9 mL, 3.96 mmol, 0.5 M in THF) and stirred at rt for 4 h. Then, a 30% aq solution of H_2O_2 (0.5 mL, 2 mmol) and 30% aq solution of NaOH (0.6 mL, 2 mmol) were added at 0 ºC and stirred 10 min at the same temperature. The reaction was quenched with aq $NaHCO_3$ (sat.) and extracted with EtOAc (3 × 5 mL). The combined organic layers were dried over anhydrous Na_2SO_4, filtered and concentrated under reduced pressure. The residue was purified by flash column chromatography on silica gel using a 2/1 mixture of hexane/EtOAc as the eluent to afford **11** as an orange oil (227 mg, 58%).

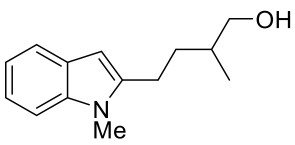

2-Methyl-4-(1-methyl-1H-indol-2-yl)butan-1-ol (11): R_f = 0.25 (hexane/EtOAc, 2/1). **^{1}H NMR** (300 MHz, $CDCl_3$): δ (ppm): 7.59 (d, *J* = 7.7 Hz, 1H), 7.31 (tap, *J* = 7.0 Hz, 1H), 7.21 (tap, *J* = 7.5 Hz, 1H), 7.17–7.08 (m, 1H), 6.32 (s, 1H), 3.71 (s, 3H), 3.59-3.56 (m, 2H), 2.87-2.78 (m, 2H), 2.00–1.88 (m, 1H), 1.84-1.78 (m, 1H), 1.67–1.53 (m, 2H), 1.07 (d, *J* = 6.7 Hz, 3H). **^{13}C{^{1}H} NMR** (75.4 MHz, $CDCl_3$): δ (ppm): 141.2, 137.4, 127.9, 120.6, 119.8, 119.3, 108.8, 98.7, 68.1, 35.5, 32.0, 29.5, 24.4, 16.6. **HRMS** (ESI+) could not be recorded.

Synthesis of tetrahydrocyclopenta[*b*]indole 12

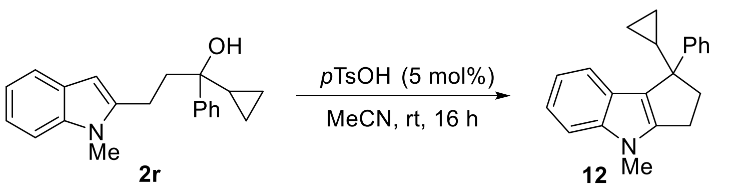

To a stirred solution of 1-cyclopropyl-3-(1-methyl-1*H*-2-yl)-1-phenylpropan-1-ol (**2q**) (152 mg, 0.5 mmol) in MeCN (1 mL, 0.5 M) was added *p*TsOH (9.5 mg, 0.05 mmol) and stirred at rt for 16 h. Then, the reaction was quenched with aq NaOH (1 M) and extracted with EtOAc (3 × 5 mL). The combined organic layers were dried over anhydrous Na_2SO_4, filtered and concentrated under reduced pressure. The residue was obtained pure without further purification as a red oil (126 mg, 88%).

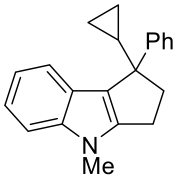

1-Cyclopropyl-4-methyl-1-phenyl-1,2,3,4-tetrahydrocyclopenta[b]indole (12): **^{1}H NMR** (500 MHz, $CDCl_3$): δ (ppm): 7.57–7.50 (m, 2H), 7.32–7.22 (m, 4H), 7.22–7.15 (m, 1H), 7.12 (ddd, *J* = 8.1, 7.1, 1.0 Hz, 1H), 6.99 (ddd, *J* = 8.1, 7.1, 1.0 Hz, 1H), 3.71 (s, 3H), 2.98–2.90 (m, 1H), 2.90–2.80 (m, 1H), 2.74 (ddd, *J* = 12.8, 8.1, 3.8 Hz, 1H), 2.71–2.63 (m, 1H), 1.61 (tt, *J* = 8.3, 5.5 Hz, 1H), 0.51–0.38 (m, 2H), 0.38–0.29 (m, 1H), 0.02–-0.06 (m, 1H). **^{13}C{^{1}H} NMR** (126 MHz, $CDCl_3$): δ (ppm): 149.8, 146.9, 141.5, 128.1, 127.3, 125.8, 124.9, 120.1, 120.0, 119.4, 119.2, 109.6, 53.2, 46.0, 30.9, 24.4, 21.2, 2.5, 2.2. **HRMS** (ESI+) calcd for $C_{21}H_{22}N^+$ [M+H]$^+$ 288.1747, found 288.1748.

Synthesis of oxepino[4,3-*b*]indole 13

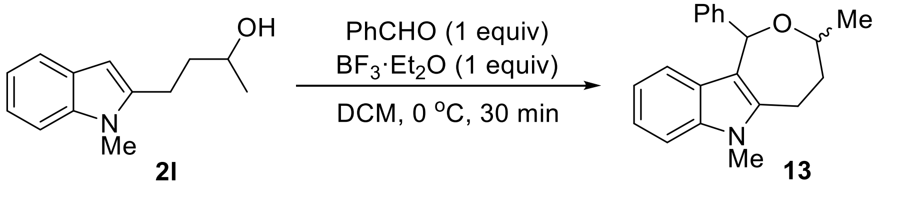

To a stirred solution of 4-(1-methyl-1*H*-indol-2-yl)butan-2-ol (**2l**) (102 mg, 0.5 mmol) in anhydrous CH_2Cl_2 (5 mL, 0.1 M) were added benzaldehyde (53 mg, 0.5 mmol) and $BF_3{\cdot}Et_2O$ (62 µL, 0.5 mmol) at 0 ºC. The resulting mixture was stirred at the same temperature for 30 min (monitored by GC-MS). Then, the reaction was quenched with aq $NaHCO_3$ (sat) and extracted with EtOAc (3 × 5 mL). The combined organic layers were dried over anhydrous Na_2SO_4, filtered and concentrated under reduced pressure, obtaining **14** as a c.a. 1/1 mixture of diastereoisomers. The residue was purified by flash column chromatography on silica gel using a 5/1 mixture of hexane/EtOAc as the eluent to afford **13** as a c.a. 2/1 mixture of diastereoisomers.

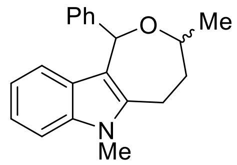

3,6-Dimethyl-1phenyl-3,4,5,6-tetrahydro-1H-oxepino[4,3-b]indole (13): purple oil (87 mg, 60%). R_f = 0.30 (hexane/EtOAc, 5/1). **^{1}H NMR** (500 MHz, $CDCl_3$): δ (ppm): 7.55–7.48 (m, 1H, major diast), 7.42–7.28 (m, 8H, both diast), 7.27-7.25 (m, 1H, minor diast), 7.24–7.16 (m, 1H, major diast), 7.14-7.11 (m, 2H, both diast), 7.06–6.98 (m, 1H, major diast), 6.93-6.85 (s, 1H, minor diast), 6.68–6.64 (m, 1H, minor diast), 6.44 (s, 1H, major diast), 5.95 (s, 1H, minor diast), 4.18–4.11 (m, 1H, minor diast), 3.99–3.91 (m, 1H, major diast), 3.77 (s, 3H, major diast), 3.76 (s, 3H, minor diast), 3.46–3.34 (m, 1H, minor diast), 3.12 (ddd, *J* = 16.3, 5.9, 2.9 Hz, 1H, major diastr), 2.97–2.81 (m, 2H, both diast), 2.15 (s, 1H, minor diast), 2.04–1.85 (m, 4H, both diast), 1.85-1.83 (m, 1H, major diast) 1.38 (dd, *J* = 6.3, 4.0 Hz, 3H, minor diast), 1.29 (dd, *J* = 6.4, 4.1 Hz, 3H, major diastr). **^{13}C{^{1}H} NMR** (126 MHz, $CDCl_3$): δ (ppm) for both diast: 142.7, 141.1, 138.2, 137.6, 136.7, 136.3, 128.8, 128.5, 128.3, 128.1, 127.8, 127.5, 127.10, 126.08, 120.9, 120.6, 119.3, 119.1, 118.8, 118.2, 113.3, 113.1, 109.0, 108.9, 80.5, 74.53, 74.51, 70.8, 35.2, 34.6, 29.8, 29.5, 23.9, 23.2, 22.8, 21.1. **HRMS** (ESI+) calcd for $C_{20}H_{22}NO^+$ $[M+H]^+$ 292.1696, found 292.1688.

Synthesis of of 2-homopropargylindoles 14

General procedure X

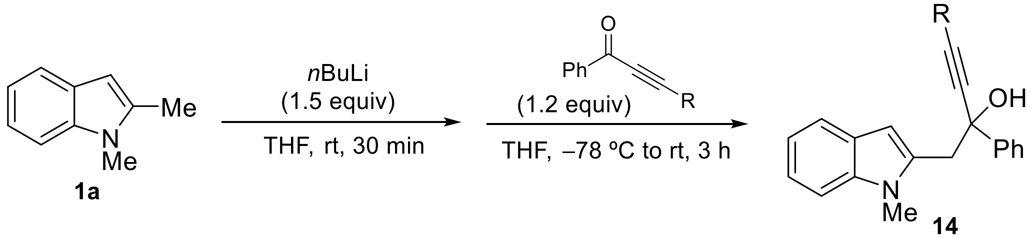

To a stirred solution of 1,2-dimethylindole (**1a**) (145 mg, 1 mmol) in anhydrous THF (2 mL, 0.5 M) was added *n*BuLi (0.6 mL, 1.5 mmol, 2.5 M in hexane) at rt and stirred for 30 min. Then, the corresponding alkynone (1.2 mmol) in THF (2 mL) was added dropwise at −78 ºC and the resulting solution was stirred for 20 min at the same temperature and then at rt until completion (monitored by CG/MS). The resulting mixture was quenched with aq NH_4Cl (5 mL) and extracted with Et_2O (3 × 5 mL). The combined organic layers were dried over anhydrous Na_2SO_4, filtered and concentrated under reduced pressure. The residue was purified by flash column chromatography on silica gel using mixtures of hexane/EtOAc as the eluent to afford the corresponding 2-homopropargylindoles **14**.

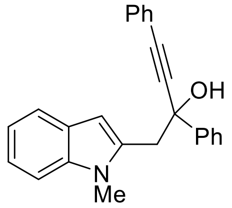

1-(1-Methyl-1H-indol-2-yl)-2,4-diphenylbut-3-yn-2-ol (14a): General procedure X was followed using 1,3-diphenylprop-2-yn-1-one (247 mg, 1.2 mmol) as alkynone obtaining **14a**, which was isolated by flash column chromatography (hexane/EtOAc, 5/1) as a brown oil (274 mg, 78%). R_f = 0.33 (hexane/EtOAc, 5/1). **^{1}H NMR** (300 MHz, $CDCl_3$): δ (ppm): 7.83–7.75 (m, 2H), 7.67 (ad, *J* = 7.7 Hz, 1H), 7.50–7.32 (m, 9H), 7.31–7.25 (m, 1H), 7.22–7.15 (m, 1H), 6.58 (s, 1H), 3.64 (s, 3H), 3.60–3.46 (m, 2H), 3.05 (bs, 1H). **^{13}C{^{1}H} NMR** (75.4 MHz, $CDCl_3$): δ (ppm): 144.0, 137.7, 134.8, 131.8, 128.8, 128.5, 128.4, 128.1, 127.7, 125.6, 122.4, 121.3, 120.3, 119.6, 109.4, 103.1, 91.3, 86.7, 72.7, 43.0, 29.9. **HRMS** (ESI+) calcd for $C_{25}H_{21}NO^+$ $[M+H]^+$ 352.1696, found 352.1705.

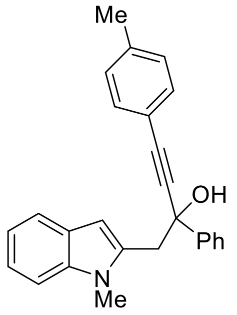

1-(1-Methyl-1H-indol-2-yl)-2-phenyl-4-(p-tolyl)but-3-yn-2-ol (14b): General procedure X was followed using 1-phenyl-3-(*p*-tolyl)prop-2-yn-1-one (238 mg, 1.2 mmol) as alkynone obtaining **14b**, which was isolated by flash column chromatography (hexane/EtOAc, 5/1) as a brown oil (256 mg, 70%). R_f = 0.35 (hexane/EtOAc, 5/1). **^{1}H NMR** (300 MHz, $CDCl_3$): δ (ppm): 7.84–7.75 (m, 2H), 7.68 (ad, J = 7.7 Hz, 1H), 7.49–7.28 (m, 7H), 7.19 (ad, J = 7.3 Hz, 3H), 6.58 (s, 1H), 3.64 (s, 3H), 3.59–3.45 (m, 2H), 3.02 (bs, 1H), 2.43 (s, 3H). **^{13}C{^{1}H} NMR** (75.4 MHz, $CDCl_3$): δ (ppm): 144.1, 138.3, 137.8, 134.9, 131.7, 129.2, 128.4, 128.1, 127.7, 125.6, 121.3, 120.3, 119.6, 119.3, 109.4, 103.1, 90.6, 86.8, 72.8, 43.1, 30.0, 21.6. **HRMS** (ESI+) calcd for $C_{26}H_{23}NO^+$ [M+H]$^+$ 366.1852, found 366.1860.

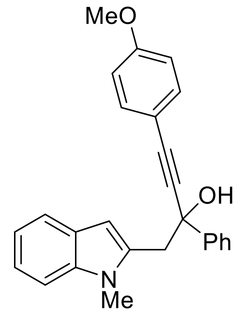

4-(4-Methoxyphenyl)-1-(1-methyl-1H-indol-2-yl)-2-phenylbut-3-yn-2-ol (14c): General procedure X was followed using 3-(4-methoxyphenyl)-1-phenylprop-2-yn-1-one (284 mg, 1.2 mmol) as alkynone obtaining **14c**, which was isolated by flash column chromatography (hexane/EtOAc, 3/1) as a brown oil (259 mg, 68%). R_f = 0.29 (hexane/EtOAc, 3/1). **^{1}H NMR** (300 MHz, $CDCl_3$): δ (ppm): 7.80–7.74 (m, 2H), 7.67 (ad, J = 7.8 Hz, 1H), 7.48–7.37 (m, 5H), 7.35–7.26 (m, 2H), 7.22–7.14 (m, 1H), 6.89 (ad, J = 8.6 Hz, 2H), 6.56 (s, 1H), 3.85 (s, 3H), 3.63 (s, 1H), 3.58–3.45 (m, 2H), 3.04 (bs, 1H). **^{13}C{^{1}H} NMR** (75.4 MHz, $CDCl_3$): δ (ppm): 160.0, 144.2, 137.8, 134.9, 133.3, 128.4, 128.0, 127.8, 125.6, 121.3, 120.3, 119.6, 114.5, 114.1, 109.4, 103.1, 89.9, 86.7, 72.8, 55.4, 43.1, 30.0. **HRMS** (ESI+) calcd for $C_{26}H_{23}NO_2^+$ [M+H]$^+$ 382.1802, found 382.1809.

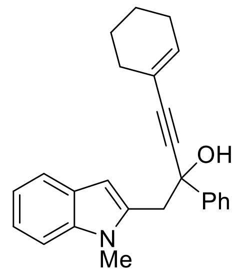

4-(Cyclohex-1-en-1-yl)-1-(1-methyl-1H-indol-2-yl)-2-phenylbut-3-yn-2-ol (14d): General procedure X was followed using 3-(cyclohex-1-en-1-yl)-1-phenylprop-2-yn-1-one (252 mg, 1.2 mmol) as alkynone obtaining **14d**, which was isolated by flash column chromatography (hexane/EtOAc, 5/1) as a brown oil (213 mg, 60%). R_f = 0.36 (hexane/EtOAc, 5/1). **^{1}H NMR** (300 MHz, $CDCl_3$): δ (ppm): 7.72–7.61 (m, 3H), 7.44–7.23 (m, 6H), 7.20–7.12 (m, 1H), 6.50 (s, 1H), 6.24–6.06 (m, 1H), 3.60 (s, 3H), 3.50–3.35 (m, 2H), 2.89 (bs, 1H), 2.23–2.07 (m, 4H), 1.75–1.59 (m, 4H). **^{13}C{^{1}H} NMR** (75.4 MHz, $CDCl_3$): δ (ppm): 144.3, 137.7, 135.8, 135.0, 128.3, 127.9, 127.7, 125.6, 121.2, 120.2, 120.1, 119.5, 109.4, 103.0, 88.5, 72.7, 43.0, 29.9, 29.1, 25.7, 22.3, 21.5. **HRMS** (ESI+) calcd for $C_{26}H_{25}NO^+$ [M+H]$^+$ 356.2009, found 356.2017.

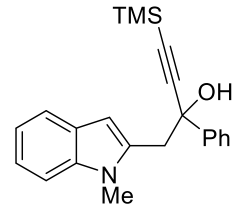

1-(1-Methyl-1H-indol-2-yl)-2-phenyl-4-(trimethylsilyl)but-3-yn-2-ol (14e): General procedure X was followed using 1,2-dimethilindole (290 mg, 2 mmol) and 1-phenyl-3-(trimethylsilyl)prop-2-yn-1-one (486 mg, 2.4 mmol) as alkynone obtaining **14e**, which was isolated by flash column chromatography (hexane/EtOAc, 5/1) as a brown solid (493 mg, 75%). M.p. = 89–91 ºC. R_f = 0.32 (hexane/EtOAc, 5/1). **^{1}H NMR** (300 MHz, $CDCl_3$): δ (ppm): 7.76–7.62 (m, 3H), 7.48–7.37 (m, 3H), 7.36–7.25 (m, 2H), 7.21–7.12 (m, 1H), 6.52 (s, 1H), 3.59 (s, 3H), 3.48–3.36 (m, 2H), 2.91 (bs, 1H), 0.26 (s, 9H). **^{13}C{^{1}H} NMR** (75.4 MHz, $CDCl_3$): δ (ppm): 143.7, 137.7, 134.6, 128.4, 128.0, 127.7, 125.5, 121.2, 120.2, 119.5, 109.4, 107.4, 103.1, 91.4, 72.6, 42.9, 30.0, -0.1. **HRMS** (ESI+) calcd for $C_{22}H_{25}NOSi^+$ [M+H]$^+$ 348.1778, found 348.1787.

Synthesis of 1-(1-methyl-1*H*-indol-2-yl)-2-phenylbut-3-yn-2-ol (14f)

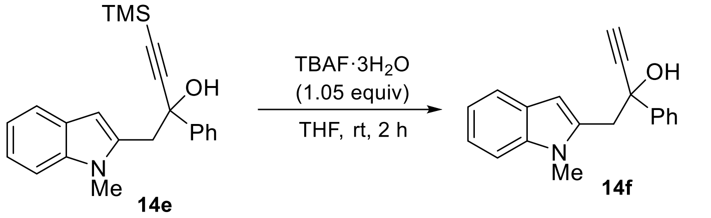

To a stirred solution of 1-(1-methyl-1H-indol-2-yl)-2-phenyl-4-(trimethylsilyl)but-3-yn-2-ol (**14e**) (348 mg, 1 mmol) in THF (5 mL, 0.2 M) was added TBAF·3H_2O (369 mg, 1.05 mmol) and the resulting solution was stirred at rt for 2 h. Then, the resulting mixture was quenched with water (2 mL) and extracted with EtOAc (3 × 5 mL). The combined organic layers were dried over anhydrous Na_2SO_4, filtered and concentrated under reduced pressure. The residue was purified by flash column chromatography on silica gel using a 5/1 mixture of hexane/EtOAc as the eluent to afford **14f** as a yellow oil (245 mg, 89%).

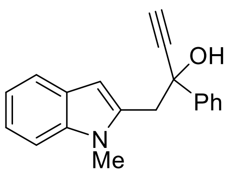

1-(1-Methyl-1H-indol-2-yl)-2-phenylbut-3-yn-2-ol (14f): R_f = 0.29 (hexane/EtOAc, 5/1). **^{1}H NMR** (300 MHz, $CDCl_3$): 7.68–7.65 (m, 2H), 7.62 (ad, *J* = 7.8 Hz, 1H), 7.42–7.36 (m, 3H), 7.31–7.29 (m, 1H), 7.25–7.21 (m, 1H), 7.15–7.12 (m, 1H), 6.49 (s, 1H), 3.57 (s, 3H), 3.48–3.36 (m, 2H), 2.91 (bs, 1H), 2.70 (s, 1H). **^{13}C{^{1}H} NMR** (75.4 MHz, $CDCl_3$): *δ* (ppm): 143.4, 137.8, 134.3, 128.5, 128.2, 127.7, 125.5, 121.4, 120.4, 119.7, 109.5, 103.1, 86.0, 74.9, 72.1, 42.7, 30.1. **HRMS** (ESI+) calcd for $C_{19}H_{17}NO^+$ $[M+H]^+$ 276.1383, found 276.1392.

Synthesis of 1-(1*H*-indol-2-yl)-2,4-diphenylbut-3-yn-2-ol (14g)

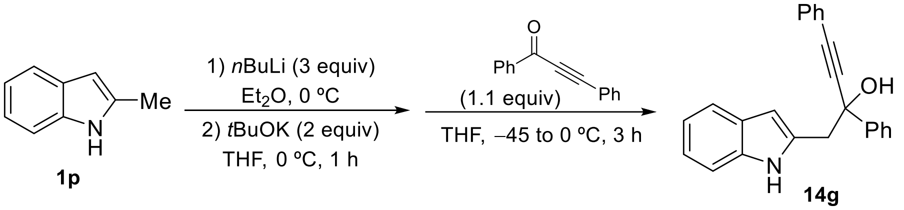

To a stirred solution of 2-methyl-1*H*-indole (**1p**) (131 mg, 1 mmol) in anhydrous Et_2O (2 mL, 0.5 M) were added *n*BuLi (1.2 mL, 3 mmol, 2.5 M in hexane) at 0 ºC and *t*BuOK (2 mL, 2 mmol, 1 M in THF). The resulting mixture was stirred at 0 ºC for 1 h. Then, 1,3-diphenylprop-2-yn-1-one (227 mg, 1.1 mmol) in THF (2 mL) was added at –45 ºC and the resulting solution was stirred at 0 ºC for 3 h (monitored by CG/MS). The mixture was quenched with aq NH_4Cl (10 mL) and extracted with Et_2O (3 × 10 mL). The combined organic layers were dried over anhydrous Na_2SO_4, filtered and concentrated under reduced pressure. The residue was purified by flash column chromatography on silica gel using a 5/1 mixture of hexane/EtOAc as the eluent to afford **14g** as a brown solid (186 mg, 55%).

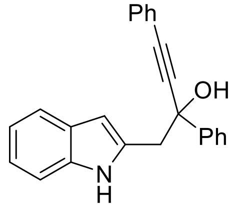

1-(1H-Indol-2-yl)-2,4-diphenylbut-3-yn-2-ol (**14g**): M.p.: 80–82 °C. R_f = 0.30 (hexane/EtOAc, 5/1). **^{1}H NMR** (300 MHz, $CDCl_3$): 8.50 (bs, 1H), 7.79–7.72 (m, 2H), 7.61 (ad, *J* = 7.7 Hz, 1H), 7.47–7.32 (m, 9H), 7.23–7.10 (m, 2H), 6.41 (as, 1H), 3.47 (s, 2H), 2.86 (bs, 1H). **^{13}C{^{1}H} NMR** (75.4 MHz, $CDCl_3$): *δ* (ppm): 143.9, 136.5, 134.0, 131.9, 128.9, 128.6, 128.5, 128.4, 128.2, 125.5, 122.2, 121.7, 120.3, 119.7, 110.8, 103.5, 91.0, 87.1, 73.2, 45.1. **HRMS** (ESI+) calcd for $C_{24}H_{19}NO^+$ $[M+H]^+$ 338.1539, found 338.1546.

Synthesis of carbazoles 15

General procedure XI

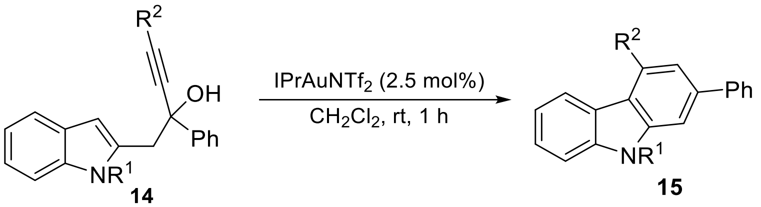

$IPrAuNTf_2$ (2.5 mol%, 11 mg) was dissolved in CH_2Cl_2 (1 mL) and the resulting solution was stirred for 5 min at rt. A solution of the corresponding alkynol **14** (0.5 mmol) in CH_2Cl_2 (2 mL, 0.25 M) was subsequently added. The reaction mixture was stirred at rt until completion monitored by TLC. The mixture was filtered through a short pad of silica gel and celite using a 5/1 mixture of hexane/EtOAc, and the solvents were removed under reduced pressure. The residue was purified by flash column chromatography on silica gel using mixtures of hexane and EtOAc as the eluent to afford the corresponding carbazoles **15**.

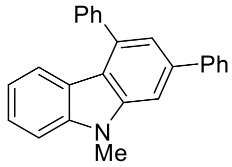

***9-Methyl-2,4-diphenyl-9H-carbazole (15a)*:**[S12] General procedure XI was followed using 1-(1-methyl-1*H*-indol-2-yl)-2,4-diphenylbut-3-yn-2-ol (**14a**) (176 mg, 0.5 mmol) obtaining **15a**, which was isolated by flash column chromatography (hexane/EtOAc, 10/1) as a yellow oil (145 mg, 87%). R_f = 0.45 (hexane/EtOAc, 10/1). **^{1}H NMR** (300 MHz, $CDCl_3$): δ (ppm): 7.85 (ad, J = 7.7 Hz, 2H), 7.78 (ad, J = 7.5 Hz, 2H), 7.68–7.43 (m, 11H), 7.13–7.04 (m, 1H), 3.97 (s, 3H). **^{13}C{^{1}H} NMR** (75.4 MHz, $CDCl_3$): δ (ppm): 142.1, 142.0, 141.8, 141.4, 139.0, 138.0, 129.4, 128.9, 128.6, 127.7, 127.3, 125.7, 122.4, 122.3, 120.5, 119.6, 118.8, 108.4, 106.0, 29.3, one carbon signal is missing due to overlapping.

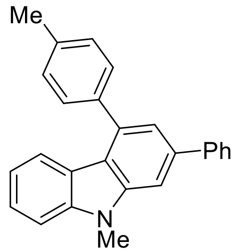

***9-Methyl-2-phenyl-4-(p-tolyl)-9H-carbazole (15b)*:**[S12] General procedure XI was followed using 1-(1-methyl-1*H*-indol-2-yl)-2-phenyl-4-(*p*-tolyl)but-3-yn-2-ol (**14b**) (183 mg, 0.5 mmol) obtaining **15b**, which was isolated by flash column chromatography (hexane/EtOAc, 10/1) as a colourless solid (146 mg, 84%). M.p.: 136–138 °C (lit. M.p.: 139–141 ºC).[S12] R_f = 0.47 (hexane/EtOAc, 10/1). **^{1}H NMR** (500 MHz, $CDCl_3$): δ (ppm): 7.87–7.81 (m, 2H), 7.70–7.63 (m, 4H), 7.58–7.54 (m, 2H), 7.49–7.40 (m, 6H), 7.13–7.03 (m, 1H), 3.97 (s, 3H), 2.57 (s, 3H). **^{13}C{^{1}H} NMR** (126 MHz, $CDCl_3$): δ (ppm): 142.1, 142.1, 141.8, 139.0, 138.4, 138.1, 137.4, 129.3, 128.9, 127.7, 127.3, 125.7, 122.5, 122.5, 120.6, 119.7, 118.8, 108.4, 105.9, 29.3, 21.5.

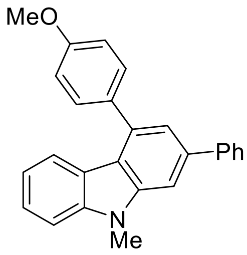

***4-(4-Methoxyphenyl)-9-methyl-2-phenyl-9H-carbazole (15c)*:**[S12] General procedure VIII was followed using 4-(4-methoxyphenyl)-1-(1-methyl-1*H*-indol-2-yl)-2-phenylbut-3-yn-2-ol (**14c**) (191 mg, 0.5 mmol) obtaining **15c**, which was isolated by flash column chromatography (hexane/EtOAc, 10/1) as a colourless solid (161 mg, 89%). M.p.: 220–222 °C (lit. M.p.: 214–216 ºC).[S12] R_f = 0.47 (hexane/EtOAc, 10/1). **^{1}H NMR** (500 MHz, $CDCl_3$): δ (ppm): 7.85–7.79 (m, 2H), 7.70–7.62 (m, 4H), 7.55–7.51 (m, 2H), 7.48–7.40 (m, 4H), 7.17–7.12 (m, 2H), 7.09–7.05 (m, 1H), 3.98 (s, 3H), 3.96 (s, 3H). **^{13}C{^{1}H} NMR** (126 MHz, $CDCl_3$): δ (ppm): 159.4, 142.1, 142.1, 141.8, 139.0, 137.7, 133.8, 130.5, 128.9, 127.7, 127.3, 125.6, 122.48, 122.47, 120.6, 119.8, 118.8, 114.0, 108.4, 105.8, 55.5, 29.3.

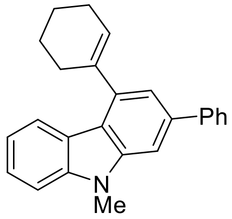

***4-(Cyclohex-1-en-1-yl)-9-methyl-2-phenyl-9H-carbazole (15d)*:** General procedure XI was followed using 4-(cyclohex-1-en-1-yl)-1-(1-methyl-1*H*-indol-2-yl)-2-phenylbut-3-yn-2-ol (**14d**) (178 mg, 0.5 mmol) obtaining **15d**, which was isolated by flash column chromatography (hexane/EtOAc, 10/1) as a yellow solid (125 mg, 74%). M.p.: 99–101 °C. R_f = 0.47 (hexane/EtOAc, 10/1). **^{1}H NMR** (500 MHz, $CDCl_3$): δ (ppm): 8.20 (ad, J = 7.8 Hz, 1H), 7.86–7.79 (m, 2H), 7.58–7.50 (m, 4H), 7.47–7.39 (m, 2H), 7.31 (ad, J = 1.5 Hz, 1H), 7.29–

7.25 (m, 1H), 6.07–6.01 (m, 1H), 3.93 (s, 3H), 2.63–2.54 (m, 2H), 2.44–2.34 (m, 2H), 2.06–1.98 (m, 2H), 1.96– 1.90 (m, 2H). **^{13}C{^{1}H} NMR** (126 MHz, $CDCl_3$): δ (ppm): 142.3, 142.0, 141.7, 140.7, 139.0, 138.3, 128.8, 127.8, 127.2, 126.2, 125.4, 122.6, 119.3, 119.0, 118.8, 108.4, 105.3, 29.8, 29.2, 25.7, 23.5, 22.5, one carbon signal is missing due to overlapping. **HRMS** (ESI+) calcd for $C_{25}H_{23}N^+$ [M+H]$^+$ 338.1903, found 338.1907.

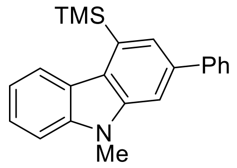

9-Methyl-2-phenyl-4-(trimethylsilyl)-9H-carbazole (15e): General procedure XI was followed using 1-(1*H*-indol-2-yl)-2-phenyl-4-(trimethylsilyl)but-3-yn-2-ol (**14e**) (167 mg, 0.5 mmol) obtaining **15e**, which was isolated by flash column chromatography (hexane/EtOAc, 10/1) as a colourless solid (148 mg, 90%). M.p.: 145–147 °C. R_f = 0.43 (hexane/EtOAc, 10/1). **^{1}H NMR** (500 MHz, $CDCl_3$): δ (ppm): 8.30 (ad, J = 8.0 Hz, 1H), 7.78 (ad, J = 8.0 Hz, 2H), 7.69 (ad, J = 6.0 Hz, 2H), 7.57–7.49 (m, 4H), 7.48–7.41 (m, 1H), 7.34–7.27 (m, 1H), 3.95 (s, 3H), 0.63 (s, 9H). **^{13}C{^{1}H} NMR** (126 MHz, $CDCl_3$): δ (ppm): 142.5, 141.8, 141.2, 138.2, 134.3, 128.9, 127.8, 127.2, 125.9, 125.5, 125.4, 123.5, 123.2, 118.8, 108.6, 108.4, 29.1, –0.2. **HRMS** (ESI+) calcd for $C_{22}H_{23}NSi^+$ [M+H]$^+$ 330.1673, found 330.1673.

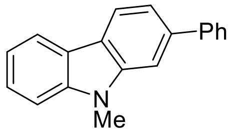

9-Methyl-2-phenyl-9H-carbazole (15f):[S13] General procedure XI was followed using 1-(1-methyl-1*H*-indol-2-yl)-2-phenylbut-3-yn-2-ol (**14f**) (138 mg, 0.5 mmol) obtaining **15f**, which was isolated by flash column chromatography (hexane/EtOAc, 10/1) as a colourless solid (113 mg, 88%). M.p.: 135–137 °C (lit. M.p.: 139–140 ºC).[S13] R_f = 0.42 (hexane/EtOAc, 10/1). **^{1}H NMR** (500 MHz, $CDCl_3$): δ (ppm): 8.19 (ad, J = 8.0 Hz, 1H), 8.17–8.15 (m, 1H), 7.82–7.77 (m, 2H), 7.63 (ad, J = 1.1 Hz, 1H), 7.56–7.51 (m, 4H), 7.46–7.40 (m, 2H), 7.33–7.27 (m, 1H), 3.92 (s, 3H). **^{13}C{^{1}H} NMR** (126 MHz, $CDCl_3$): δ (ppm): 142.4, 141.7, 141.6, 139.3, 128.9, 127.7, 127.2, 125.8, 122.7, 122.2, 120.6, 120.5, 119.1, 118.8, 108.6, 107.2, 29.2.

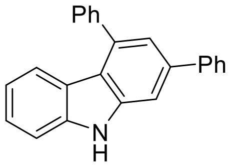

2,4-Diphenyl-9H-carbazole (15g):[S12] General procedure XI was followed using 1-(1*H*-indol-2-yl)-2,4-diphenylbut-3-yn-2-ol (**14g**) (169 mg, 0.5 mmol) obtaining **15g**, which was isolated by flash column chromatography (hexane/EtOAc, 10/1) as a brown solid (138 mg, 83%). M.p.: 175–176 °C (M.p.: 170–172 ºC).[S12] R_f = 0.30 (hexane/EtOAc, 10/1). **^{1}H NMR** (500 MHz, $CDCl_3$): δ (ppm): 8.22 (bs, 1H), 7.78–7.74 (m, 2H), 7.74–7.70 (m, 2H), 7.66 (ad, J = 1.5 Hz, 1H), 7.60–7.56 (m, 2H), 7.54–7.48 (m, 4H), 7.45 (ad, J = 8.1 Hz, 1H), 7.42–7.36 (m, 3H), 7.07–7.01 (m, 1H). **^{13}C{^{1}H} NMR** (126 MHz, $CDCl_3$): δ (ppm): 141.7, 141.3, 140.6, 140.3, 139.2, 138.1, 129.4, 128.9, 128.6, 127.8, 127.7, 127.3, 125.9, 122.9, 122.5, 121.0, 120.3, 119.4, 110.6, 108.1.

HPLC Traces

HPLC trace for 2l: Column: Chiralpak OD-H. Eluent: hex/*i*PrOH 90/10.

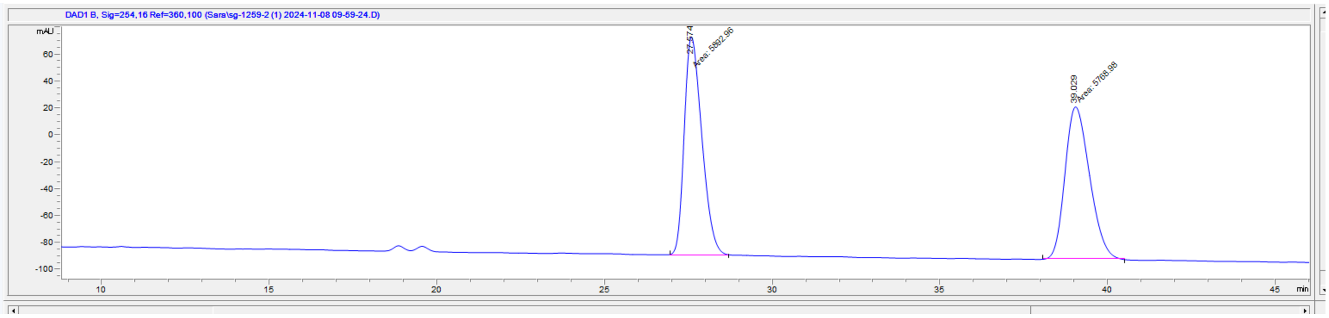

Signal 2: DAD1 B, Sig=254,16 Ref=360,100

Peak #	RetTime [min]	Type	Width [min]	Area [mAU*s]	Height [mAU]	Area %
1	27.574	MM	0.6093	5892.95508	161.18692	50.5315
2	39.029	MM	0.8523	5768.98096	112.80794	49.4685

Totals : 1.16619e4 273.99486

HPLC Traces for (*S*)-2l: Column: Chiralpak OD-H. Eluent: hex/*i*PrOH 90/10.

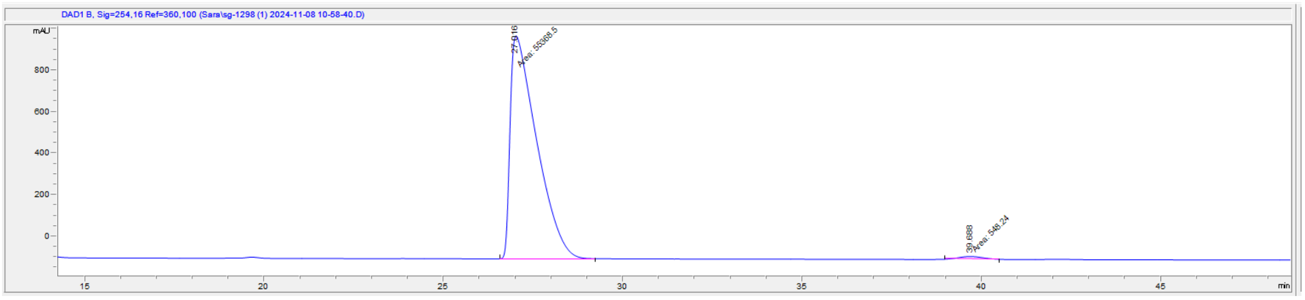

Signal 2: DAD1 B, Sig=254,16 Ref=360,100

Peak #	RetTime [min]	Type	Width [min]	Area [mAU*s]	Height [mAU]	Area %
1	27.016	MM	0.8575	5.53685e4	1076.14624	99.0195
2	39.688	MM	0.7401	548.24036	12.34600	0.9805

Totals : 5.59168e4 1088.49224

References

(*S1a*) Templ, J.; Gjata, E.; Getzner, F.; Schürch, M. Monoselective *N*-Methylation of Amides, Indoles, and Related Structures Using Quaternary Ammonium Salts as Solid Methylating Agents. *Org. Lett.* **2022**, *24*, 7315–7319.

(*S1b*) Zhang, J.; Kohlbouni, S. T.; Borhan, B. Cu-Catalyzed Oxidation of C2 and C3 Alkyl-Substituted Indole via Acyl Nitroso Reagents. *Org. Lett.* **2019**, *21*, 14–17.

(*S2a*) Comercially available CAS: 55556-49-5 (Chemieliva).

(*S2b*) Comercially available CAS: 17591-06-9 (Angene).

(*S2c*) Elsherbei, S. A.; Melen, R. L.; Pulis, A. P.; Morrill, L. C. Accessing Highly Substituted Indoles via $B(C_6F_5)_3$-Catalyzed Secondary Alkyl Group Transfer. *J. Org. Chem.* **2024**, *89*, 4244–4248.

(*S2d*) Kawada, H.; Takano, K.; Kotake, T.; Kage, M.; Hashimoto, S.; Tamiya, M.; Wakamiya, Y.; Hayashi, R.; Morita, Y. Preparation of cyclic peptides having inhibitory effect selective for KRAS but not for HRAS and NRAS. WO 2022234853, November 10, 2022.

(*S2e*) Nadres, E. T.; Lazareva, A.; Daugulis, O. Palladium-Catalyzed Indole, Pyrrole, and Furan Arylation by Aryl Chlorides. *J. Org. Chem.* **2011**, *76*, 471–483.

(*S3*) Comercially available CAS: 1971-46-6.

(*S4*) Zhu, L.; Guo, P.; Li, G.; Lan, J.; Xie, R.; You, J. Simple Copper Salt-Catalyzed N-Arylation of Nitrogen-Containing Heterocycles with Aryl and Heteroaryl Halides. *J. Org. Chem.* **2007**, *72*, 8535–8538.

(*S5*) Labadie, S. S.; Teng, E. Indol-2-yltributylstannane: A Versatile Reagent for 2-Substituted Indoles. *J. Org. Chem.* **1994**, *59*, 4250–4254.

(*S6*) Nie, G.; Tu, T.; Liao, T.; Liu, D.; Ye, W.; Ren, S. *N*-heterocyclic carbene and photocatalyst-catalyzed rapid access to indole ketones *via* radical C(sp^3)-H acylation. *Green. Chem.* **2024**, *26*, 5397–5408.

(*S7*) Han, X.; Lu, X. Novel Palladium-Catalyzed Acyloxylation/Cyclization of 2-(3'-Alkenyl)indoles. *Org. Lett.* **2009**, *11*, 2381–2384.

(*S8*) Commercially available: CAS: 866483-53-6.

(*S9*) Masutani, K.; Minowa, T.; Hagiwara, Y.; Mukaiyama, T. Cyanation of Alcohols with Diethyl Cyanophosphonate and 2,6-Dimethyl-1,4-benzoquinone by a New Type of Oxidation-Reduction Condensation. *Bull. Chem. Soc. Jpn.* **2006**, *79*, 1106–1117.

(*S10*) Duez, S.; Steib, A. K.; Knochel, P. Benzylic Arylation of 2-Methyl-5-membered Heterocycles Using TMP-Bases. *Org. Lett.* **2012**, *14*, 1951–1953.

(*S11*) Vidal, C.; García-Alvárez, J.; Hernán-Gómez, A.; Kennedy, A. R.; Hevia, E. Exploiting Deep Eutectic Solvents and Organolithium Reagent Partnerships: Chemoselective Ultrafast Addition to Imines and Quinolines Under Aerobic Ambient Temperature Conditions. *Angew. Chem. Int. Ed.,* **2016**, *55*, 16145–16148.

(*S12*) Chen, S.; Li, Y.; Ni, P.; Huang, H.; Deng, G.-J. Indole-to-Carbazole Strategy for the Synthesis of Substituted Carbazoles under Metal-Free Conditions. *Org. Lett.* **2016**, *18*, 5384–5387.

(*S13*) Kong, W.; Fu, C.; Ma, S. An efficient synthesis of carbazoles from $PtCl_2$-catalyzed cyclization of 1-(indol-2-yl)-2,3-allenols. *Chem. Comm.* **2009**, 4572–4574.

NMR SPECTRA

^{1}H-NMR (300 MHz, $CDCl_3$)

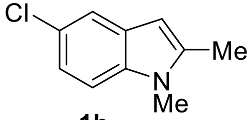

1b

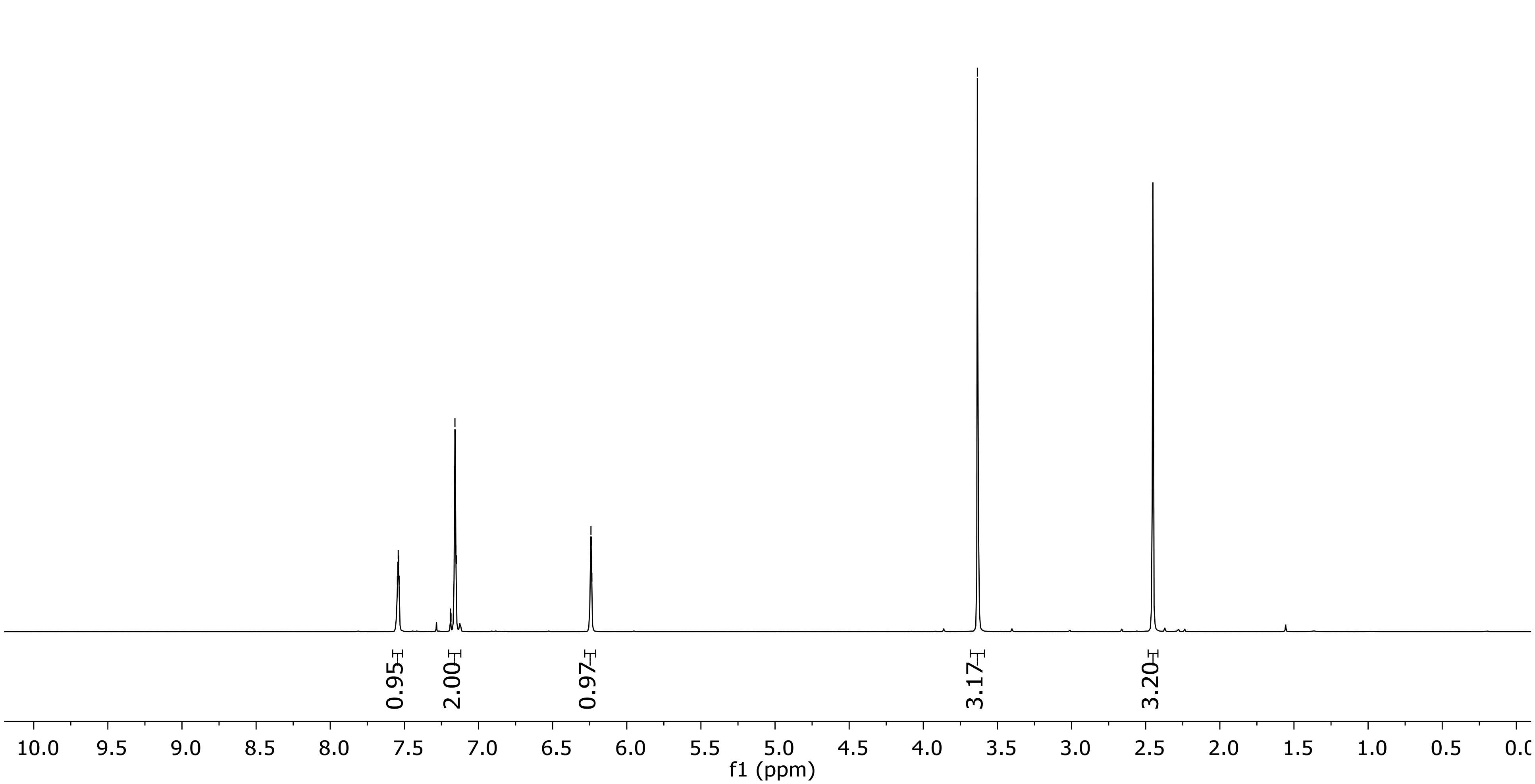

$^{13}C\{^1H\}$-NMR (75.4 MHz, $CDCl_3$)

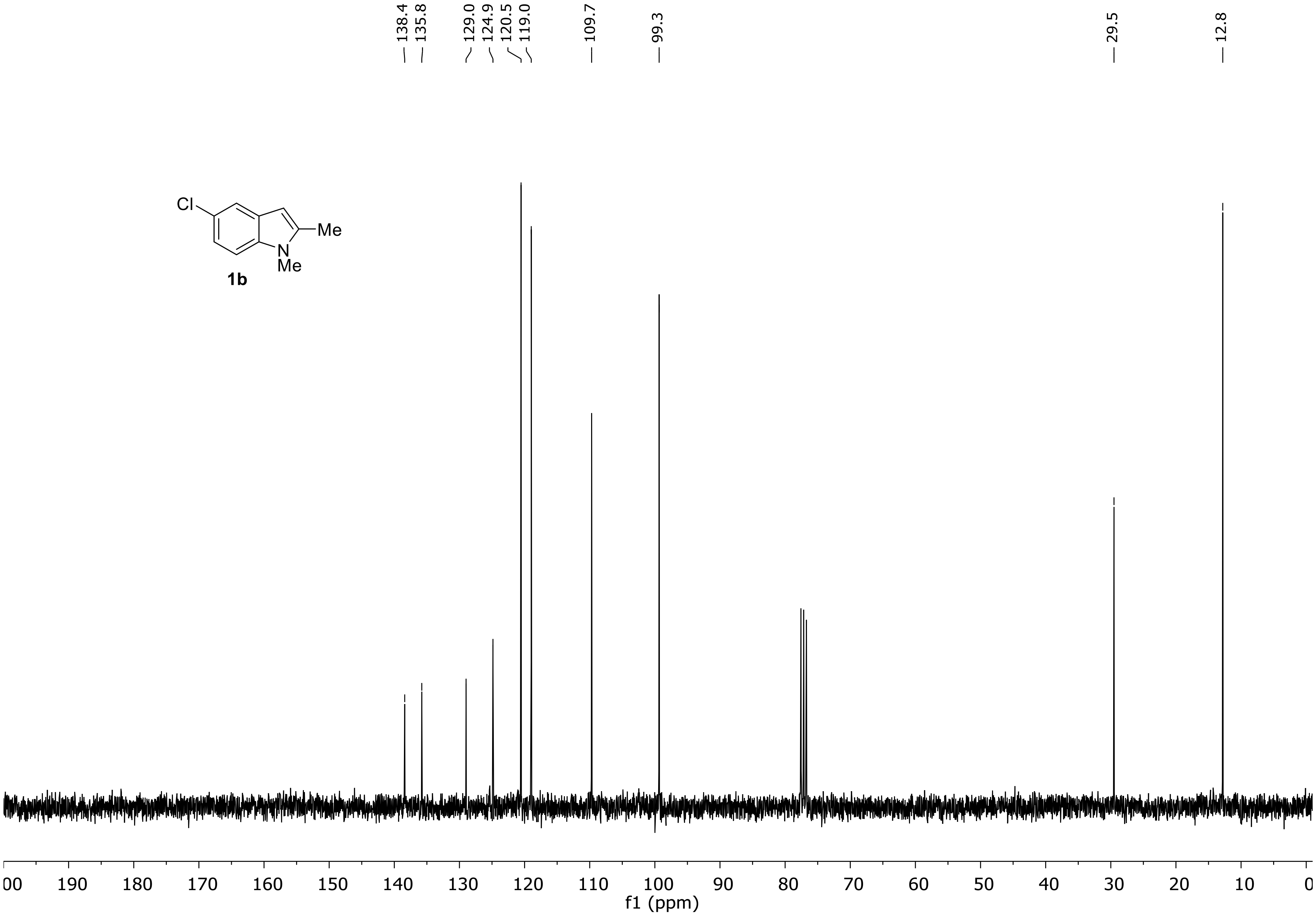

^{1}H-NMR (300 MHz, $CDCl_3$)

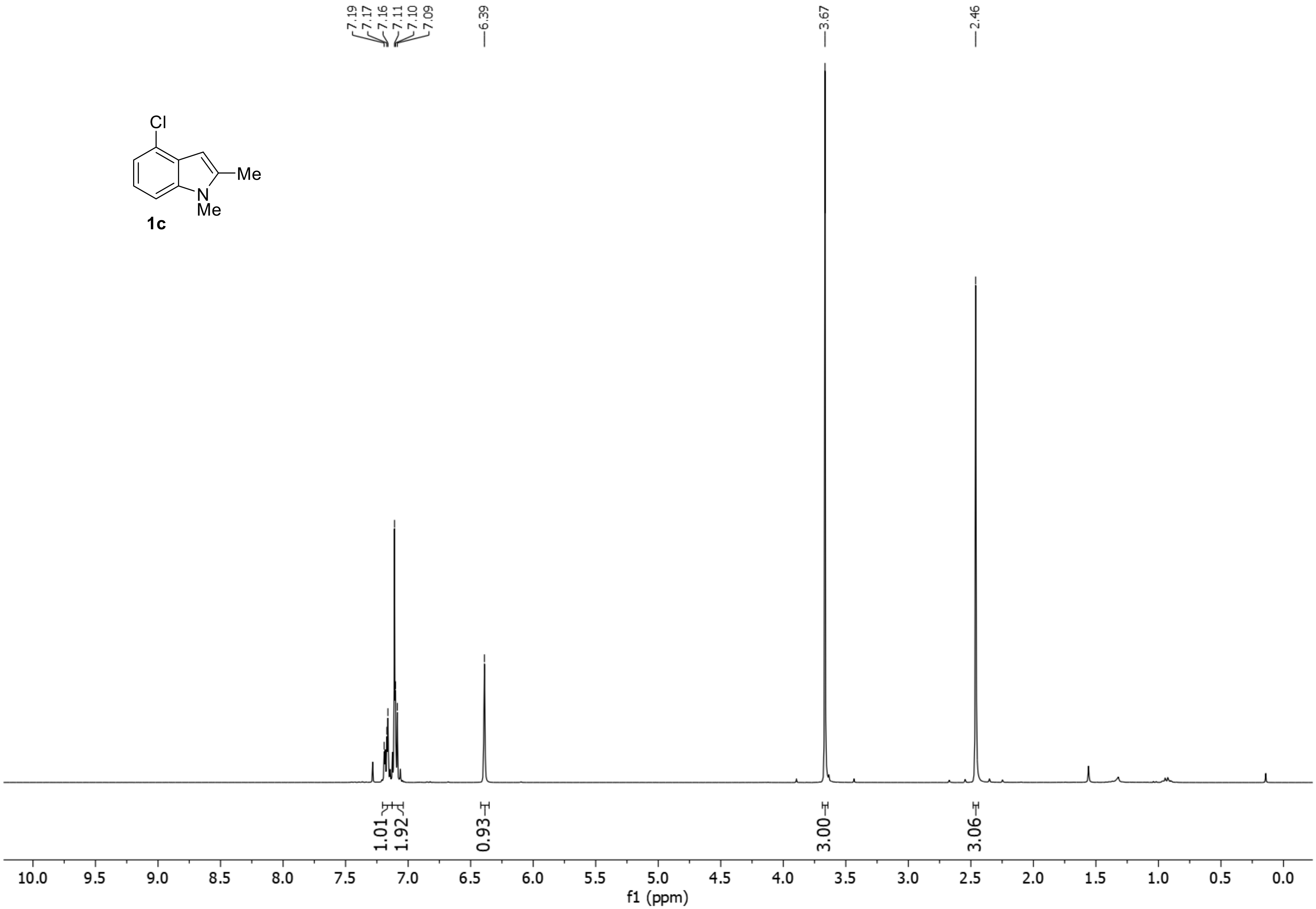

1c

$^{13}C\{^{1}H\}$-NMR (75.4 MHz, $CDCl_3$)

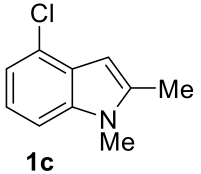

1c

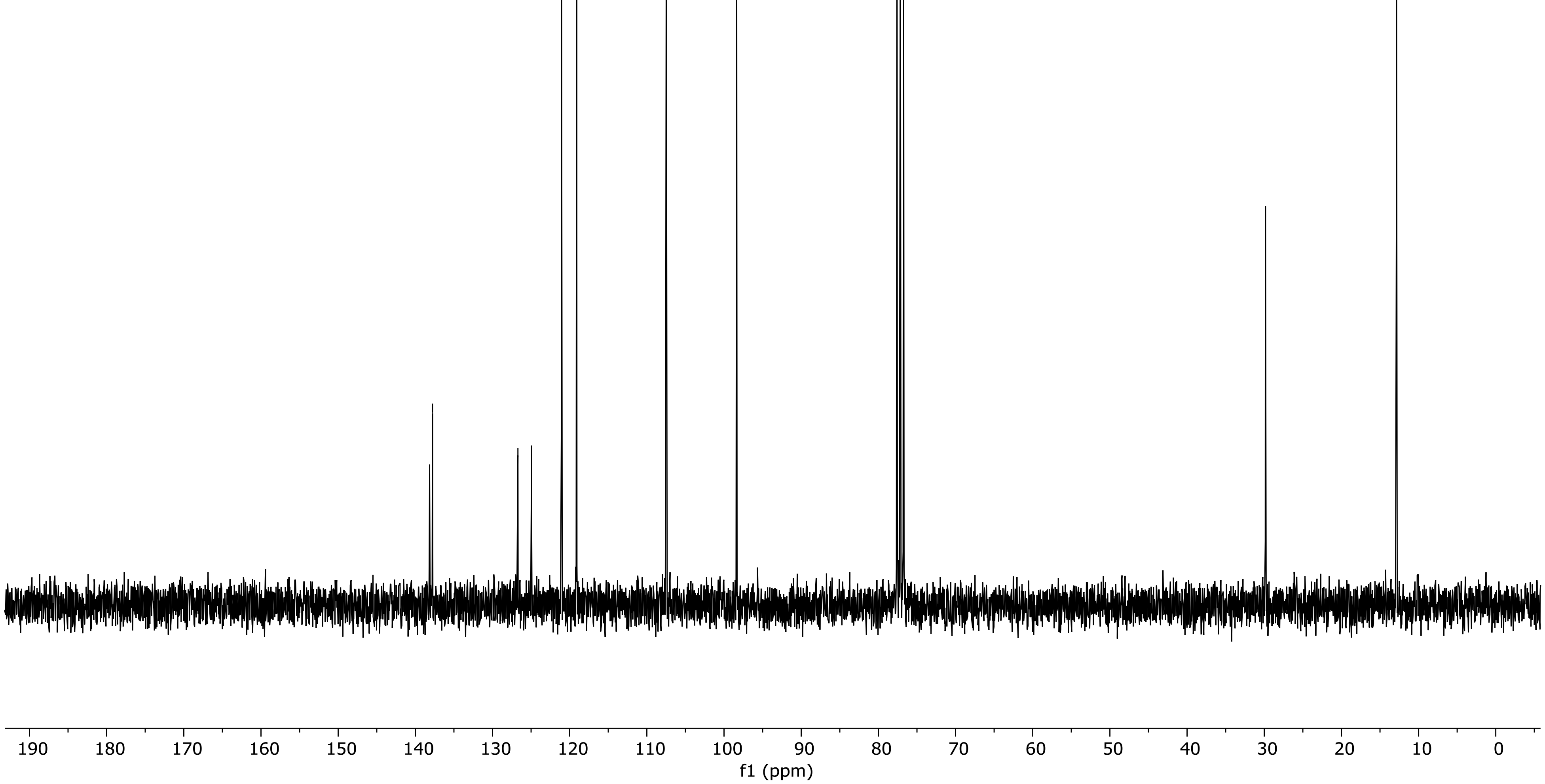

^{1}H-NMR (300 MHz, $CDCl_3$)

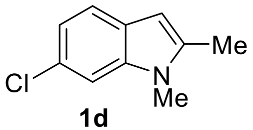

1d

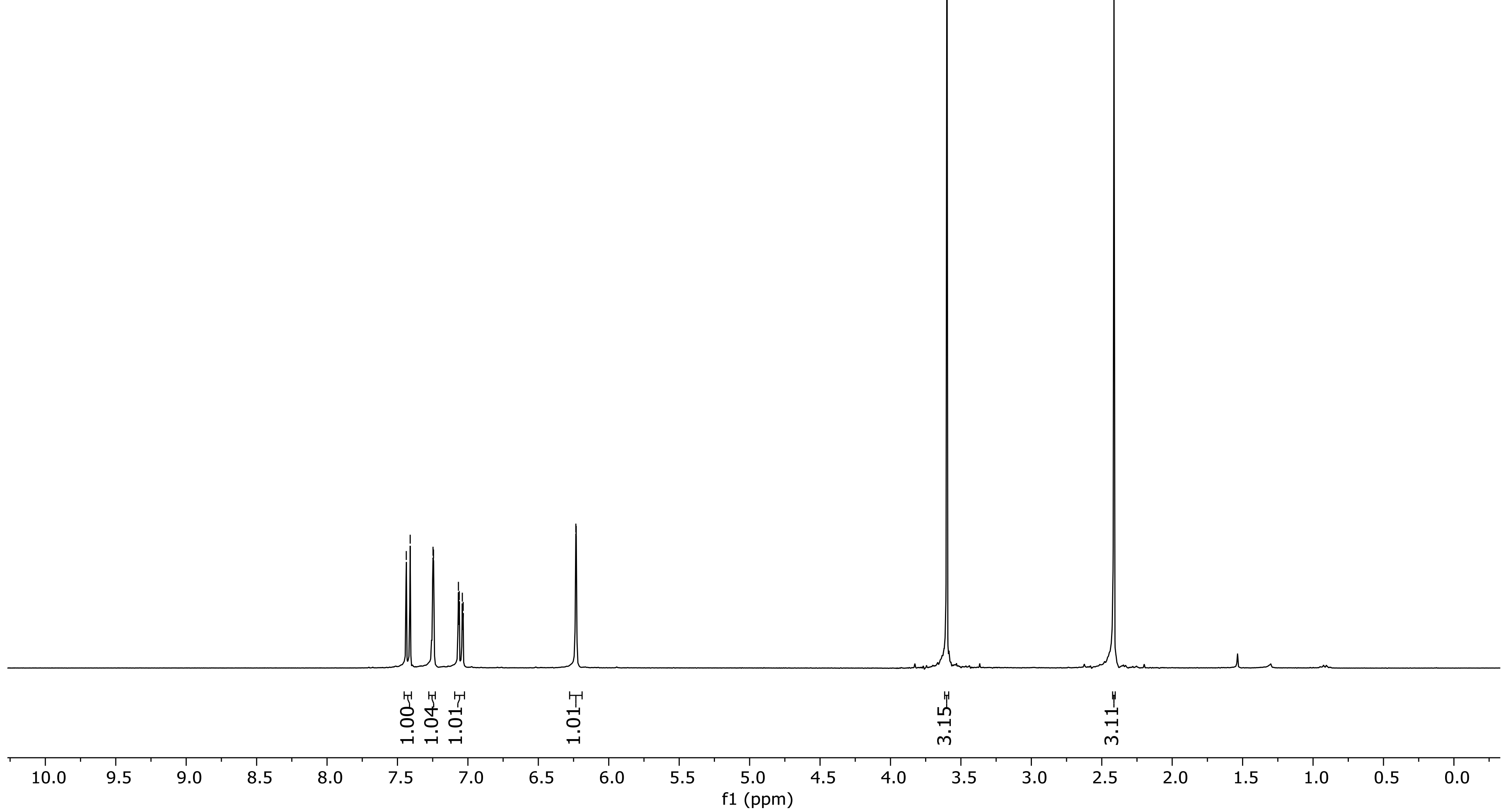

$^{13}C\{^1H\}$-NMR (75.4 MHz, $CDCl_3$)

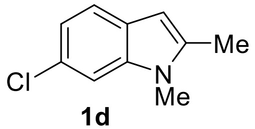

1d

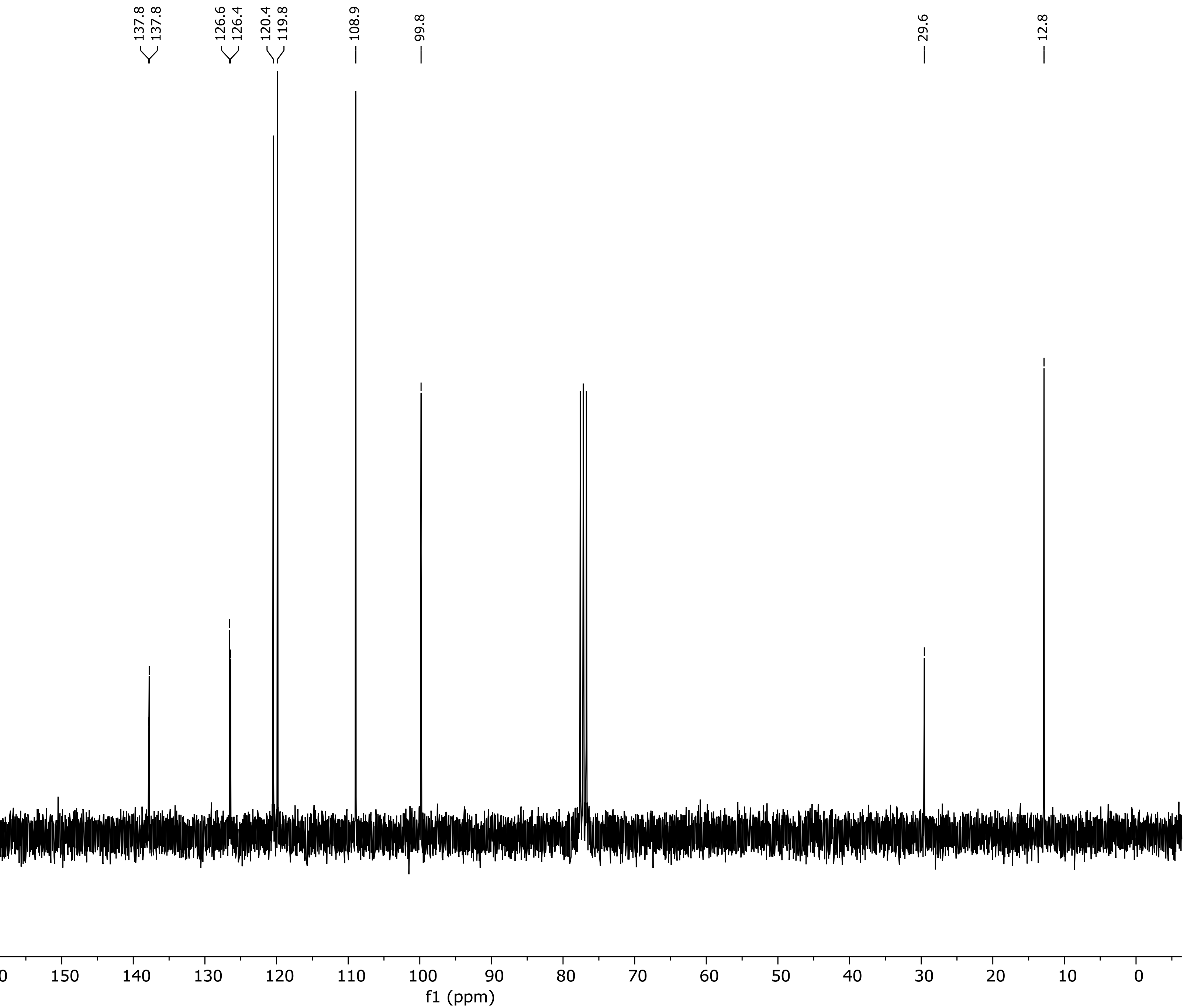

^{1}H-NMR (300 MHz, $CDCl_3$)

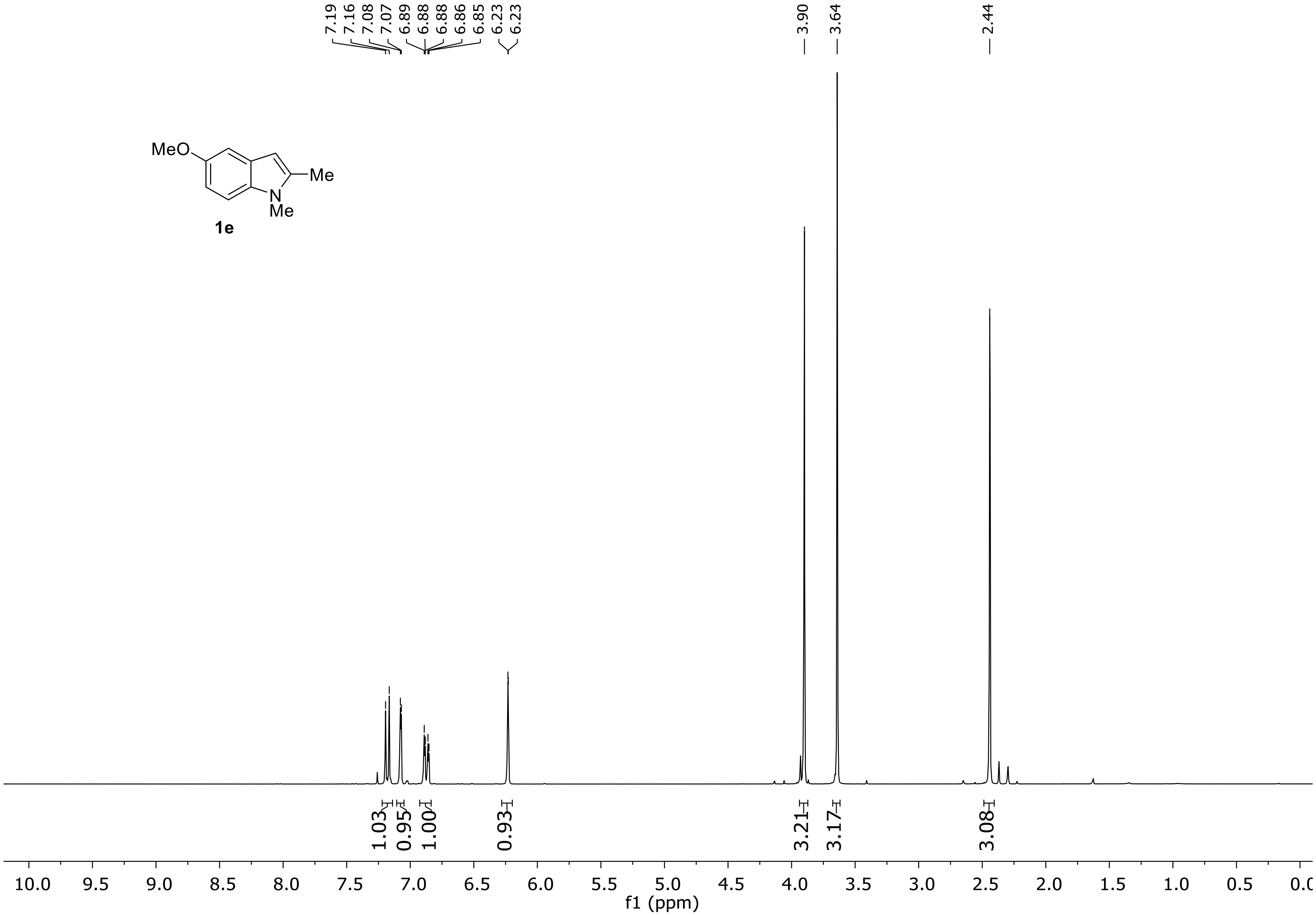

$^{13}C\{^1H\}$-NMR (75.4 MHz, $CDCl_3$)

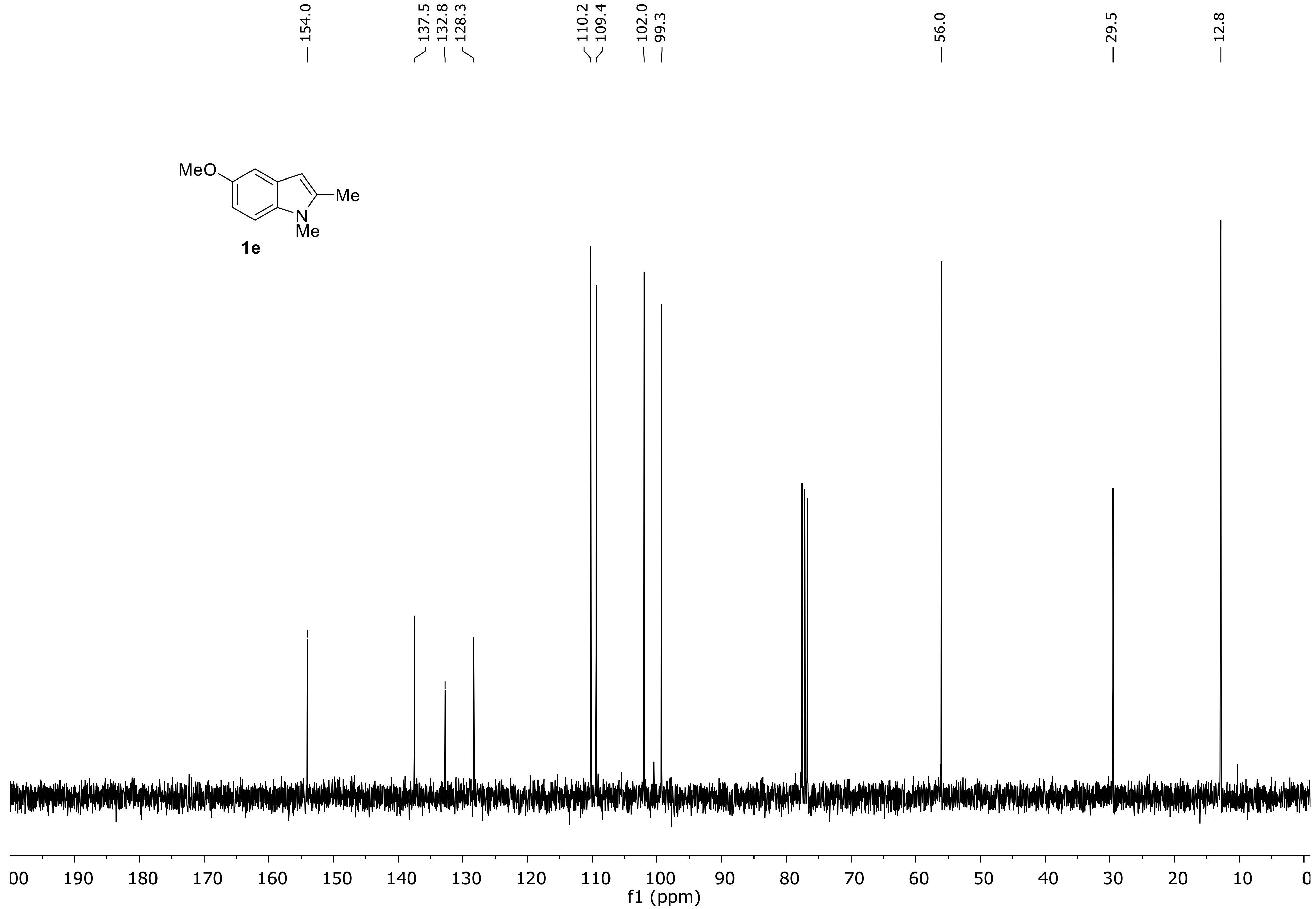

^{1}H-NMR (300 MHz, $CDCl_3$)

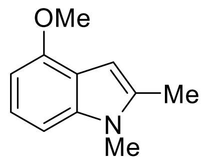

1f

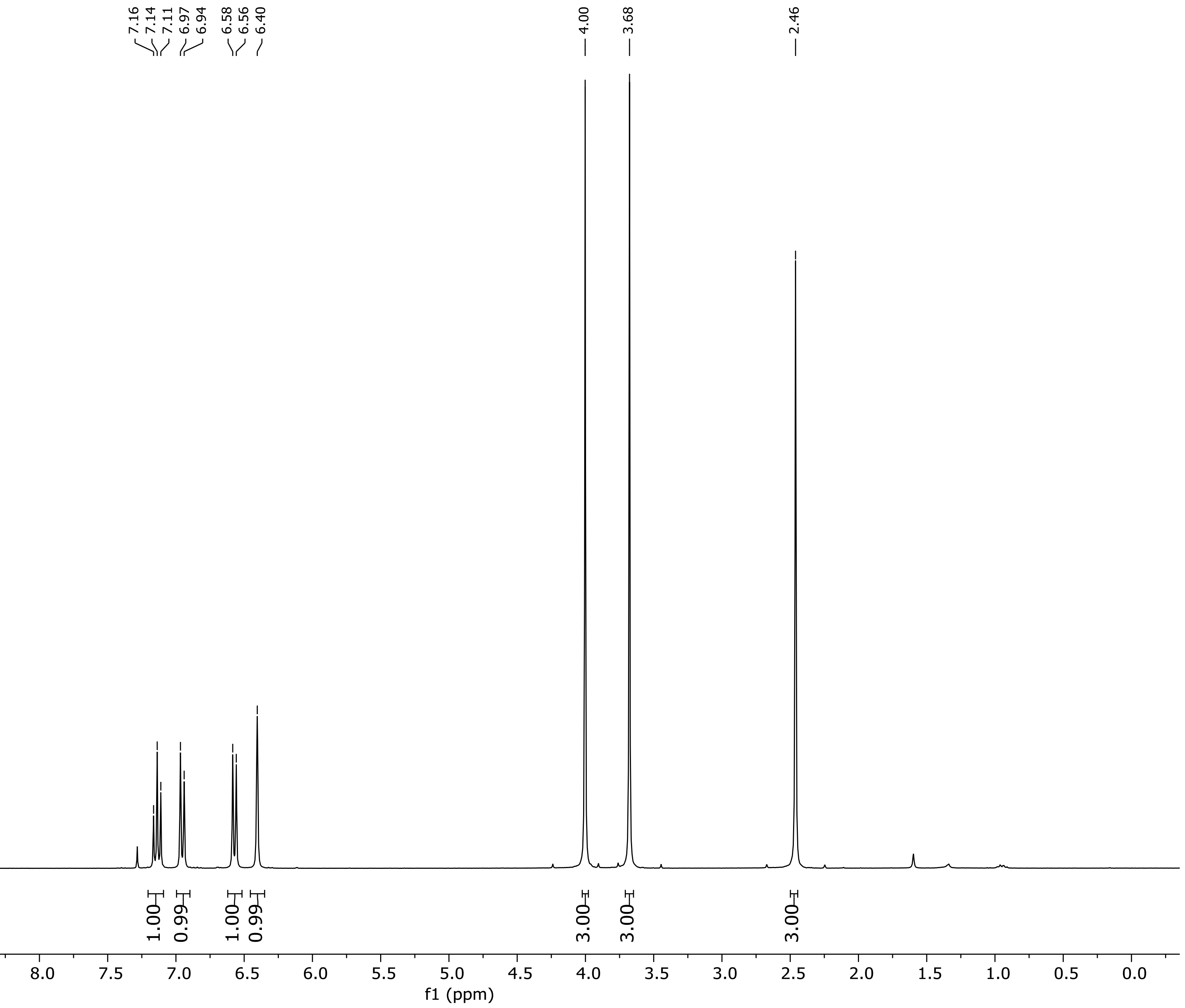

$^{13}C\{^1H\}$-NMR (75.4 MHz, $CDCl_3$)

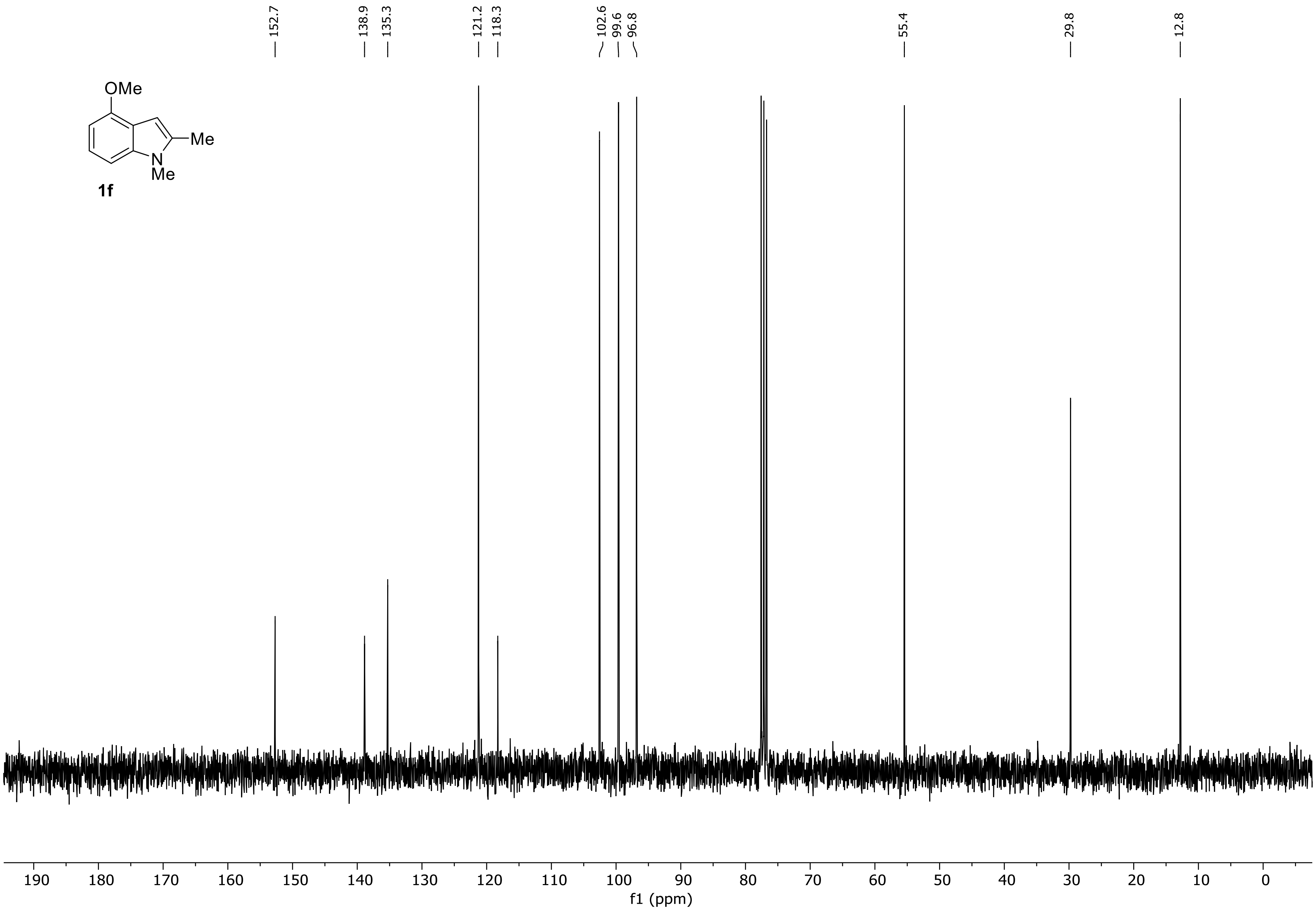

^{1}H-NMR (300 MHz, $CDCl_3$)

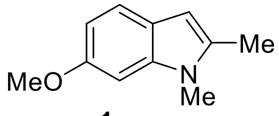

1g

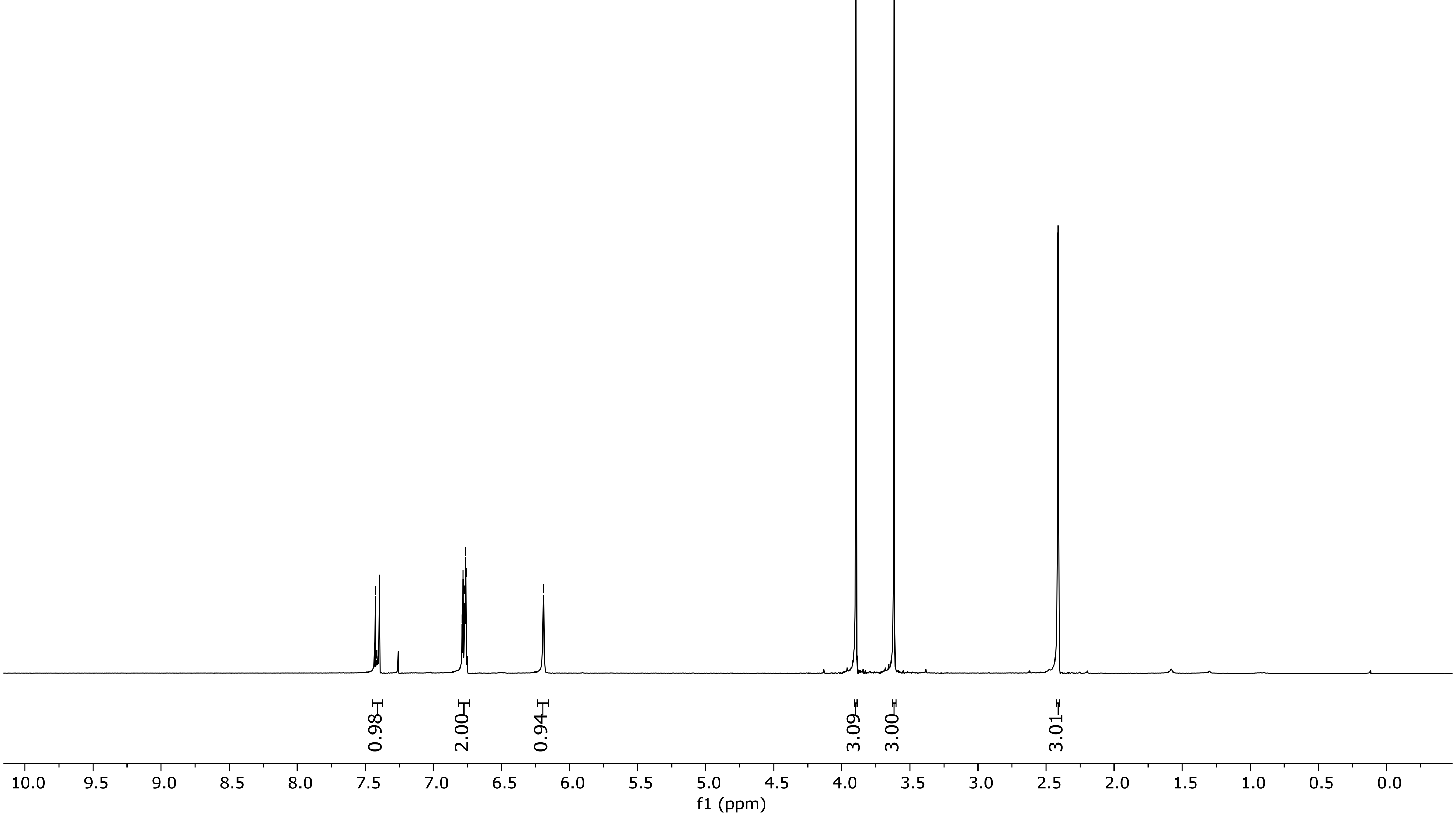

$^{13}C\{^1H\}$-NMR (75.4 MHz, $CDCl_3$)

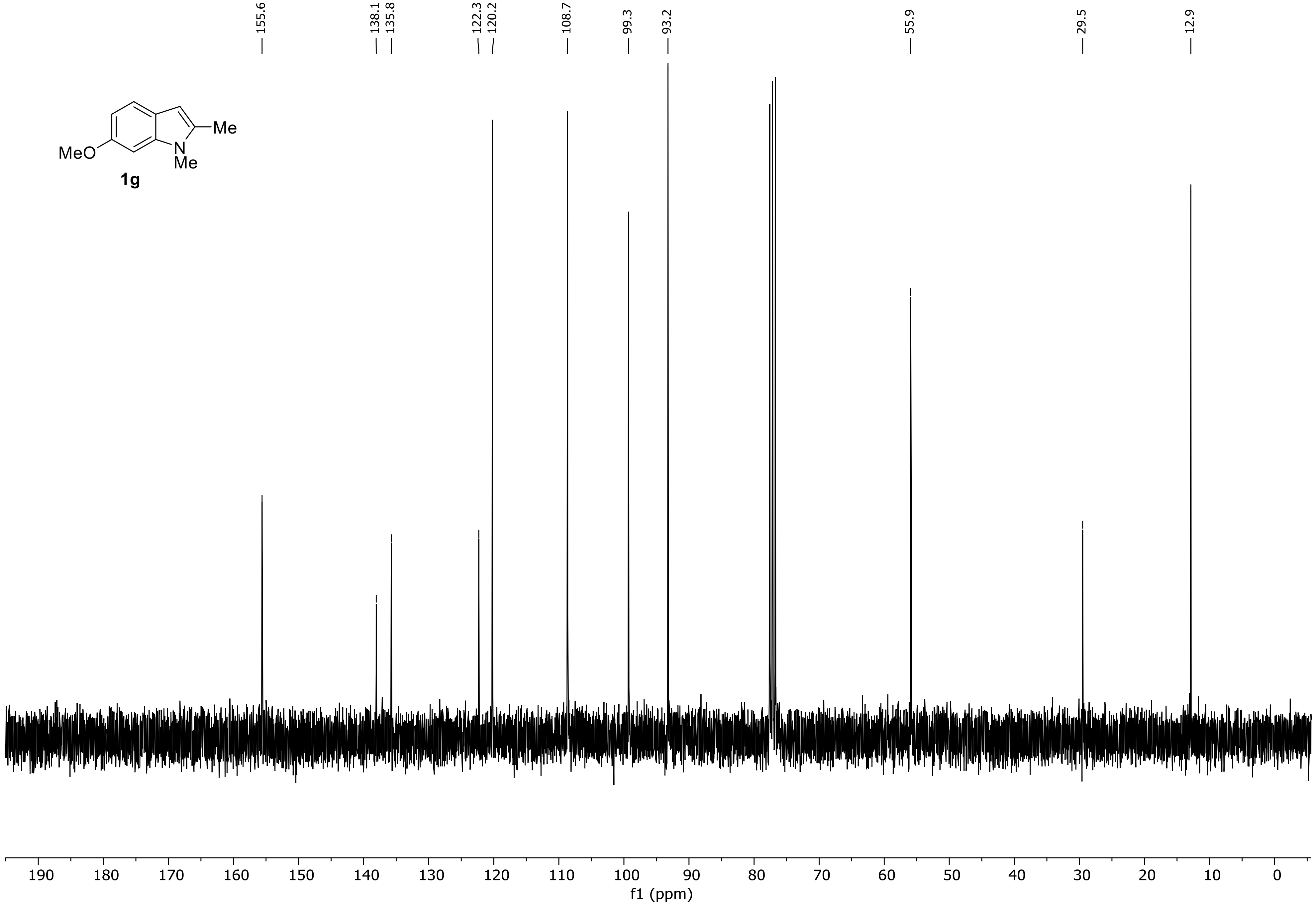

^{1}H-NMR (300 MHz, $CDCl_3$)

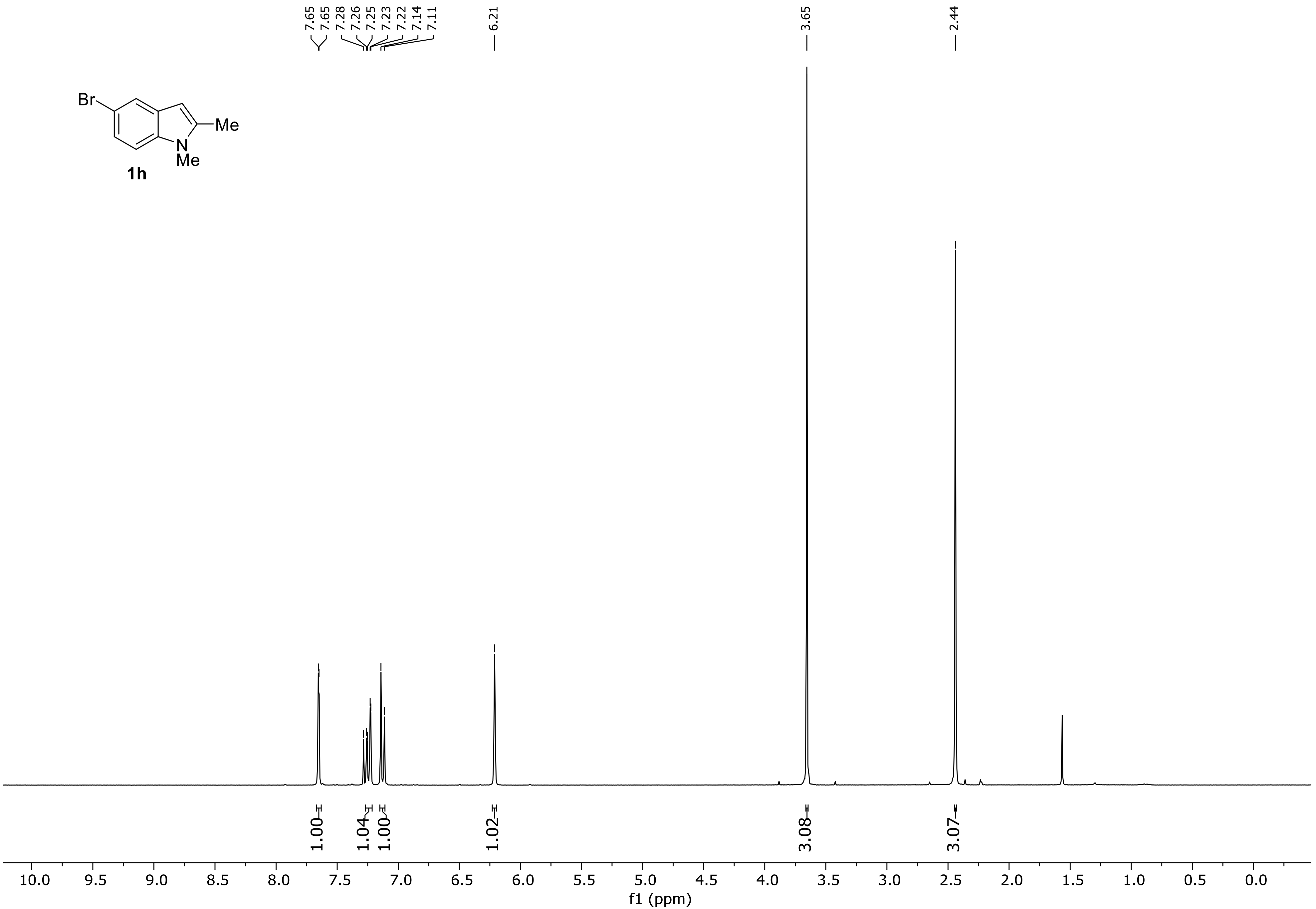

$^{13}C\{^{1}H\}$-NMR (75.4 MHz, $CDCl_3$)

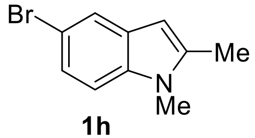

1h

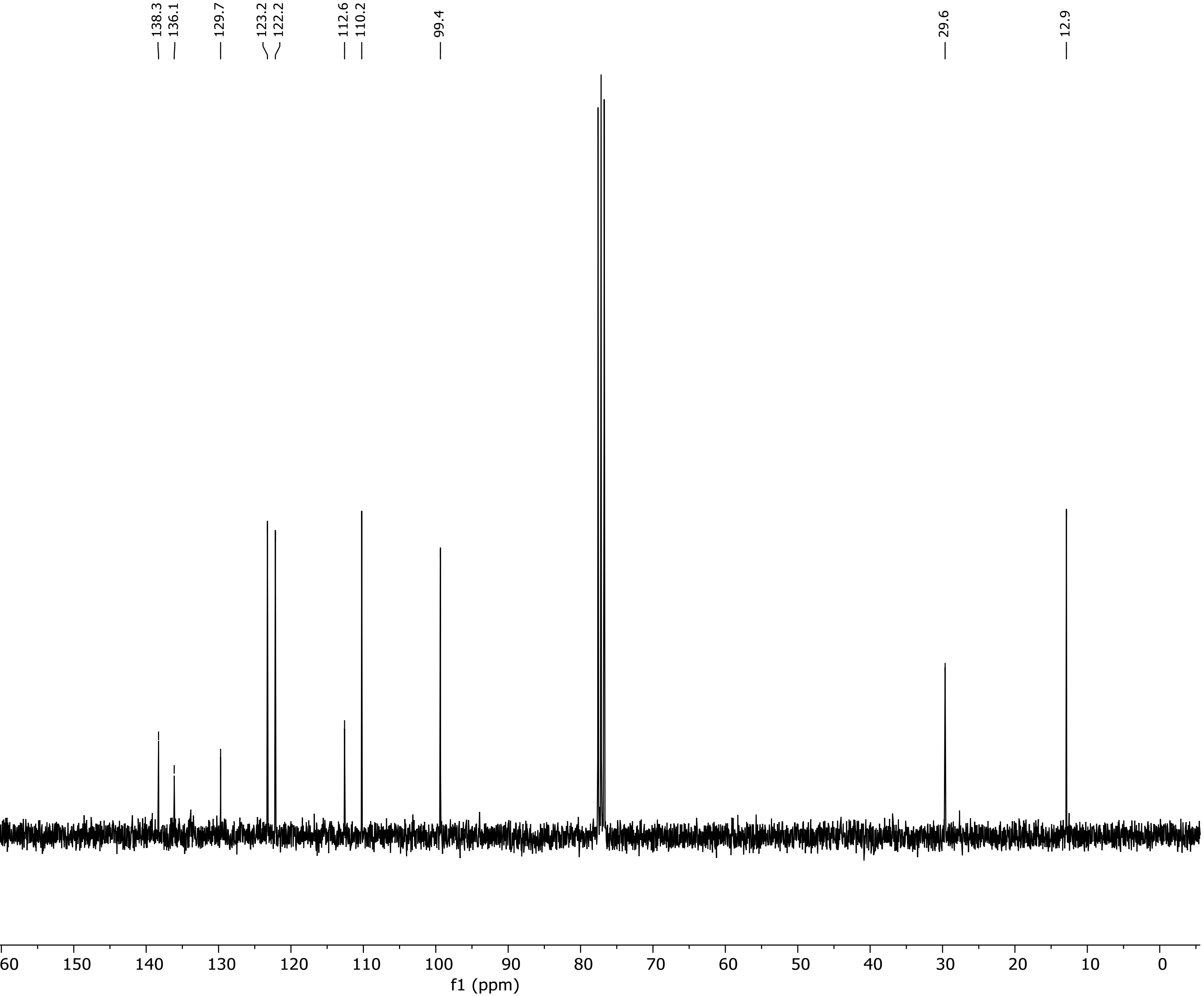

^{1}H-NMR (300 MHz, $CDCl_3$)

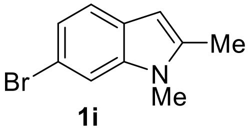

1i

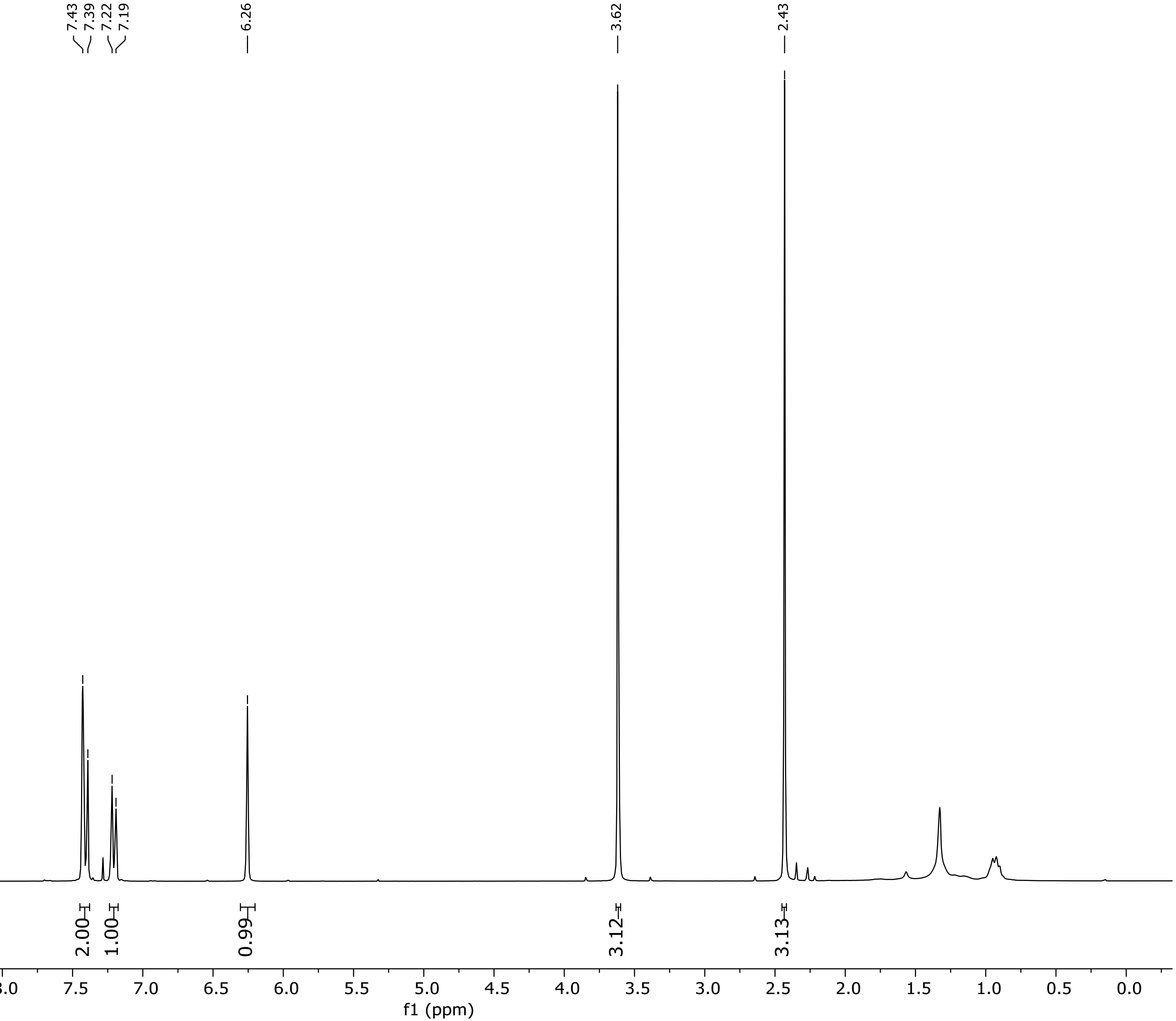

$^{13}C\{^1H\}$-NMR (75.4 MHz, $CDCl_3$)

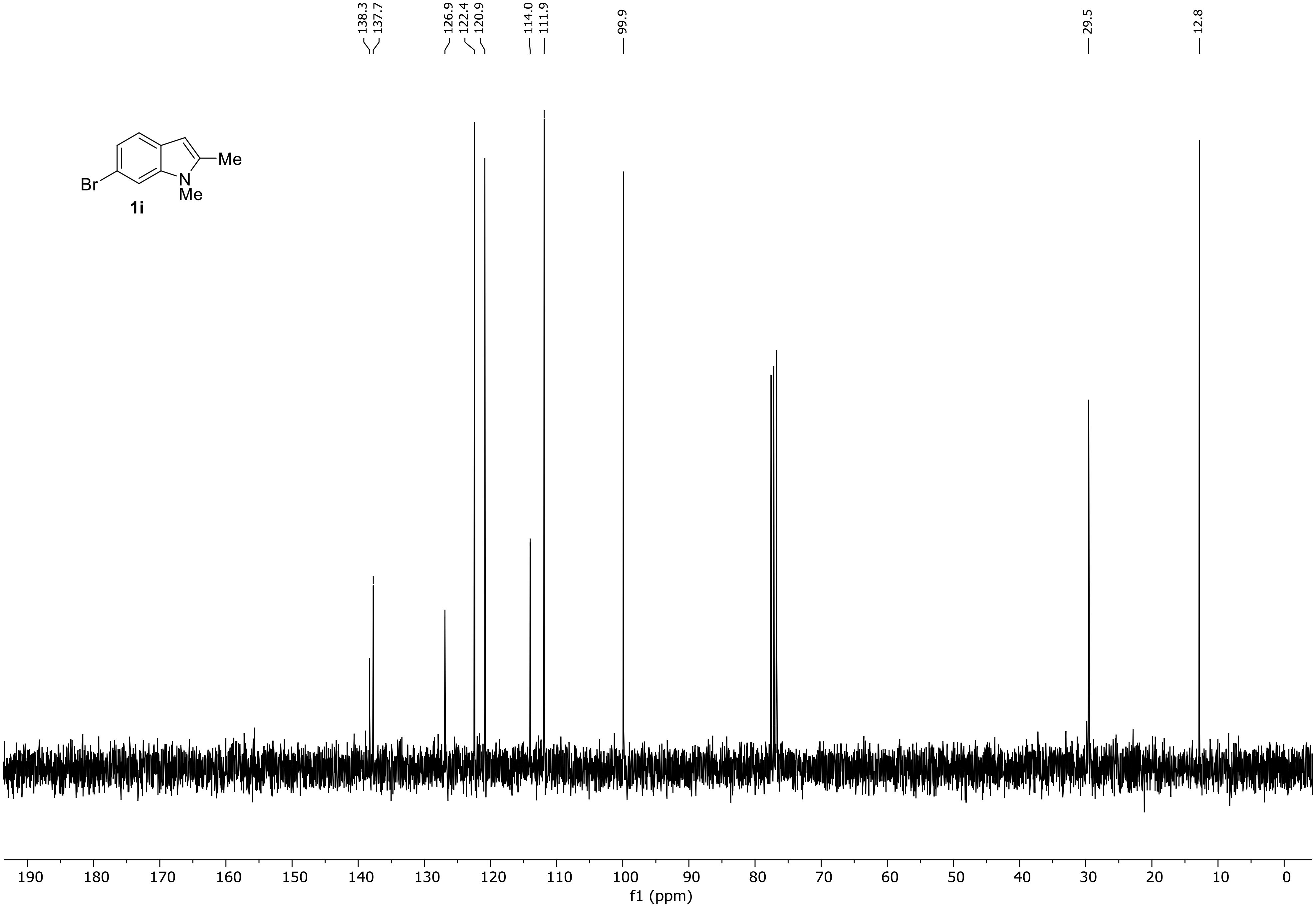

1i

^{1}H-NMR (300 MHz, $CDCl_3$)

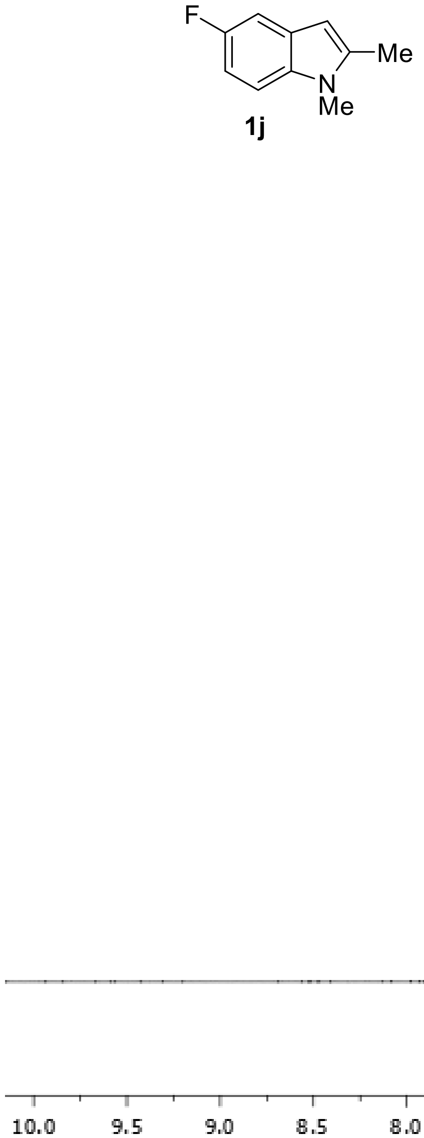

1j

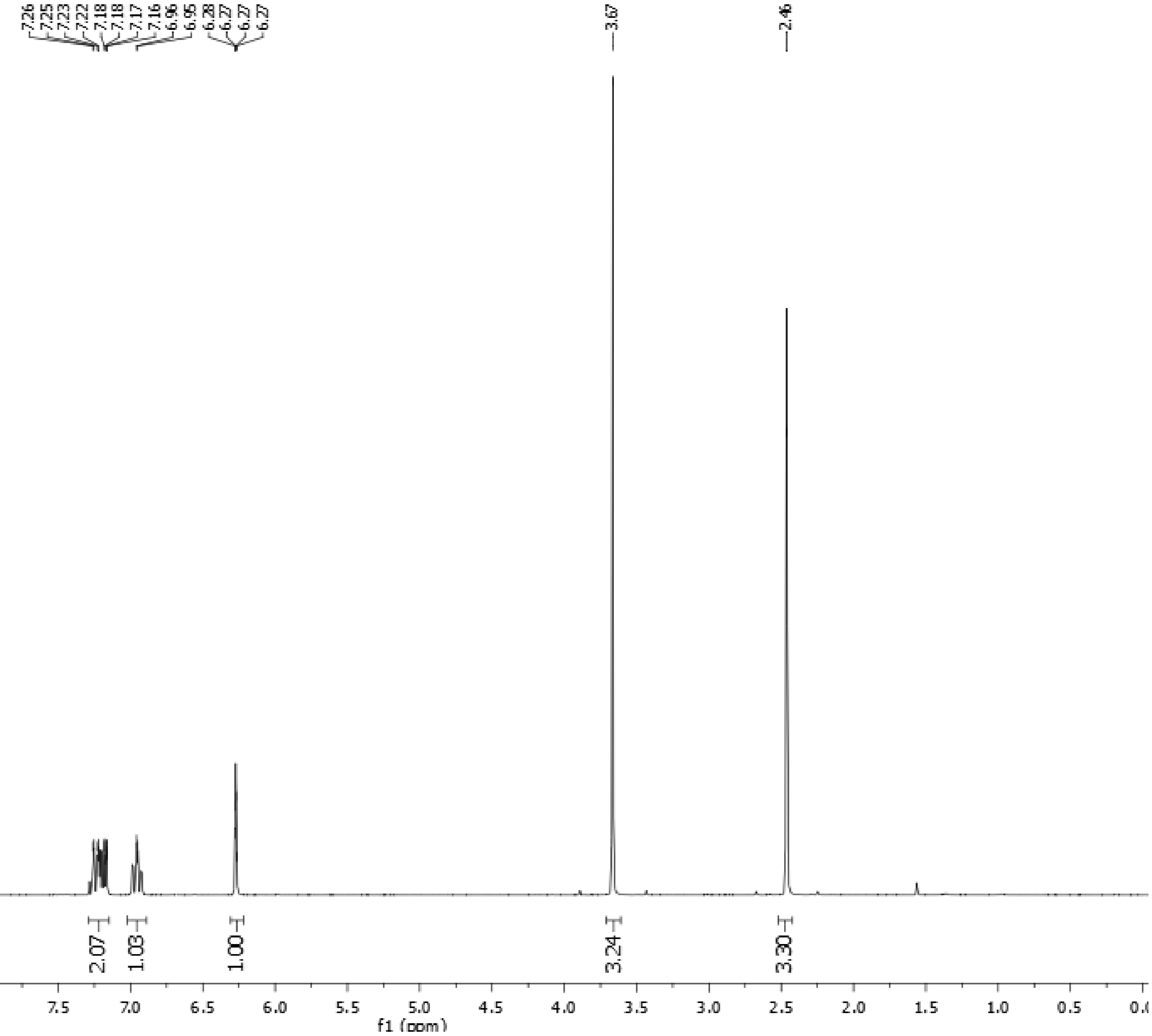

$^{13}C\{^{1}H\}$-NMR (75.4 MHz, $CDCl_3$)

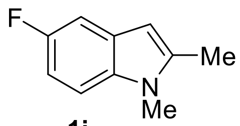

1j

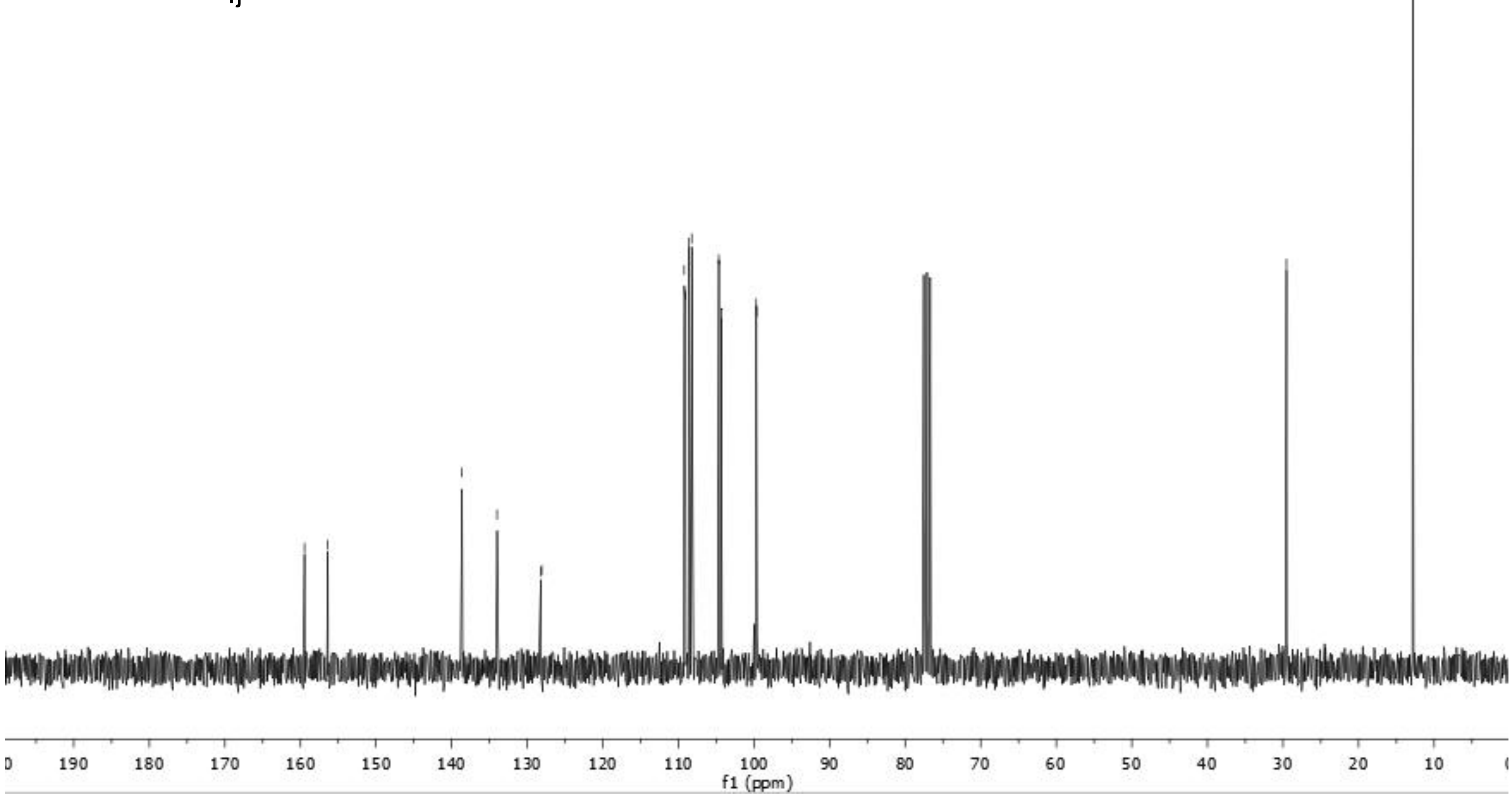

^{19}F-NMR (252 MHz, $CDCl_3$)

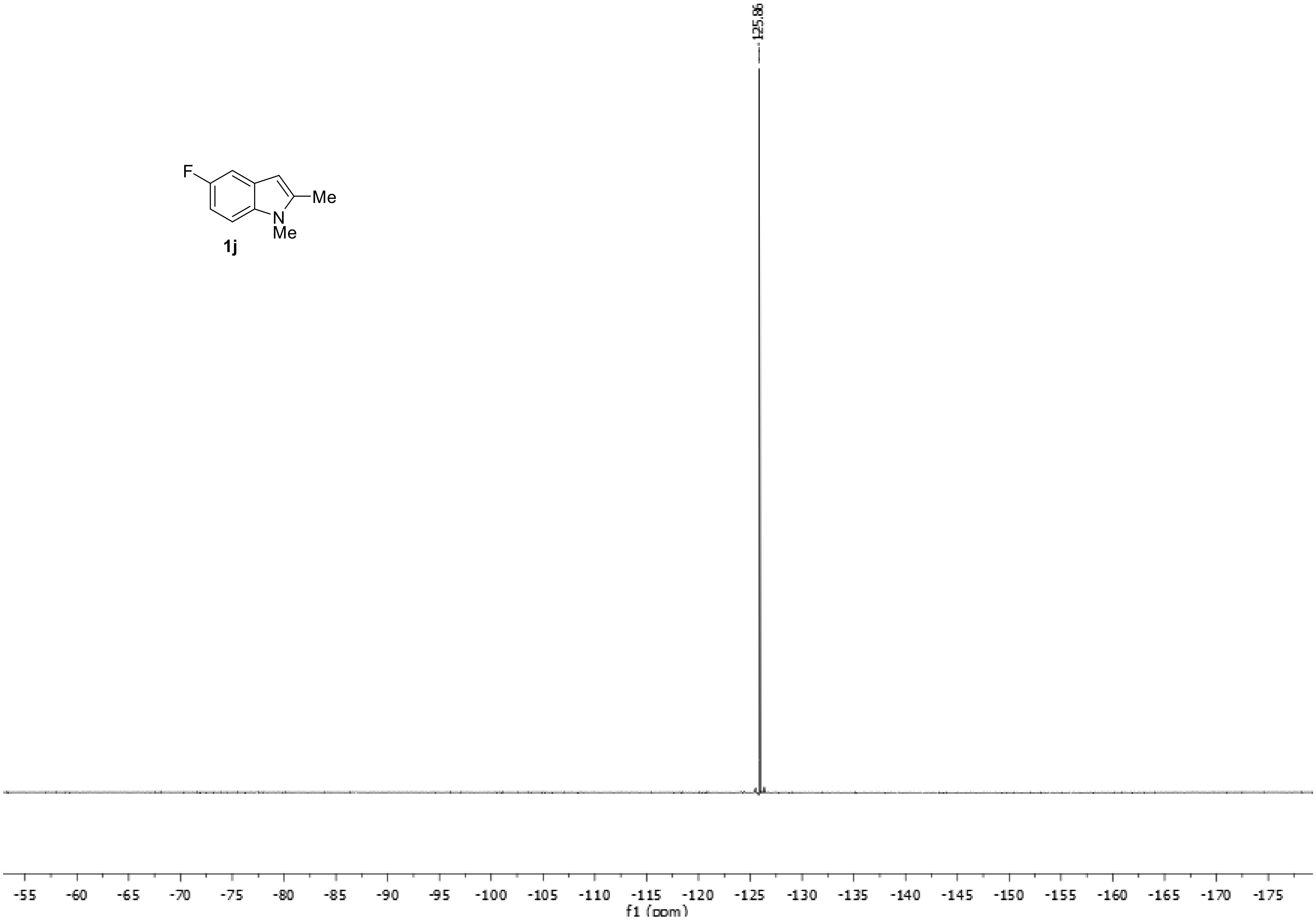

^{1}H-NMR (75.4 MHz, $CDCl_3$)

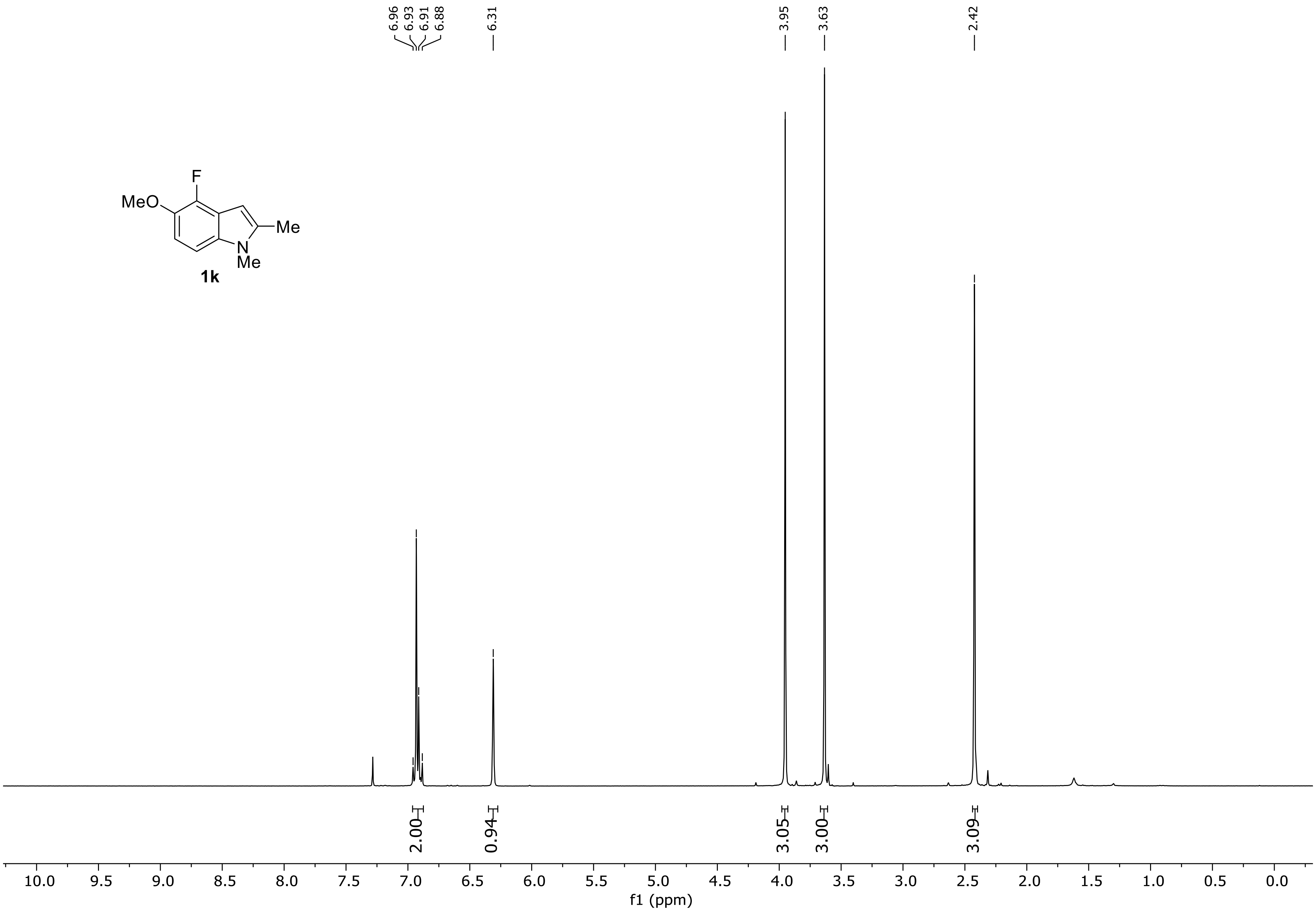

$^{13}C\{^{1}H\}$-NMR (300 MHz, $CDCl_3$)

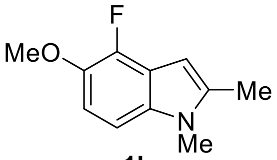

1k

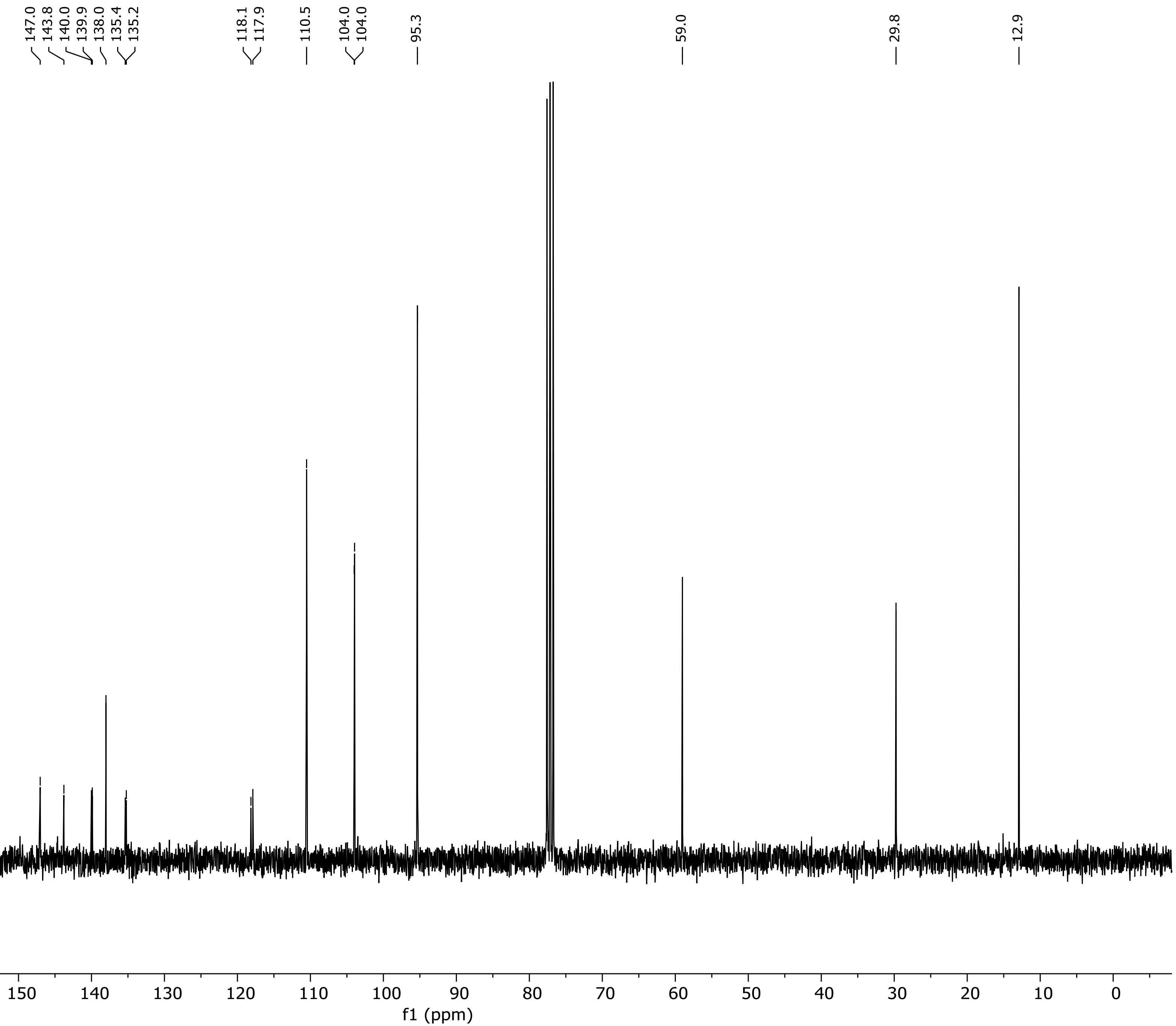

^{1}H-NMR (75.4 MHz, $CDCl_3$)

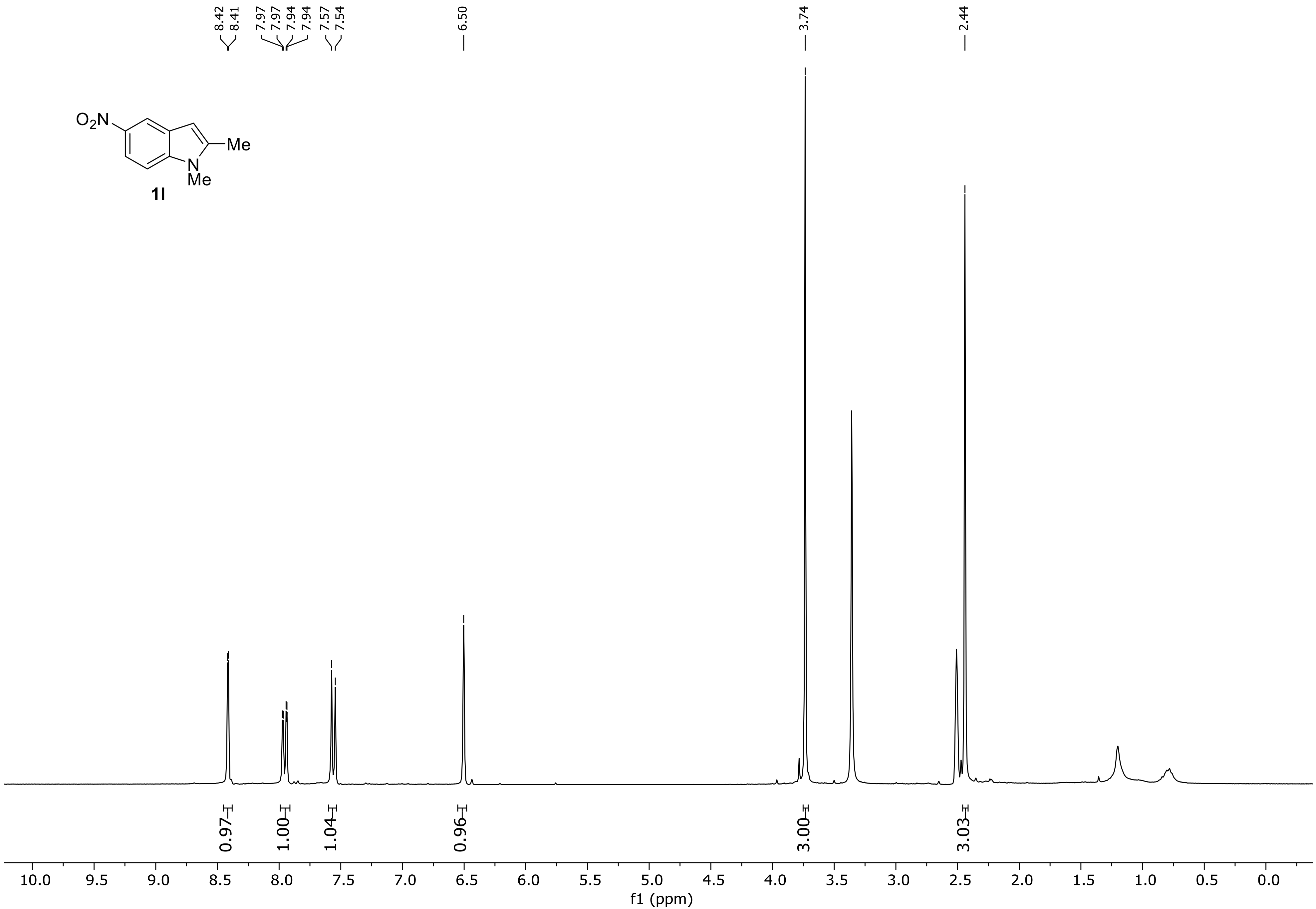

$^{13}C\{^{1}H\}$-NMR (300 MHz, $CDCl_3$)

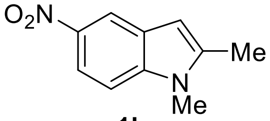

1l

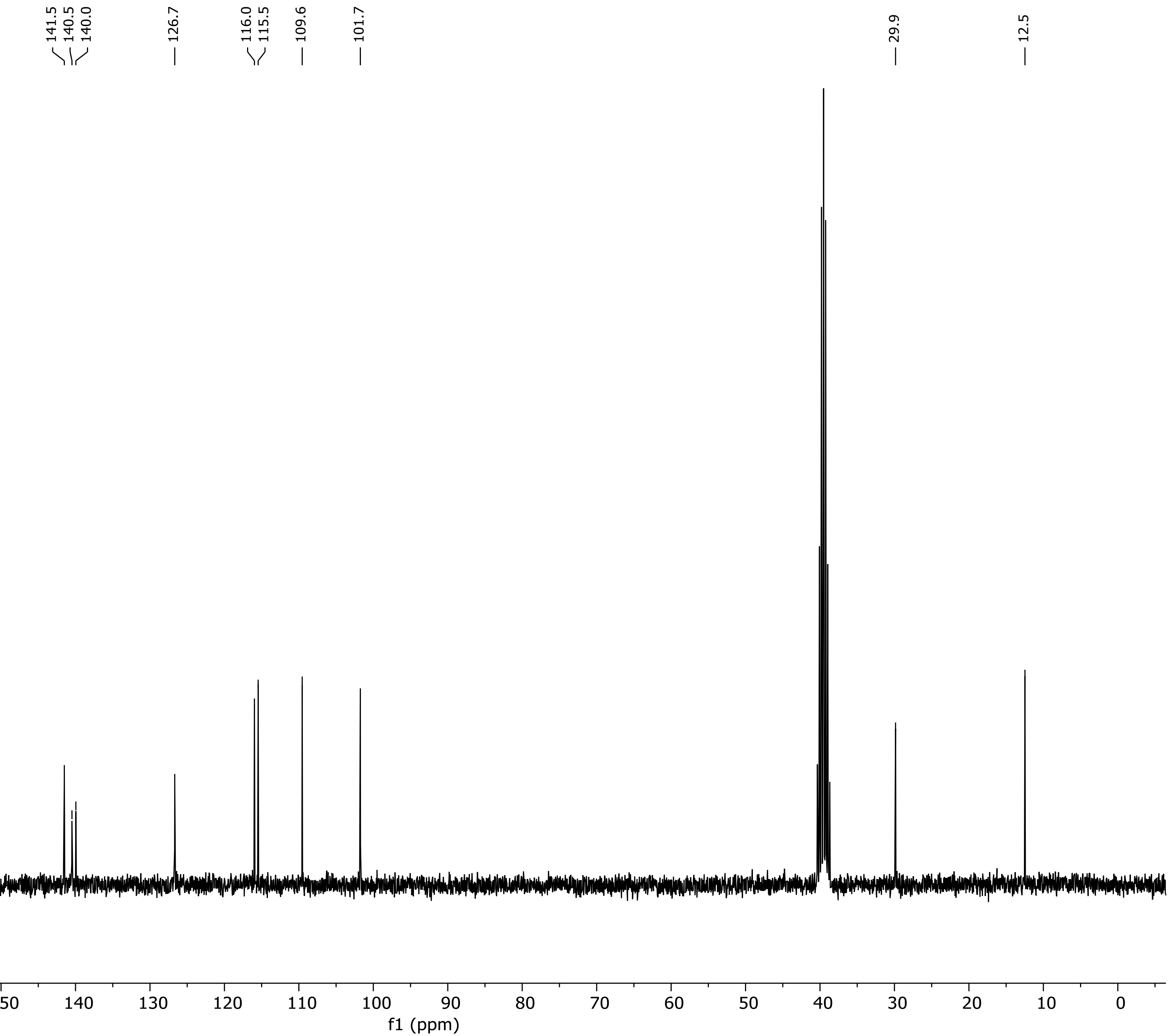

^{1}H-NMR (75.4 MHz, $CDCl_3$)

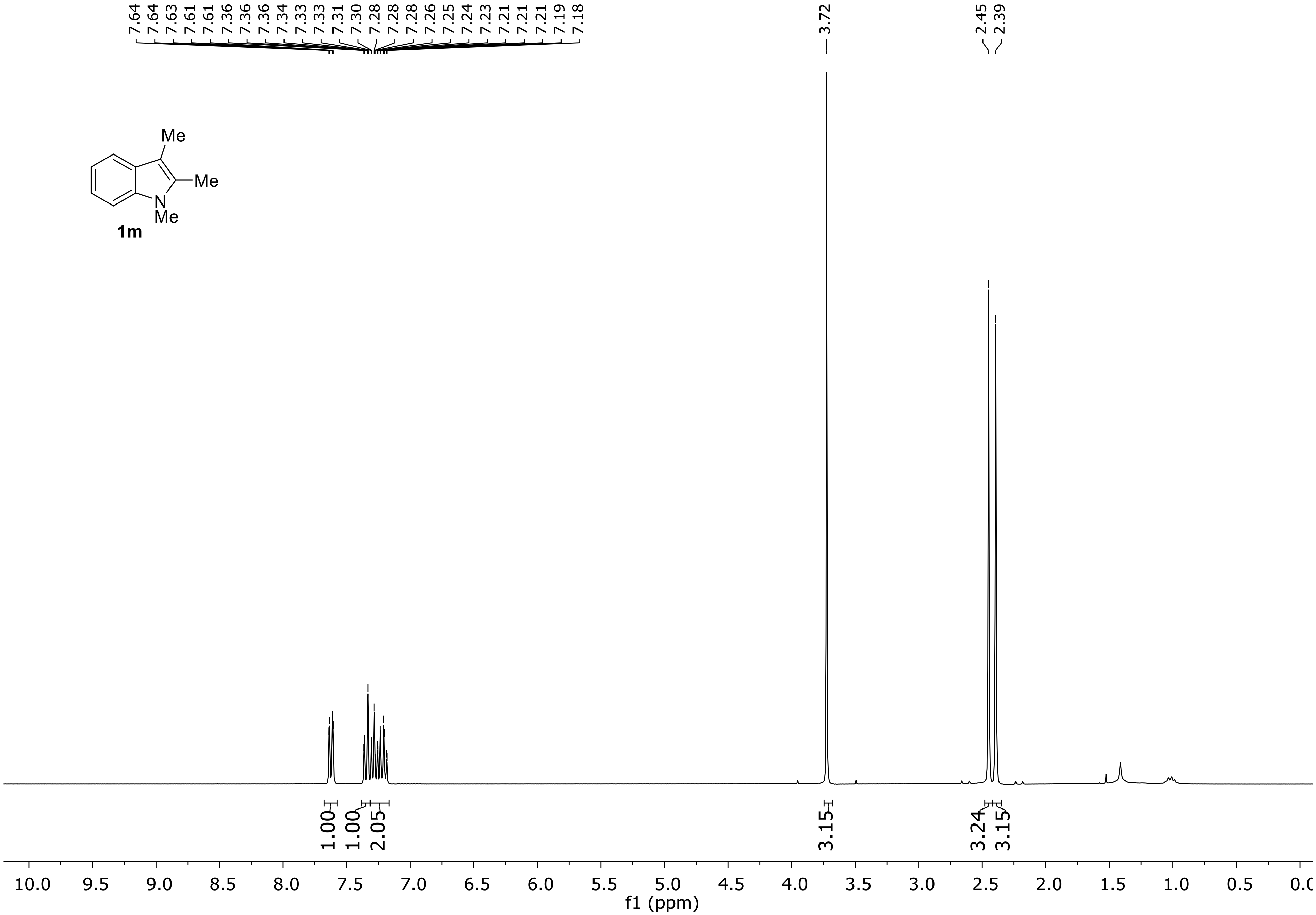

$^{13}C\{^{1}H\}$-NMR (300 MHz, $CDCl_3$)

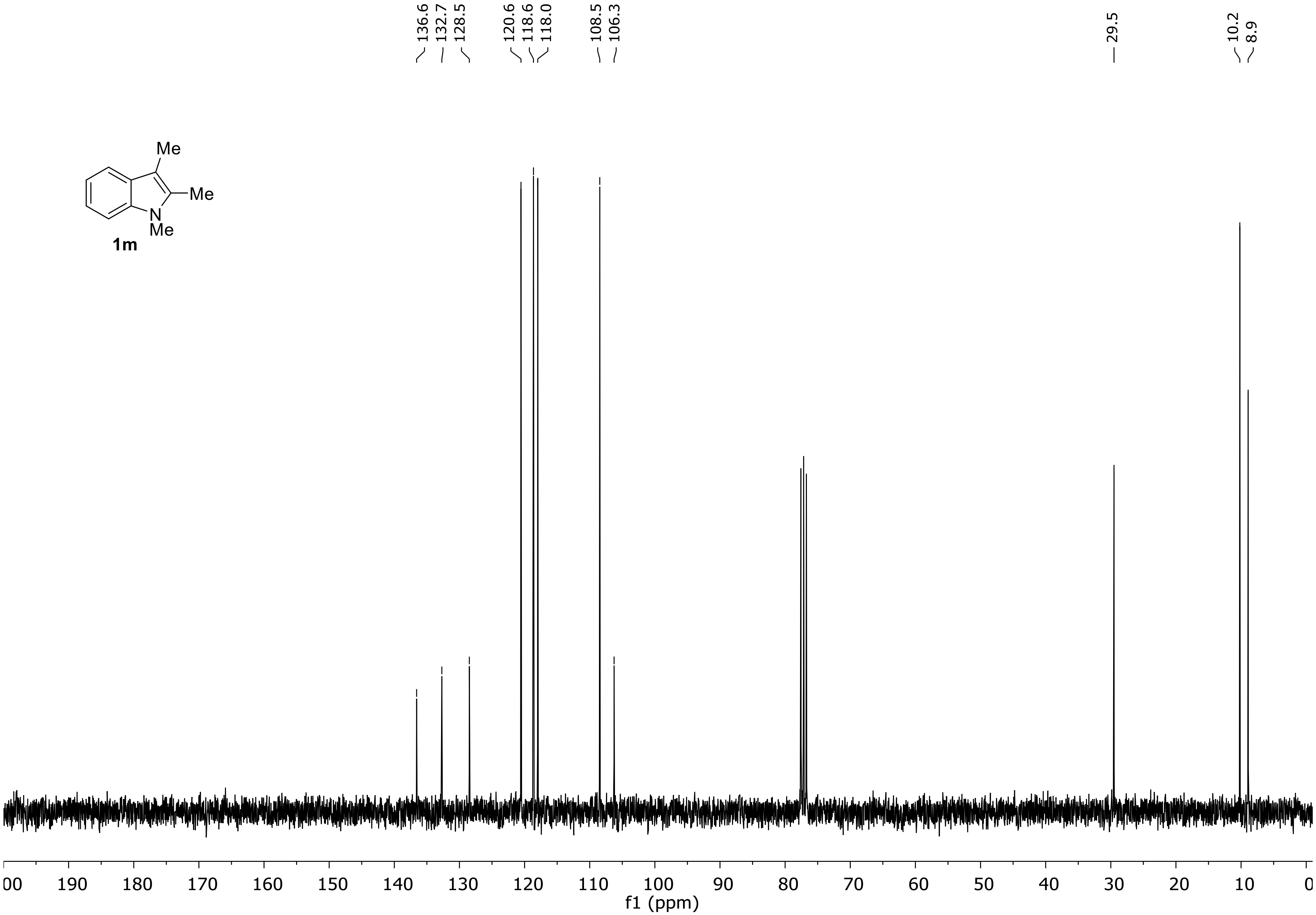

^{1}H-NMR (75.4 MHz, $CDCl_3$)

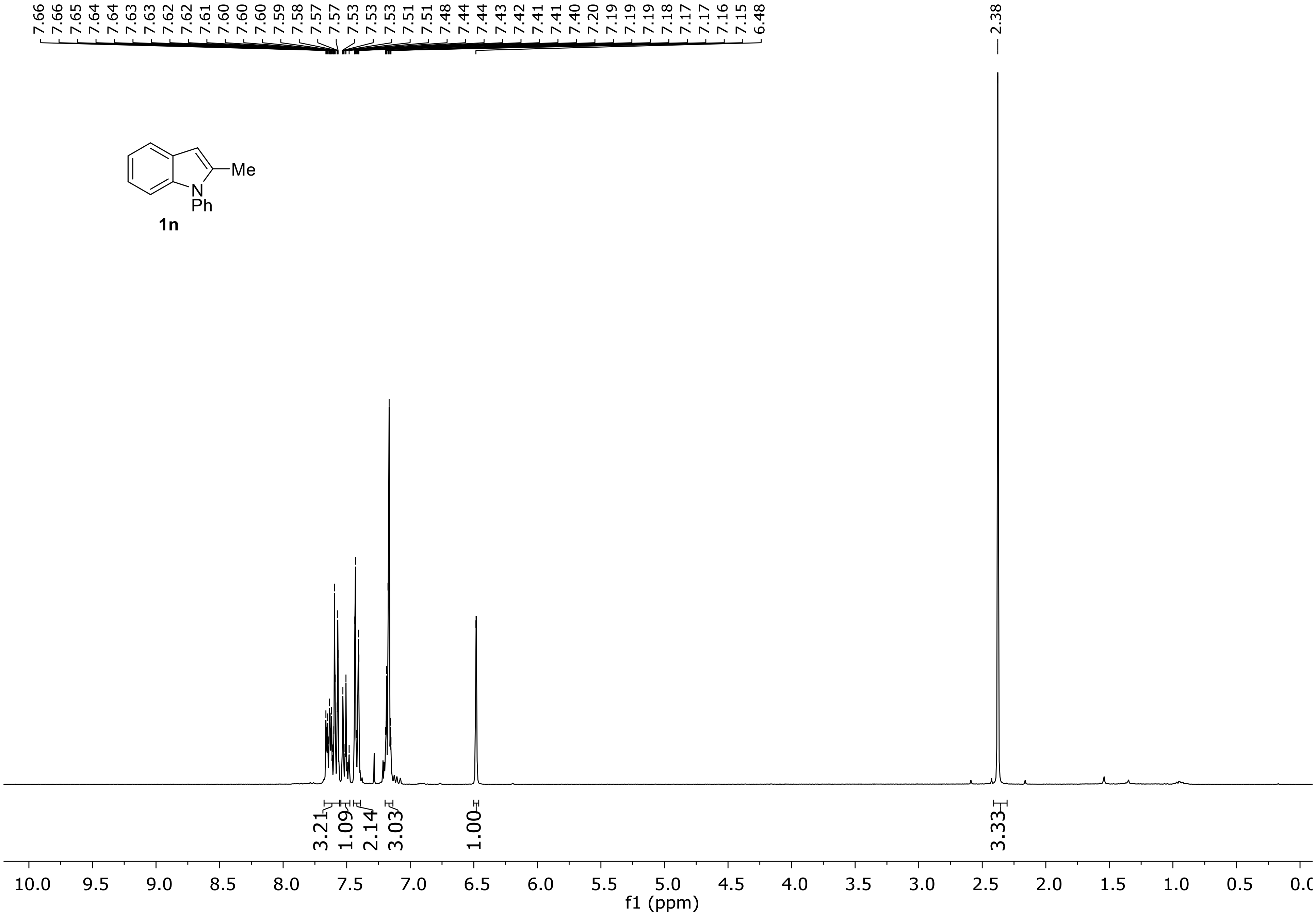

$^{13}C\{^1H\}$-NMR (300 MHz, $CDCl_3$)

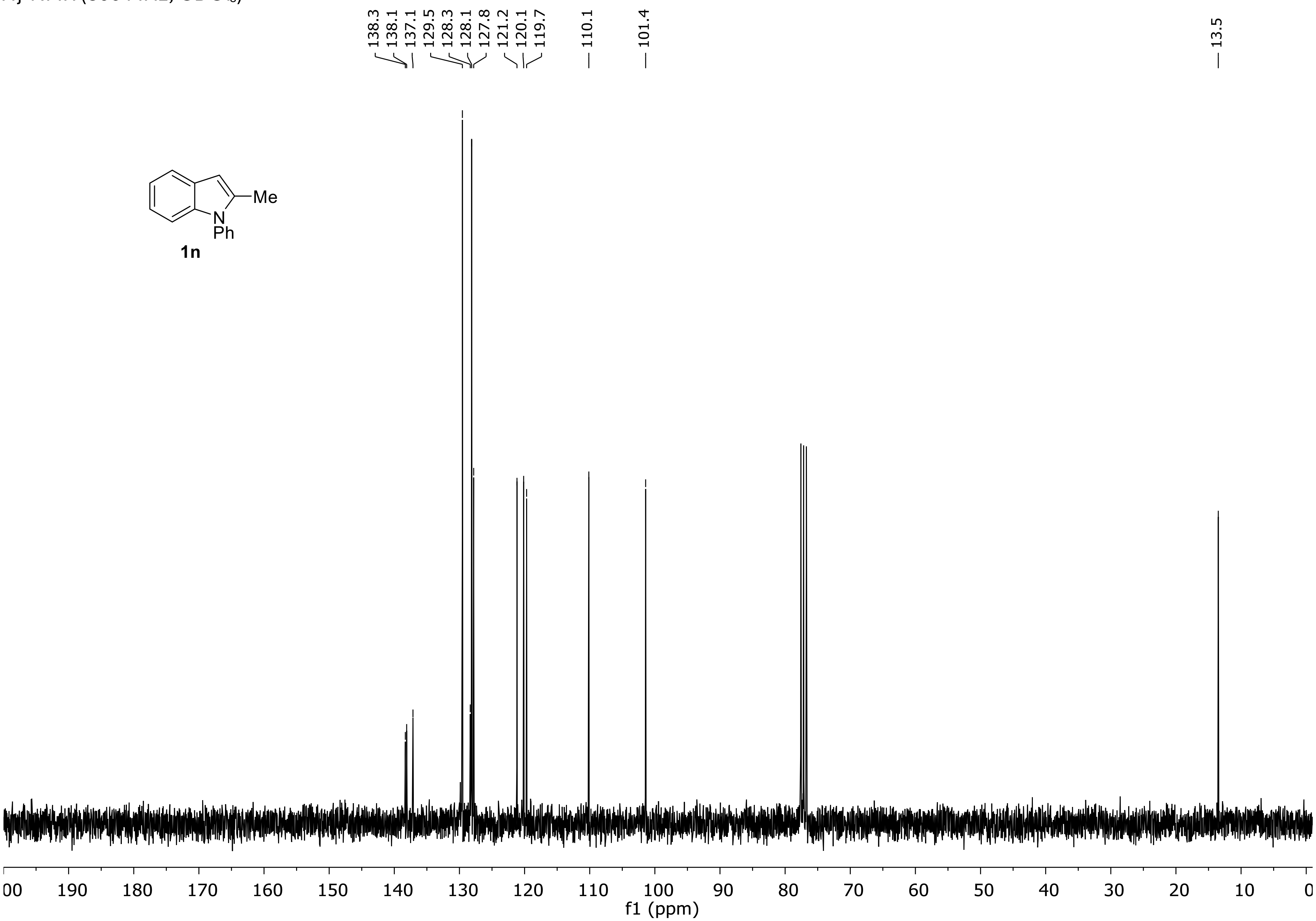

^{1}H-NMR (75.4 MHz, $CDCl_3$)

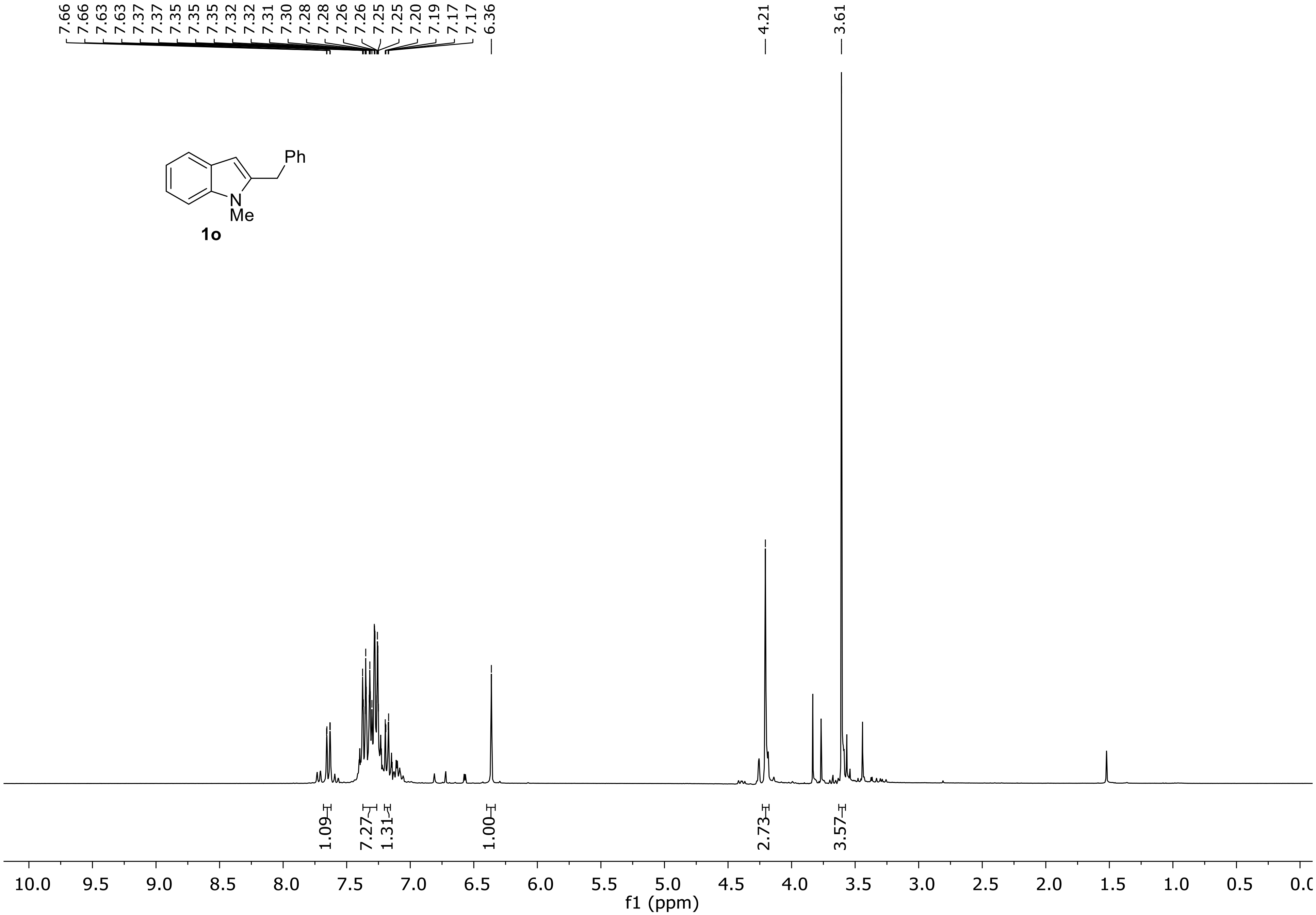

1o

$^{13}C\{^{1}H\}$-NMR (300 MHz, $CDCl_3$)

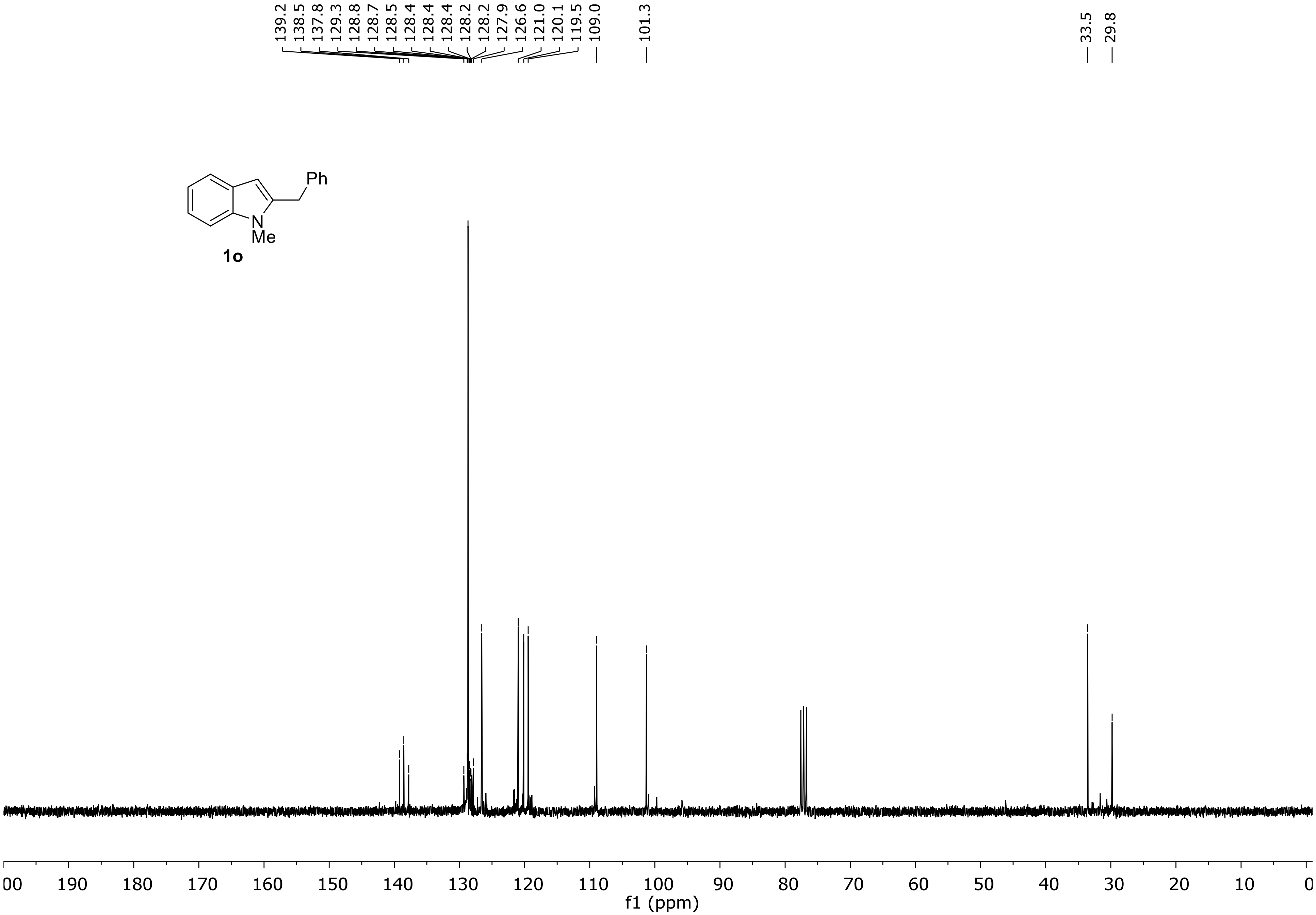

^{1}H-NMR (126 MHz, $CDCl_3$)

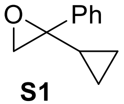

S1

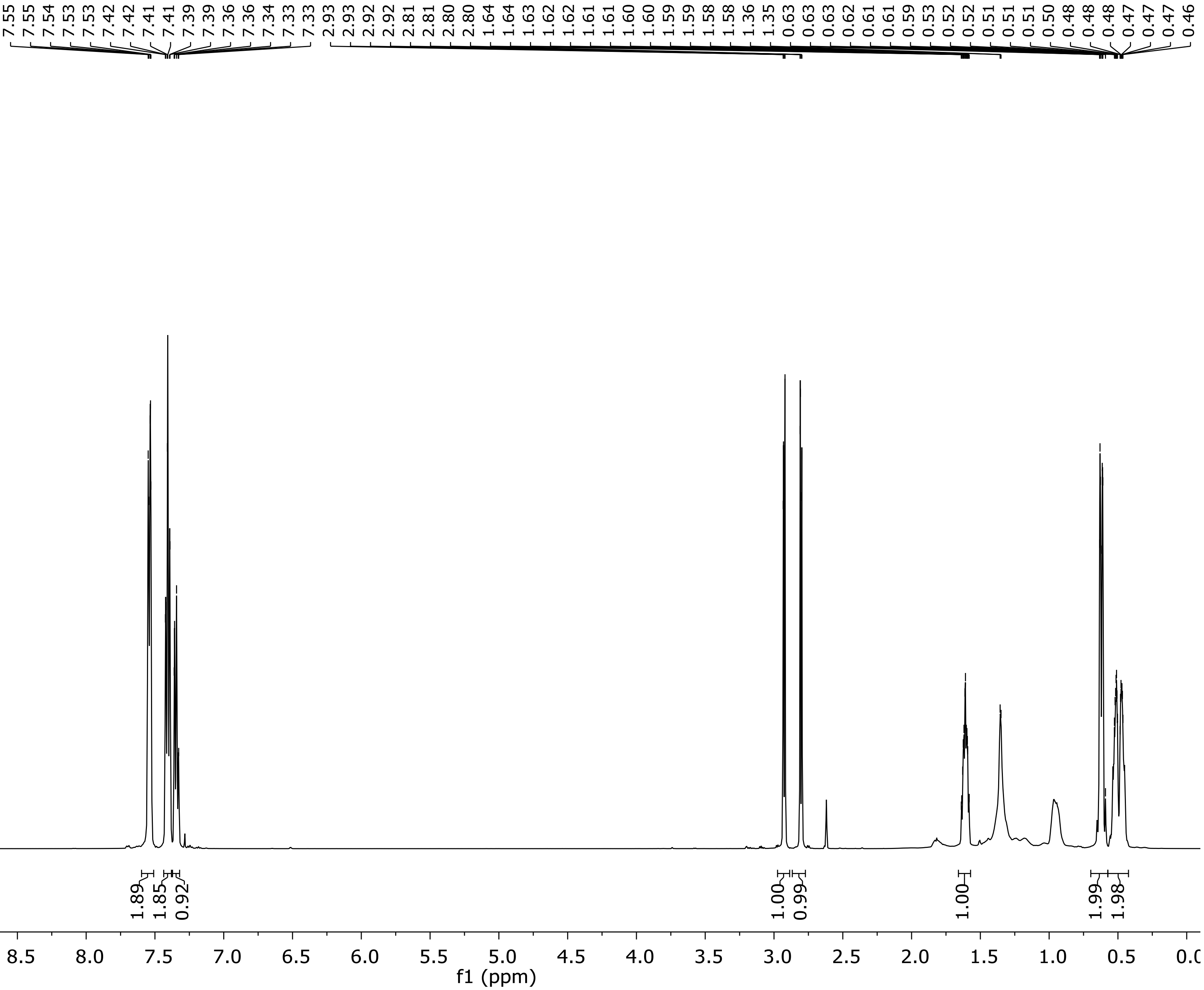

$^{13}C\{^1H\}$-NMR (500 MHz, $CDCl_3$)

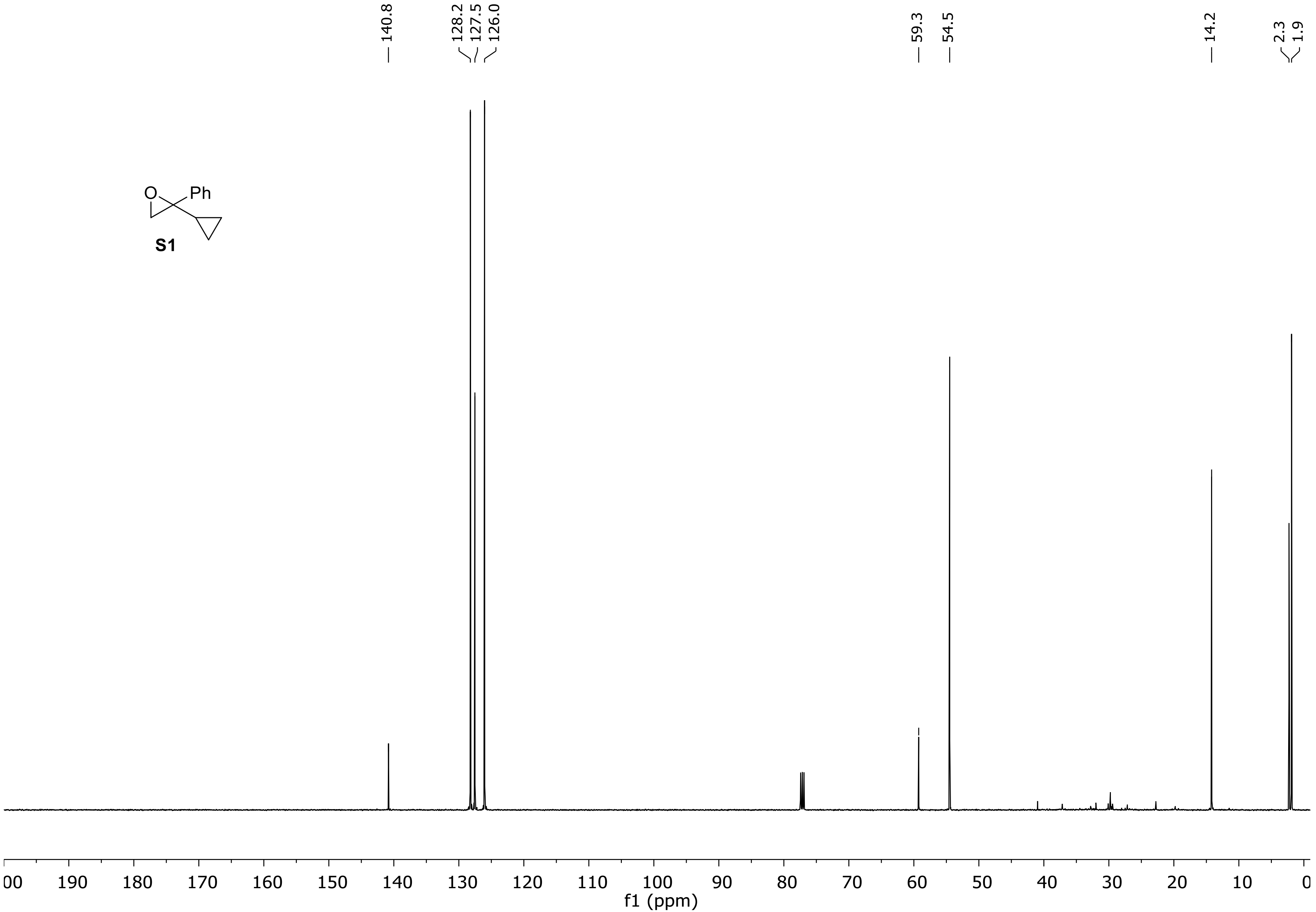

^{1}H-NMR (75.4 MHz, $CDCl_3$)

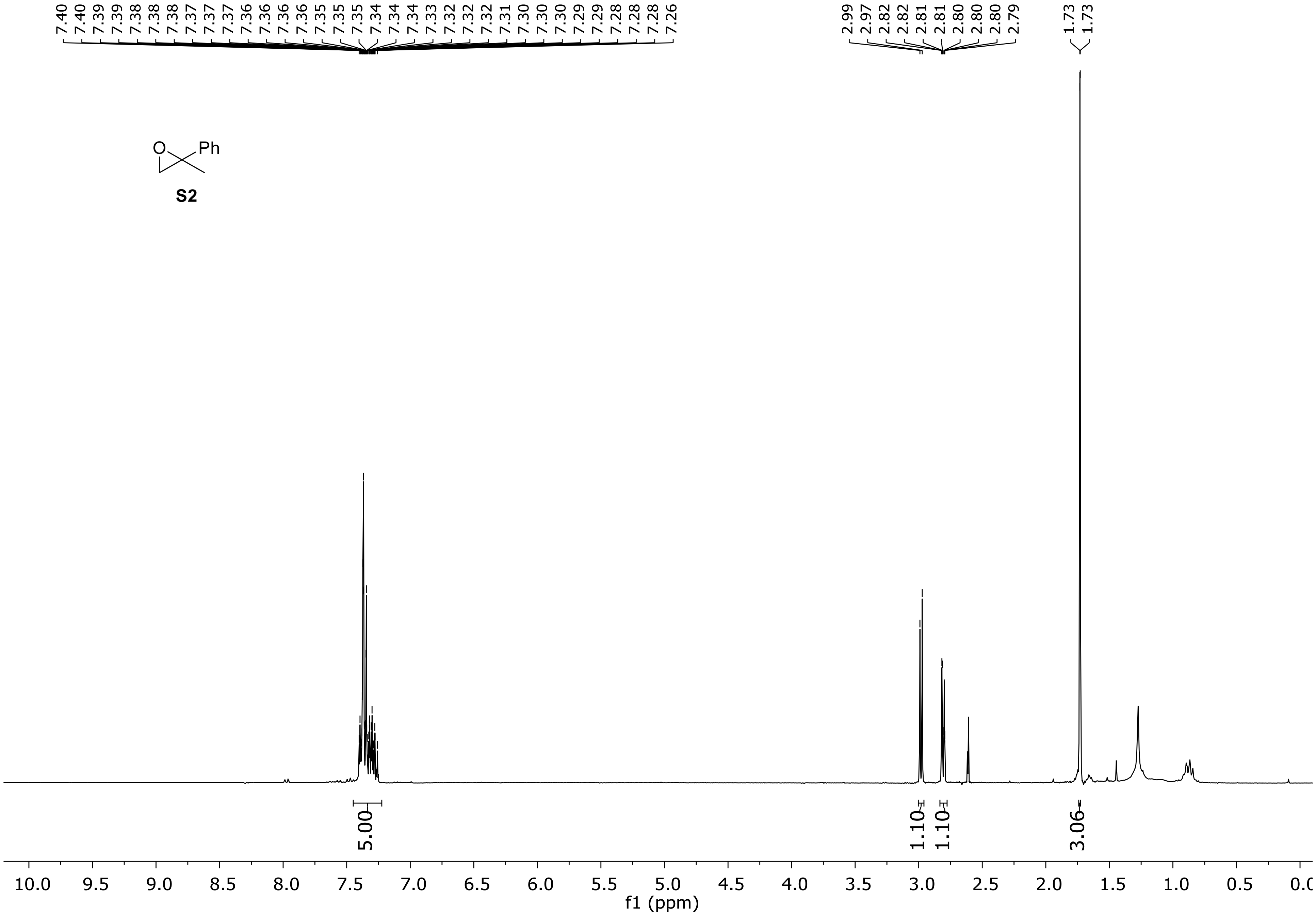

$^{13}C\{^1H\}$-NMR (300 MHz, $CDCl_3$)

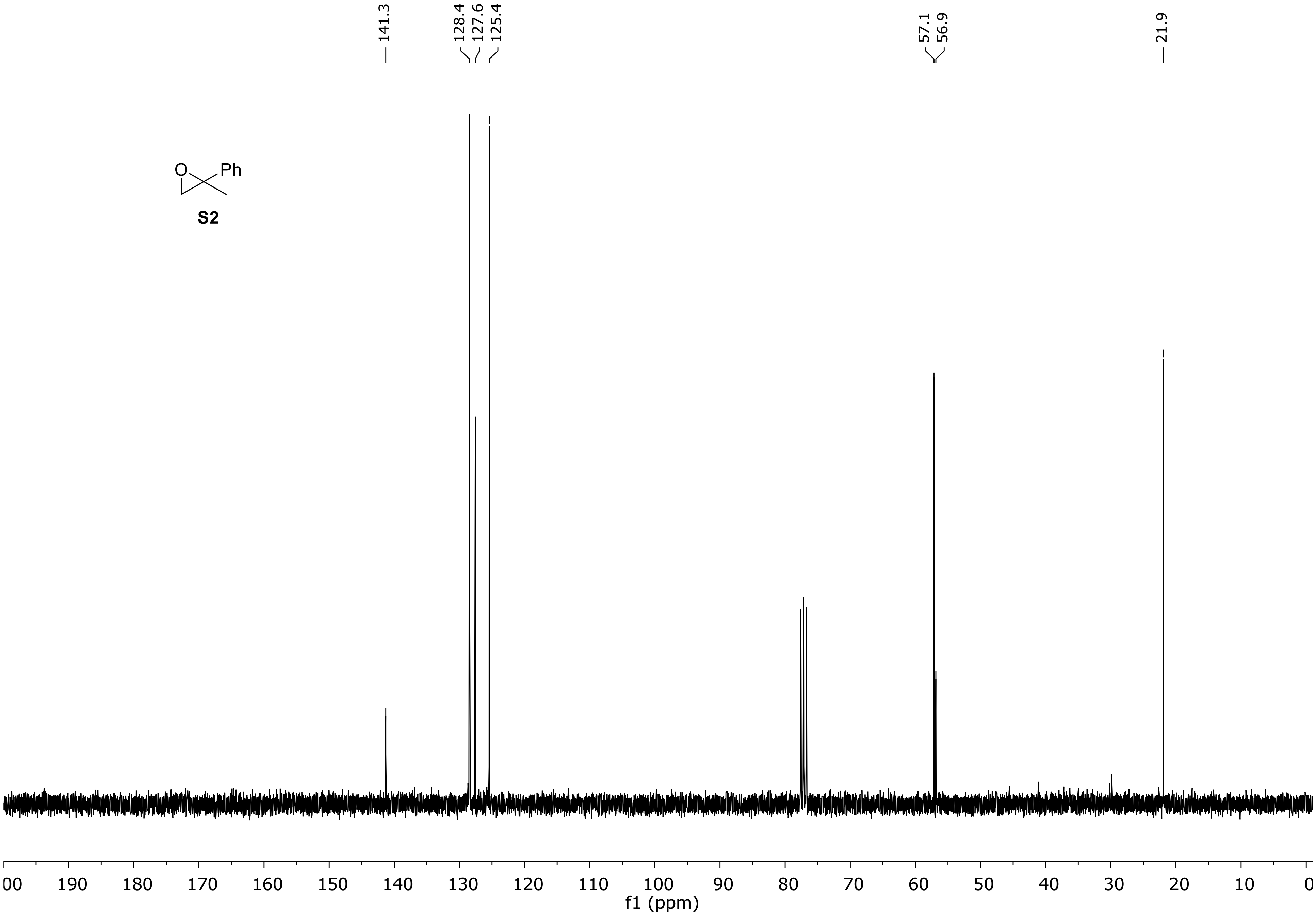

^{1}H-NMR (75.4 MHz, $CDCl_3$)

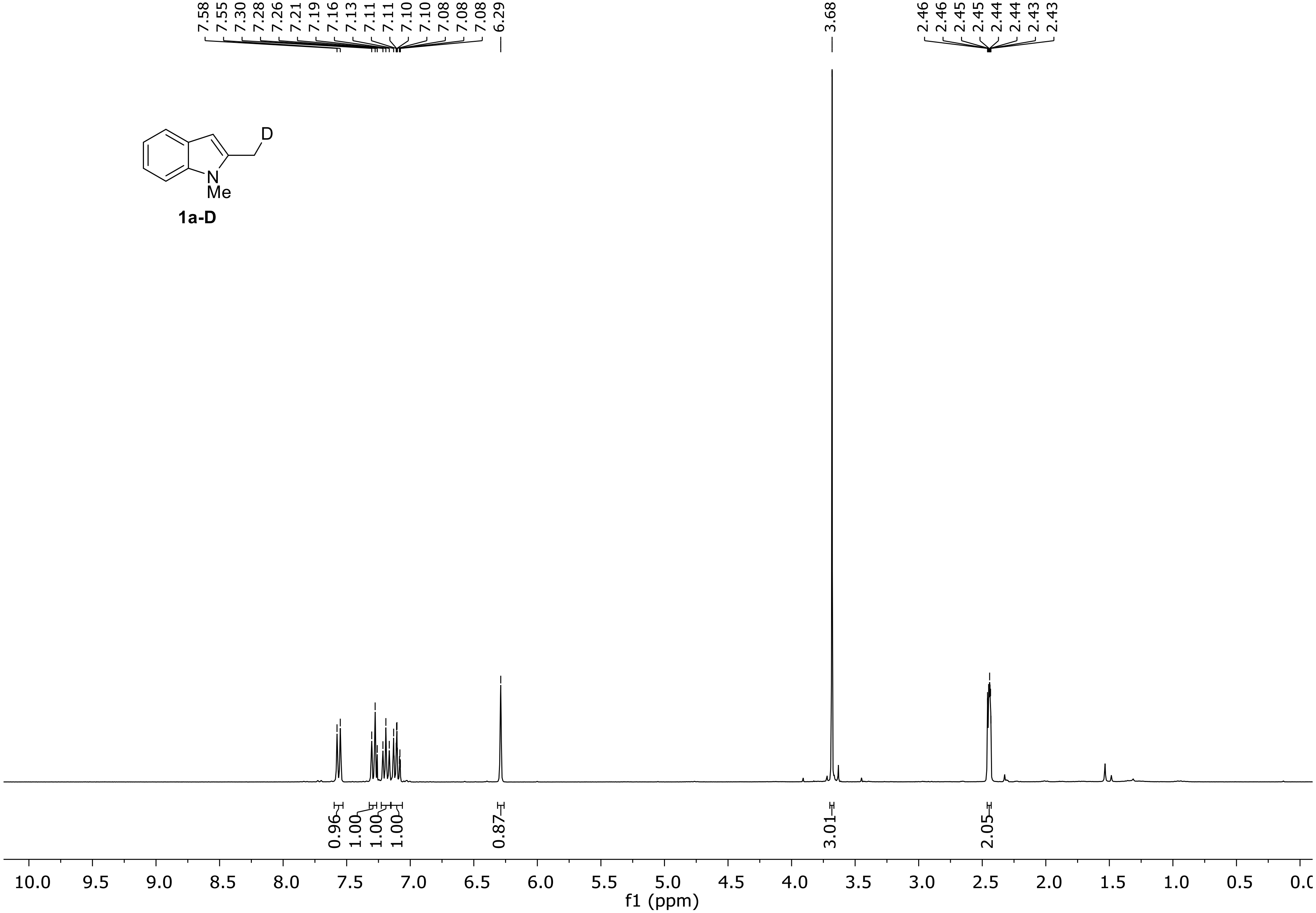

$^{13}C\{^{1}H\}$-NMR (300 MHz, $CDCl_3$)

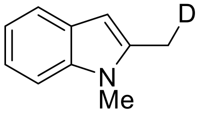

1a-D

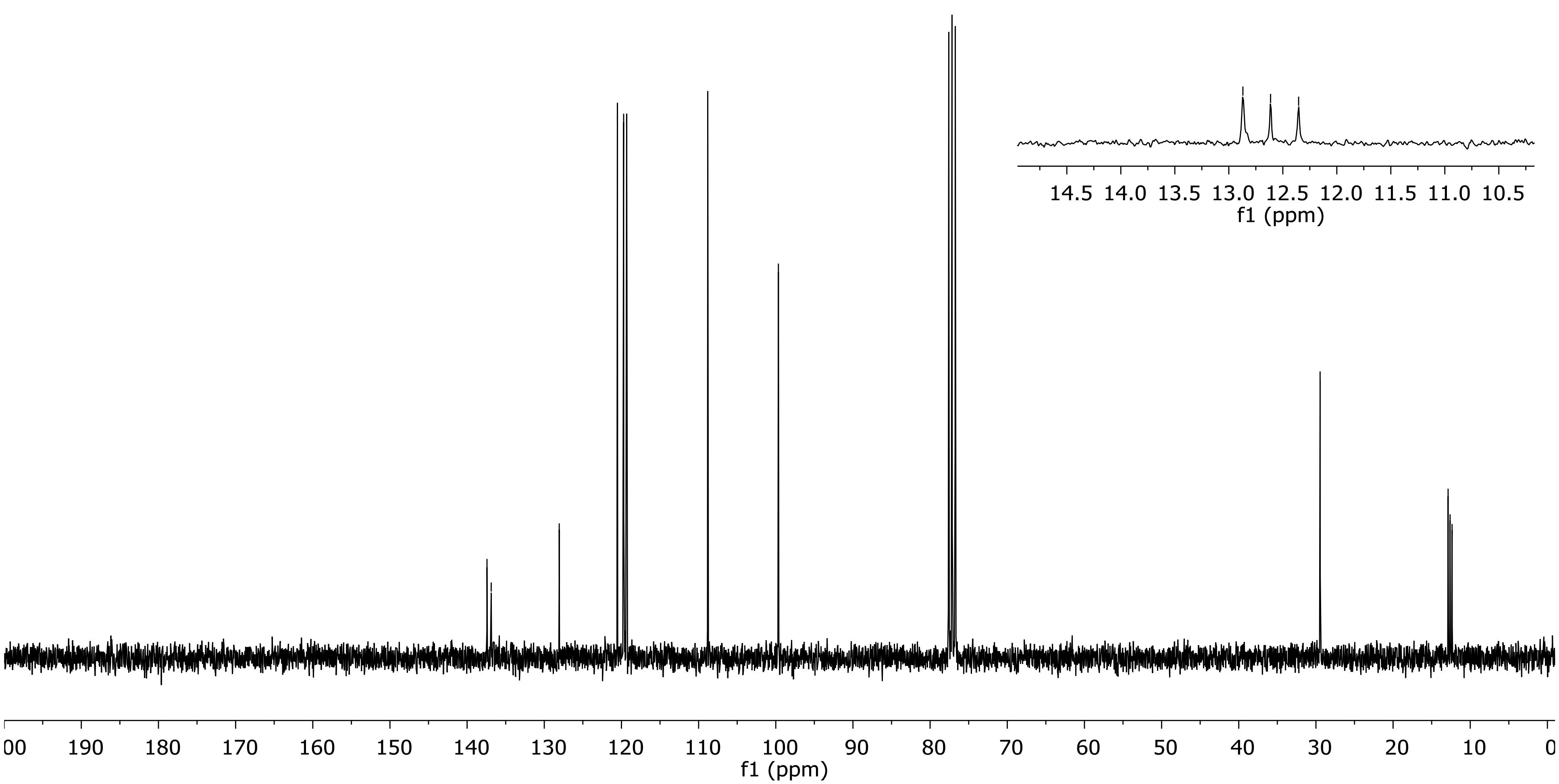

^{1}H-NMR (75.4 MHz, $CDCl_3$)

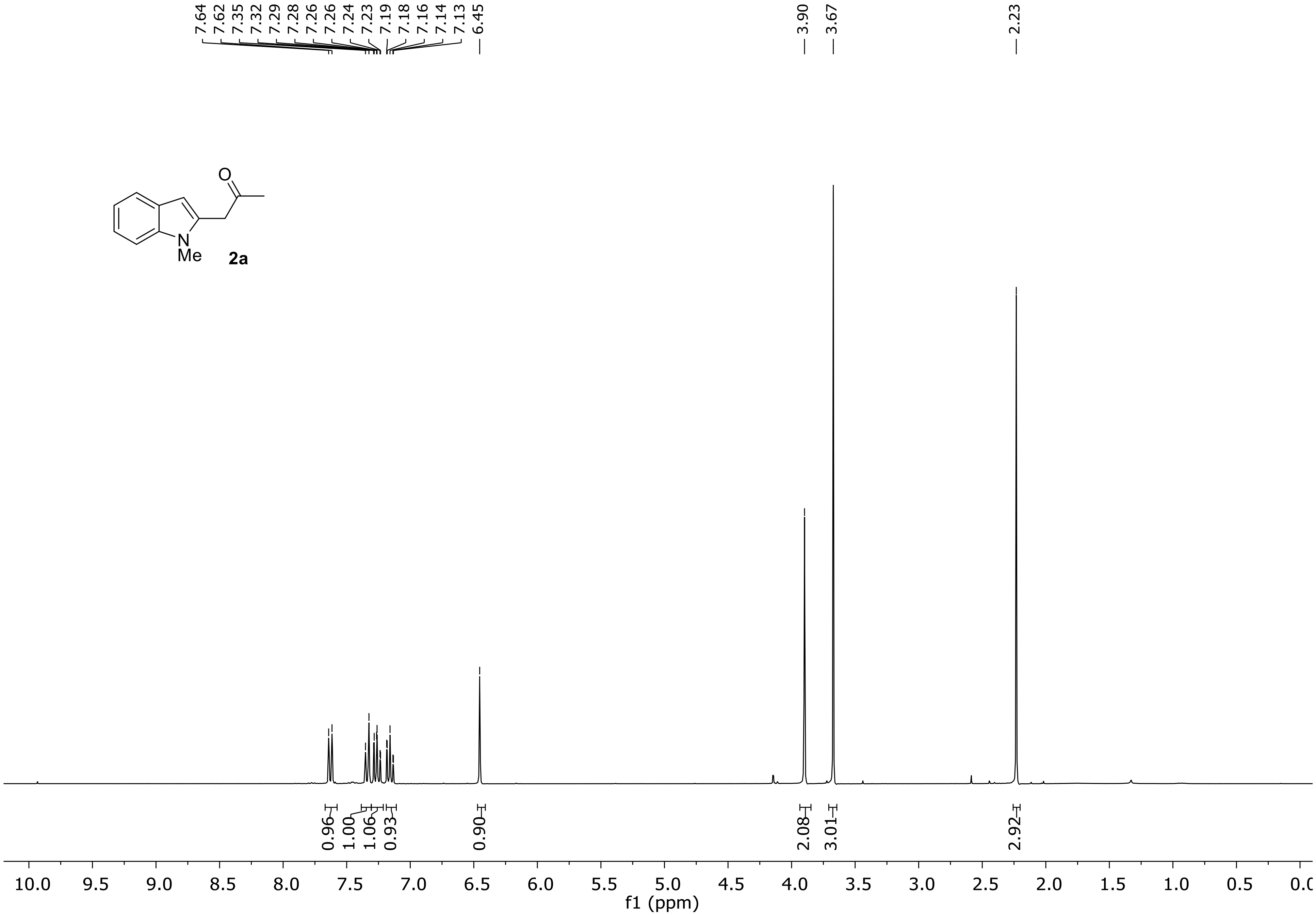

$^{13}C\{^1H\}$-NMR (300 MHz, $CDCl_3$)

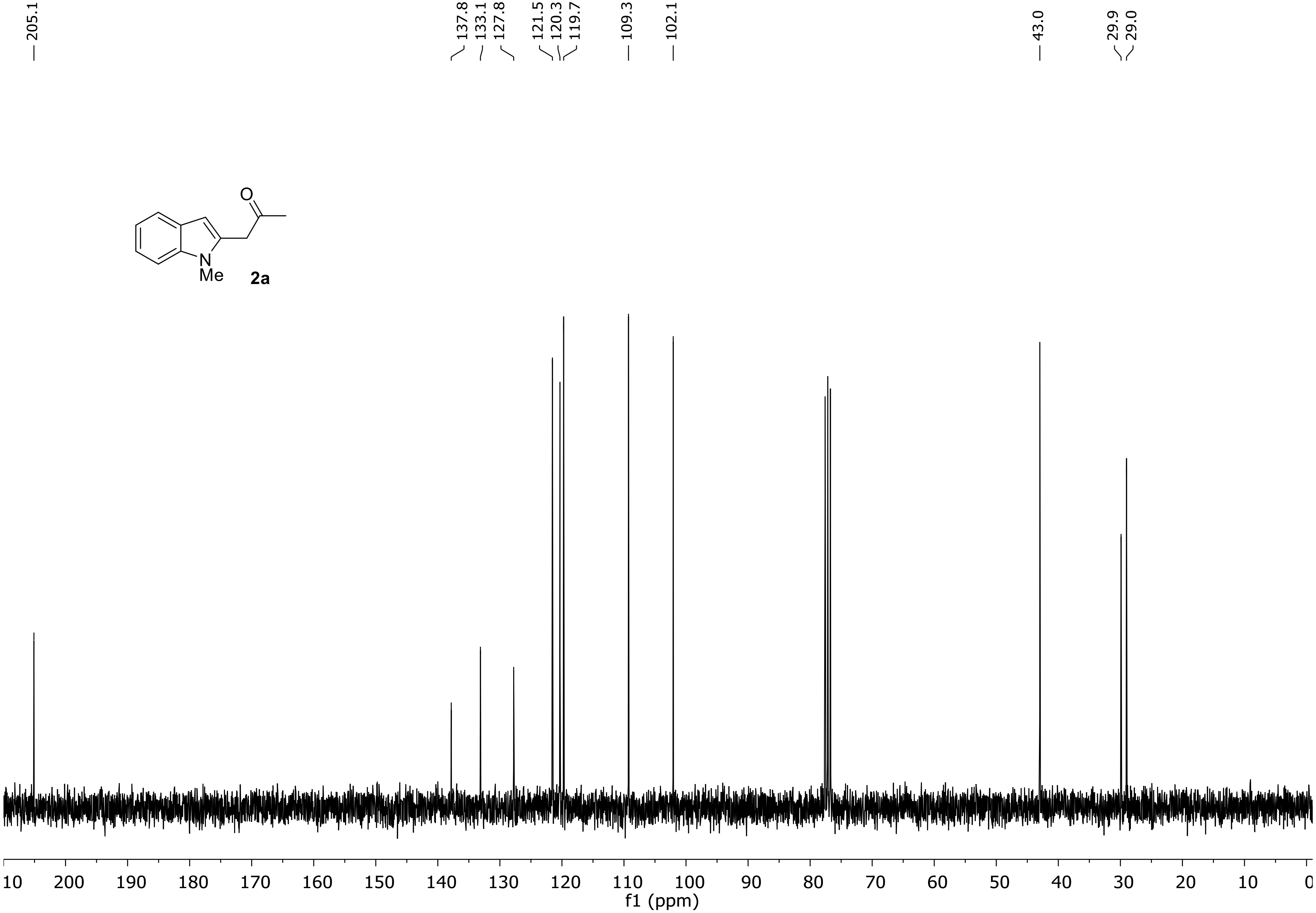

^{1}H-NMR (75.4 MHz, $CDCl_3$)

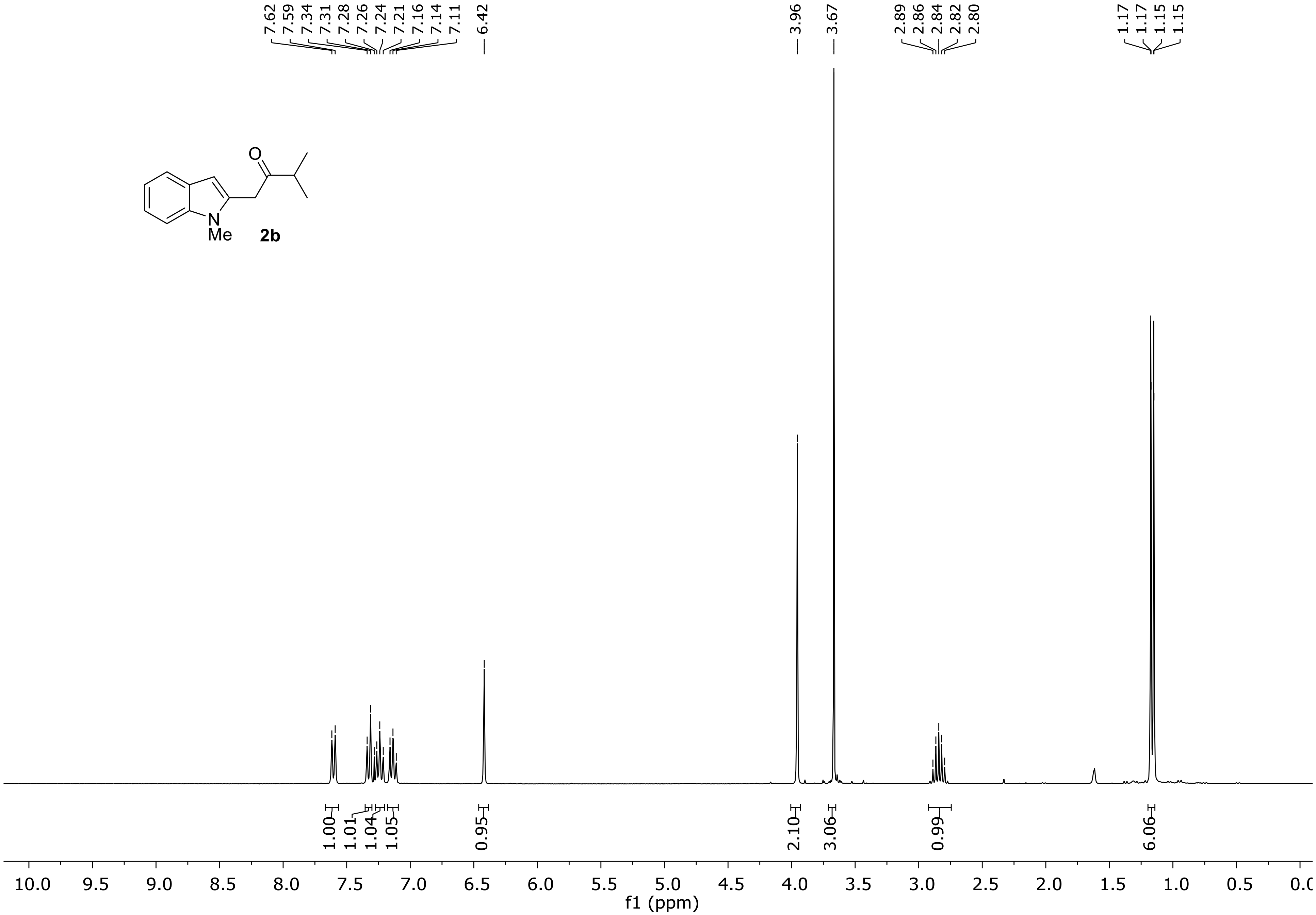

$^{13}C\{^{1}H\}$-NMR (300 MHz, $CDCl_3$)

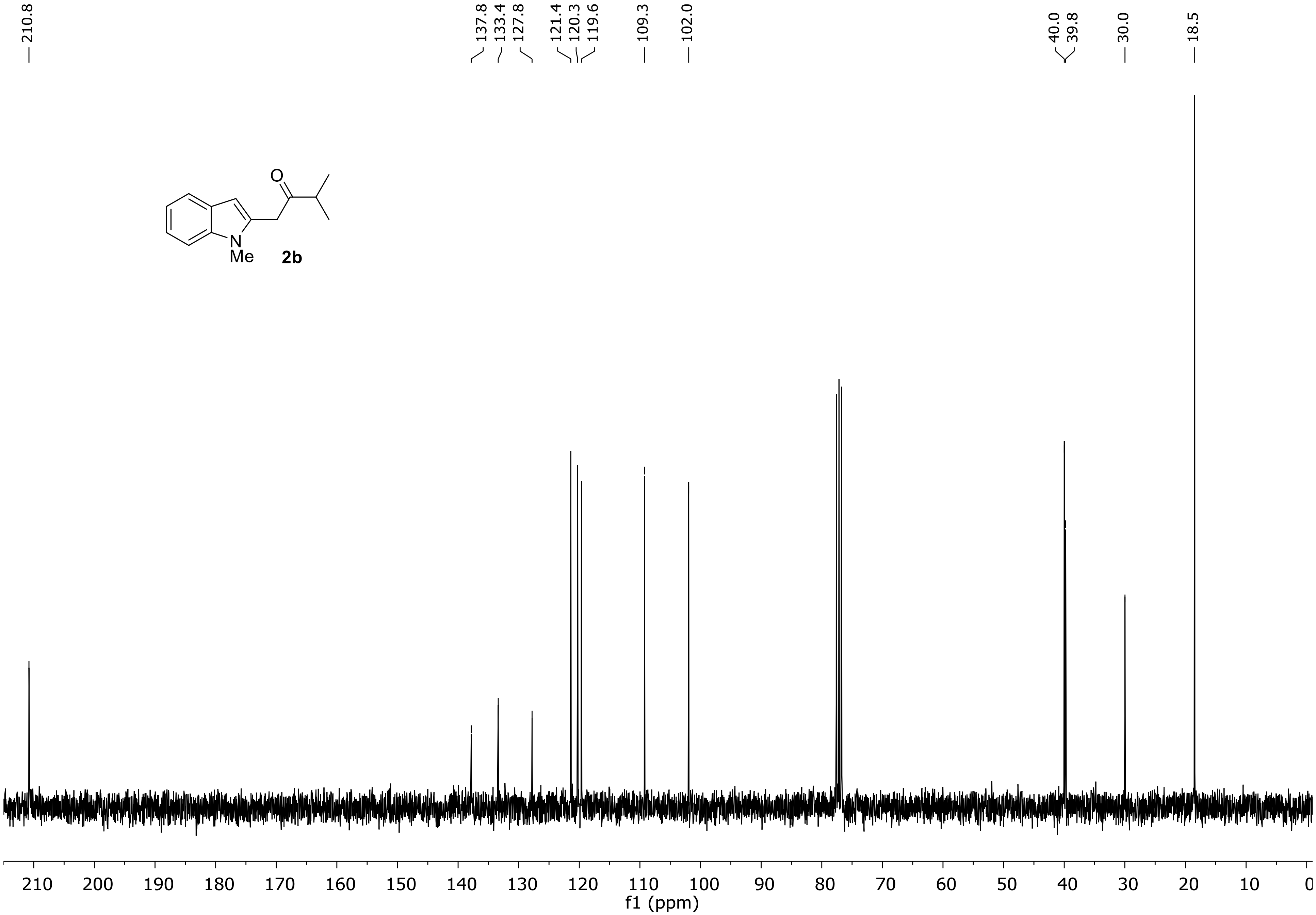

^{1}H-NMR (75.4 MHz, $CDCl_3$)

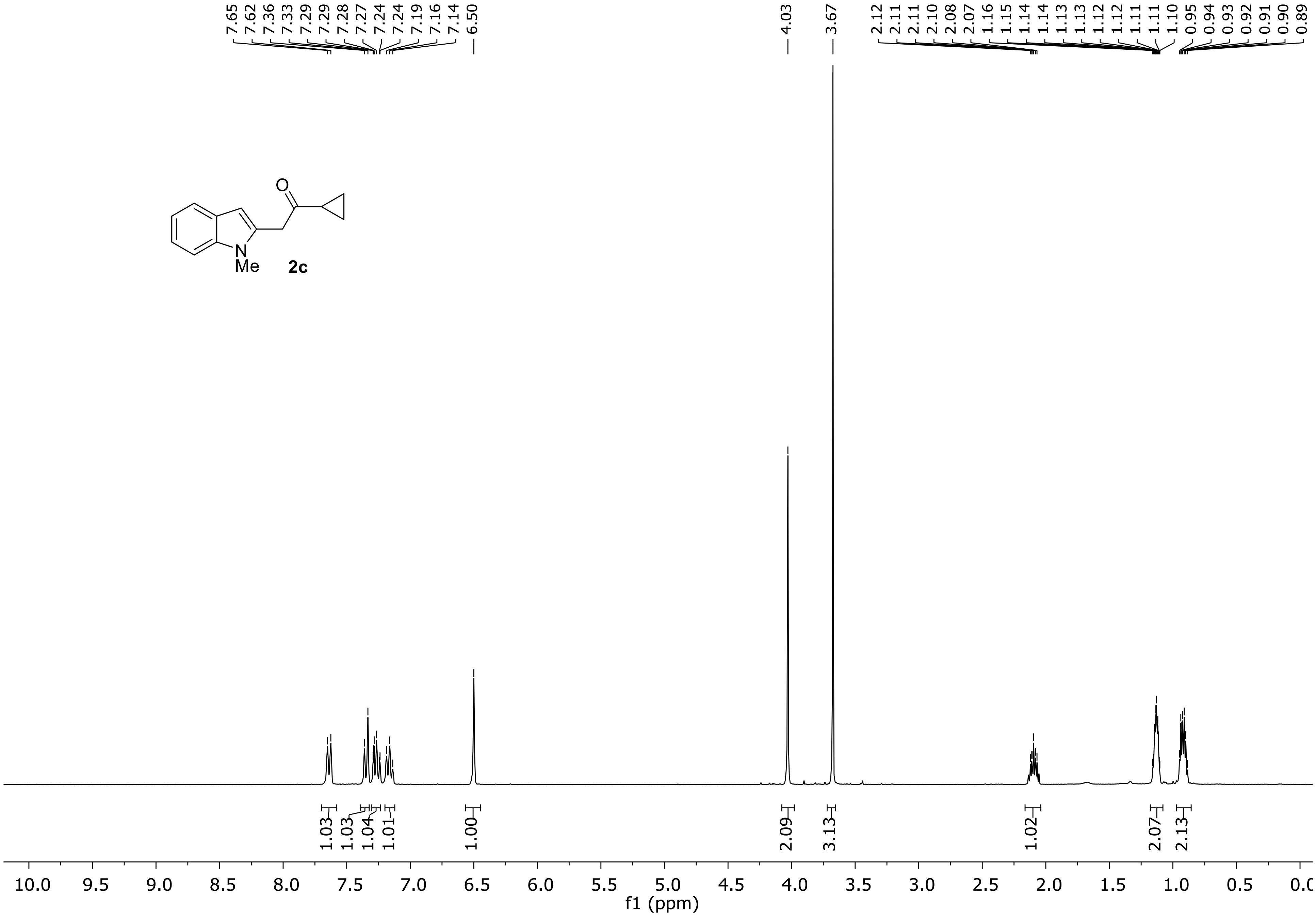

$^{13}C\{^{1}H\}$-NMR (300 MHz, $CDCl_3$)

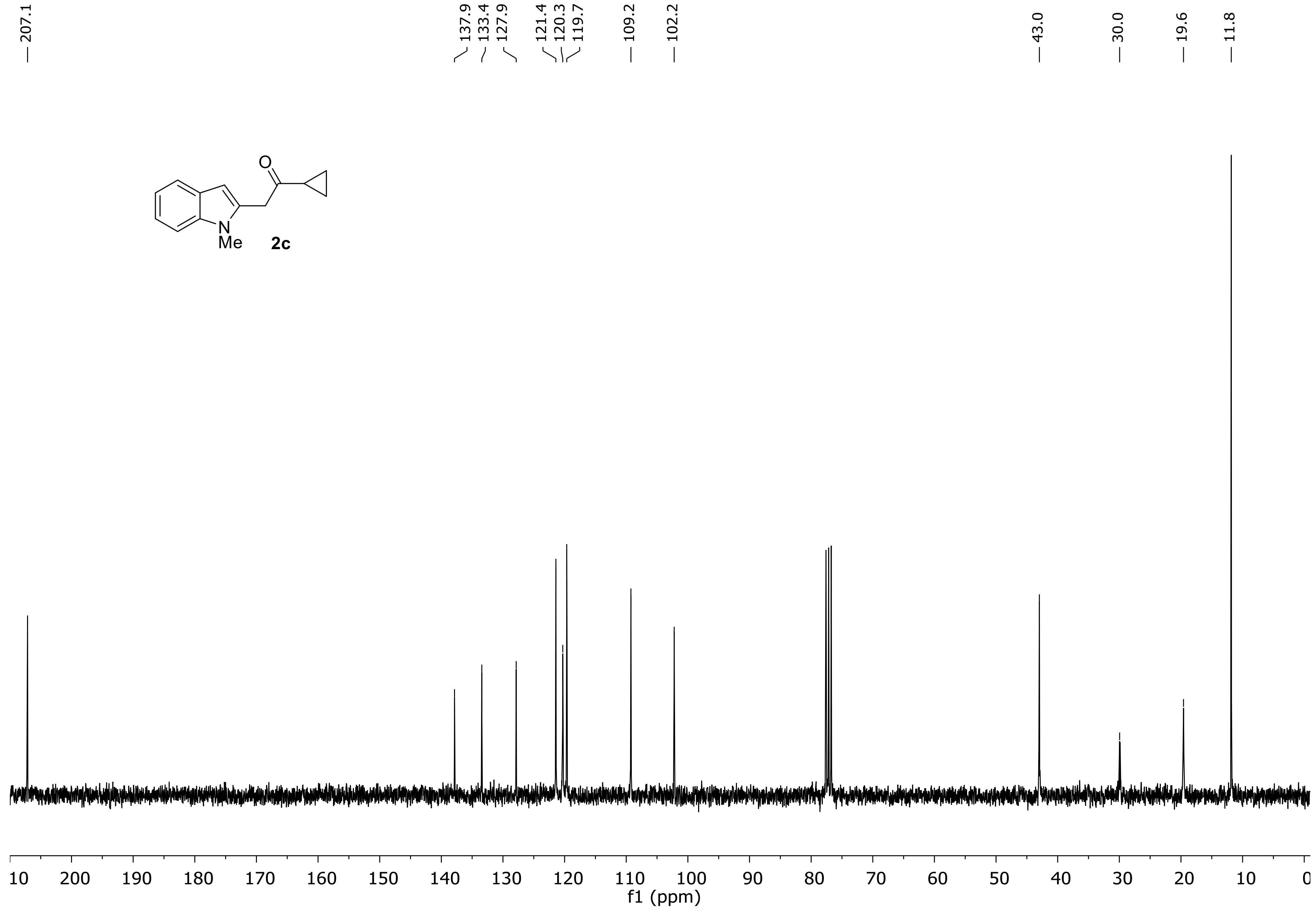

^{1}H-NMR (75.4 MHz, $CDCl_3$)

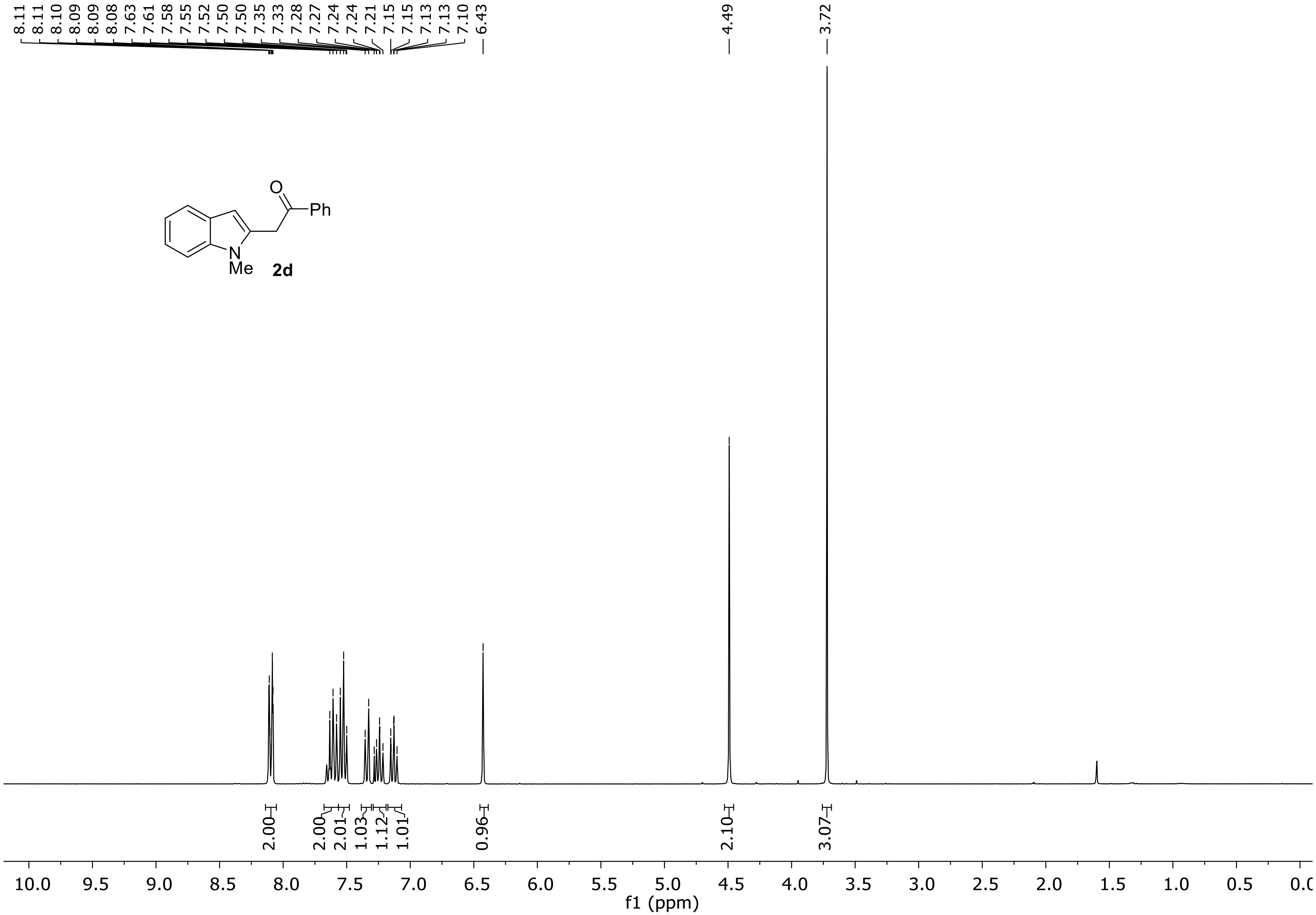

$^{13}C\{^{1}H\}$-NMR (300 MHz, $CDCl_3$)

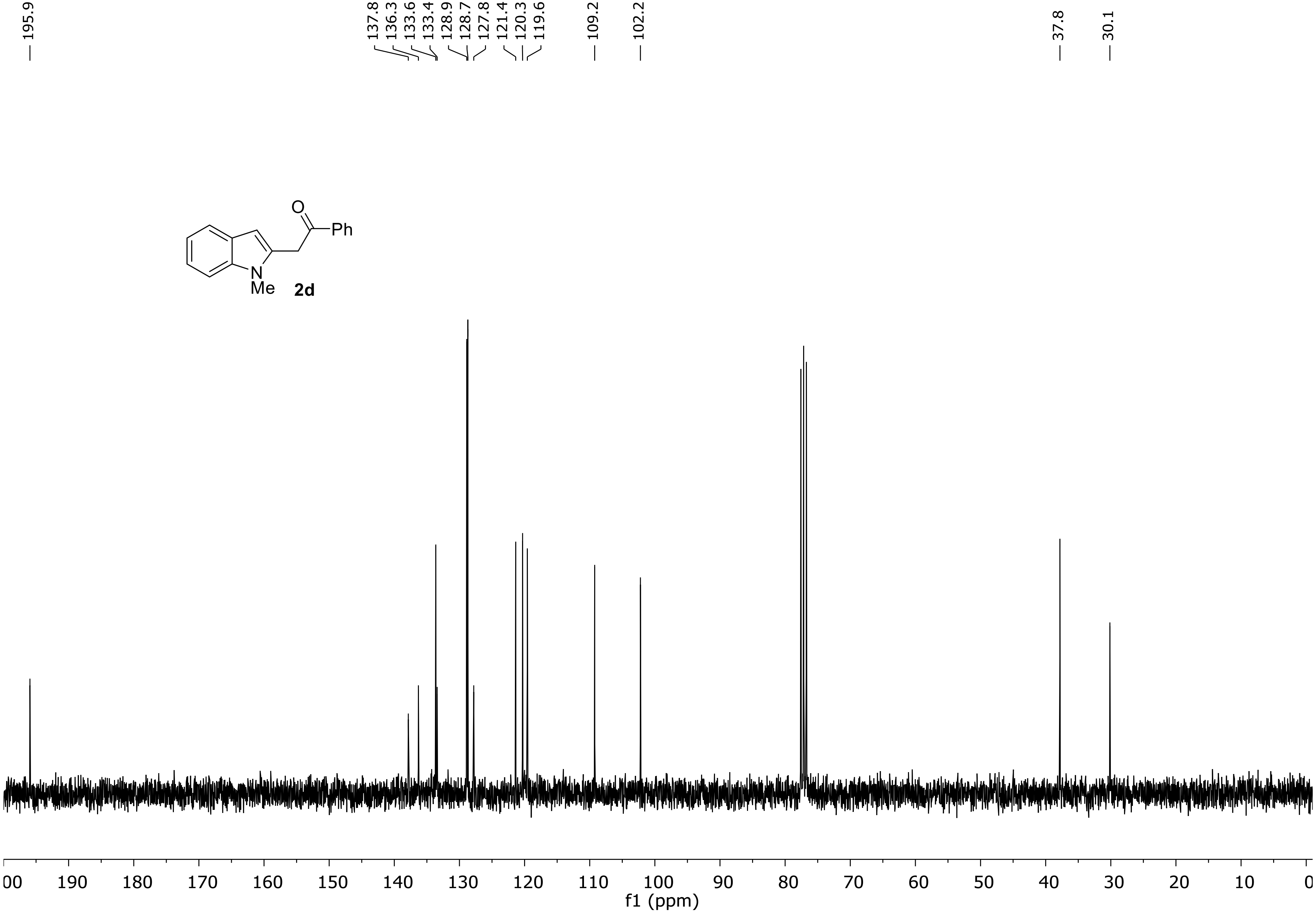

^{1}H-NMR (75.4 MHz, $CDCl_3$)

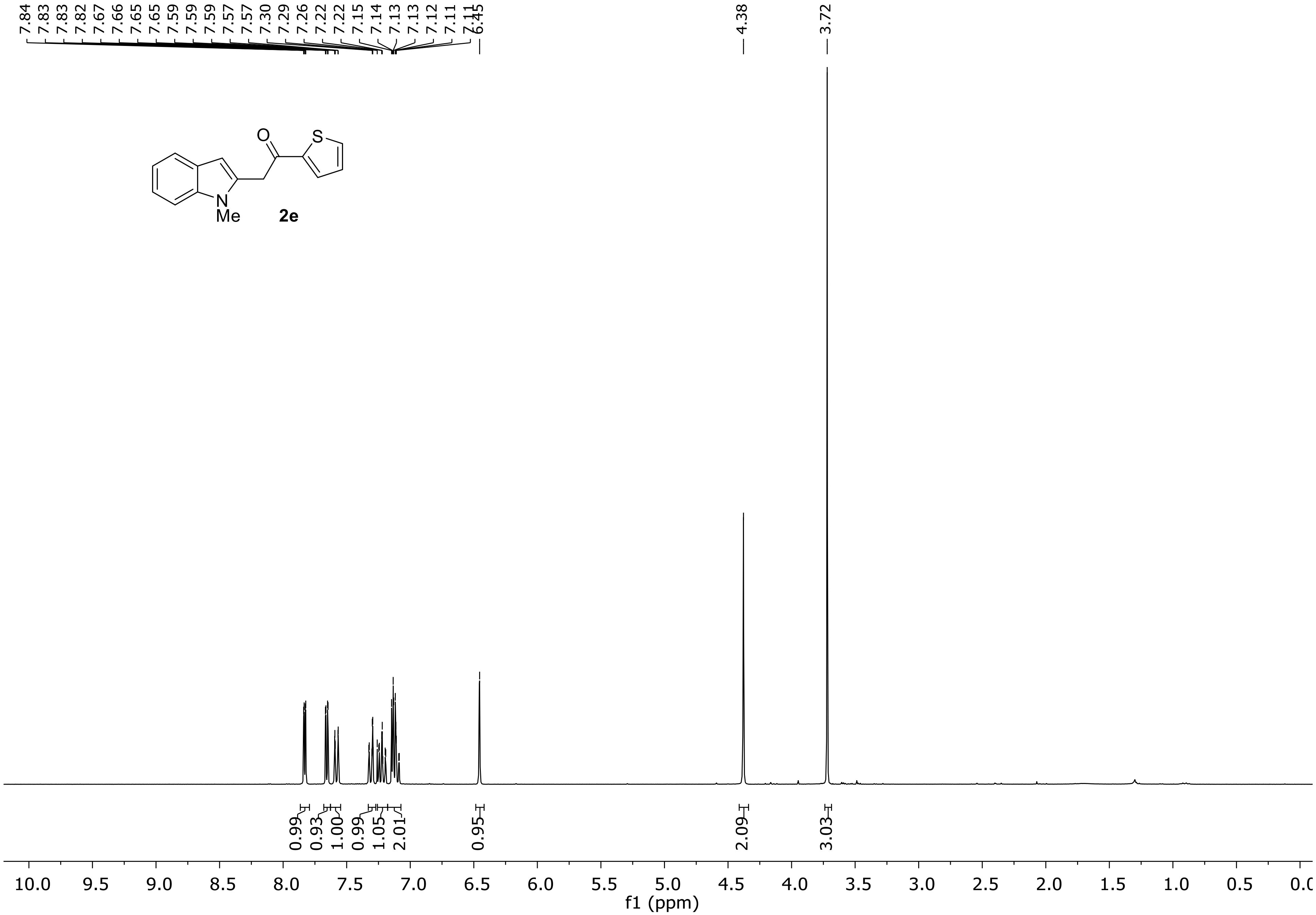

2e

$^{13}C\{^{1}H\}$-NMR (300 MHz, $CDCl_3$)

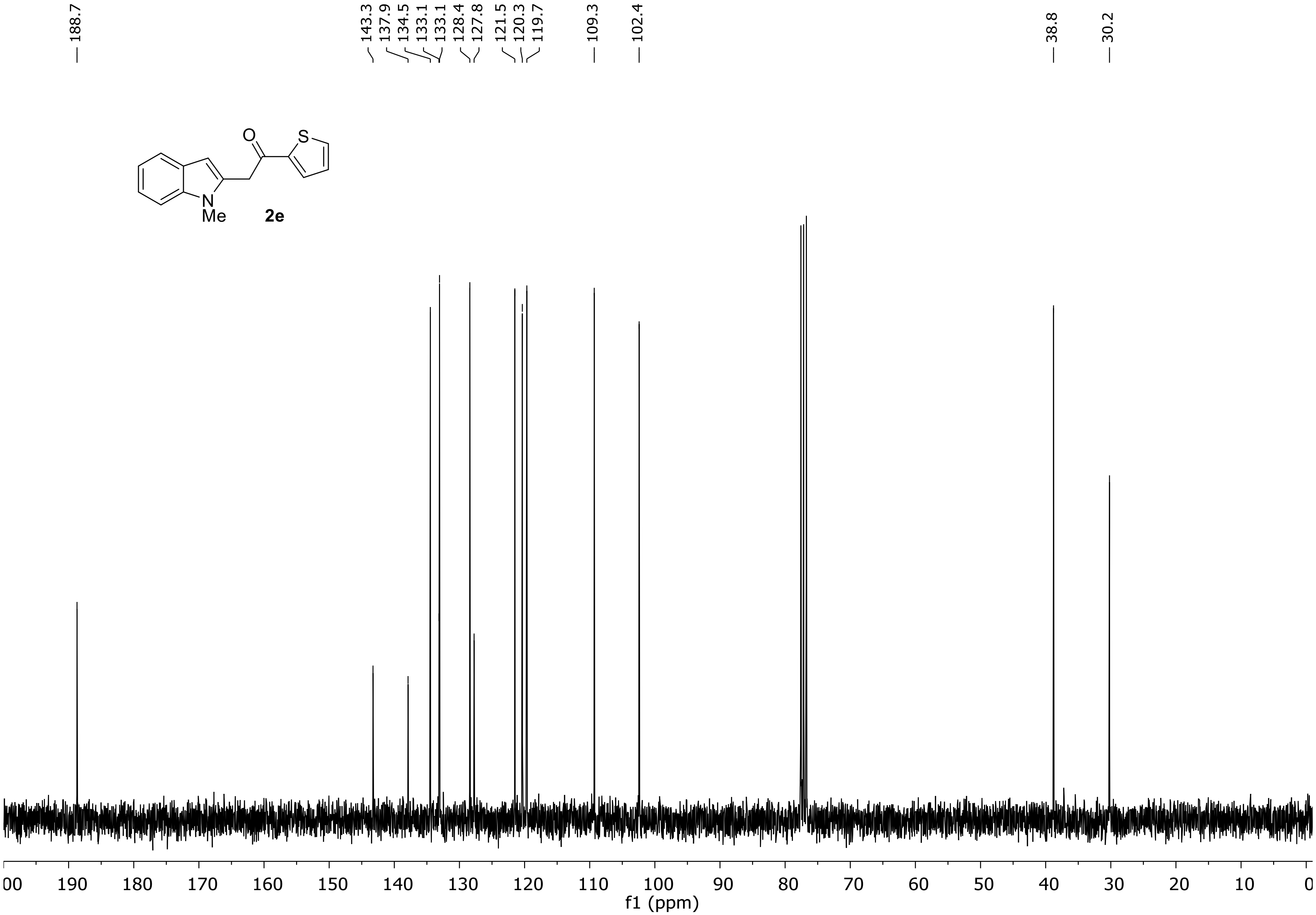

^{1}H-NMR (75.4 MHz, $CDCl_3$)

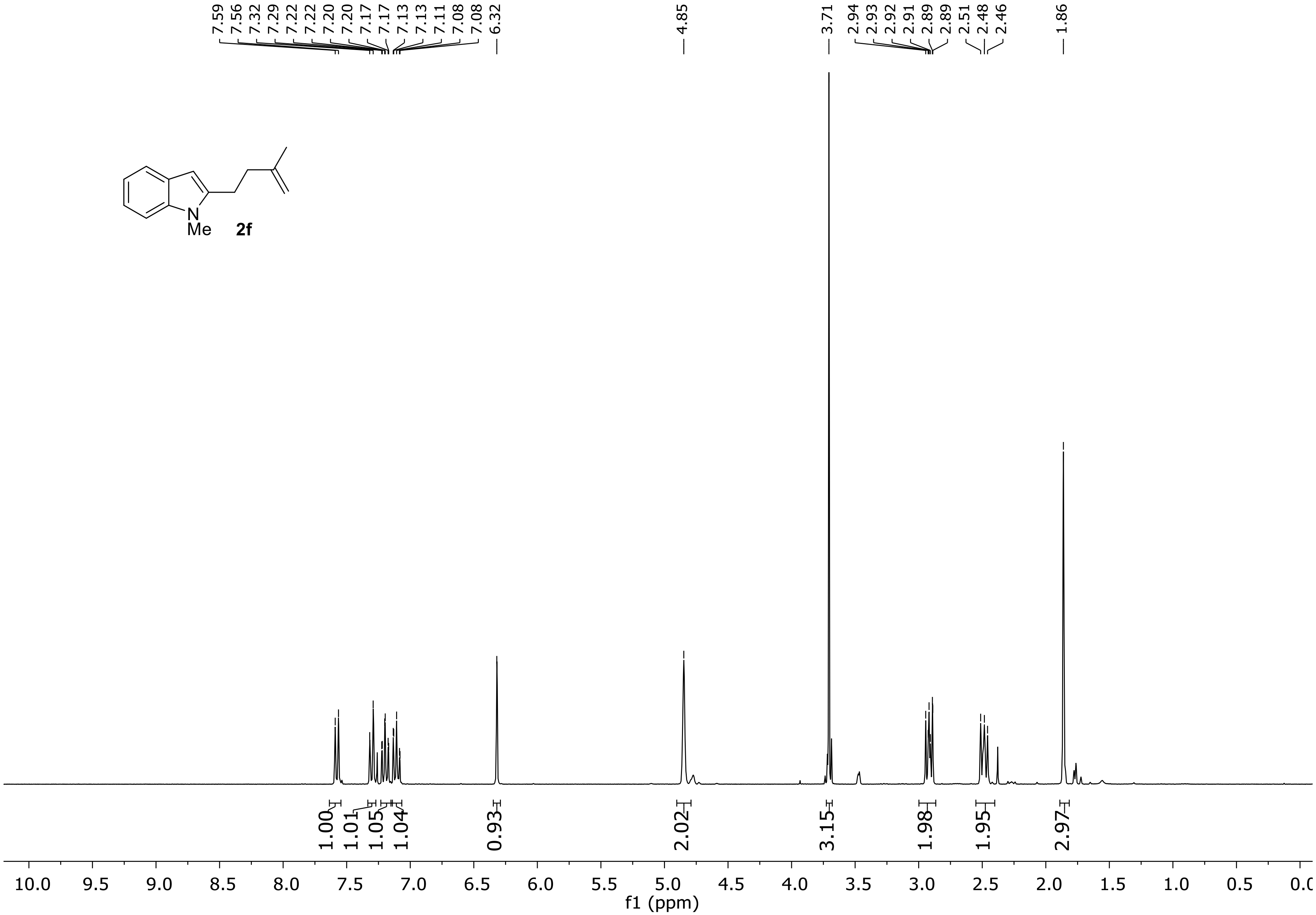
2f

$^{13}C\{^{1}H\}$-NMR (300 MHz, $CDCl_3$)

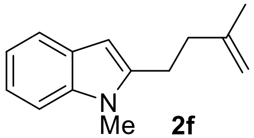

2f

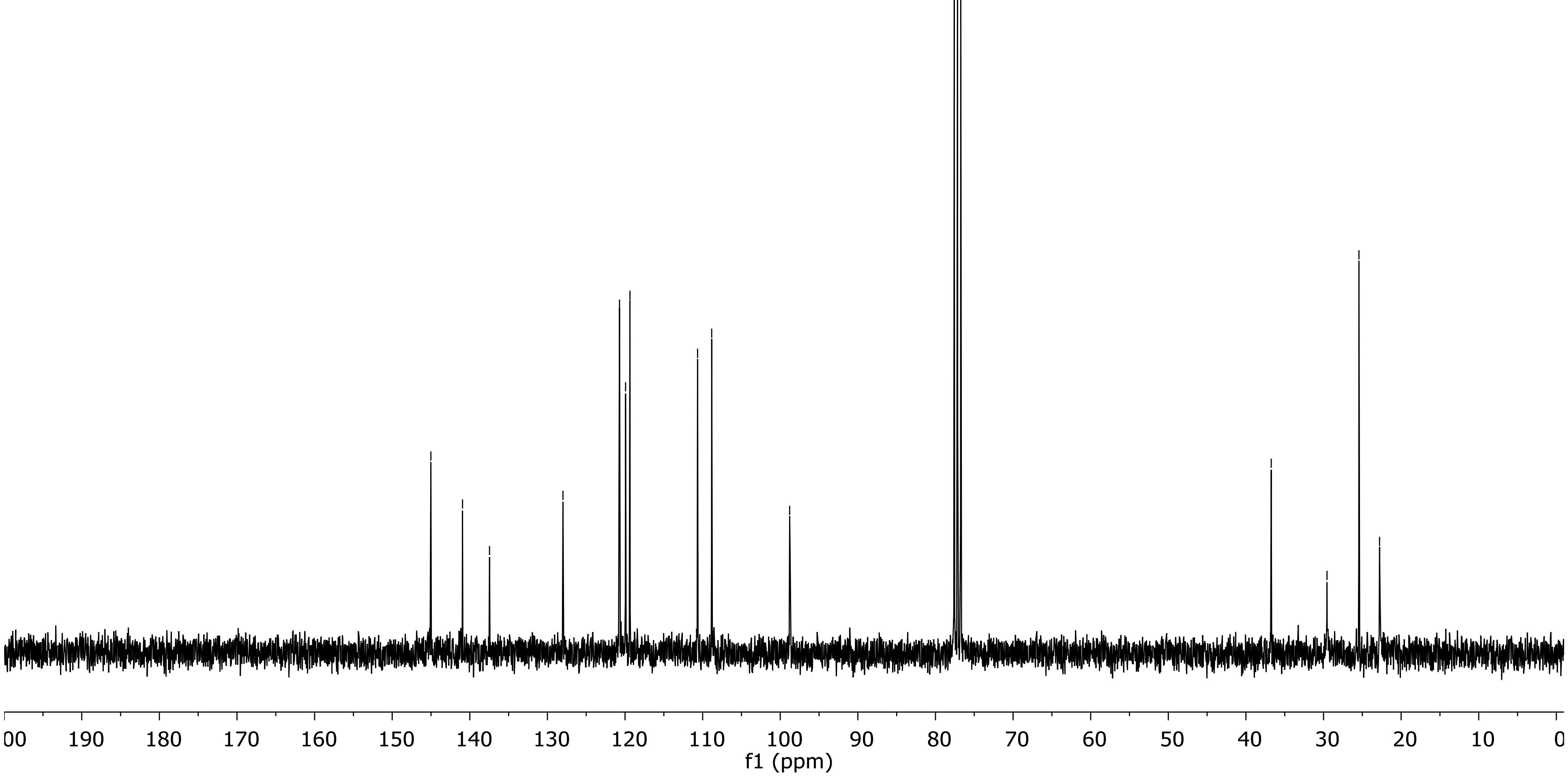

^{1}H-NMR (75.4 MHz, $CDCl_3$)

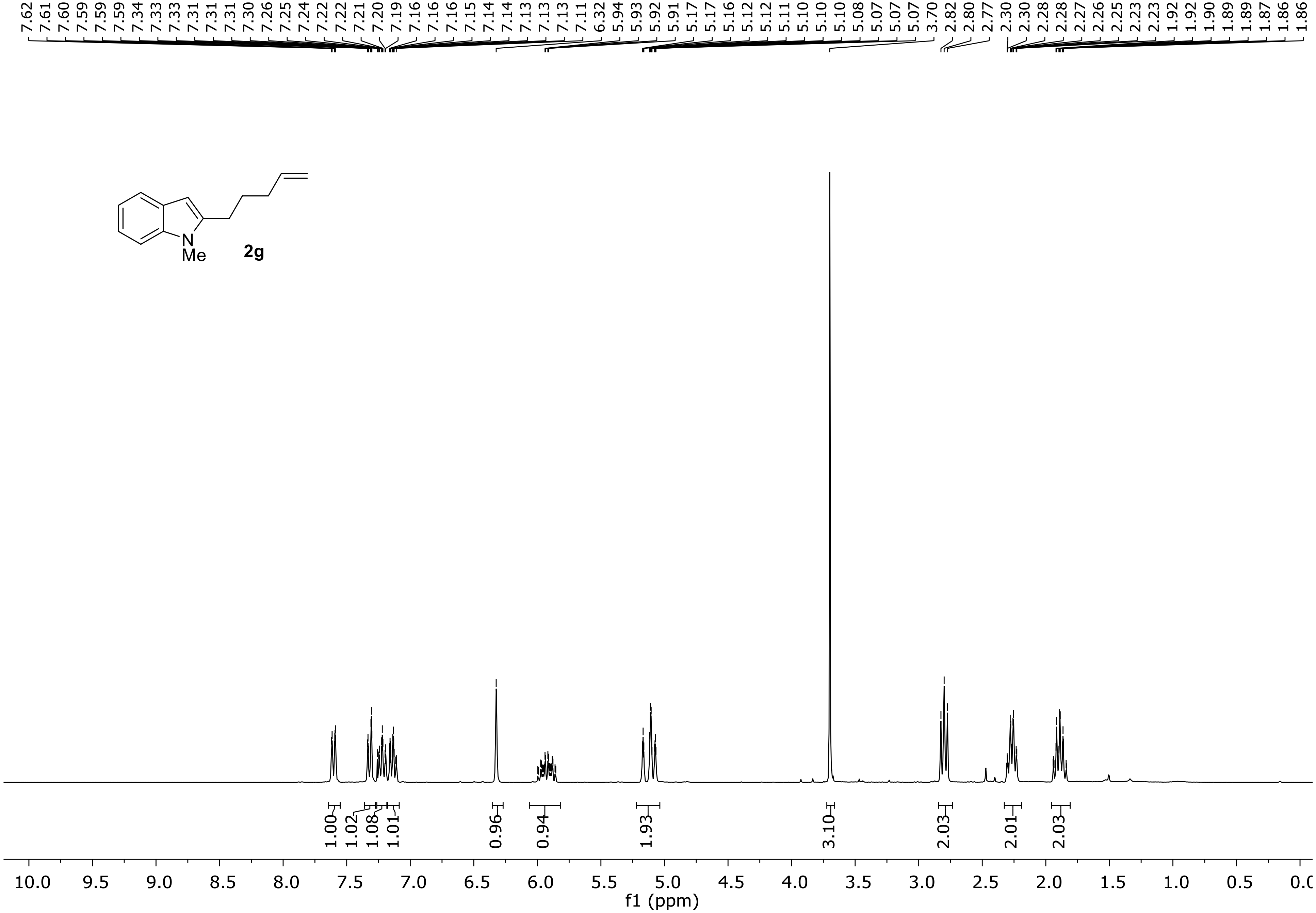

2g

$^{13}C\{^1H\}$-NMR (300 MHz, $CDCl_3$)

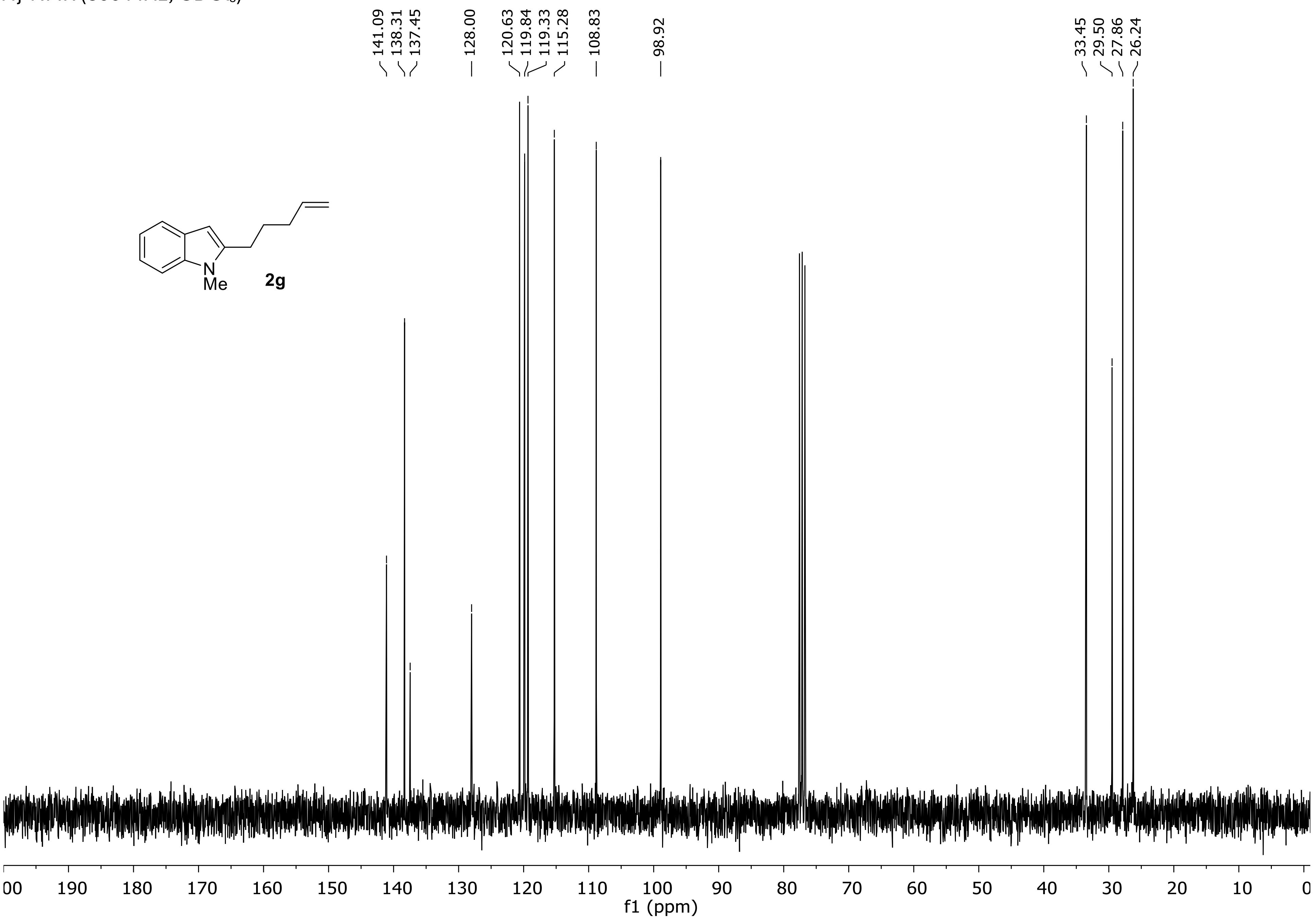

^{1}H-NMR (75.4 MHz, $CDCl_3$)

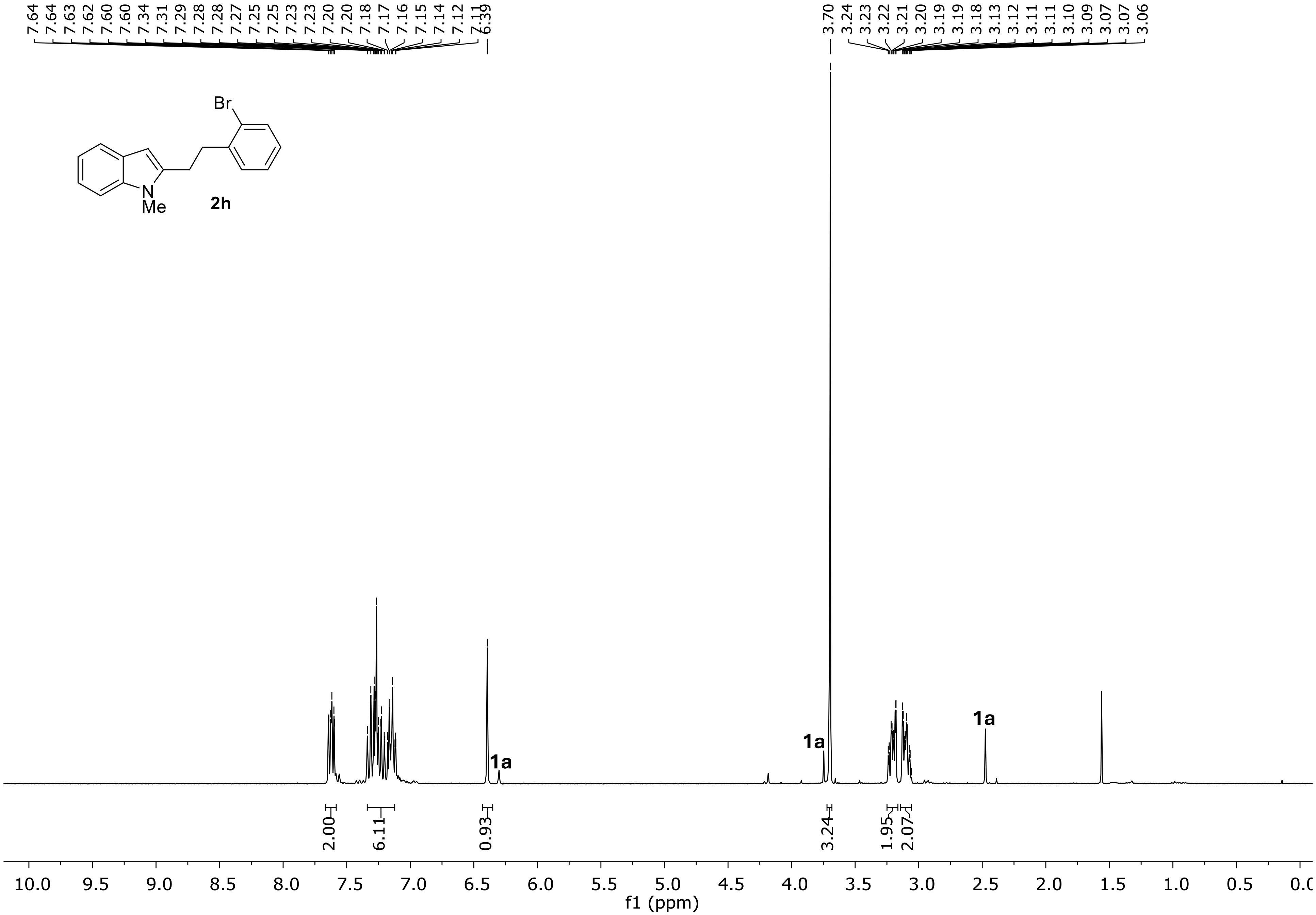

$^{13}C\{^{1}H\}$-NMR (300 MHz, $CDCl_3$)

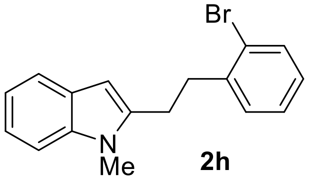

2h

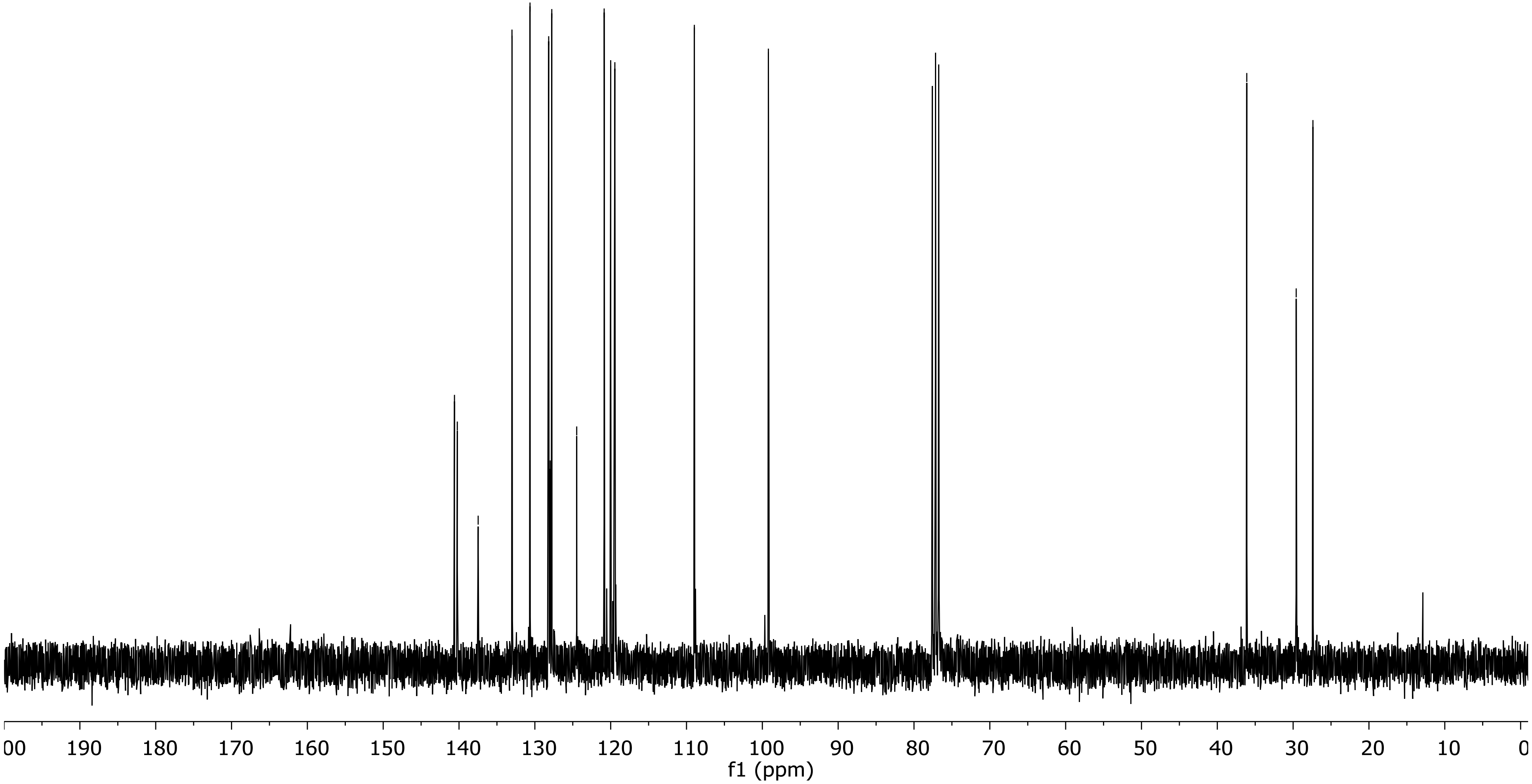

^{1}H-NMR (75.4 MHz, $CDCl_3$)

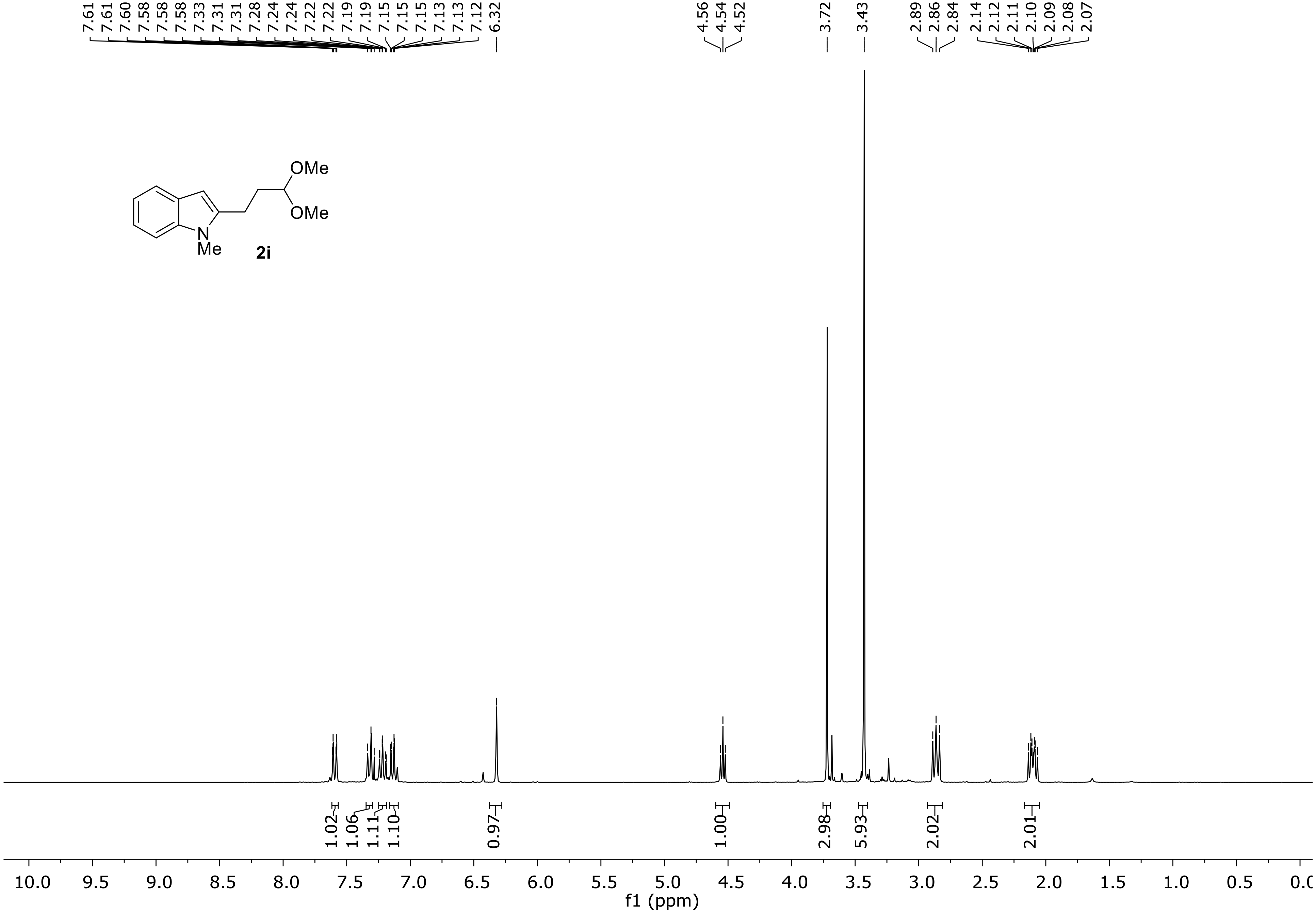

$^{13}C\{^1H\}$-NMR (300 MHz, $CDCl_3$)

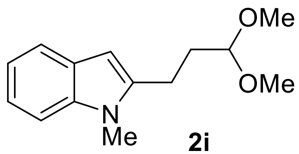

2i

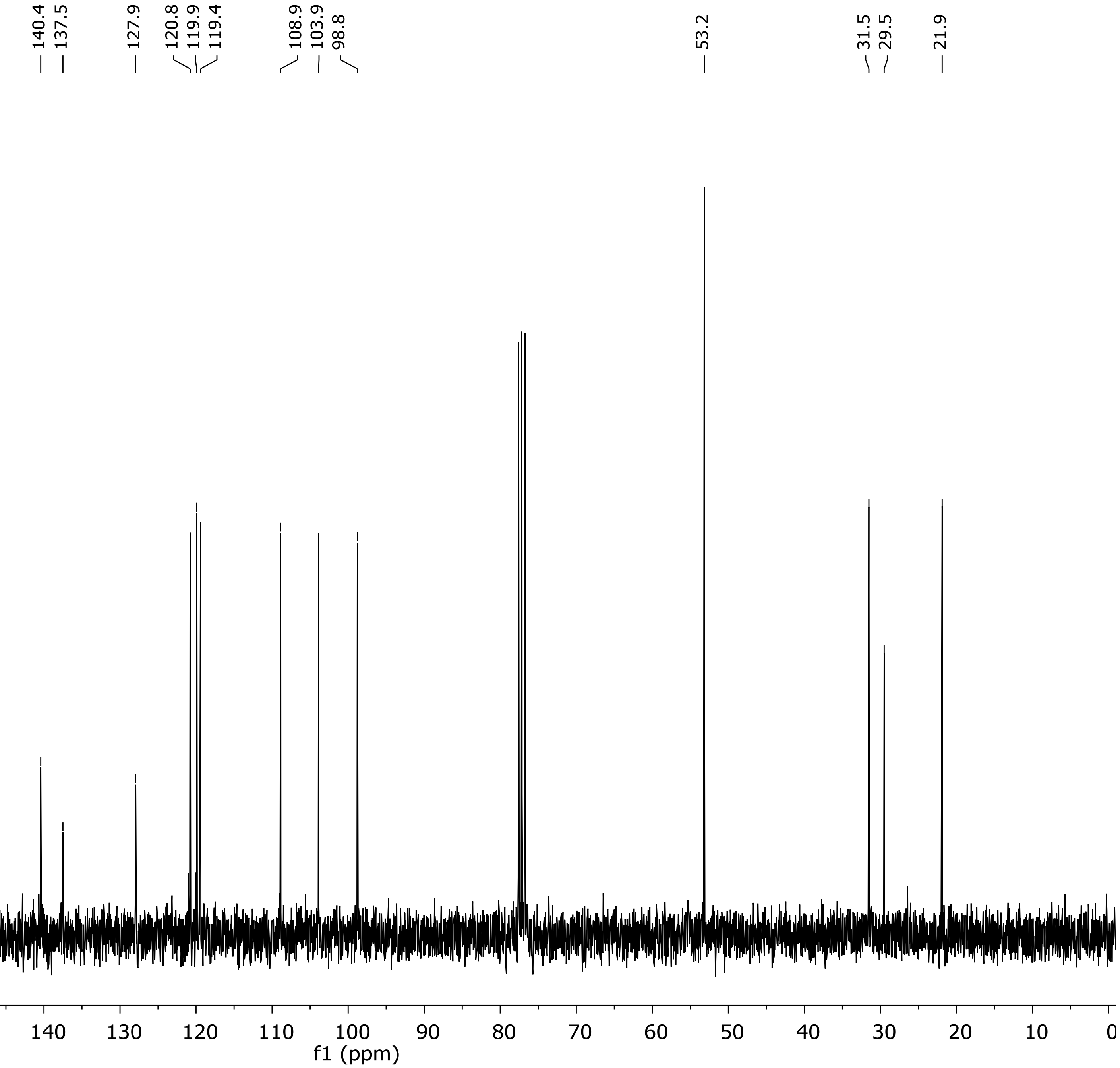

^{1}H-NMR (75.4 MHz, $CDCl_3$)

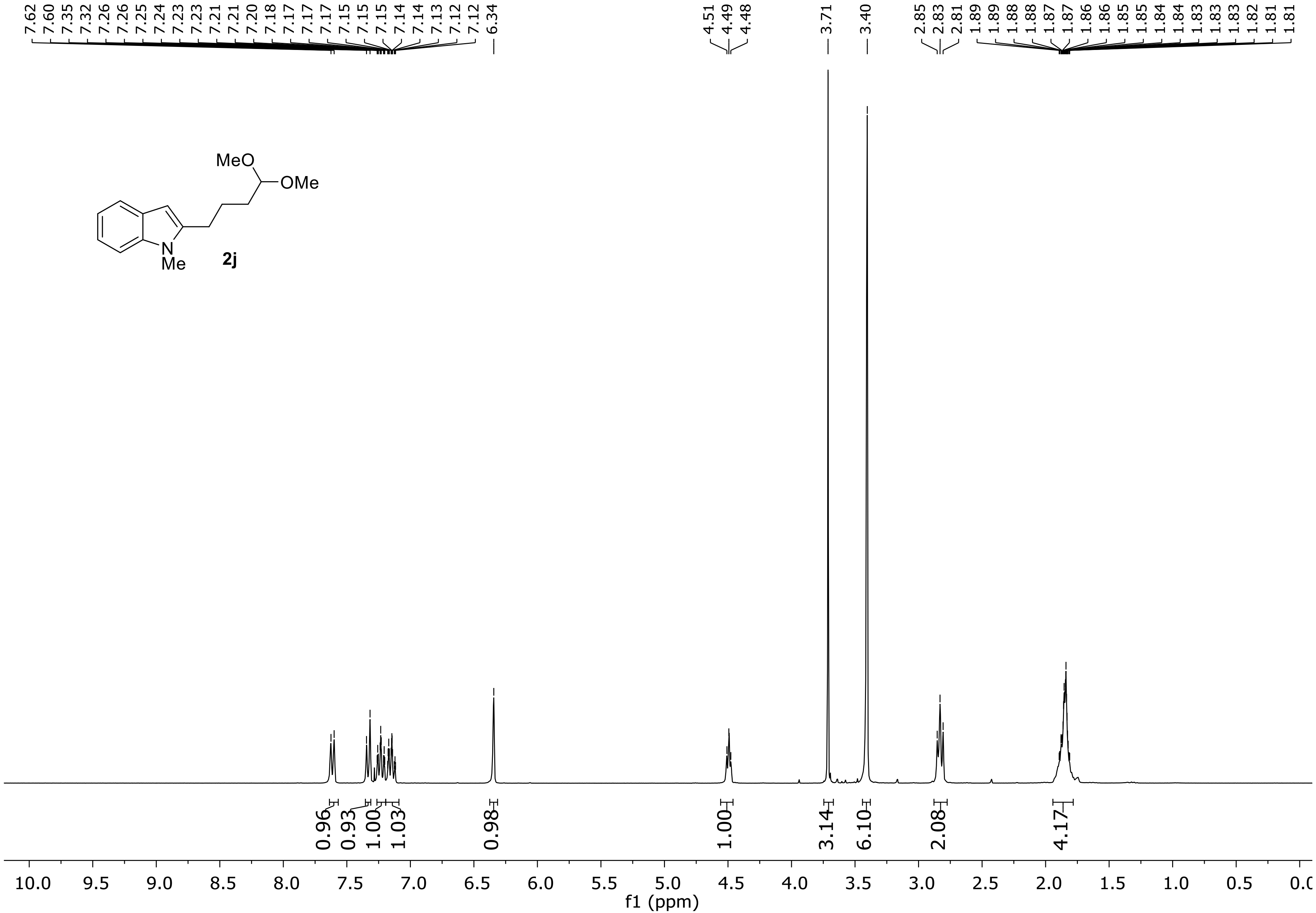

2j

$^{13}C\{^{1}H\}$-NMR (300 MHz, $CDCl_3$)

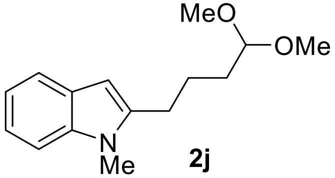

2j

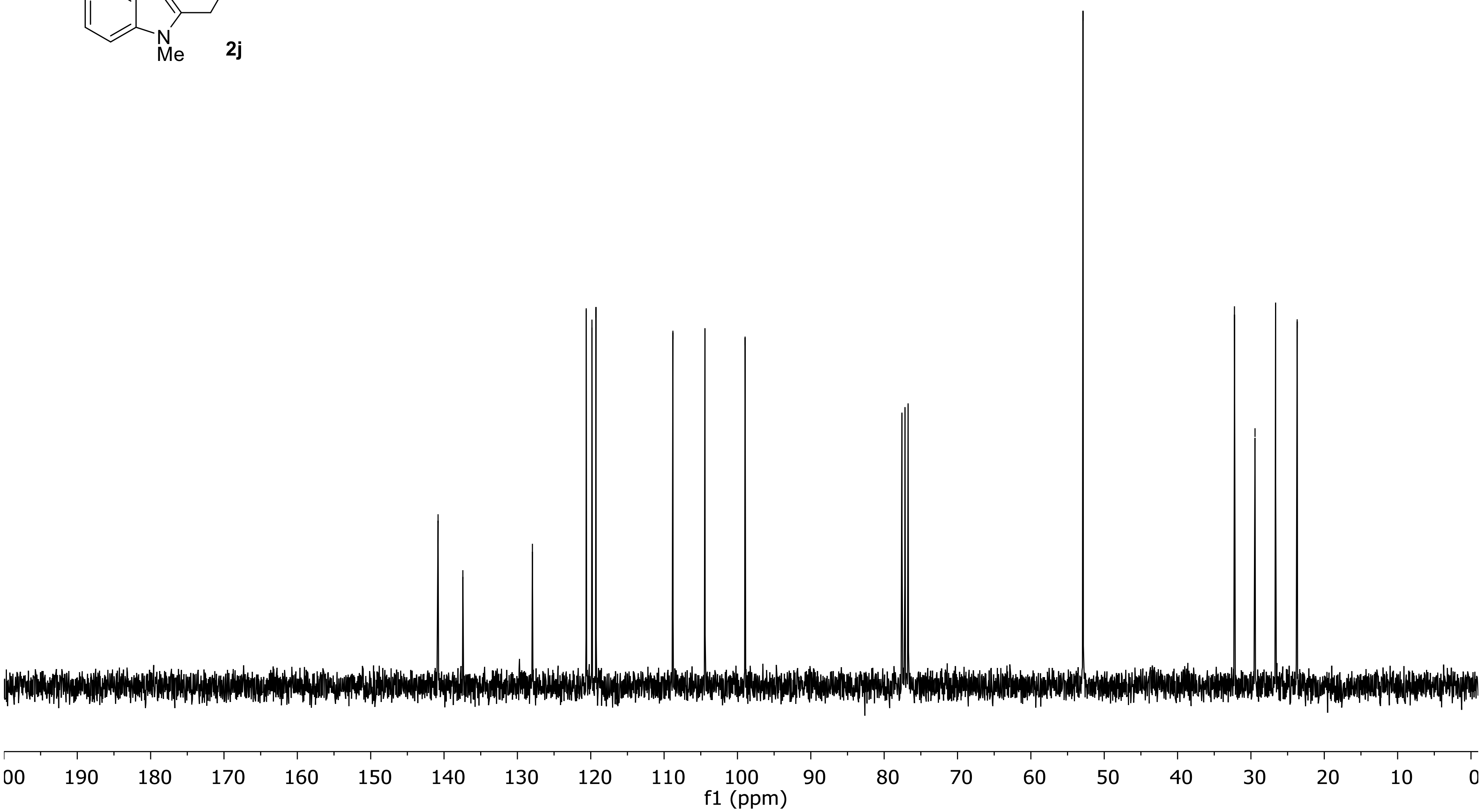

^{1}H-NMR (75.4 MHz, $CDCl_3$)

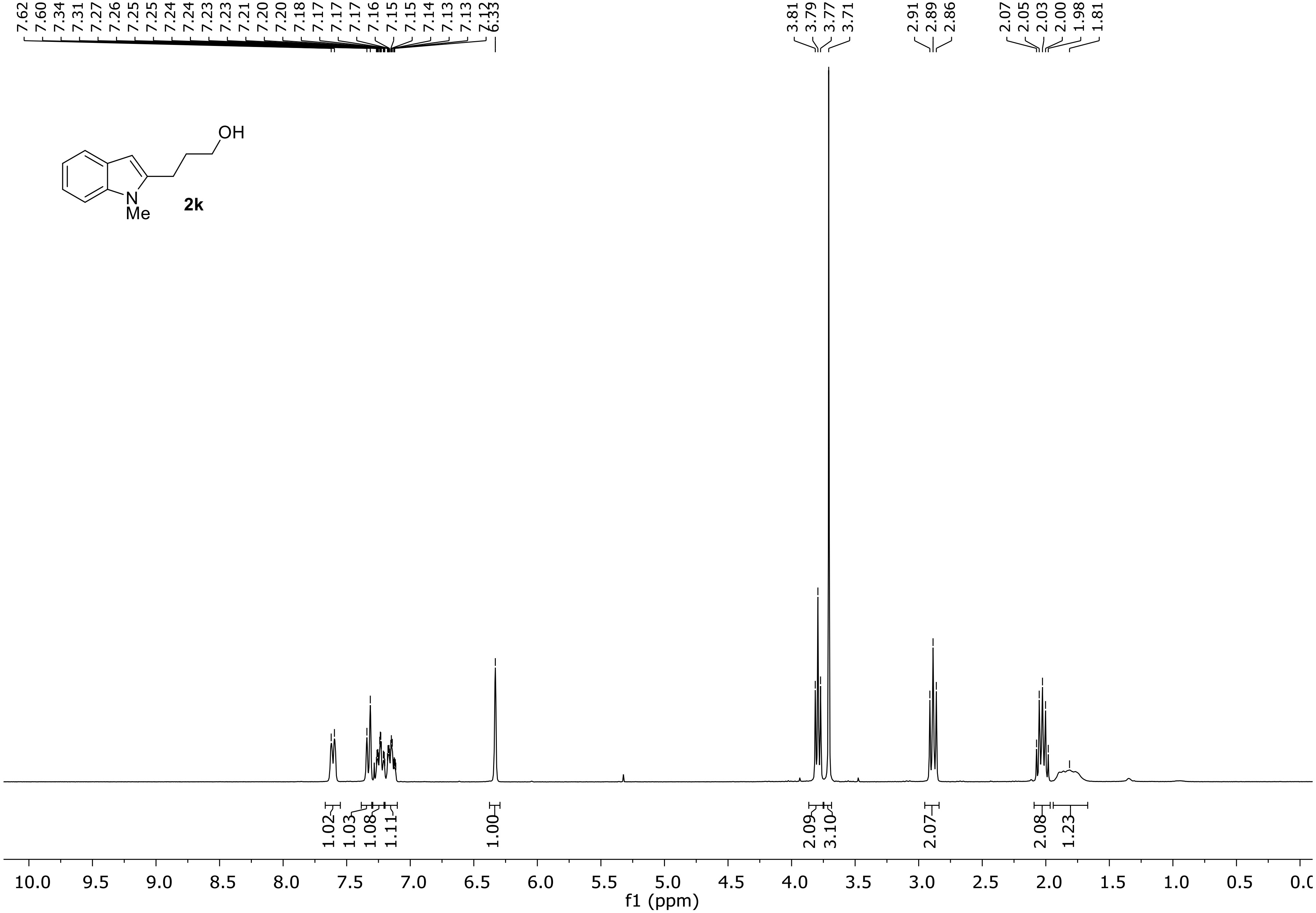

2k

$^{13}C\{^{1}H\}$-NMR (300 MHz, $CDCl_3$)

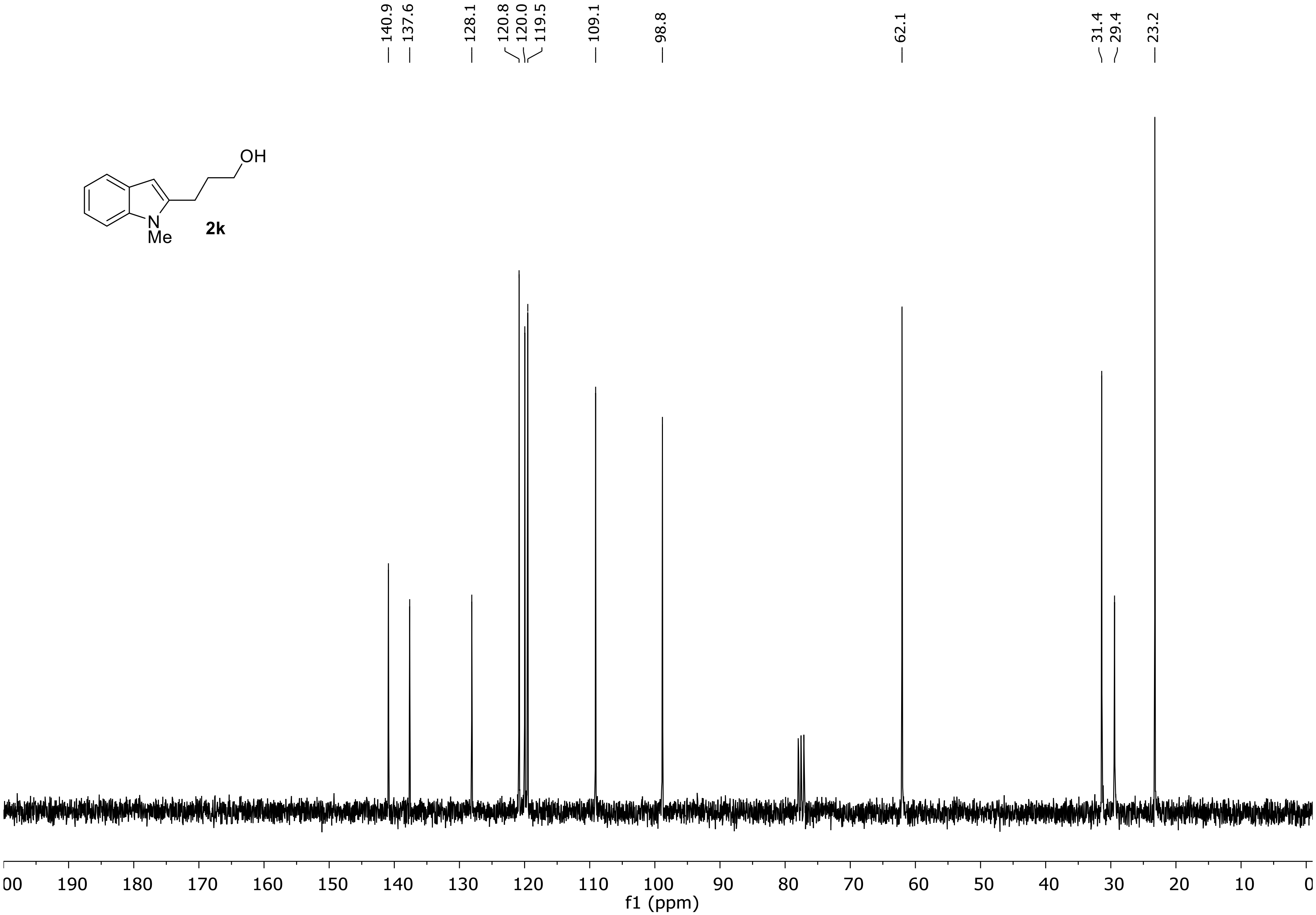

^{1}H-NMR (126 MHz, $CDCl_3$)

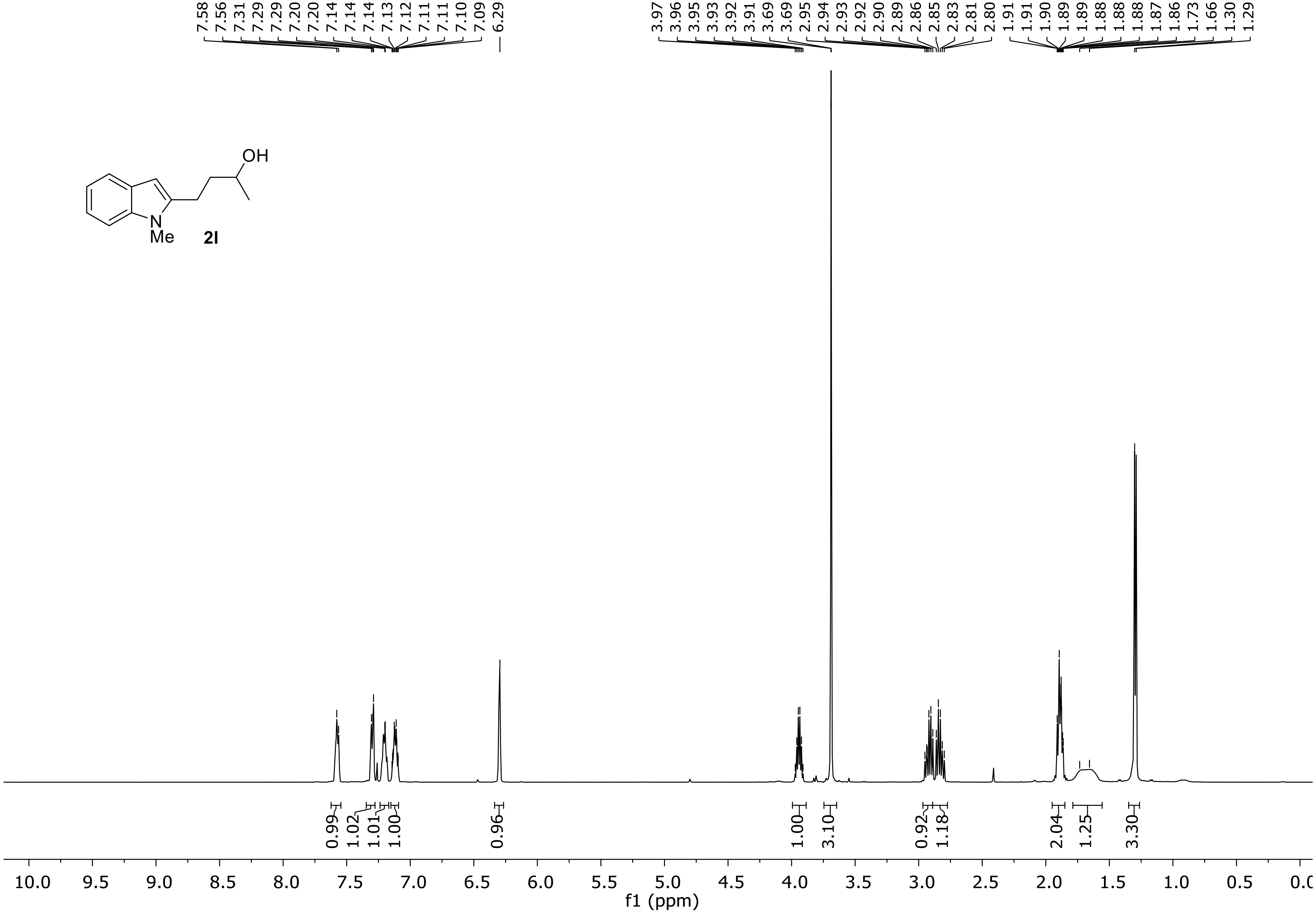

$^{13}C\{^1H\}$-NMR (500 MHz, $CDCl_3$)

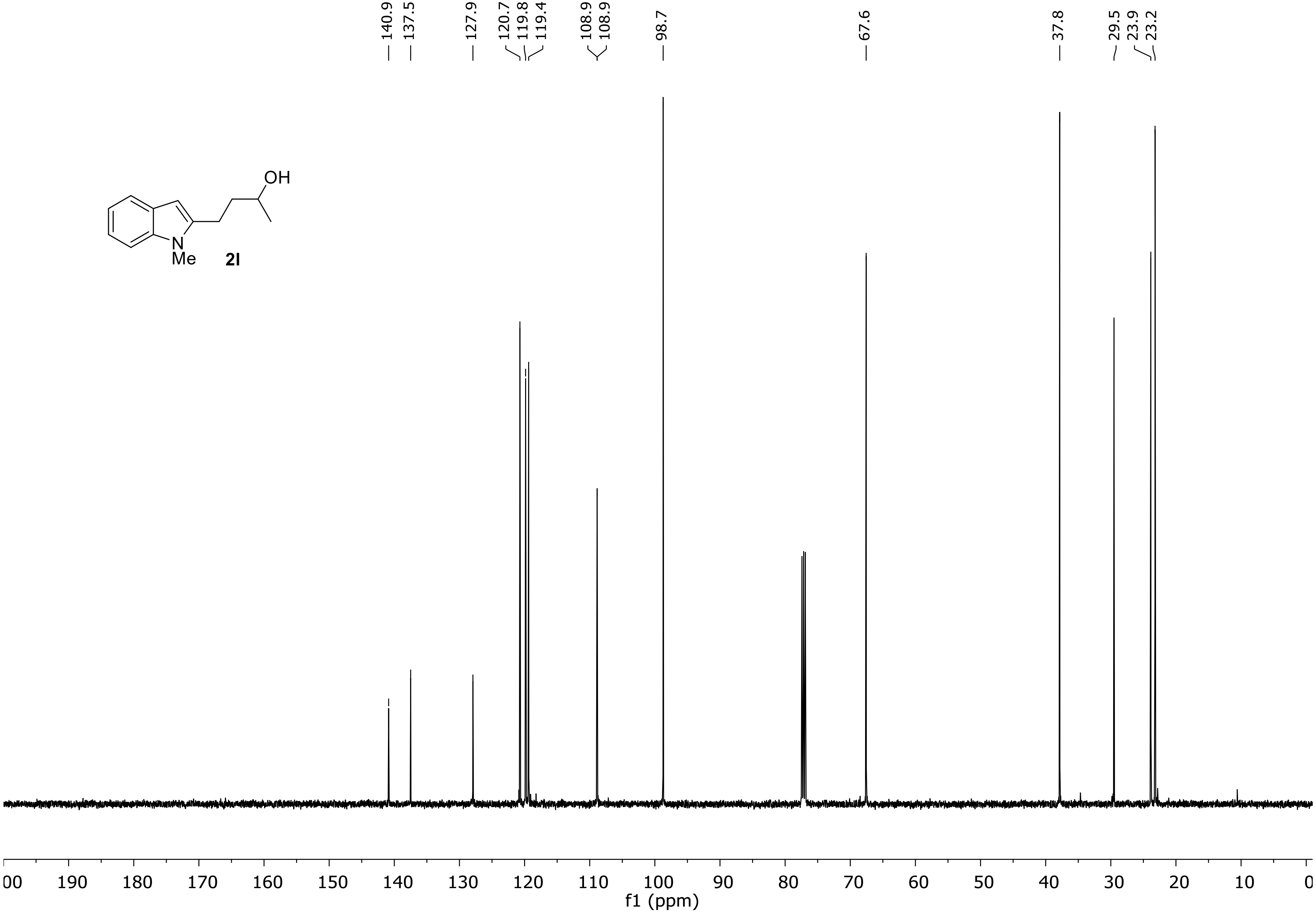

^{1}H-NMR (75.4 MHz, $CDCl_3$)

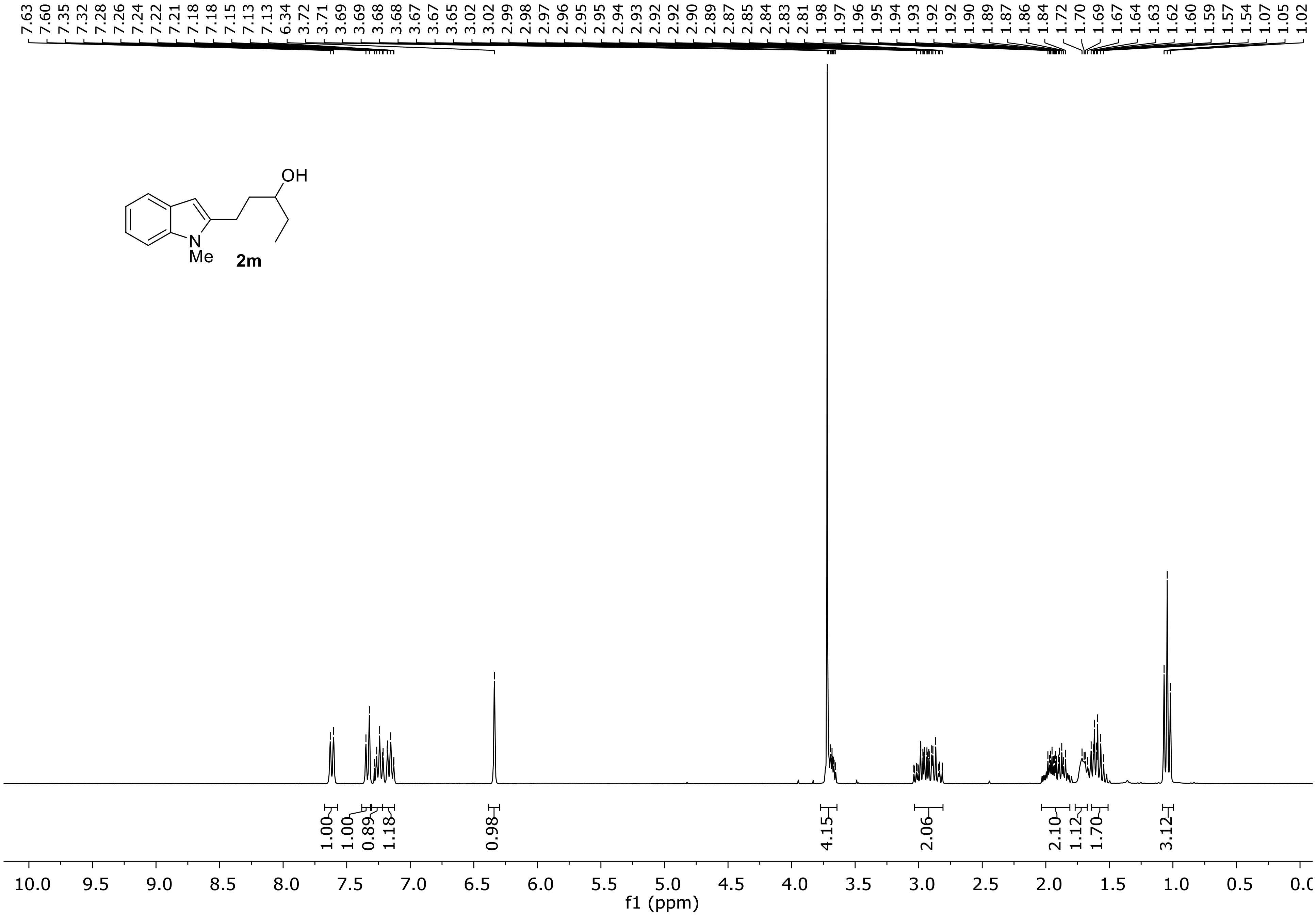

$^{13}C\{^{1}H\}$-NMR (300 MHz, $CDCl_3$)

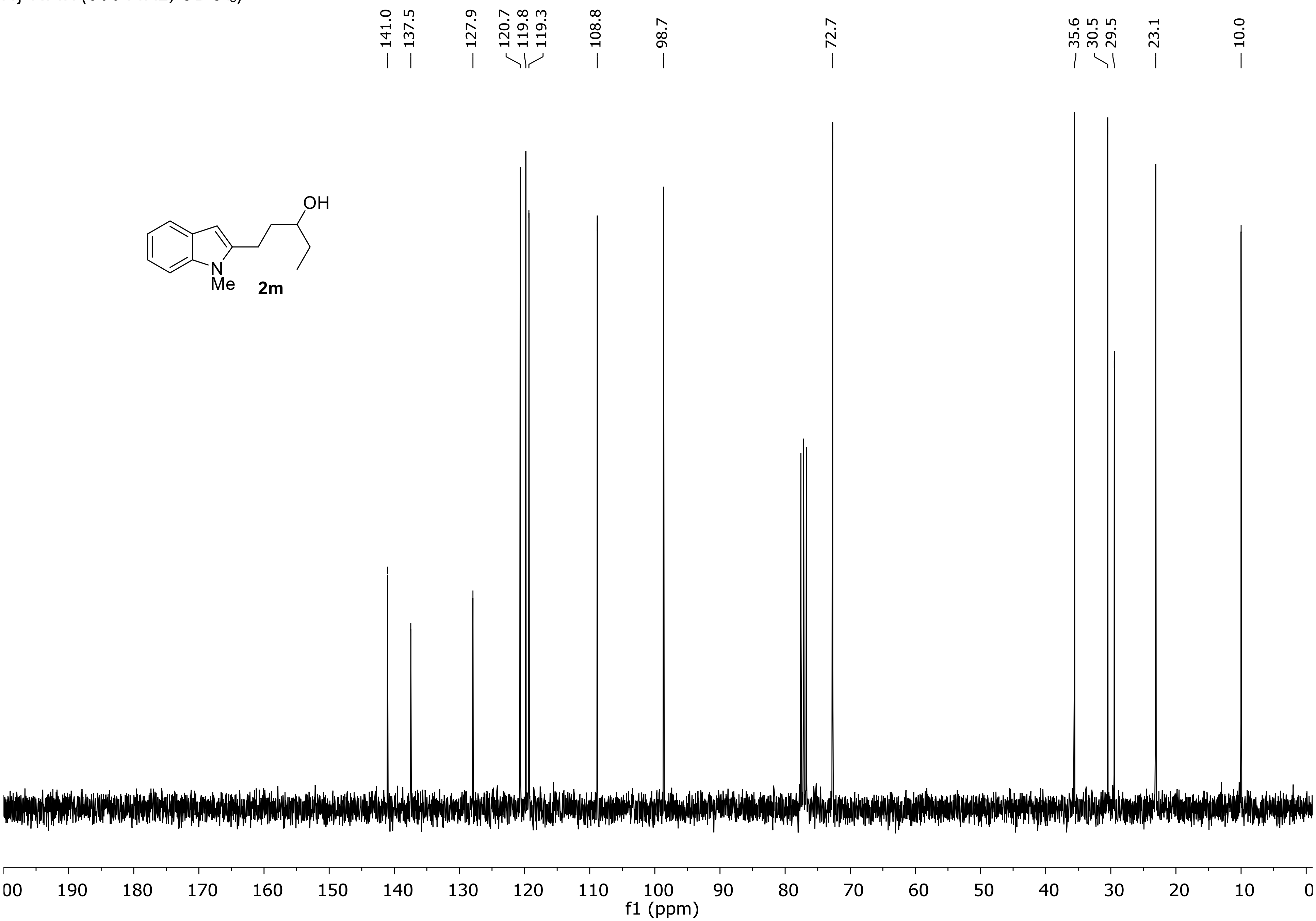

^{1}H-NMR (75.4 MHz, $CDCl_3$)

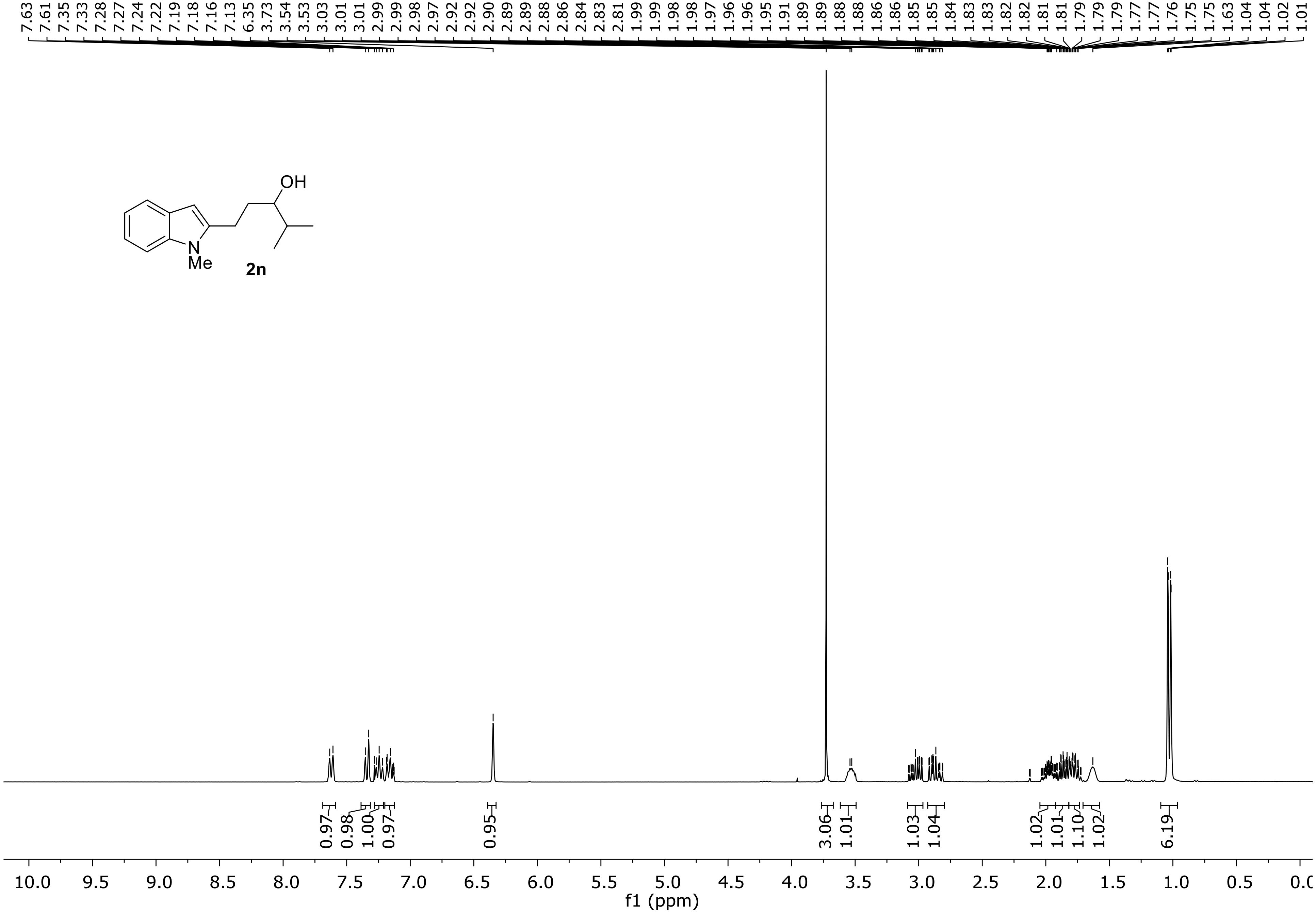

2n

$^{13}C\{^{1}H\}$-NMR (300 MHz, $CDCl_3$)

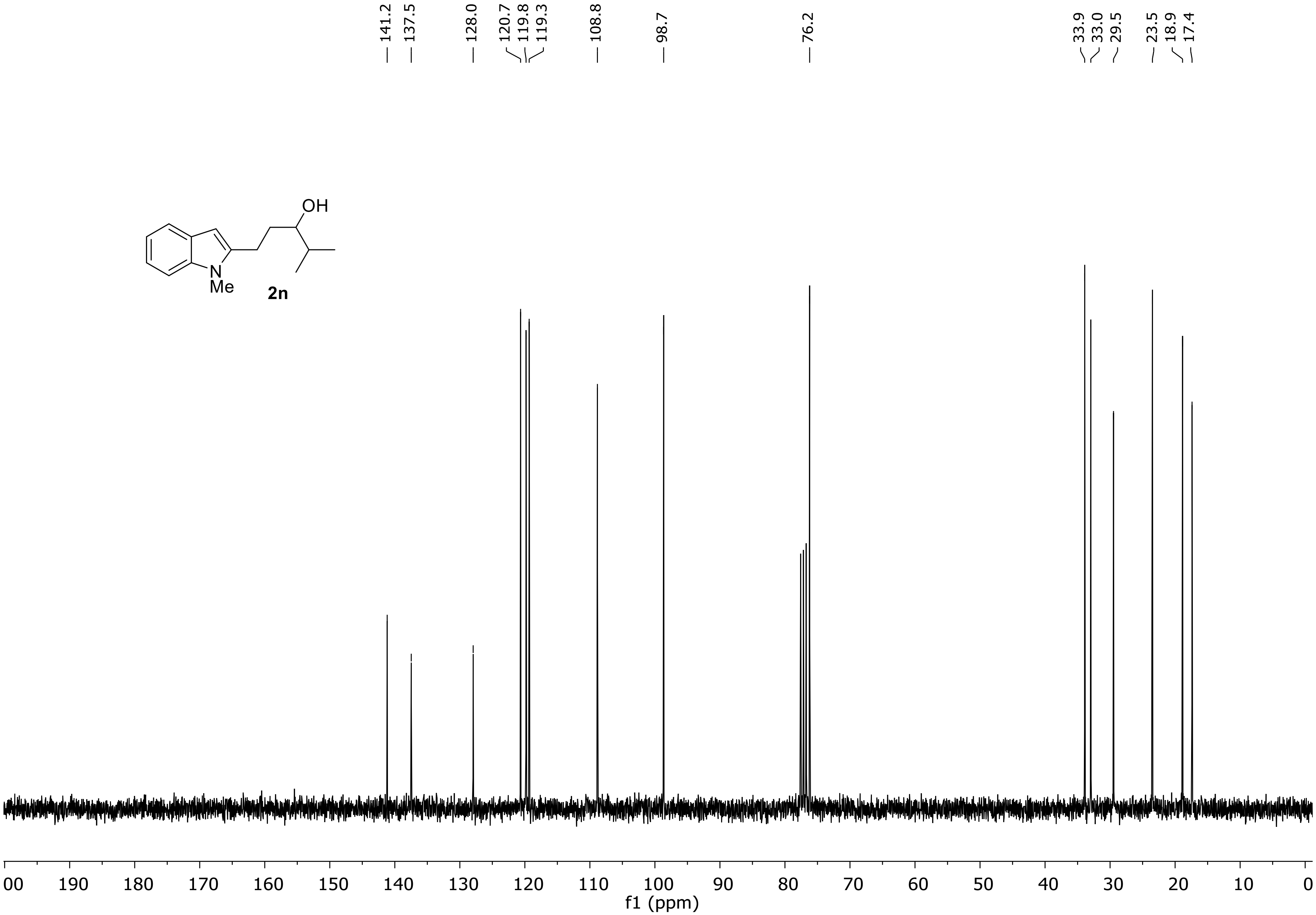

^{1}H-NMR (75.4 MHz, $CDCl_3$)

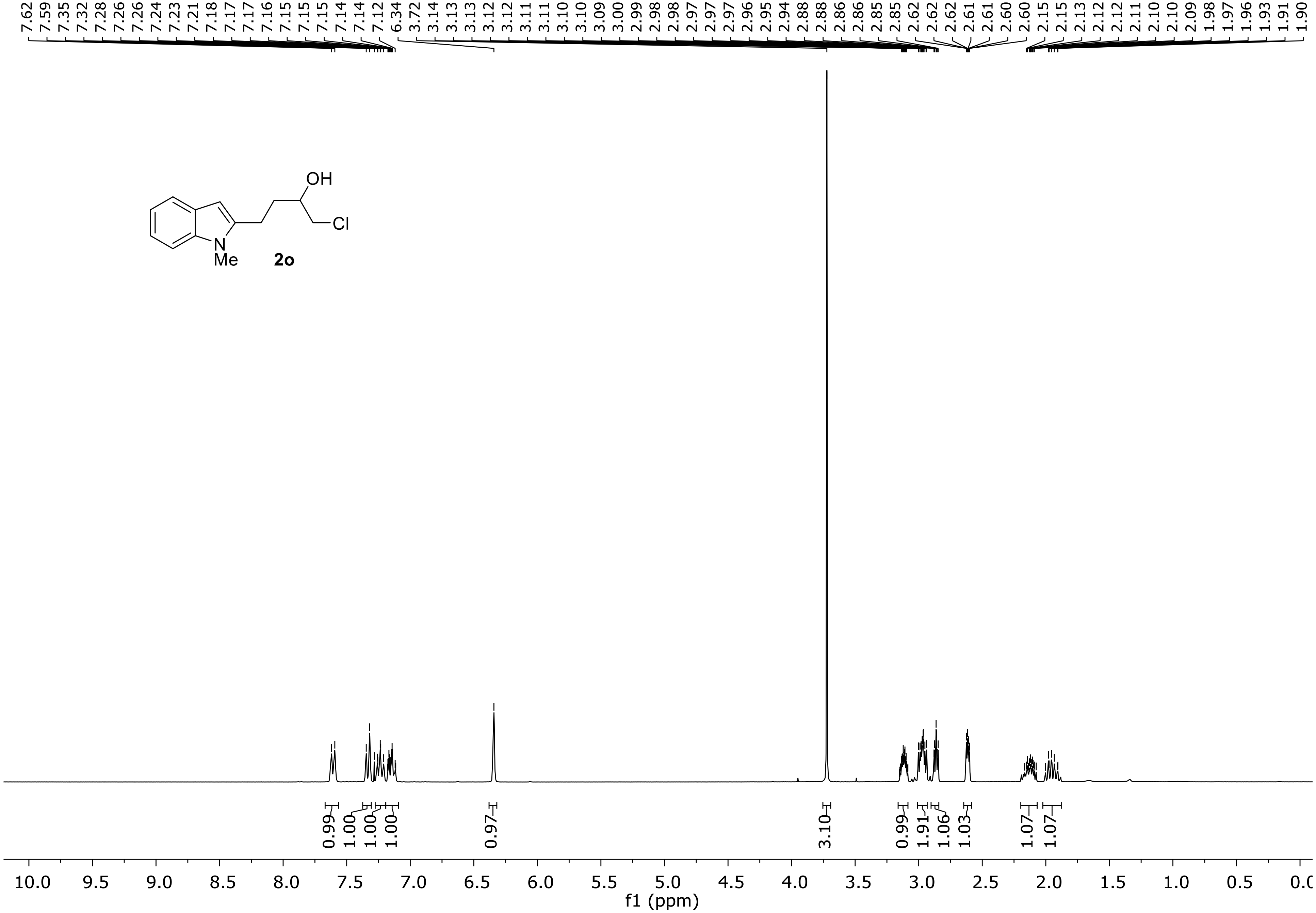

$^{13}C\{^{1}H\}$-NMR (300 MHz, $CDCl_3$)

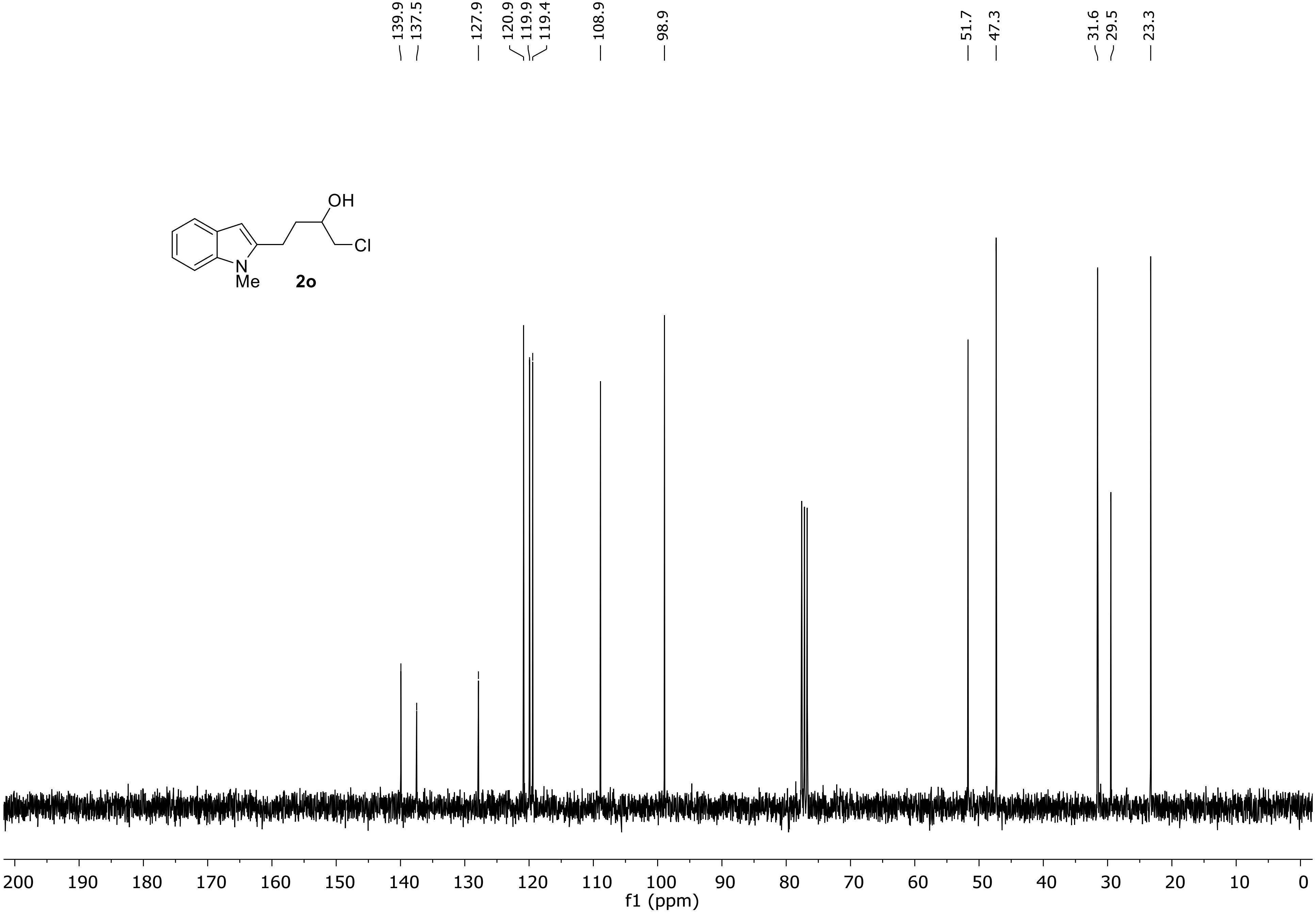

^{1}H-NMR (126 MHz, $CDCl_3$)

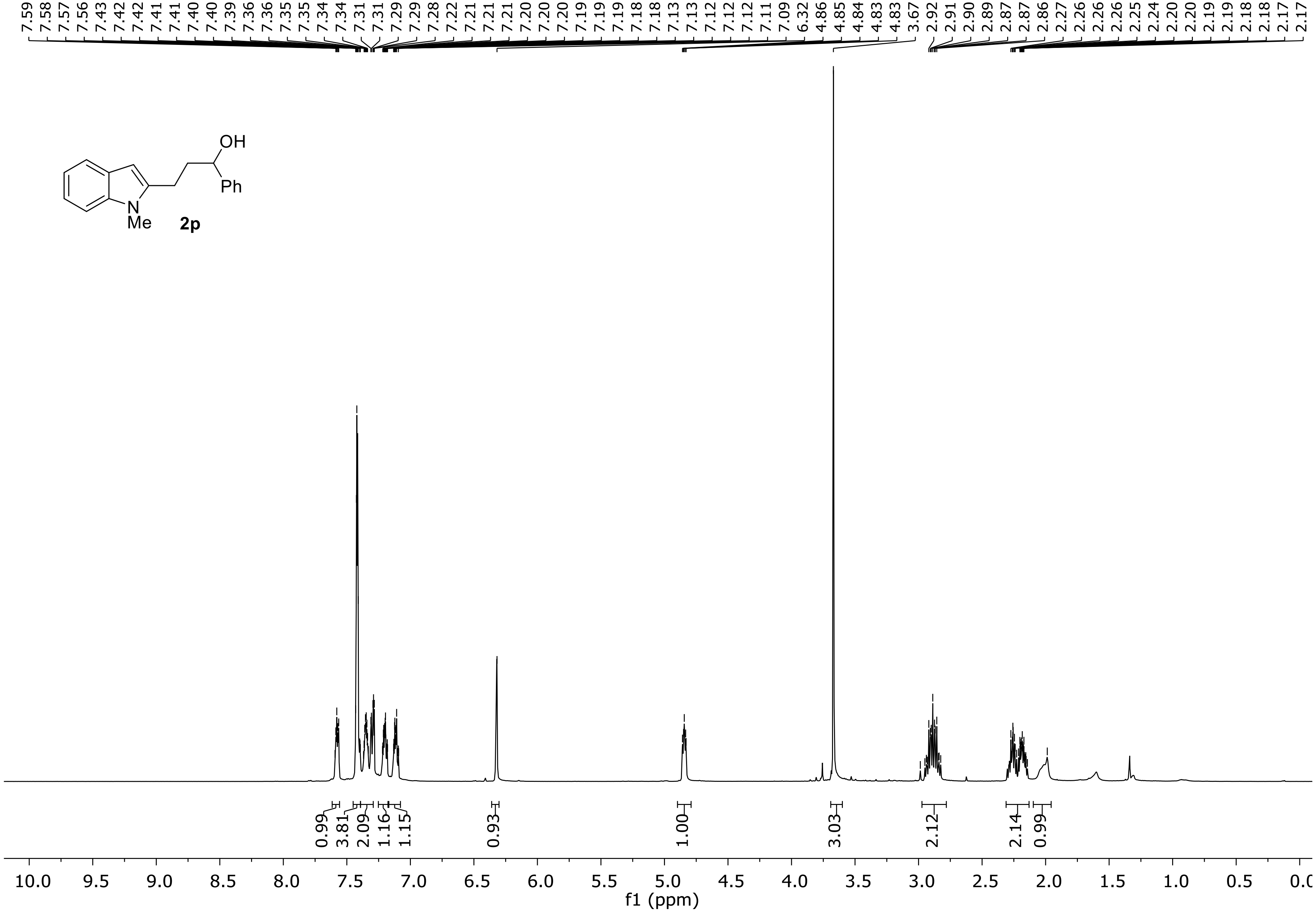

$^{13}C\{^1H\}$-NMR (500 MHz, $CDCl_3$)

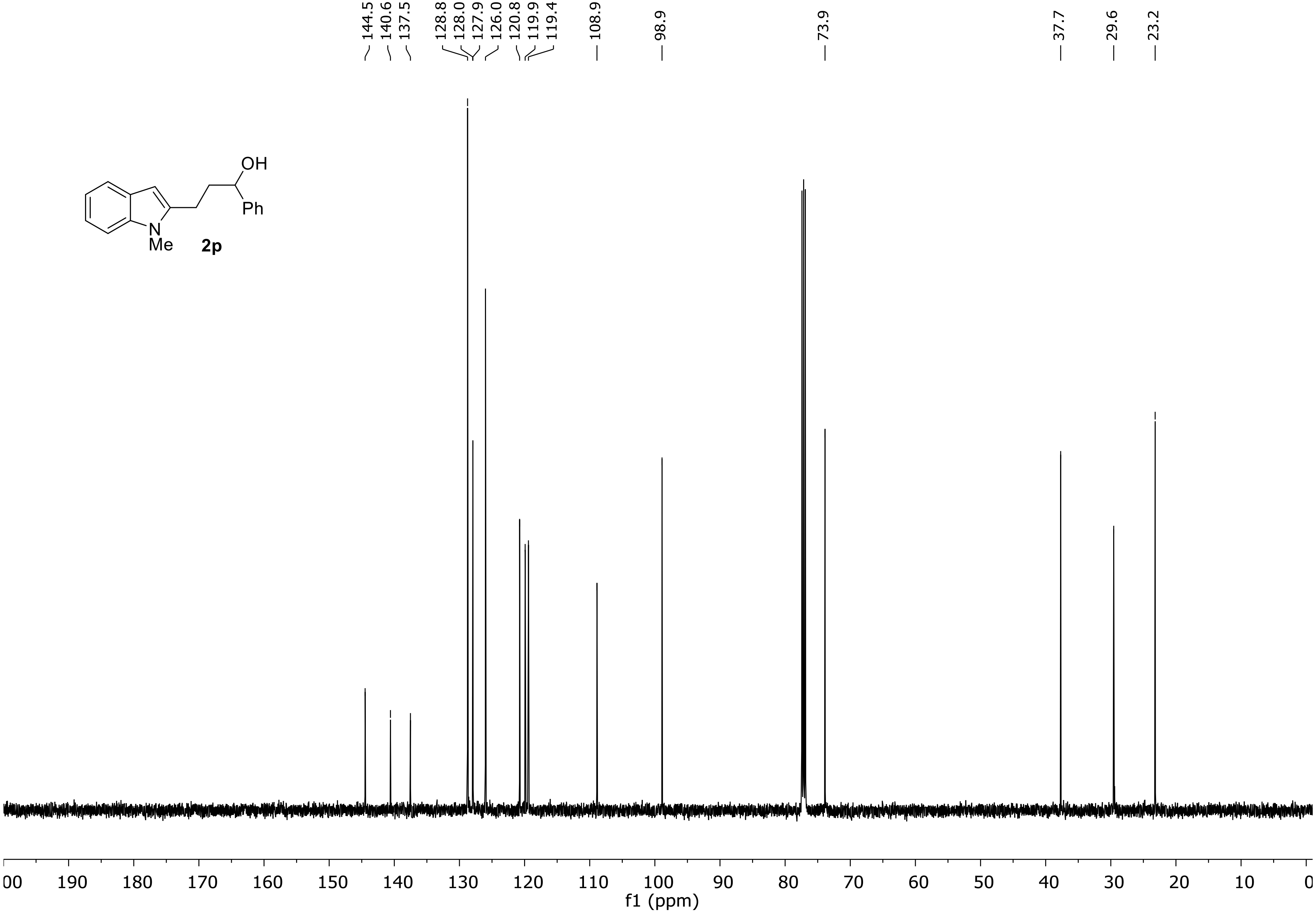

^{1}H-NMR (126 MHz, $CDCl_3$)

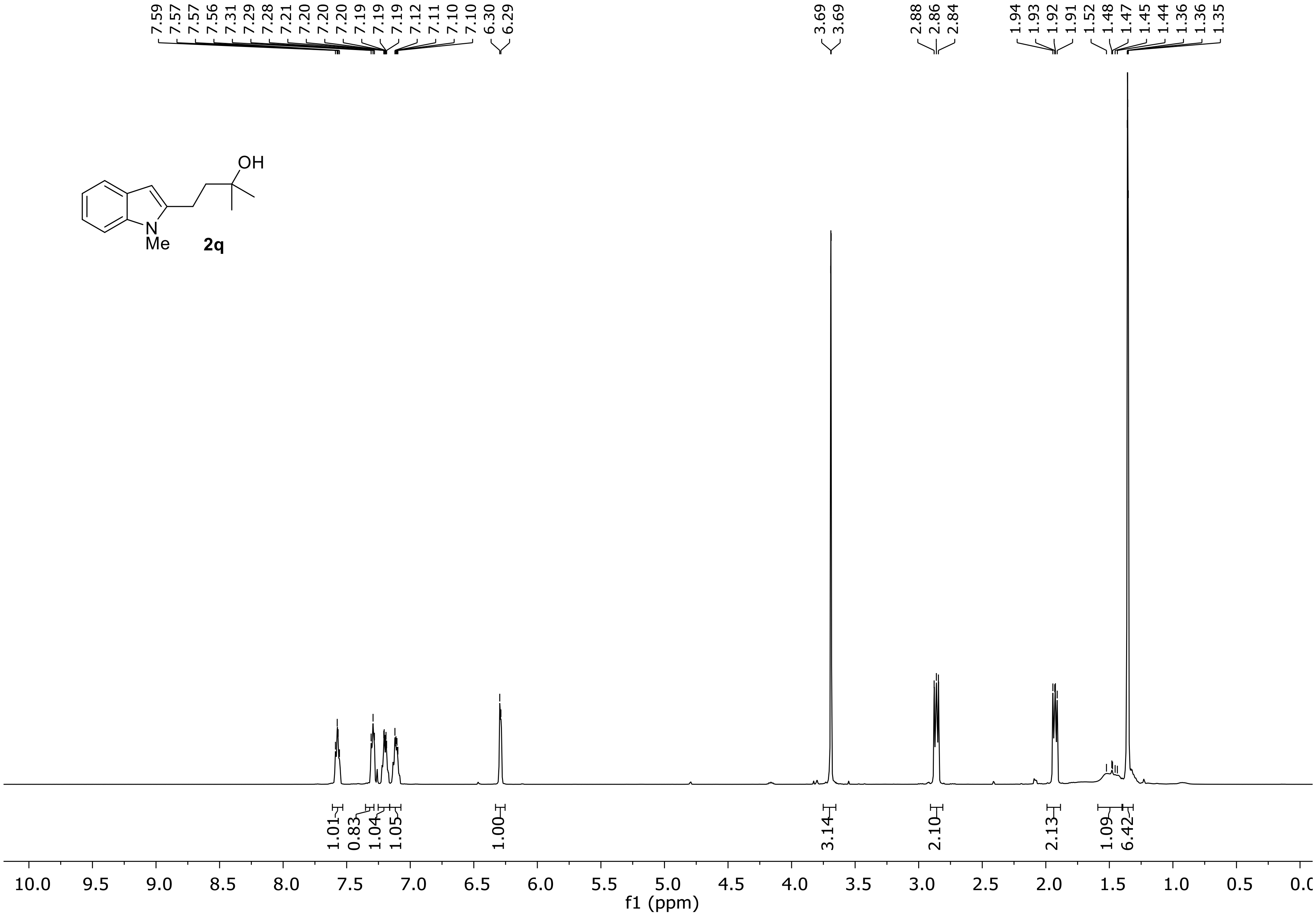

$^{13}C\{^{1}H\}$-NMR (500 MHz, $CDCl_3$)

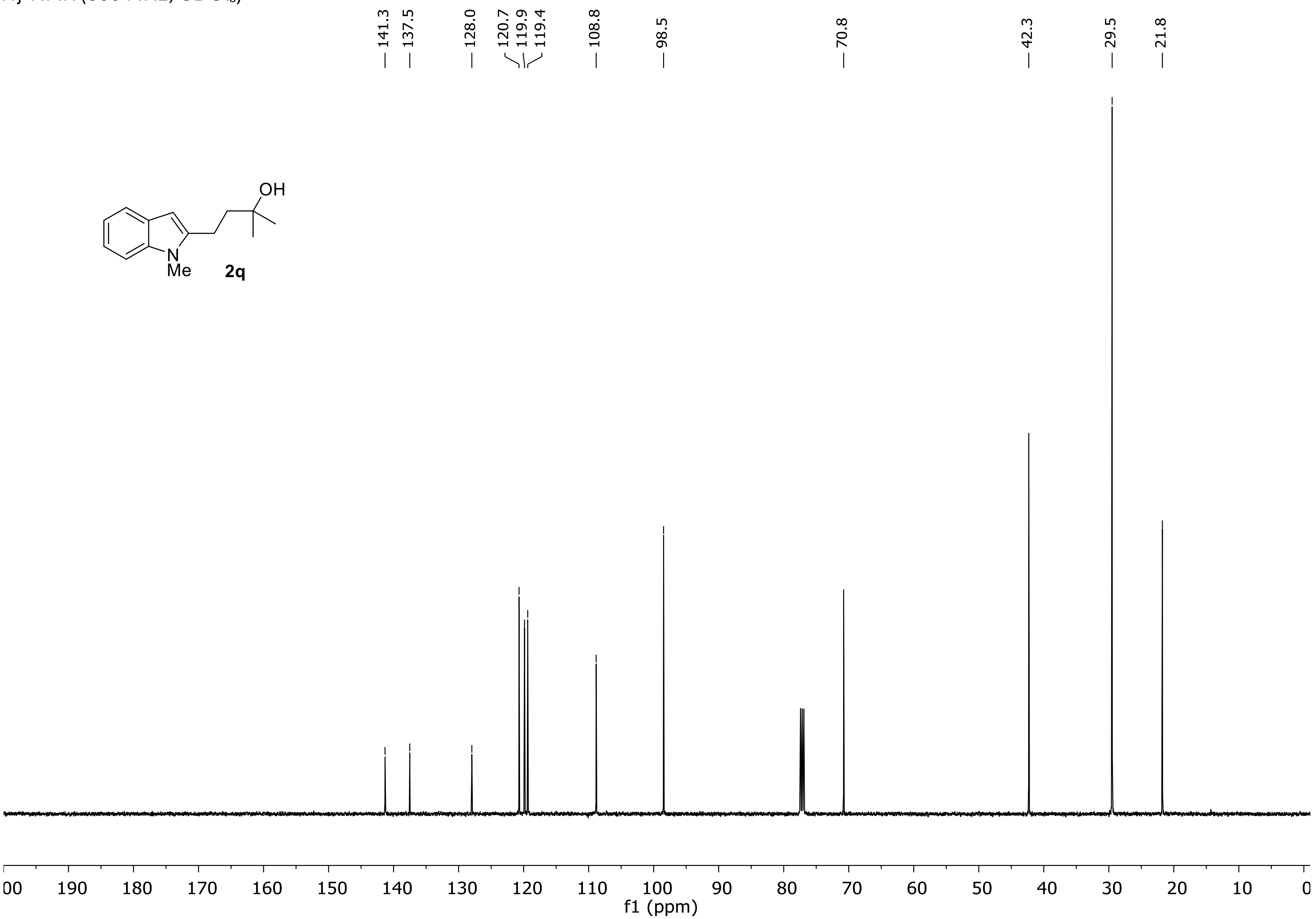

2q

^{1}H-NMR (126 MHz, $CDCl_3$)

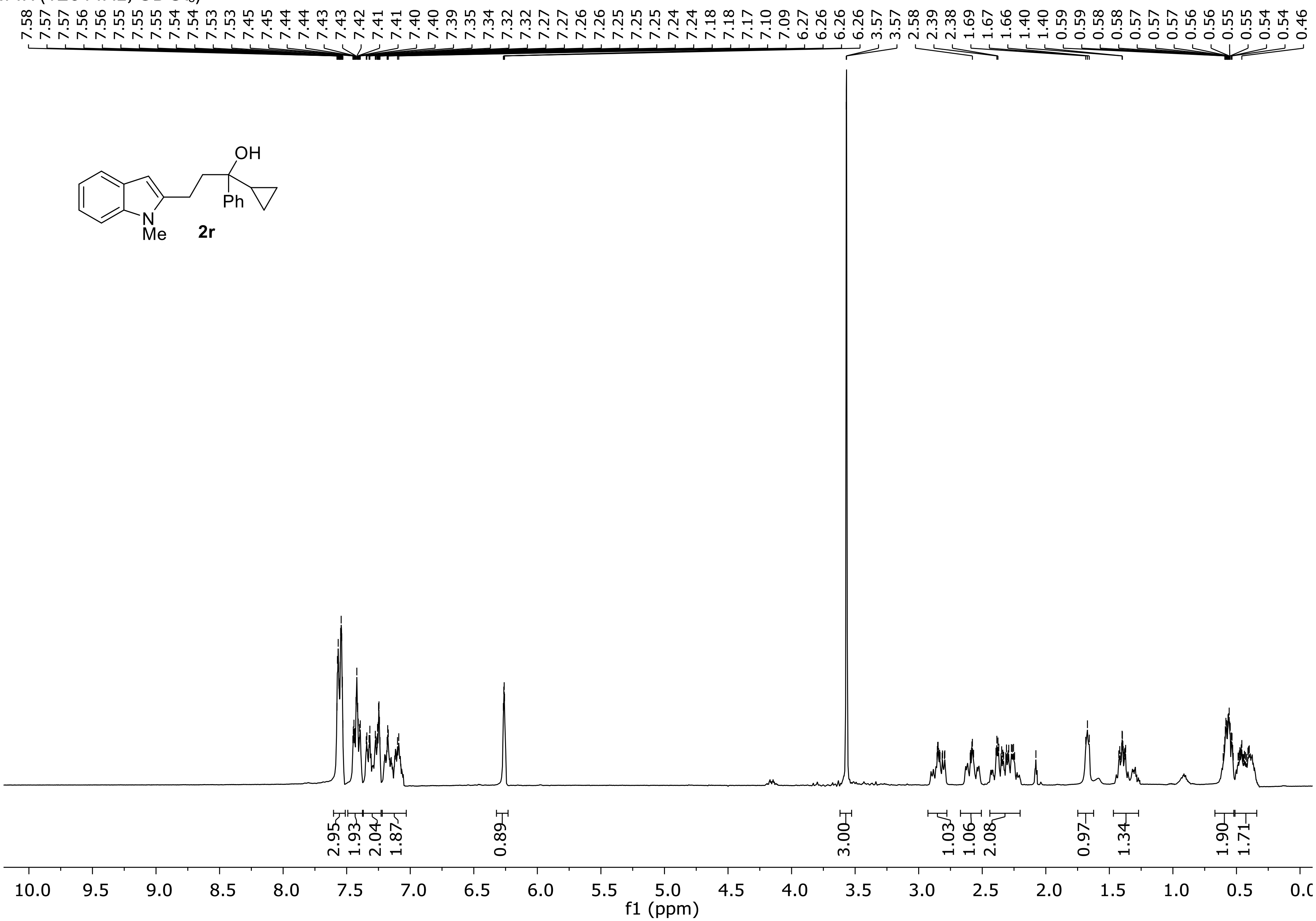

2r

$^{13}C\{^1H\}$-NMR (500 MHz, $CDCl_3$)

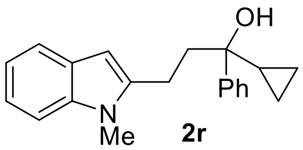

2r

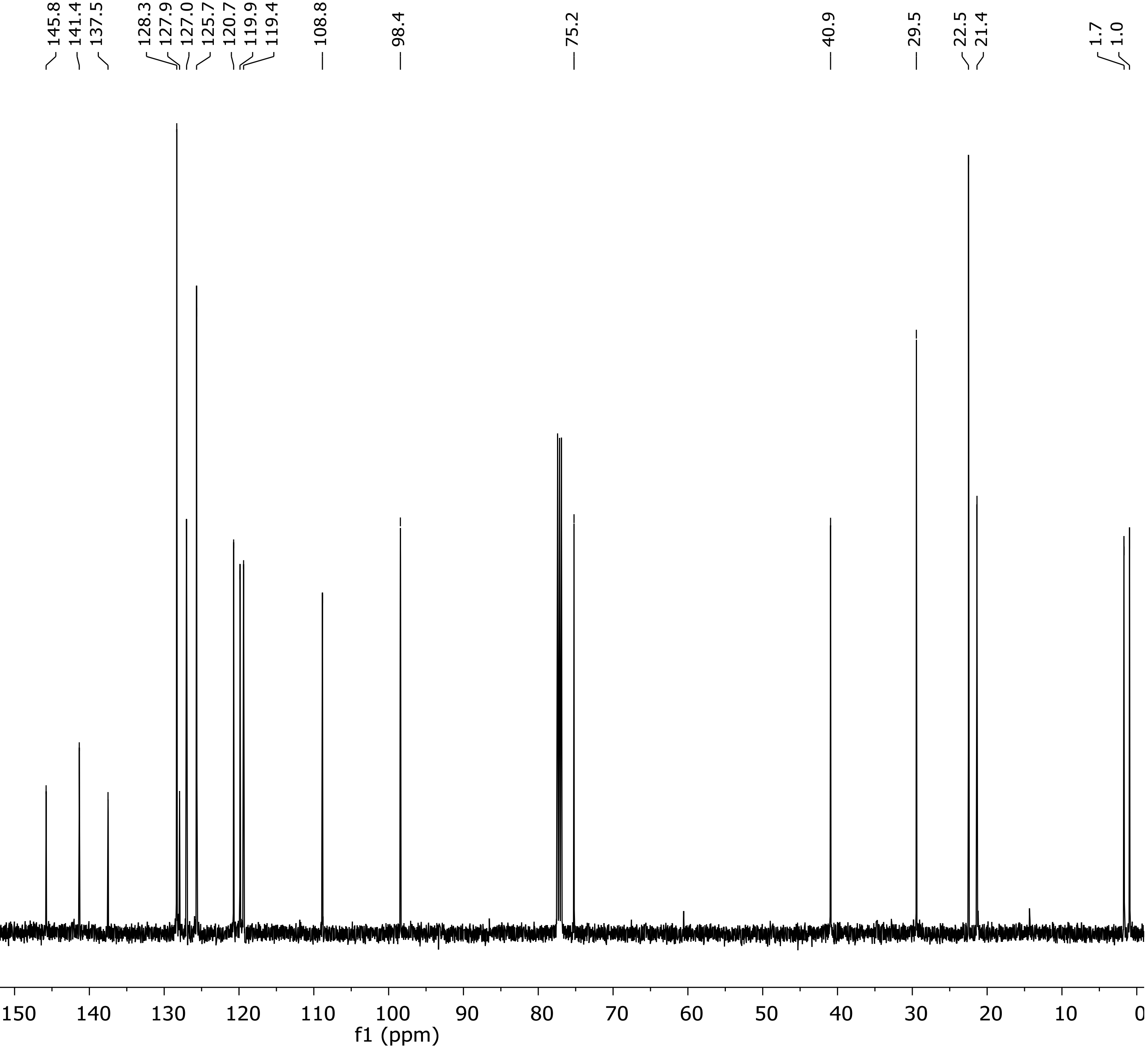

^{1}H-NMR (126 MHz, $CDCl_3$)

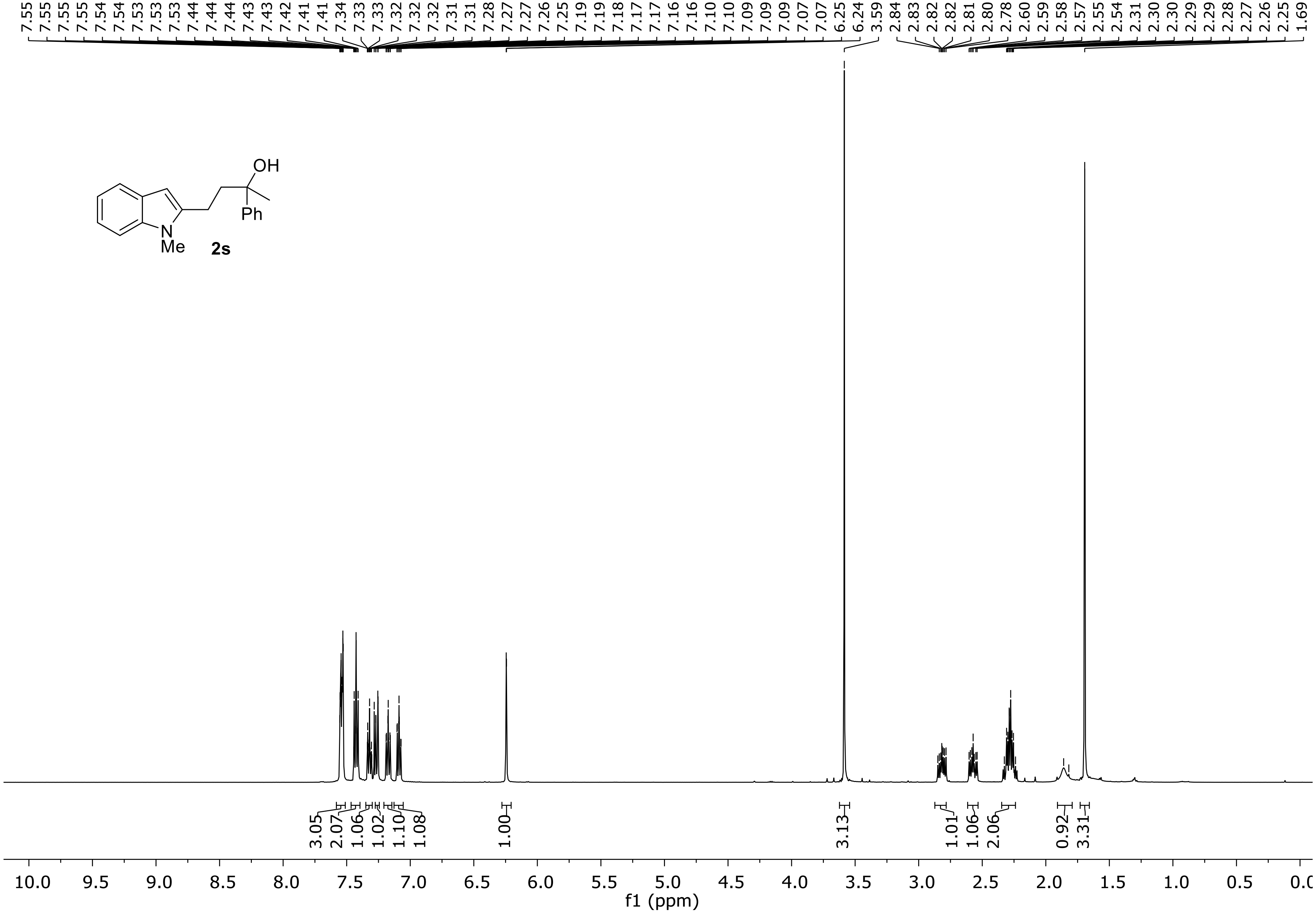

$^{13}C\{^{1}H\}$-NMR (500 MHz, $CDCl_3$)

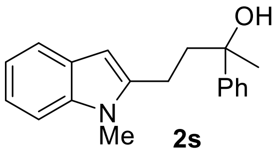

2s

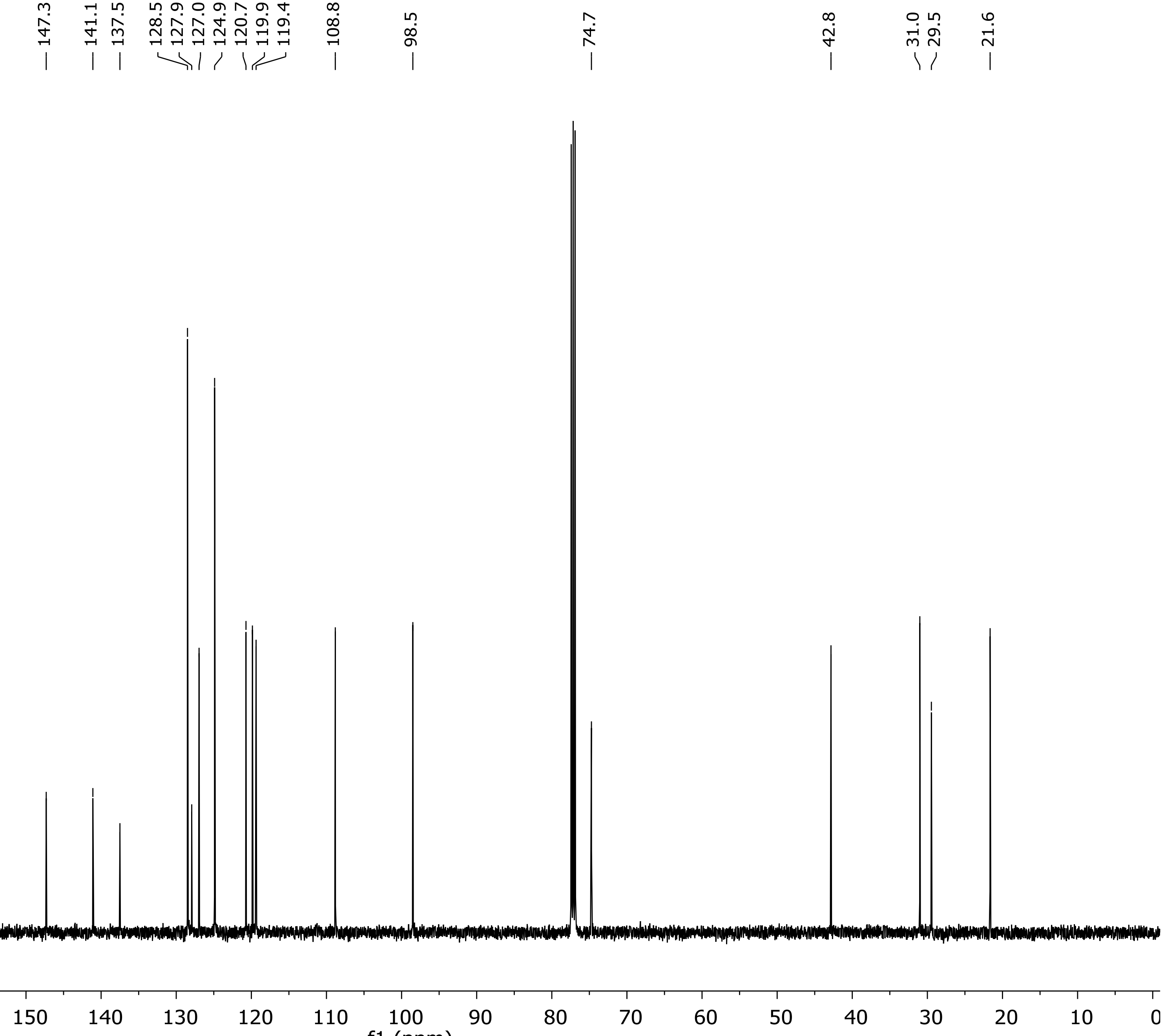

^{1}H-NMR (126 MHz, $CDCl_3$)

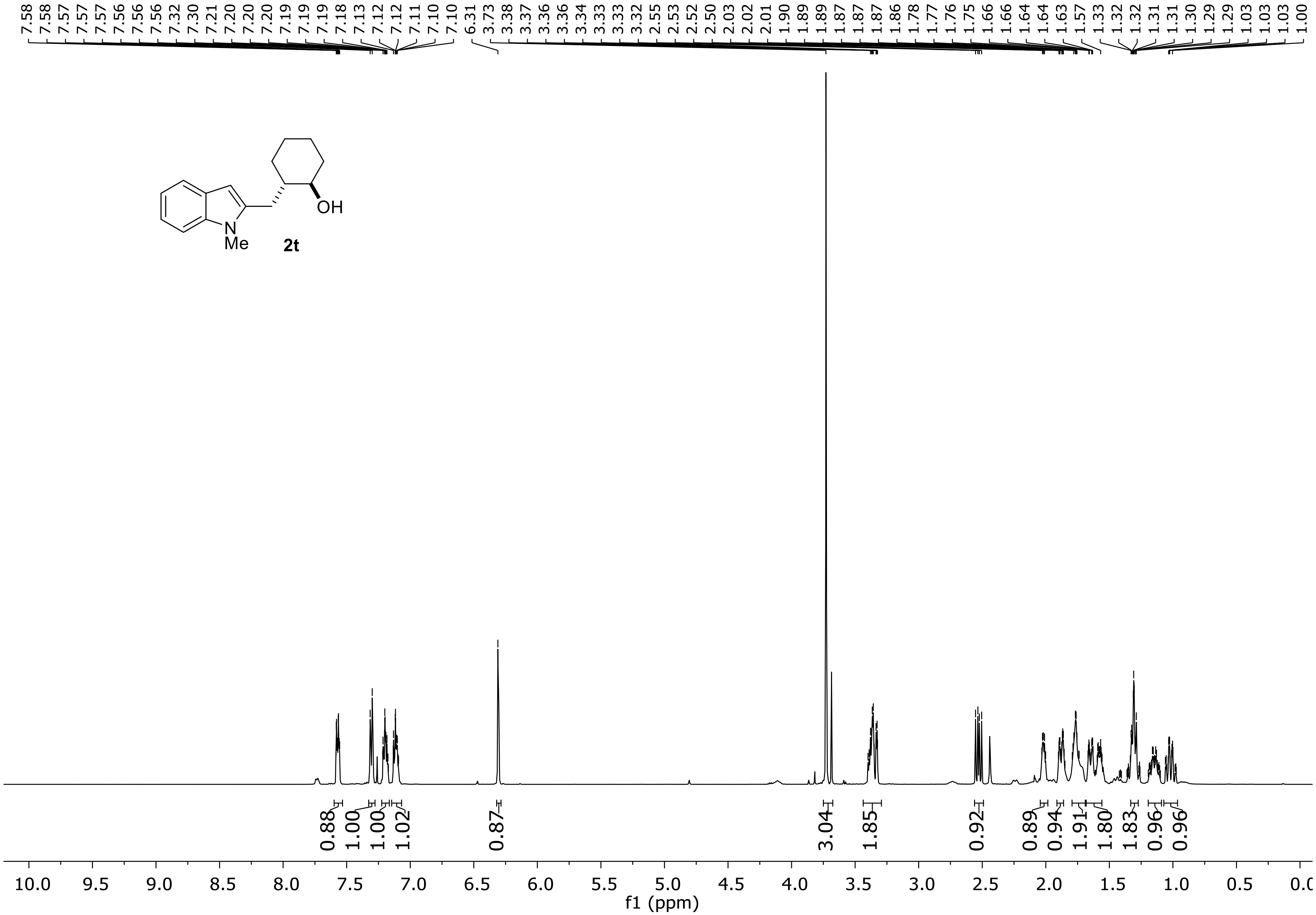

$^{13}C\{^1H\}$-NMR (500 MHz, $CDCl_3$)

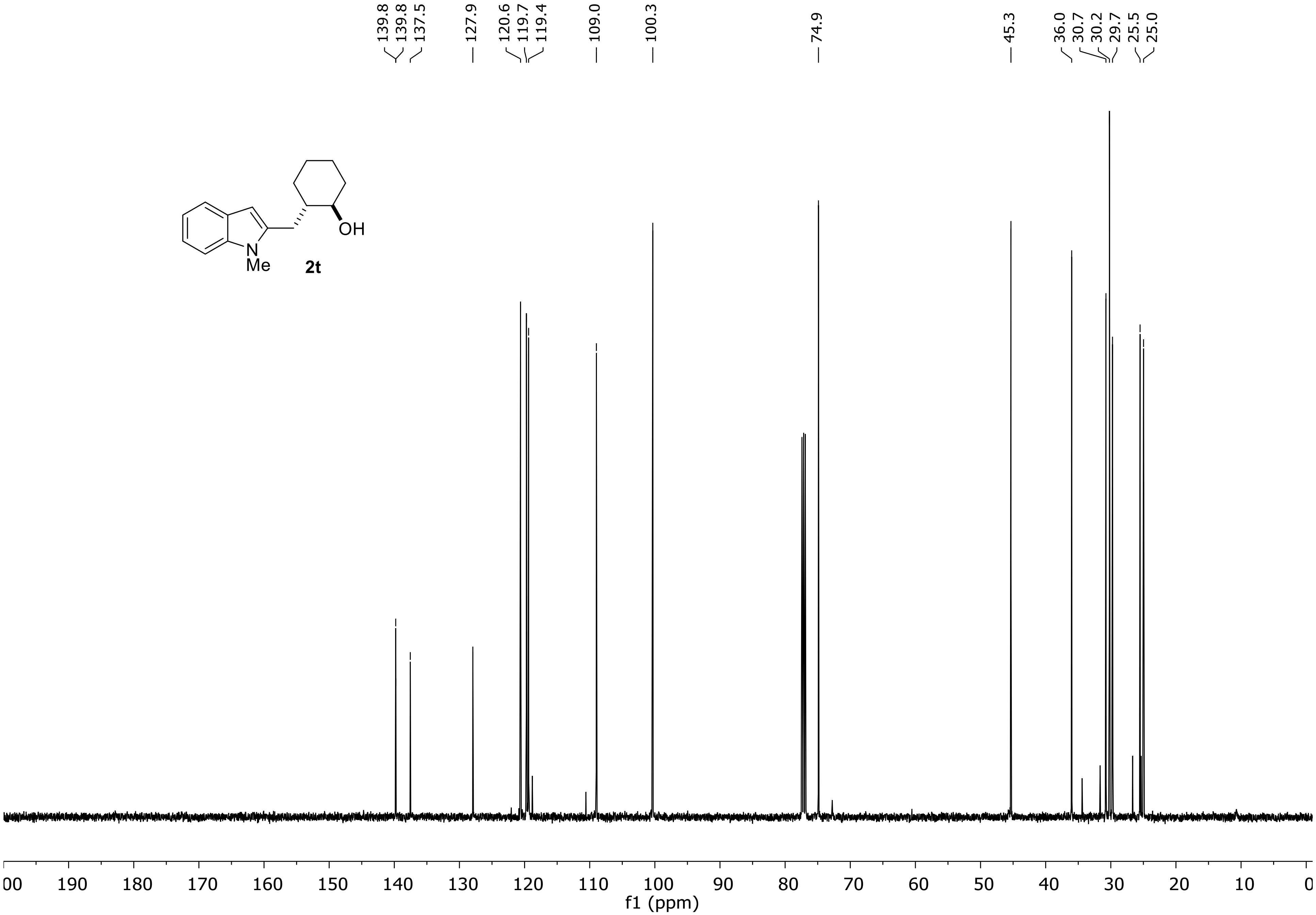

^{1}H-NMR (126 MHz, $CDCl_3$)

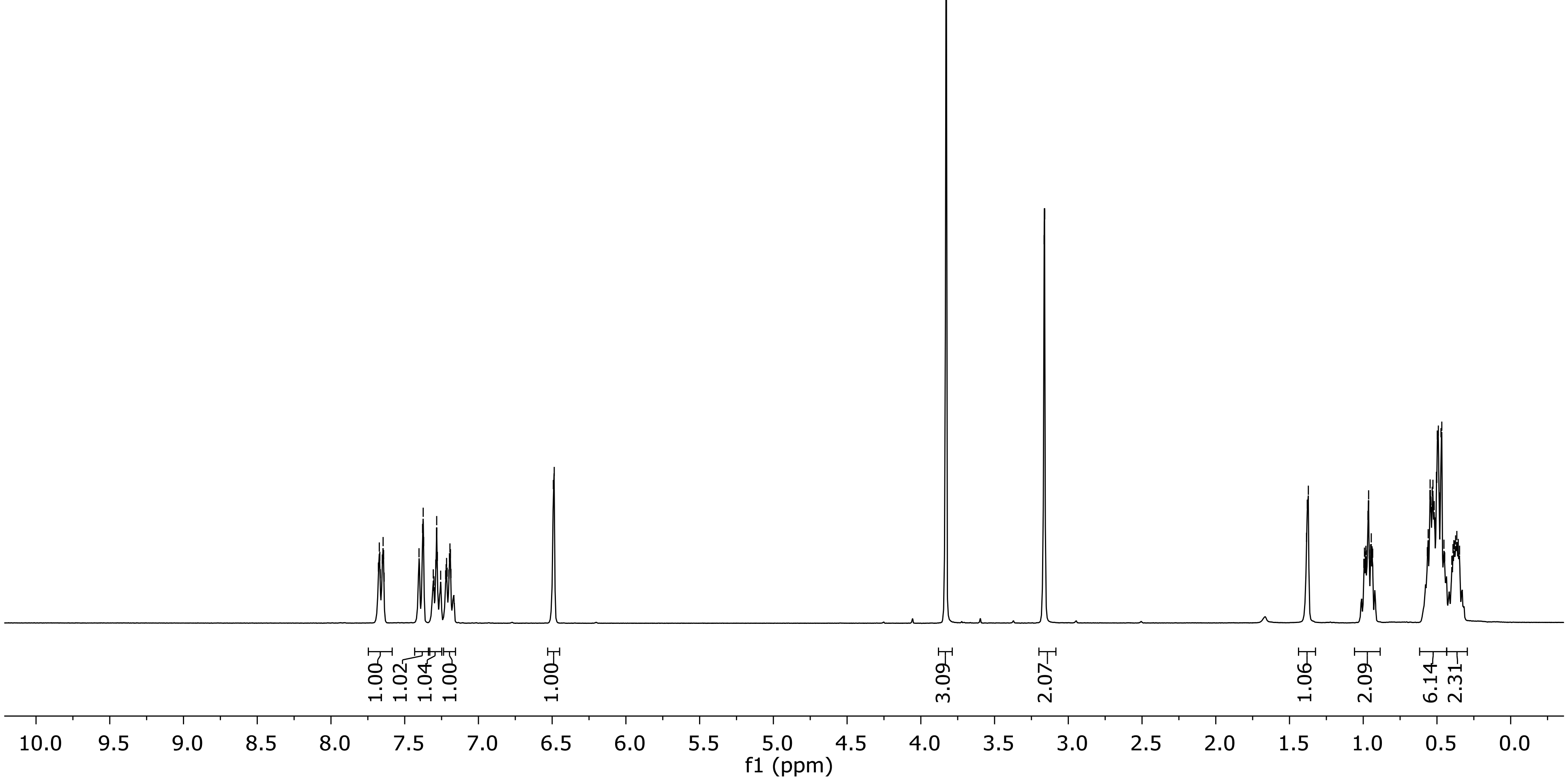

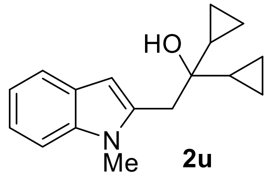

2u

$^{13}C\{^1H\}$-NMR (500 MHz, $CDCl_3$)

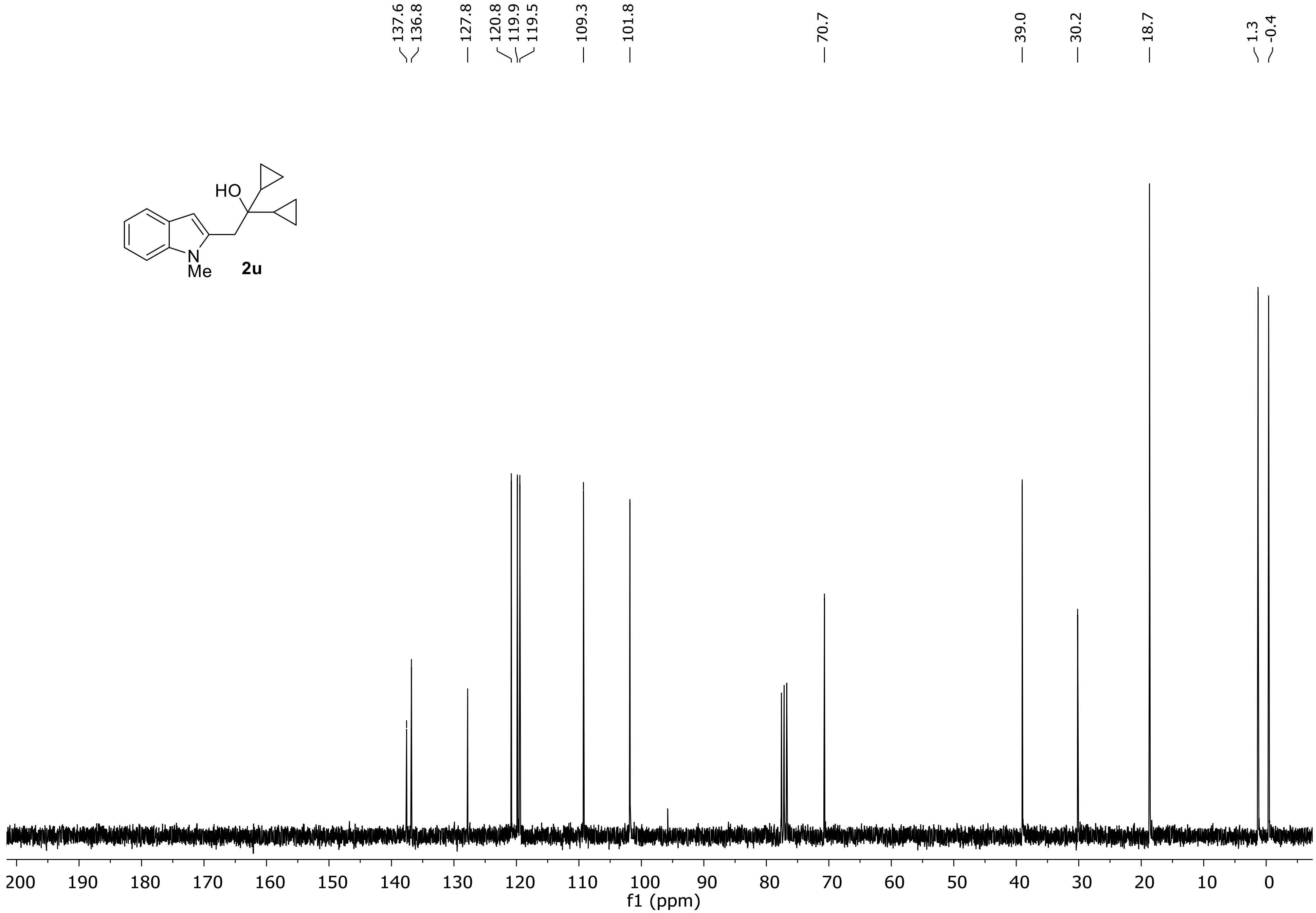

^{1}H-NMR (126 MHz, $CDCl_3$)

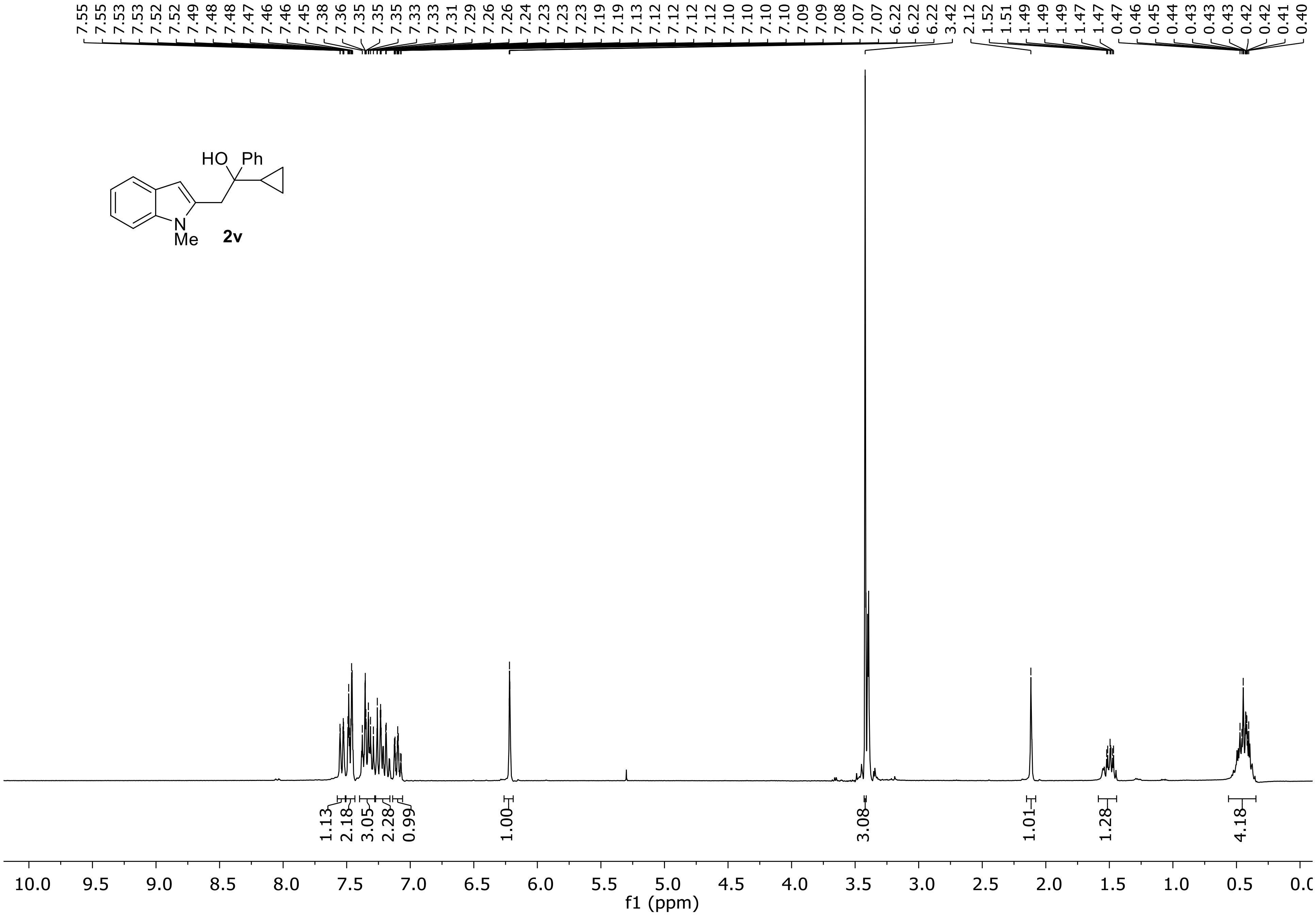

$^{13}C\{^1H\}$-NMR (500 MHz, $CDCl_3$)

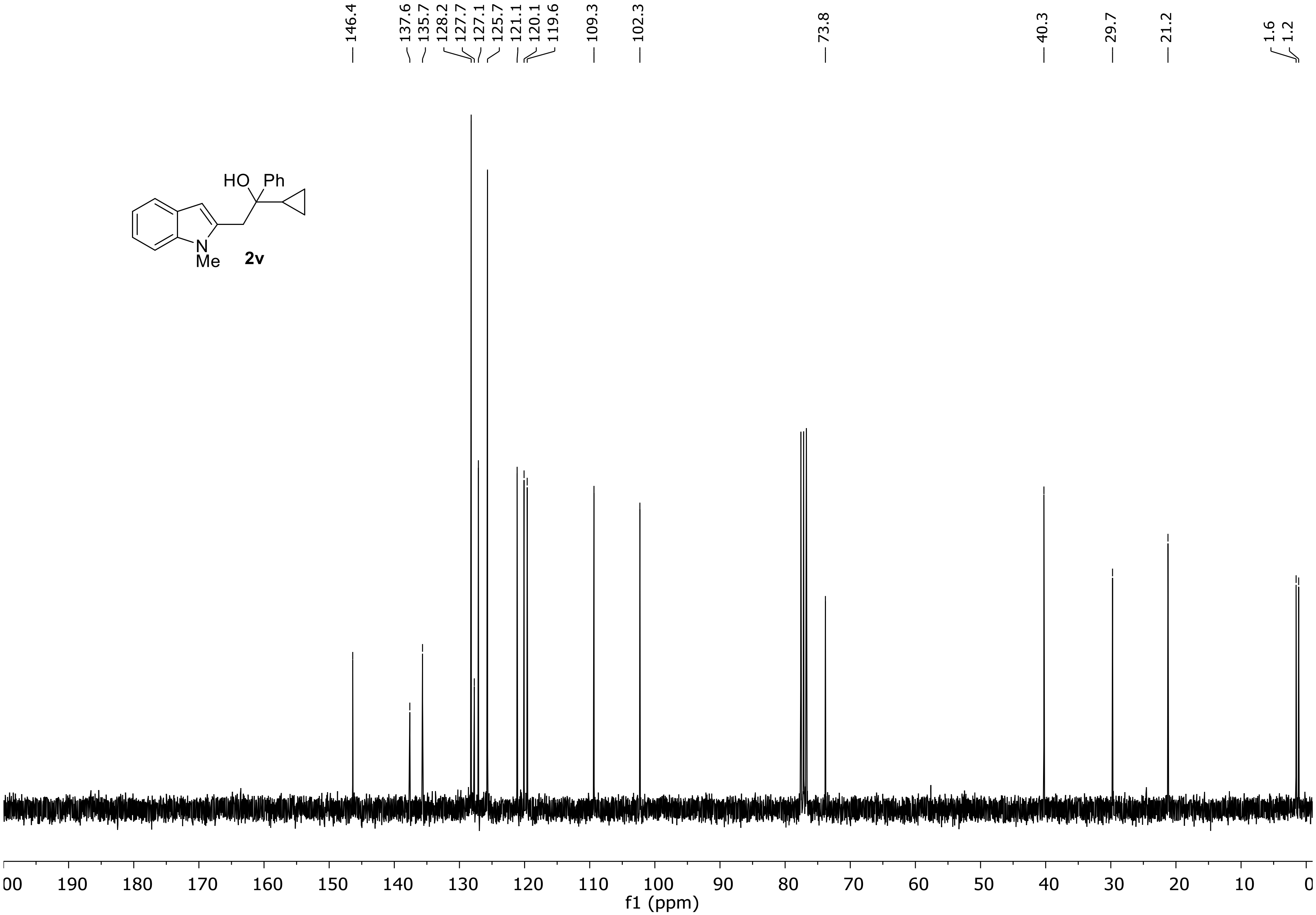

^{1}H-NMR (126 MHz, $CDCl_3$)

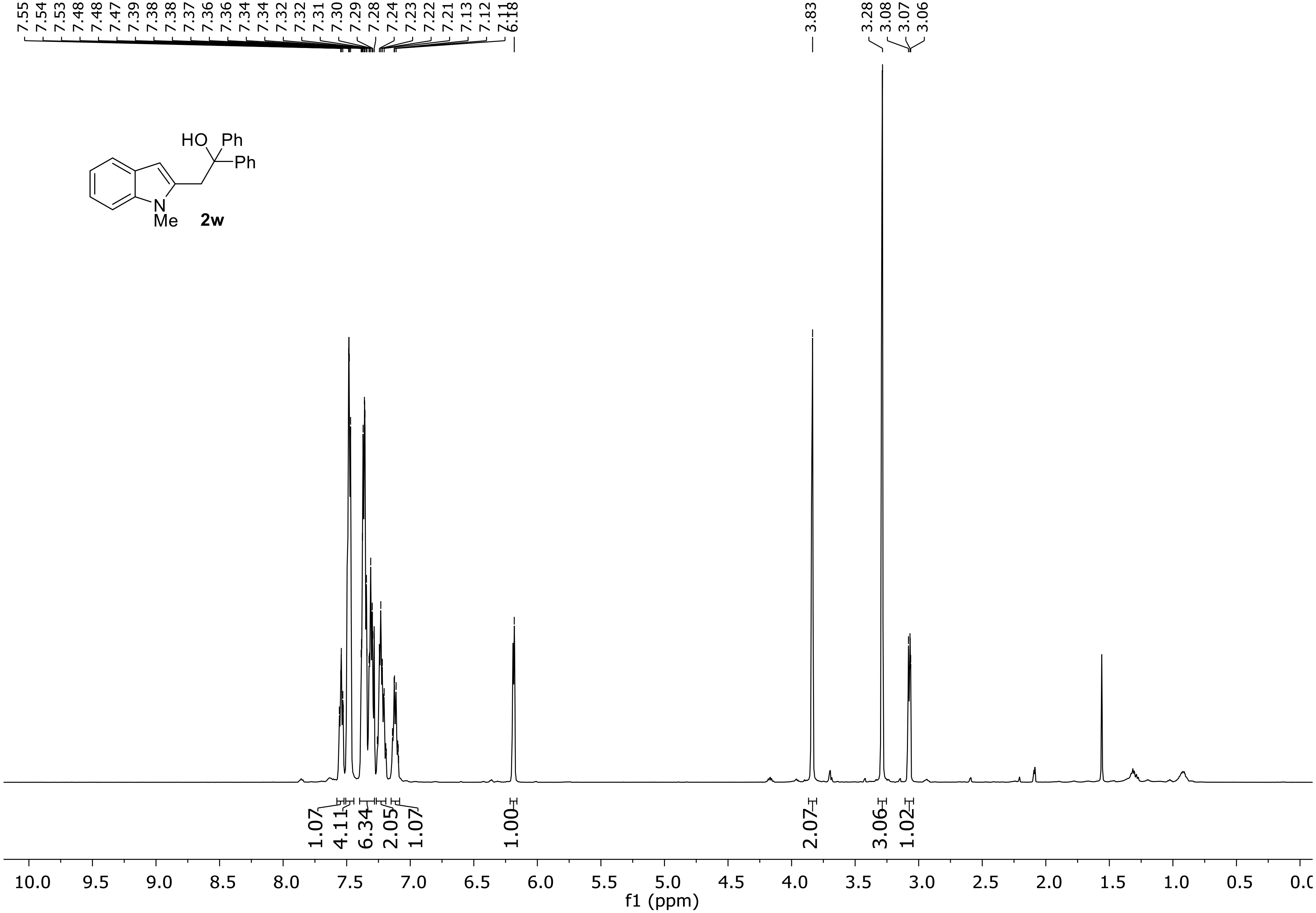

$^{13}C\{^{1}H\}$-NMR (500 MHz, $CDCl_3$)

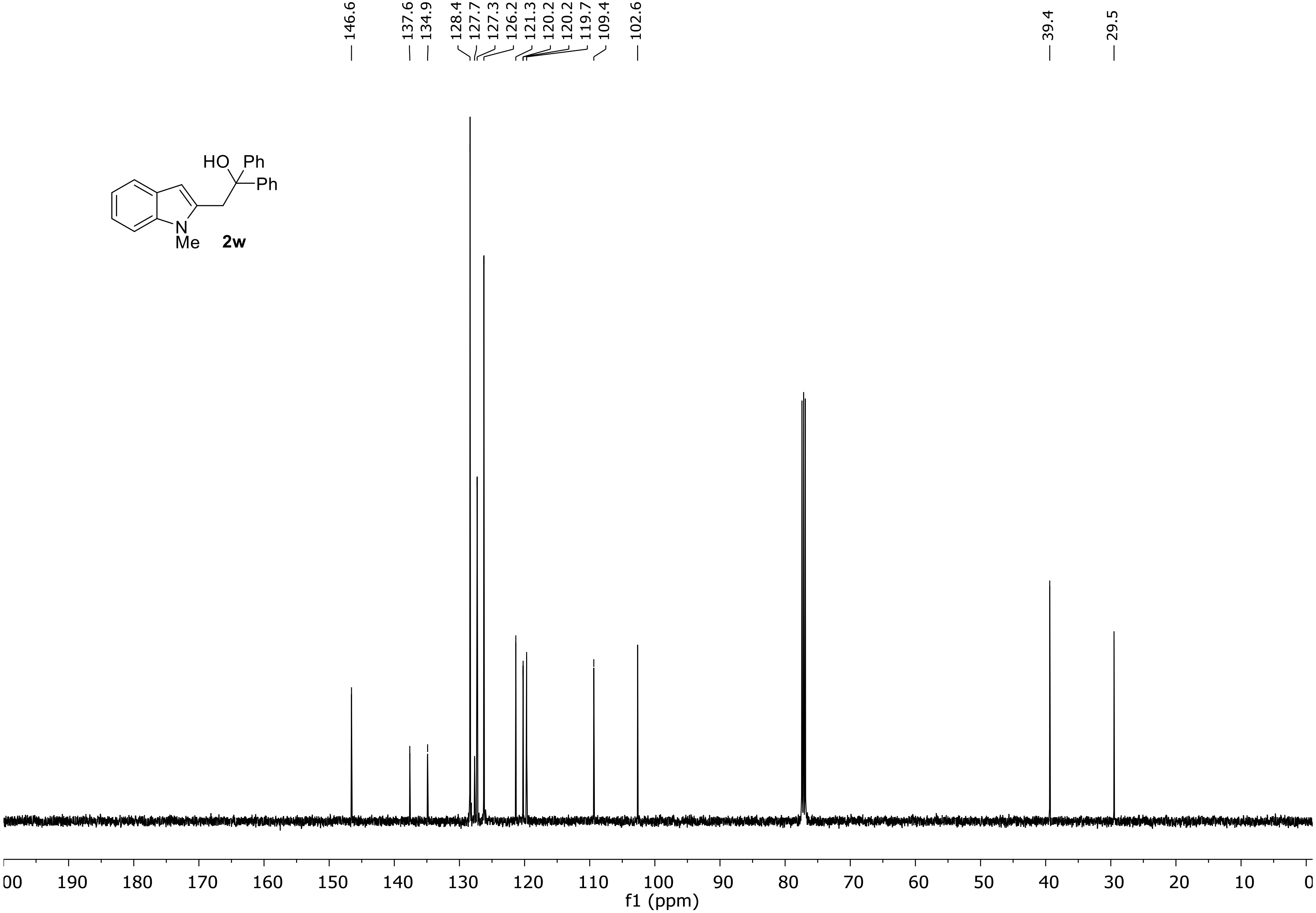

^{1}H-NMR (75.4 MHz, $CDCl_3$)

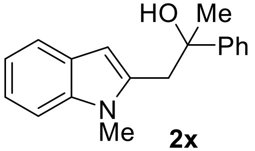

2x

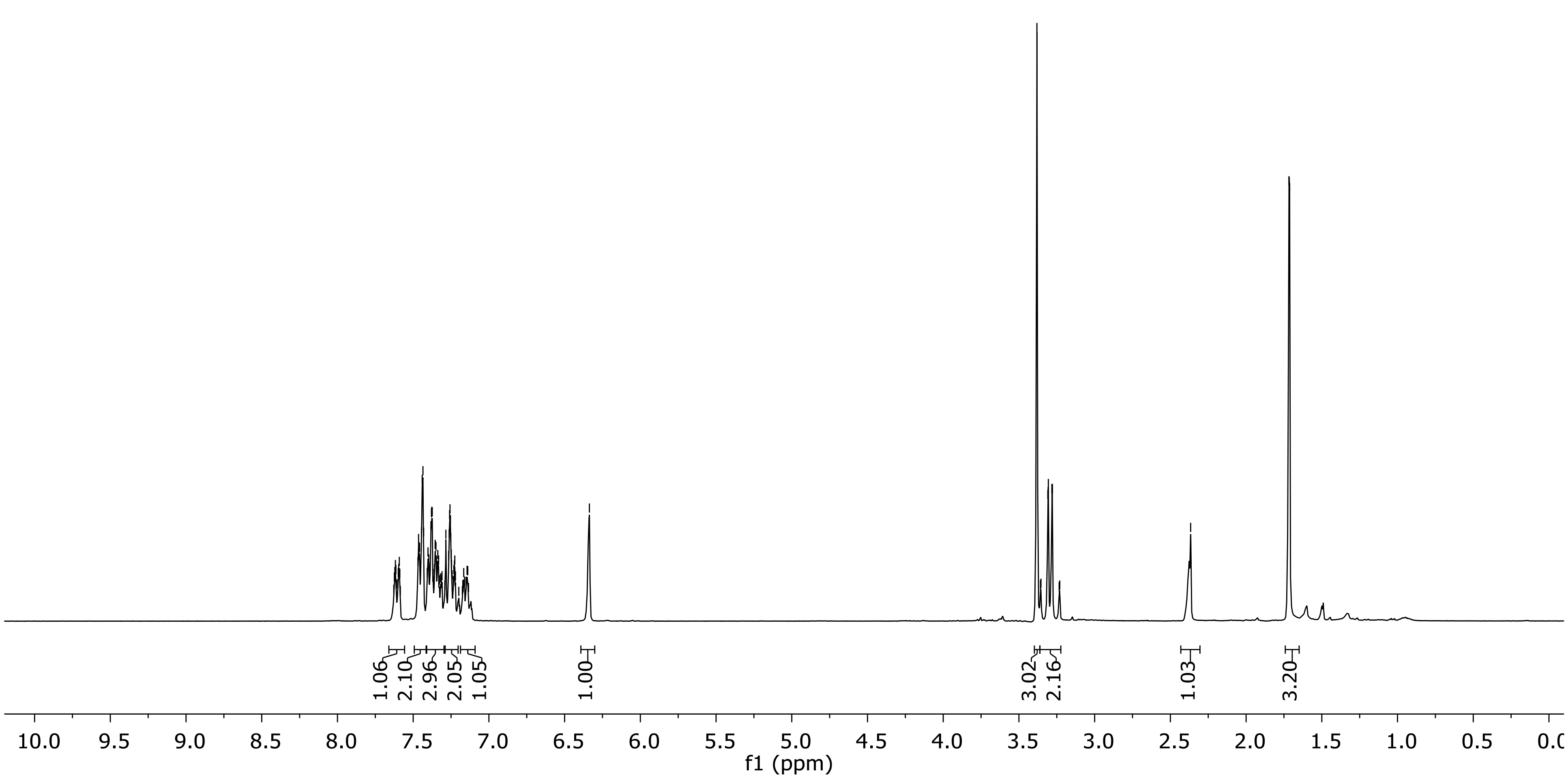

$^{13}C\{^1H\}$-NMR (300 MHz, $CDCl_3$)

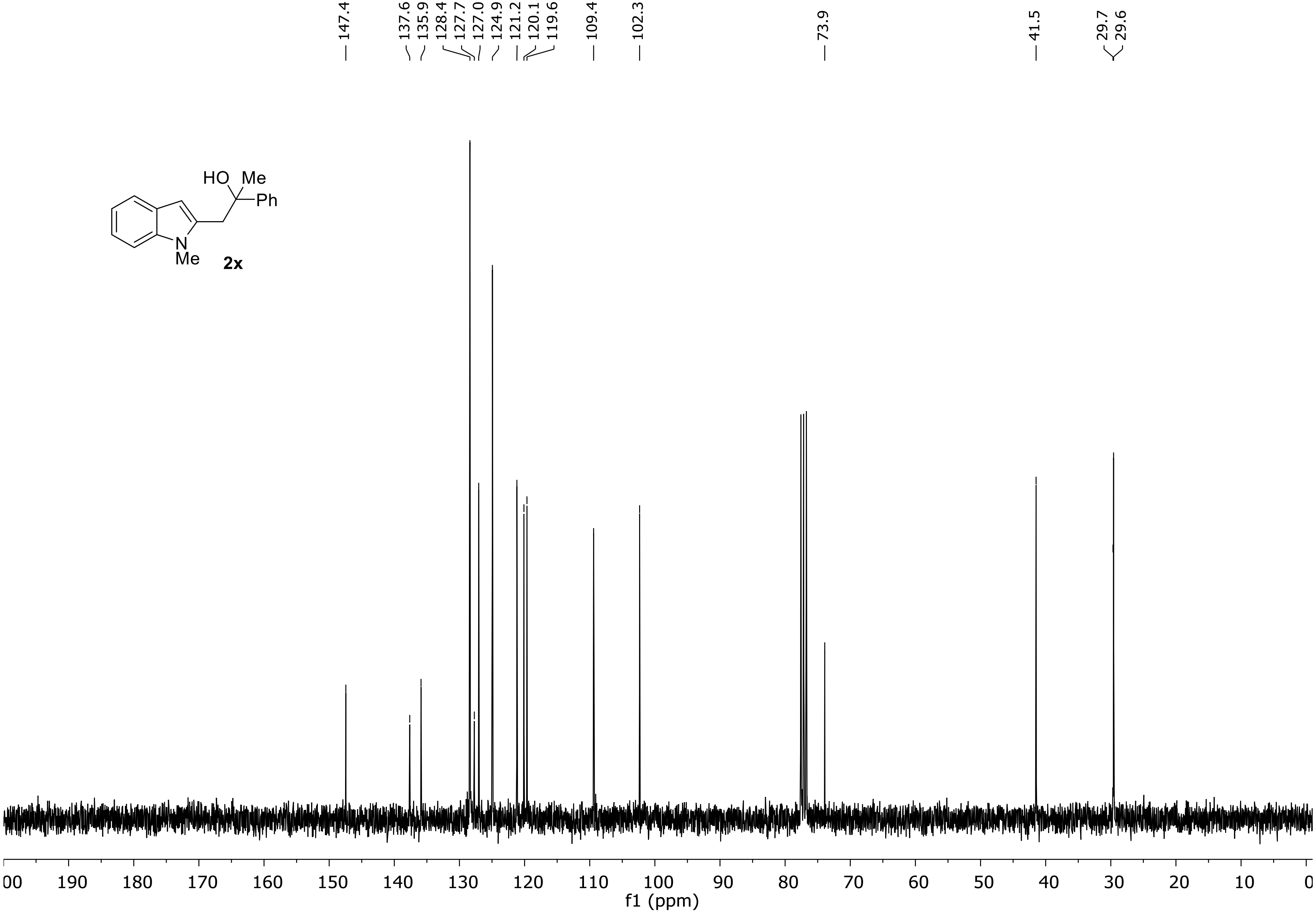

^{1}H-NMR (75.4 MHz, $CDCl_3$)

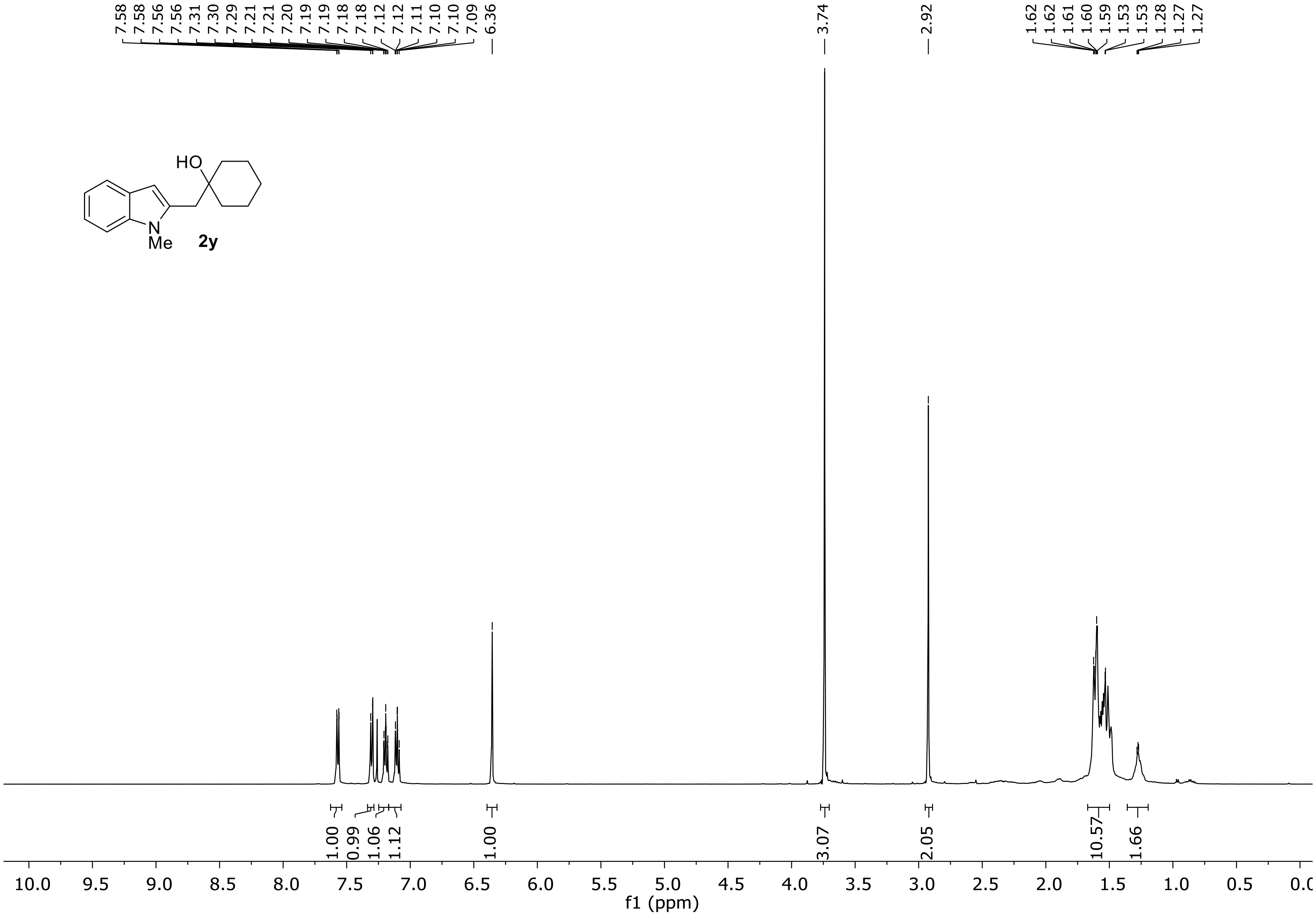

$^{13}C\{^1H\}$-NMR (300 MHz, $CDCl_3$)

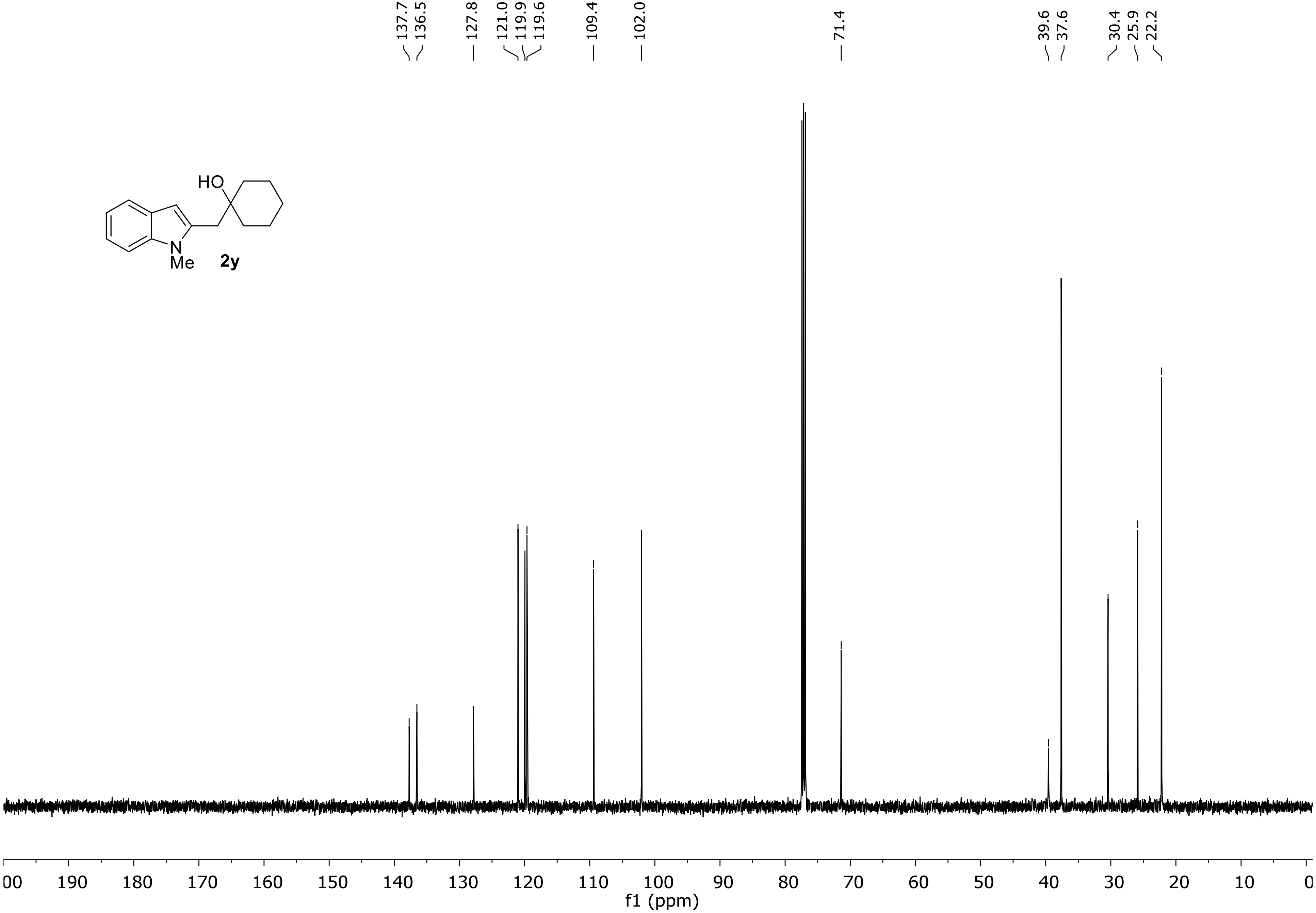

^{1}H-NMR (75.4 MHz, $CDCl_3$)

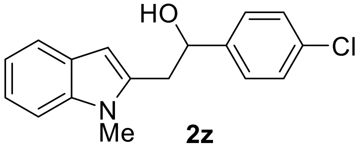

2z

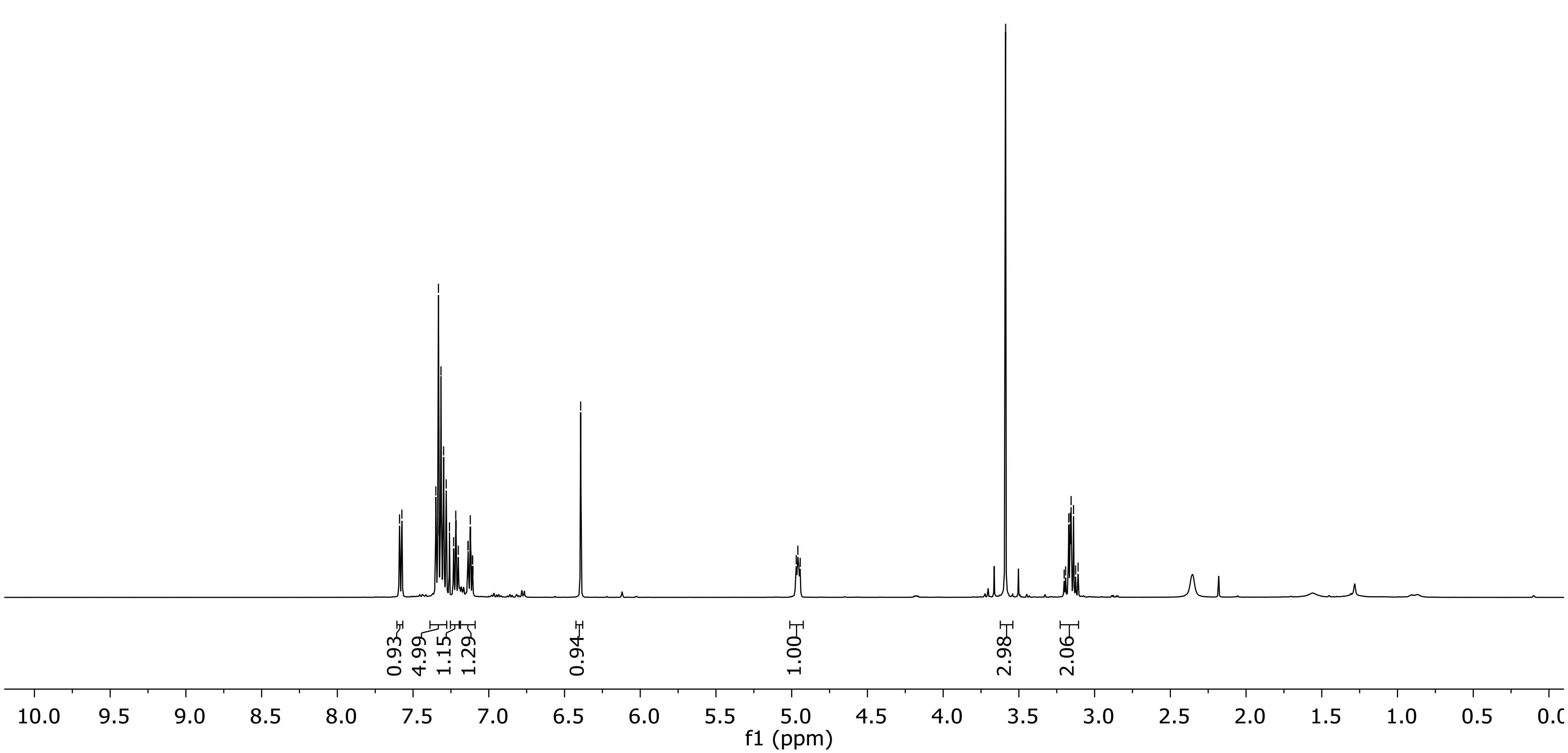

$^{13}C\{^{1}H\}$-NMR (300 MHz, $CDCl_3$)

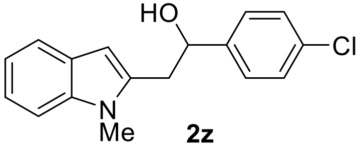

2z

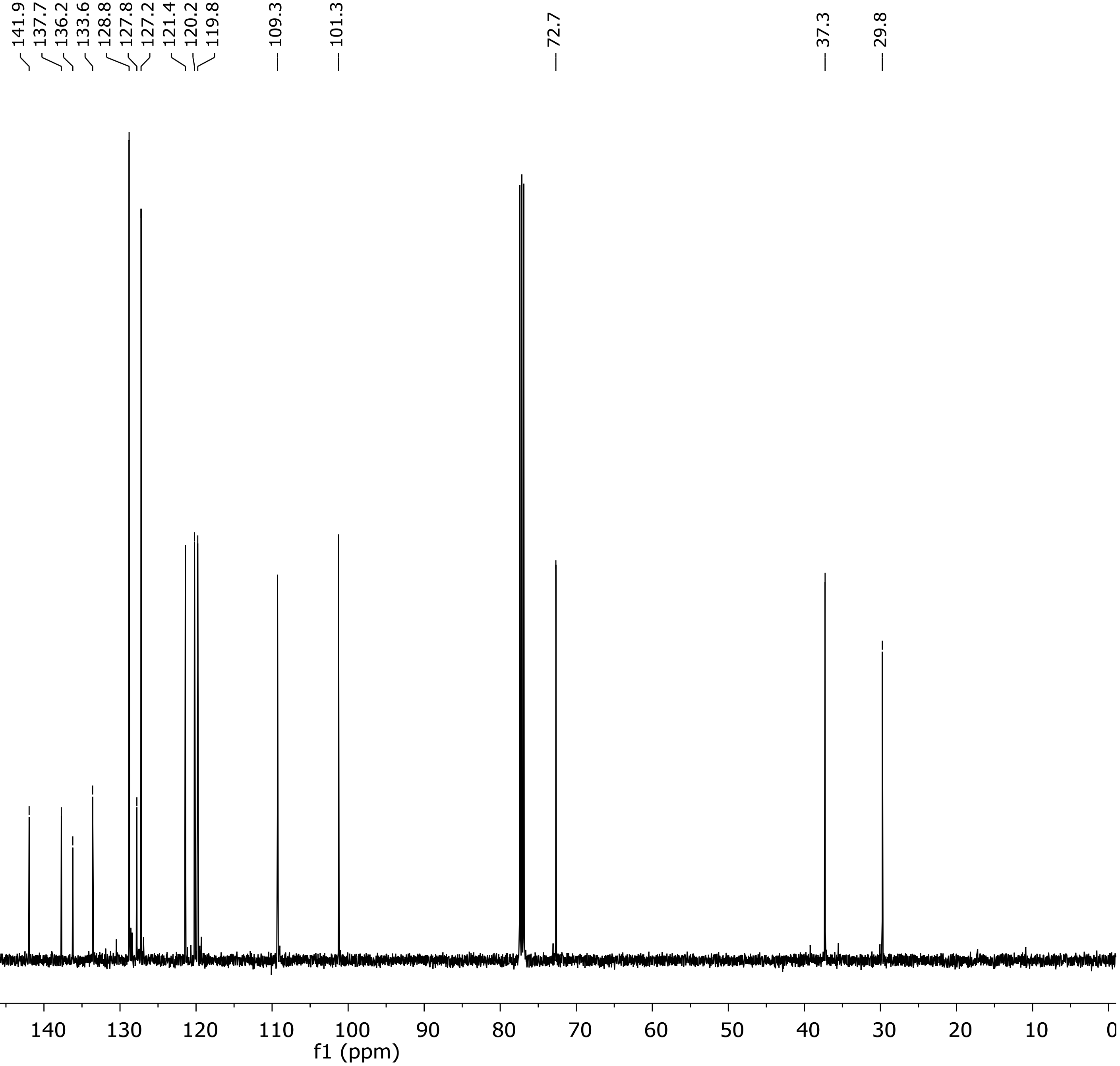

^{1}H-NMR (126 MHz, $CDCl_3$)

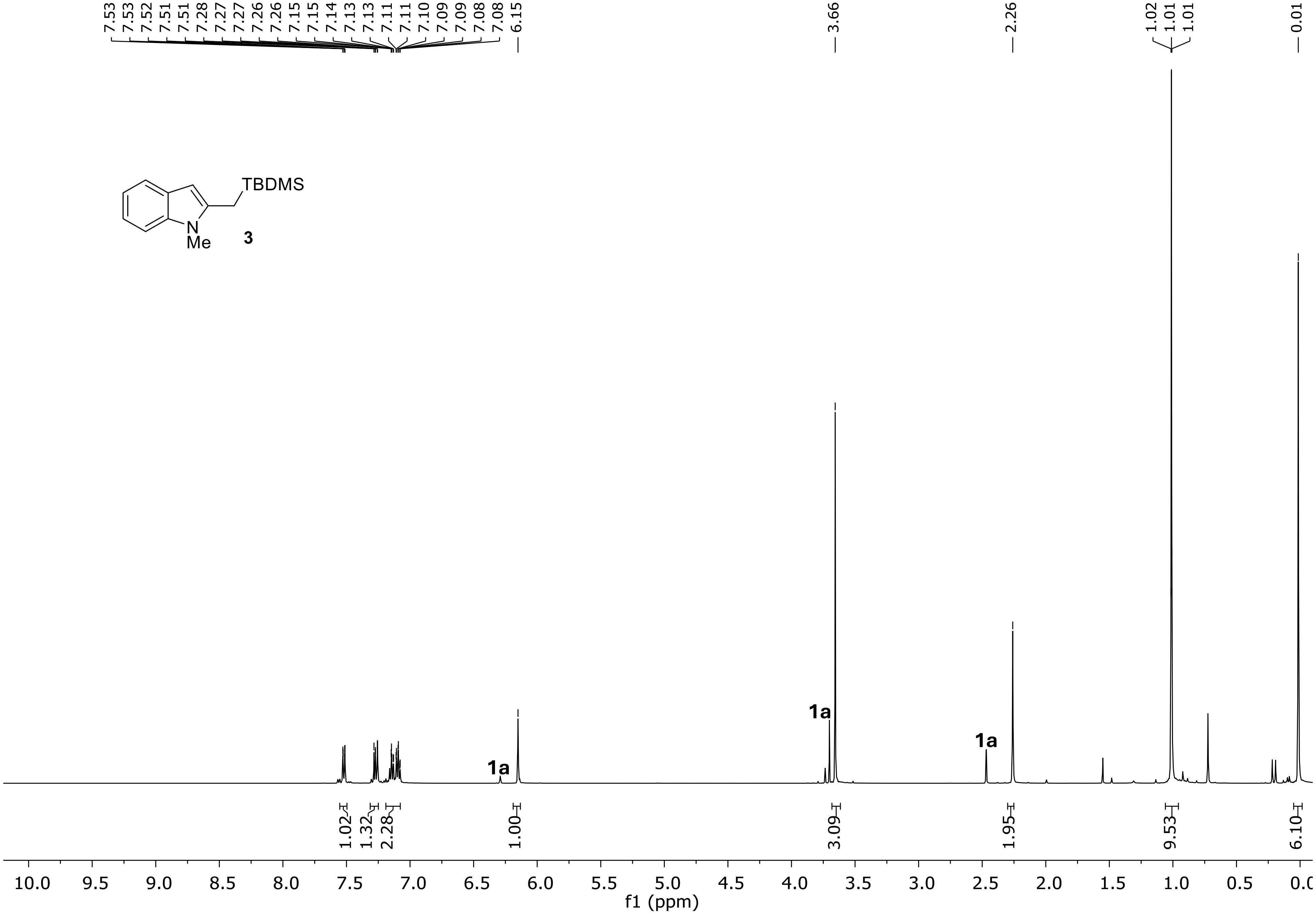

$^{13}C\{^1H\}$-NMR (500 MHz, $CDCl_3$)

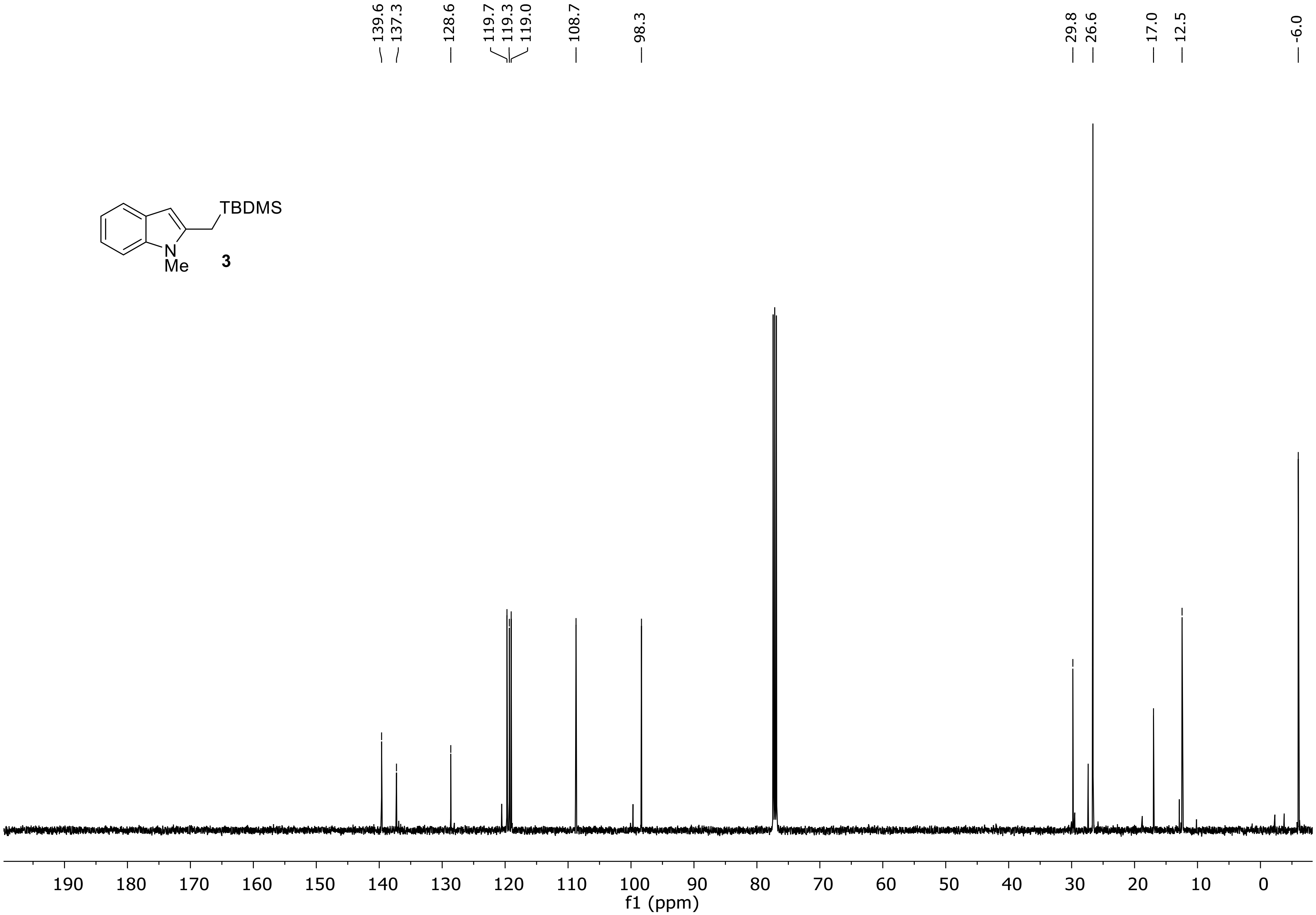

^{1}H-NMR (126 MHz, $CDCl_3$)

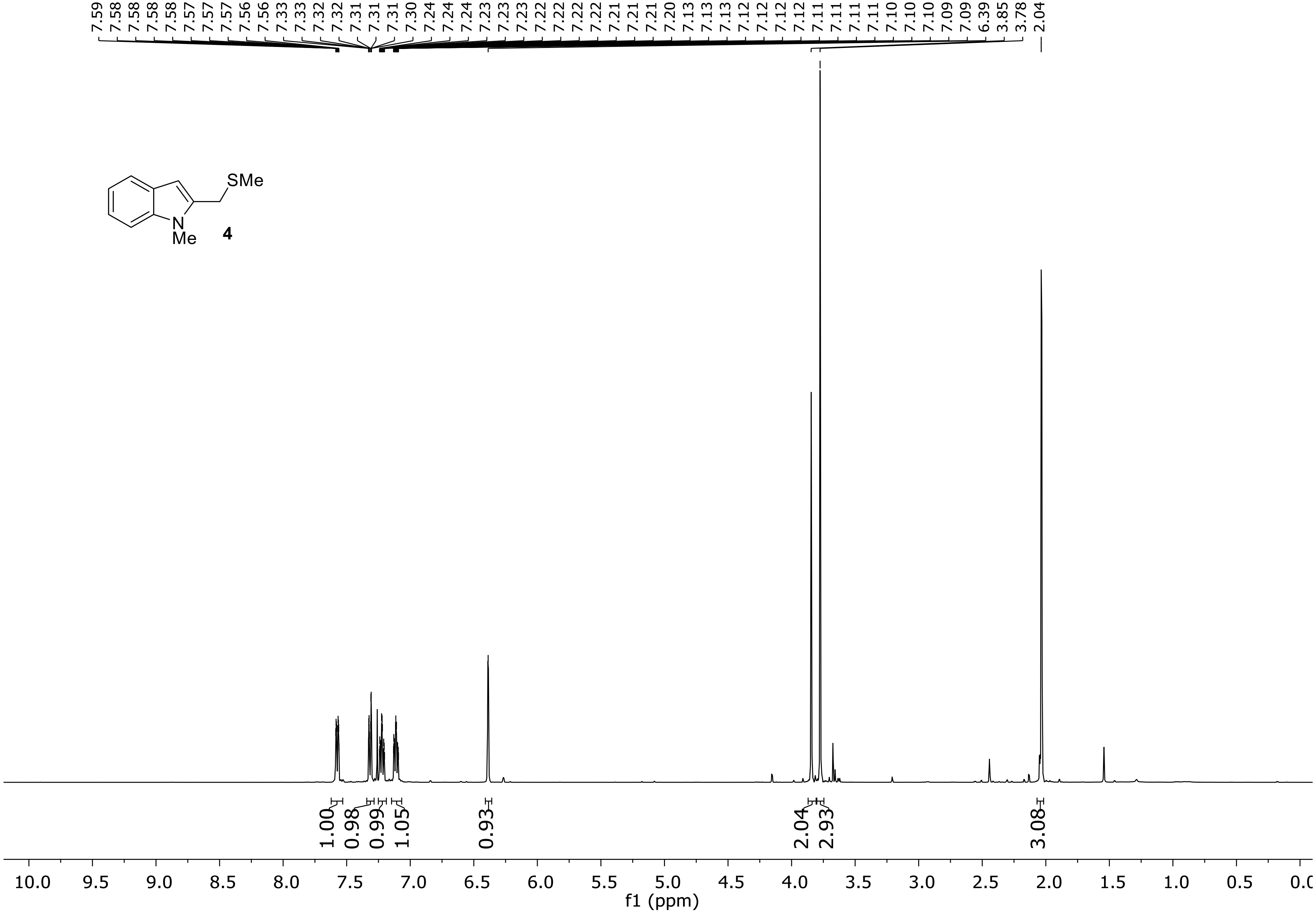

$^{13}C\{^1H\}$-NMR (500 MHz, $CDCl_3$)

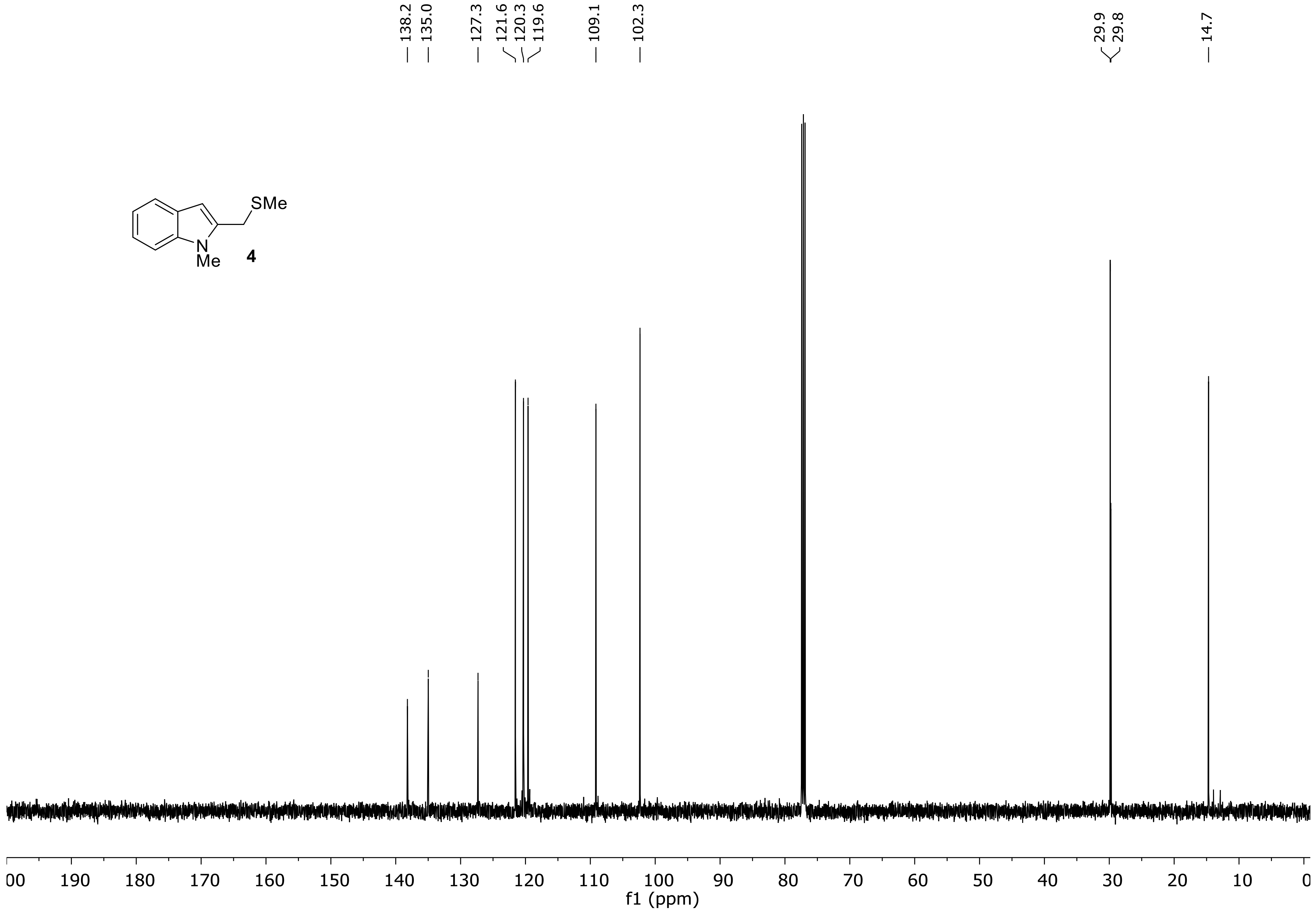

^{1}H-NMR (75.4 MHz, $CDCl_3$)

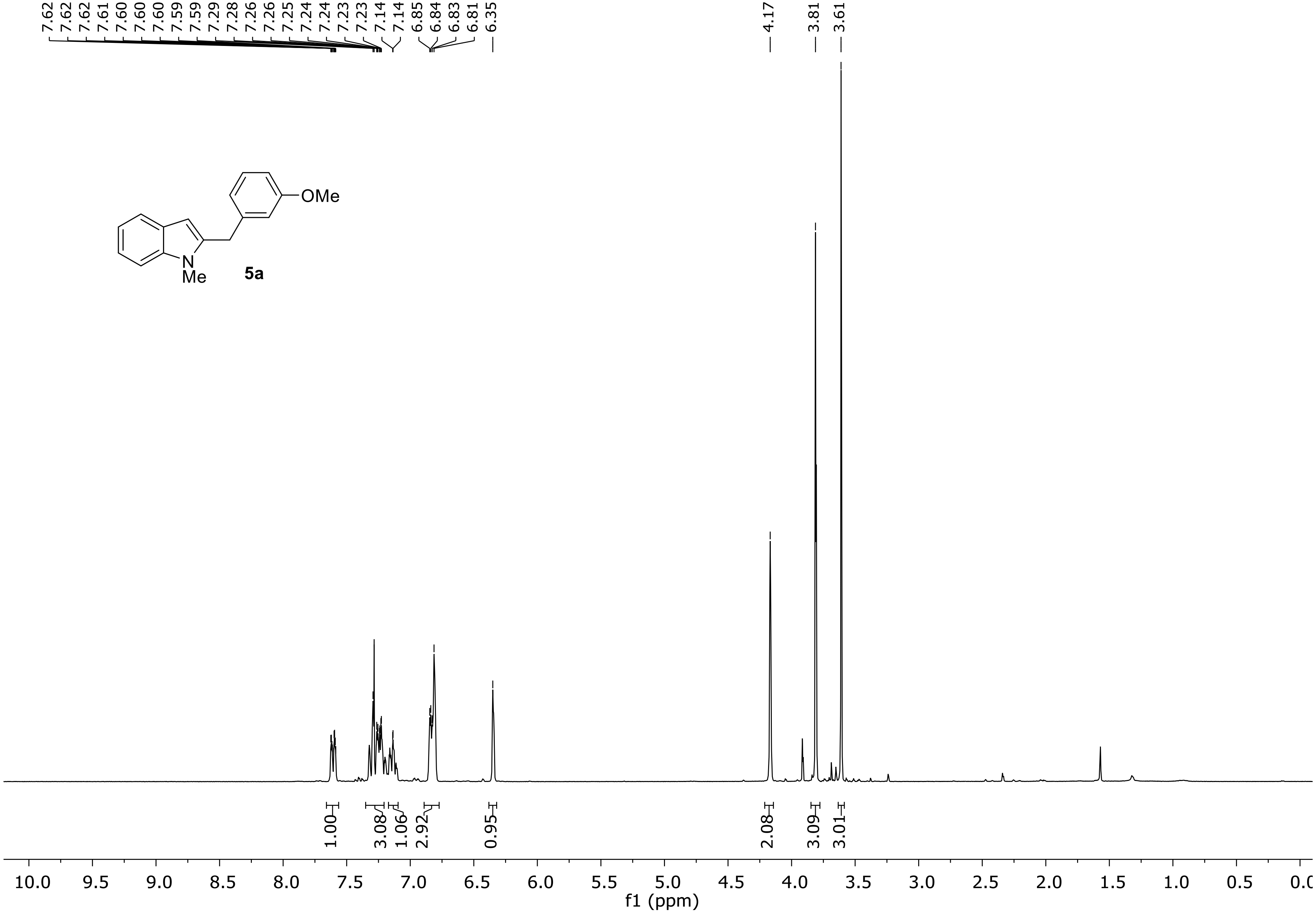

$^{13}C\{^{1}H\}$-NMR (300 MHz, $CDCl_3$)

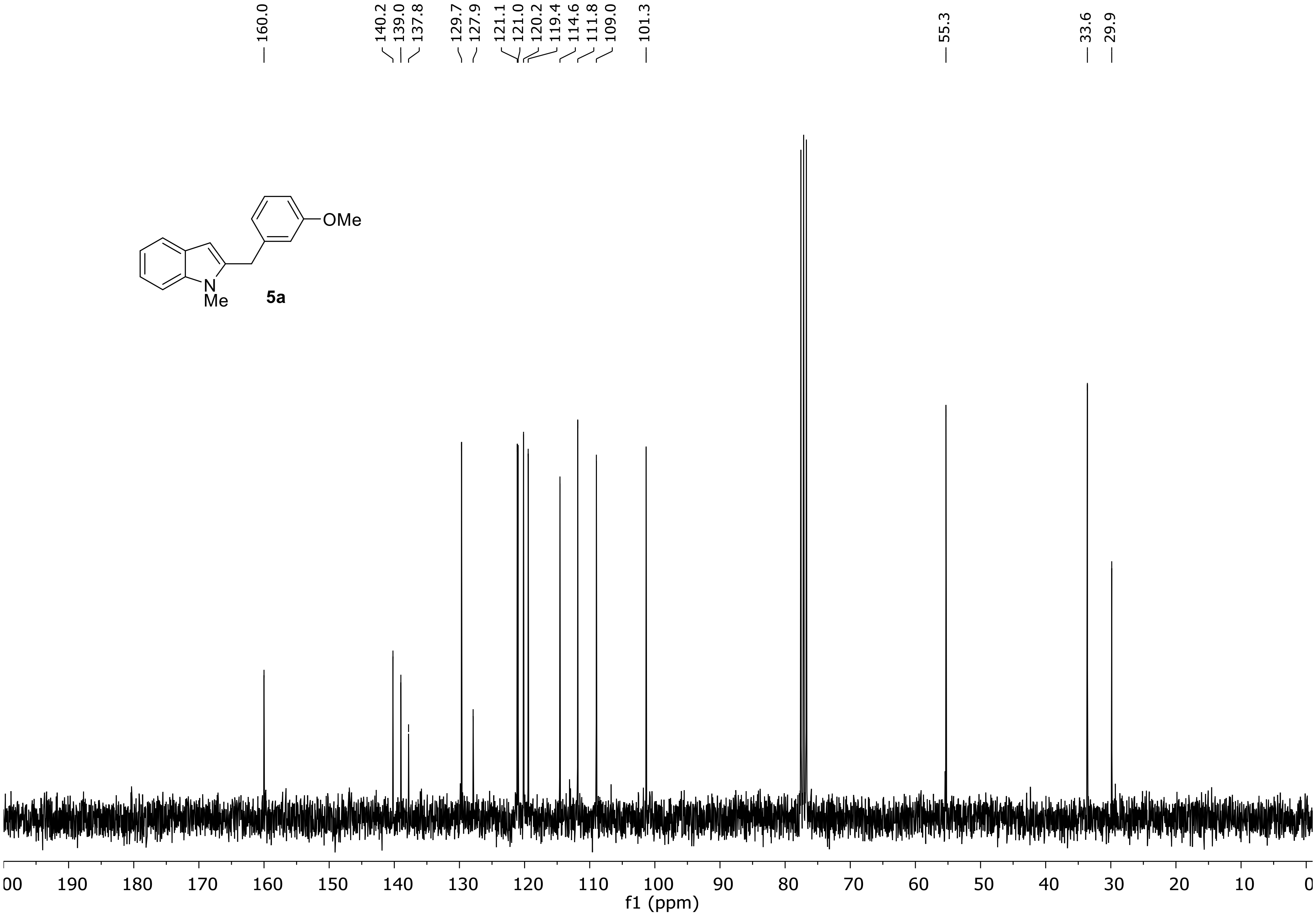

^{1}H-NMR (75.4 MHz, $CDCl_3$)

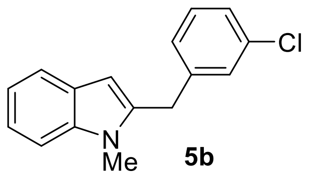

5b

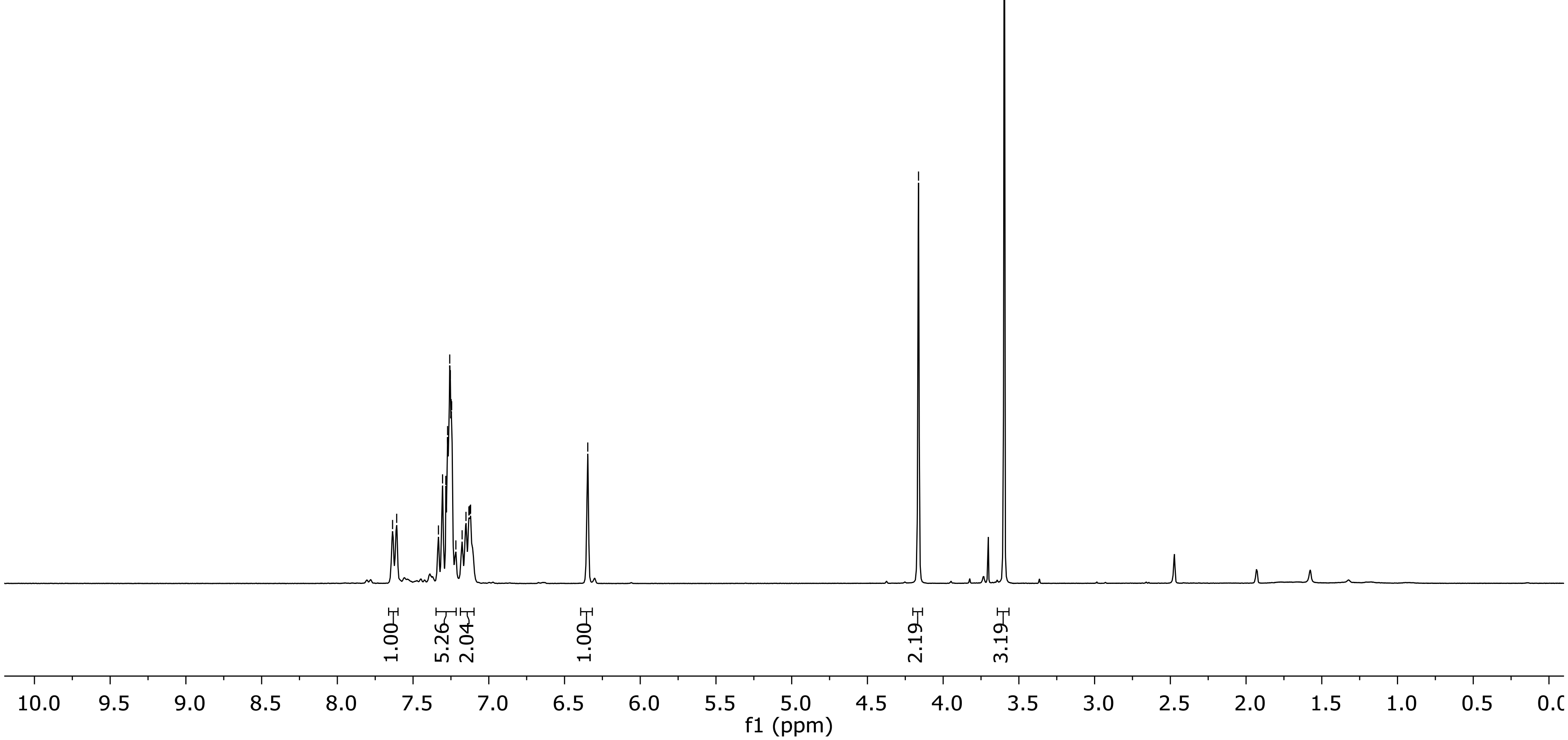

$^{13}C\{^{1}H\}$-NMR (300 MHz, $CDCl_3$)

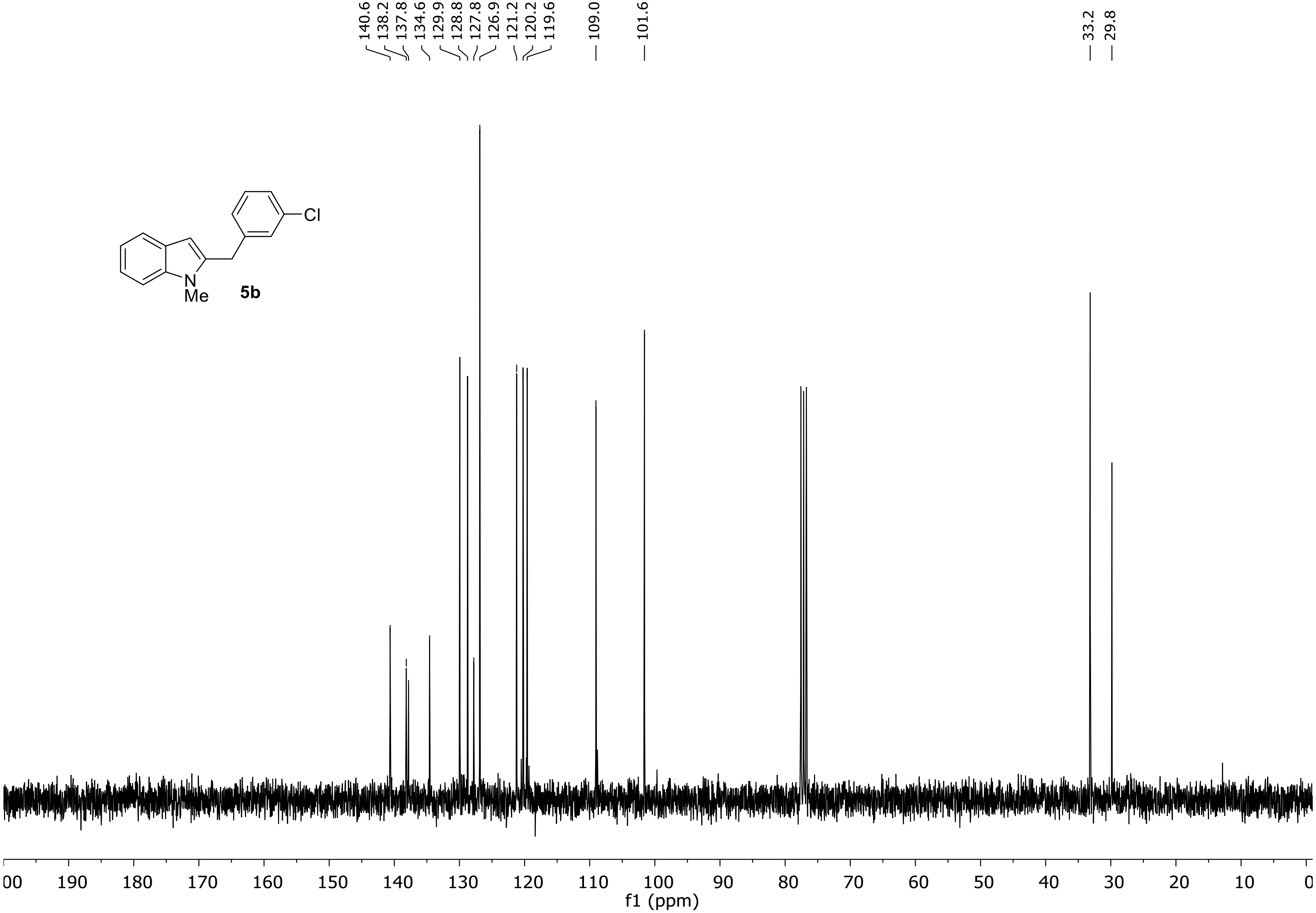

^{1}H-NMR (75.4 MHz, $CDCl_3$)

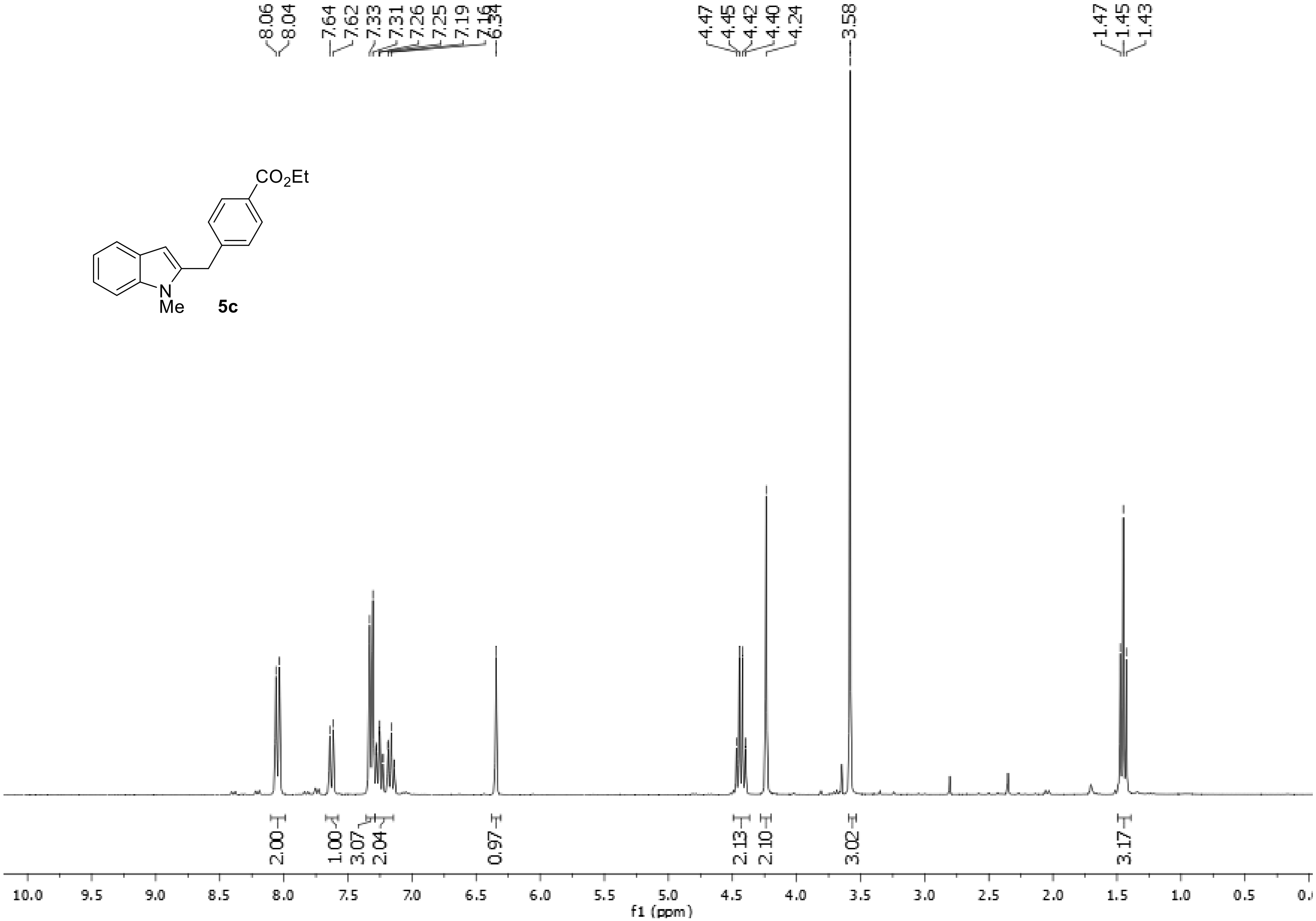

$^{13}C\{^{1}H\}$-NMR (300 MHz, $CDCl_3$)

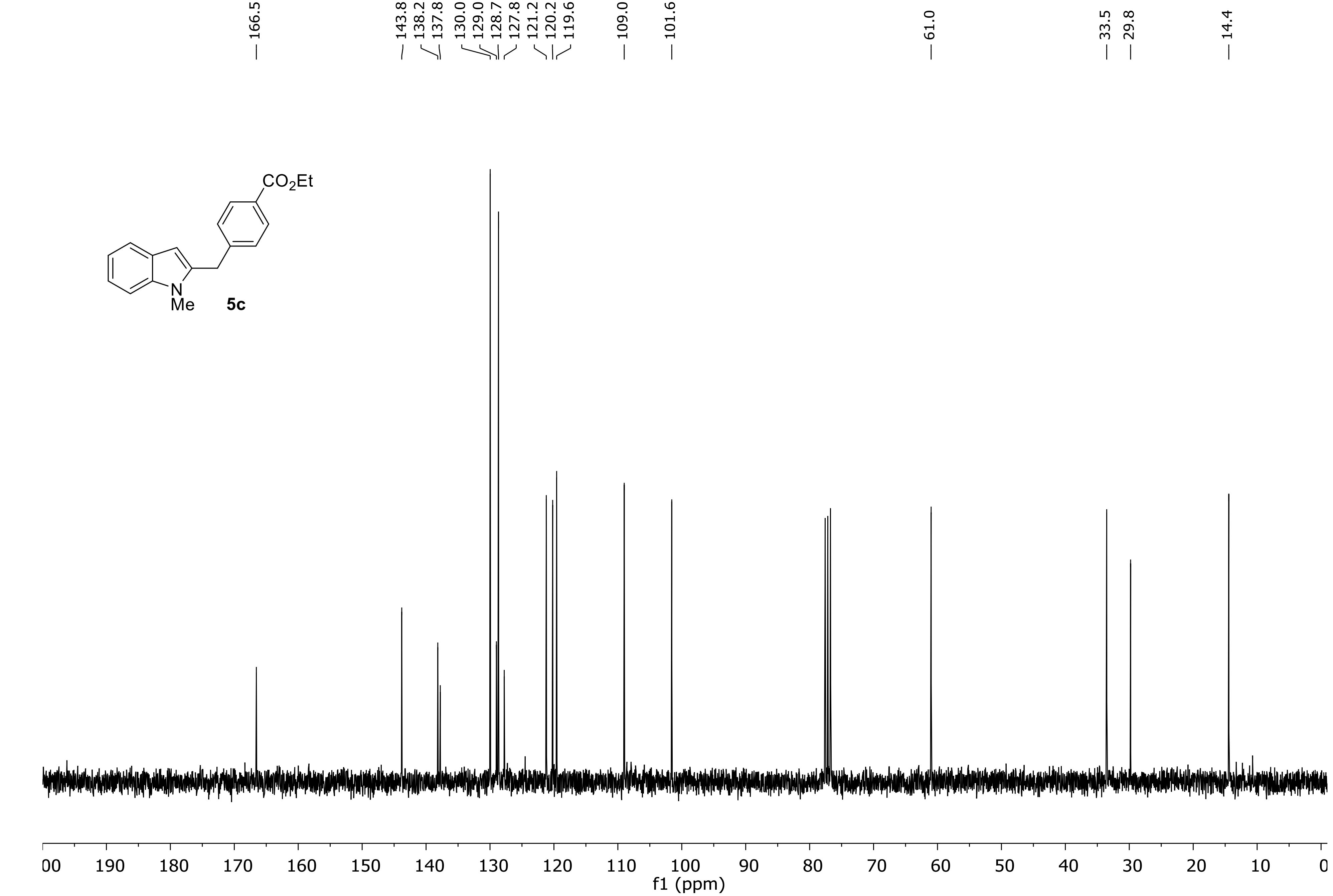

^{1}H-NMR (75.4 MHz, $CDCl_3$)

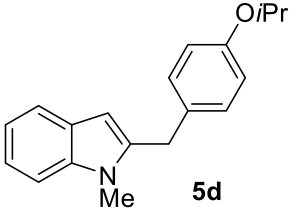

5d

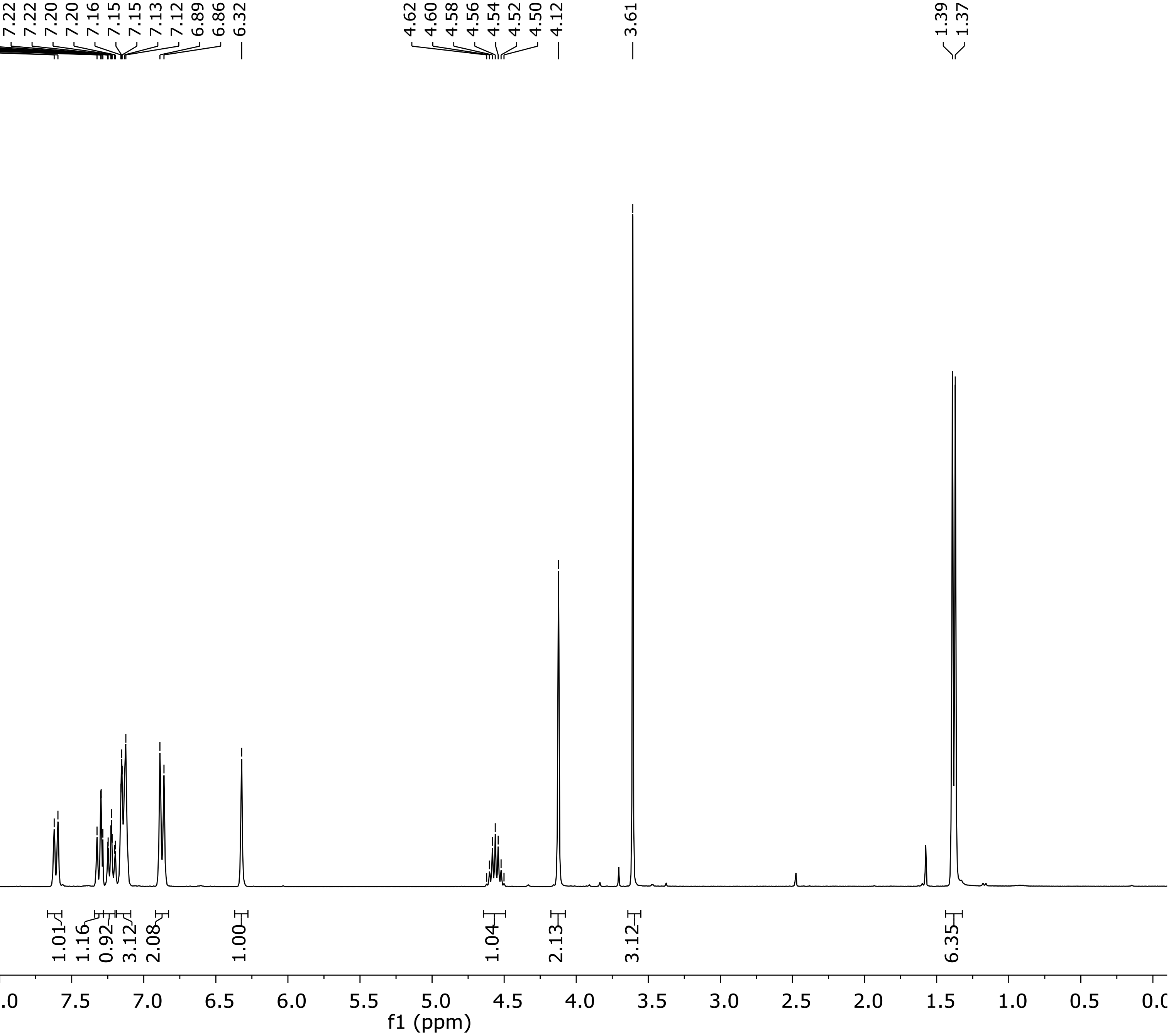

$^{13}C\{^{1}H\}$-NMR (300 MHz, $CDCl_3$)

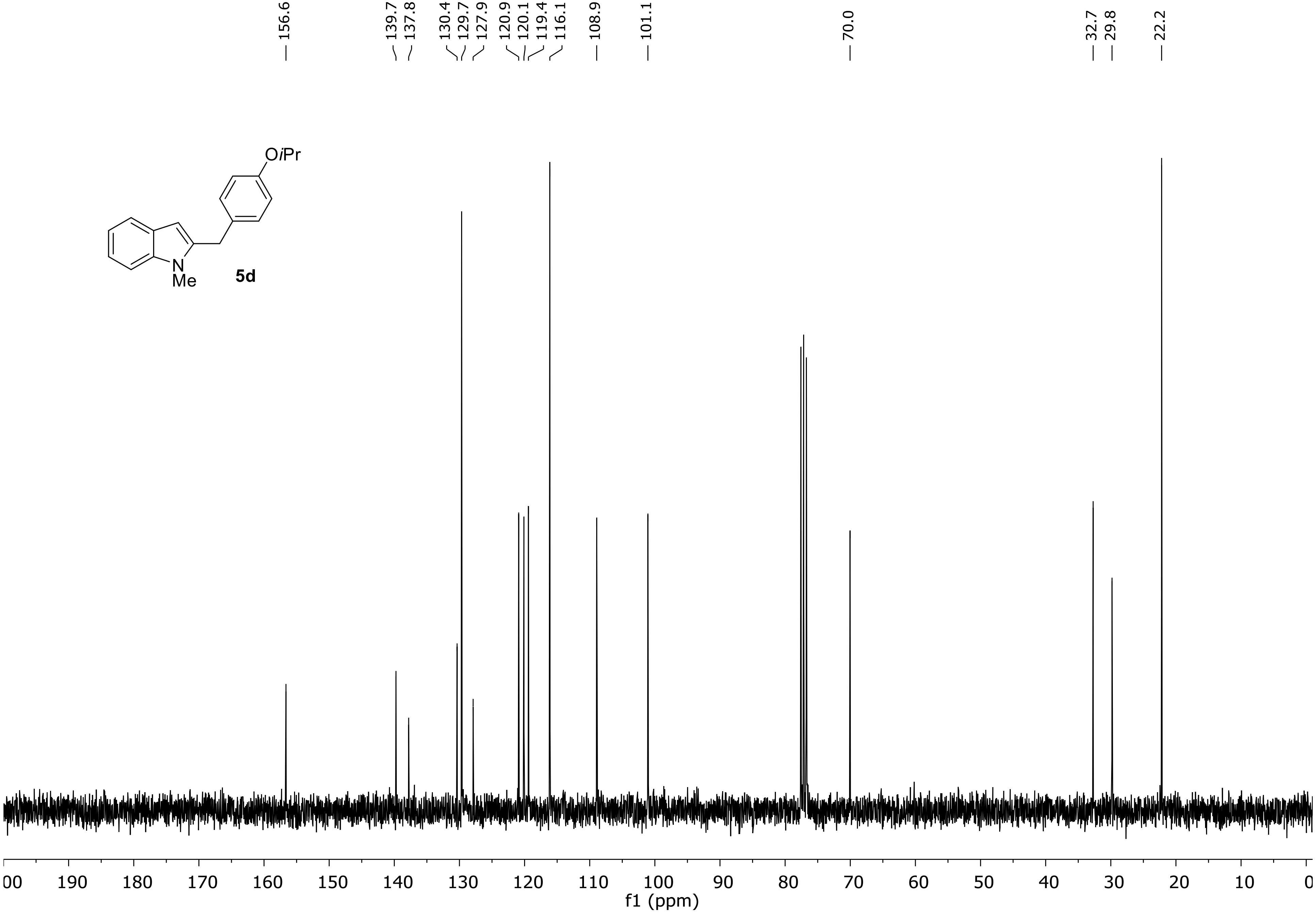

^{1}H-NMR (75.4 MHz, $CDCl_3$)

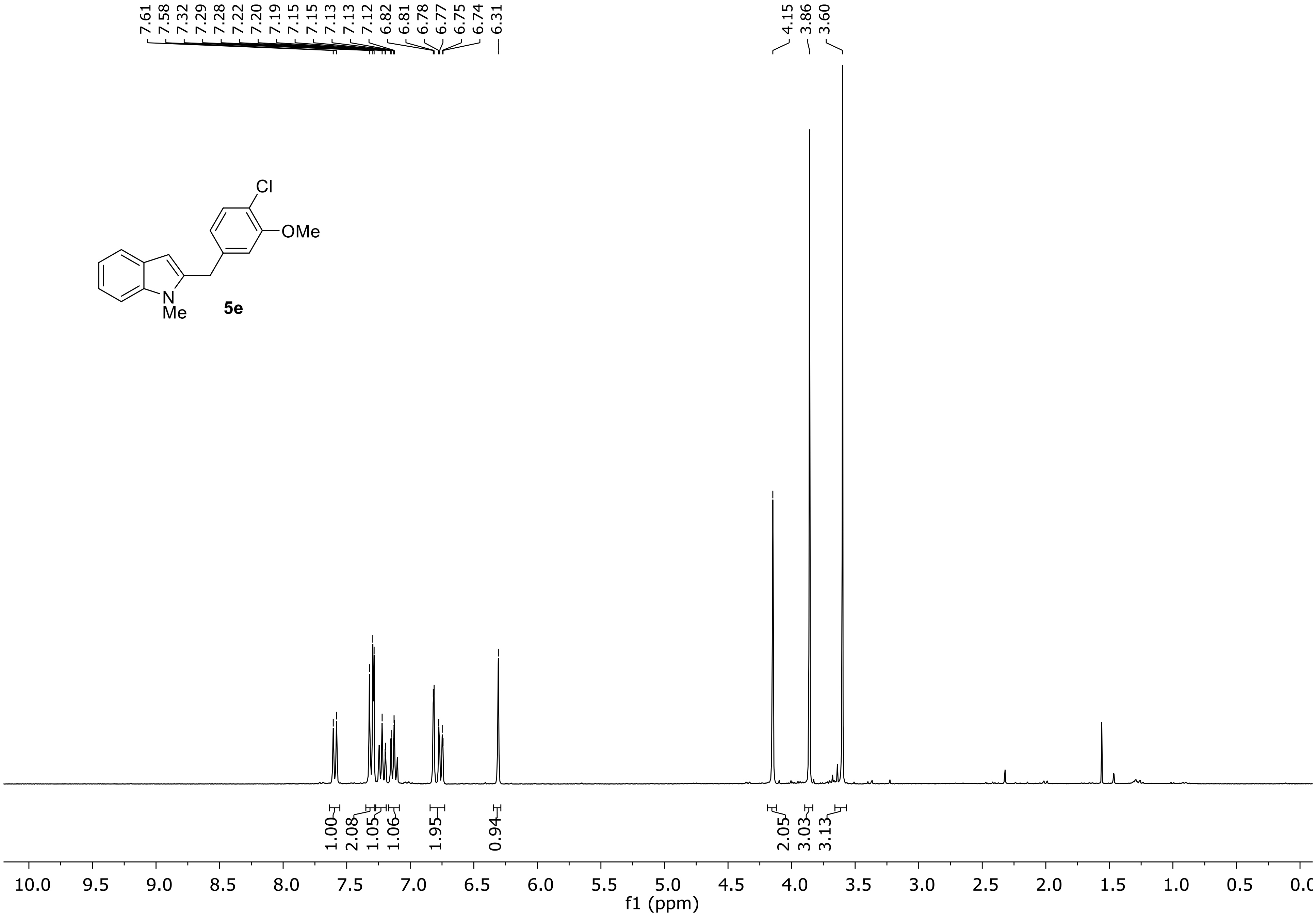

5e

$^{13}C\{^1H\}$-NMR (300 MHz, $CDCl_3$)

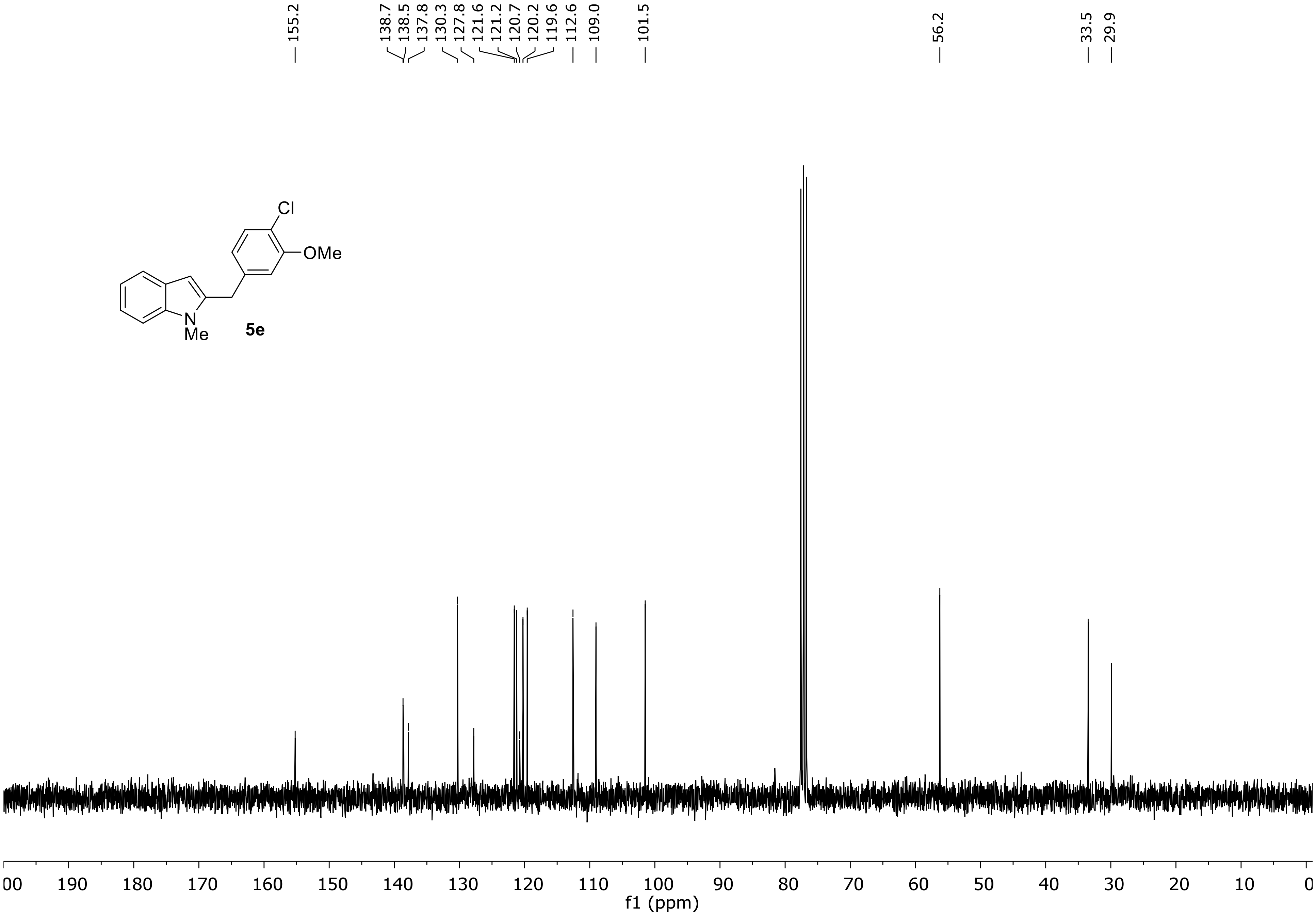

5e

^{1}H-NMR (75.4 MHz, $CDCl_3$)

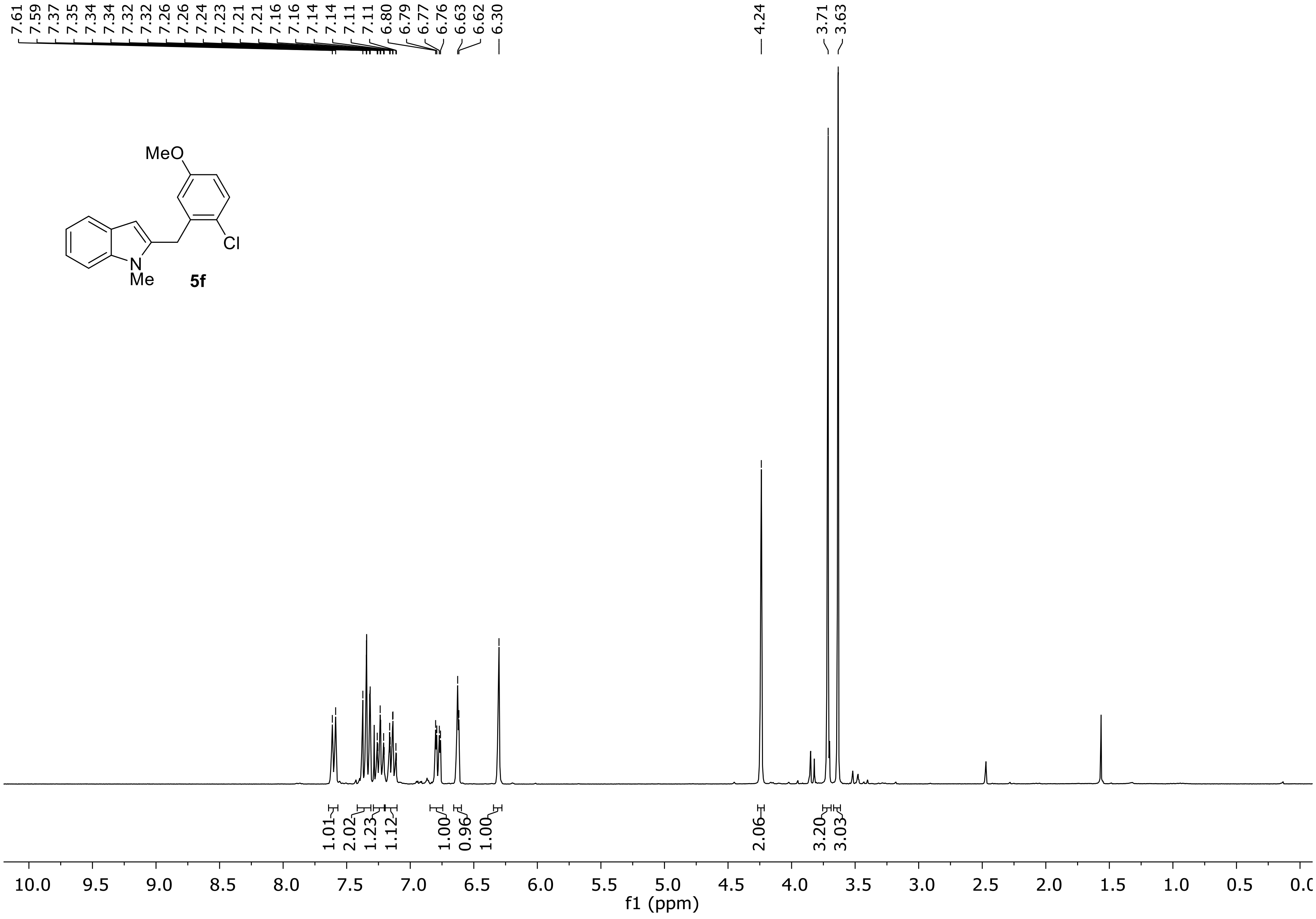

$^{13}C\{^1H\}$-NMR (300 MHz, $CDCl_3$)

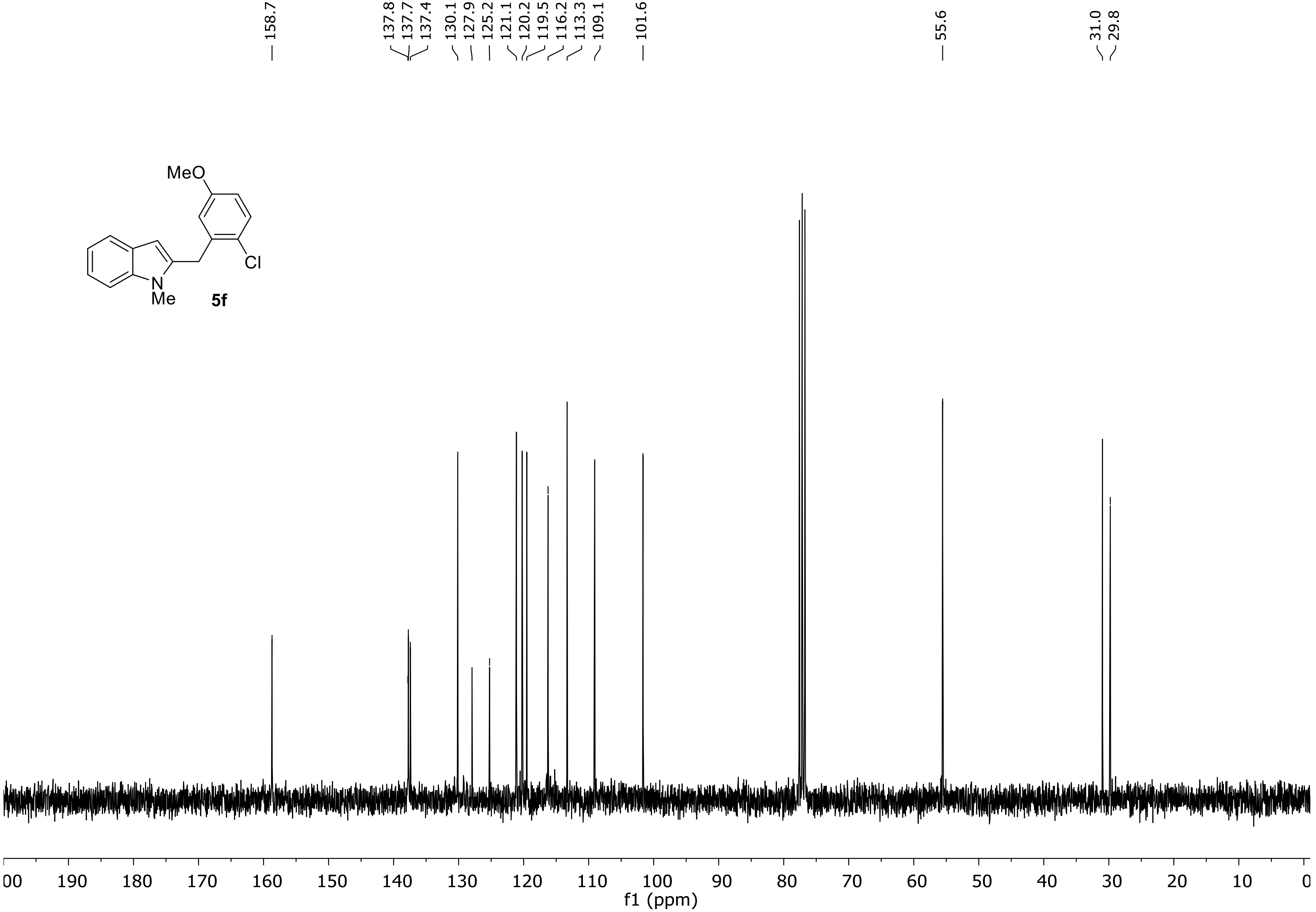

^{1}H-NMR (75.4 MHz, $CDCl_3$)

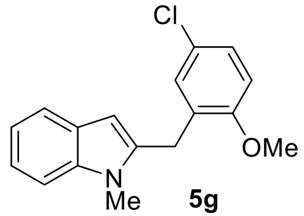

5g

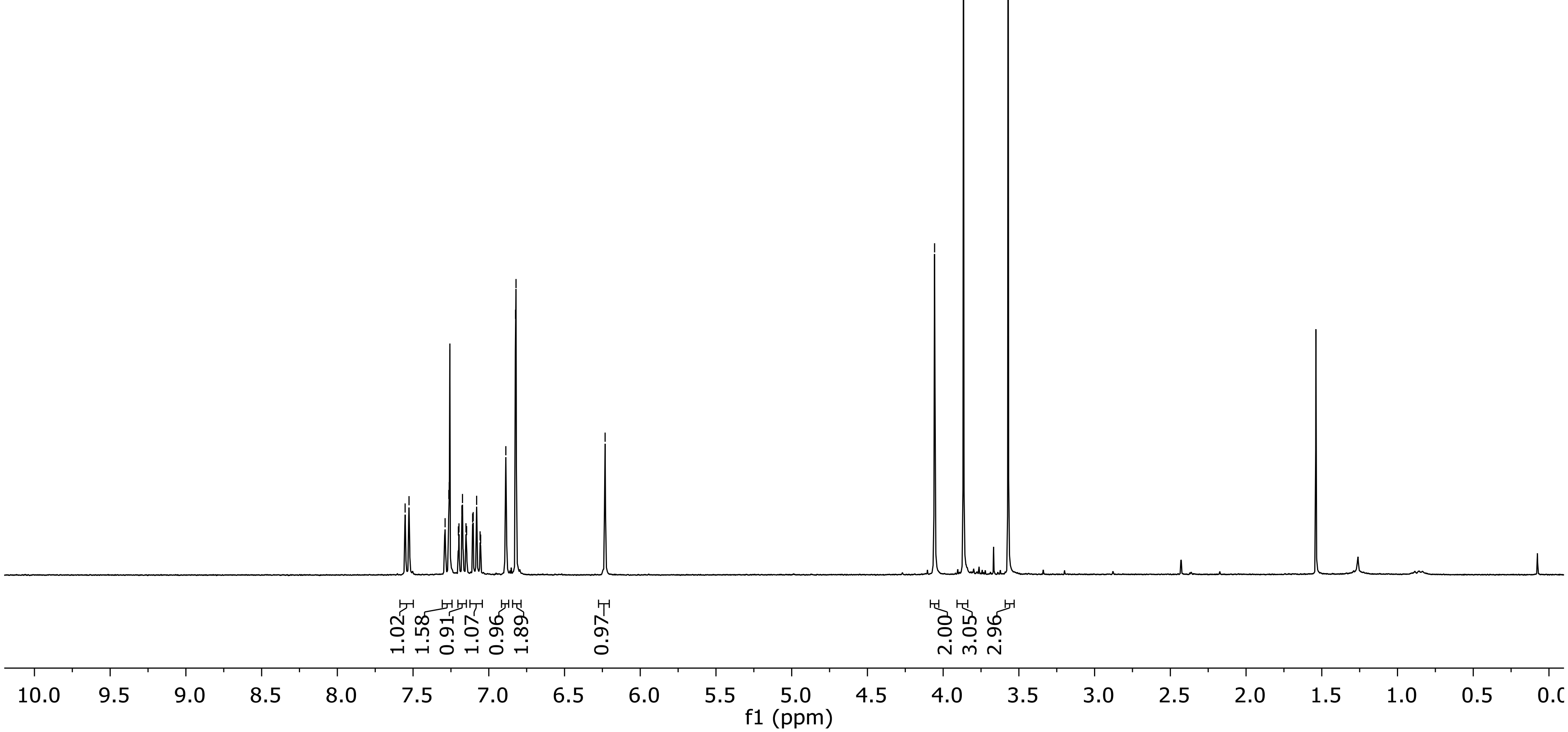

$^{13}C\{^1H\}$-NMR (300 MHz, $CDCl_3$)

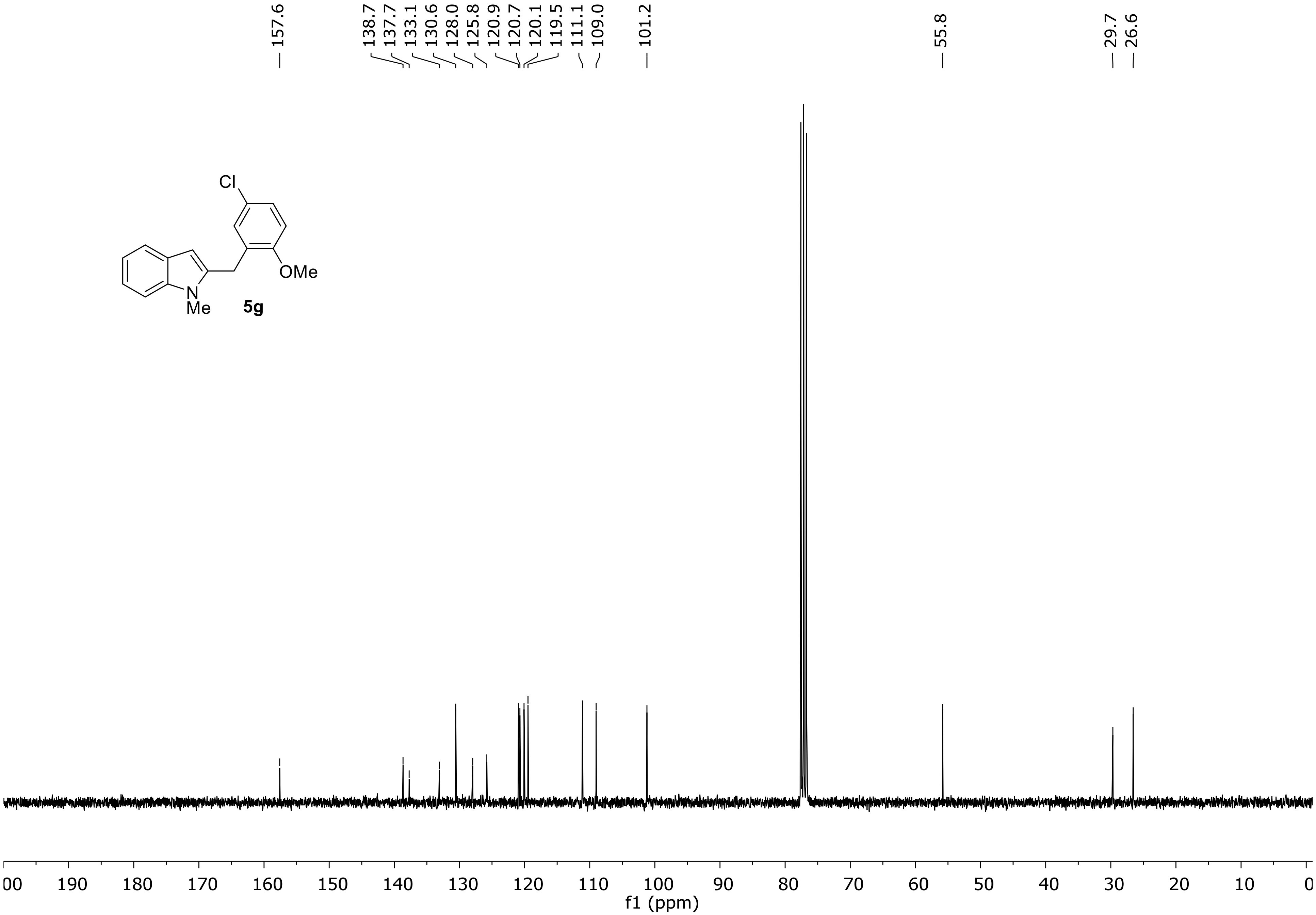

^{1}H-NMR (75.4 MHz, $CDCl_3$)

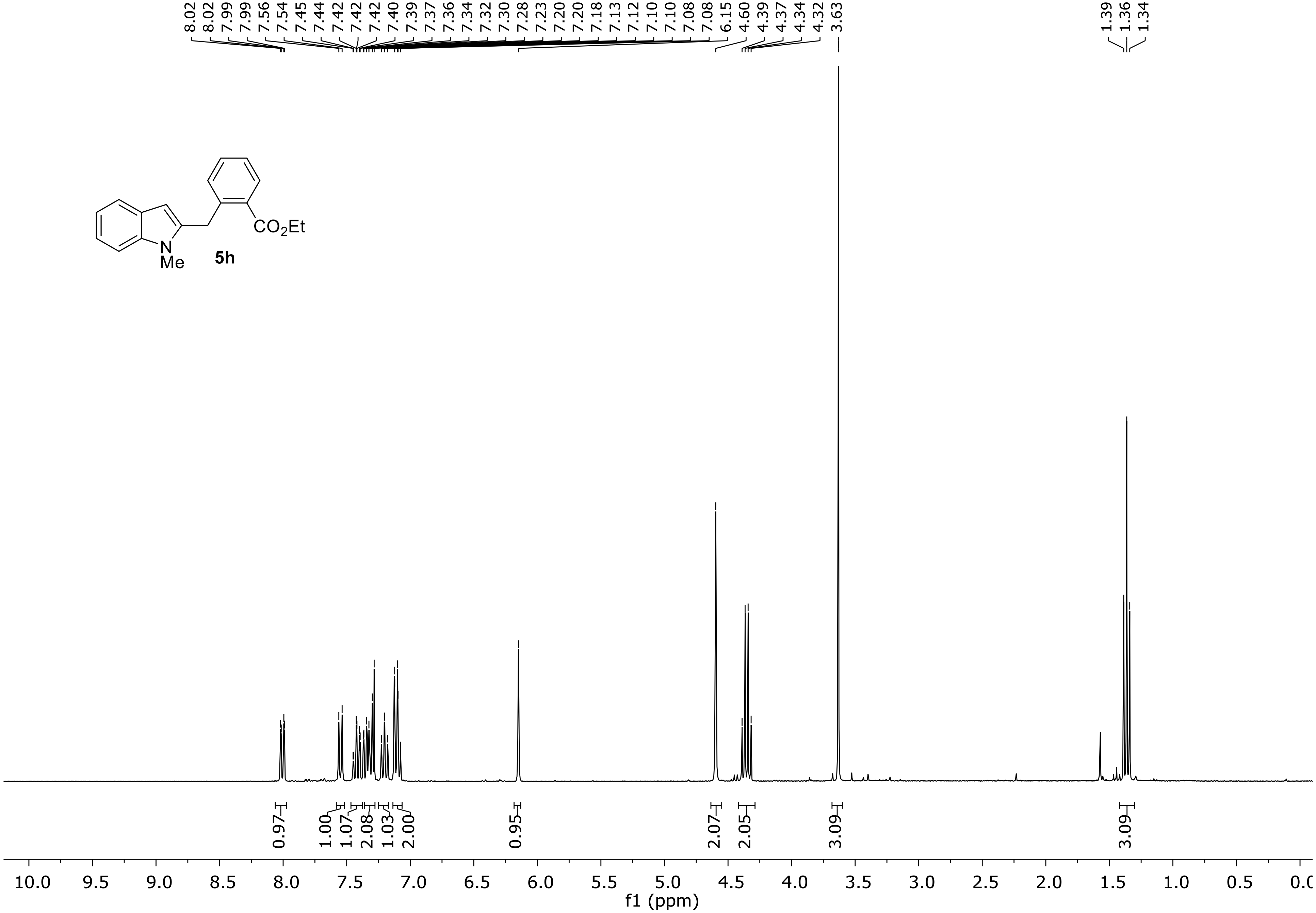

$^{13}C\{^1H\}$-NMR (300 MHz, $CDCl_3$)

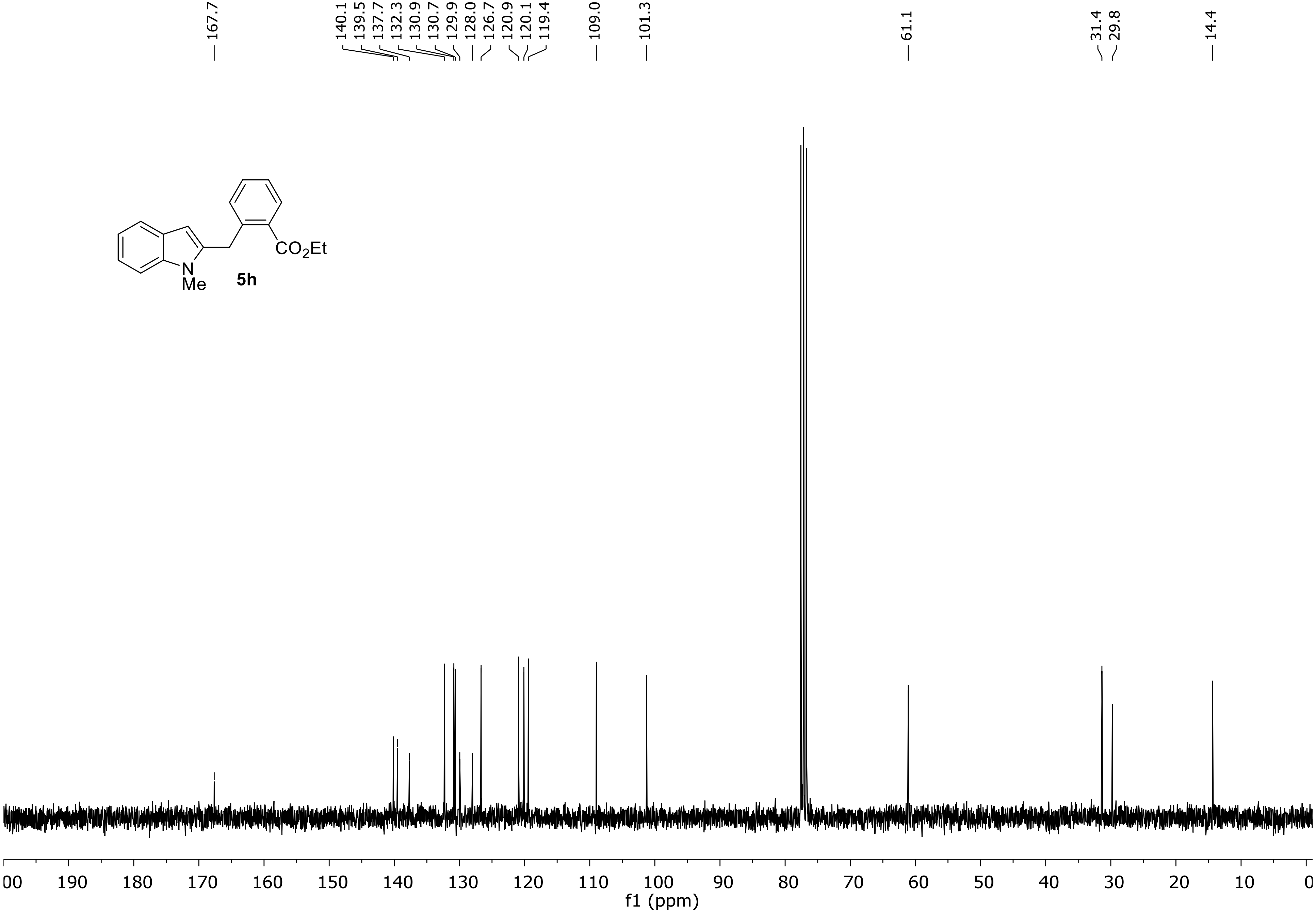

^{1}H-NMR (75.4 MHz, $CDCl_3$)

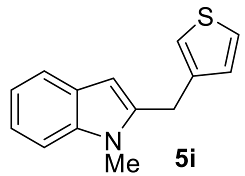

5i

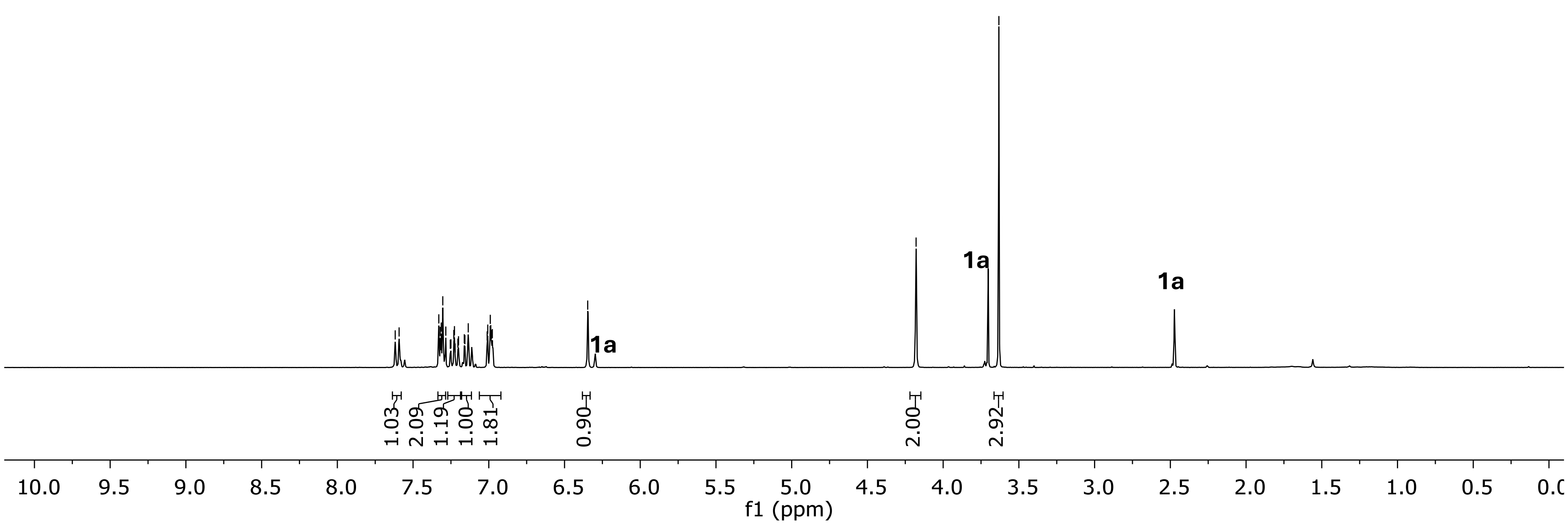

$^{13}C\{^{1}H\}$-NMR (300 MHz, $CDCl_3$)

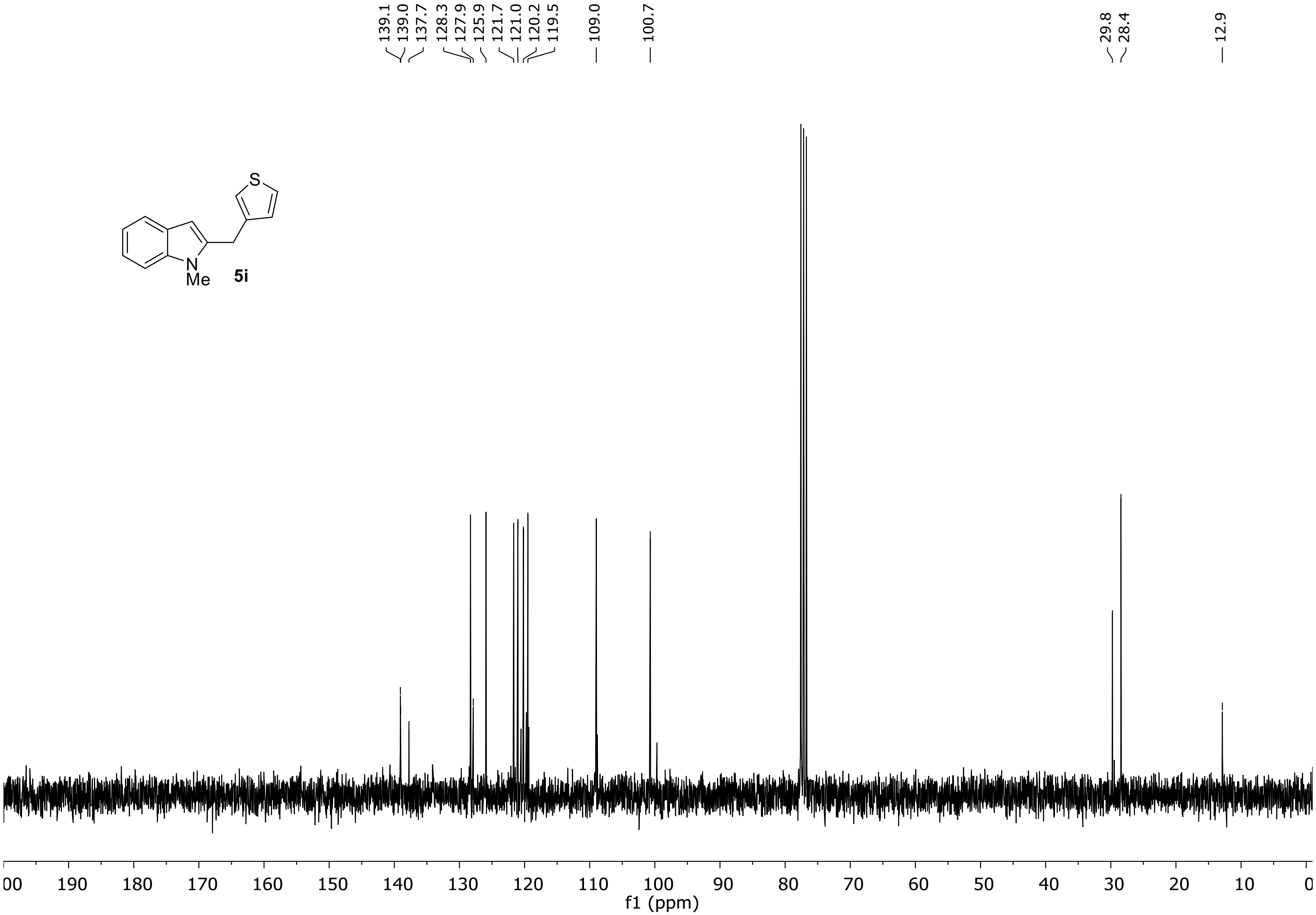

^{1}H-NMR (126 MHz, $CDCl_3$)

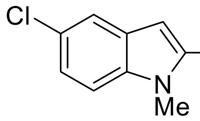

1b-D

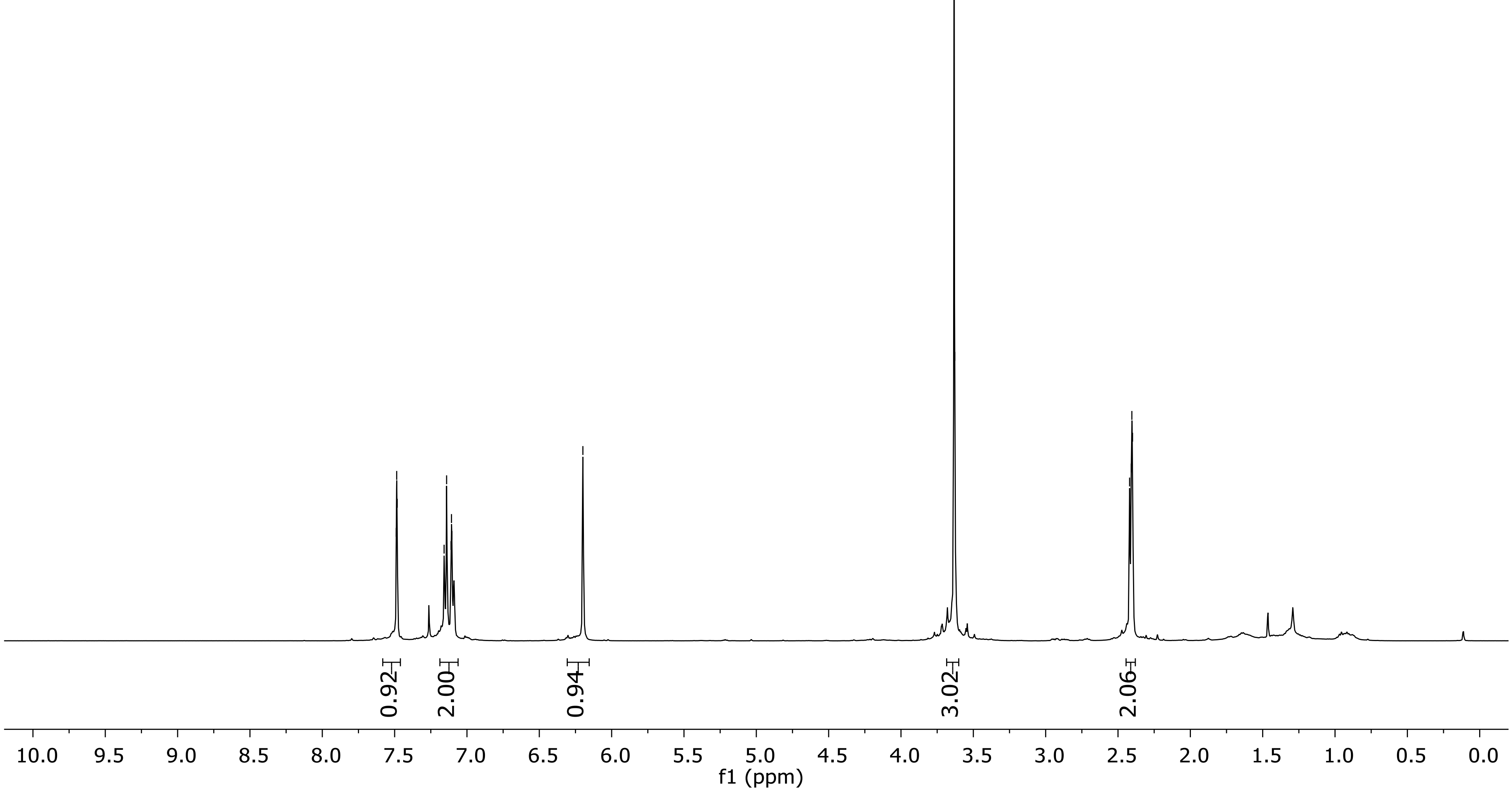

$^{13}C\{^1H\}$-NMR (500 MHz, $CDCl_3$)

1b-D

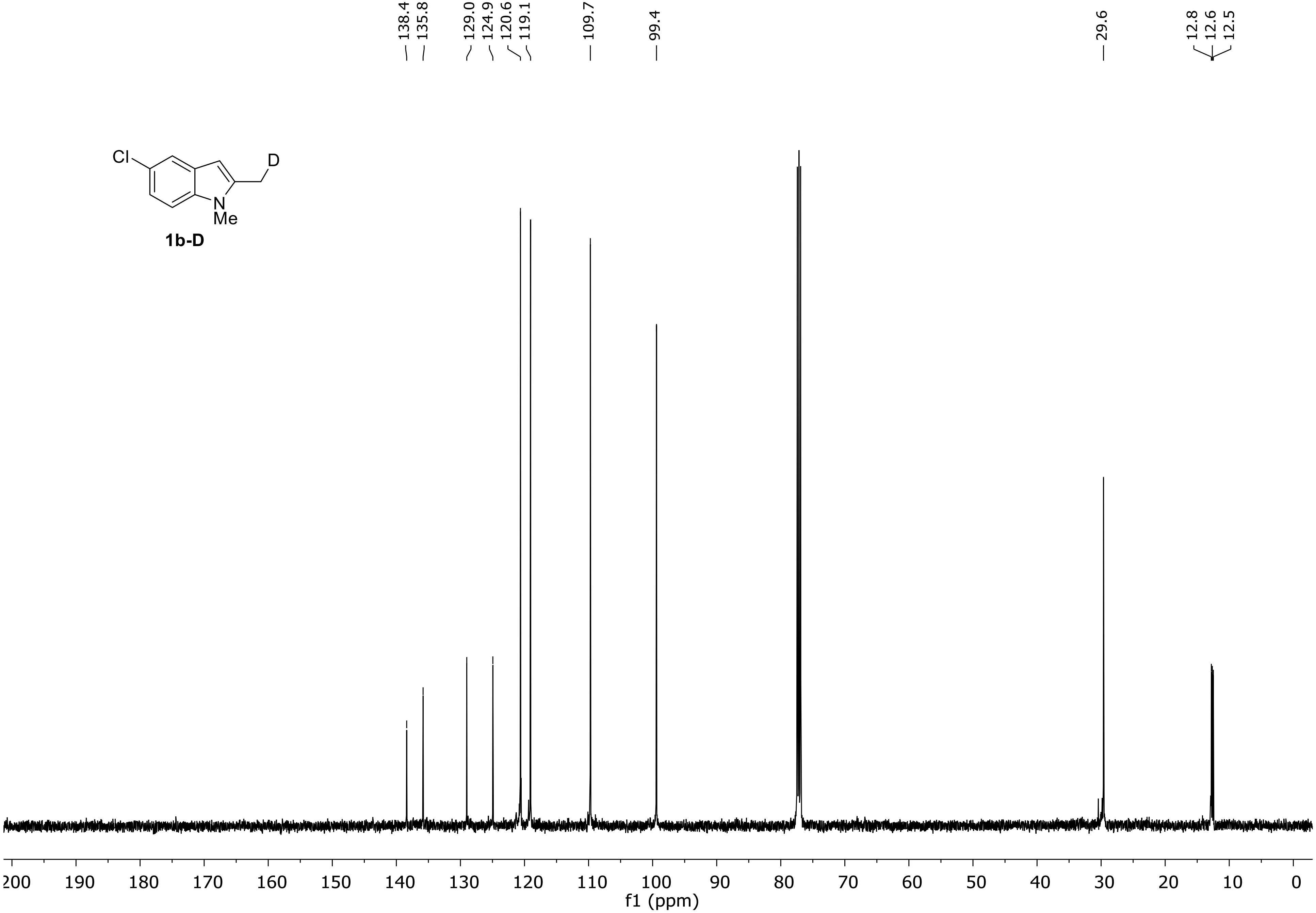

^{1}H-NMR (75.4 MHz, $CDCl_3$)

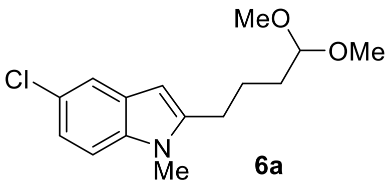

6a

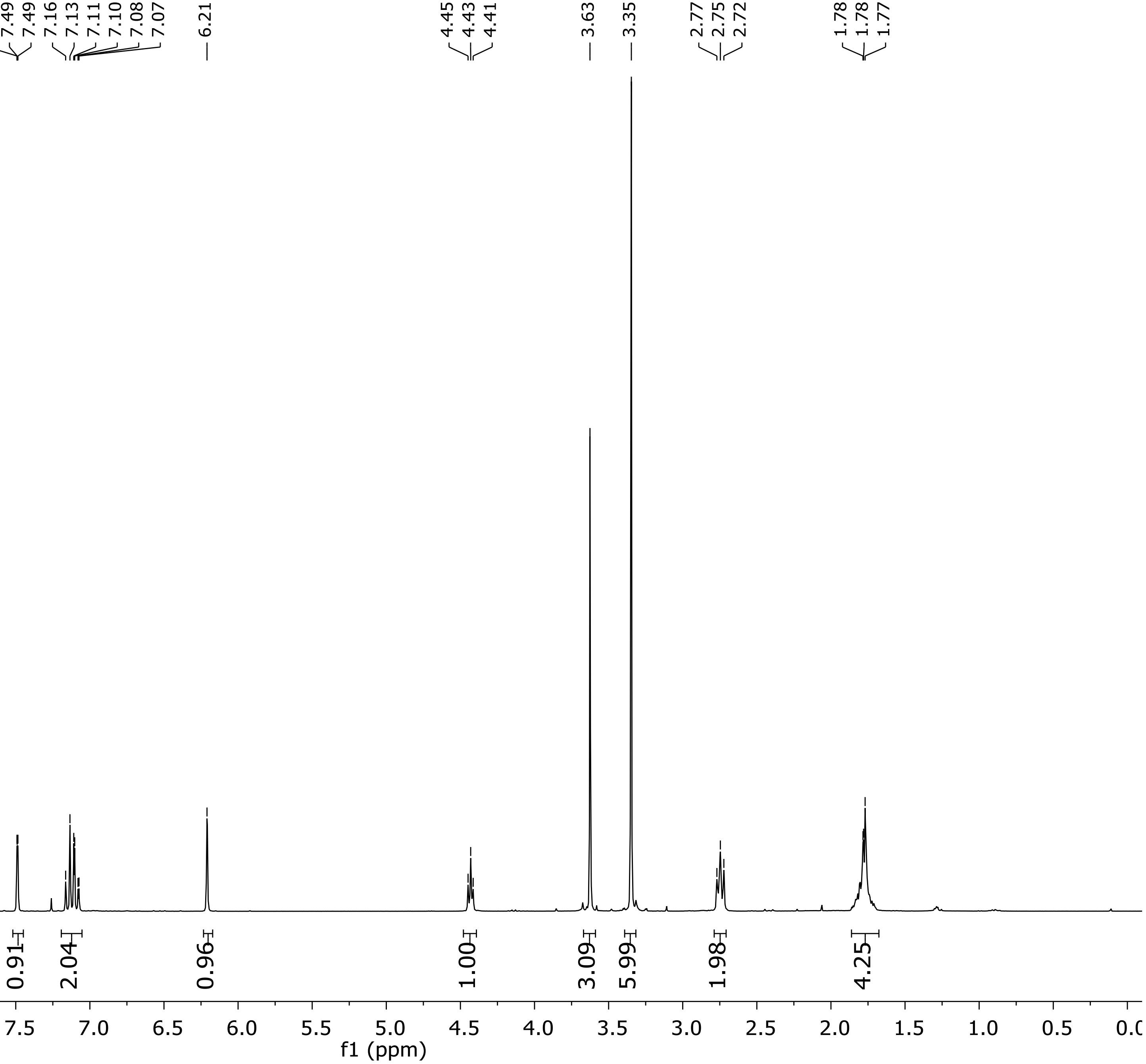

$^{13}C\{^{1}H\}$-NMR (300 MHz, $CDCl_3$)

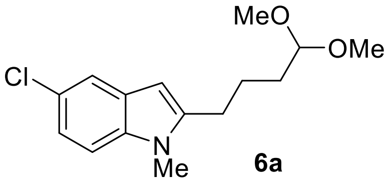

6a

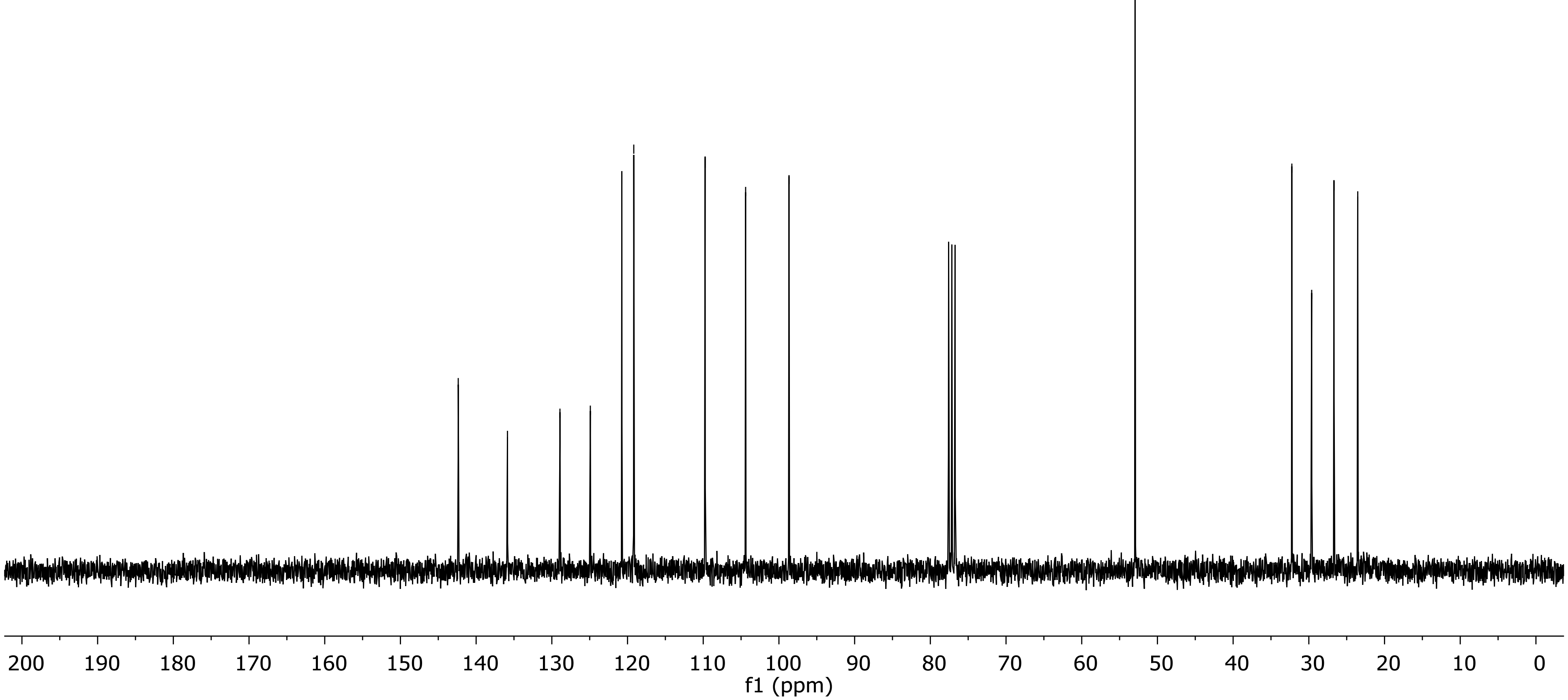

^{1}H-NMR (75.4 MHz, $CDCl_3$)

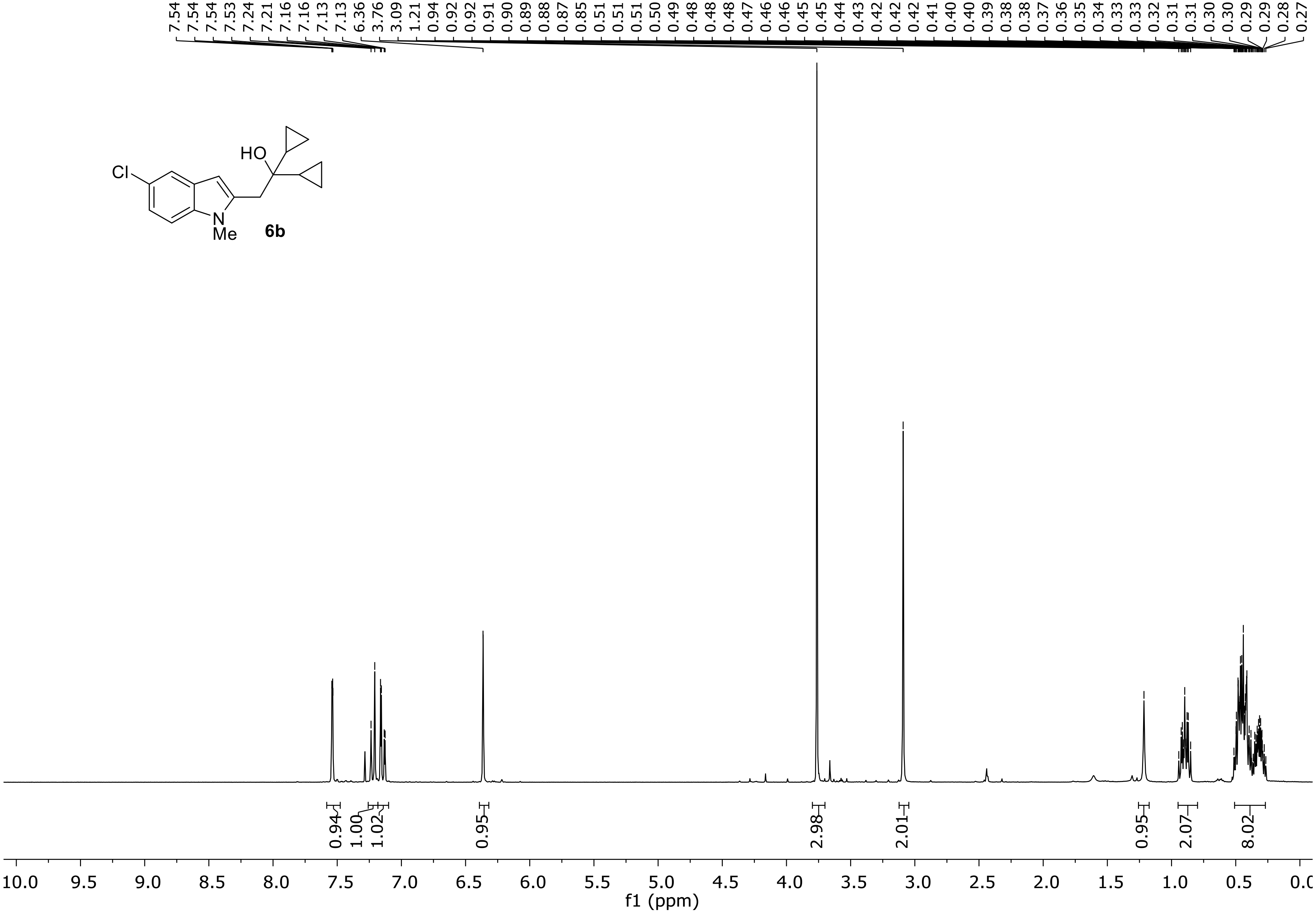

$^{13}C\{^1H\}$-NMR (300 MHz, $CDCl_3$)

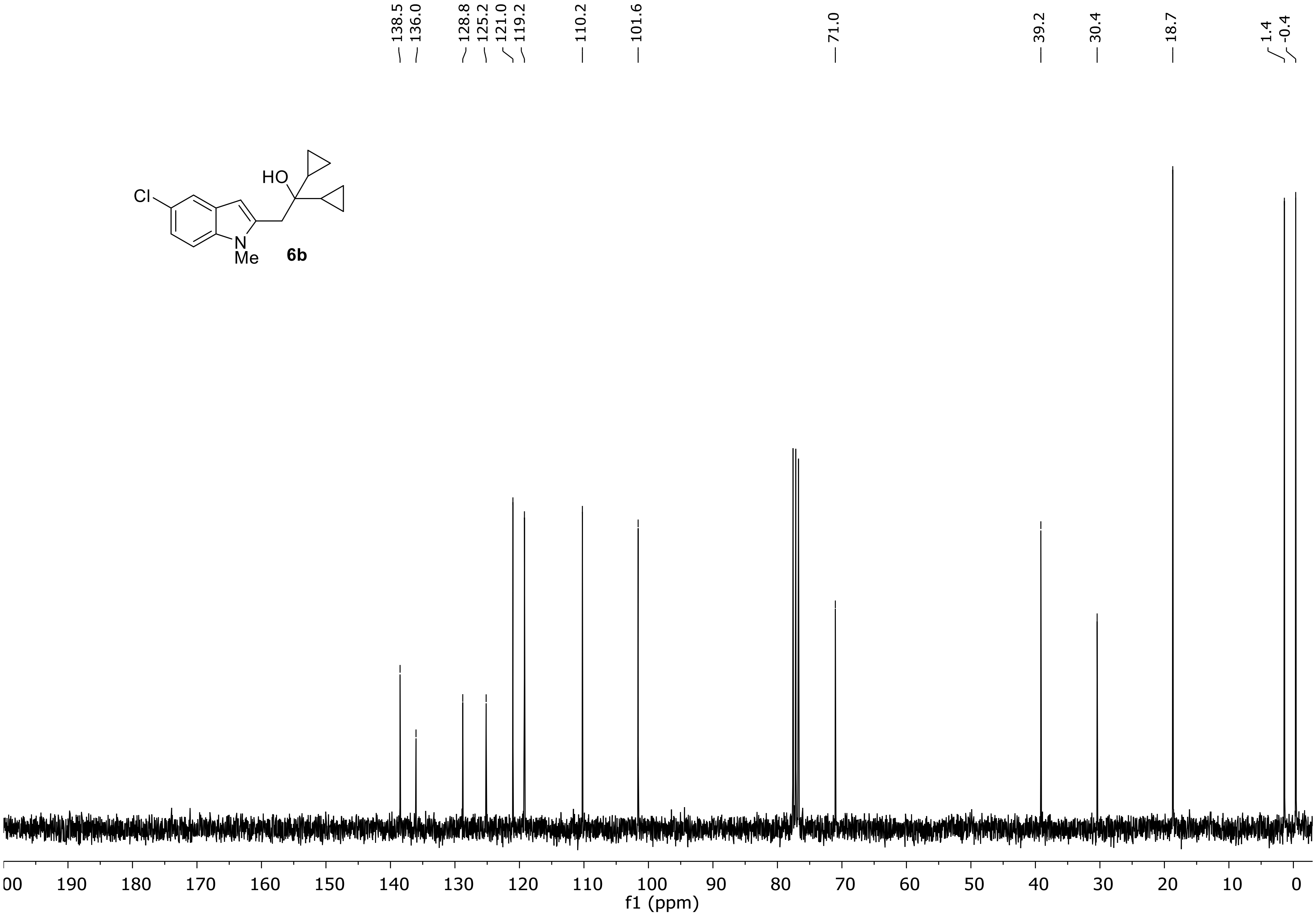

^{1}H-NMR (75.4 MHz, $CDCl_3$)

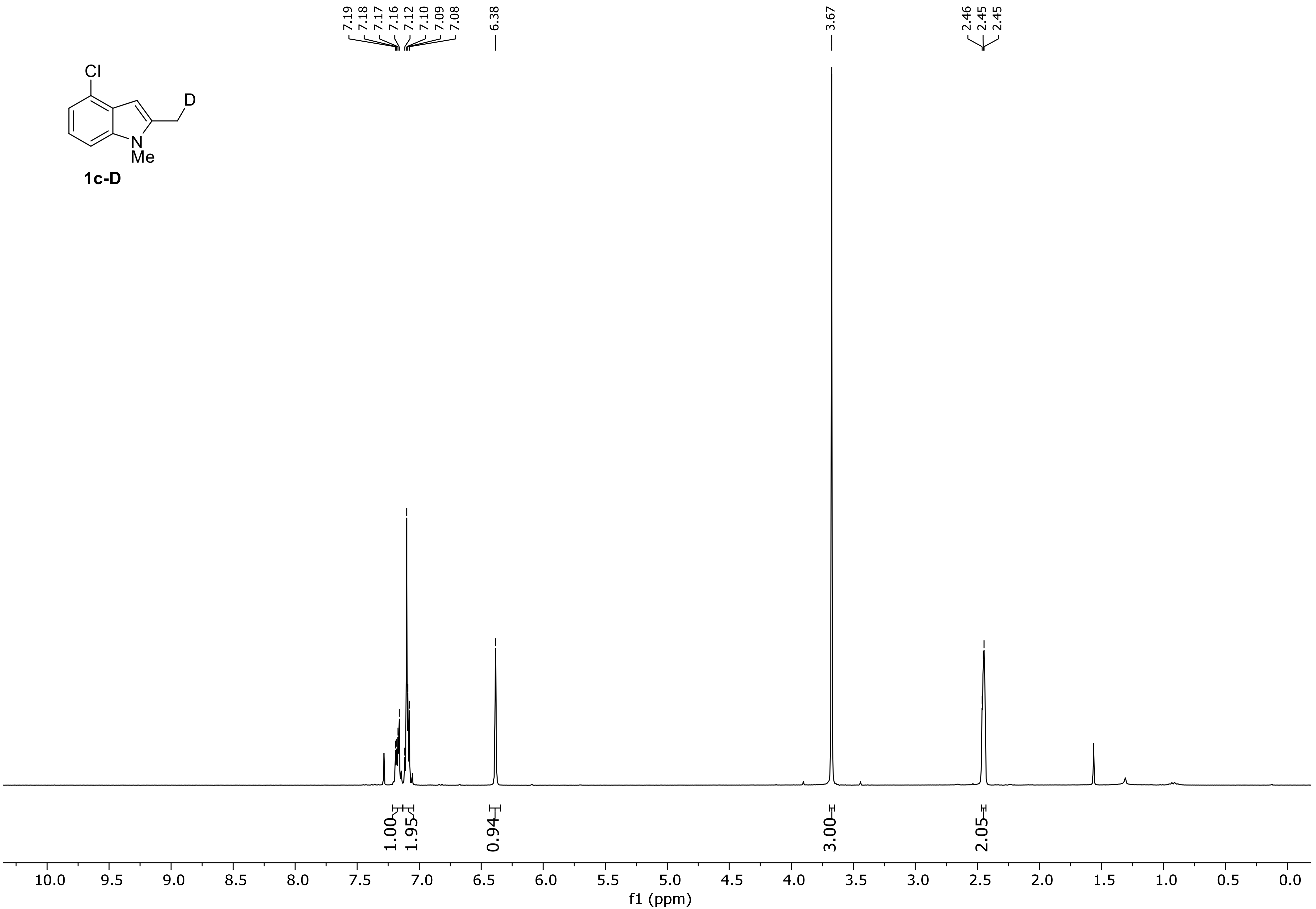

$^{13}C\{^{1}H\}$-NMR (300 MHz, $CDCl_3$)

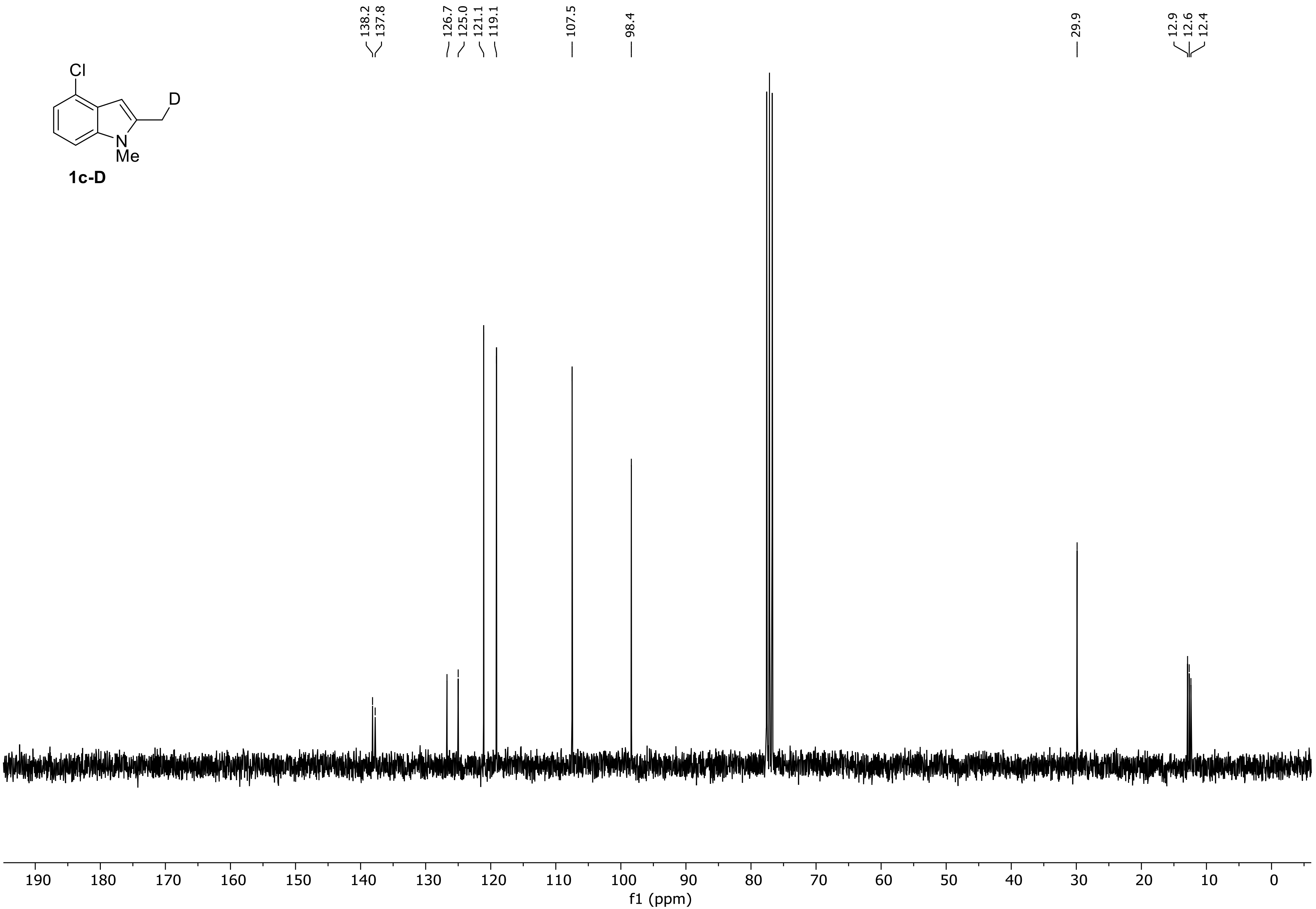

^{1}H-NMR (75.4 MHz, $CDCl_3$)

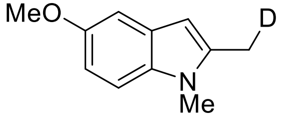
1c-D

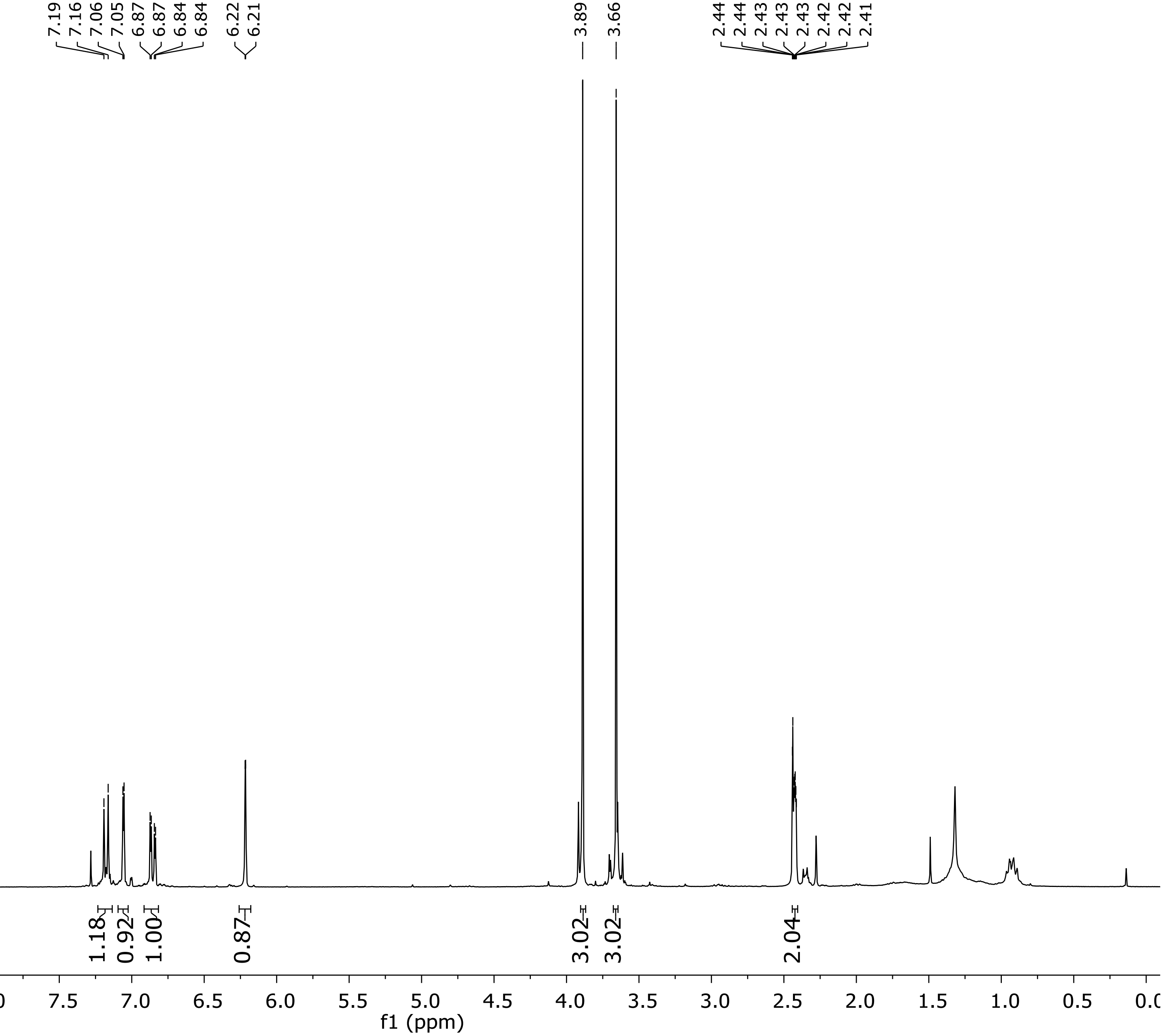

$^{13}C\{^1H\}$-NMR (300 MHz, $CDCl_3$)

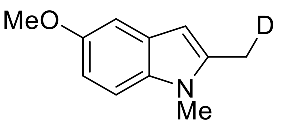

1c-D

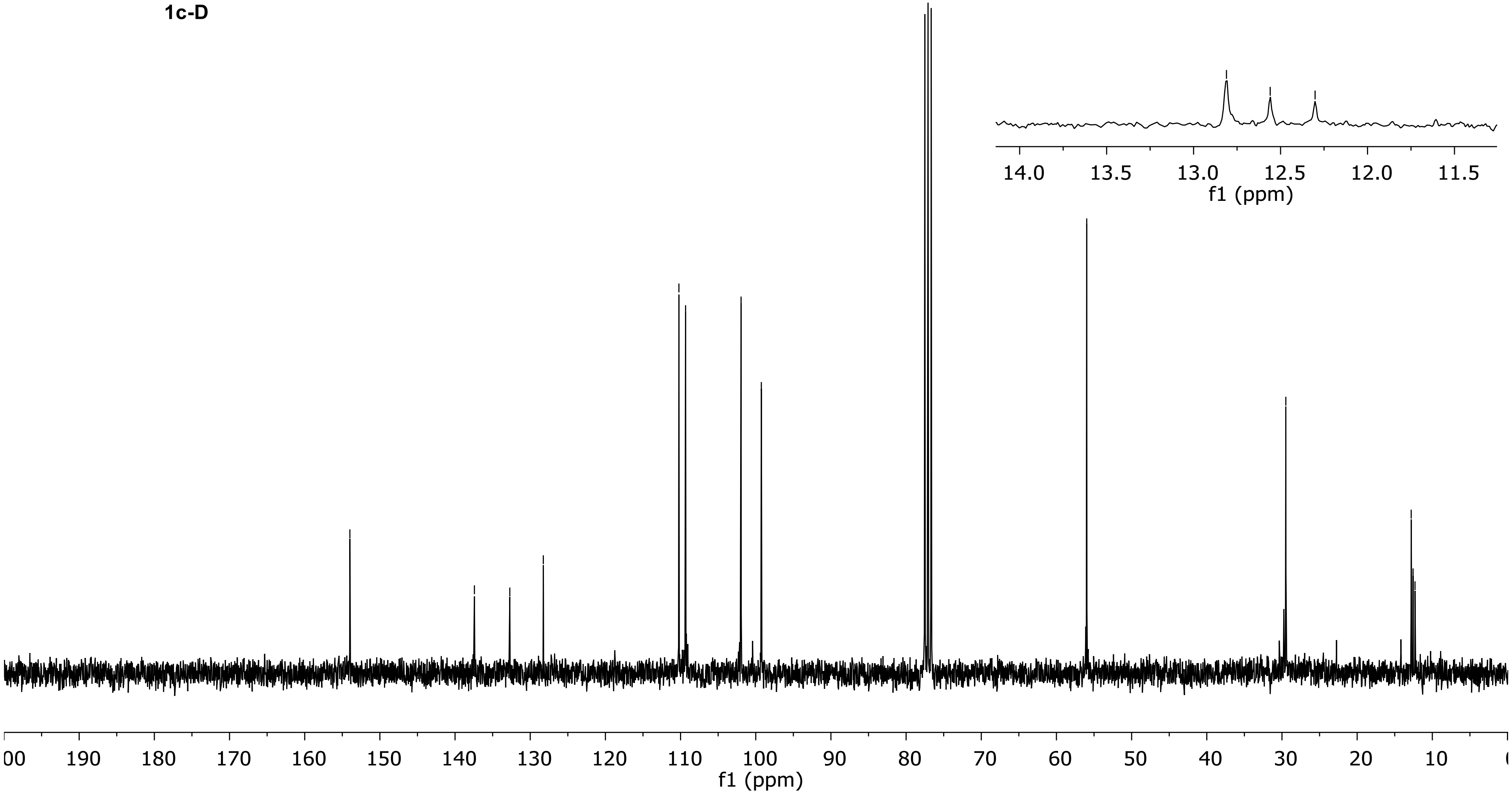

^{1}H-NMR (75.4 MHz, $CDCl_3$)

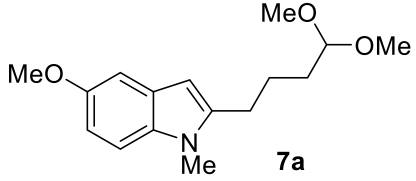

7a

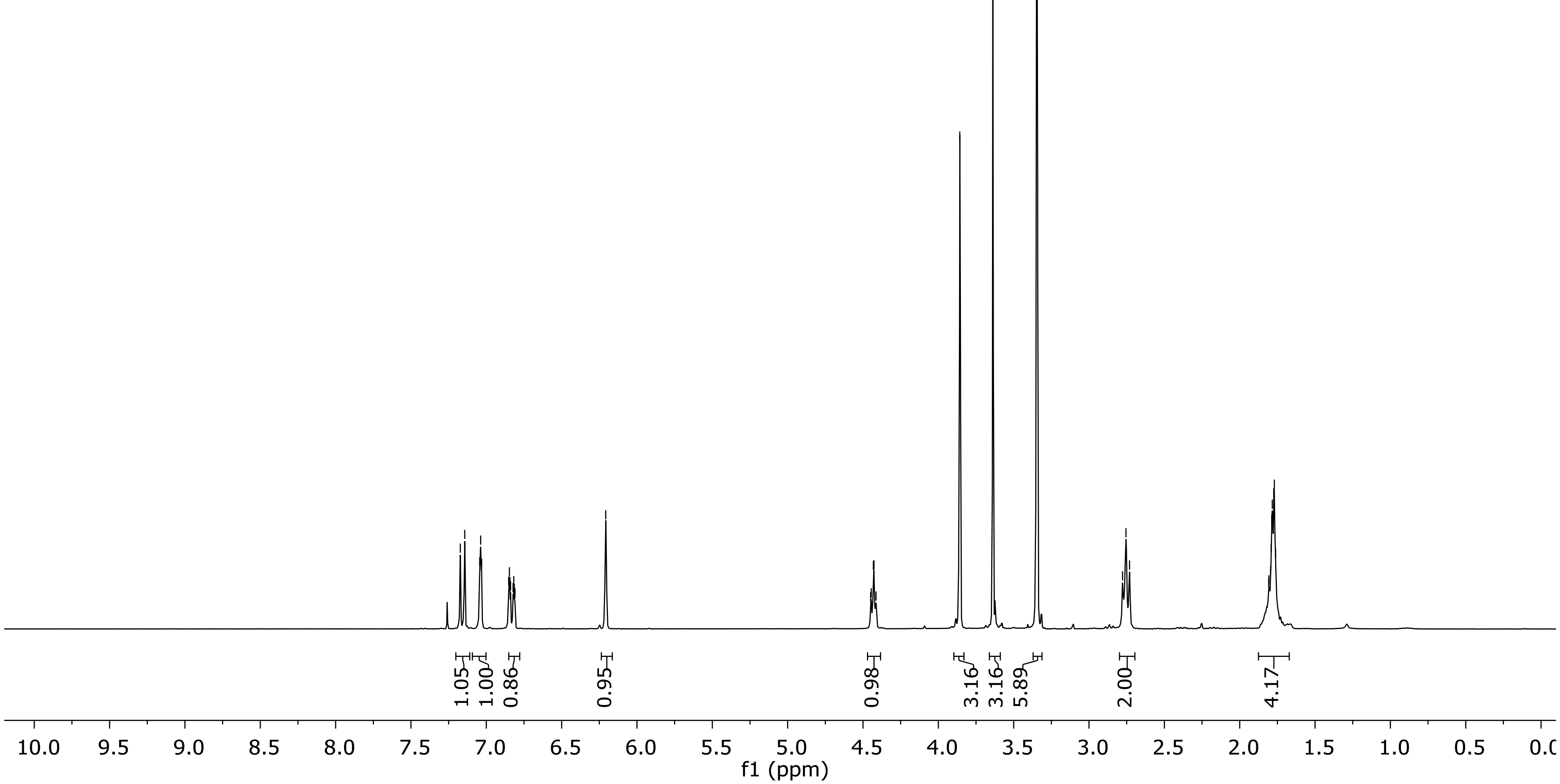

$^{13}C\{^{1}H\}$-NMR (300 MHz, $CDCl_3$)

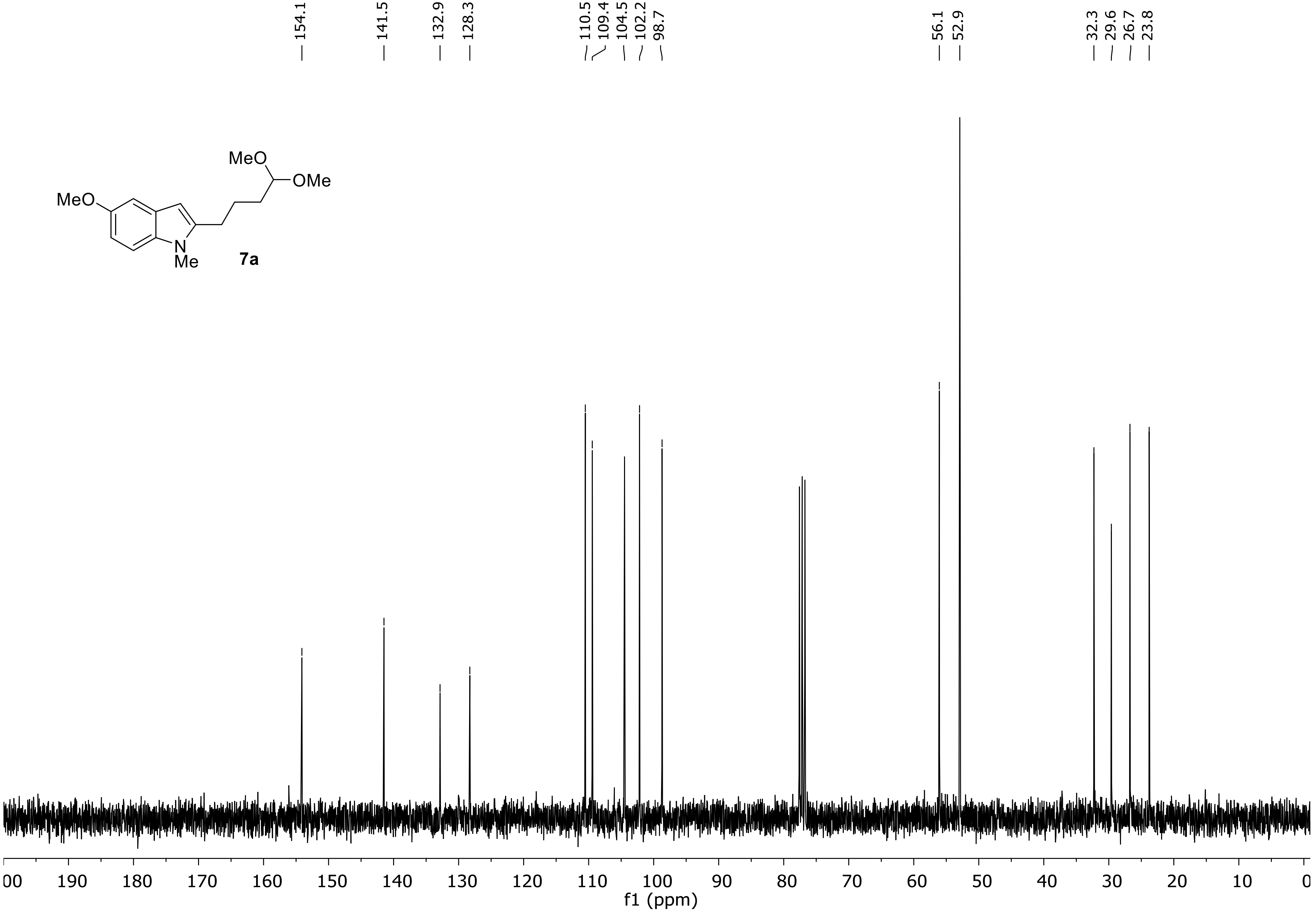

^{1}H-NMR (126 MHz, $CDCl_3$)

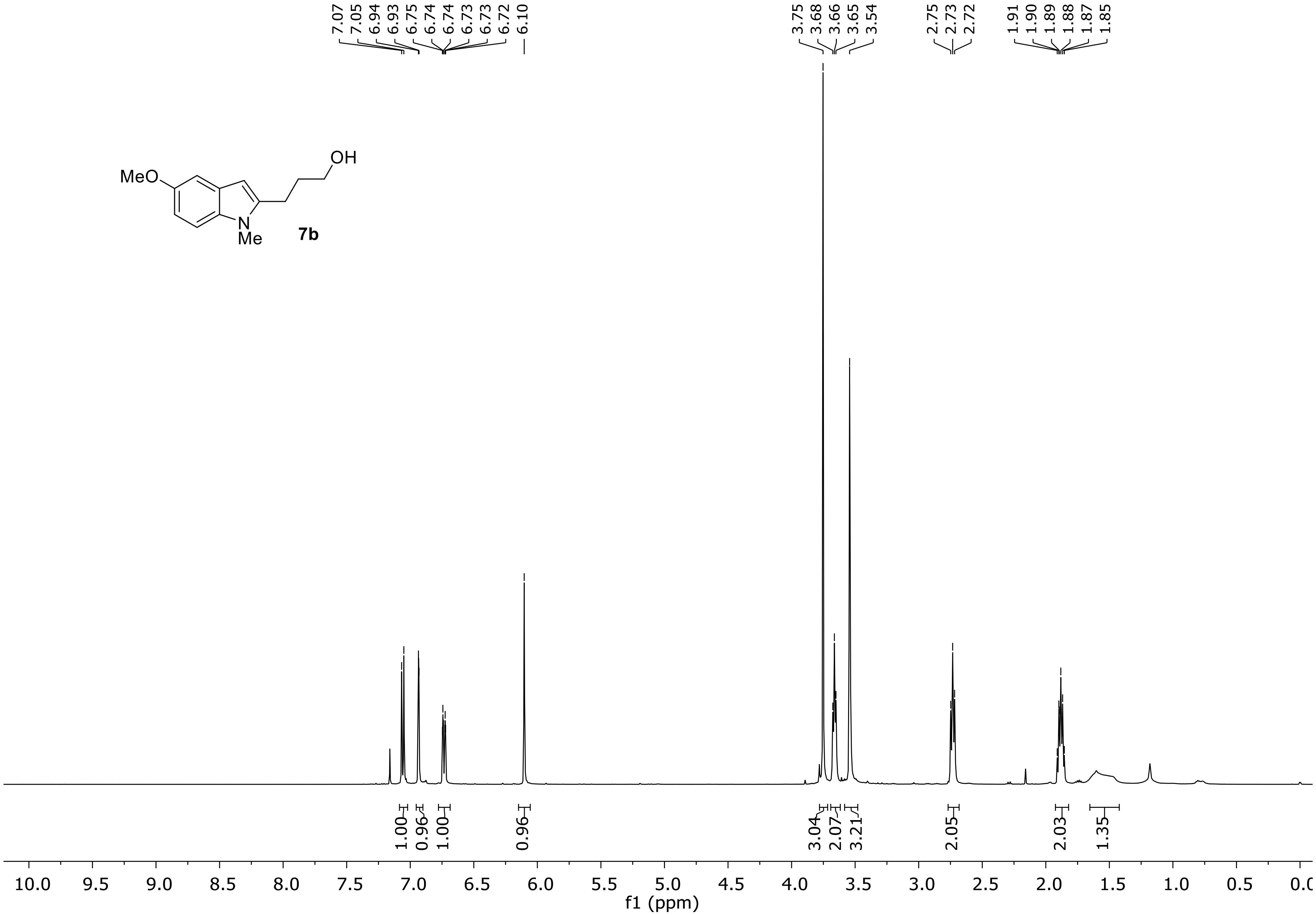

$^{13}C\{^{1}H\}$-NMR (500 MHz, $CDCl_3$)

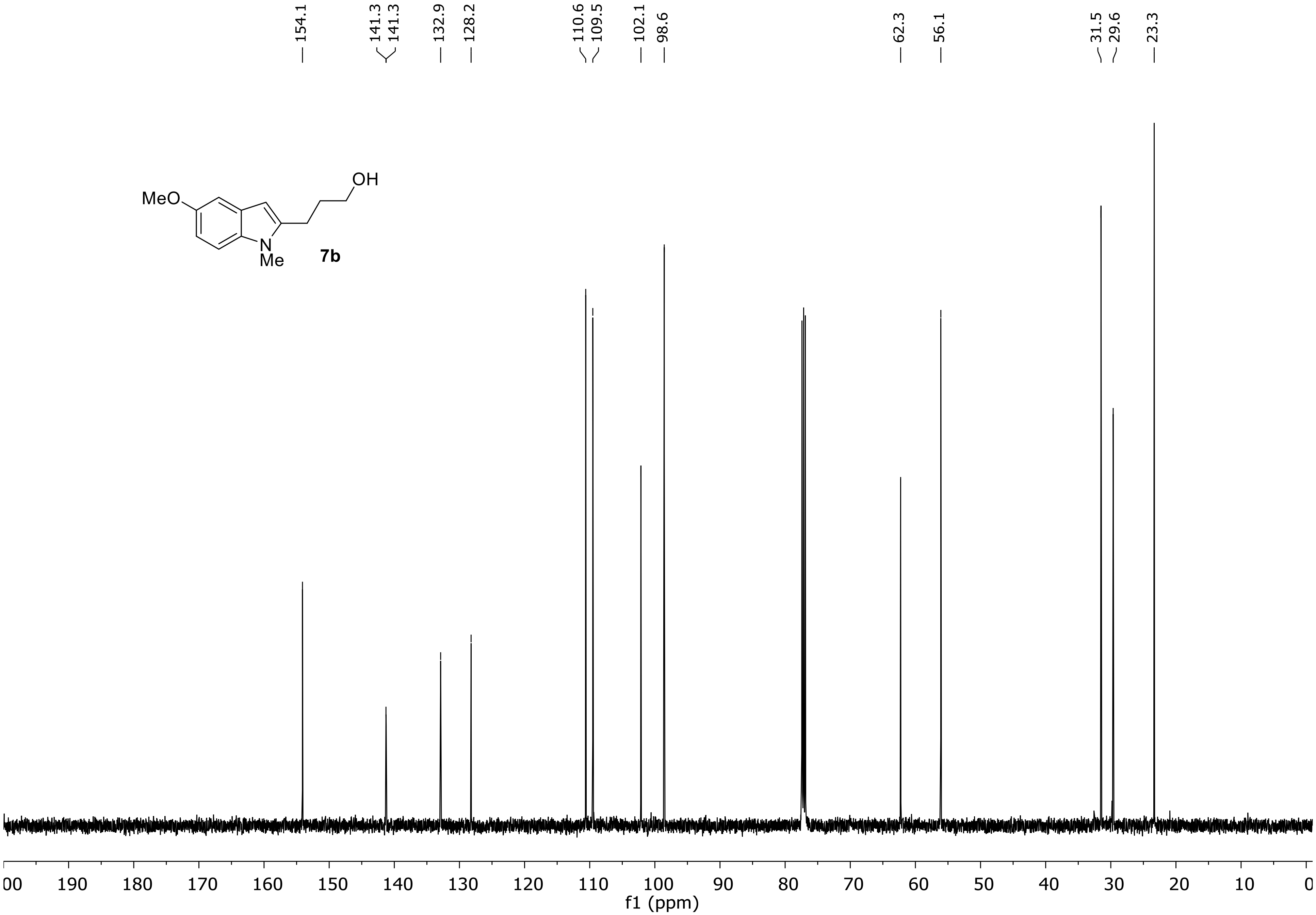

^{1}H-NMR (126 MHz, $CDCl_3$)

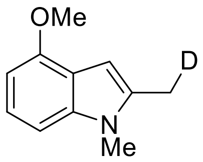

1f-D

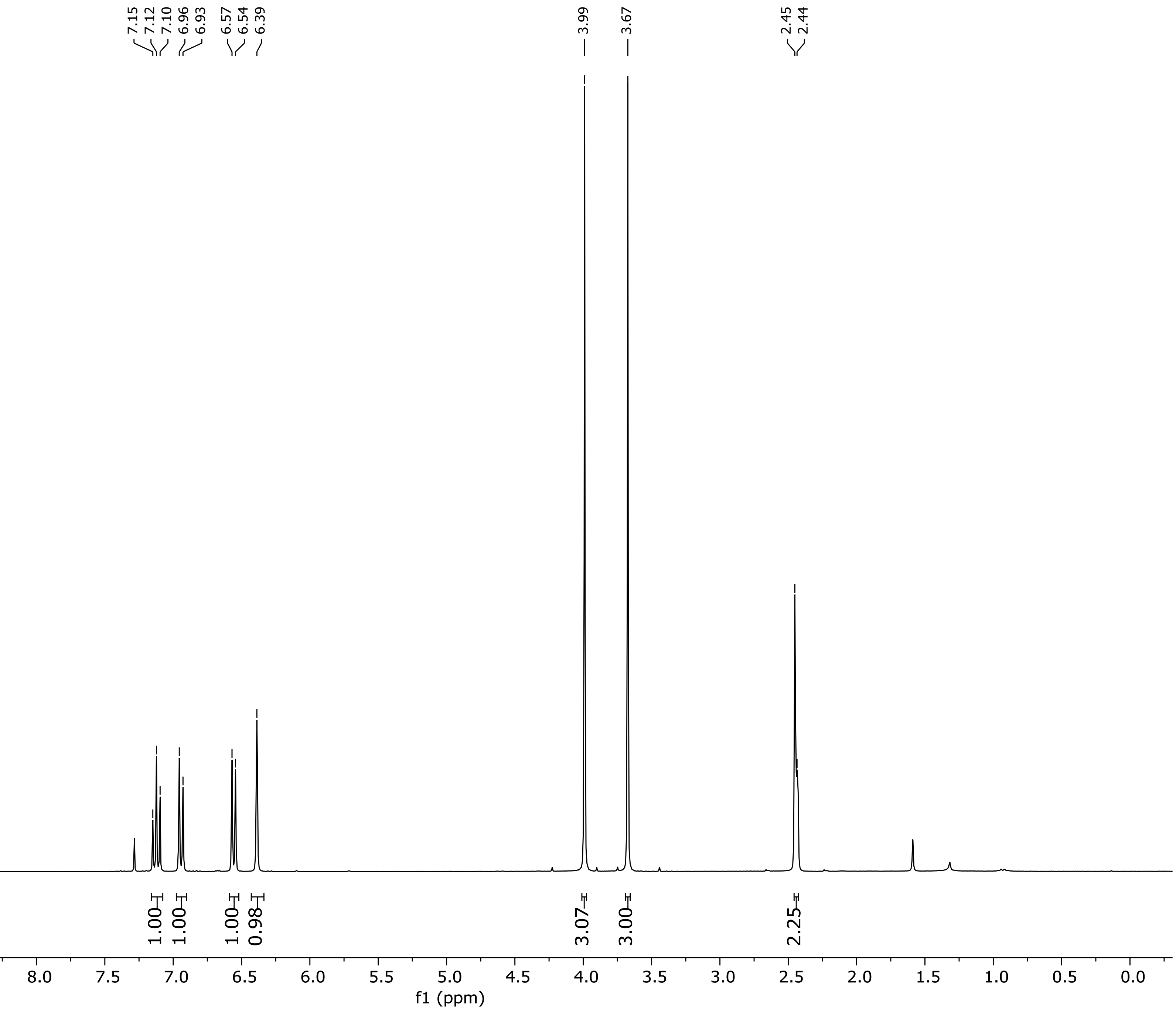

$^{13}C\{^1H\}$-NMR (500 MHz, $CDCl_3$)

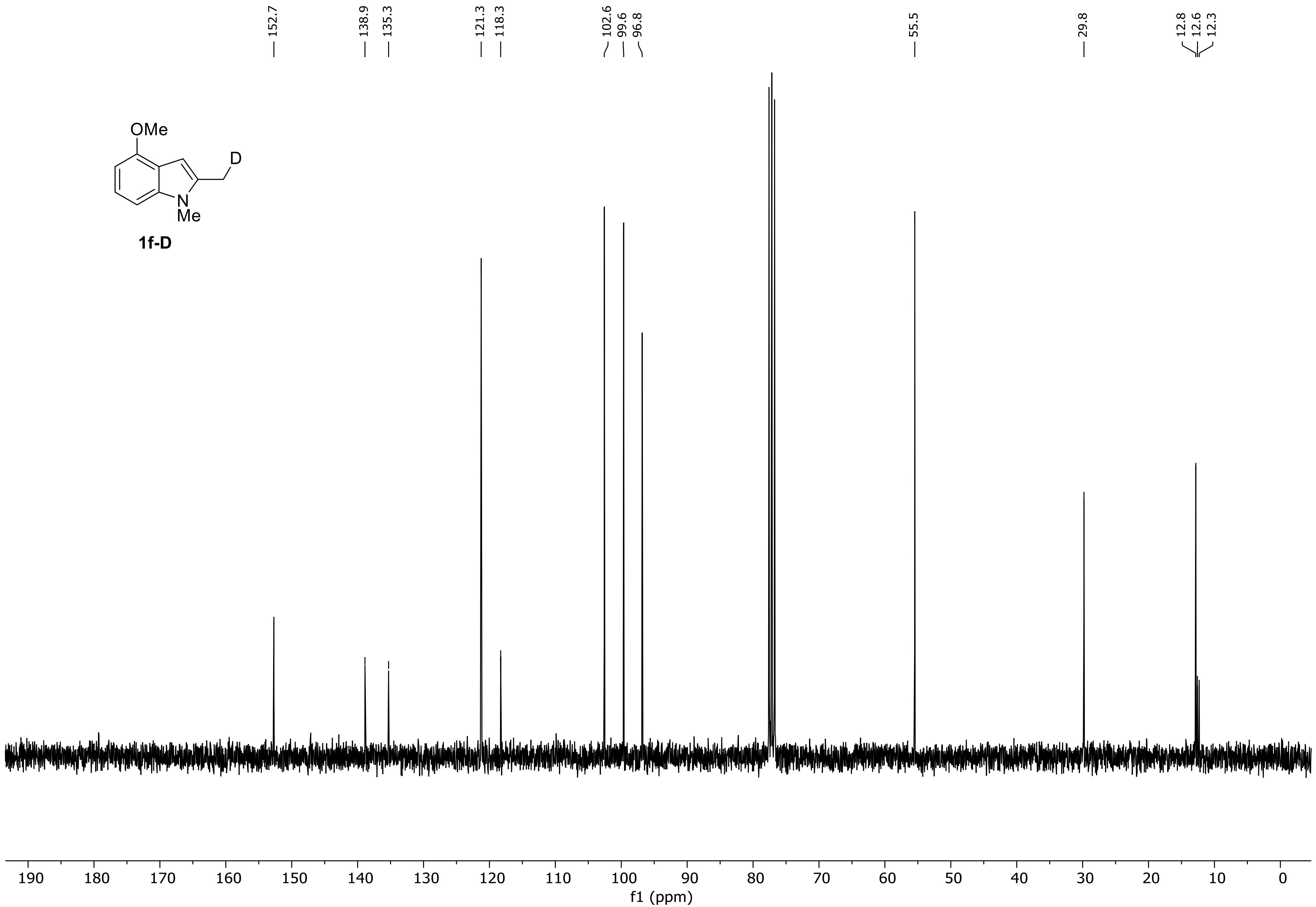

^{1}H-NMR (126 MHz, $CDCl_3$)

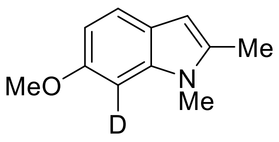

1g-D

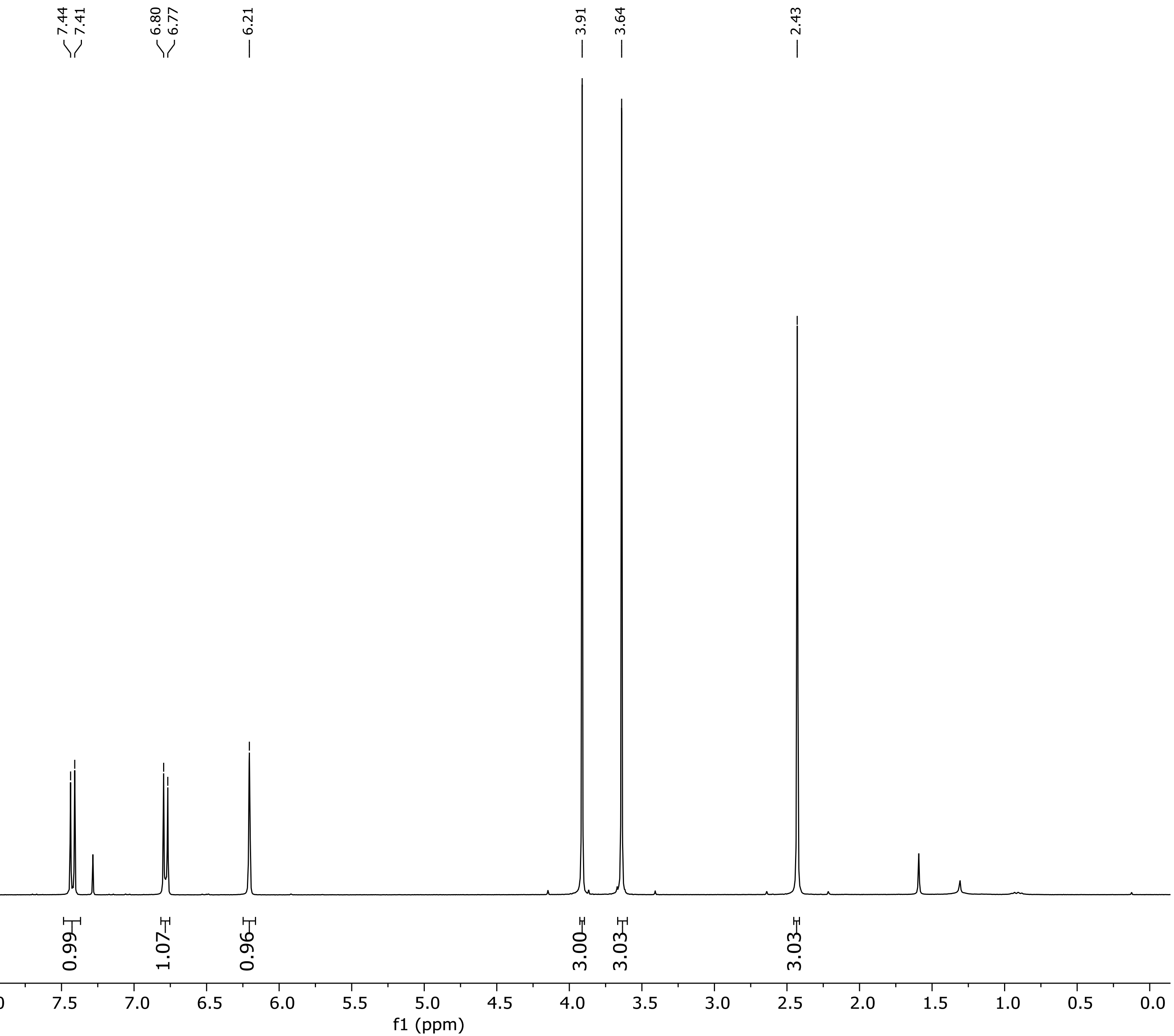

$^{13}C\{^1H\}$-NMR (500 MHz, $CDCl_3$)

1g-D

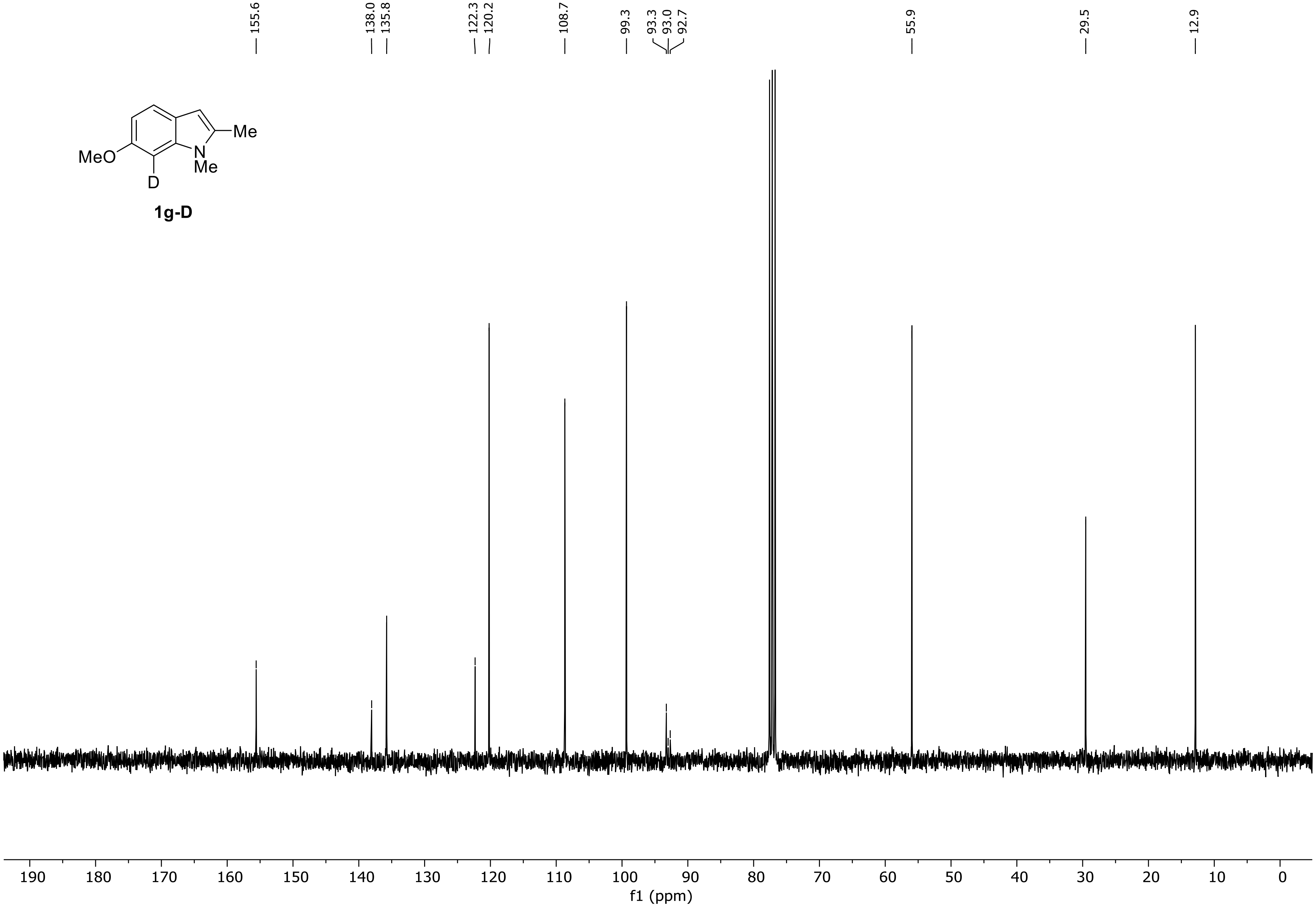

^{1}H-NMR (126 MHz, $CDCl_3$)

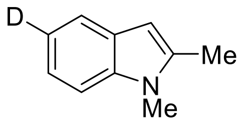

1h-D

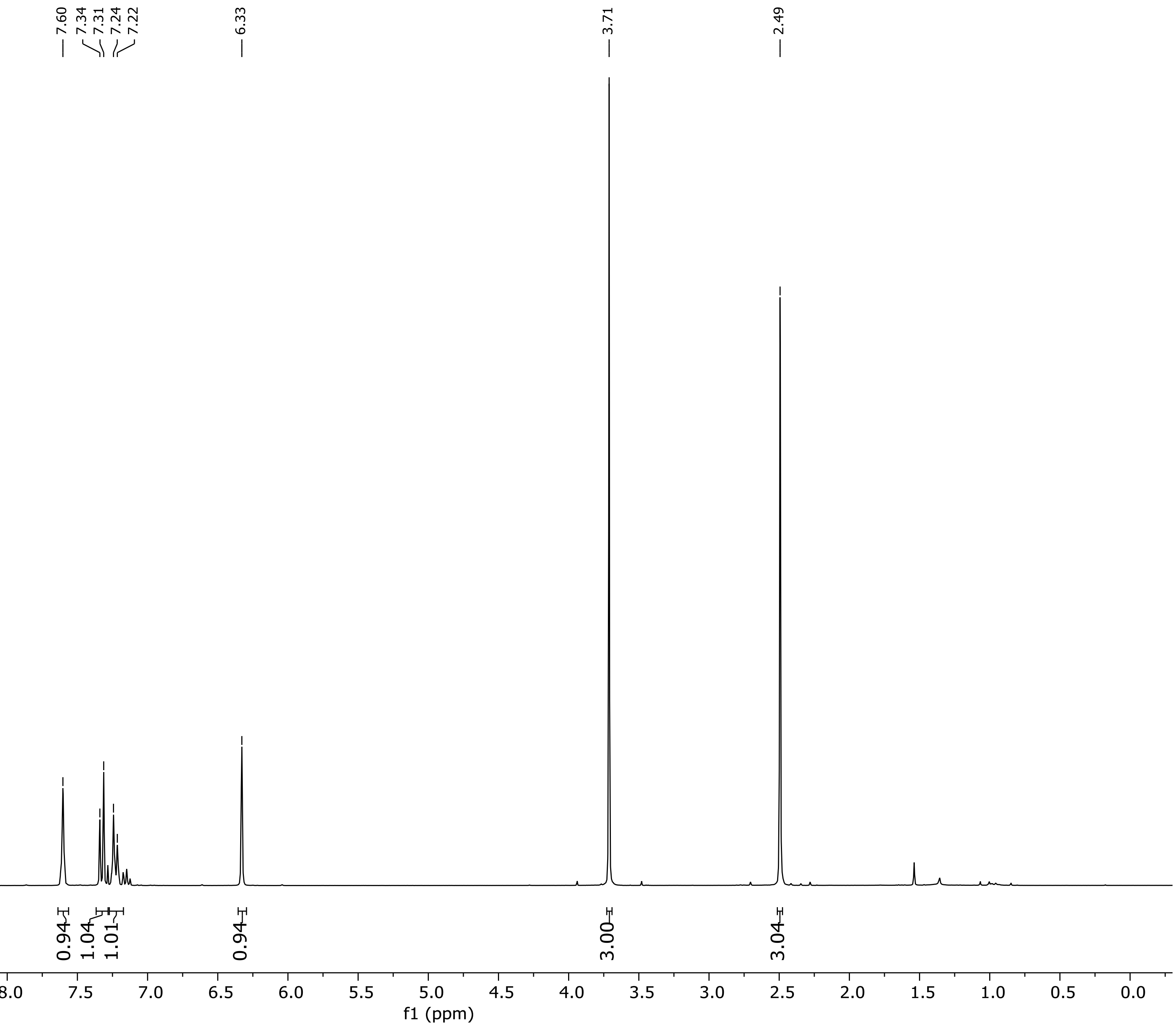

$^{13}C\{^{1}H\}$-NMR (500 MHz, $CDCl_3$)

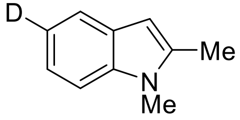

1h-D

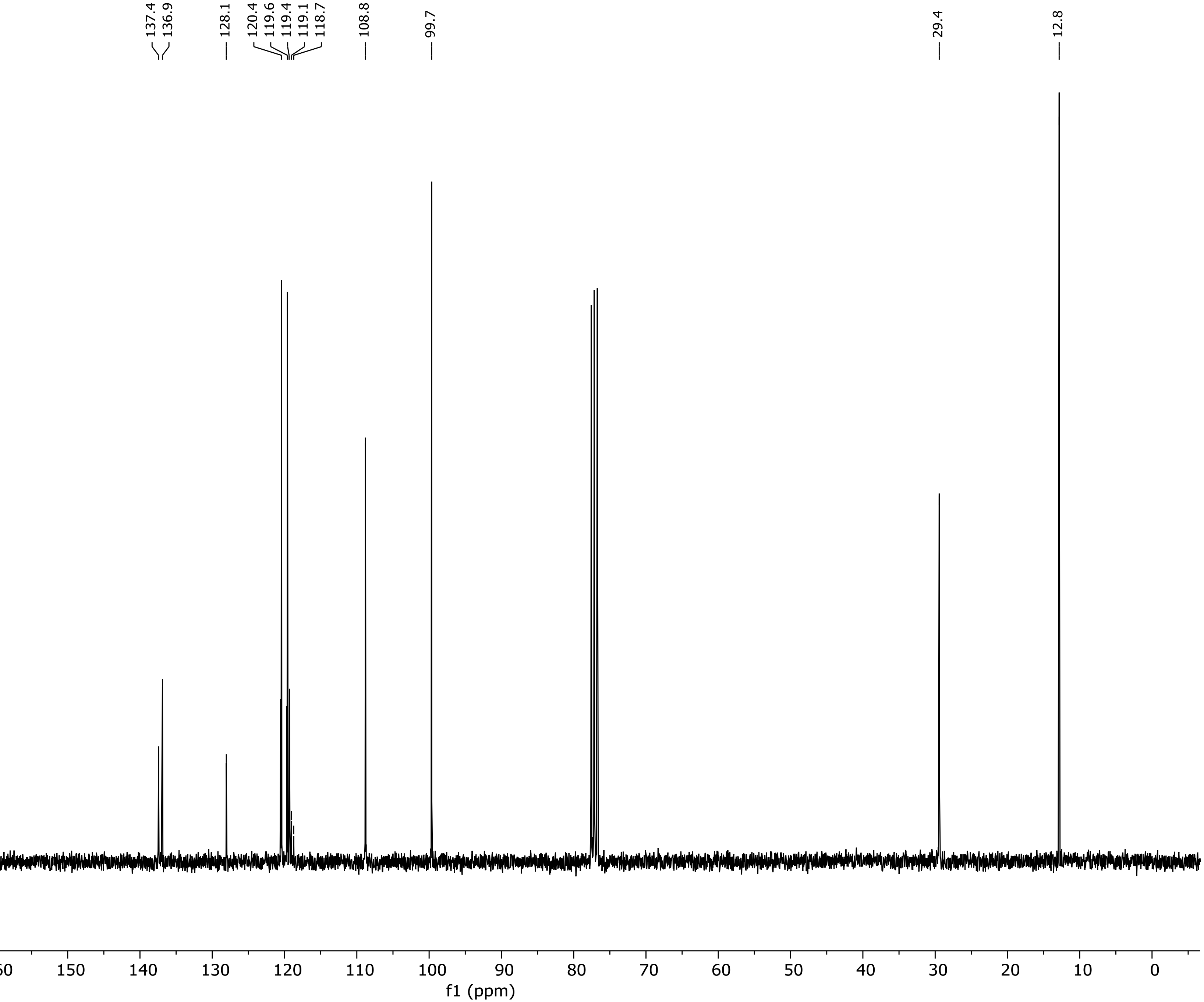

^{1}H-NMR (126 MHz, $CDCl_3$)

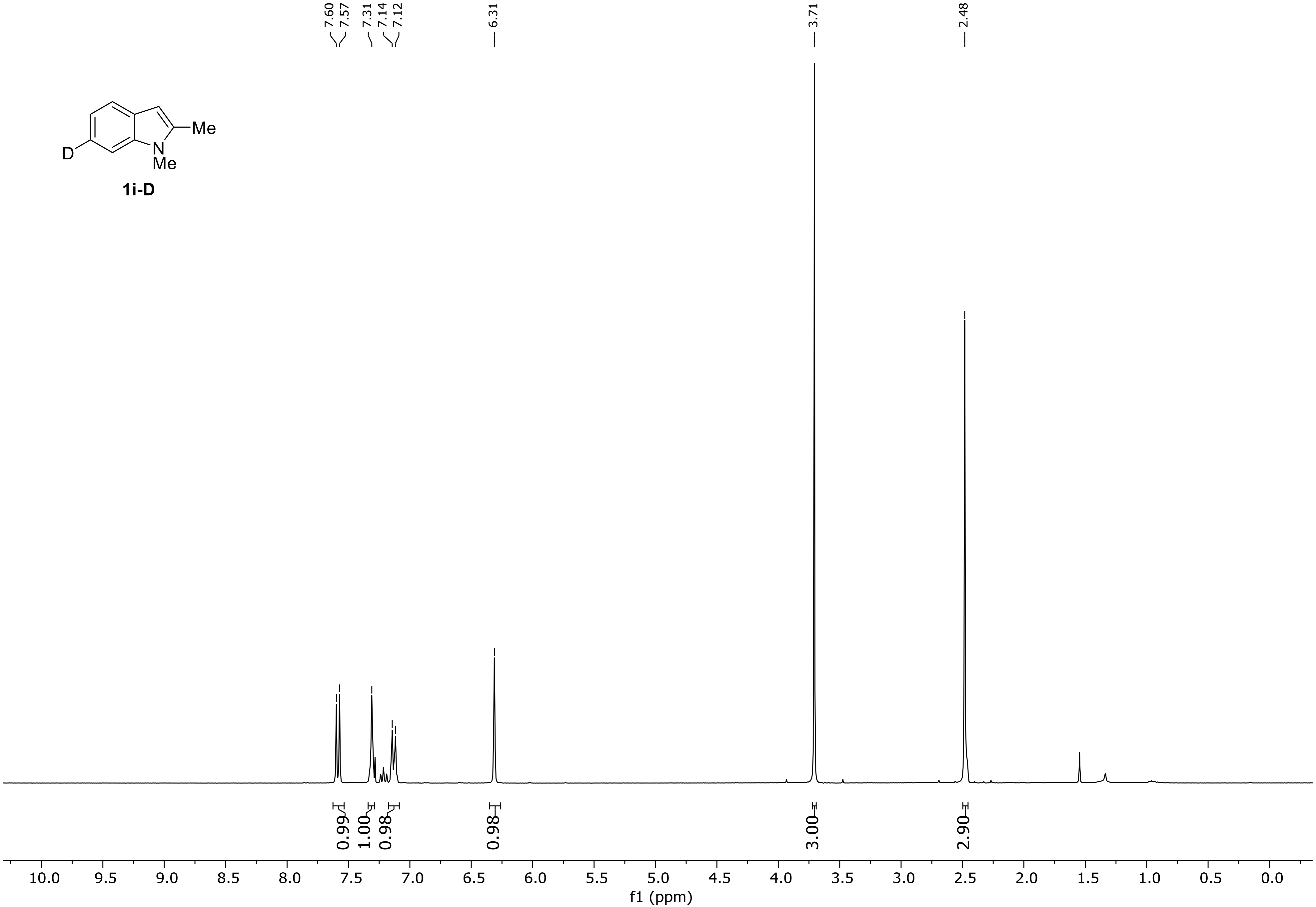

$^{13}C\{^1H\}$-NMR (500 MHz, $CDCl_3$)

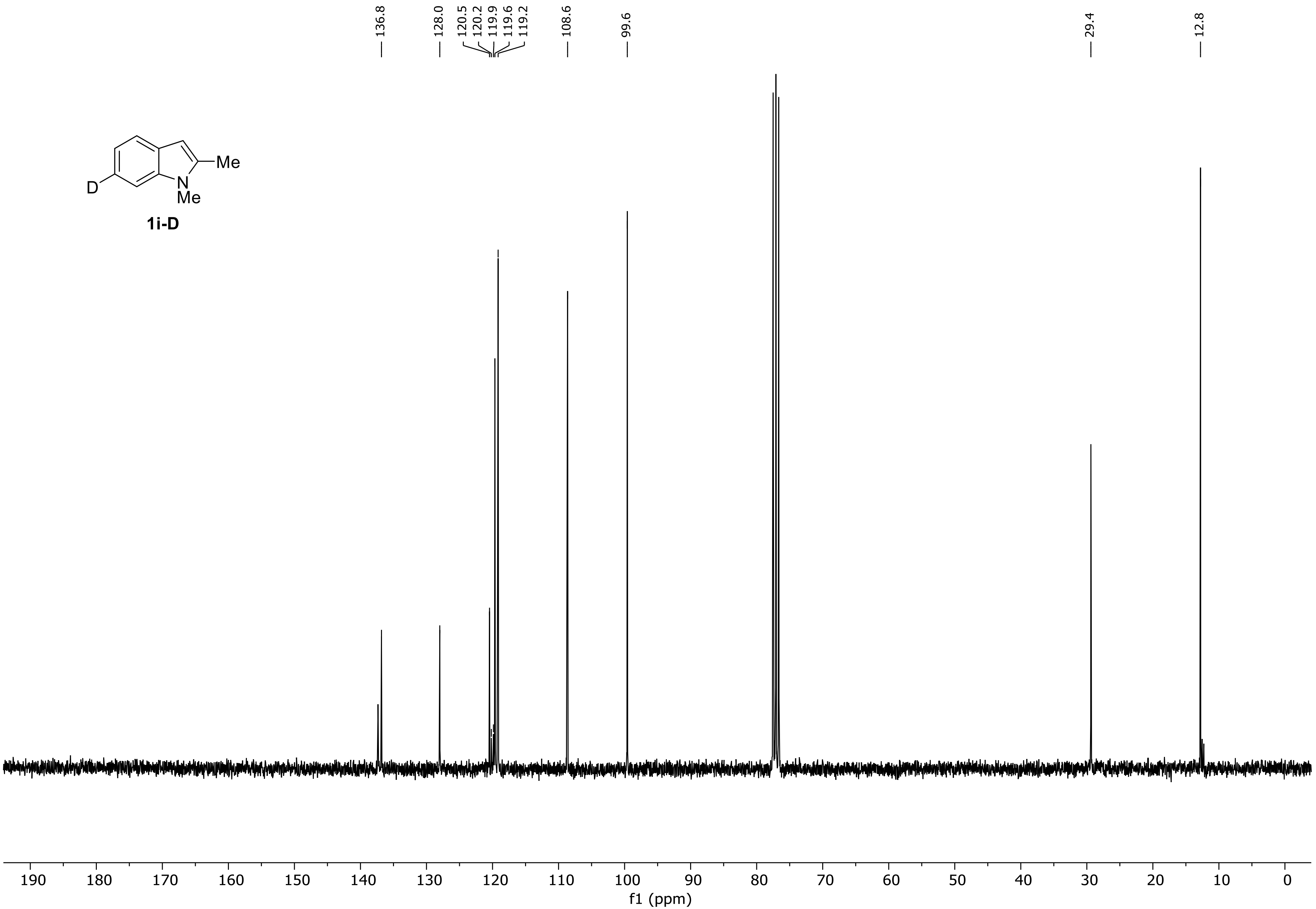

^{1}H-NMR (126 MHz, $CDCl_3$)

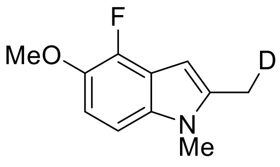

1k-D

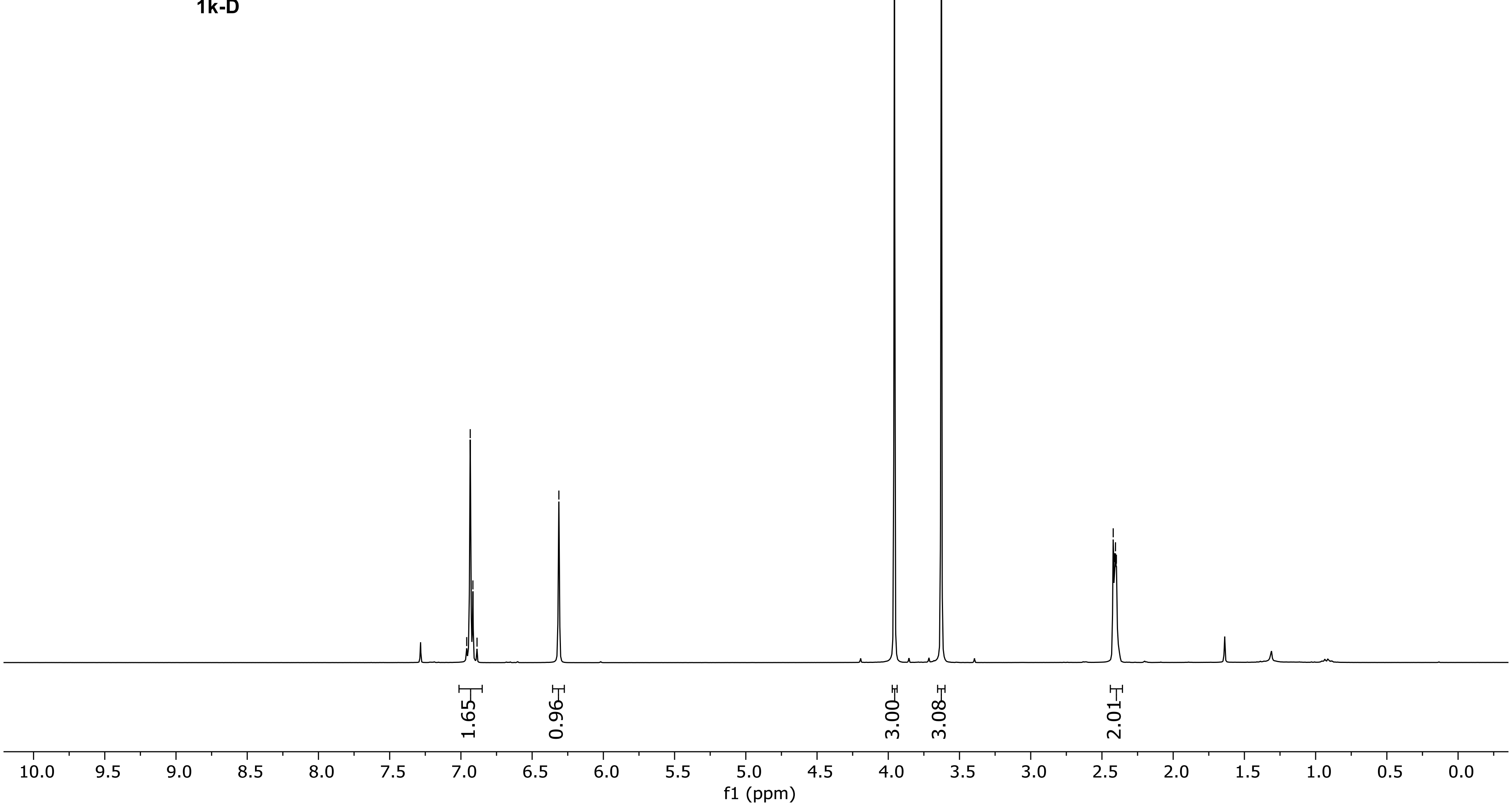

$^{13}C\{^1H\}$-NMR (500 MHz, $CDCl_3$)

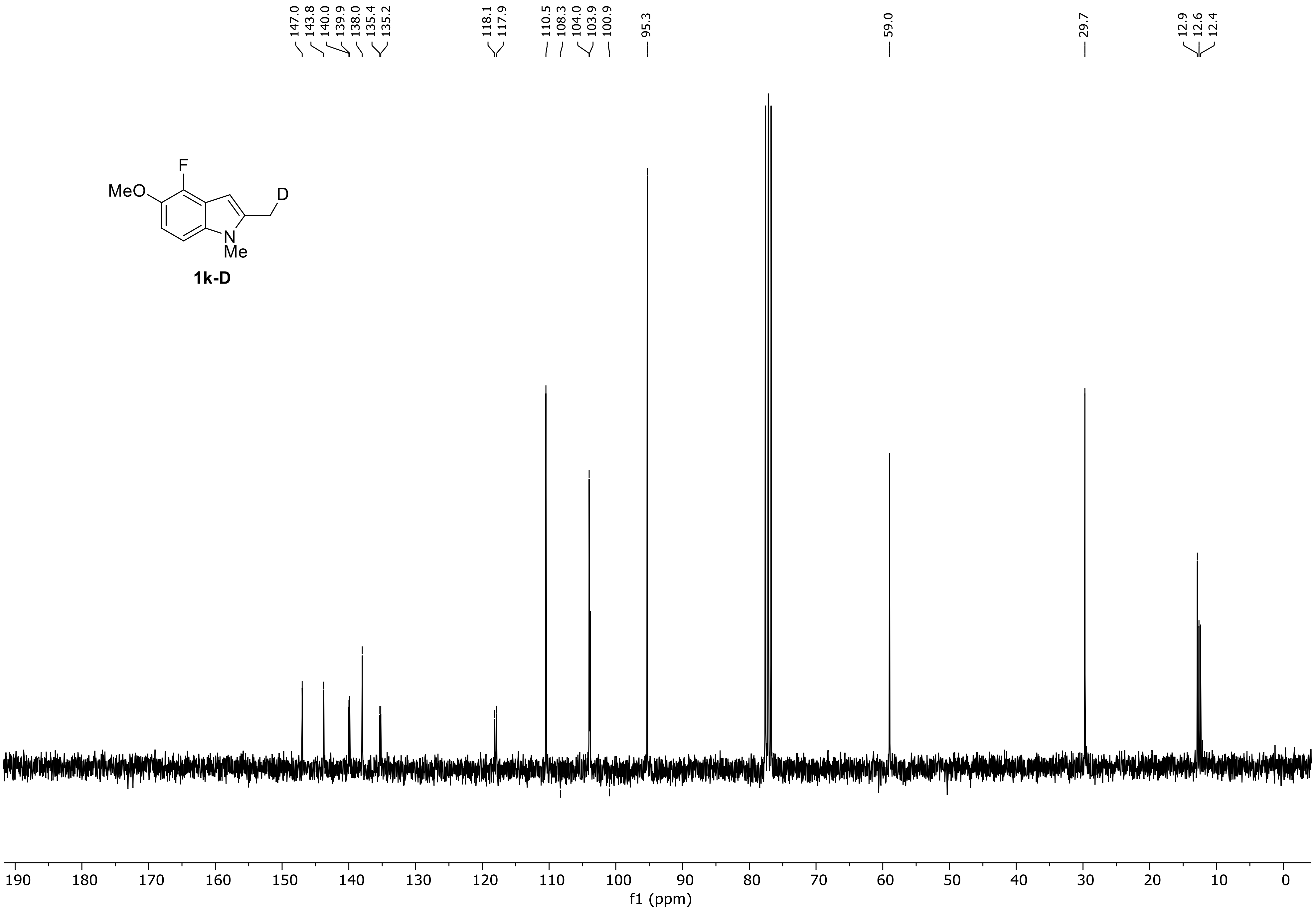

^{1}H-NMR (126 MHz, $CDCl_3$)

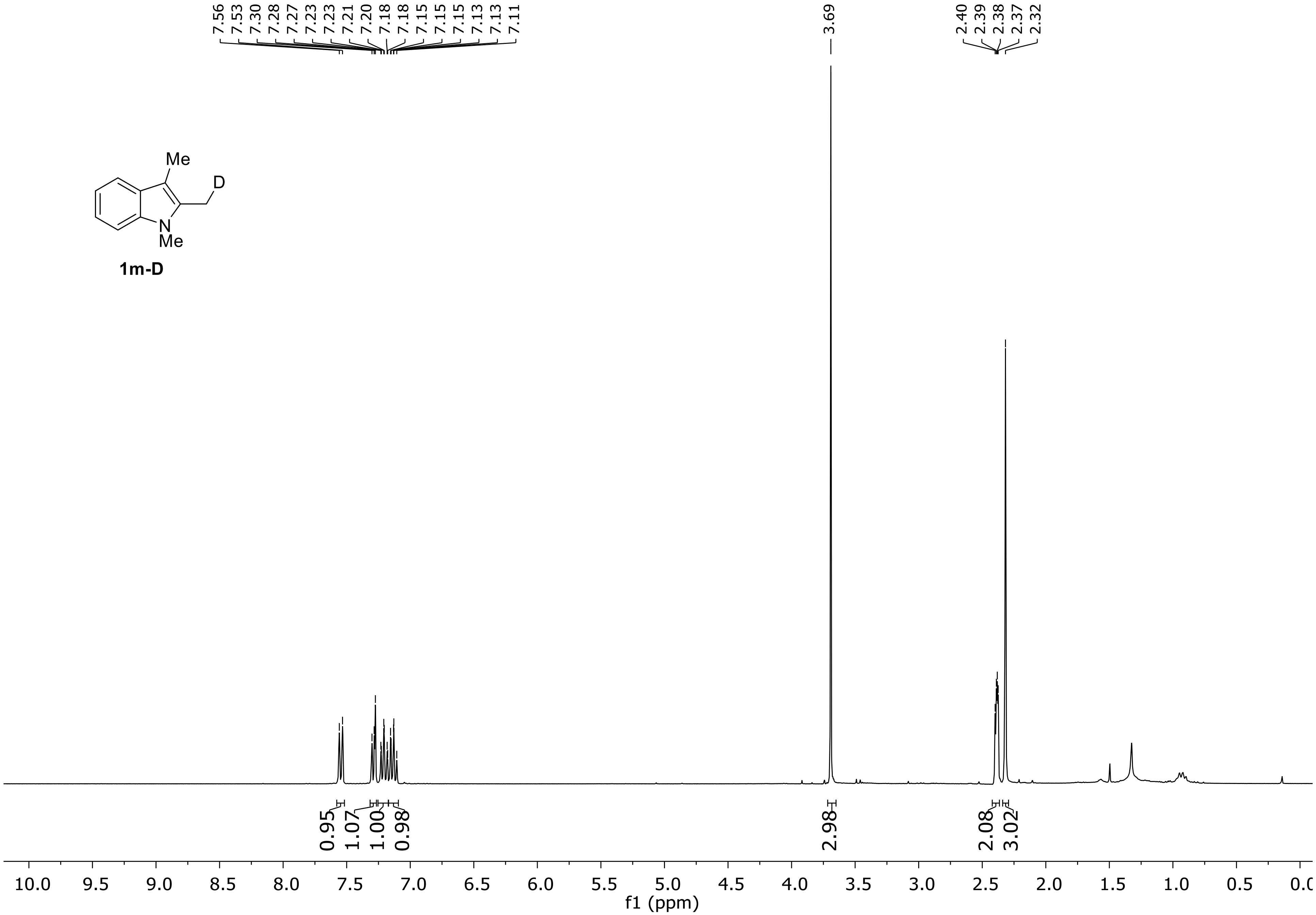

$^{13}C\{^{1}H\}$-NMR (500 MHz, $CDCl_3$)

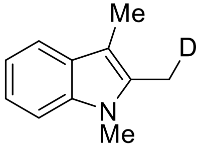

1m-D

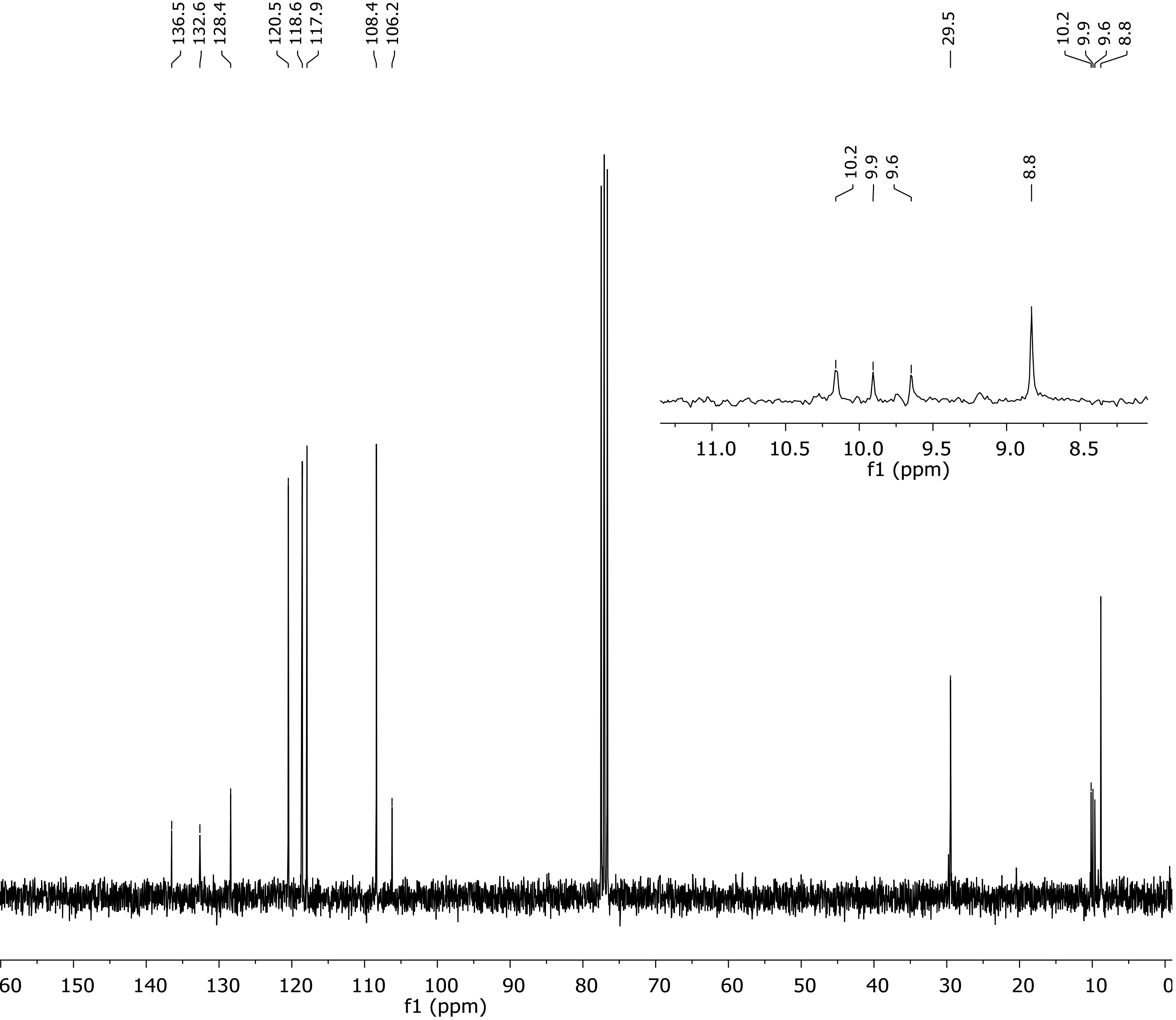

^{1}H-NMR (75.4 MHz, $CDCl_3$)

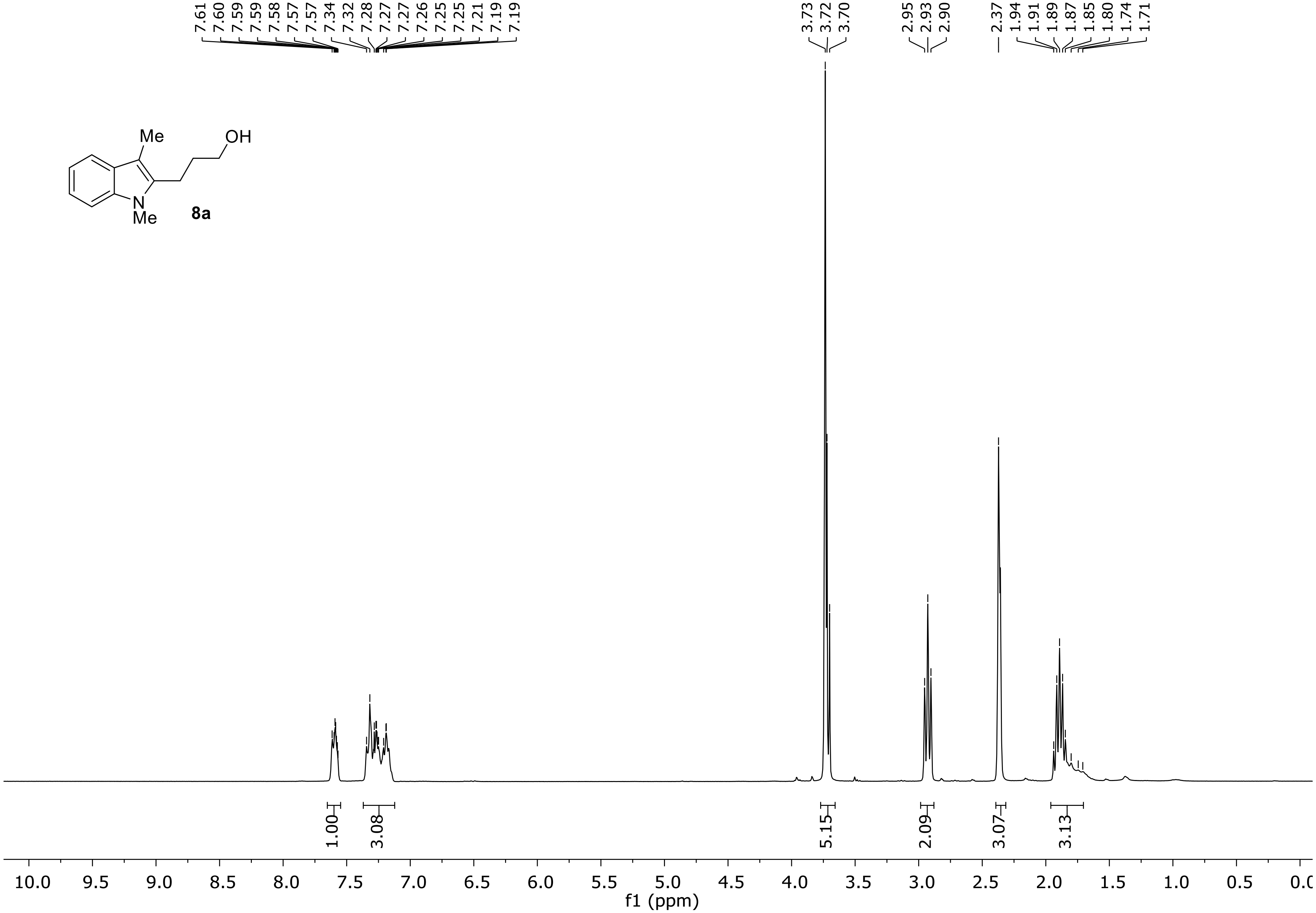

^{3}C{^{1}H}-NMR (300 MHz, $CDCl_3$)

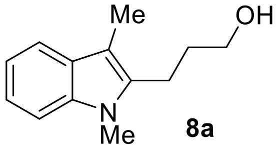

8a

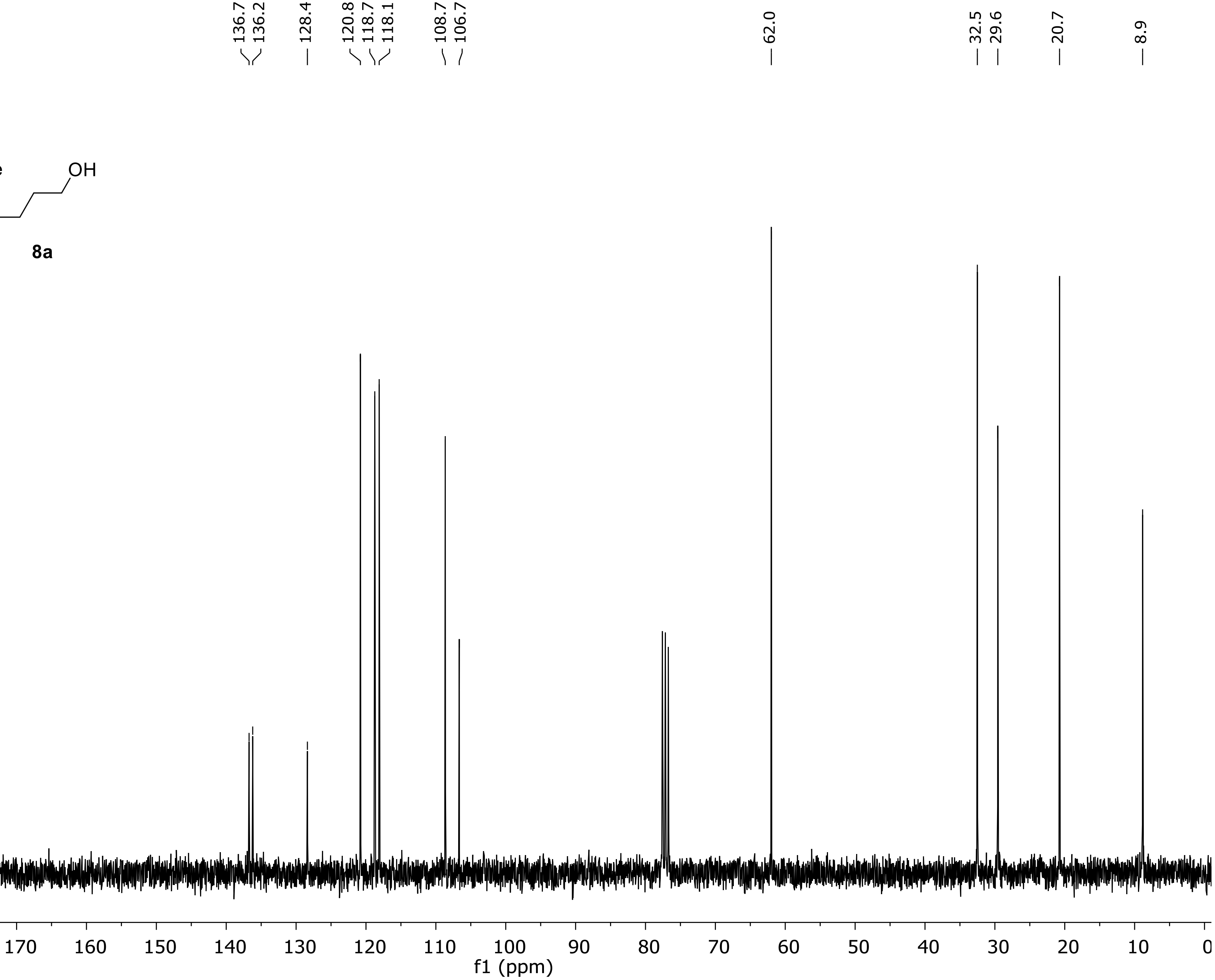

^{1}H-NMR (75.4 MHz, $CDCl_3$)

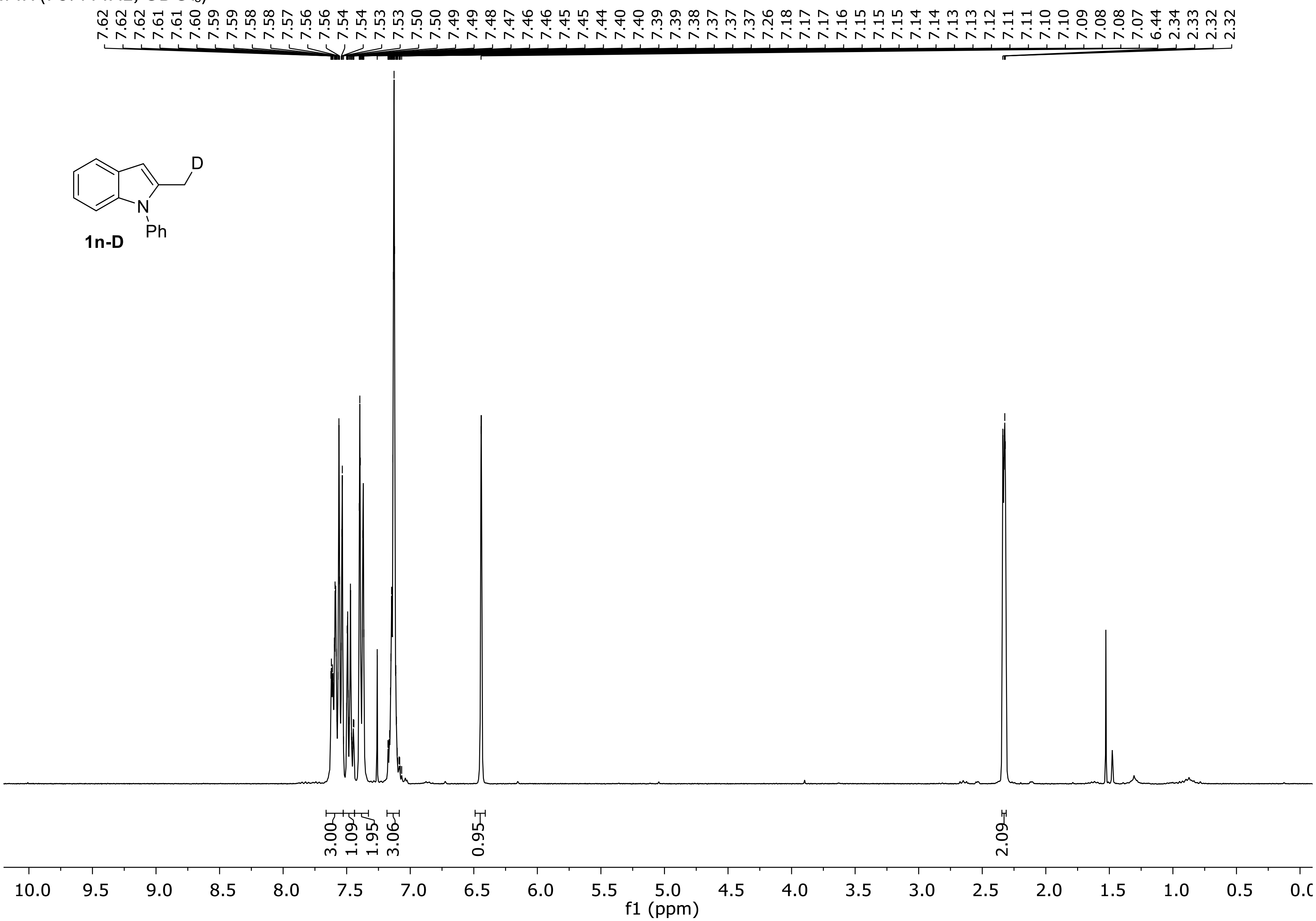

$^{3}C\{^{1}H\}$-NMR (300 MHz, $CDCl_3$)

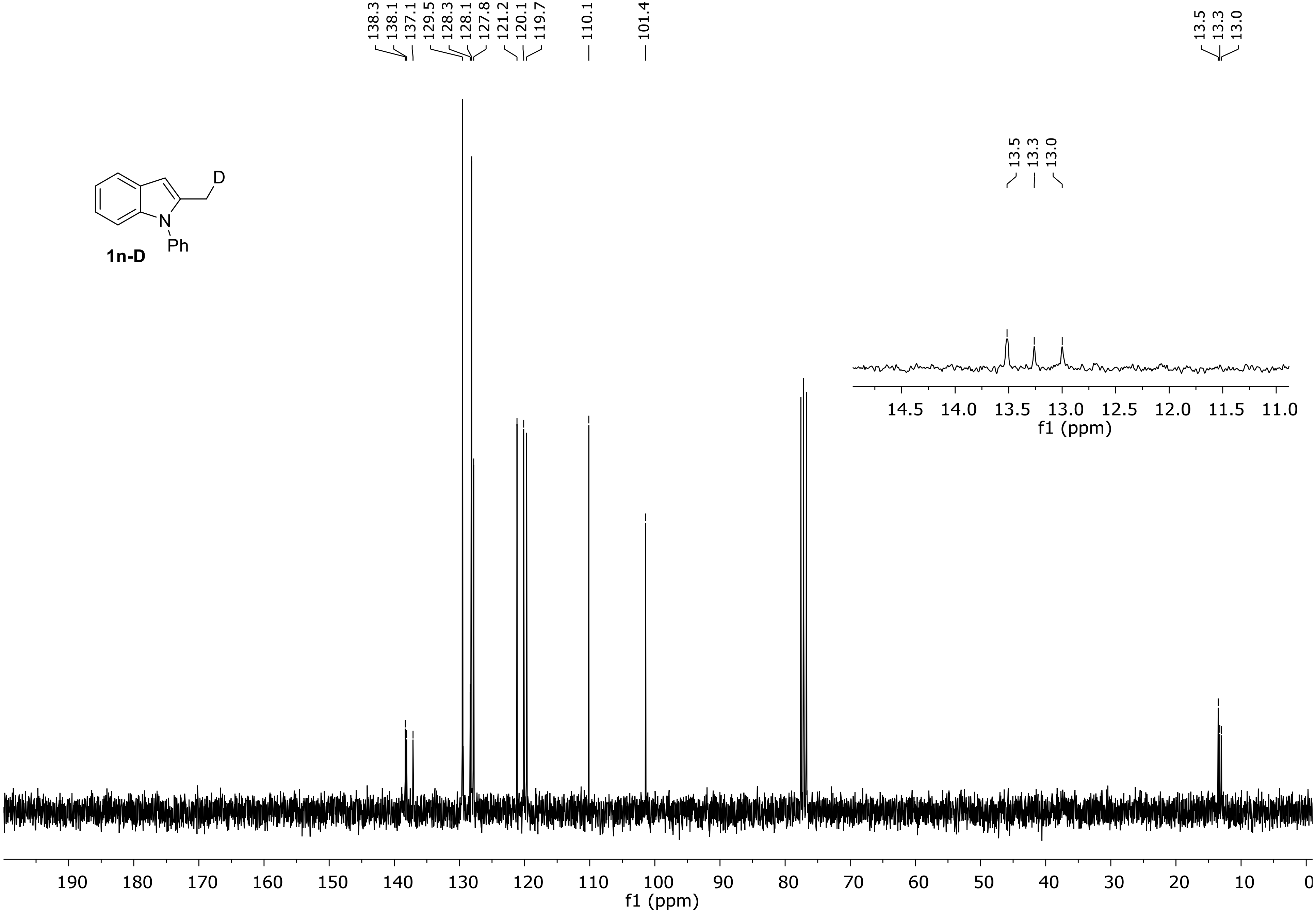

^1H-NMR (75.4 MHz, $CDCl_3$)

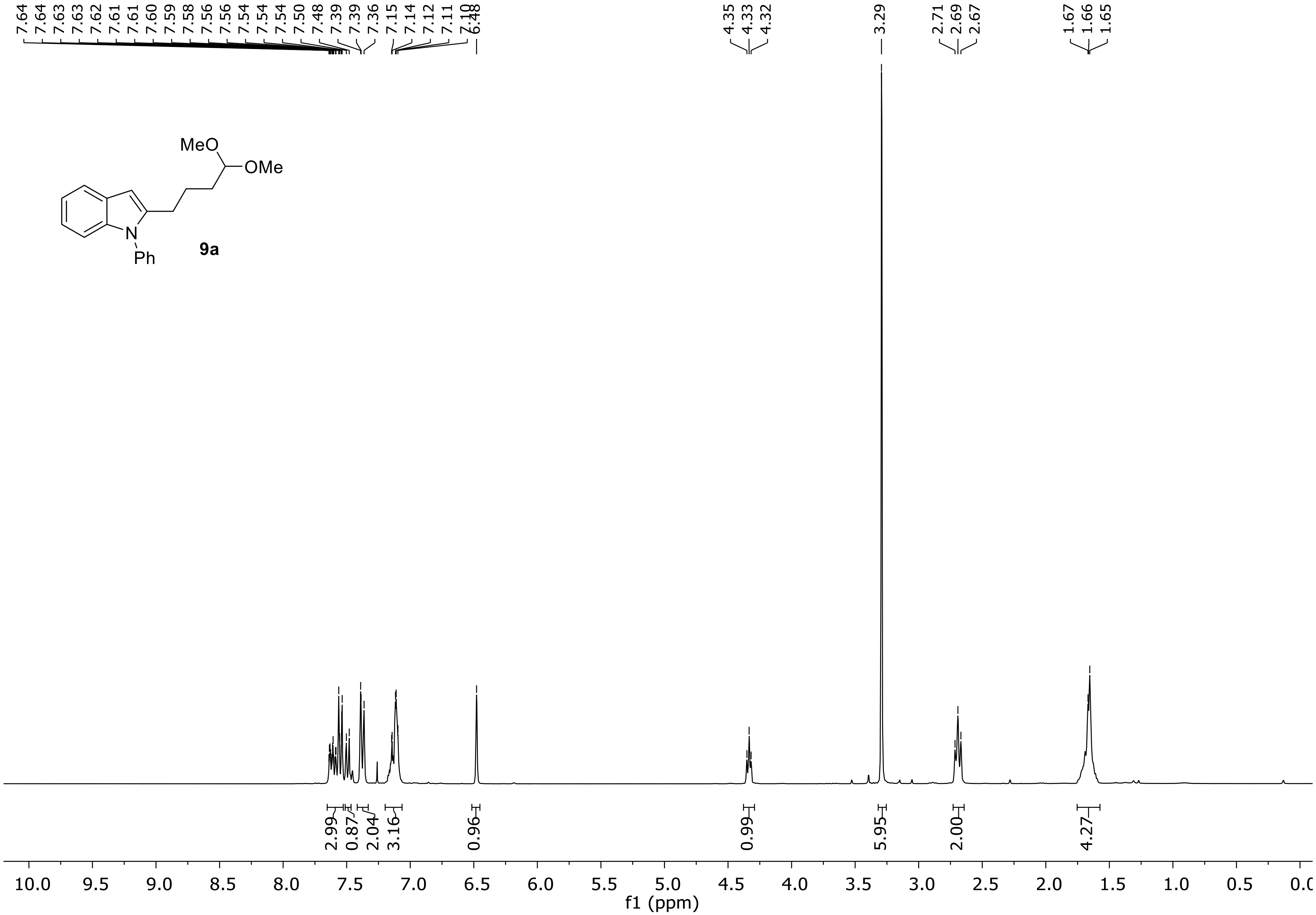

9a

$^{3}C\{^{1}H\}$-NMR (300 MHz, $CDCl_3$)

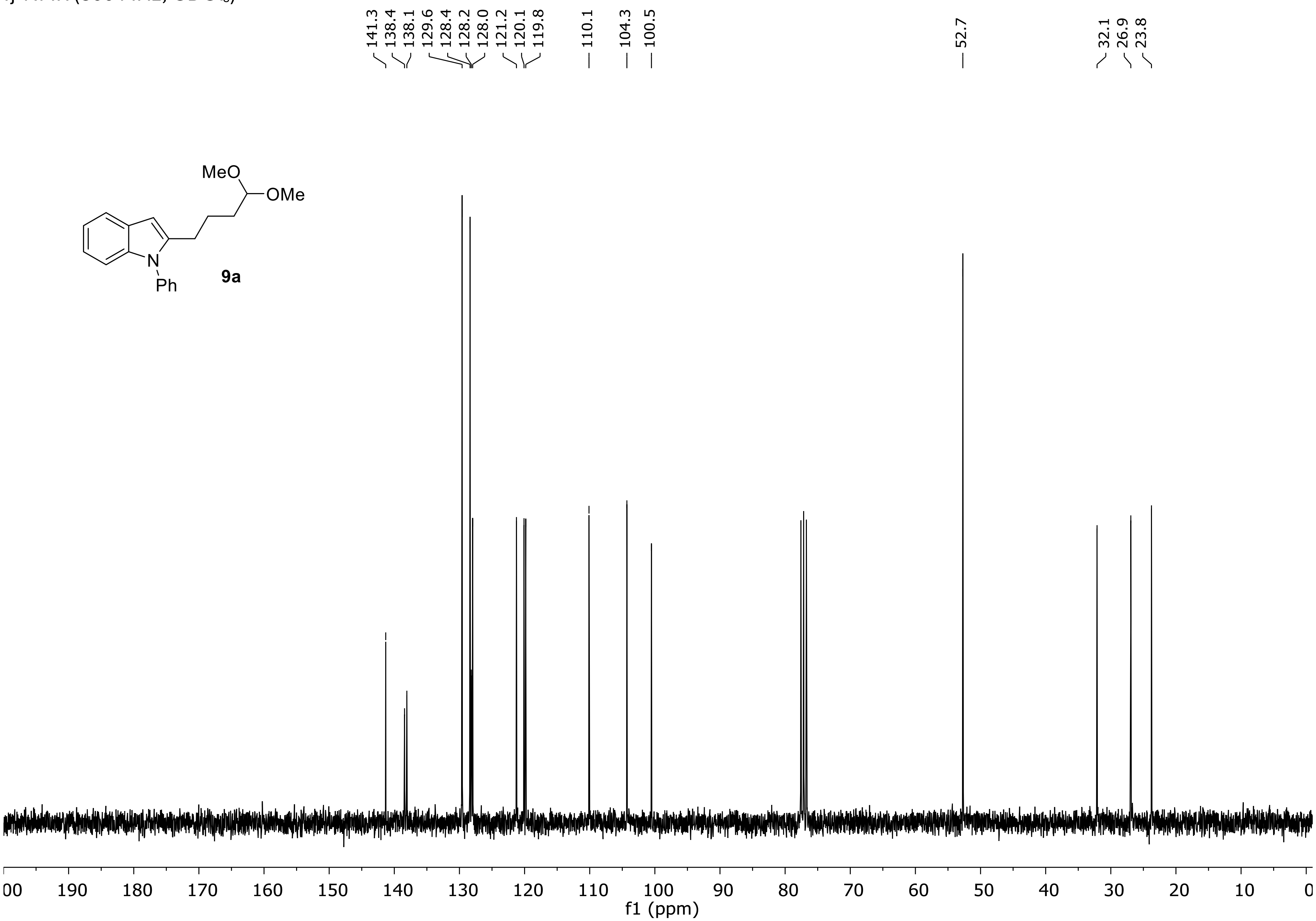

^{1}H-NMR (75.4 MHz, $CDCl_3$)

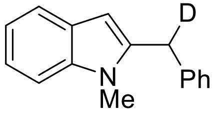

1o-D

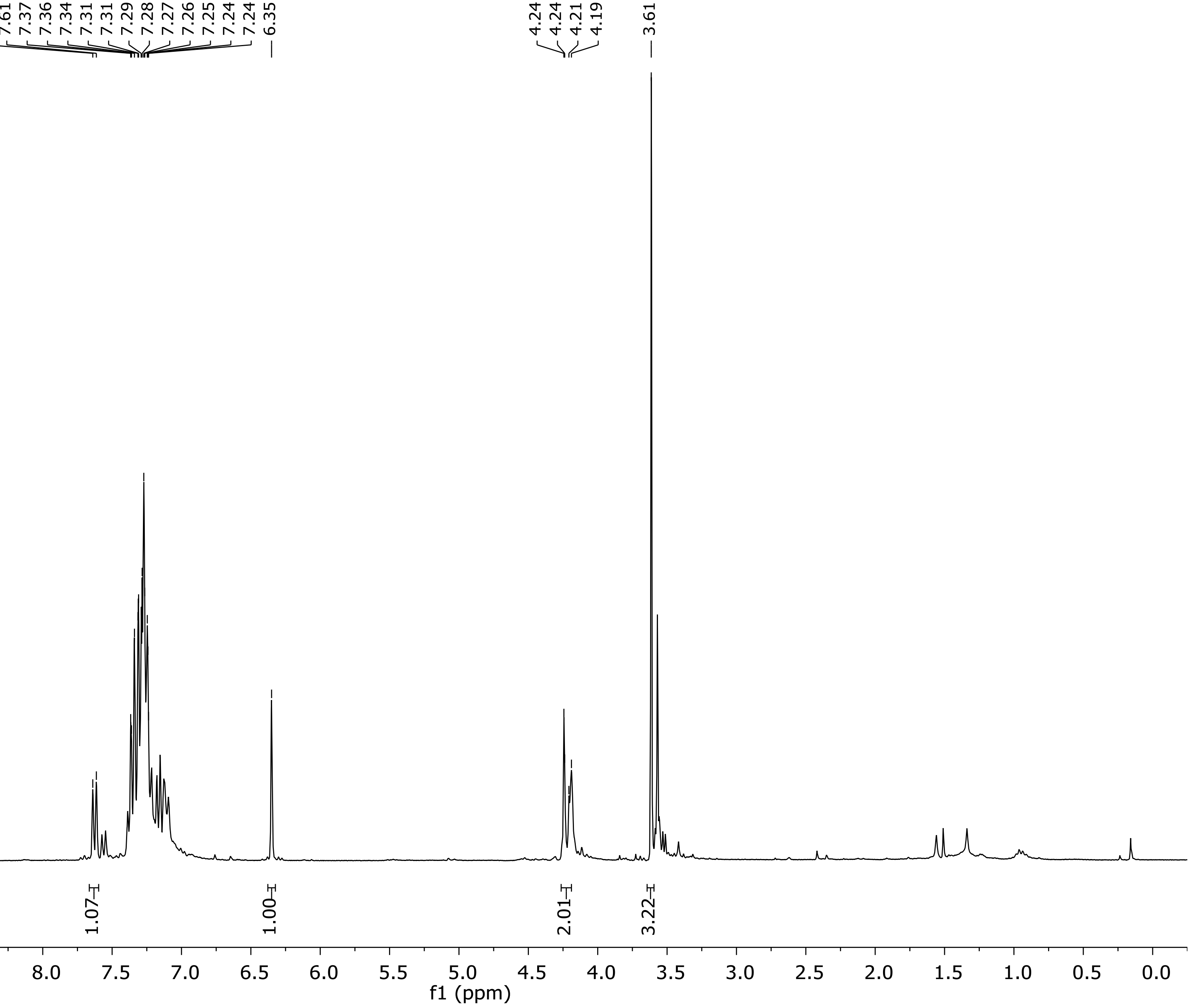

3C{1H}-NMR (300 MHz, $CDCl_3$)

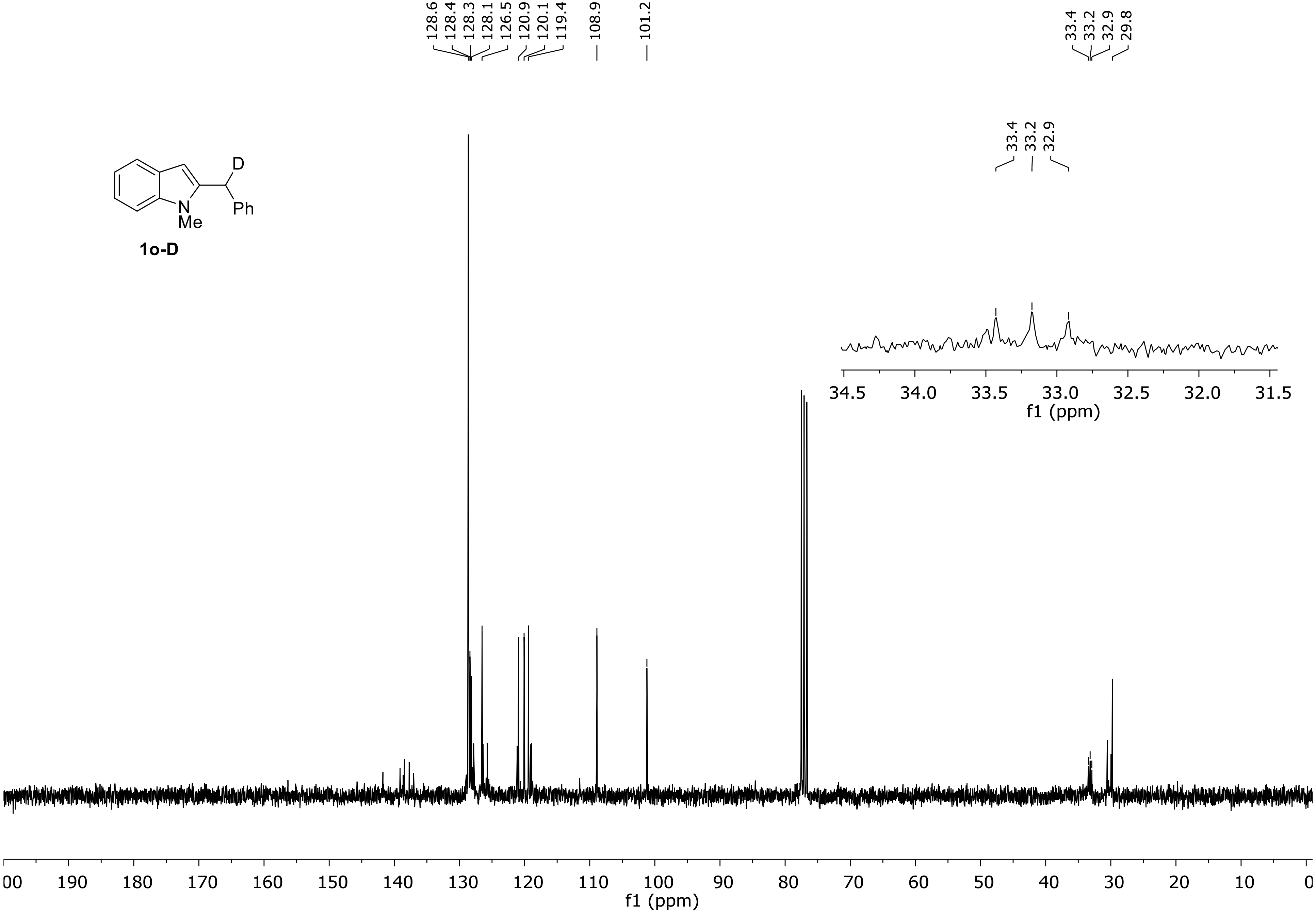

^{1}H-NMR (126 MHz, $CDCl_3$)

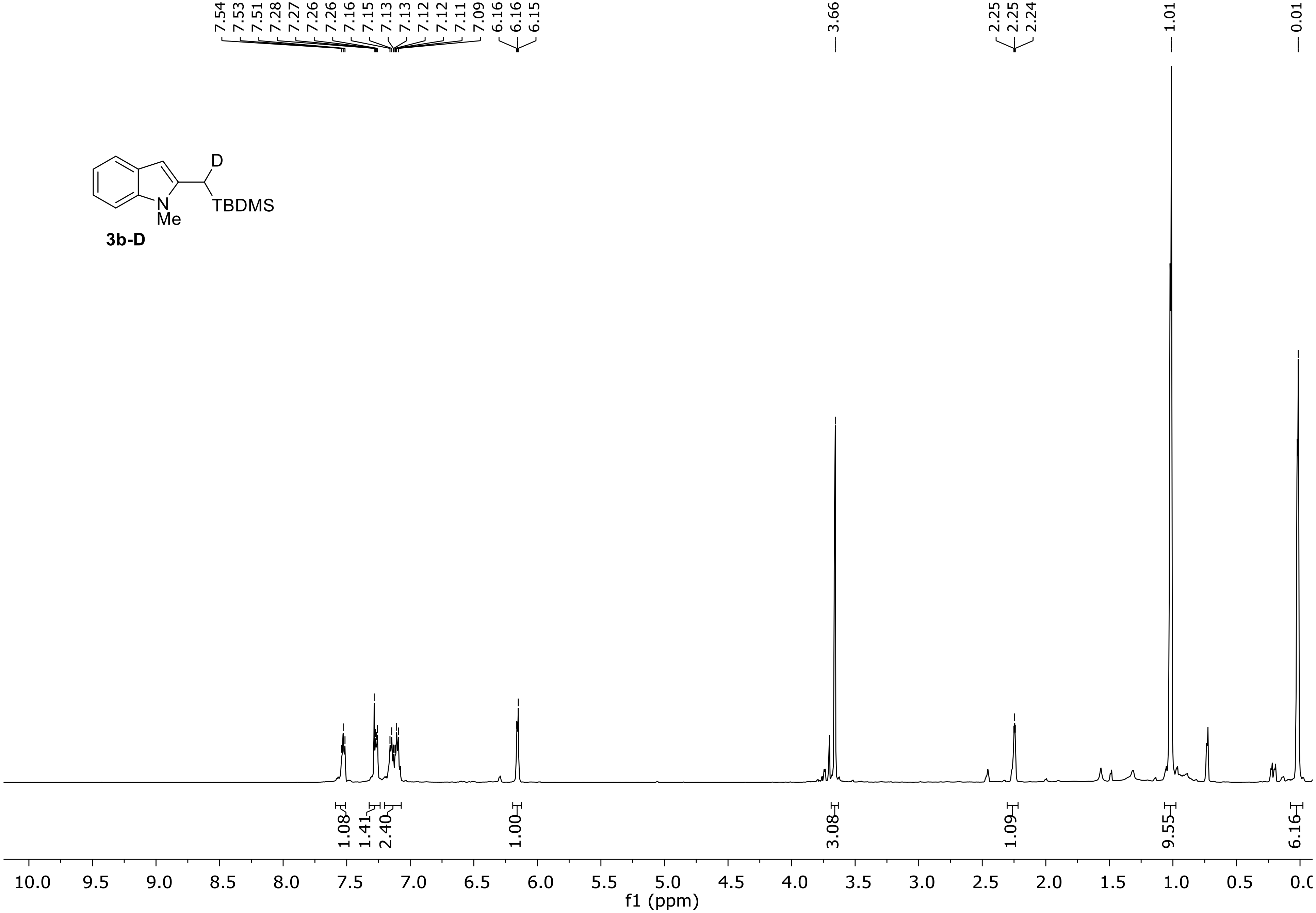

$^{13}C\{^{1}H\}$-NMR (500 MHz, $CDCl_3$)

3b-D

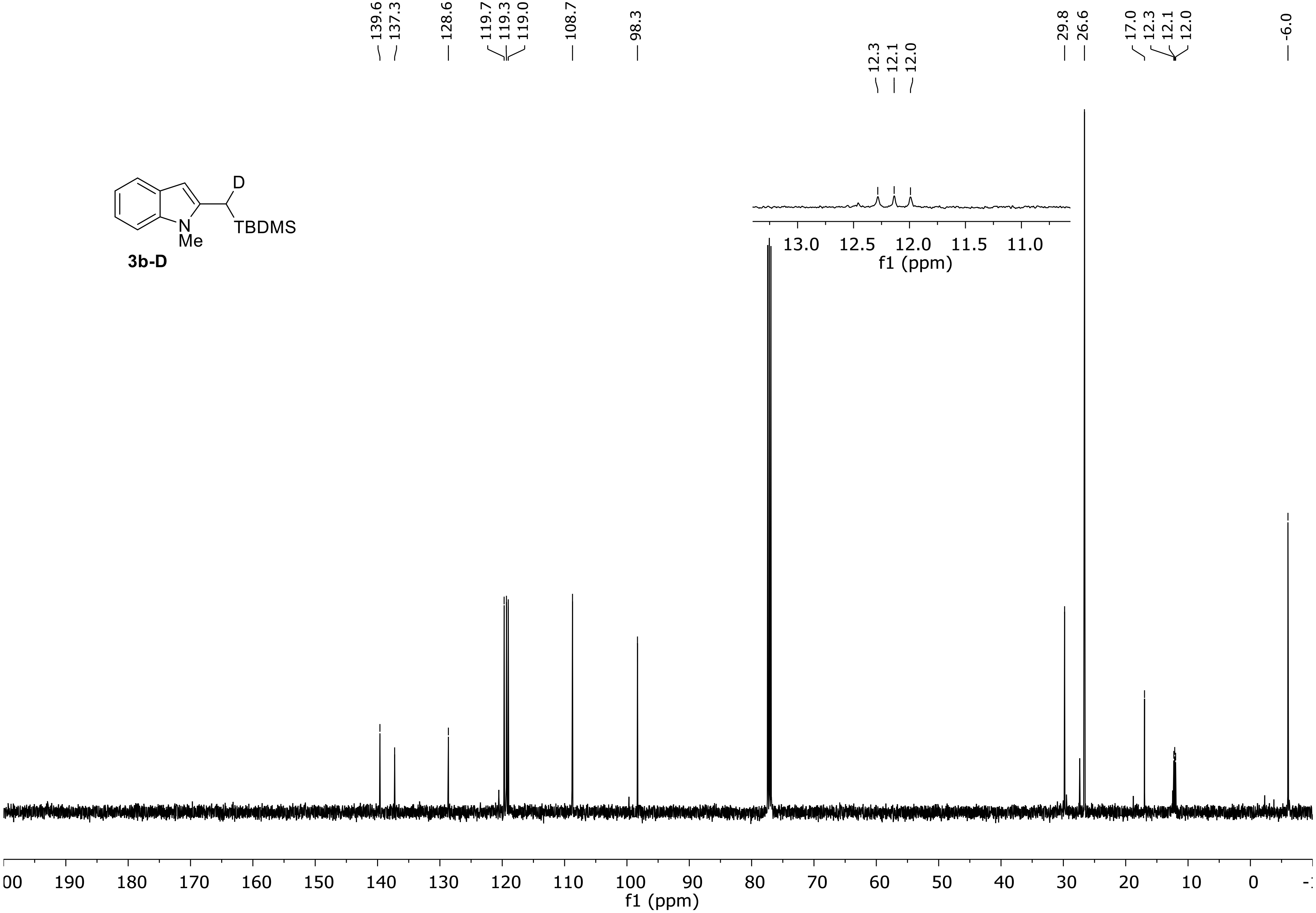

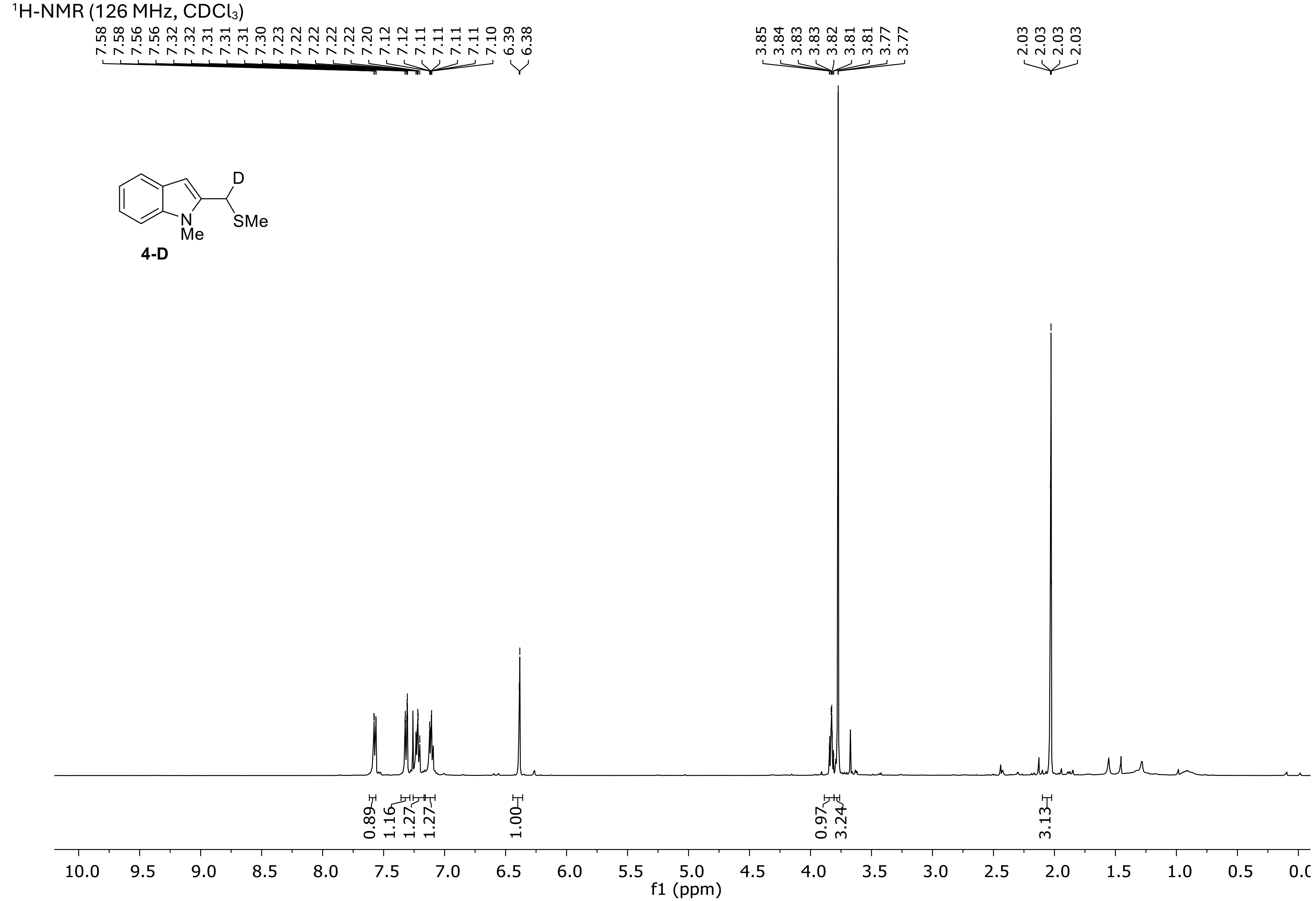

^{1}H-NMR (126 MHz, CDCl3)
7.58 7.58 7.56 7.56 7.32 7.32 7.31 7.31 7.31 7.30 7.23 7.22 7.22 7.22 7.22 7.20 7.12 7.12 7.11 7.11 7.11 7.11 7.10 6.39 6.38
3.85 3.84 3.83 3.83 3.82 3.81 3.81 3.77 3.77
2.03 2.03 2.03 2.03
D
N
Me
SMe
4-D
0.89 1.16 1.27 1.27 1.00 0.97 3.24 3.13
10.0 9.5 9.0 8.5 8.0 7.5 7.0 6.5 6.0 5.5 5.0 4.5 4.0 3.5 3.0 2.5 2.0 1.5 1.0 0.5 0.0
f1 (ppm)

$^{13}C\{^1H\}$-NMR (500 MHz, $CDCl_3$)

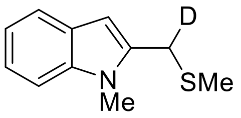

4-D

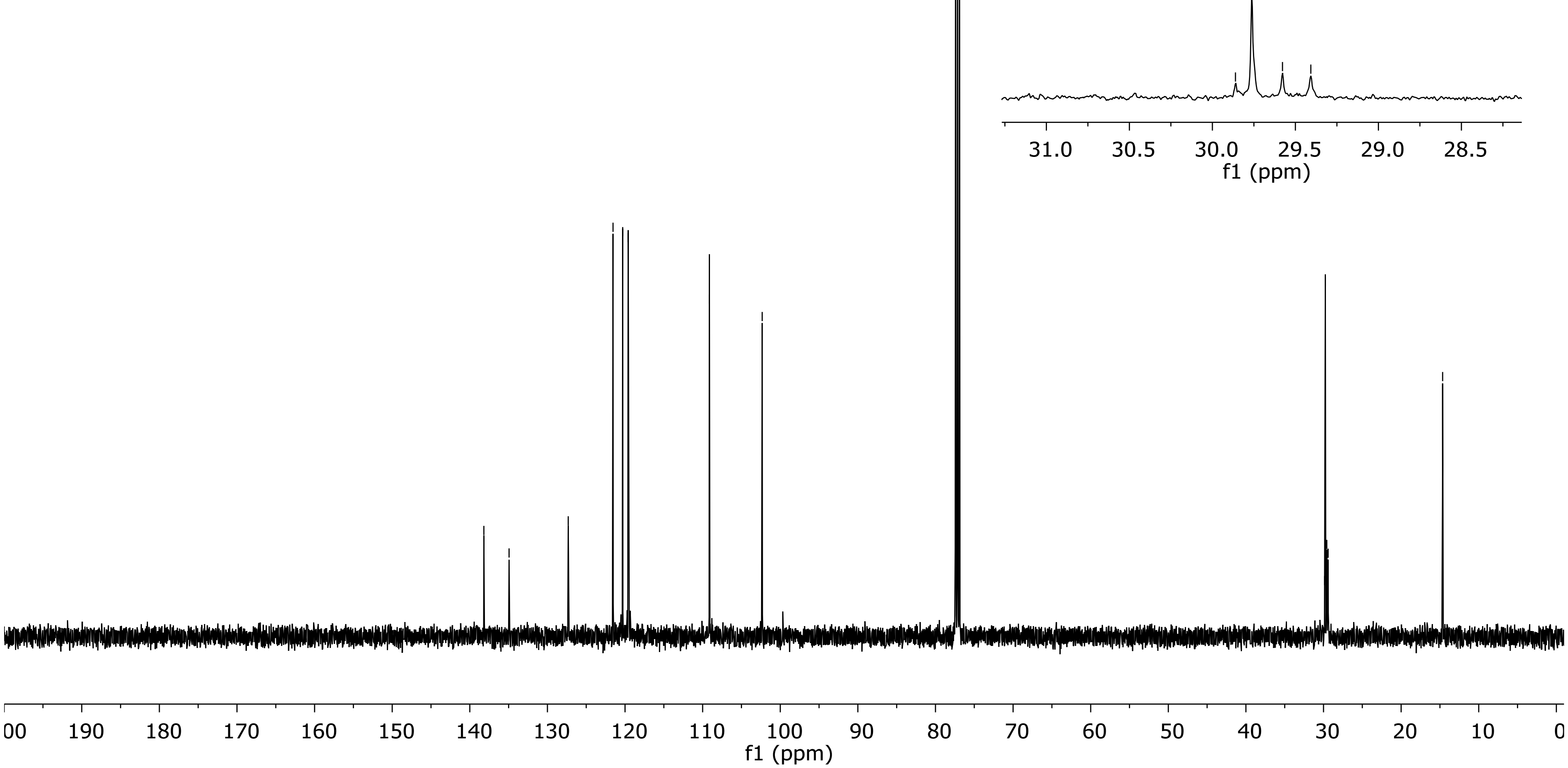

^{1}H-NMR (126 MHz, $CDCl_3$)

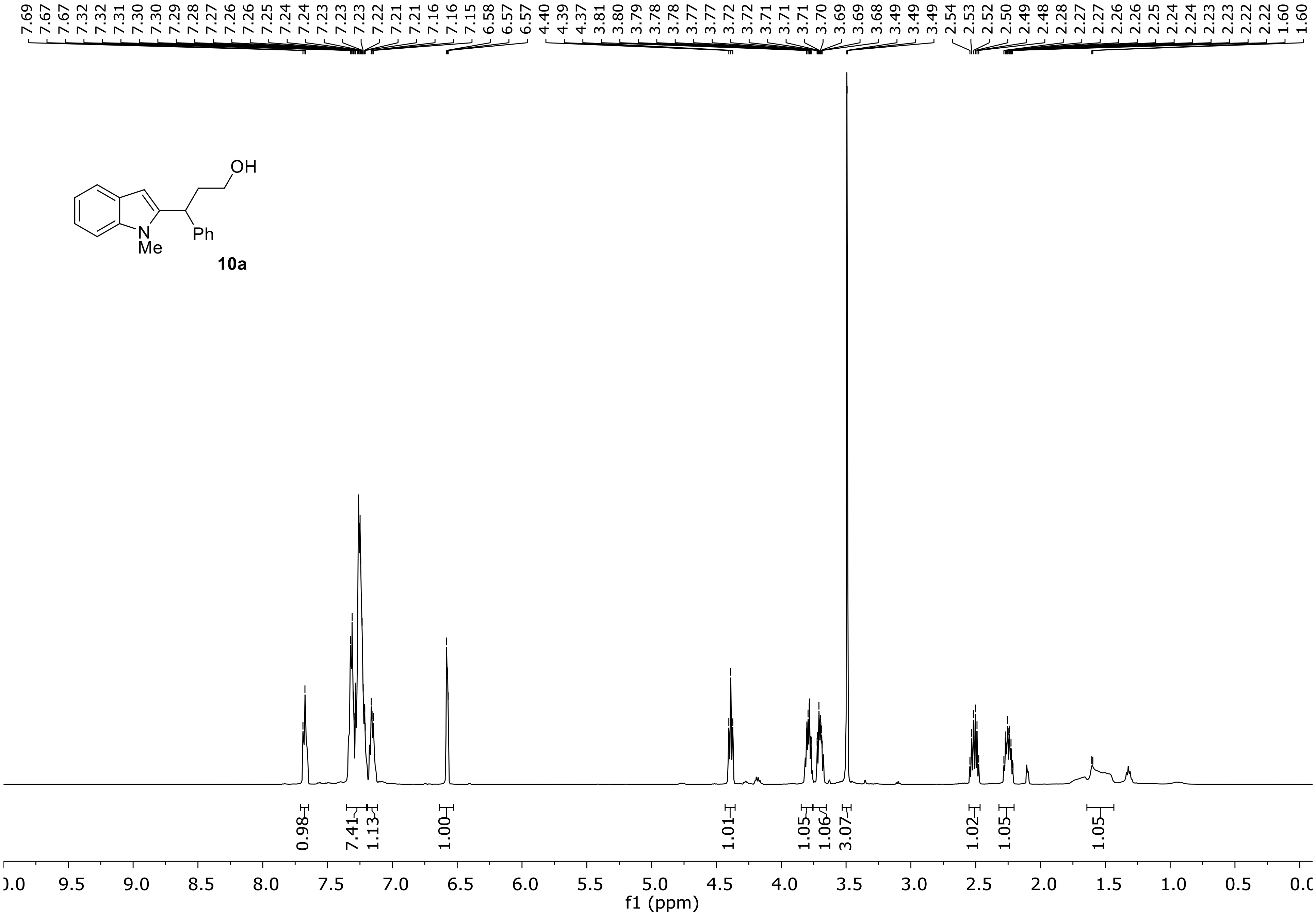

$^{13}C\{^1H\}$-NMR (500 MHz, $CDCl_3$)

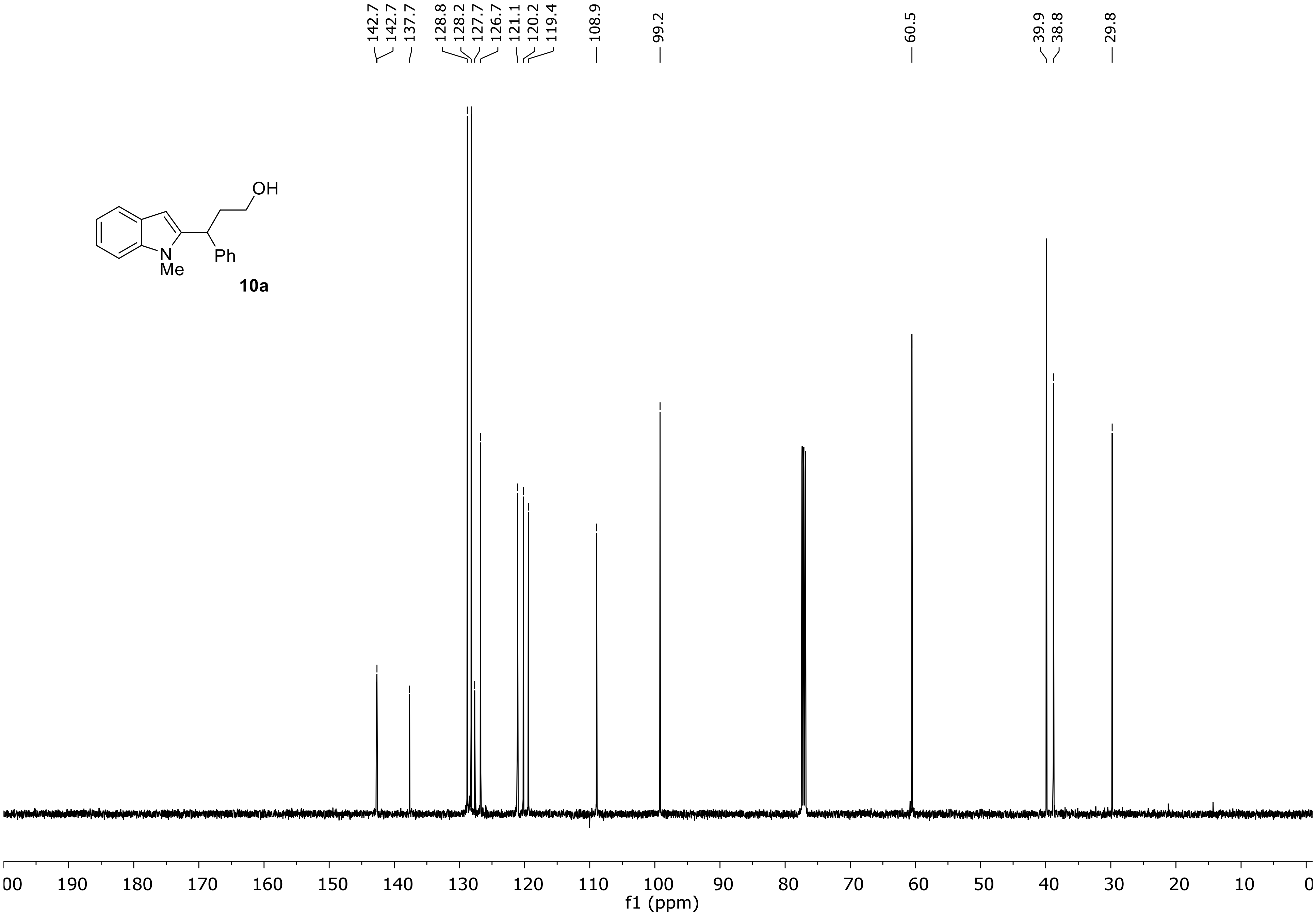

10a

^{1}H-NMR (126 MHz, $CDCl_3$)

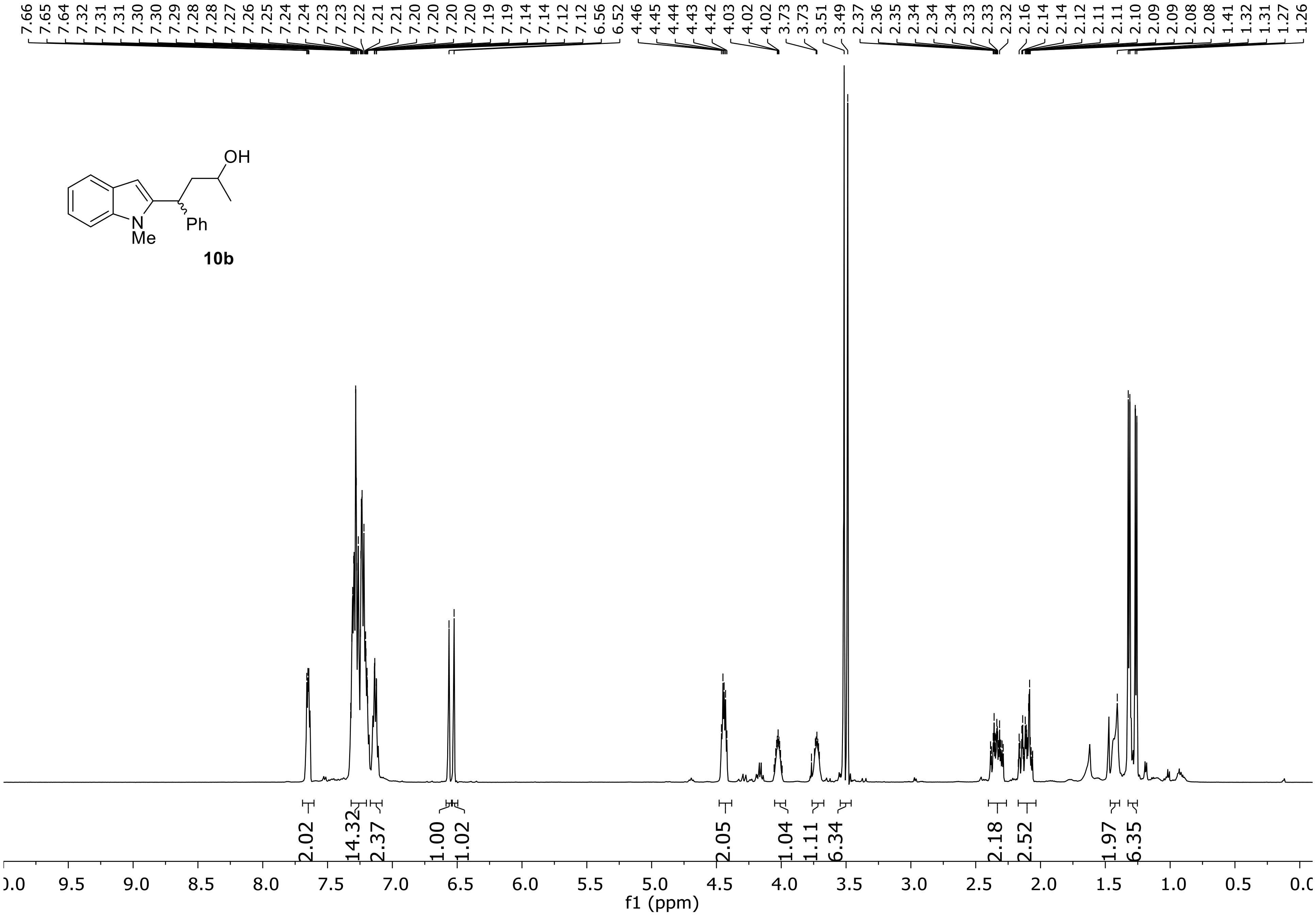

10b

$^{13}C\{^{1}H\}$-NMR (500 MHz, $CDCl_3$)

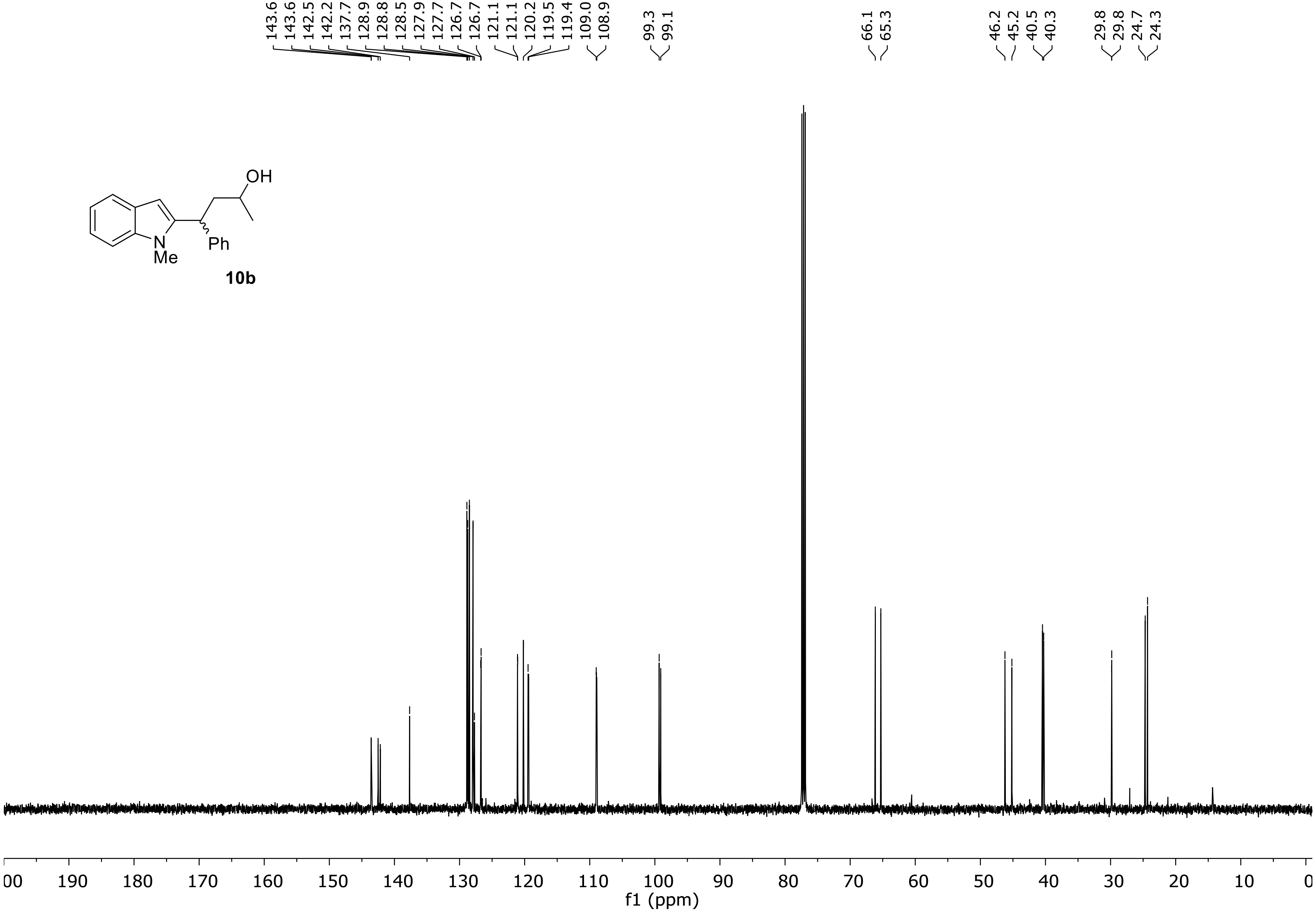

^{1}H-NMR (126 MHz, $CDCl_3$)

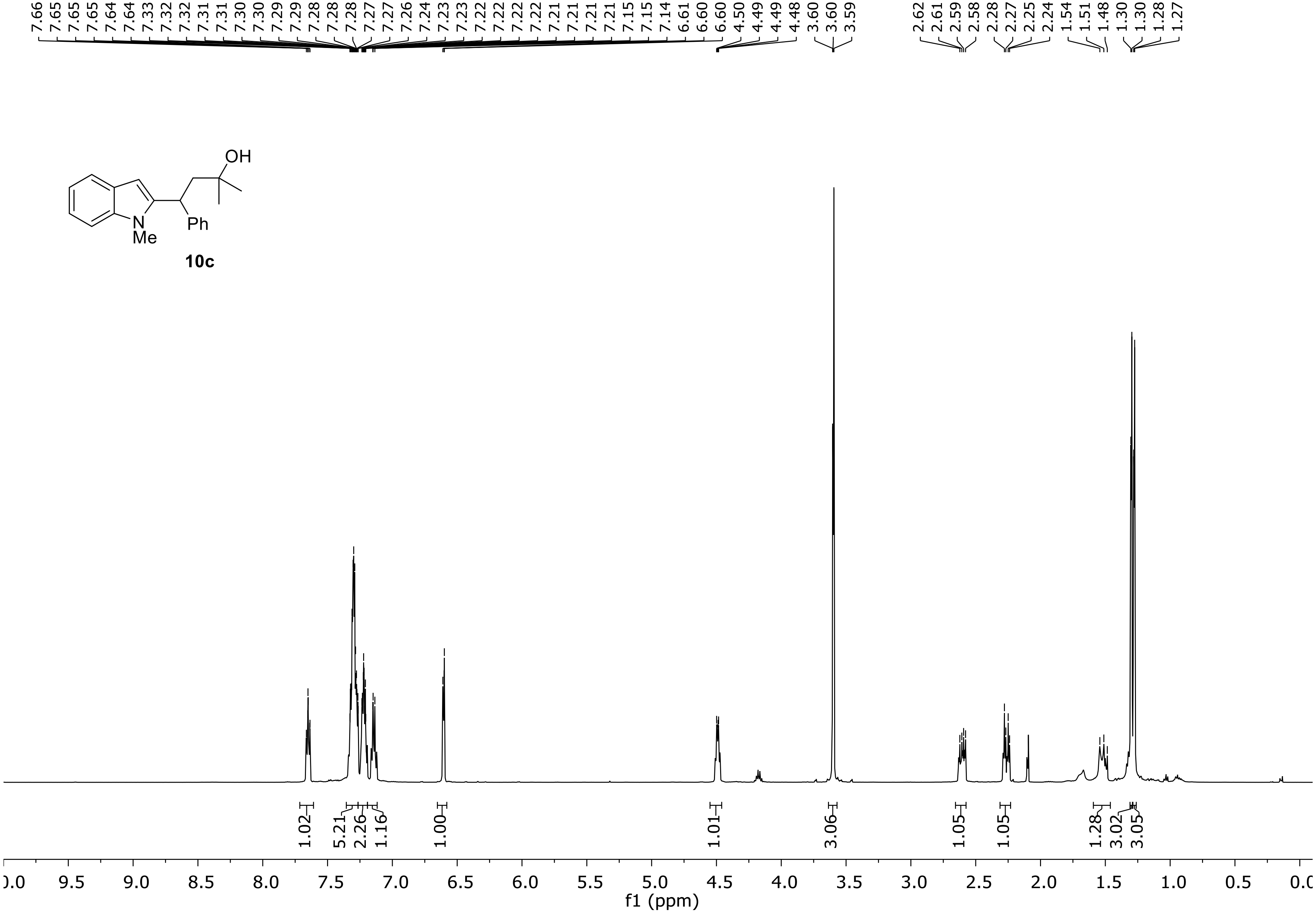

$^{13}C\{^1H\}$-NMR (500 MHz, $CDCl_3$)

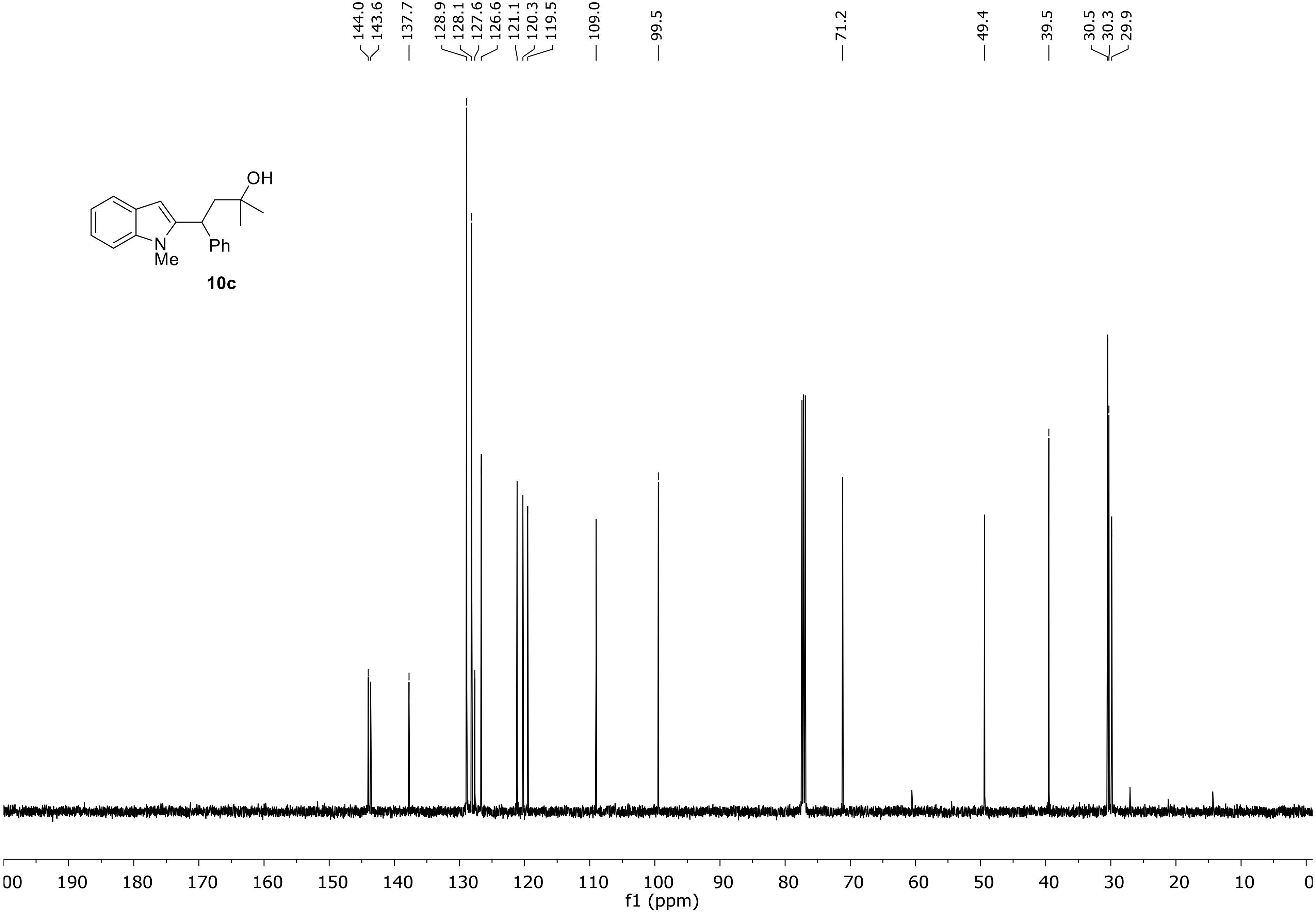

^{1}H-NMR (126 MHz, $CDCl_3$)

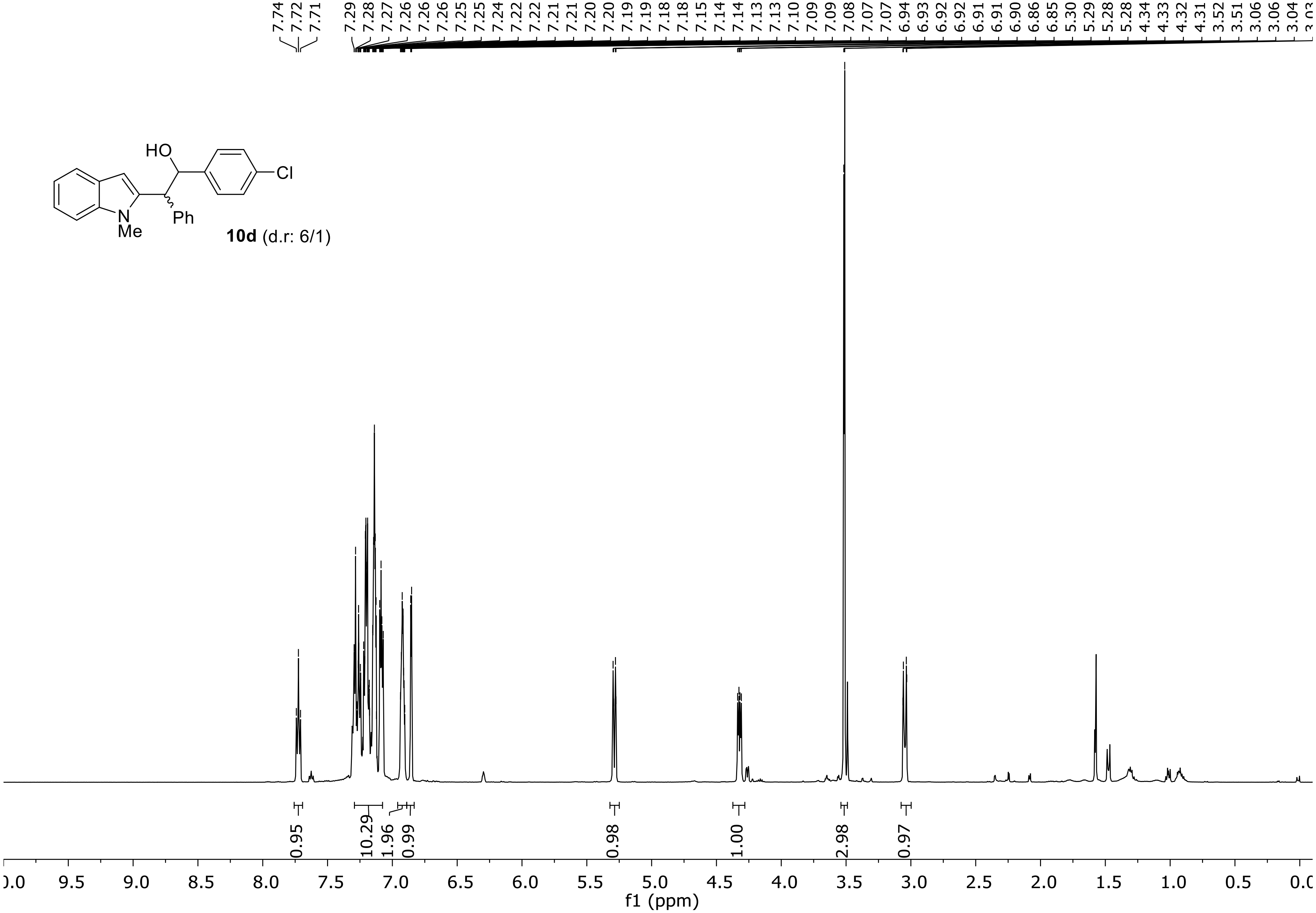

$^{13}C\{^{1}H\}$-NMR (500 MHz, $CDCl_3$)

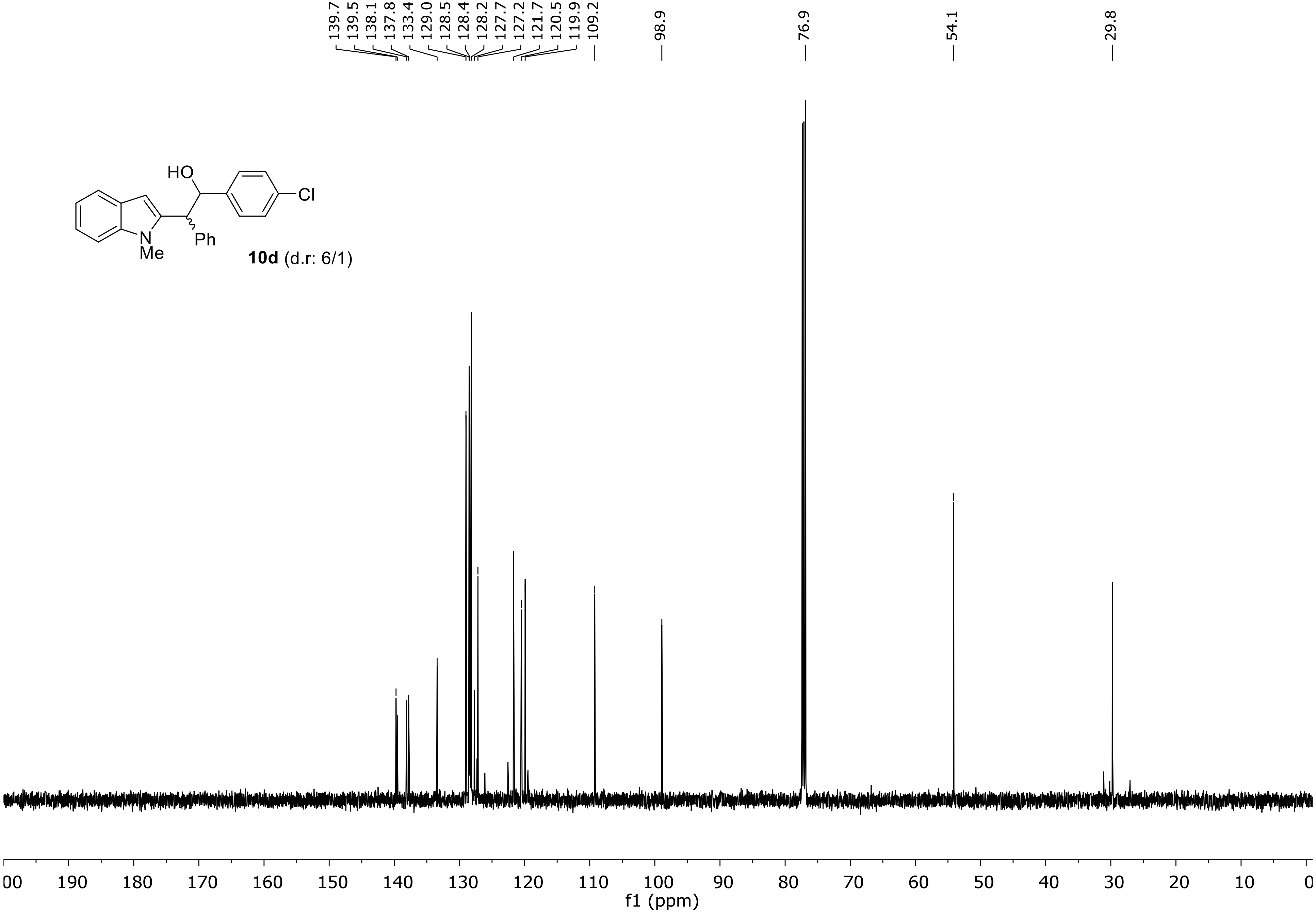

^{1}H-NMR (75.4 MHz, $CDCl_3$)

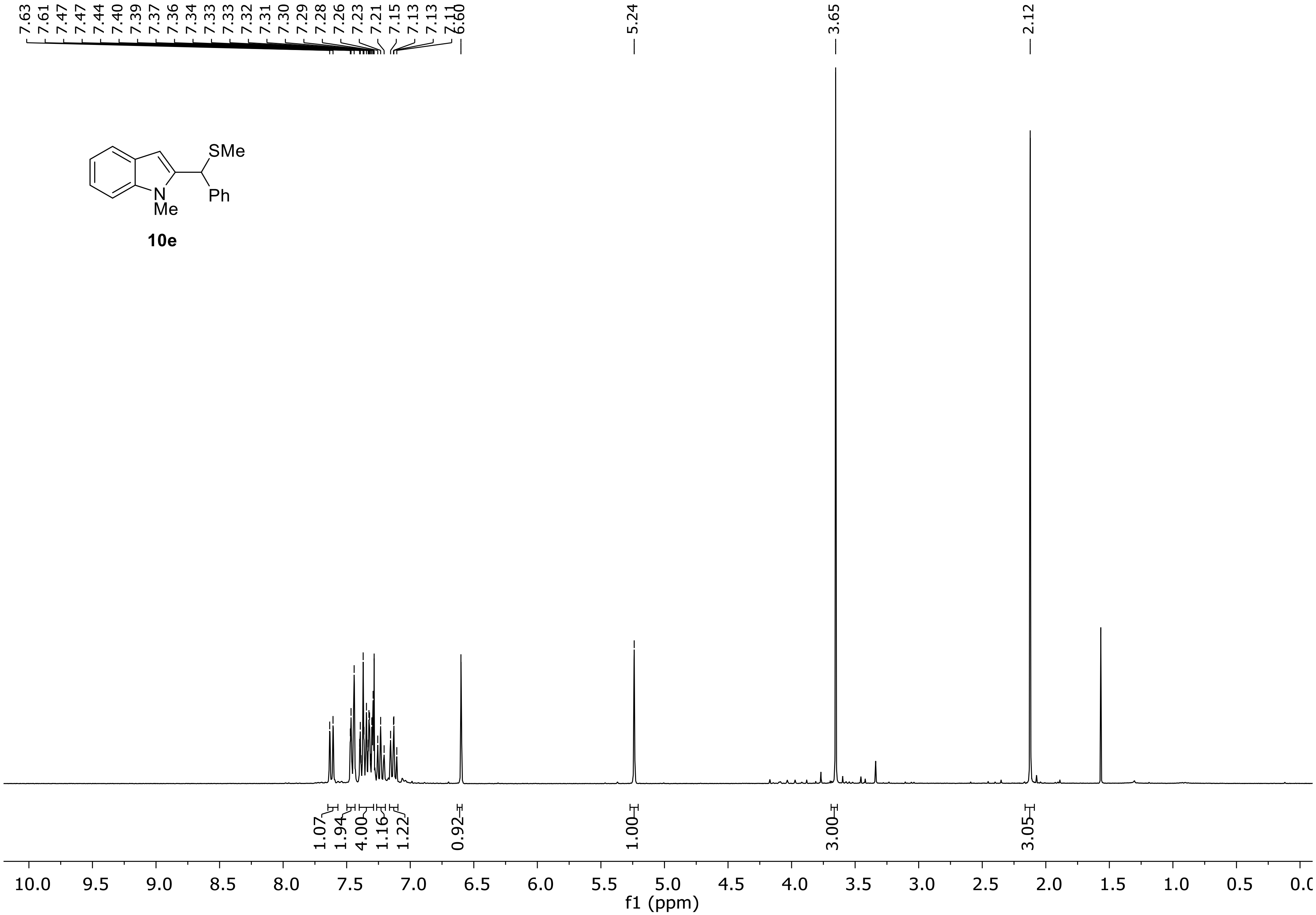

$^{13}C\{^{1}H\}$-NMR (300 MHz, $CDCl_3$)

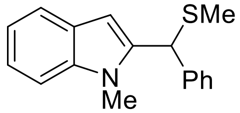

10e

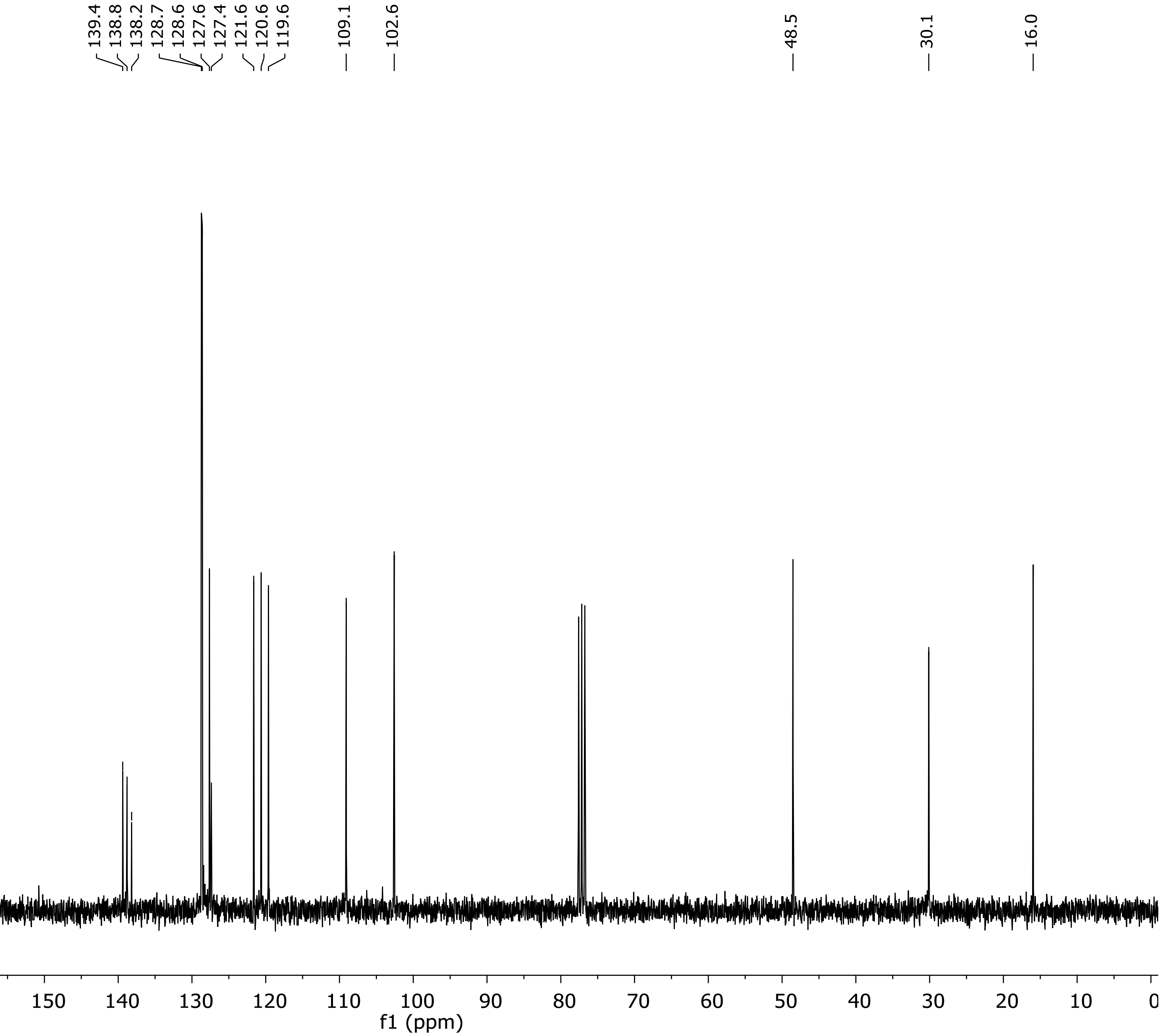

^{1}H-NMR (75.4 MHz, $CDCl_3$)

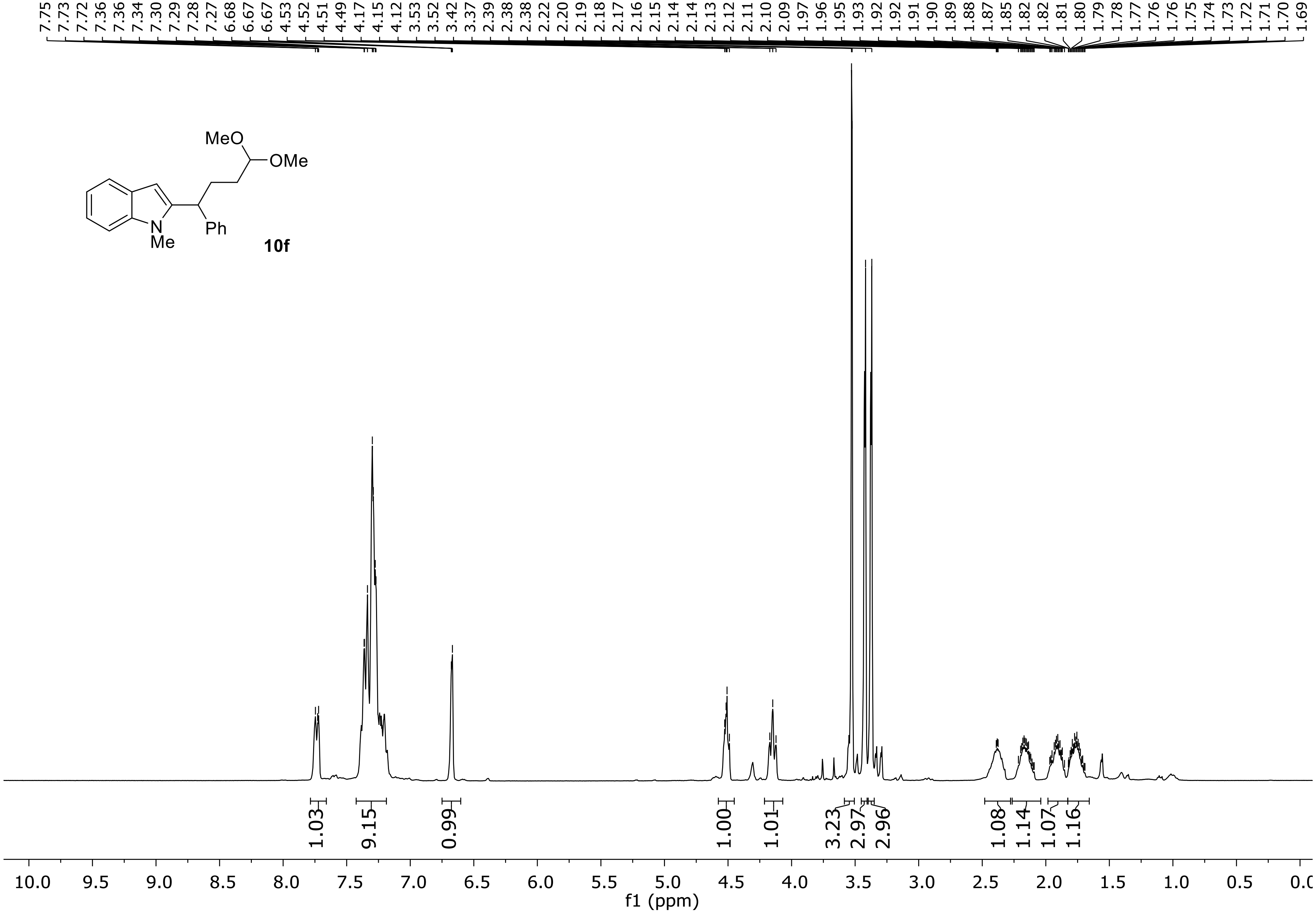

$^{13}C\{^1H\}$-NMR (300 MHz, $CDCl_3$)

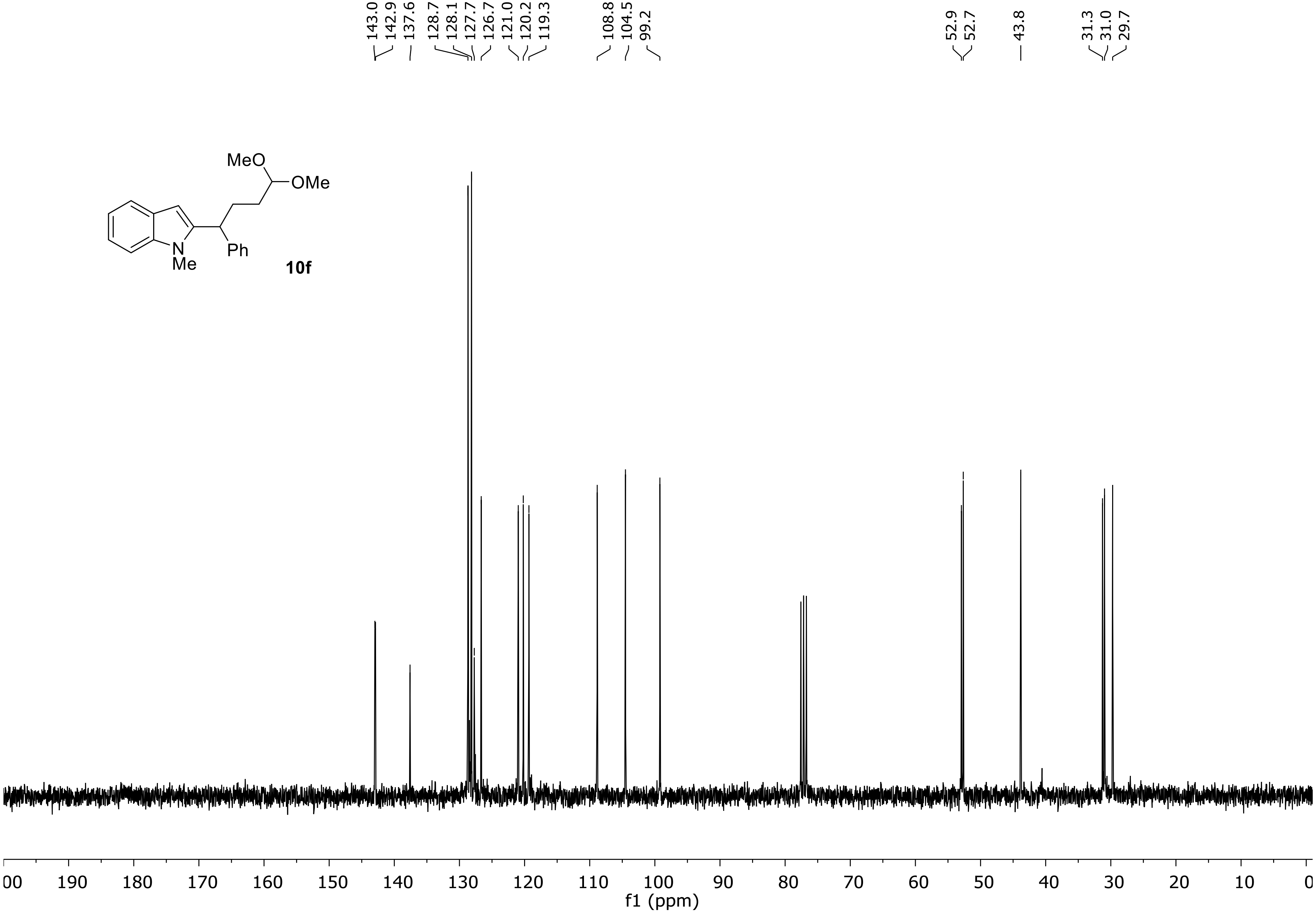

10f

^{1}H-NMR (75.4 MHz, $CDCl_3$)

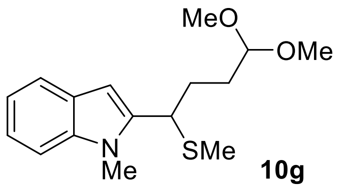

10g

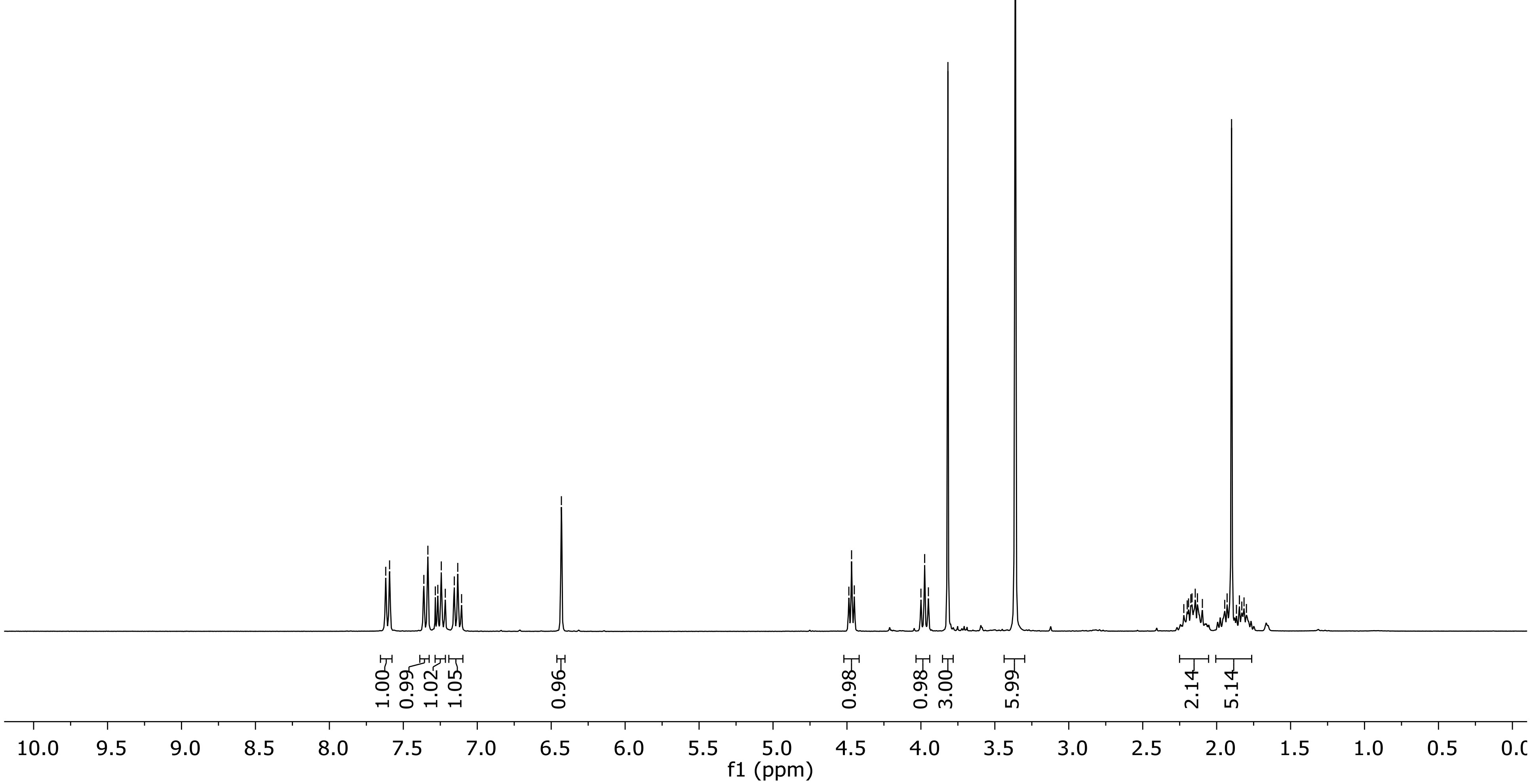

$^{13}C\{^{1}H\}$-NMR (300 MHz, $CDCl_3$)

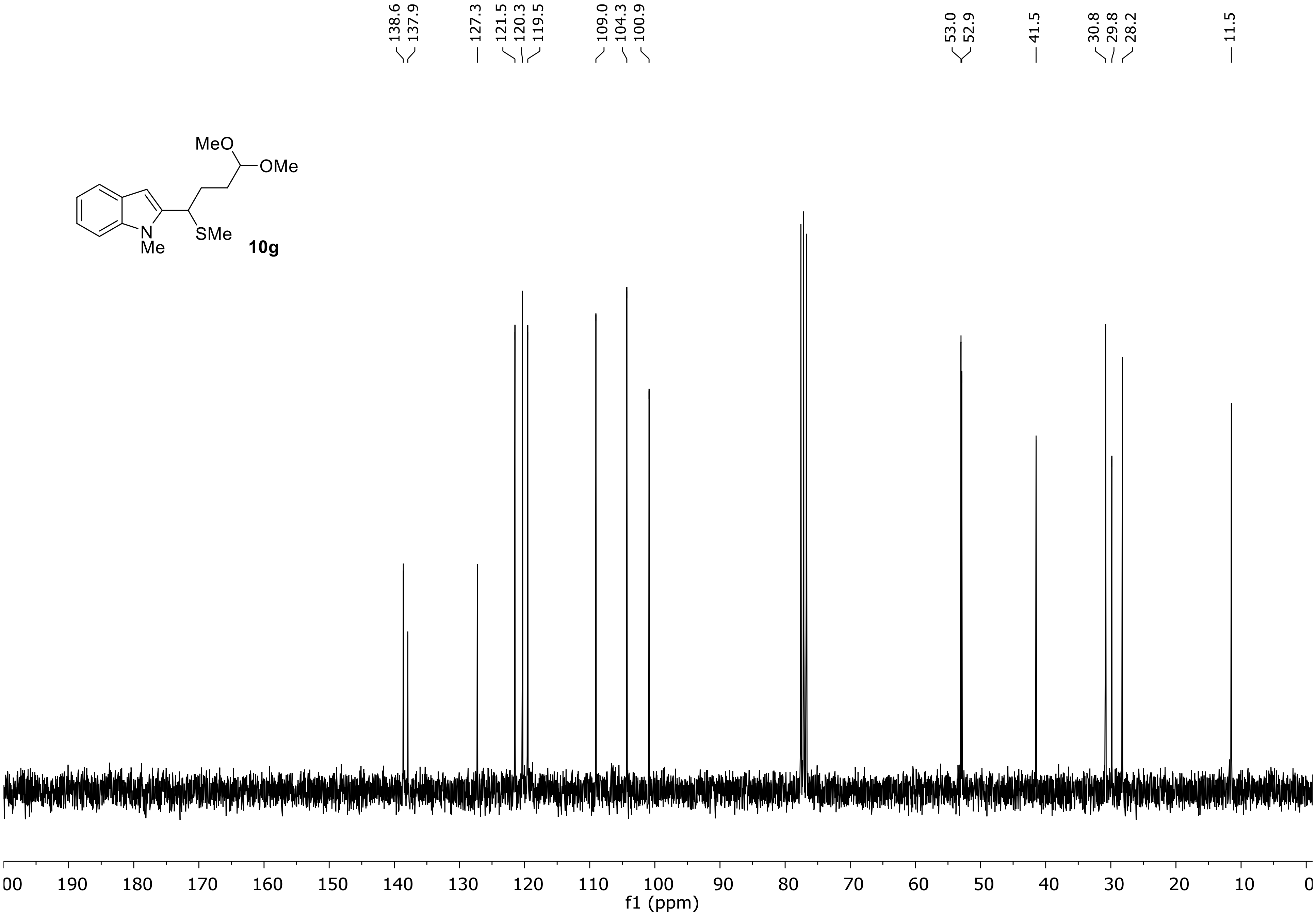

^{1}H-NMR (75.4 MHz, $CDCl_3$)

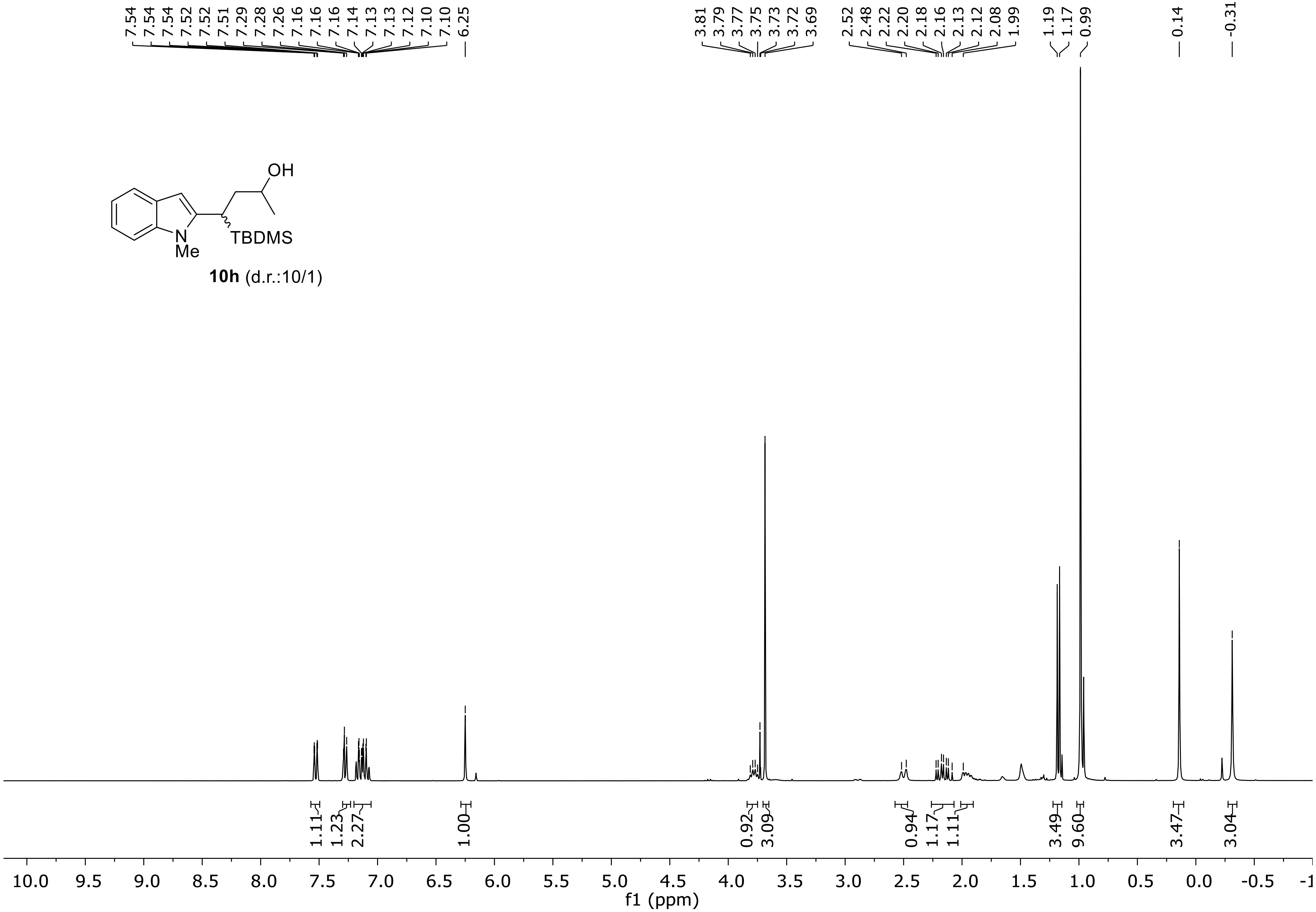

$^{13}C\{^{1}H\}$-NMR (300 MHz, $CDCl_3$)

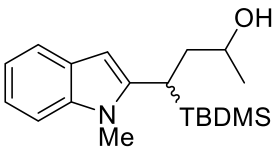

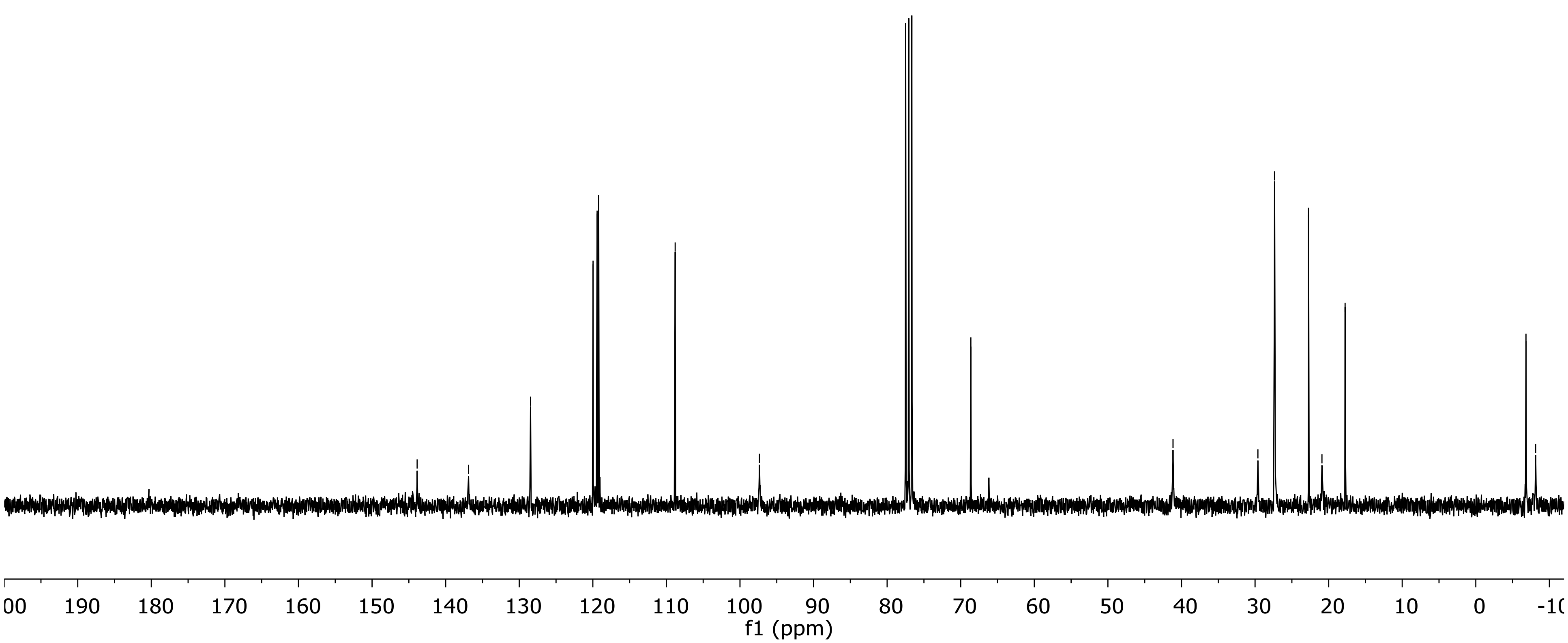

10h (d.r.:10/1)

^{1}H-NMR (75.4 MHz, $CDCl_3$)

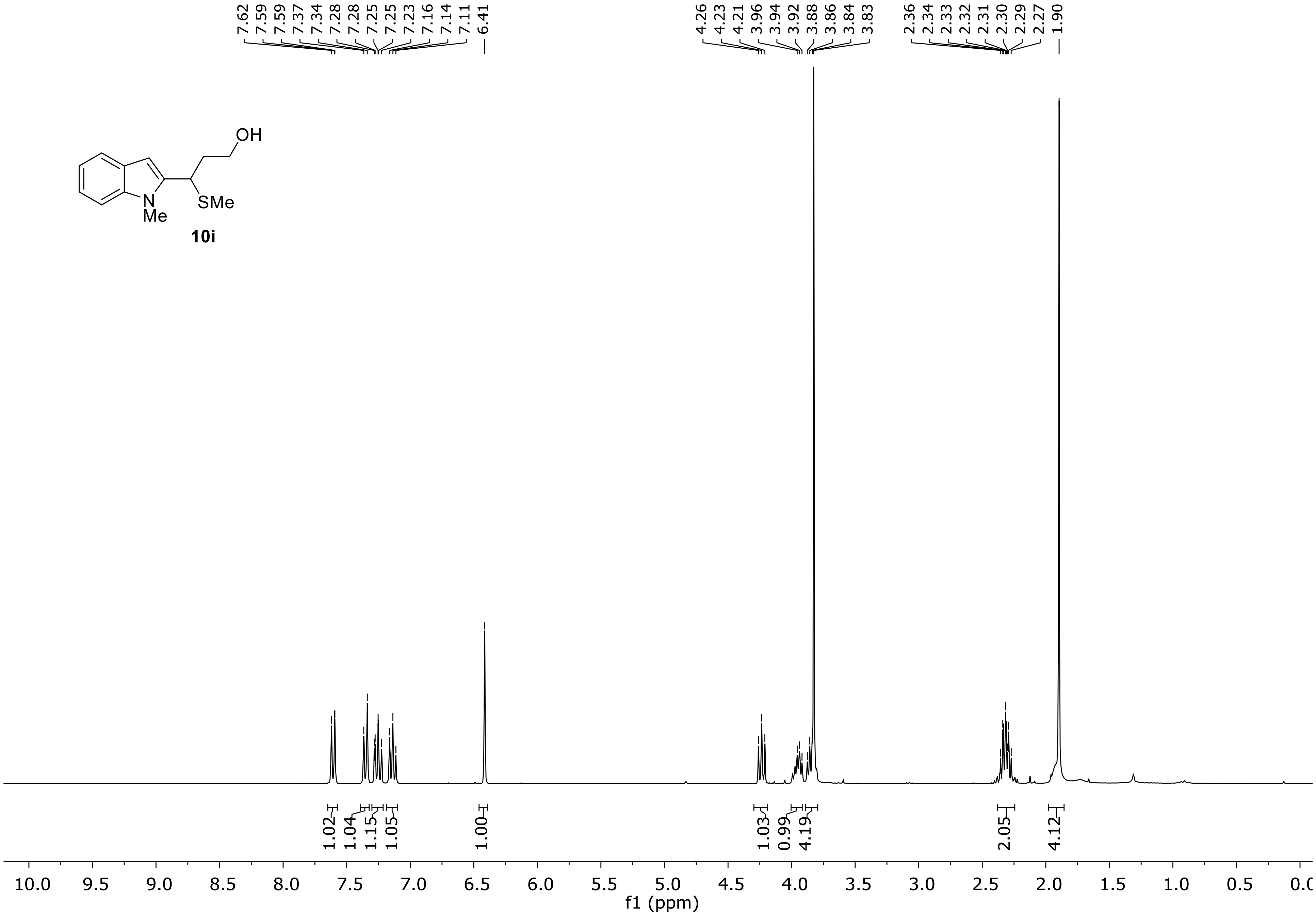

10i

$^{13}C\{^{1}H\}$-NMR (300 MHz, $CDCl_3$)

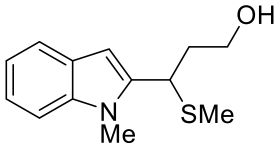

10i

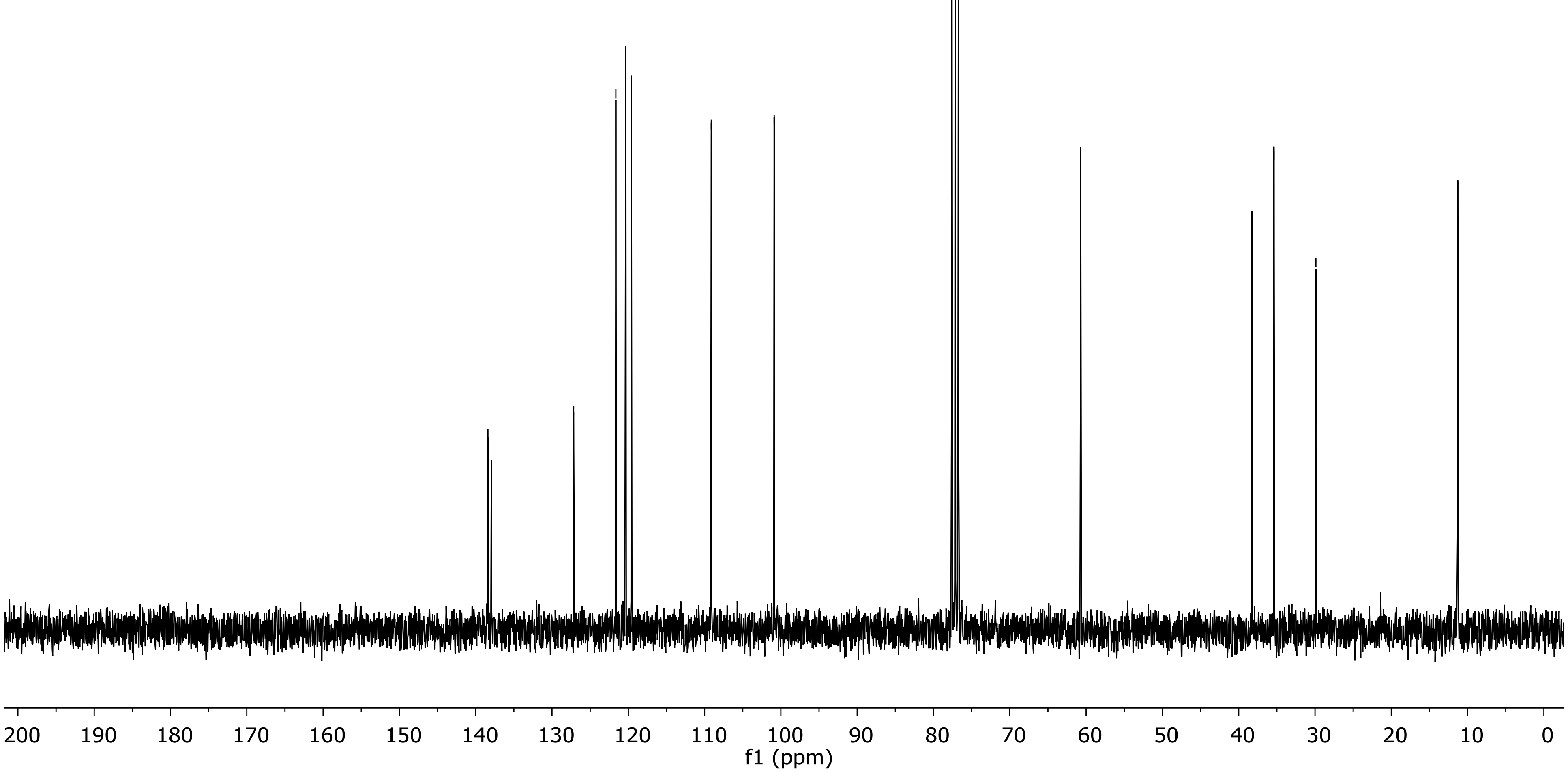

^{1}H-NMR (75.4 MHz, $CDCl_3$)

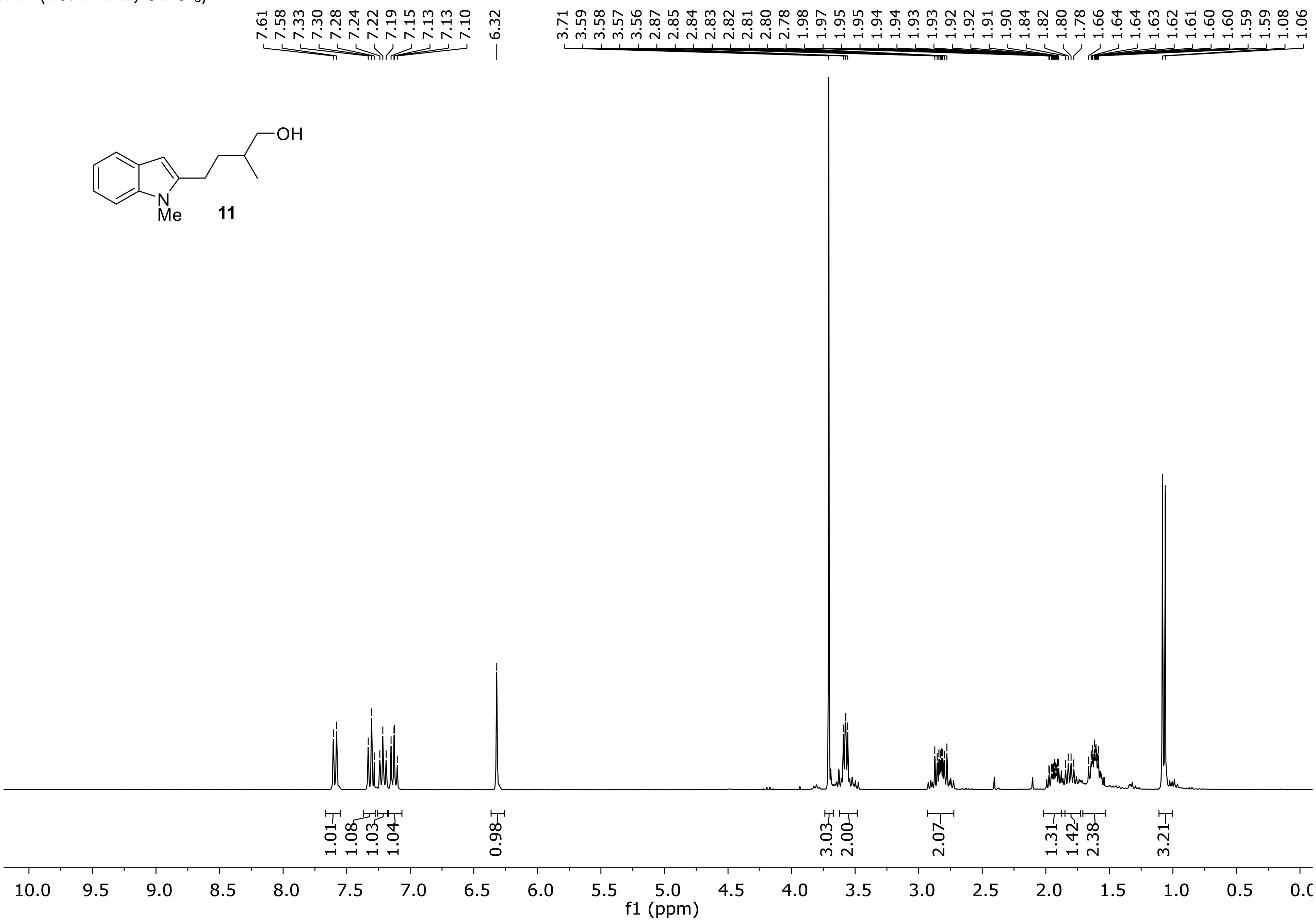

$^{13}C\{^{1}H\}$-NMR (300 MHz, $CDCl_3$)

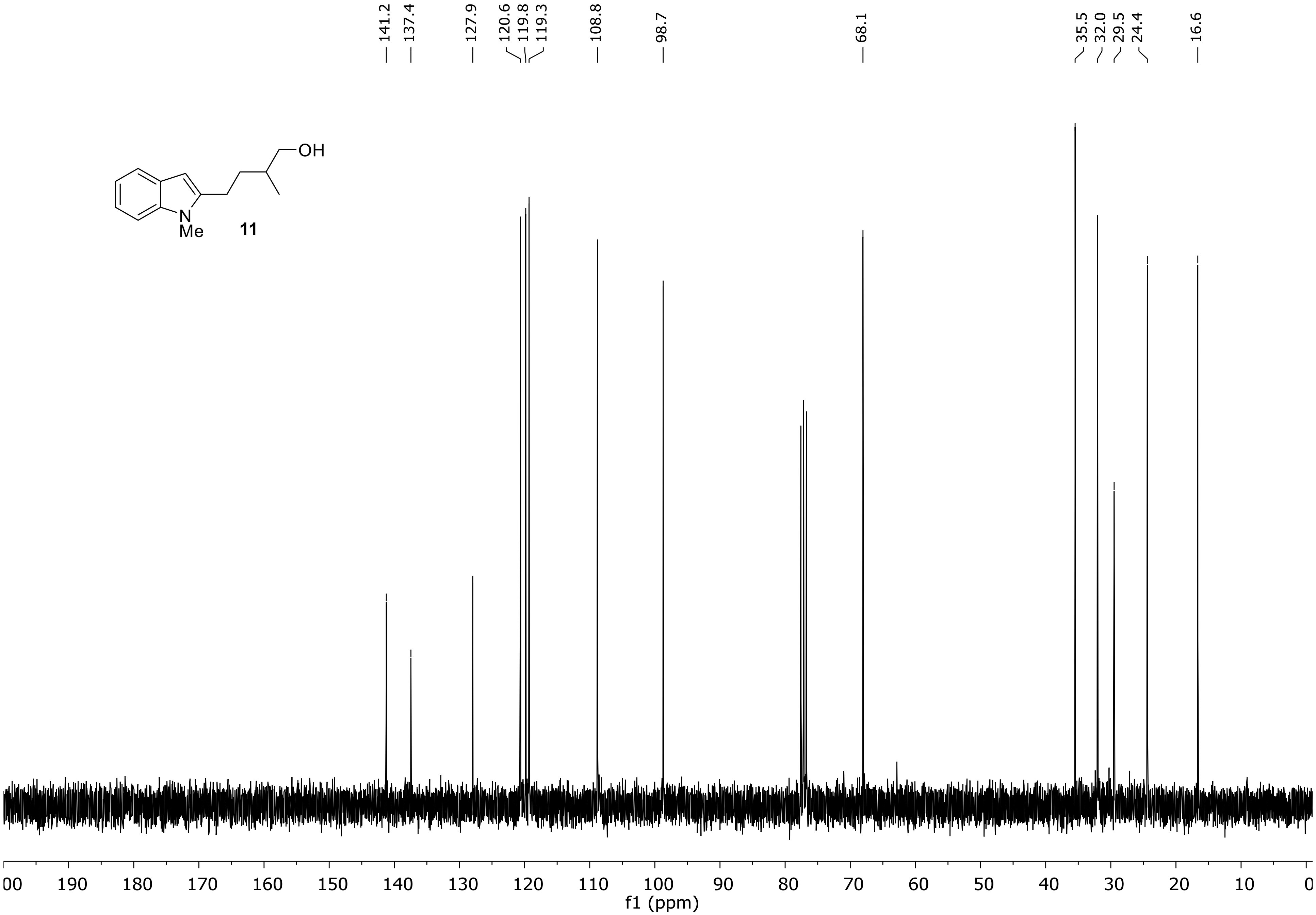

^{1}H-NMR (126 MHz, $CDCl_3$)

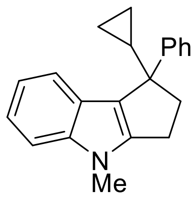

12

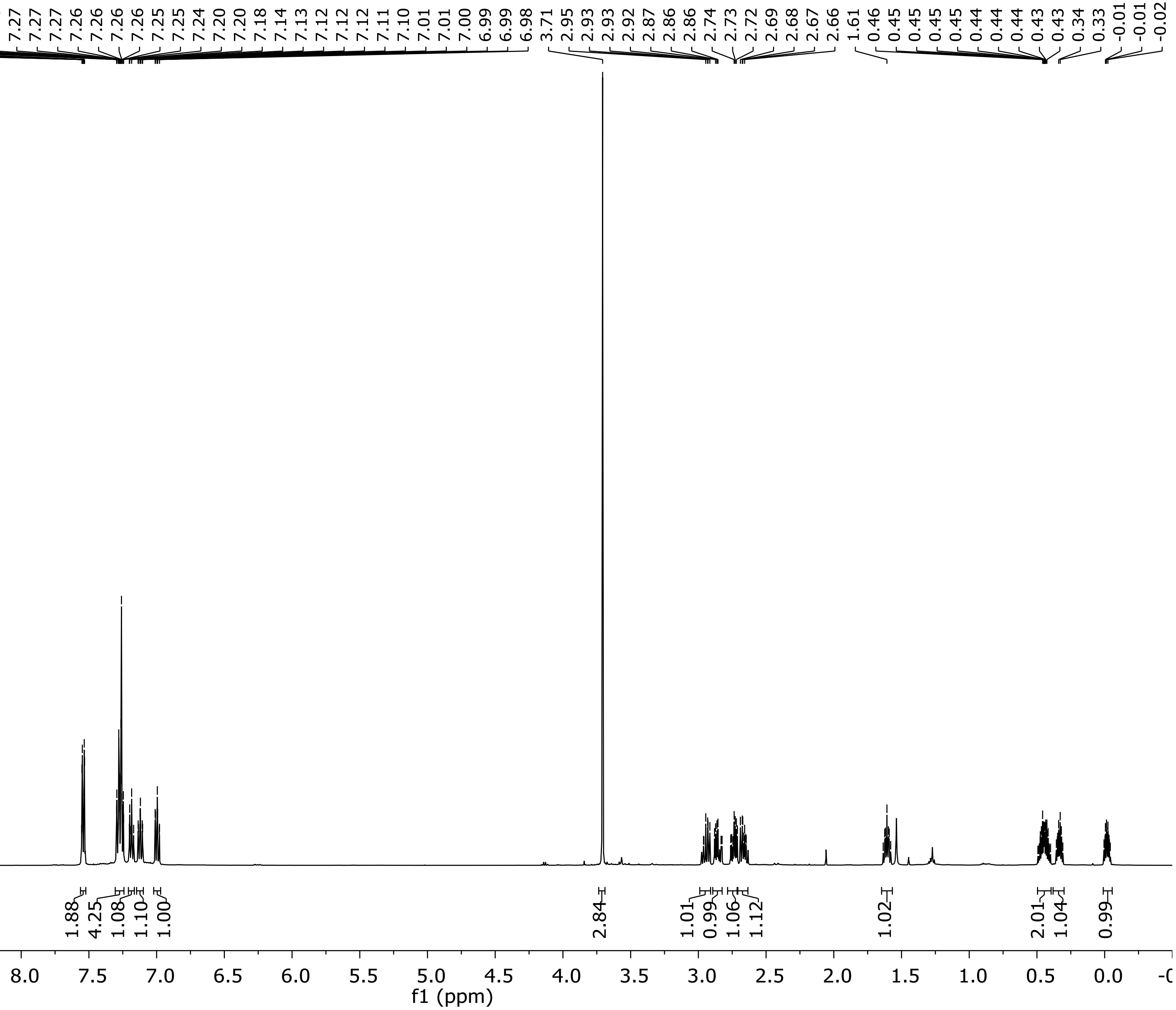

$^{13}C\{^1H\}$-NMR (500 MHz, $CDCl_3$)

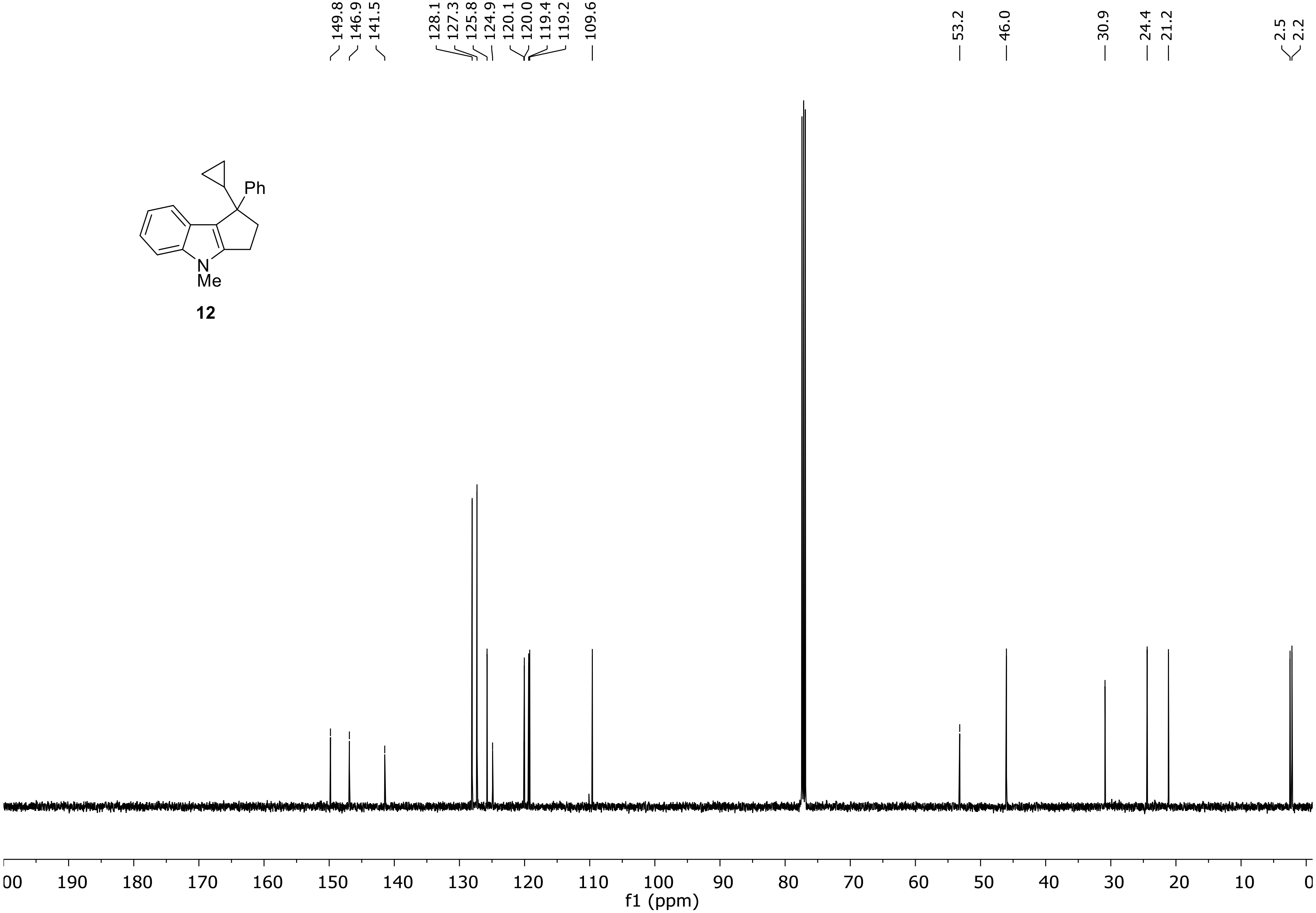

^{1}H-NMR (126 MHz, $CDCl_3$)

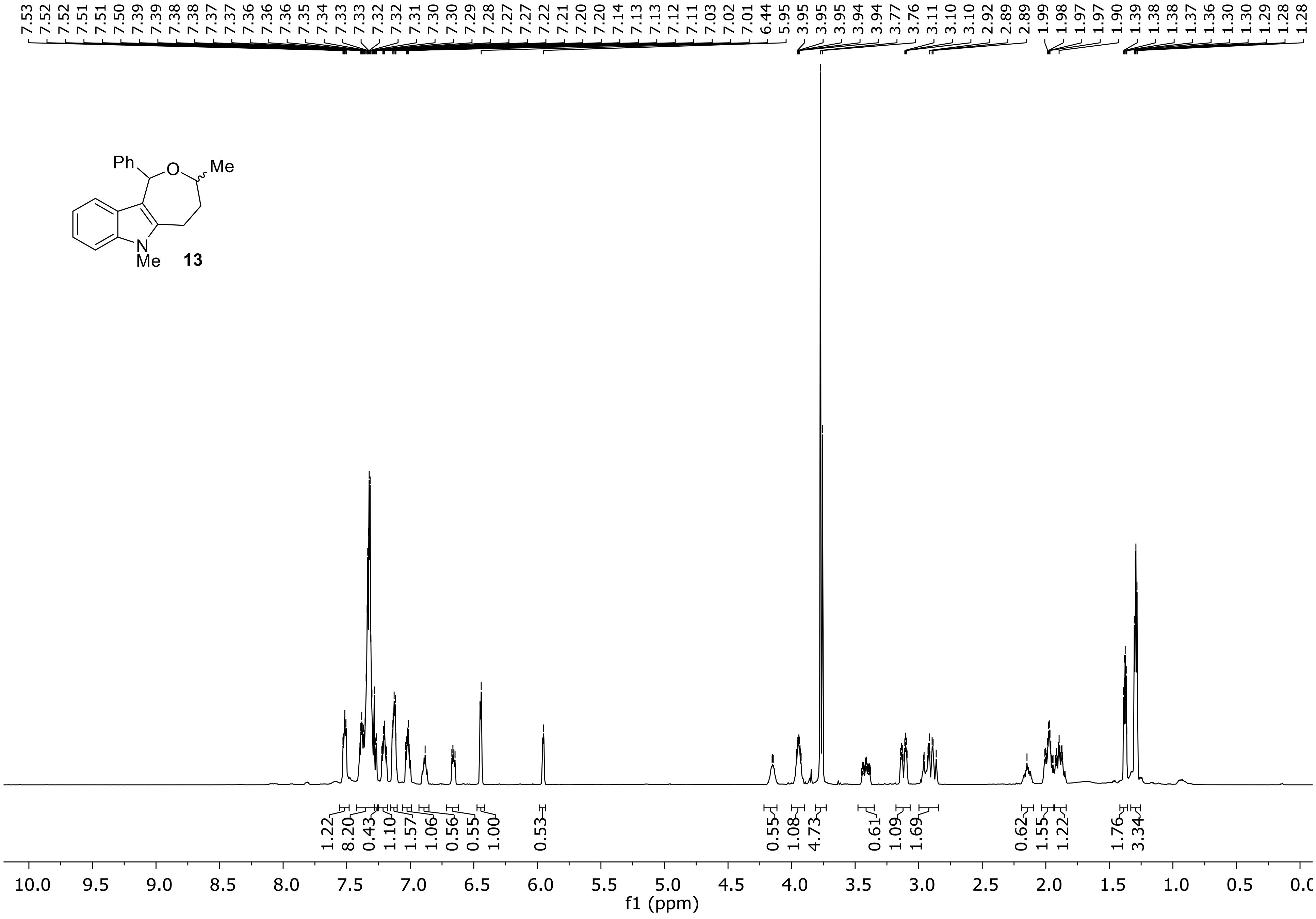

$^{13}C\{^{1}H\}$-NMR (500 MHz, $CDCl_3$)

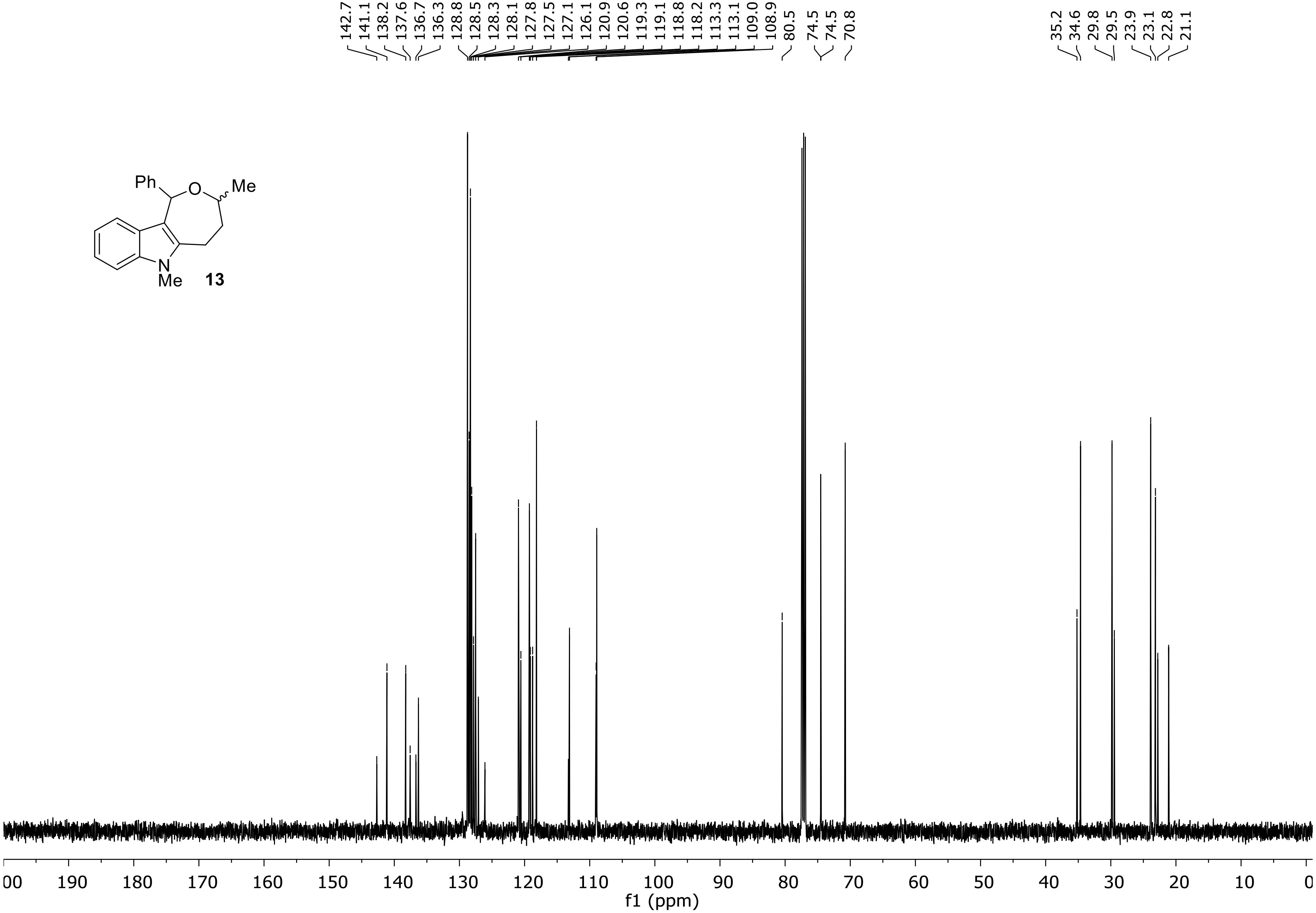

^{1}H-NMR (75.4 MHz, $CDCl_3$)

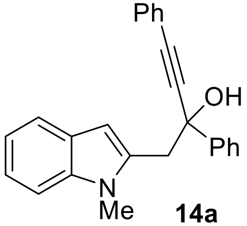

14a

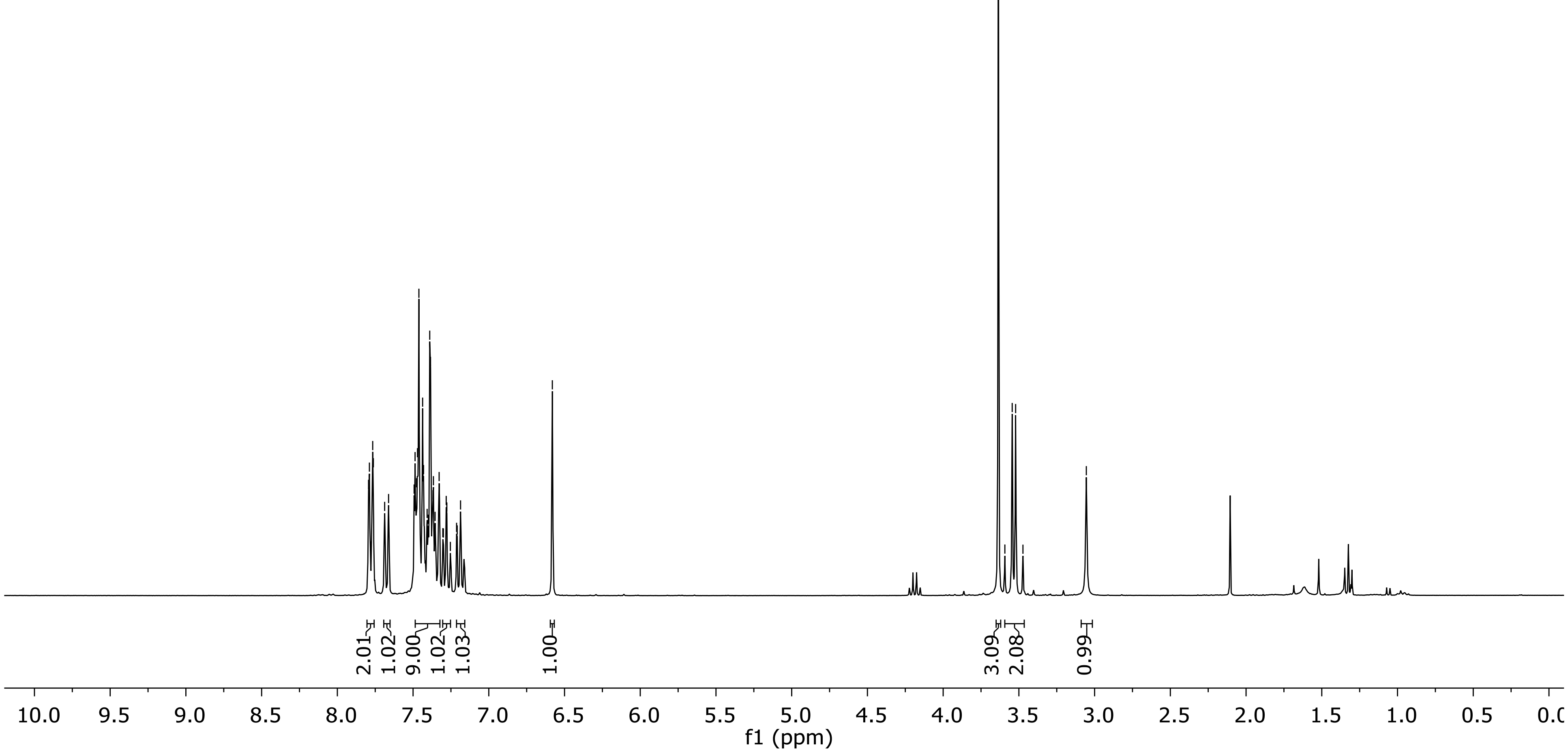

$^{13}C\{^1H\}$-NMR (300 MHz, $CDCl_3$)

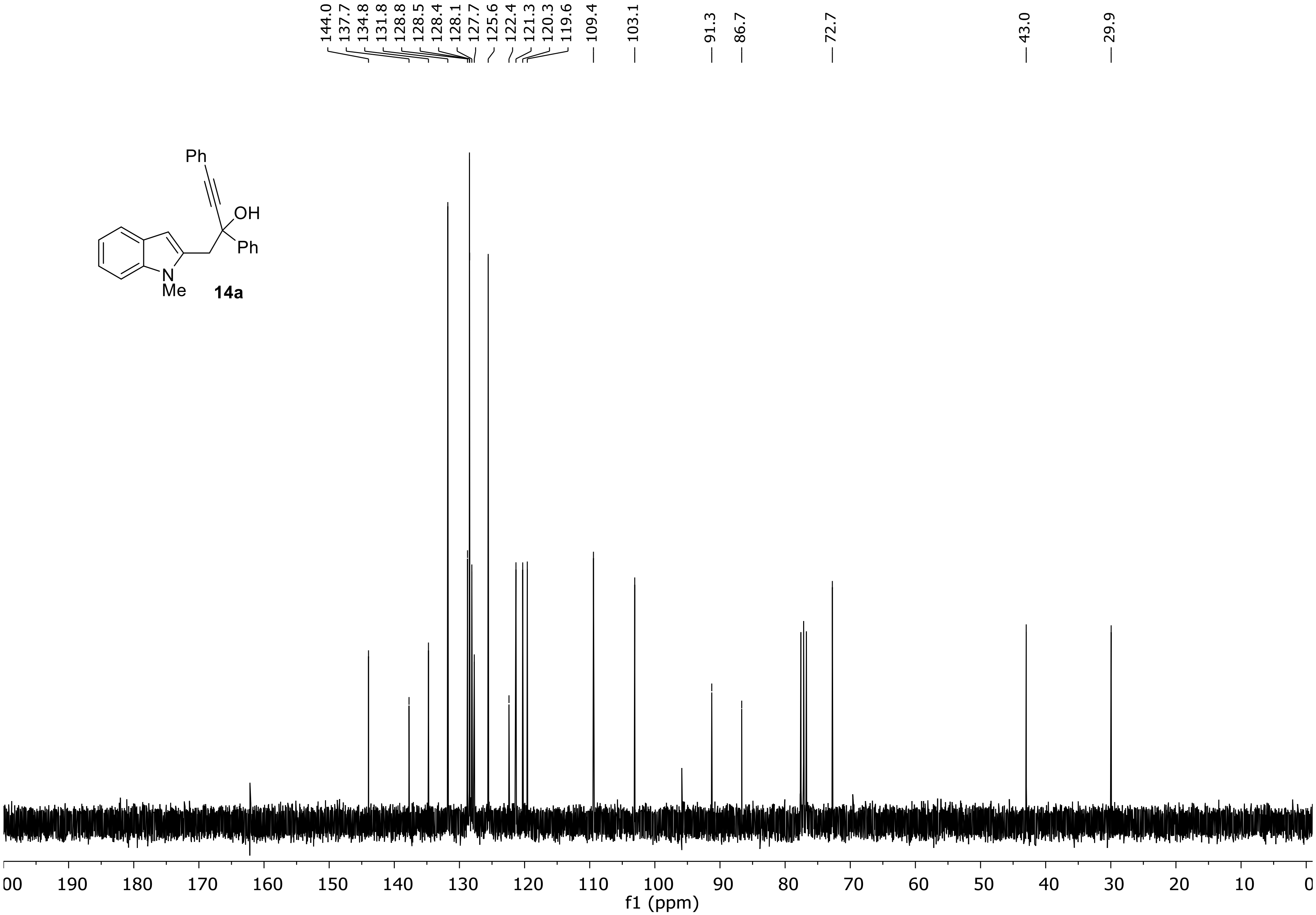

^{1}H-NMR (75.4 MHz, $CDCl_3$)

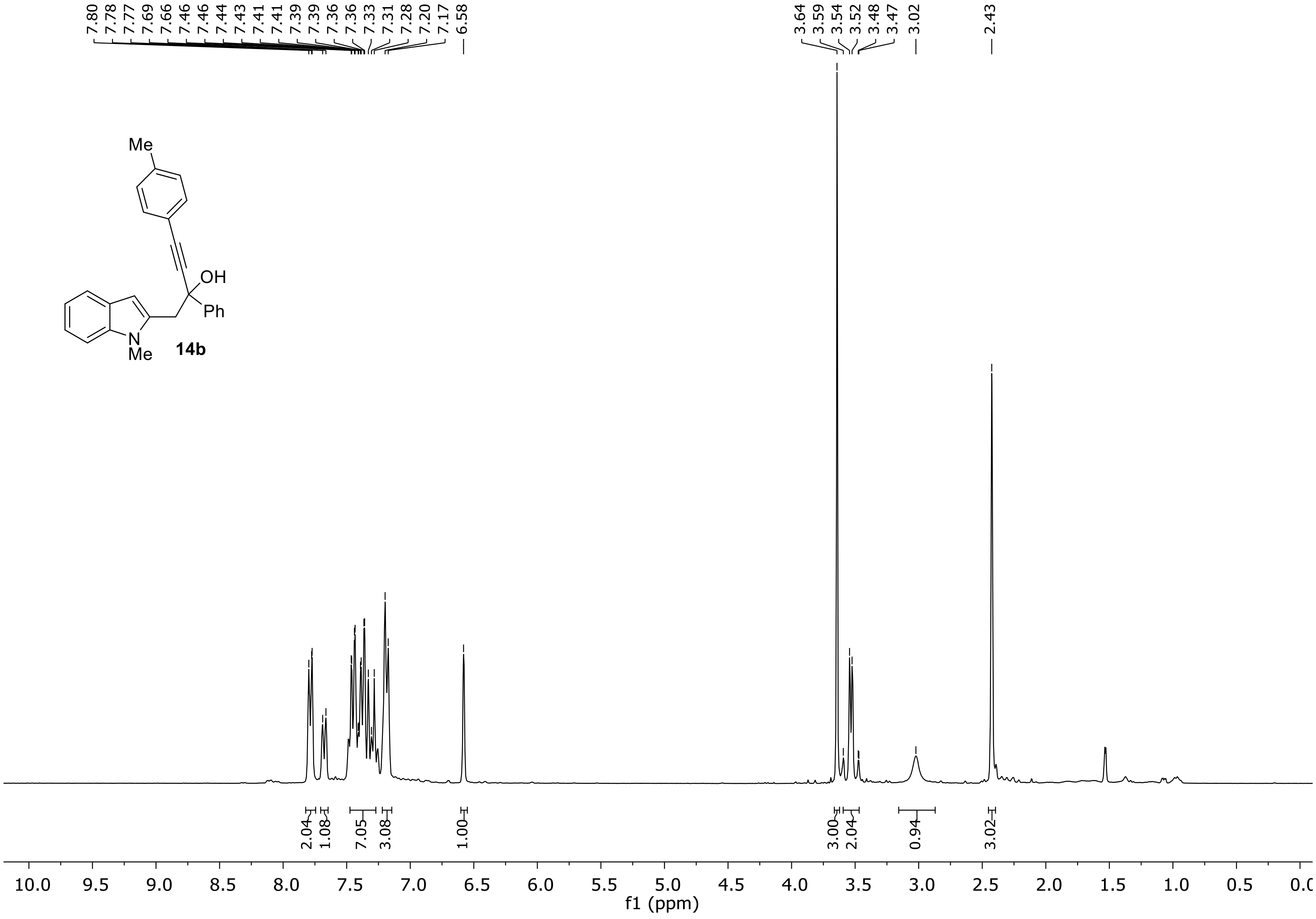

$^{13}C\{^{1}H\}$-NMR (300 MHz, $CDCl_3$)

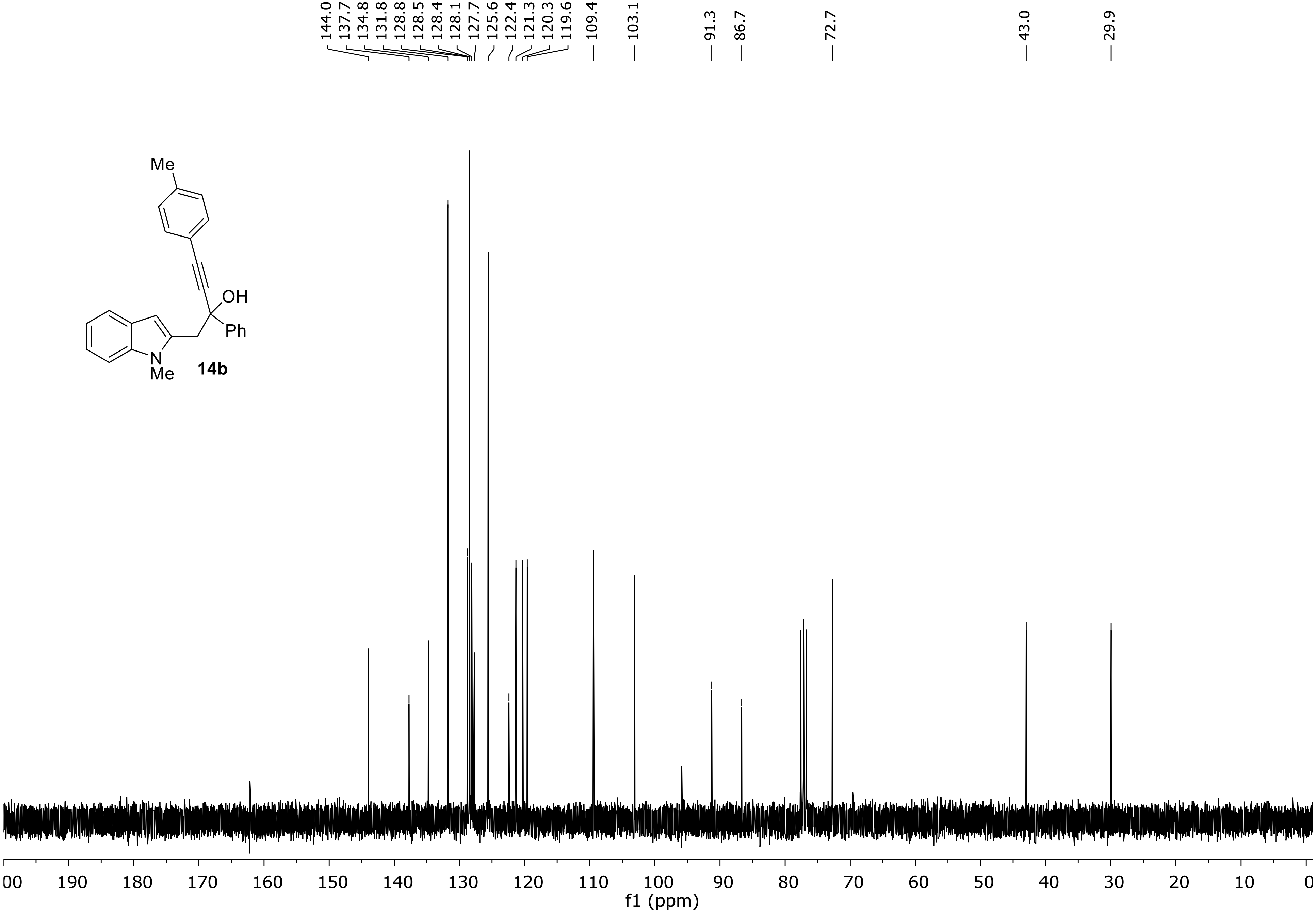

^{1}H-NMR (75.4 MHz, $CDCl_3$)

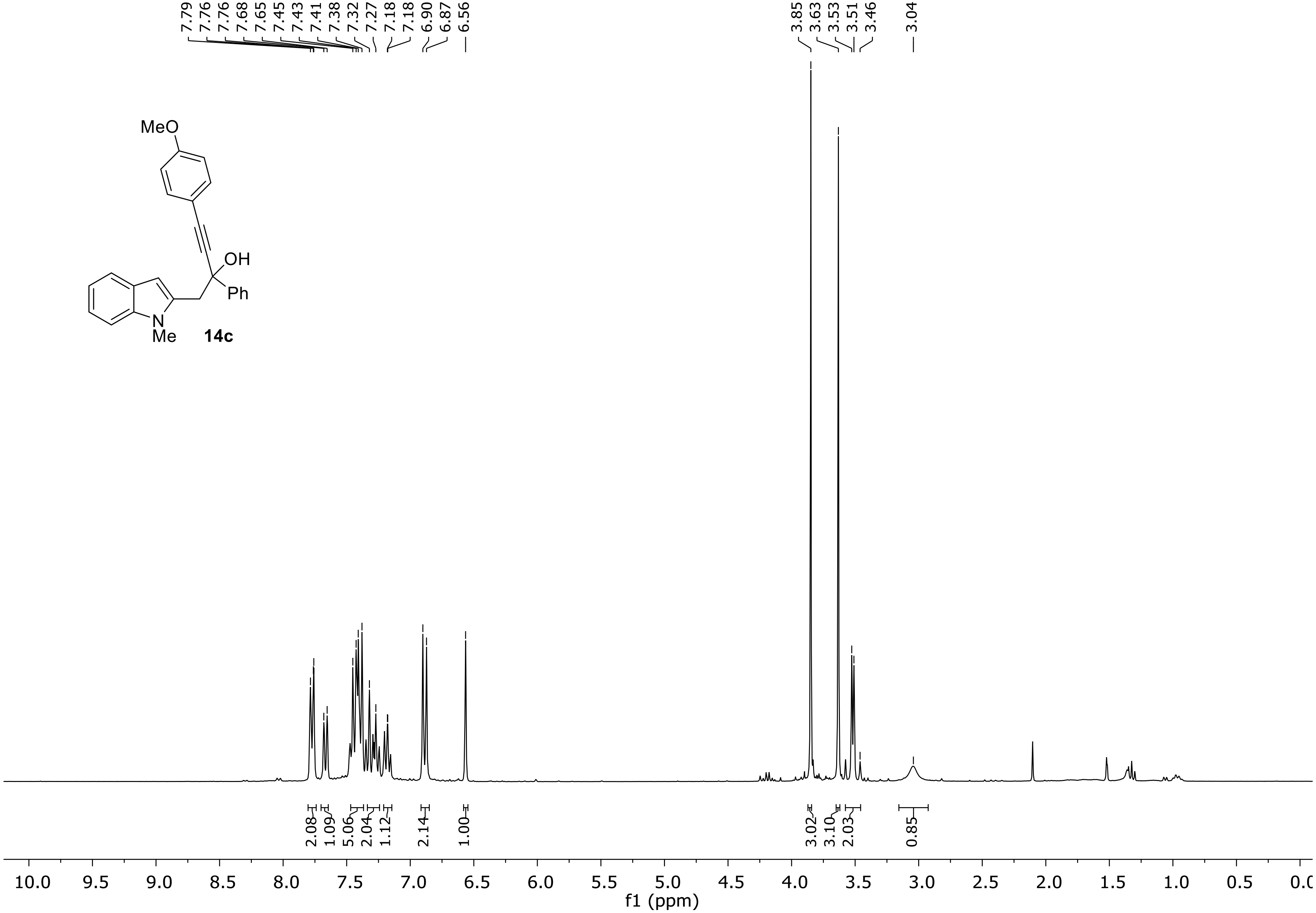

$^{13}C\{^1H\}$-NMR (300 MHz, $CDCl_3$)

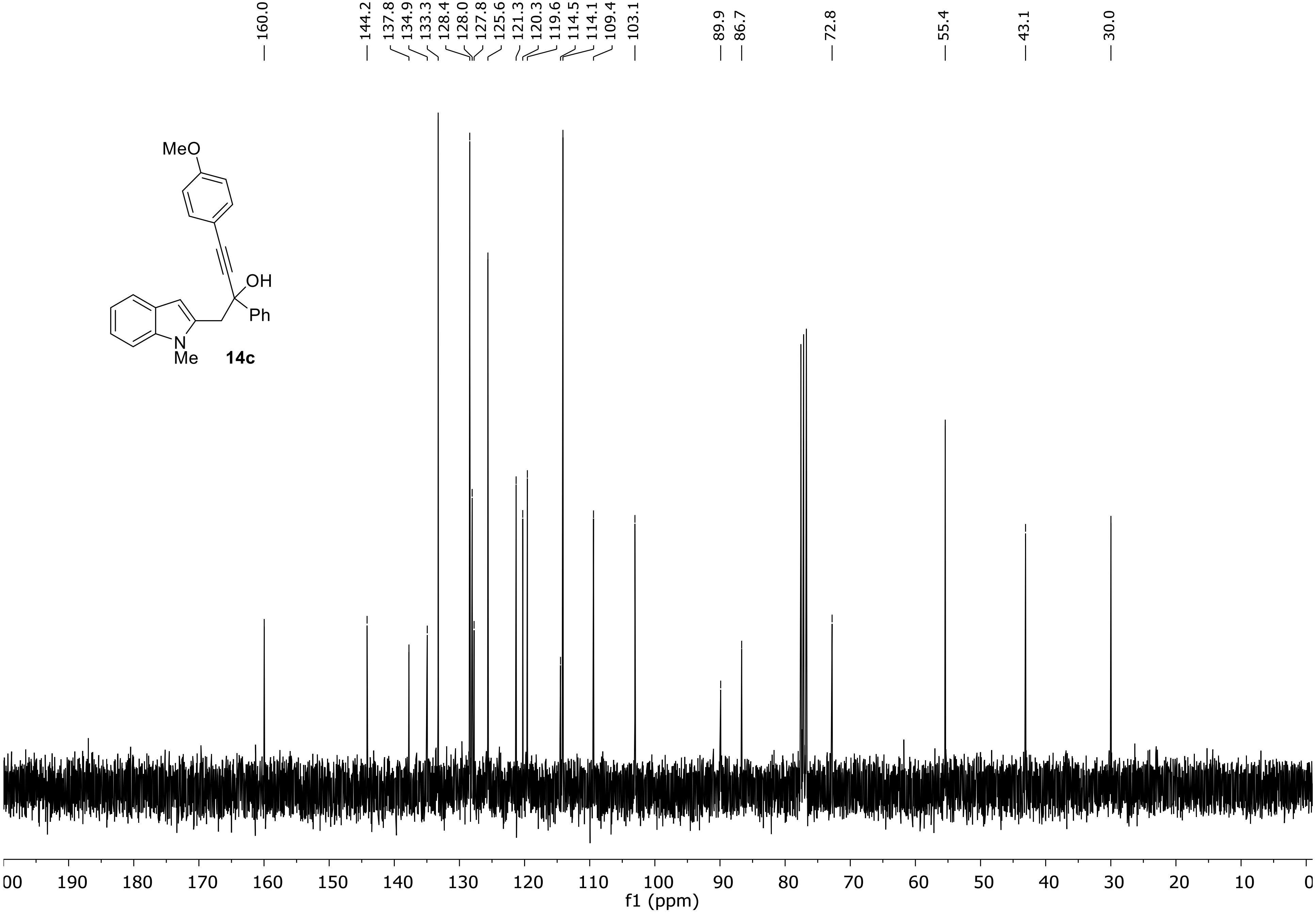

^{1}H-NMR (75.4 MHz, $CDCl_3$)

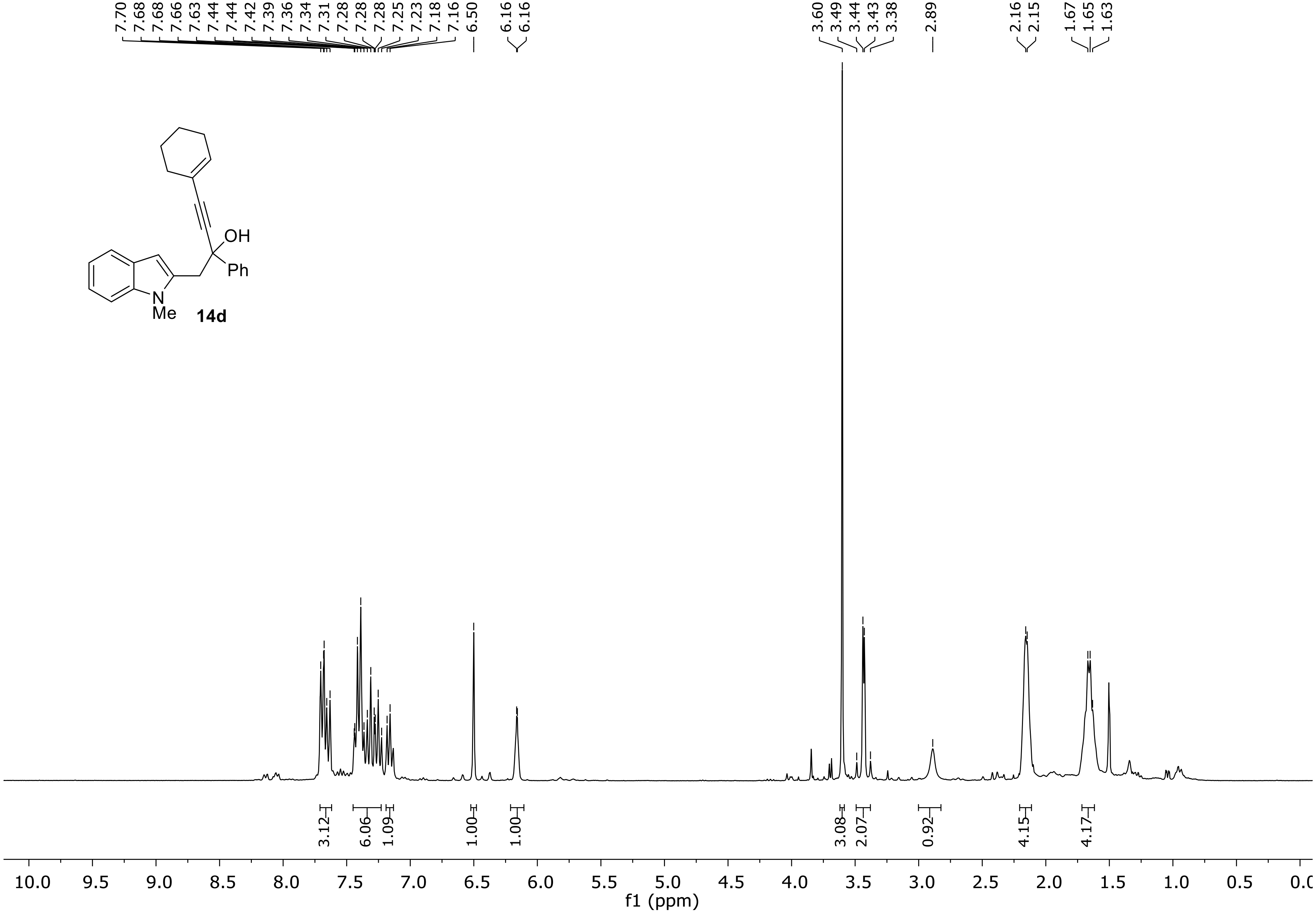

$^{13}C\{^1H\}$-NMR (300 MHz, $CDCl_3$)

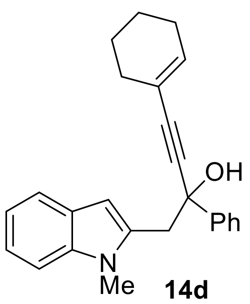

14d

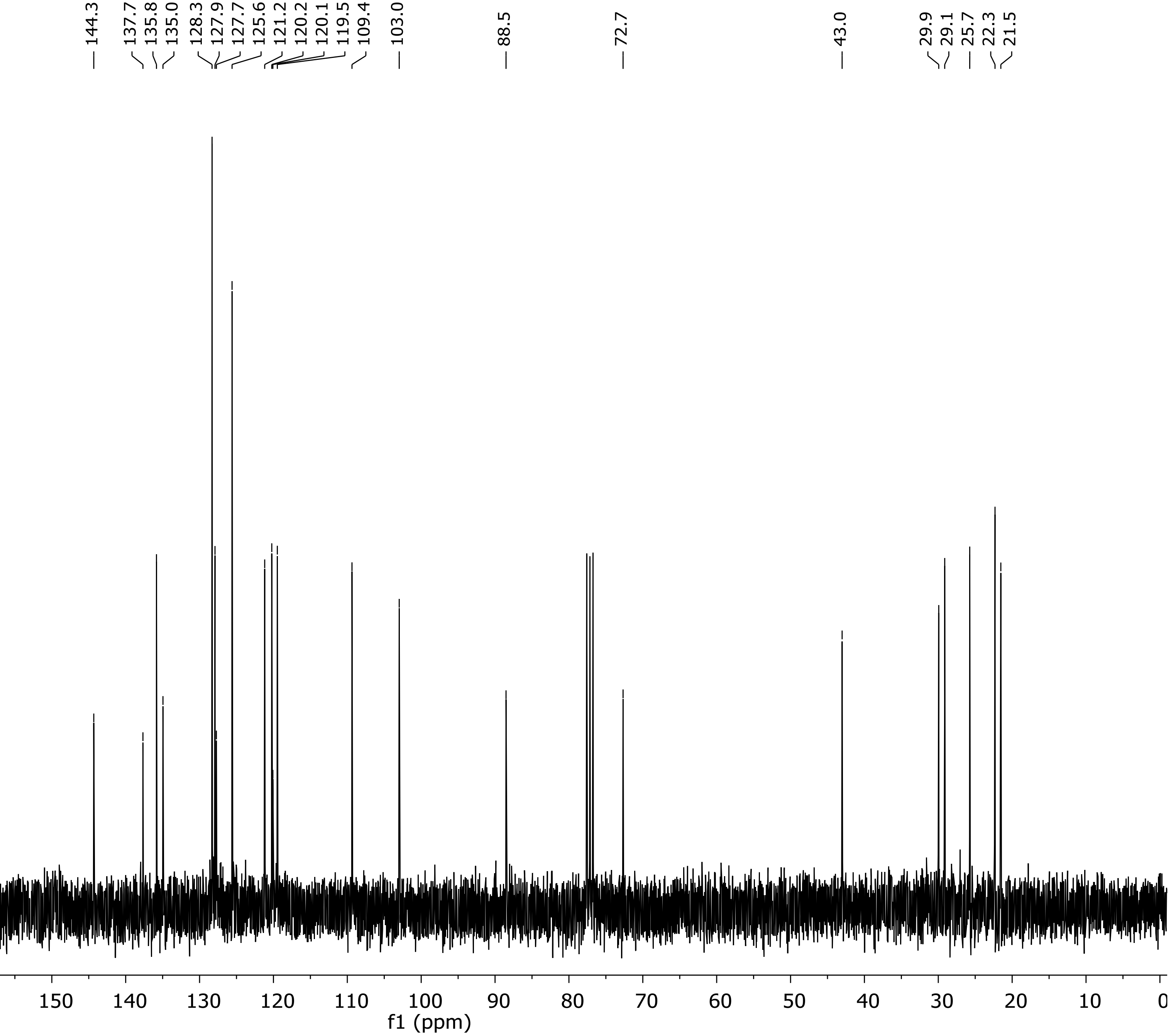

^{1}H-NMR (75.4 MHz, $CDCl_3$)

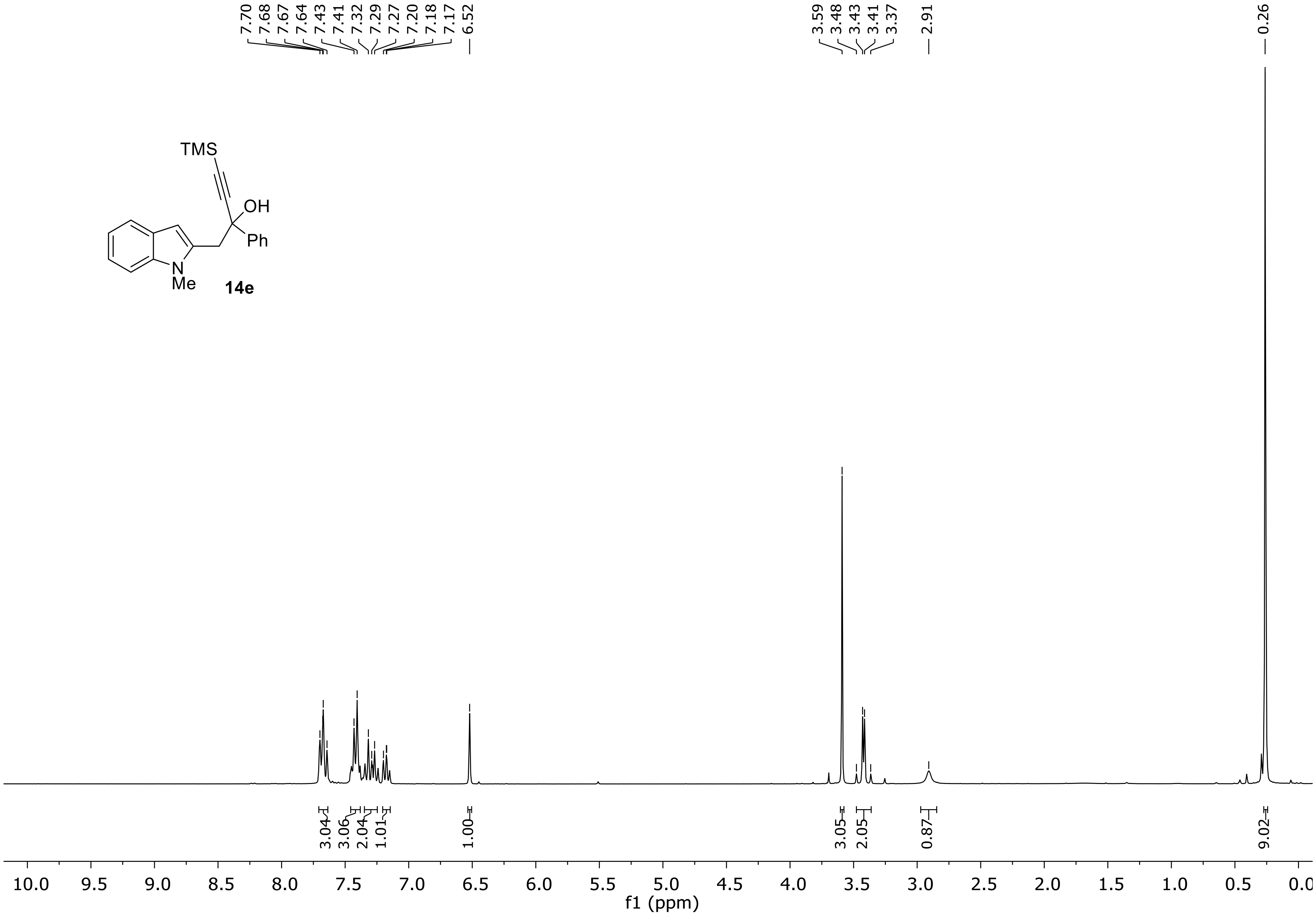

14e

$^{13}C\{^{1}H\}$-NMR (300 MHz, $CDCl_3$)

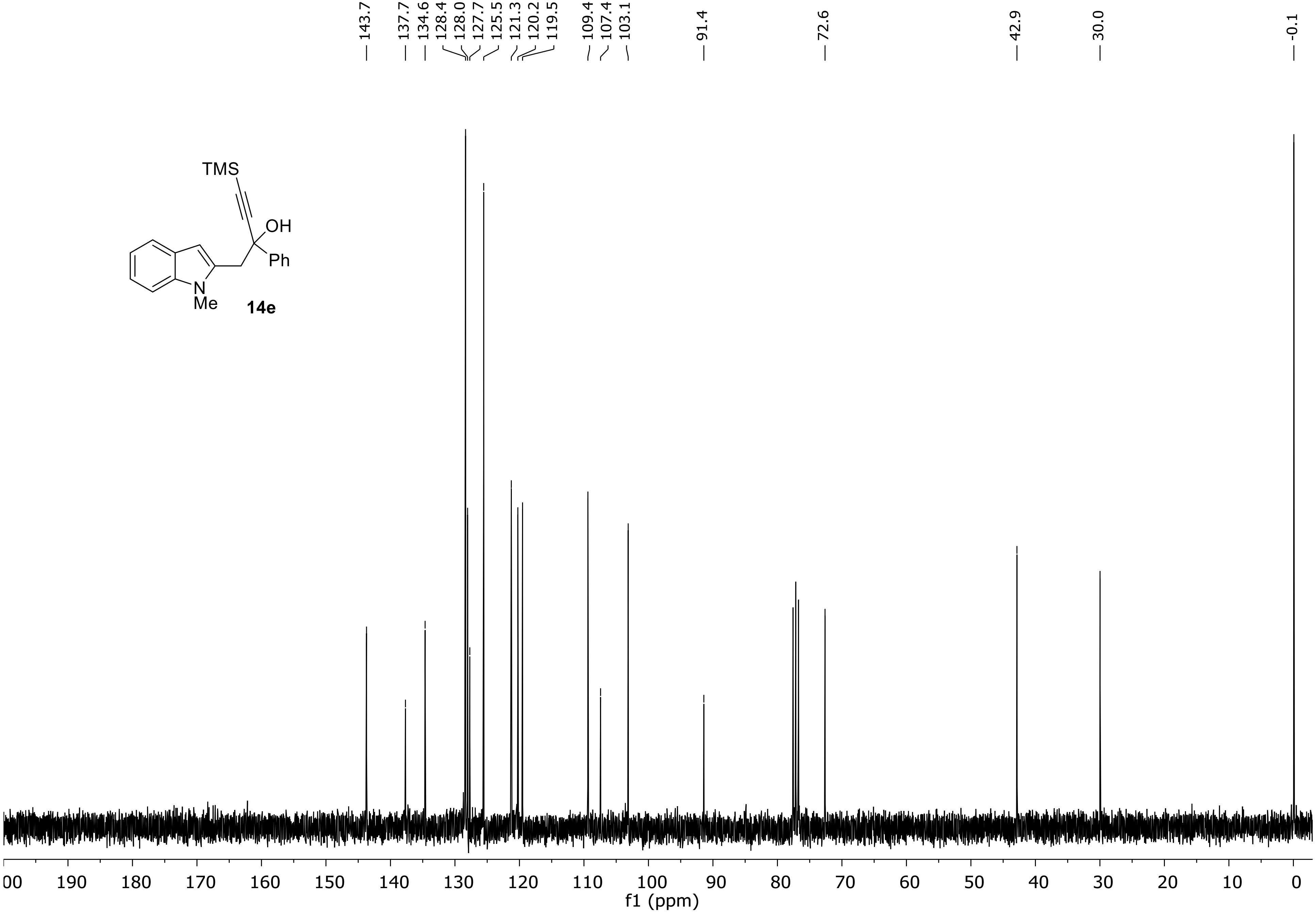

^{1}H-NMR (75.4 MHz, $CDCl_3$)

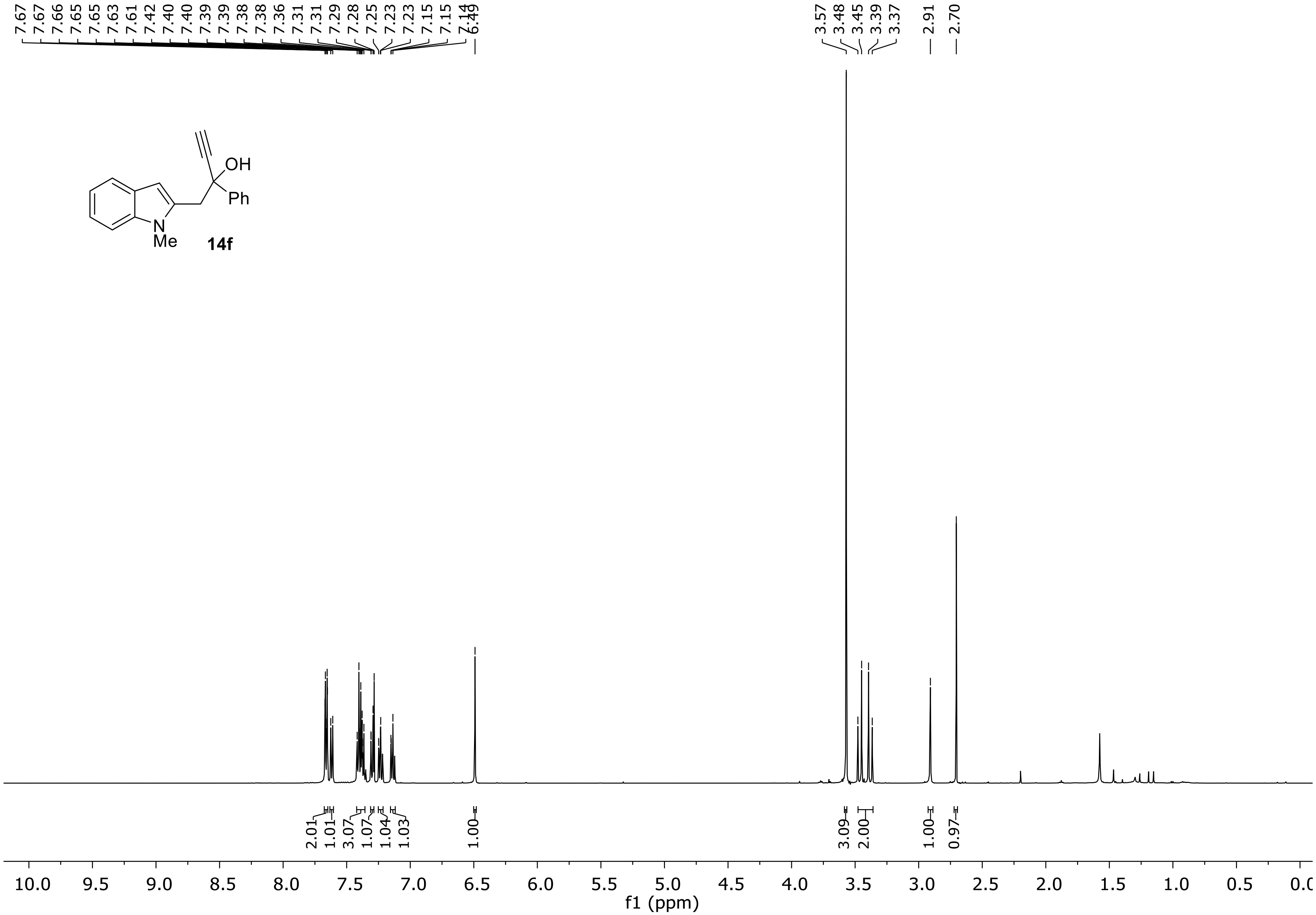

$^{13}C\{^1H\}$-NMR (300 MHz, $CDCl_3$)

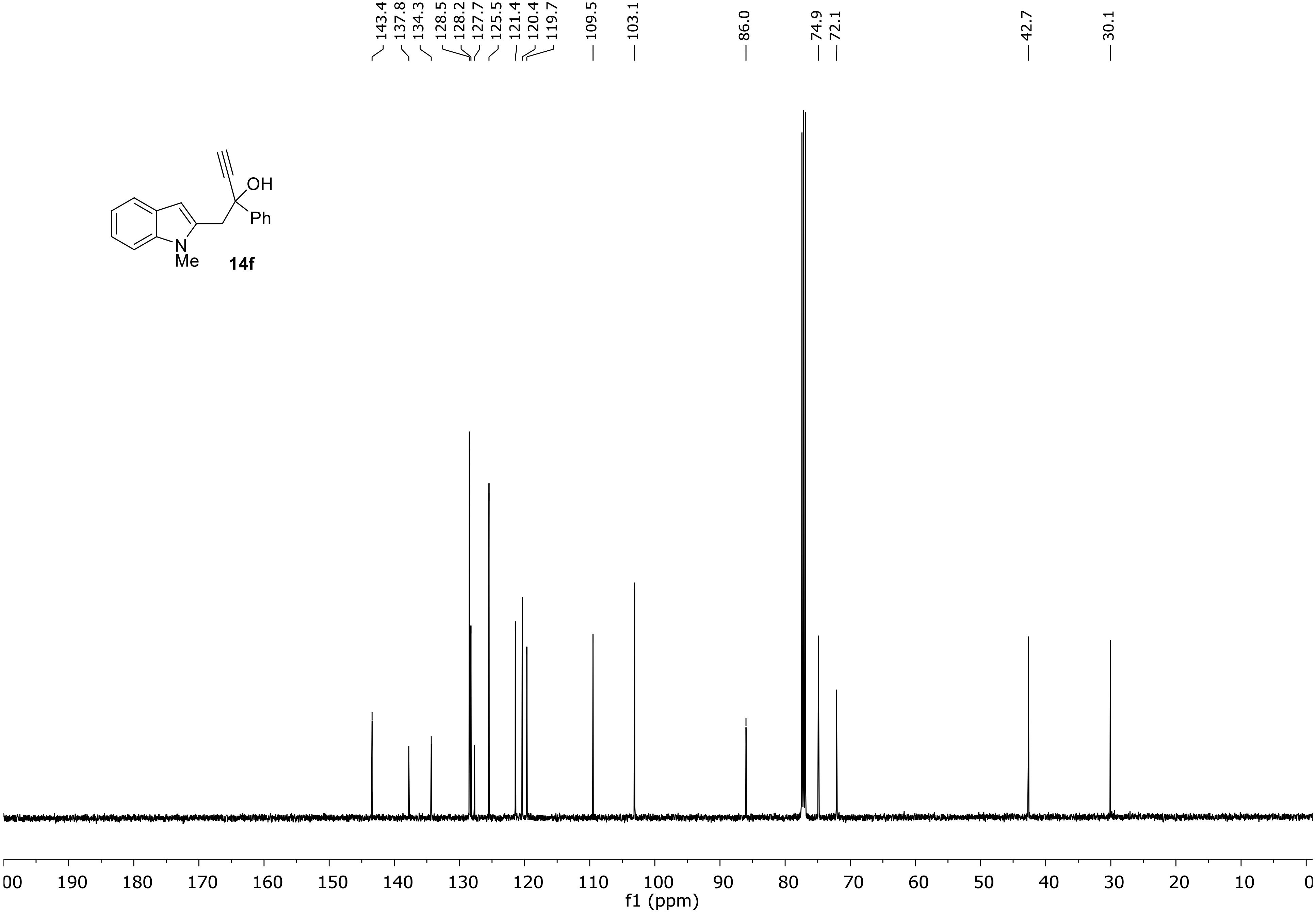

^{1}H-NMR (75.4 MHz, $CDCl_3$)

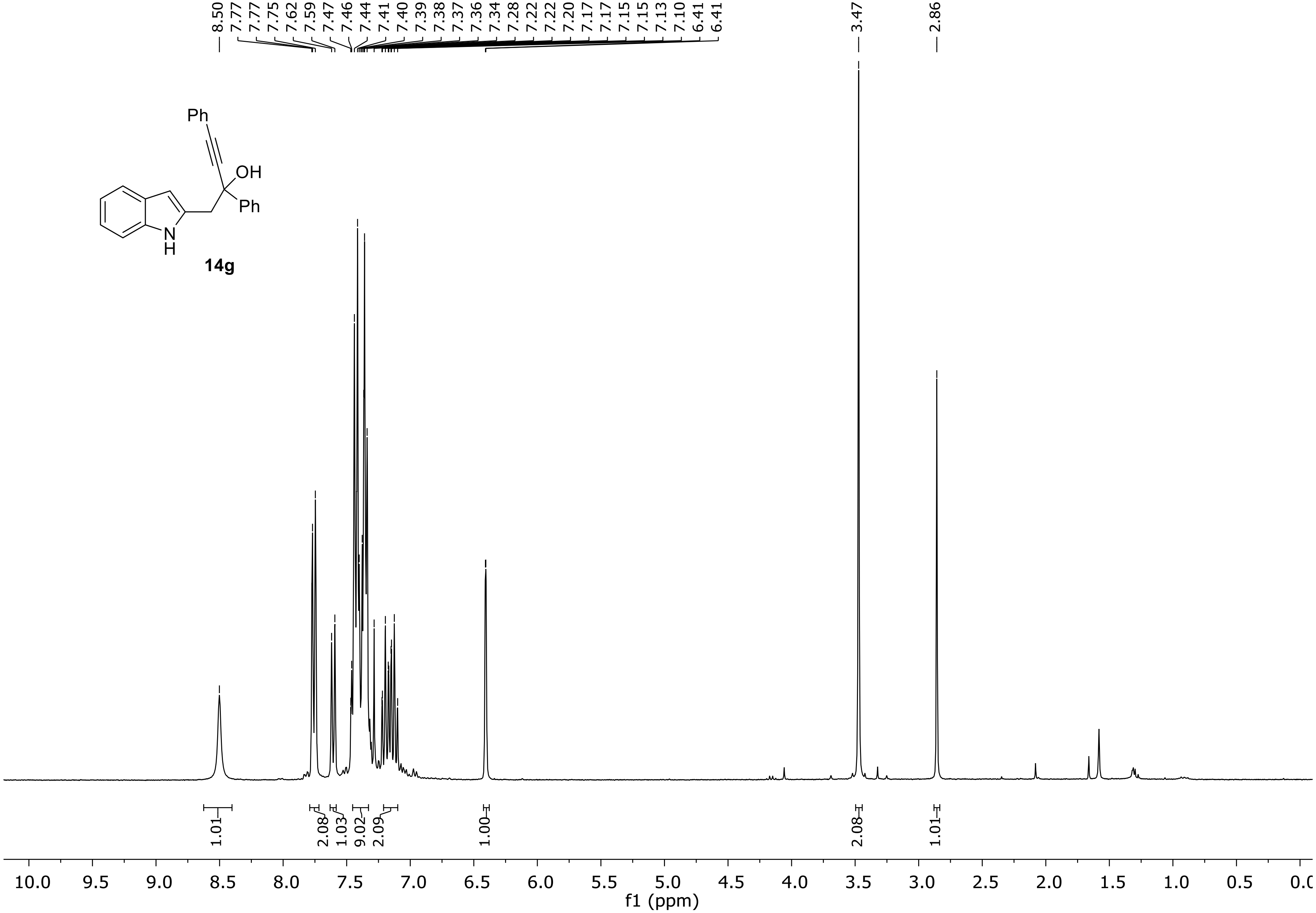

$^{13}C\{^{1}H\}$-NMR (300 MHz, $CDCl_3$)

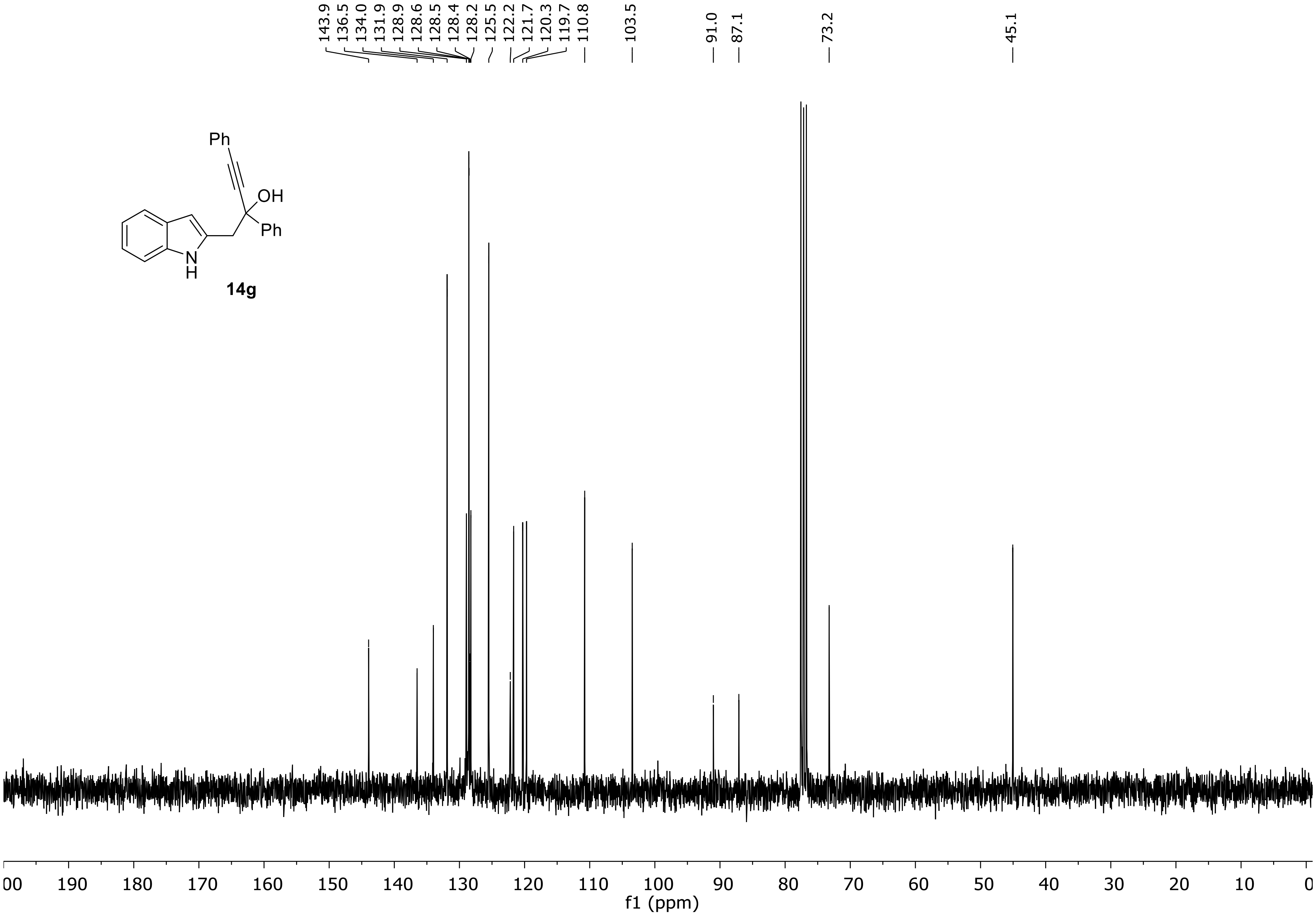

^{1}H-NMR (75.4 MHz, $CDCl_3$)

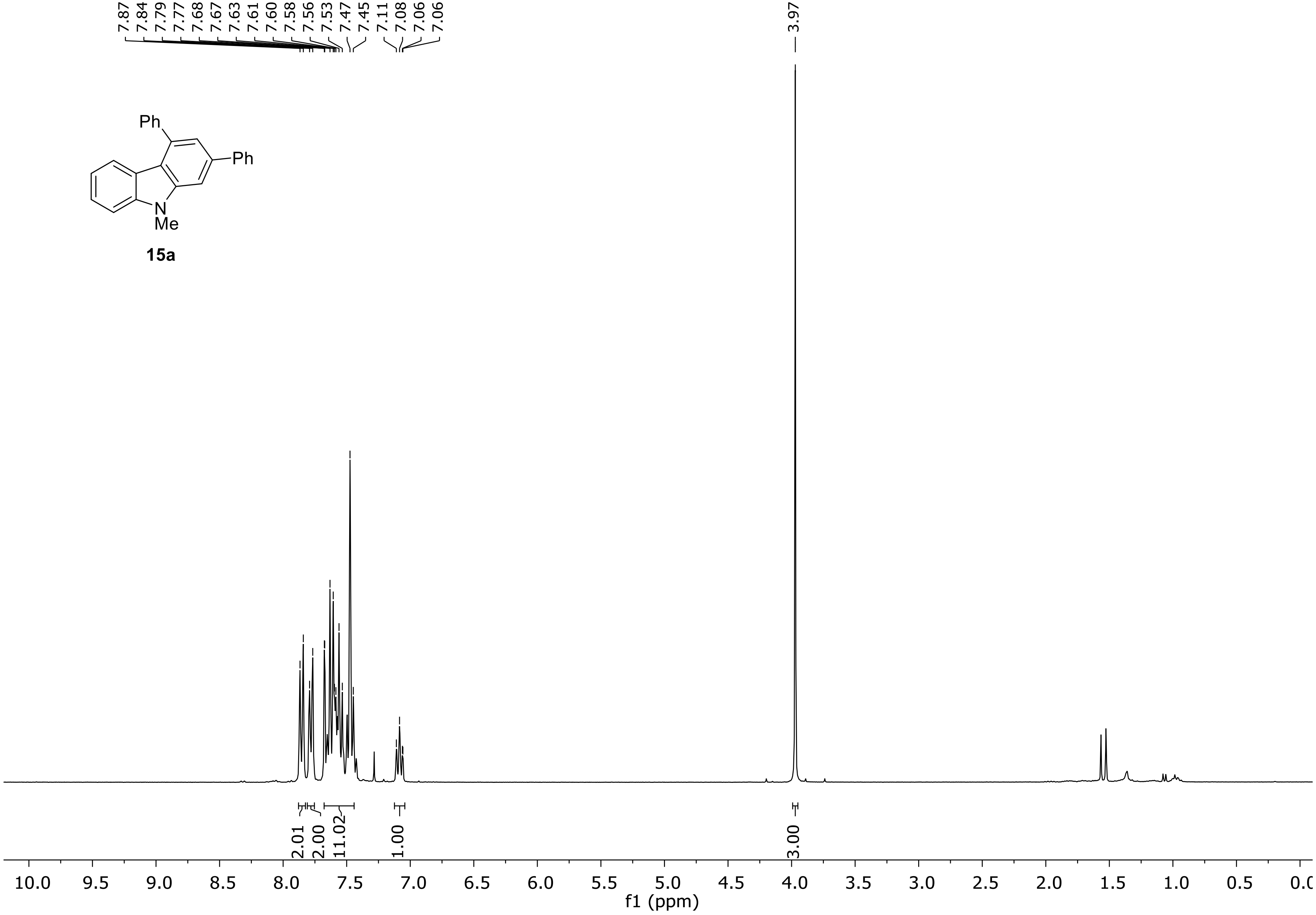

15a

$^{13}C\{^{1}H\}$-NMR (300 MHz, $CDCl_3$)

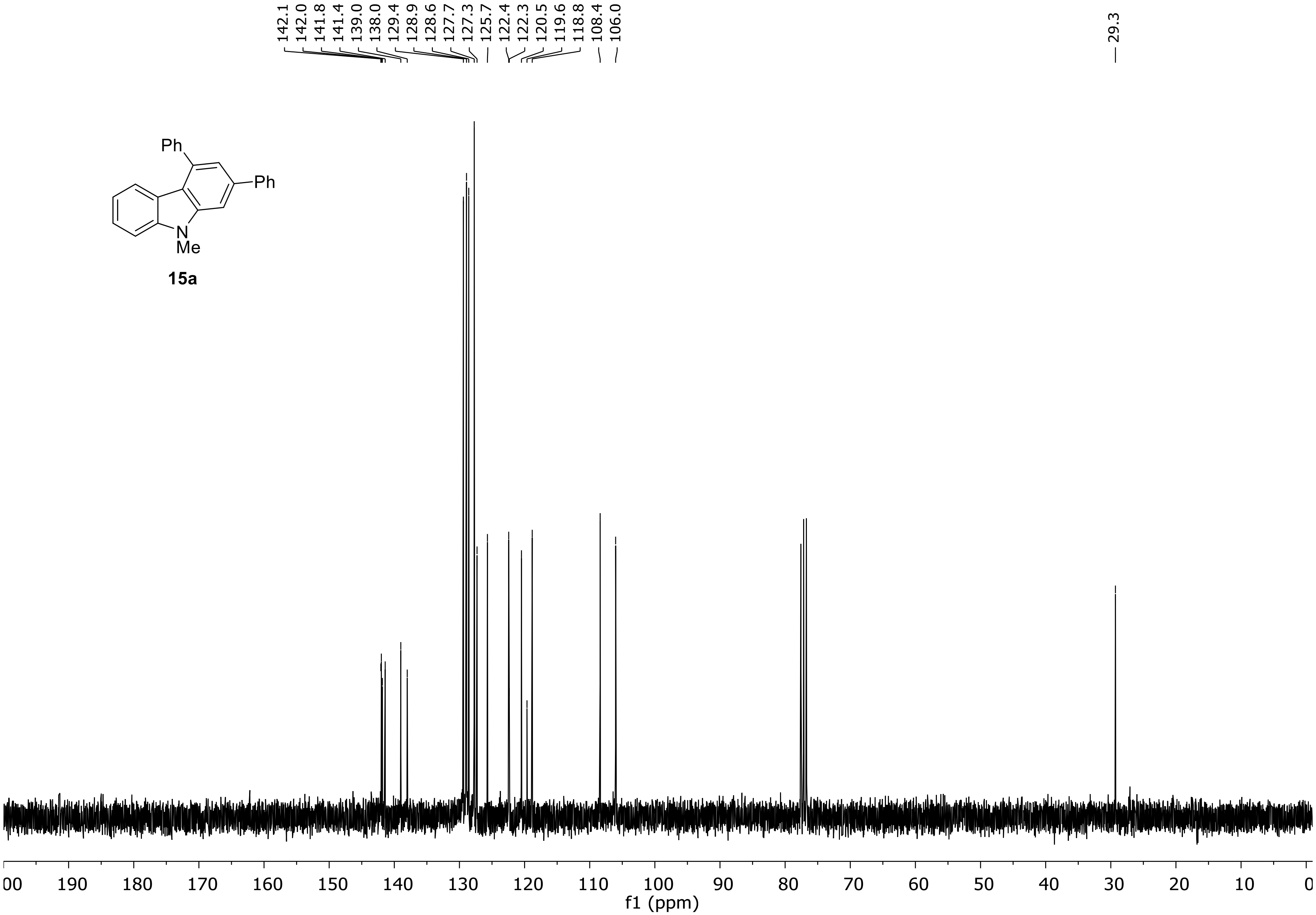

^{1}H-NMR (126 MHz, $CDCl_3$)

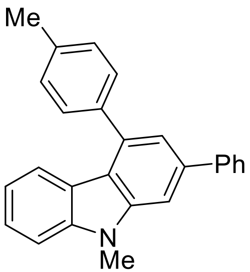

15b

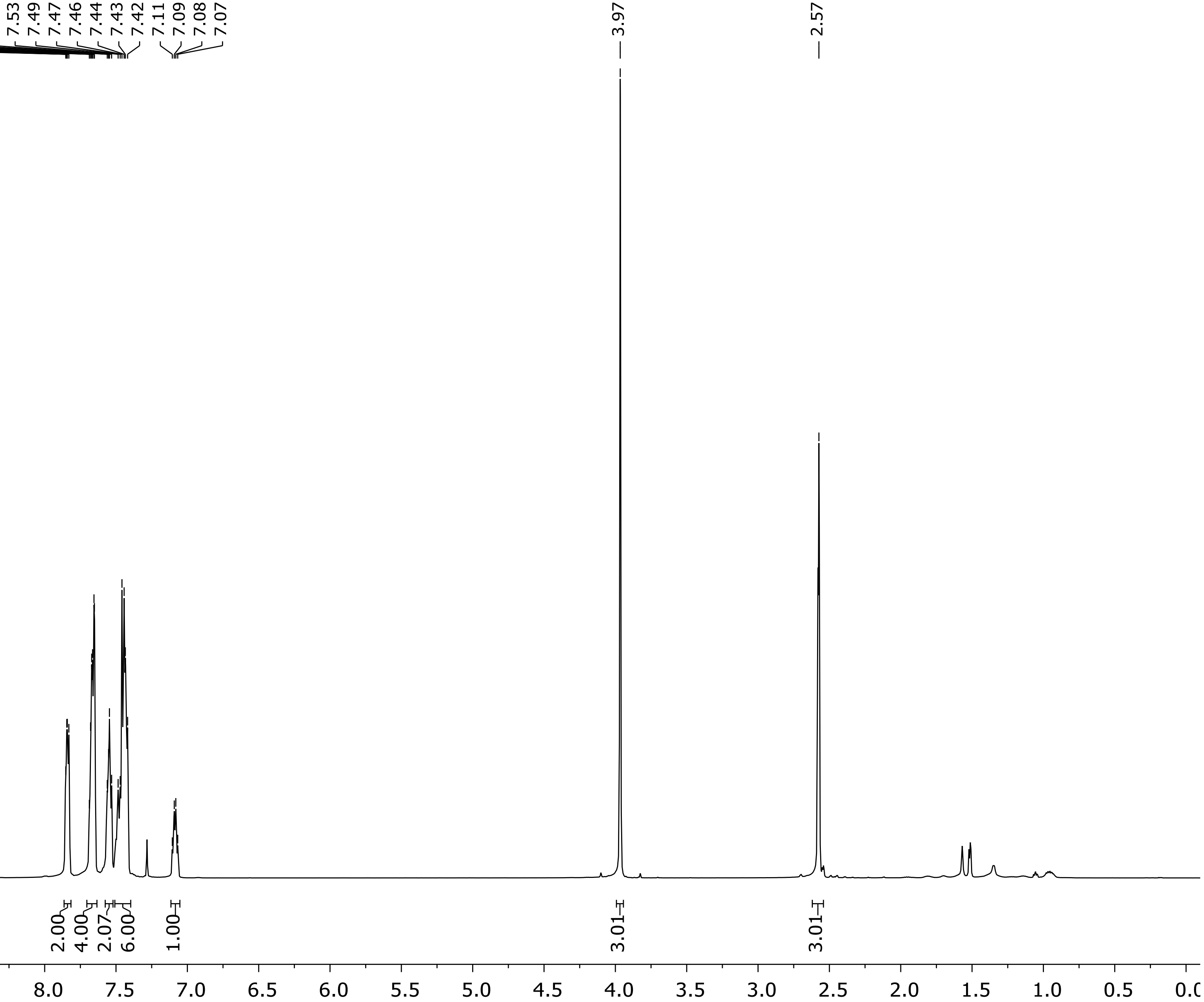

$^{13}C\{^{1}H\}$-NMR (500 MHz, $CDCl_3$)

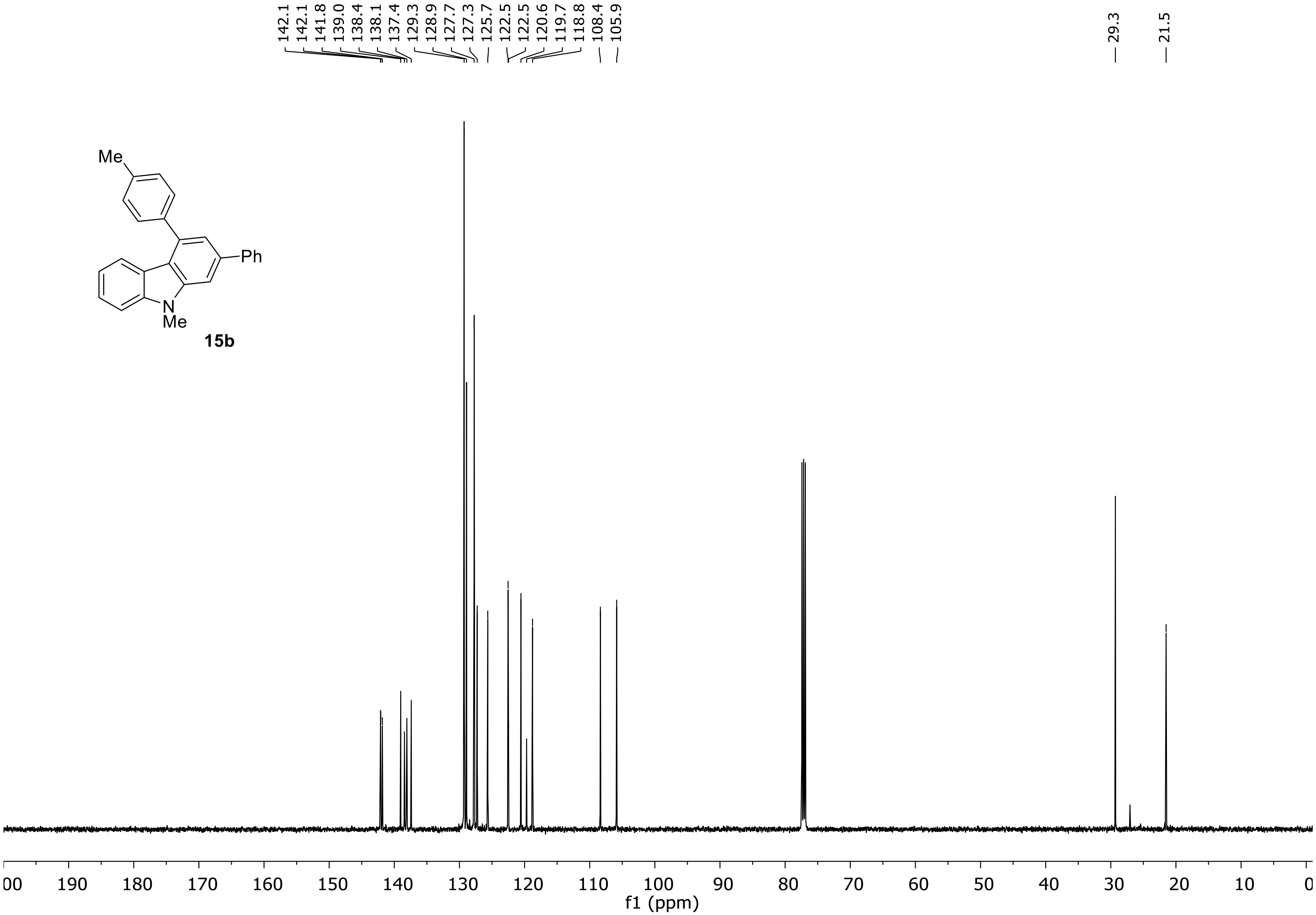

^{1}H-NMR (126 MHz, $CDCl_3$)

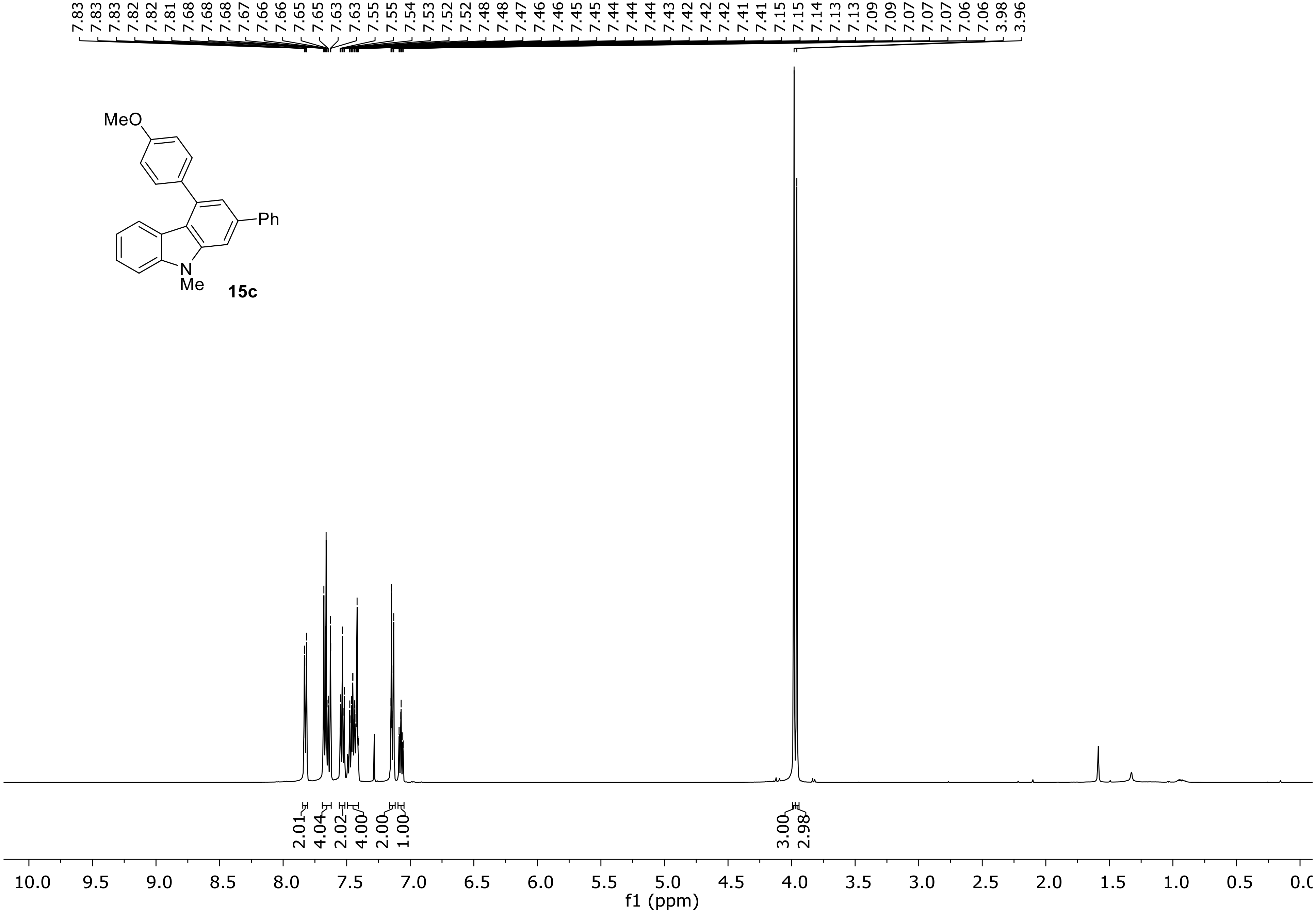

$^{13}C\{^1H\}$-NMR (500 MHz, $CDCl_3$)

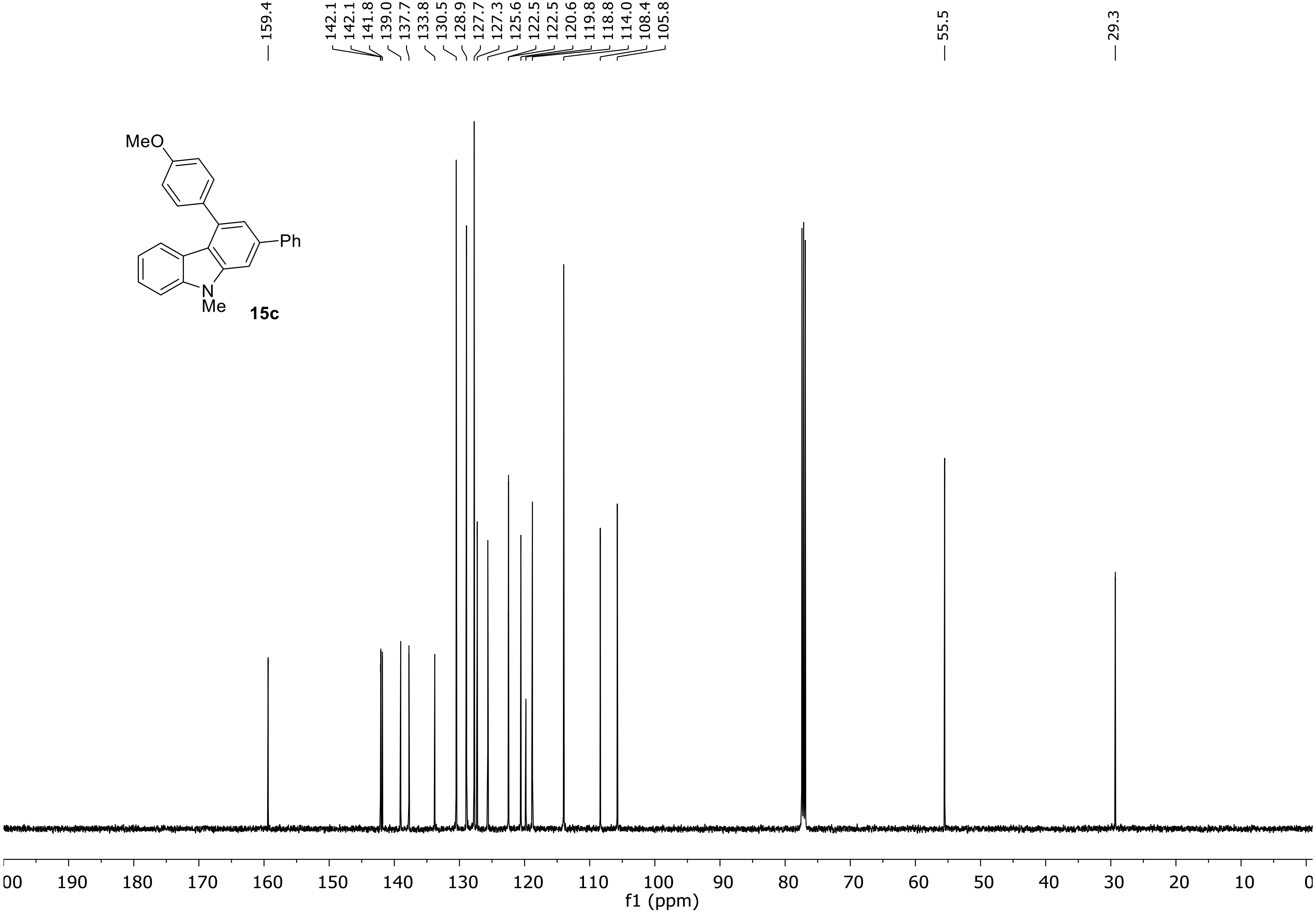

15c

^{1}H-NMR (126 MHz, $CDCl_3$)

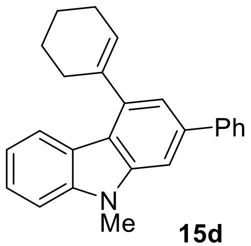

15d

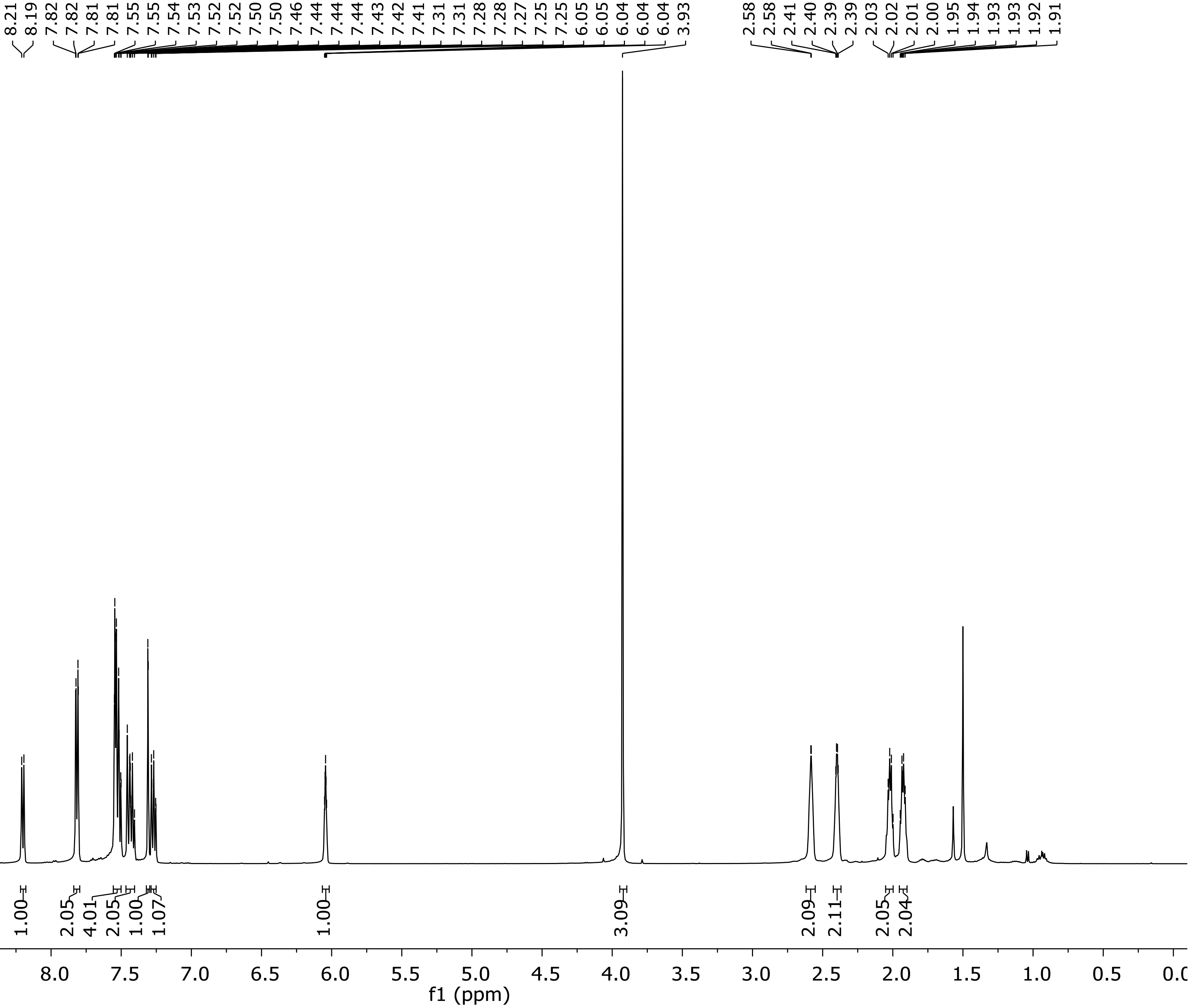

$^{13}C\{^1H\}$-NMR (500 MHz, $CDCl_3$)

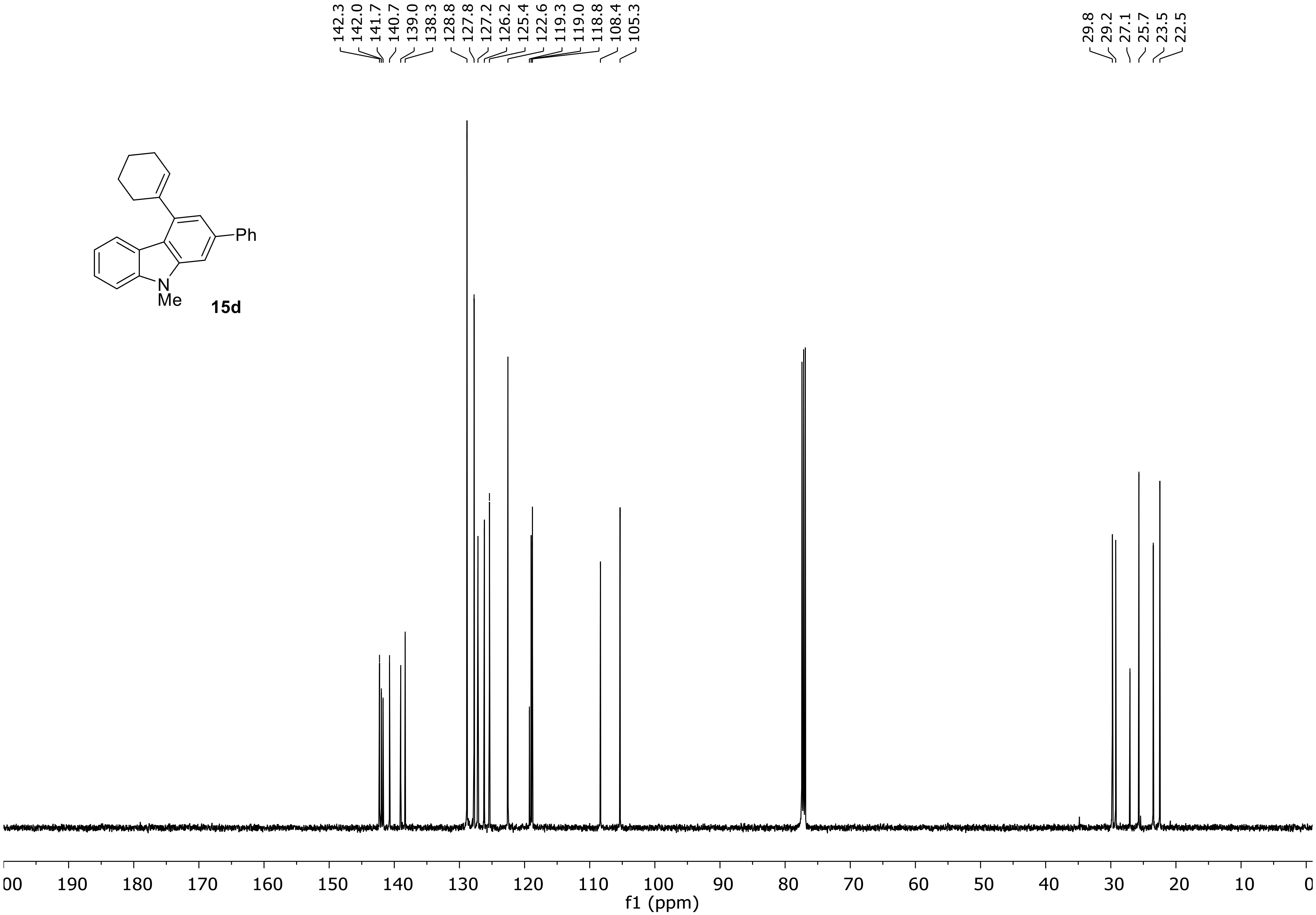

^{1}H-NMR (126 MHz, $CDCl_3$)

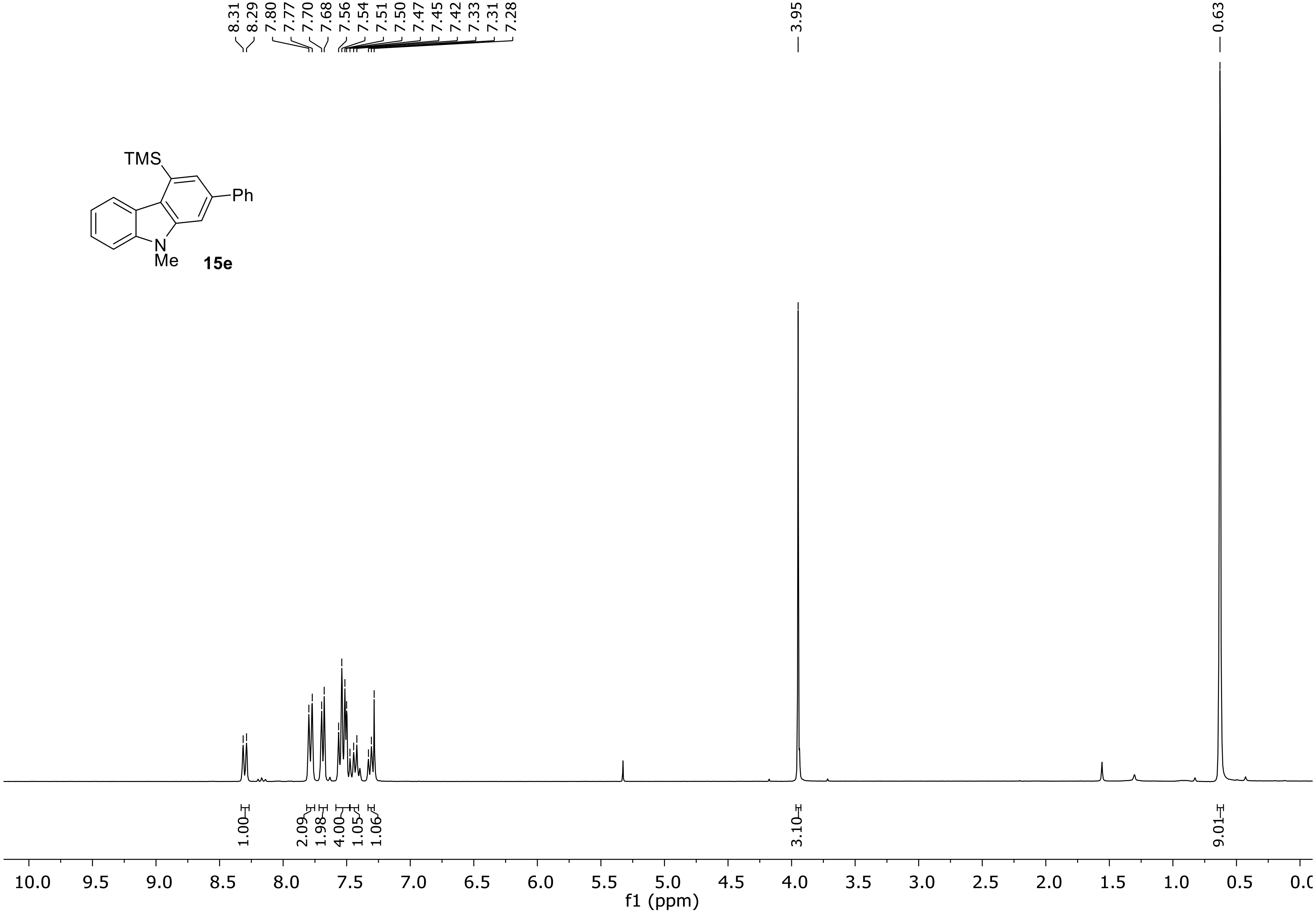

$^{13}C\{^1H\}$-NMR (500 MHz, $CDCl_3$)

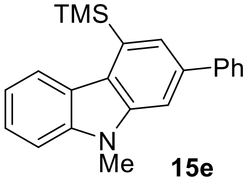

15e

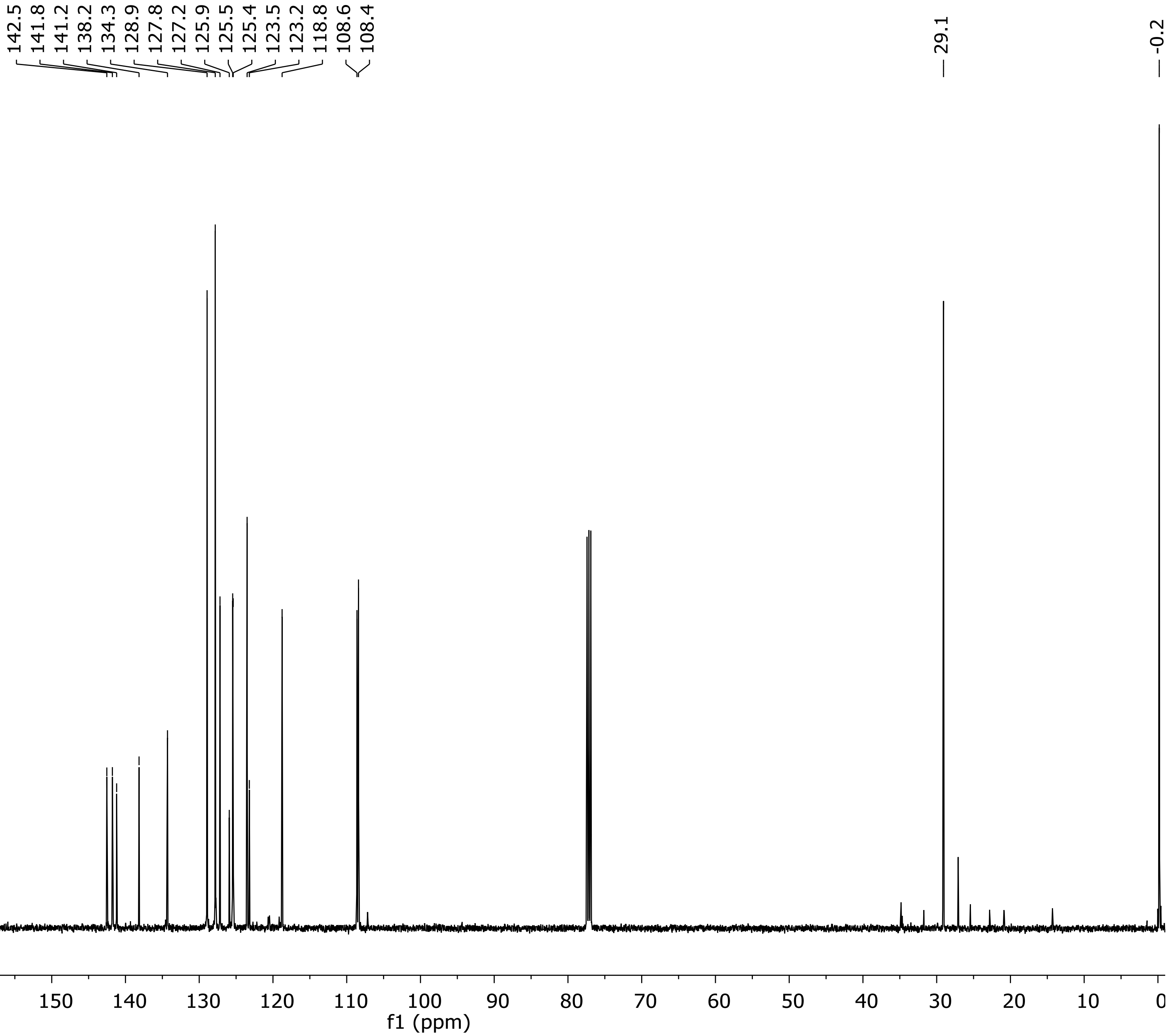

^{1}H-NMR (126 MHz, $CDCl_3$)

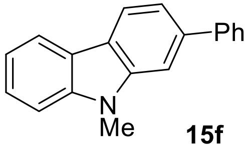

15f

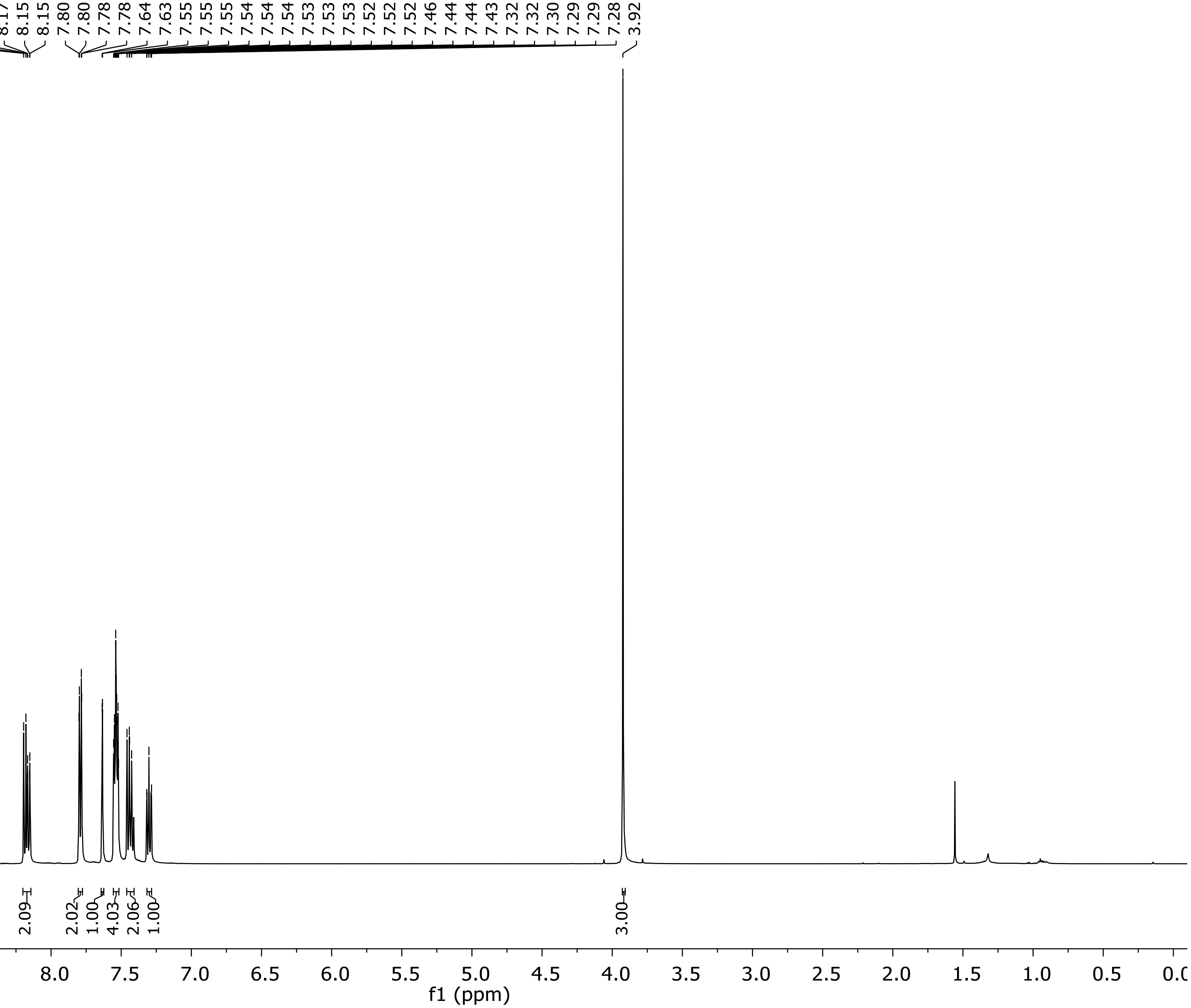

$^{13}C\{^1H\}$-NMR (500 MHz, $CDCl_3$)

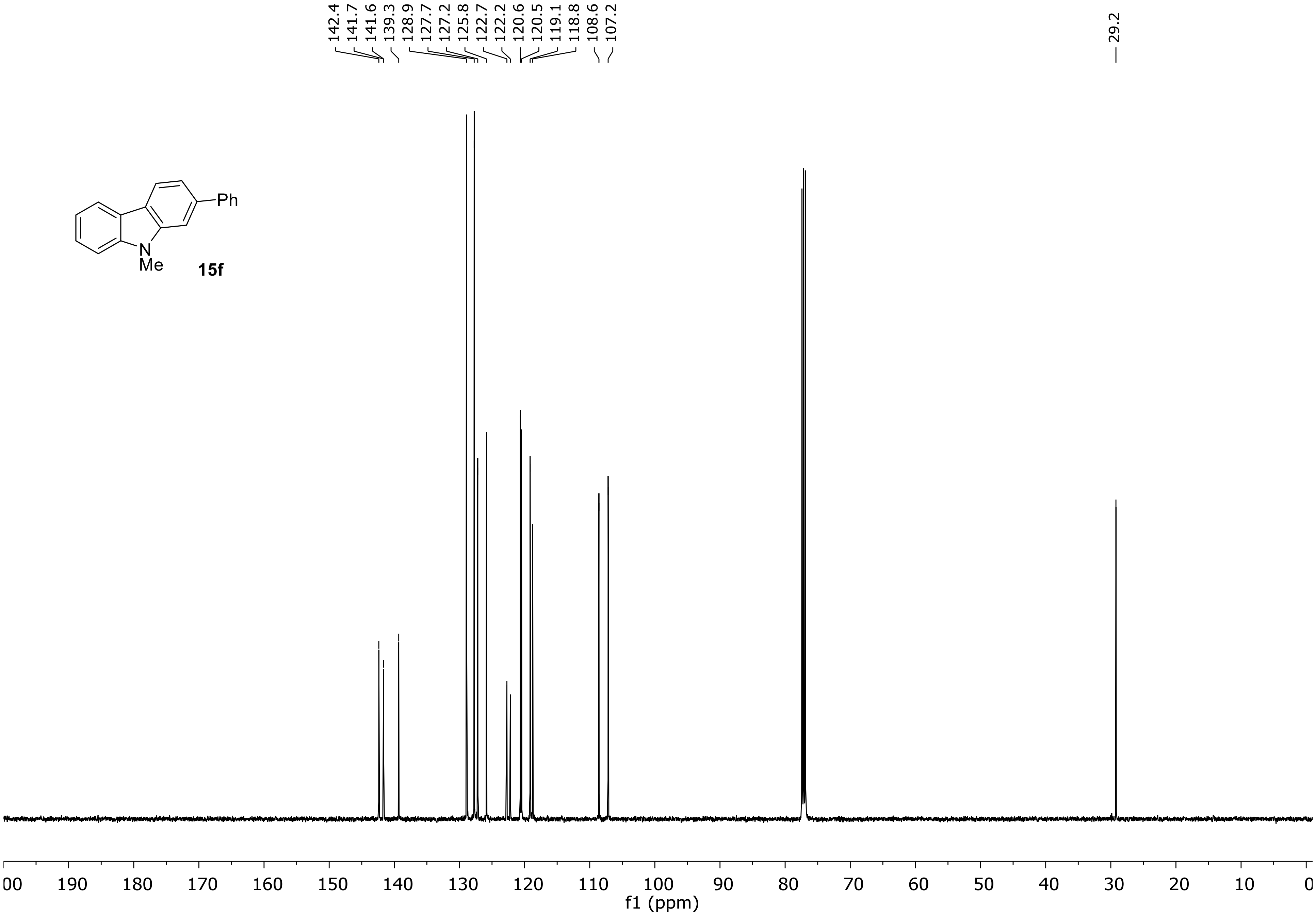

^{1}H-NMR (126 MHz, $CDCl_3$)

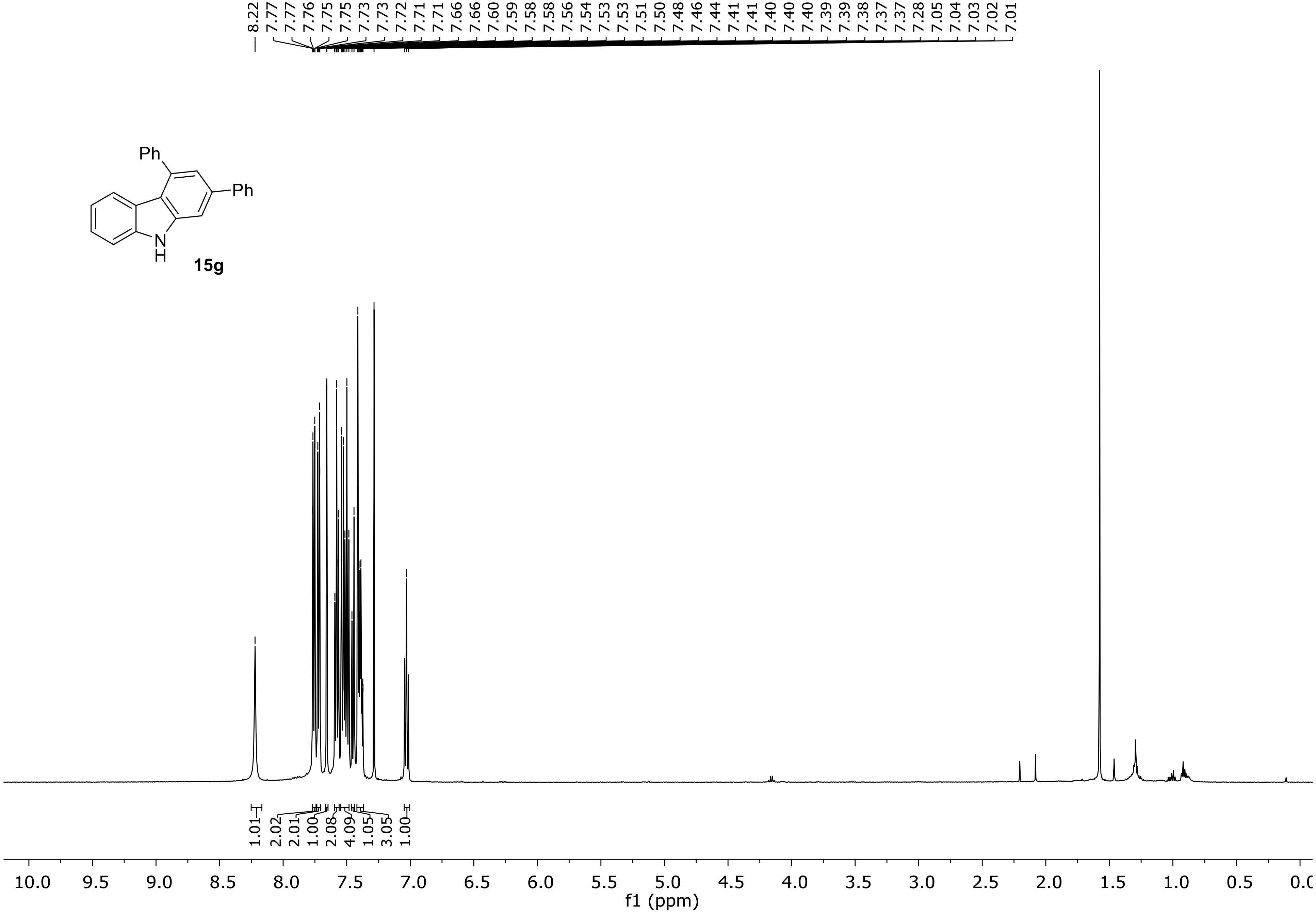

15g

$^{13}C\{^1H\}$-NMR (500 MHz, $CDCl_3$)

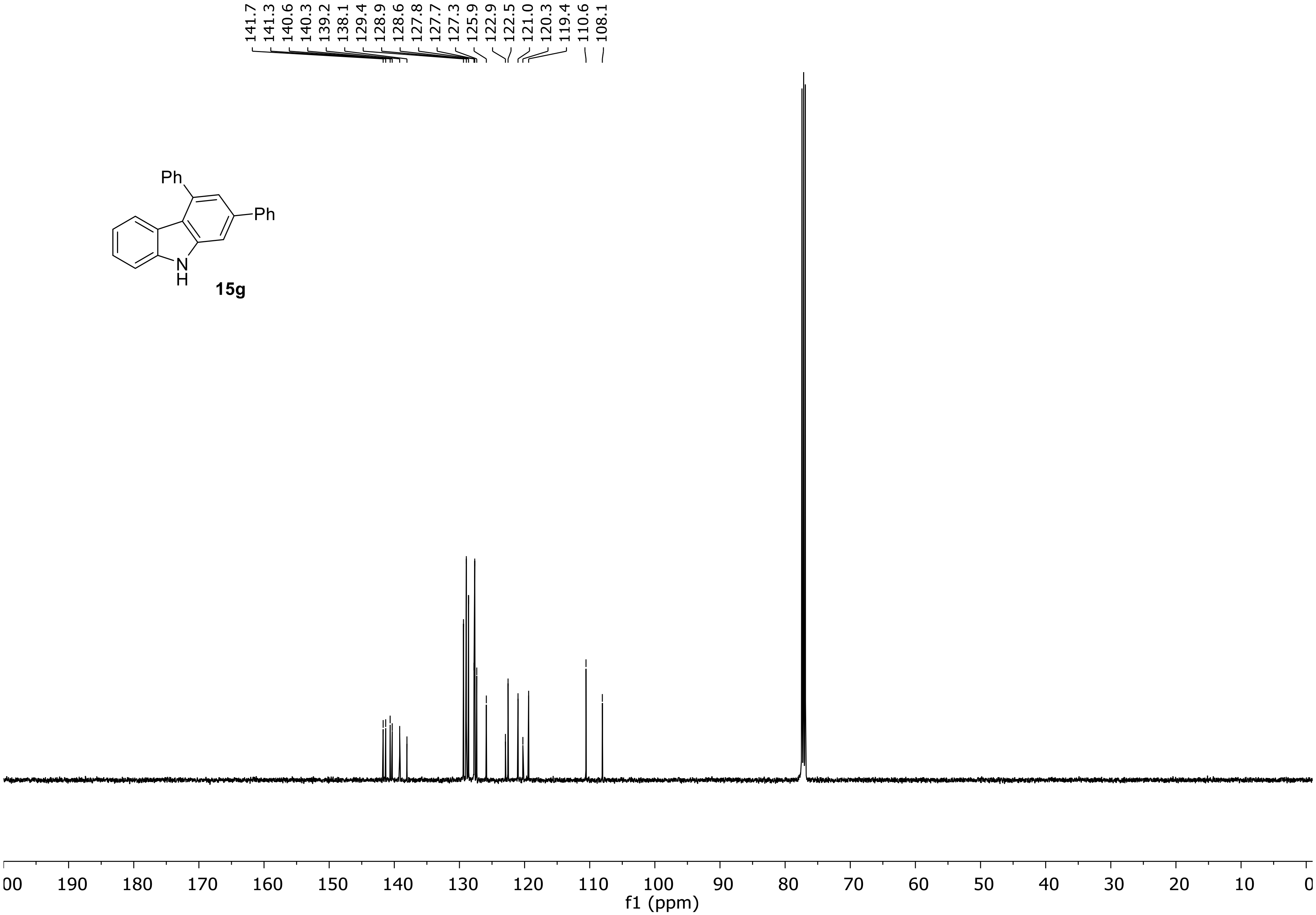

15g
